# Cross-Electrophile Coupling of *N*‑Hydroxyphthalimide Esters with Aryl Bromides Using an Inner-Sphere Homogeneous Reductant

**DOI:** 10.1021/jacs.5c18451

**Published:** 2026-01-08

**Authors:** Kasturi Ganguli, Alexandro R. Cruz, Justin B. Diccianni, Pablo García-Reynaga, Daniel J. Weix

**Affiliations:** † Department of Chemistry, UW-Madison, Madison, Wisconsin 53706, United States; ‡ Global Discovery Chemistry, Johnson & Johnson, 1400 mckean Road, Spring House, Pennsylvania 19477, United States; § Global Discovery Chemistry, Johnson & Johnson, 3210 Merryfield Row, San Diego, California 92121, United States

## Abstract

Cross-electrophile coupling of aryl bromides and iodides with *N*-hydroxyphthalimide (NHP) esters offers a valuable strategy for forming C­(sp^2^)–C­(sp^3^) bonds that has recently seen increasing use in organic synthesis. However, developing a broadly applicable method remains challenging, particularly with electron-rich aryl bromides, often requiring electrochemical tools or electron-rich NHP derivatives to succeed. Here, we report new conditions that help overcome key limitations in solving this challenge and provide a broad scope of reactivity without the need for specialized hardware. Key to success is the identification of a new general class of homogeneous reductants for XEC: 1,4-bis­(trialkylsilyl)­dihydropiperazines (Si-DHP). These reductants reduce nickel­(II) to nickel(0) faster than other common reductants and can be tuned by modifying the silicon substituents and the substitution pattern on the dihydropiperazine ring. We found that 1,4-bis­(trimethylsilyl)­dihydropiperazine (TMS-DHP) and 1,4-bis­(trimethylsilyl)-2,3,5,6-tetramethyldihydropiperazine (TMS-Me_4_DHP) are the most useful. The improved scope is demonstrated with a broad set of NHP esters and aryl bromides relevant to medicinal chemistry, including drug-like aryl bromide informers and comparisons to existing methodologies. Finally, these homogeneous reductants work well in a variety of solvents, addressing several long-standing issues for small- and large-scale applications.

## Graphical abstract

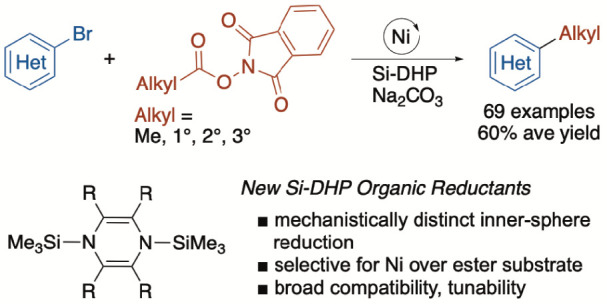

## 1 Introduction

Ni-catalyzed cross-electrophile coupling (XEC) has emerged as an attractive alternative to polarity-matched cross-coupling for C­(sp^2^)–C­(sp^3^) bond formation because of the generality of the approach^1–4^ and because molecules with increased sp^3^ character occupy more three-dimensional chemical space compared to flat, aromatic-rich structures, which can lead to improved biological and drug-like properties.^5–9^ In particular, the XEC of aryl halides with alkyl carboxylic acid *N*-hydroxyphthalimide esters (NHP esters^10–13^ ) has attracted broad interest from industrial researchers (Scheme 1B).^4,14–22^ This is because the starting materials are widely available and represent diverse chemical space (Scheme 1A).^22^ While aryl iodides may sometimes be used to bypass challenges in NHP ester coupling, they are limited in availability and can be an unreliable solution due to their higher reactivity and cost. There remains a need for reliable methods to couple alkanoyl NHP esters with less reactive aryl halides, particularly the more abundant and less activated aryl bromides (Scheme 1B).^17–19,23^

**Scheme 1:**
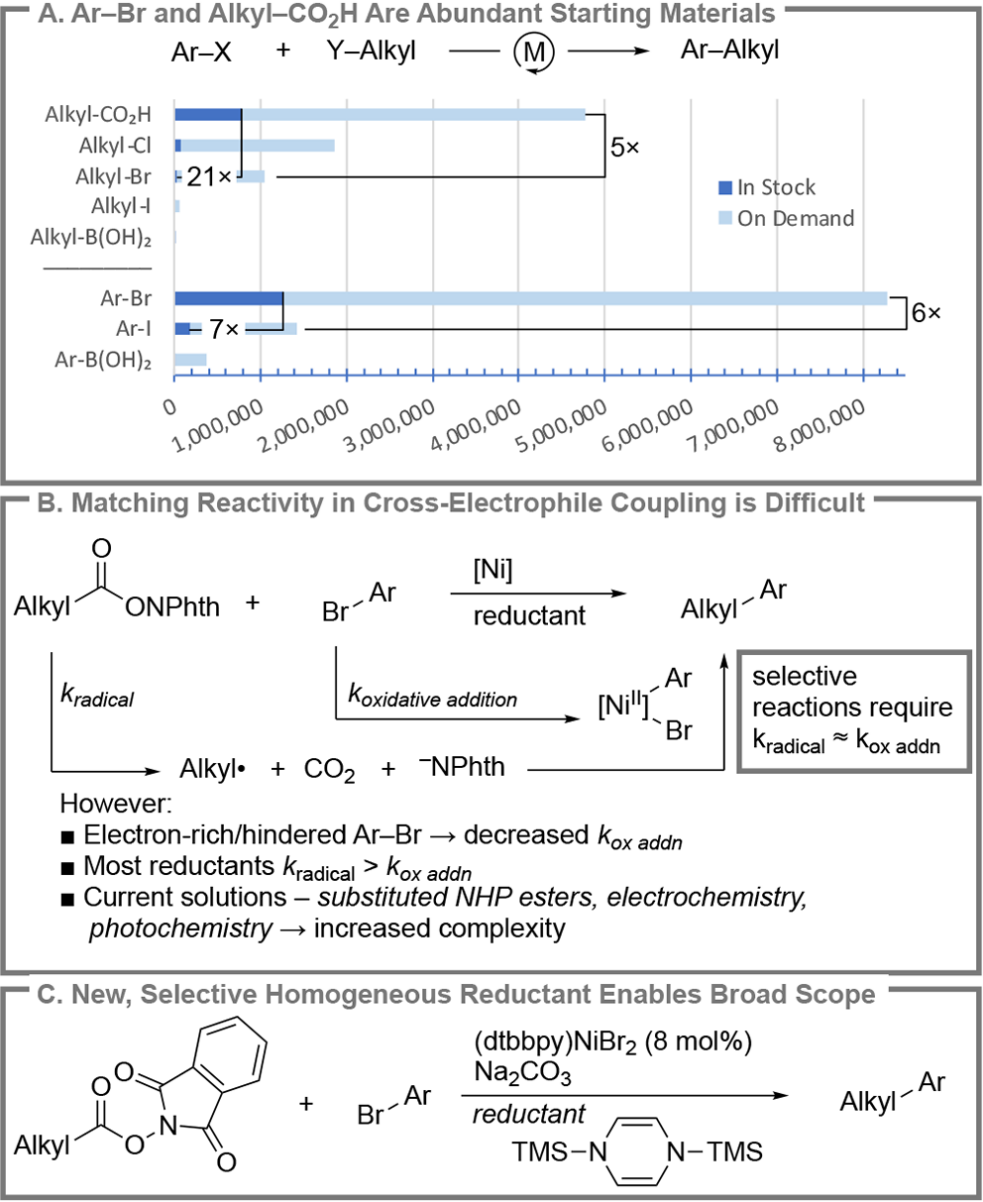
XEC of NHP Esters with Ar-Br

The key challenge that must be overcome is matching the rate of formation of the two intermediates: the alkyl radical (from NHP ester reduction) and the arylnickel­(II) intermediate (from nickel reduction and oxidative addition of the aryl bromide; Scheme 1B). Recently, progress has been made by slowing the rate of alkyl radical generation (via Ag-doped Ni cathode,^16,17,24,25^ electron-rich NHP derivatives,^18^ slow addition of NHP ester^26^) and speeding the rate of arylnickel­(II) generation (by increasing nickel concentration up to 20 mol % while using up to 30 equiv Zn^19,23,27,28^ ). Photoredox approaches with NHP esters have primarily focused on electron-poor aryl bromides.^29–32^ The use of an organic reductant, tetrakis­(dimethylamino)­ethylene (TDAE), has enabled coupling with vinyl bromides^33,34^ but not aryl bromides. The challenge of coupling with electron-rich aryl bromides has motivated approaches based upon two-step processes that are stoichiometric in nickel.^20,21^

We postulated that the challenge in balancing the rate of radical generation and aryl nickel formation arises from mismatched rates of reduction of NHP ester and nickel catalyst reduction. If this were the case, then outer-sphere reductants, the focus of all previous reports (Scheme 2A), are a poor choice because NHP esters and (dtbbpy)­Ni^II^X_2_ have similar reduction potentials (∼−1.7 V and −1.5 V vs Fc/Fc^+^, respectively).^18,35^ Indeed, the direct reduction of NHP esters has been reported to be fast and catalyzed by Lewis acids.^18,36,37^ One way to achieve the needed selectivity would be via inner-sphere mechanisms, where factors beyond the reduction potential could control selectivity. This led us to explore silylated dihydropyrazines (Si-DHPs, Scheme 2A and B).^38–40^ These formally antiaromatic compounds are readily prepared by lithium reduction of pyrazines and chlorosilanes.^41^ Furthermore, Mashima and coworkers reported that these reagents were excellent for the reduction of metal salts, despite their modest electrochemical reduction potentials, suggesting that an inner-sphere mechanism is operative (Scheme 2C).^42,43^ Although Mashima reported their use for aryl bromide homocoupling, arylation of aldehydes, and cyanation of aryl bromides,^44,45^ their application in XEC has not been reported in the literature.

**Scheme 2:**
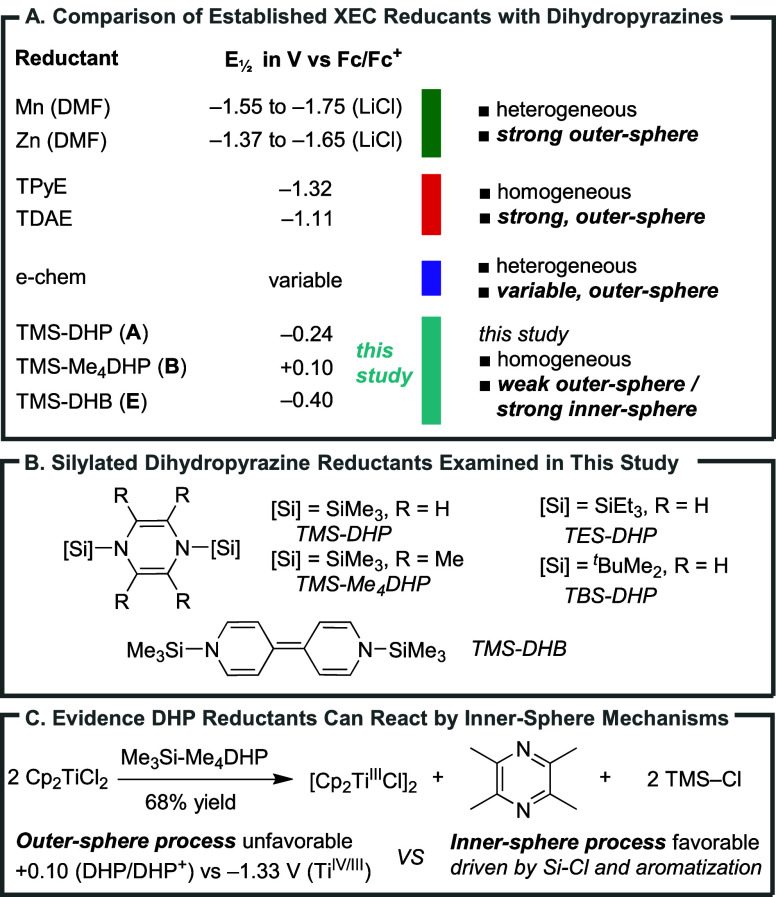
Si-DHP Reductants vs Established Reductants

We show herein that TMS-DHP and TMS-Me_4_DHP reductants enable a general solution to the coupling of NHP esters with aryl bromides without the need for specialized photochemical or electrochemical equipment (Scheme 1C). Mechanistic studies show that the Si-DHP reductants in nonpolar solvents are selective for nickel reduction over NHP ester reduction, enabling the enhanced scope.

## 2 Results and Discussion

After some preliminary studies using a variety of organic reductants, we found that the combination of TMS-DHP, nickel catalyst, and Na_2_CO_3_ was optimal for the coupling of ethyl 4-bromobenzoate (**1**) with the NHP ester of 4-phenylbutyrate (**2a**) (Table 1, entries 1 and 2). The reaction was complete in only 4 h and was quantitative. Na_2_CO_3_ is not necessary at higher temperatures but does accelerate the reaction overall (entries 1–4). We hypothesized that Na_2_CO_3_ could potentially activate the TMS-DHP reductant by attack at silicon^46^ or by reacting with TMS-Br (formed during the reduction step). We surveyed a number of potential activators/silyl transfer agents and found that Li_2_CO_3_, CsF, and DIPEA performed similarly to Na_2_CO_3_ (89–93% yields). NaOAc and K_2_CO_3_ provided slightly lower yields (71%) while NaOPiv, H_15_C_7_CO_2_Na, Cs_2_CO_3_, and CsOAc either gave no product or provided significantly lower yields (0–26% yield, Supporting Information, Section 3.1). Notably, we observed mixed anhydride formation from the NHP ester when soluble carboxylate bases were used (H_15_C_7_CO_2_Na and CsOAc). These mixed anhydrides would not act as radical donors, resulting in lower yields (see Supporting Information, Section 3.1). Both TMS-DHP and TMS-Me_4_DHP were equally effective (entry 5). More hindered silyl groups slowed the rate of reaction, with the order of reactivity being TMS > TES > TBS (entries 1, 6, and 7). For the reaction with TBS-DHP, substantial aryl bromide remained at the end of the reaction. The stronger 4,4′-bipyridine-based reductant, TMS-DHB, produced only 24% product, with the major products being aryl dimer and protodehalogenated arene (entry 8). Over-reduction of arylnickel species can lead to biaryl formation.^47^ Outer-sphere reductants were less effective. For example, consistent with literature reports,^34,48^ tetrakis (dimethylamino)­ethylene (TDAE), despite being a much stronger outer-sphere reductant than TMS-DHP (Scheme 2A), exhibited significantly lower reactivity (entry 9). Among heterogeneous reductants, Zn (82%) was superior to Mn (48%), although both suffered from incomplete conversion of aryl bromide and complete consumption of NHP ester despite the activated nature of the aryl bromide (entries 10 and 11).

**Table 1:**
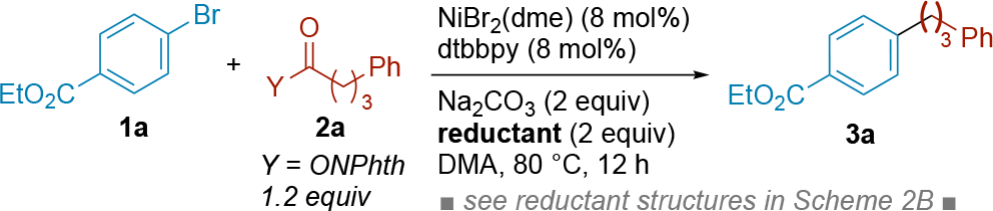
Initial Optimization of the Si-DHP-Driven XEC

Entry	Deviation from Conditions in Scheme^a^	Yield **3a** (%)^b^	Returned **1a** (%)^b^
1	TMS-DHP	100	0
2	TMS-DHP, rt, 4 h	100	0
3	TMS-DHP, omit Na_2_CO_3_	93	6
4	TMS-DHP, rt, 4 h, omit Na_2_CO_3_	38	58
5	TMS-Me_4_DHP	100	0
6	TES-DHP	82	18
7	TBS-DHP	10	77
8	TMS-DHB	24^c^	9
9	TDAE	8	90
10	Zn	82	18
11	Mn	48	25
12	TMS-DHP (1.1 equiv)	90	10
13	TMS-DHP, 5 mol % [Ni]/L	100	0
14	omit [Ni] or TMS-DHP	n.d.	100
15	TMS-DHP, omit dtbbpy	65^d^	0
16	TMS-DHP, THF for DMA	81^e^	0
17	THS-DHP, toluene for DMA	78^f^	0
18	Benchtop reaction under N_2_	99	0
19^g^	Benchtop reaction under air	85	12
20^h^	Benchtop reaction under air	94	0
21	TMS-DHP, 20 °C	94	0

a Reactions run on a 0.1 mmol scale in 160 μL DMA.

b GC yield.

c 25% Ar–Ar.

d 12% Ar–Ar and 6% Ar–H.

e 11% Ar–Ar.

f 9% Ar–Ar.

g Reaction after 24 h; reaction is incomplete at 12 h (71% **3a** with 30% recovered **1a**).

h 24 h with 4 equiv TMS-DHP.

We utilized the conditions in entry 1 as the basis for our scope studies (Scheme 3), but reasonable yields could be obtained with lower equivalents of TMS-DHP (1.1 equiv, entry 12) or lower catalyst loading (5 mol %, entry 13 and Supporting Information Section 3.7). Omitting nickel or reductant led to no conversion of the aryl bromide, but a 65% yield could be obtained without dtbbpy (entries 14 and 15; yield lower due to biaryl side product). Under the optimized conditions without the TMS-DHP reductant, there was no conversion of the NHP ester **2a**. However, with TMS-DHP present and no nickel, we observed complete conversion of the NHP ester **2a** in DMA, but with poor mass balance (only 12% alkyl-H). An additional advantage of working with homogeneous organic reductants lies in greater solvent compatibility, providing additional flexibility in synthesis and scale-up work.^49^ We found that a number of solvents work well for this transformation (1,4-dioxane, THF, toluene, MeCN, ^*i*^PrOAc, DMF, DMSO), with reactions in less polar solvents being slower (Table 1, entries 1, 16, 17, and Supporting Information Sections 3.6 and 5.8). Although TMS-DHP is unstable to air, reactions can be assembled on the benchtop without any impact on yield as long as dry, degassed solvents are used and the TMS-DHP solution is kept air-free (entries 18–20 and Supporting Information, Section 2.3). Indeed, even a reaction run under air in a sealed vial performed well, albeit more slowly (24 h vs 2 h). Our standard coupling partners (**1a** and **2a**) could be run at ambient temperature with minimal difference in yield (entry 21), but more deactivated substrates, such as 4-bromoanisole, required higher temperatures for high yields (see Scheme 3 and Supporting Information, Section 3.5). Finally, although TMS-DHP is easily synthesized, it is also commercially available.^50^

**Scheme 3:**
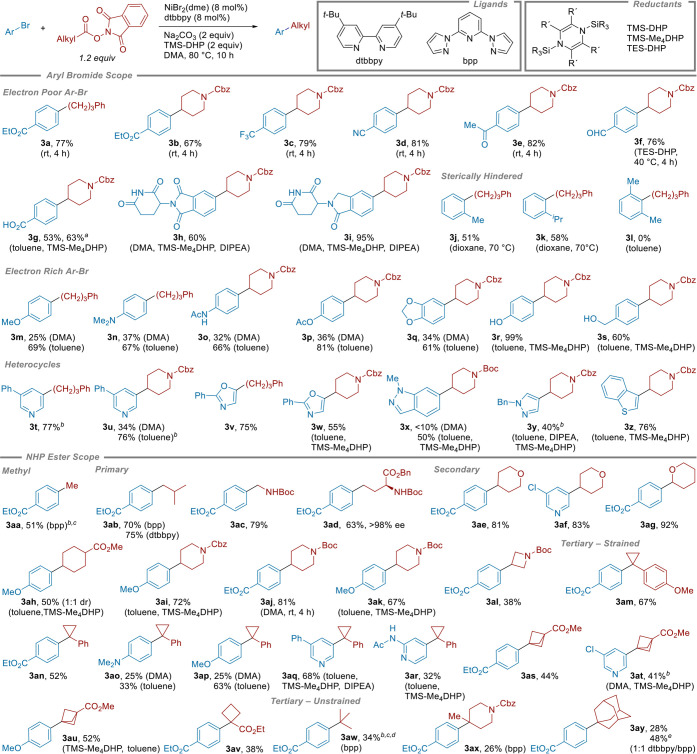
Substrate Scope for the Coupling of Aryl Bromides with N-Hydroxyphthalimide Esters Using Si-DHP Reductants^a^ [Fn sch3-fn2] ^c^ ^d^ ^e^ [Fn sch3-fn6]

i Reactions were run on a 0.1 mmol scale in 200 μL of DMA at 80 °C for 10 h unless otherwise specified. Isolated yields were obtained after purification
a Bis­(trimethylsilyl)­acetamide (BSA, 2 equiv) was added to the aryl bromide, and the mixture was stirred in toluene (0.1 mL) for 15 min before the addition of the other reagents.
b
^1^H NMR yield.
c 1.5 equiv of the NHP ester.
d The ratio of pdt/isomerized pdt (**3aw**/**3ab**) was 34:1.
e 20 mol % NiBr_2_(dme), 10 mol % dtbbpy and 10 mol % bpp were used instead of 8 mol % NiBr_2_(dme), 4 mol % dtbbpy, and 4 mol % bpp.

Our optimal conditions are effective for both electron-poor (**3a**-**3i**) and electron-rich aryl bromides (**3m**-**3s**) (Scheme 3), and we found that the use of TMS-Me_4_DHP and dioxane or toluene as solvents provided even higher yields for aryl bromides that are deactivated toward oxidative addition (e.g., electron-donating groups in *para*- or *ortho*-positions; see Supporting Information, Sections 3.8 and 5.8.2 for details). The results with electron-rich aryl bromides are particularly noteworthy because their low reactivity has often required higher catalyst loadings or the use of iodoarenes instead. For example, our results with bromoanisole are better than those reported previously (**3ah**, 50% vs 39% yield)^17^ and match those we reported with iodoanisole (**3au**).^18^ Other primary and secondary NHP esters could be coupled with anisyl bromide (**3m**, **3ai**, and **3ak**) in even higher yields (67–72%). The more electron-rich anilines could be coupled (**3n**, **3o**), albeit in slightly lower yields (66–67%, see also discussion of **4a** and **4h**). For *ortho*-substitution, 2-cumyl bromide could be coupled (**3k**, 58% yield), but 1-bromo-2,6-dimethylbenzene was unreactive (**3l**).^51^

Heteroaryl halides, which are challenging substrates for XEC, worked well, including pyridine, oxazole, and pyrazole halides (**3t**–**3z**). Under our optimal conditions, *N*-benzyl-4-bromopyrazole was coupled in 40% yield (**3y**), a substantial improvement over literature examples using higher nickel loading (20 mol %).^17,19,52^ In another example, alkylated pyridine **3af** could be formed in 83% yield, vs the 56% yield reported previously.^17^

We found the functional group tolerance of reactions with these new reductants to be as broad as those of reactions with Zn or Mn. Although Ni/Si-DHP has been reported for aldehyde arylation^44^ and C–CN bond cleavage reactions,^45^ aldehyde (**3f**, 76%), ketone (**3e**, 82%), and nitrile groups (**3d**, 81%) are all tolerated. Consistent with literature reports on the reactivity of TMS-DHP with aldehydes,^46^ we found that the aldehyde substrate gave a higher yield when TES-DHP was used instead of TMS-DHP (34% yield vs 76% yield), perhaps due to the decreased Lewis acidity of TES-X vs TMS-X. As with Zn and Mn reductants, acidic N–H and O–H bonds are tolerated (**3g, 3h, 3i, 3o, 3r, 3s, 3ac**, **3ad**). The free carboxylic acid in **3g** was also tolerated (53% yield), but in situ silylation using *N*,*O*-bis­(trimethylsilyl)­acetamide (BSA^53^) enabled a 63% yield.

Derivatives of thalidomide and lenalidomide are of high interest in medicinal chemistry because they can act as ligands for E3 ligases in PROTACs^54–61^ but are challenging substrates due to the acidity and base sensitivity of glutarimide derivatives. High yields could be obtained for both bromothalidomide (**3h**, 60% yield) and bromolenalidomide (**3i**, 95% yield). To put this result into context, the Boc-protected version of product **3i** has been synthesized by a variety of approaches in the context of PROTACs discovery efforts, including photochemical XEC with the NHP ester^62^ (69%) or the alkyl bromide^63^ (87%, 3 equiv alkyl-Br) and thermal XEC with the alkyl tosylate^64,65^ (56–57%). Inspired by the work of Krumb and coworkers, we found that a one-pot XEC (eq 1) and amide-bond formation (eq 2) also proceeded in good yield, providing a convenient route to making PROTAC derivative **3az**.^62^ By omitting base/activator, the Boc group could be deprotected during the XEC step (eq 1), presumably by the TMS-Br generated during nickel catalyst turnover. The deprotected intermediate (1) was then telescoped into the next step to form **3az** (Supporting Information, Section 2.7). *Safety Note: Derivatives of thalidomide and lenalidomide can have serious effects on human health at very low exposure levels. These derivatives should be handled with the same level of caution, using suitable safety procedures.*

1

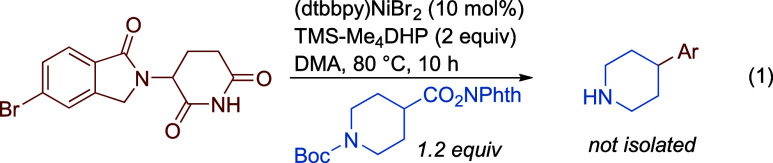



2

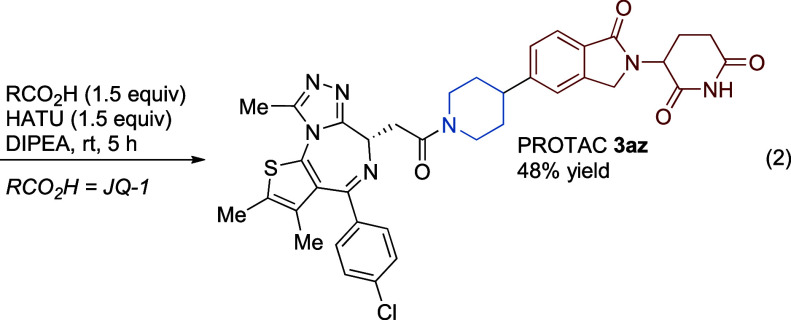




On the alkyl side, a broad range of NHP esters bearing methyl, primary, secondary, strained-ring tertiary, adamantyl, and even unstrained tertiary substituents could be coupled. Standard conditions sufficed for 1°, 2°, and strained-ring 3° NHP esters, but methyl, adamantyl, and unstrained tertiary NHP esters required adjustment of the catalyst. Inspired by the work of Sevov,^66^ we found that reactions with bis­(pyrazole)­pyridine (bpp) or a mixture of dtbbpy and bpp as the catalyst provided promising yields (**3aw**, **3ax**, **3ay**) and avoided biaryl formation. Of note, we observed <5% (3%) isomerization of the *tert*-butyl group in **3aw** to the rearranged product **3ab**, a known challenge in *tert*-alkyl coupling reactions of all types.^67^ Product **3aw** was formed in 34% yield, on par with previous electrochemical approaches.^25,66^ Adamantane carboxylic acid NHP ester, a challenging substrate,^17,25^ could be coupled to form **3ay** in up to 47% yield. For coupling with bicyclopentane, a phenyl ring isotere, pyridyl derivative **3at** could be obtained in 41% yield.^25^ NHP esters with α-stabilizing groups, such as oxygen (**3ag**) or nitrogen (**3ac**), were also compatible substrates, whereas the corresponding halides, alcohols, or amines are less convenient because of low stability. The synthesis of unnatural amino acids from more abundant natural amino acids is a valuable use of XEC chemistry.^11,24,52,68,69^ We found that homophenylalanine derivative **3ad** could be made in 63% yield and without racemization (>98% *ee*).

To further test the generality of our method, particularly with challenging, medicinally relevant substrates, we examined the Merck “informer” aryl bromide set (**X1**–**X13**) against the NHP ester of *N*-Cbz 4-pipecolic acid (Scheme 4).^20,70–74^ As a testament to the generality of the Si-DHP approach, 10 of the 13 Ar–Br informers underwent successful coupling (we did not test the iodide and chloride informers). Significant improvements in the yields of **4a** and **4h** were observed upon switching from our initial optimized conditions to toluene and TMS-Me_4_DHP, consistent with our initial finding that electron-rich aryl bromides require a slowing of NHP ester consumption. We found that moving to 4-methyl-*N*-hydroxyphthalimide esters (^Me^NHP) further slowed radical generation^18^ and this effect was additive with the solvent and reductant effects. While we did not examine this more specialized activating group with all of the informers, significant improvements in yield were observed for **4a** (40% in toluene with NHP to 95% yield in toluene/DMA with ^Me^NHP) and **4h** (45% in toluene with NHP to 81% yield in toluene with ^Me^NHP). For informer **X2**, we demonstrated coupling with 1°, 2°, strained-ring 3°, and methyl with similarly high yields (**4b**–**4e**). Informers **X7**, **X9,** and **X11** did not form detectable product, and we attribute this to the known reactivity of nickel with isoxazoles (**X9**)^75^ and electronic/steric deactivation (**X7**, **X11**).^51^ Notably, these three informers failed for 2° carboxylic acid coupling partners even under the best-reported stoichiometric^20^ and metallaphotoredox conditions^71^ (see inset).^76^ Informer **X10** is the second most challenging one (after **X11**), perhaps due to potential catalyst chelation by the latent 8-hydroxyquinoline moiety.^77^ We found that in situ silylation of the phenol using BSA^53^ allowed for a 34% yield (**4m**).

**Scheme 4:**
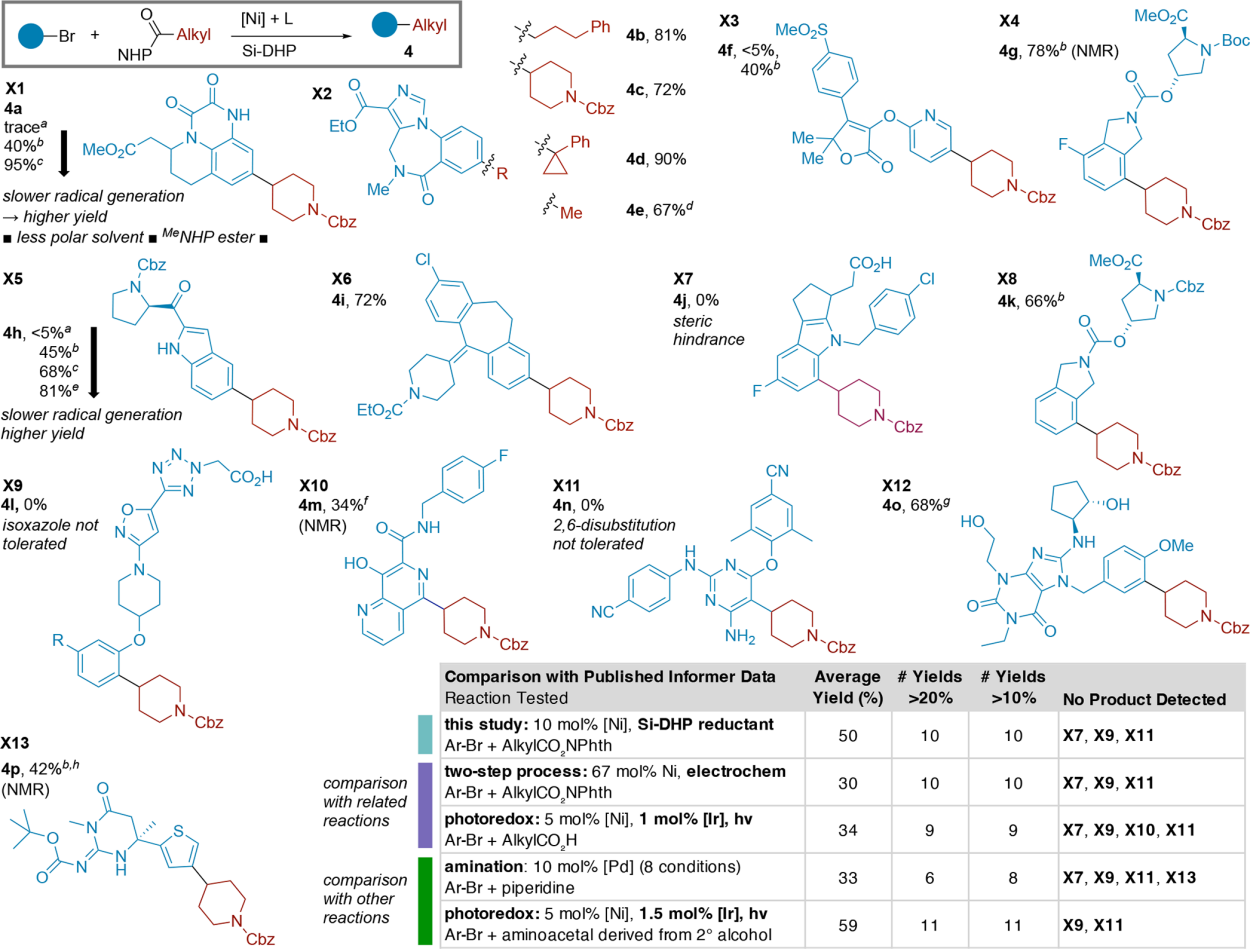
Coupling of 2° NHP Ester with Merck Informer Aryl Bromide Collection and Comparison to Literature^a^ ^b^ ^c^ ^d^ ^e^ ^f^ ^g^ ^h^

a Reactions were run on a 0.05 or 0.10 mmol scale with 10 mol % NiBr_2_(dme)/dtbbpy, 2 equiv Na_2_CO_3_, 2 equiv of TMS-DHP reductant in DMA (0.25 M) at 80 °C for 15 h unless otherwise specified. Isolated yields were obtained after purification.
b Toluene instead of DMA, and TMS-Me_4_DHP instead of TMS-DHP.
c DMA/Toluene (1:1) instead of DMA, TMS-Me_4_DHP instead of TMS-DHP, and ^Me^NHP instead of NHP ester.
d A 1:1 ratio of dtbbpy/bpp (10 mol % total) was used instead of dtbbpy, 2.0 equiv of NHP ester instead of 1.2 equiv.
e Toluene instead of DMA, TMS-Me_4_DHP instead of TMS-DHP, and ^Me^NHP instead of NHP ester.
f Bis­(trimethylsilyl)­acetamide (2.0 equiv) was used, along with toluene instead of DMA, and TMS-Me_4_DHP instead of TMS-DHP.
g Toluene instead of DMA, TMS-Me_4_DHP instead of TMS-DHP, and DIPEA was used instead of Na_2_CO_3_.
h After the reaction was completed, (Boc)_2_O (10.0 equiv) was added to reprotect any deprotected guanidine.

For reference, we collected data from the relatively small number of publications reporting yields for the full Ar–Br informer set (Scheme 4, inset, and Supporting Information Section 4). Among reactions for coupling carboxylic acid derivatives, these conditions offer some improvement while avoiding the need for Ir photocatalysts, stoichiometric amounts of nickel, and/or specialized photochemical or electrochemical equipment. As a reference, all three sets of decarboxylative coupling conditions compare favorably to often-used Pd-catalyzed C–N bond-forming reactions with the same set of informers. Among all C­(sp^2^)–C­(sp^3^) bond-forming reactions, the nickel metallaphotoredox coupling of alcohol-derived amide acetals with aryl bromides^74^ has reported better results (table in Scheme 4), but worse results have been reported with zinc and boron reagents (Supporting Information Section 4).^70,77^

To better understand why Si-DHPs are successful in the present reaction, we elected to study their reactivity in stoichiometric reactions with a Ni catalyst and organic substrates. Given that reactions in DMA proceeded very rapidly, we chose to study the reaction conditions in toluene. Collectively, these studies show that the Si-DHP reductants are fast at reducing the nickel catalyst but are slow to react with organic substrates (Scheme 5). First, TMS-DHP by itself does not react even with an electron-poor aryl bromide after 18 h. However, in the presence of nickel, quantitative conversion to biaryl is observed within an hour (Scheme 5A).^44^ Second, Si-DHP reductants are slow to react with NHP esters in toluene at 80 °C (as followed by GC and in situ NMR), but in the presence of nickel, complete consumption is observed within one hour (see Supporting Information, Section 5.2.1). Consistent with our observed solvent effects, TMS-DHP did reduce NHP esters in DMA, shedding light on why reactions in less polar solvents resulted in improved yields with less reactive aryl bromides (Supporting Information sections 5.2.2 and 5.2.3). Also, contrary to previous reports by Reisman and Yang,^34^ TMS-Br was found to inhibit the reduction of NHP ester **2a** by the TMS-DHP reductant (Supporting Information, Section 5.2.3). Third, TMS-DHP and TMS-Me_4_DHP reduce nickel within 10 min in toluene, which is visualized as nickel black precipitation.

**Scheme 5:**
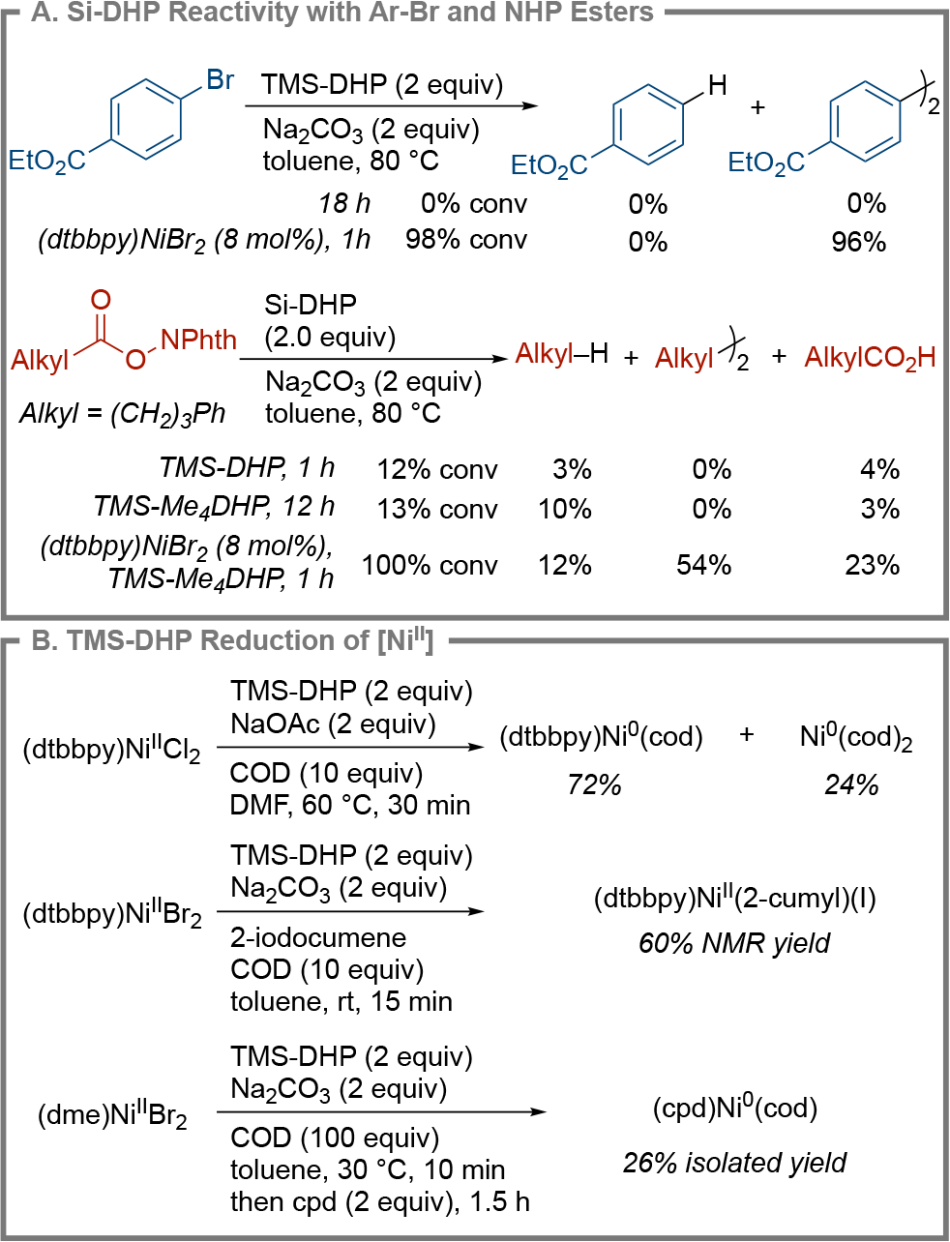
Studies on Si-DHP Reduction Reactions^a^

a cpd = 2,3,4,5-tetraphenylcyclopentene-1-one. See Supporting Information Section 5.

Confirmation of the formation of Ni^0^ was achieved using several approaches (Scheme 5B and Supporting Information sections 5.3 and 5.4) revealing that Si-DHP reagents act faster than metallic reductants. If the reduction is run in DMF, a 96% NMR yield of [Ni^0^] (a combination of (dtbbpy)­Ni­(cod) and Ni­(cod)_2_) could be obtained. In the presence of 2-iodocumene, reduction in toluene produced (dtbbpy)­Ni^II^(2-cumyl)­(I) in 60% yield after 15 min. Finally, following the work of Engle, we could trap nickel(0) with tetraphenylcyclopentadienone (CPD^H^) to form the known (cod)­Ni^0^(CPD^H^) in 26% yield.^78^ Notably, a reduction to Ni^0^ was not observed for Zn or Mn in toluene. In DMF, Zn was slower than TMS-DHP (64% yield vs 96% yield of Ni(0) at 30 min), even when LiCl was added to enhance the reduction rate.^79–81^ UV–vis time-course experiments established that the relative rate of reduction of Ni^II^ to Ni^0^ by Si-DHP reagents in DMA followed the order TMS-DHB ≫ TMS-DHP > TMS-Me_4_DHP ∼ TES-DHP (Section 5.7, Supporting Information).

The data in Scheme 5 suggest that the Si-DHP reductants reduce the nickel catalyst, and the reduced nickel catalyst is responsible for both the oxidative addition of the aryl halide and radical formation. Stoichiometric reactions of NHP ester with (dtbbpy)­Ni^0^(cod) provided high yields of the dialkyl product (66% yield, Supporting Information, Section 5.5), demonstrating that nickel(0) activates NHP esters efficiently. This process appears to occur at about the same rate regardless of the nature of the radical that is formed (Supporting Information, Section 5.8.2). The reaction of (dtbbpy)­Ni^II^(Ar)­(NPhth) with an NHP ester formed the cross-product in high yield (73% yield, Section 5.6.2) in the presence of the TMS-DHP reductant, suggesting that [Ni^I^] ^82–85^ or [Ni^0^] species are responsible for radical formation. Only a trace amount of the product was formed in the absence of the reductant.

## 3 Conclusions

In conclusion, our data show that dihydropyrazine-based organosilicon reductants offer notable advantages for decarboxylative C­(sp^3^)-C­(sp^2^) cross-electrophile coupling. In particular, reactivity with electron-rich aryl bromides, a long-standing challenge in the field, without the need for electrochemistry or photochemistry, is a useful advance. The tunability of the Si-DHP reductants, along with the inner-sphere nature of the reduction mechanism, offers many opportunities for cross-electrophile coupling and other reductive reactions by altering the relative rates of catalyst and substrate reduction compared to more typical outer-sphere reductants. Finally, the homogeneous conditions and solvent flexibility should enable applications across a range of scales, from small-scale HTE to scale-up reactions. Exploration of these possibilities, as well as application of these reductants to other cross-electrophile reactions, is under study and will be presented in due course.

## Supplementary Material



## Data Availability

The data underlying this study are available in the published article and its online Supporting Information.

## Notes

Supplementary optimization data, full experimental procedures, characterization data for all new compounds, copies of ^1^H and ^13^C NMR spectra (PDF)

## Abbreviations

DHP: dihydropyrazine
NHP: *N*-hydroxyphthalimide
HTE: high-throughput experimentation

## References

[1] Goldfogel, M. J. ; Huang, L. ; Weix, D. J. Cross-Electrophile Coupling: Principles and New Reactions. In Nickel Catalysis in Organic Synthesis: Methods and Reactions, Ogoshi, S. , Ed., Wiley, 2020, pp 183−222 10.1002/9783527813827.ch9.

[2] Diccianni J. B., Diao T. (2019). Mechanisms of Nickel-Catalyzed
Cross-Coupling Reactions. Trends Chem..

[3] Wang X., Dai Y., Gong H. (2016). Nickel-Catalyzed
Reductive Couplings. Top. Curr. Chem..

[4] Ehehalt L. E., Beleh O. M., Priest I. C., Mouat J. M., Olszewski A. K., Ahern B. N., Cruz A. R., Chi B. K., Castro A. J., Kang K. (2024). Cross-Electrophile Coupling:
Principles, Methods, and
Applications in Synthesis. Chem. Rev..

[5] Lovering F., Bikker J., Humblet C. (2009). Escape from
Flatland: Increasing
Saturation as an Approach to Improving Clinical Success. J. Med. Chem..

[6] Roughley S. D., Jordan A. M. (2011). The Medicinal Chemist’s Toolbox: An Analysis
of Reactions Used in the Pursuit of Drug Candidates. J. Med. Chem..

[7] Dombrowski A. W., Gesmundo N. J., Aguirre A. L., Sarris K. A., Young J. M., Bogdan A. R., Martin M. C., Gedeon S., Wang Y. (2020). Expanding
the Medicinal Chemist Toolbox: Comparing Seven C­(sp^2^)–C­(sp^3^) Cross-Coupling Methods by Library Synthesis. ACS Med. Chem. Lett..

[8] Lovering F. (2013). Escape from
Flatland 2: Complexity and promiscuity. MedChemComm.

[9] Monteleone S., Fuchs J. E., Liedl K. R. (2017). Molecular
Connectivity Predefines
Polypharmacology: Aliphatic Rings, Chirality, and sp3 Centers Enhance
Target Selectivity. Front. Pharmacol..

[10] Cornella J., Edwards J. T., Qin T., Kawamura S., Wang J., Pan C.-M., Gianatassio R., Schmidt M., Eastgate M. D., Baran P. S. (2016). Practical Ni-Catalyzed
Aryl–Alkyl Cross-Coupling
of Secondary Redox-Active Esters. J. Am. Chem.
Soc..

[11] Huihui K. M. M., Caputo J. A., Melchor Z., Olivares A. M., Spiewak A. M., Johnson K. A., DiBenedetto T. A., Kim S., Ackerman L. K. G., Weix D. J. (2016). Decarboxylative Cross-Electrophile Coupling of N-Hydroxyphthalimide
Esters with Aryl Iodides. J. Am. Chem. Soc..

[12] Lu X., Xiao B., Liu L., Fu Y. (2016). Formation of C­(sp 3
)–C­(sp 3) Bonds through Nickel-Catalyzed Decarboxylative Olefin
Hydroalkylation Reactions. Chem. -Eur. J..

[13] Parida S. K., Mandal T., Das S., Hota S. K., De Sarkar S., Murarka S. (2021). Single Electron Transfer-Induced
Redox Processes Involving
N-(Acyloxy)­phthalimides. ACS Catal..

[14] Li H., Breen C. P., Seo H., Jamison T. F., Fang Y.-Q., Bio M. M. (2018). Ni-Catalyzed Electrochemical
Decarboxylative C–C
Couplings in Batch and Continuous Flow. Org.
Lett..

[15] Watanabe E., Chen Y., May O., Ley S. V. (2020). A Practical Method
for Continuous Production of sp3-Rich Compounds from (Hetero)­Aryl
Halides and Redox-Active Esters. Chem. -Eur.
J..

[16] Harwood S. J., Palkowitz M. D., Gannett C. N., Perez P., Yao Z., Sun L., Abruña H. D., Anderson S. L., Baran P. S. (2022). Modular
terpene synthesis enabled by mild electrochemical couplings. Science.

[17] Palkowitz M. D., Laudadio G., Kolb S., Choi J., Oderinde M. S., Ewing T. E.-H., Bolduc P. N., Chen T., Zhang H., Cheng P. T. W. (2022). Overcoming Limitations in Decarboxylative Arylation
via Ag–Ni Electrocatalysis. J. Am. Chem.
Soc..

[18] Salgueiro D. C., Chi B. K., Guzei I. A., García-Reynaga P., Weix D. J. (2022). Control of Redox-Active Ester Reactivity Enables a
General Cross-Electrophile Approach to Access Arylated Strained Rings. Angew. Chem., Int. Ed..

[19] DeCicco E. M., Berritt S., Knauber T., Coffey S. B., Hou J., Dowling M. S. (2023). Decarboxylative Cross-Electrophile Coupling of (Hetero)­Aromatic
Bromides and NHP Esters. J. Org. Chem..

[20] Dinh L. P., Starbuck H. F., Hamby T. B., LaLama M. J., He C. Q., Kalyani D., Sevov C. S. (2024). Persistent
organonickel complexes
as general platforms for Csp2–Csp3 coupling reactions. Nat. Chem..

[21] Odena C., Santiago T. G., Linares M. L., Castellanos-Blanco N., McGuire R. T., Chaves-Arquero B., Alonso J. M., Diéguez-Vázquez A., Tan E., Alcázar J. (2024). Late-Stage C­(sp^2^)–C­(sp^3^) Diversification via Nickel Oxidative
Addition Complexes. J. Am. Chem. Soc..

[22] Morvan J., Tang B., Ryabchuk P., Renders E., Last S., Van Eynde L., Bartkowiak K., Buijnsters P. J. J. A., Jones A. X., Carvalho M.-A. (2025). Enabling electrochemical,
decarboxylative C­(sp^2^)–C­(sp^3^) cross-coupling
for parallel medicinal chemistry. Eur. J. Med.
Chem..

[23] Michel N. W. M., Gabbey A. L., Edjoc R. K., Fagbola E., Hughes J. M. E., Campeau L.-C., Rousseaux S. A. L. (2024). Nickel-Catalyzed
Reductive Arylation
of Redox Active Esters for the Synthesis of α-Aryl Nitriles:
Investigation of a Chlorosilane Additive. J.
Org. Chem..

[24] Laudadio G., Neigenfind P., Chebolu R., Blasczak V. D., Maddirala S. J., Palkowitz M. D., Bolduc P. N., Nicastri M. C., Puthukanoori R. K., Paraselli B. R. (2024). Synthesis of Unnatural Amino Acids via Ni/Ag
Electrocatalytic Cross-Coupling. Org. Lett..

[25] Laudadio G., Neigenfind P., Péter Á., Rubel C. Z., Emmanuel M. A., Oderinde M. S., Ewing T. E.-H., Palkowitz M. D., Sloane J. L., Gillman K. W. (2024). Nickel-Electrocatalytic
Decarboxylative Arylation to Access Quaternary Centers. Angew. Chem., Int. Ed..

[26] Sun J., Wang S., Harper K. C., Kawamata Y., Baran P. S. (2025). Stereoselective
amino alcohol synthesis via chemoselective electrocatalytic radical
cross-couplings. Nat. Chem..

[27] West M. S., Gabbey A. L., Huestis M. P., Rousseaux S. A. L. (2022). Ni-Catalyzed
Reductive Cross-Coupling of Cyclopropylamines and Other Strained Ring
NHP Esters with (Hetero)­Aryl Halides. Org. Lett..

[28] Choi E. S., Rousseaux S. A. L., Huestis M. P. (2024). Nickel-Catalyzed Synthesis of Benzylamines
from (Hetero)­aryl Halides and Glycine-Derived N-Hydroxyphthalimide
Esters. Synlett.

[29] Matsuo B., Granados A., Levitre G., Molander G. A. (2023). Photochemical Methods
Applied to DNA Encoded Library (DEL) Synthesis. Acc. Chem. Res..

[30] Kammer L. M., Badir S. O., Hu R.-M., Molander G. A. (2021). Photoactive
electron
donor–acceptor complex platform for Ni-mediated C­(sp^3^)–C­(sp^2^) bond formation. Chem. Sci..

[31] Escolano M., Cabrera-Afonso M. J., Ribagorda M., Badir S. O., Molander G. A. (2022). Nickel-Mediated
Synthesis of Non-Anomeric C-Acyl Glycosides through Electron Donor–Acceptor
Complex Photoactivation. J. Org. Chem..

[32] Xie K. A., Bednarova E., Joe C. L., Sherwood T. C., Welin E. R., Rovis T. (2024). A Unified
Method for Oxidative and Reductive Decarboxylative Arylation
with Orange Light-Driven Ir/Ni Metallaphotoredox Catalysis. J. Am. Chem. Soc..

[33] Suzuki N., Hofstra J. L., Poremba K. E., Reisman S. E. (2017). Nickel-Catalyzed
Enantioselective Cross-Coupling of N-Hydroxyphthalimide Esters with
Vinyl Bromides. Org. Lett..

[34] Turro R. F., Wahlman J. L. H., Tong Z. J., Chen X., Yang M., Chen E. P., Hong X., Hadt R. G., Houk K. N., Yang Y.-F. (2023). Mechanistic
Investigation of Ni-Catalyzed Reductive
Cross-Coupling of Alkenyl and Benzyl Electrophiles. J. Am. Chem. Soc..

[35] Lin Q., Dawson G., Diao T. (2021). Experimental Electrochemical Potentials
of Nickel Complexes. Synlett.

[36] Ye Y., Chen H., Sessler J. L., Gong H. (2019). Zn-Mediated Fragmentation
of Tertiary Alkyl Oxalates Enabling Formation of Alkylated and Arylated
Quaternary Carbon Centers. J. Am. Chem. Soc..

[37] Wang J., Lundberg H., Asai S., Martín-Acosta P., Chen J. S., Brown S., Farrell W., Dushin R. G., O’Donnell C. J., Ratnayake A. S. (2018). Kinetically guided radical-based
synthesis of C­(sp^3^)-C­(sp^3^) linkages on DNA. Proc. Natl. Acad. Sci. U. S. A..

[38] Sulzbach R. A., Iqbal A. F. M. (1971). 1,4-Bis­(trimethylsilyl)-1,4-dihydropyrazine by Reductive
Silylation of Pyrazine. Angew. Chem., Int. Ed..

[39] Lichtblau A., Ehlend A., Hausen H.-D., Kaim W. N. (1995). N′ -Disilylated
1,4-Dihydropyrazines: Organosilyl Substitution Reactions, Structural
Effects of Steric Hindrance, and Electron Exchange with C60. Chem. Ber..

[40] Kaim W. (1983). Effects of
cyclic 8π-electron conjugation in reductively silylated nitrogen
heterocycles. J. Am. Chem. Soc..

[41] Parsutkar M. M., Moore C. E., RajanBabu T. V. (2022). Activator-free
single-component Co­(i)-catalysts
for regio- and enantioselective heterodimerization and hydroacylation
reactions of 1,3-dienes. New reduction procedures for synthesis of
[L]­Co­(i)-complexes and comparison to in situ generated catalysts. Dalton Trans..

[42] Tsurugi H., Mashima K. (2019). Salt-Free Reduction of Transition Metal Complexes by
Bis­(trimethylsilyl)­cyclohexadiene, -dihydropyrazine, and −4,4′-bipyridinylidene
Derivatives. Acc. Chem. Res..

[43] Saito T., Nishiyama H., Tanahashi H., Kawakita K., Tsurugi H., Mashima K. (2014). 1,4-Bis­(trimethylsilyl)-1,4-diaza-2,5-cyclohexadienes
as Strong Salt-Free Reductants for Generating Low-Valent Early Transition
Metals with Electron-Donating Ligands. J. Am.
Chem. Soc..

[44] Yurino T., Ueda Y., Shimizu Y., Tanaka S., Nishiyama H., Tsurugi H., Sato K., Mashima K. (2015). Salt-Free Reduction
of Nonprecious Transition-Metal Compounds: Generation of Amorphous
Ni Nanoparticles for Catalytic C–C Bond Formation. Angew. Chem., Int. Ed..

[45] Ueda Y., Tsujimoto N., Yurino T., Tsurugi H., Mashima K. (2019). Nickel-catalyzed
cyanation of aryl halides and triflates using acetonitrile via C–CN
bond cleavage assisted by 1,4-bis­(trimethylsilyl)-2,3,5,6-tetramethyl-1,4-dihydropyrazine. Chem. Sci..

[46] Tsurugi H., Matsuno M., Kawakami T., Mashima K. (2022). Pyrazine Alkylation
with Aldehydes and Haloalkanes Using N, N’–Bis­(trimethylsilyl)–1,4–dihydropyrazine. Eur. J. Org. Chem..

[47] Romero-Arenas A., Popescu M. V., Goetz M. K., Bhatnagar R., Goljani H., Punchihewa B. T., Sanders K. M., Guzei I. A., Rafiee M., Weix D. J. (2025). Reductively Induced
Aryl Transmetalation: An Alternative Catalytically Relevant Ni-Catalyzed
Biaryl Coupling Mechanism. J. Am. Chem. Soc..

[48] Charboneau D. J., Huang H., Barth E. L., Germe C. C., Hazari N., Mercado B. Q., Uehling M. R., Zultanski S. L. (2021). Tunable
and Practical Homogeneous Organic Reductants for Cross-Electrophile
Coupling. J. Am. Chem. Soc..

[49] Anka-Lufford L. L., Huihui K. M. M., Gower N. J., Ackerman L. K. G., Weix D. J. (2016). Nickel-Catalyzed
Cross-Electrophile Coupling with Organic Reductants in Non-Amide Solvents. Chem. -Eur. J..

[50] Sinocompound sells TMS-DHP (SC-6228) and we found this material to provide yields equal to those with material we made.

[51] Wu T., Castro A. J., Ganguli K., Rotella M. E., Ye N., Gallou F., Wu B., Weix D. J. (2025). Cross-Electrophile
Coupling to Form Sterically Hindered C­(sp^2^)–C­(sp^3^) Bonds: Ni and Co Afford Complementary Reactivity. J. Am. Chem. Soc..

[52] Bombonato E., Fasano V., Pecorari D., Fornasari L., Castagnini F., Marcaccio M., Ronchi P. (2024). Electrochemical Synthesis
of Unnatural Amino Acids Embedding 5- and 6-Membered Heteroaromatics. ACS Omega.

[53] Klebe J. F., Finkbeiner H., White D. M. (1966). Silylations with Bis­(trimethylsilyl)­acetamide,
a Highly Reactive Silyl Donor. J. Am. Chem.
Soc..

[54] Sosič I., Bricelj A., Steinebach C. (2022). E3 ligase ligand chemistries: From
building blocks to protein degraders. Chem.
Soc. Rev..

[55] Wang C., Zhang Y., Wu Y., Xing D. (2021). Developments of CRBN-based
PROTACs as potential therapeutic agents. Eur.
J. Med. Chem..

[56] Steinebach C., Lindner S., Udeshi N. D., Mani D. C., Kehm H., Köpff S., Carr S. A., Gütschow M., Krönke J. (2018). Homo-PROTACs for the Chemical Knockdown of Cereblon. ACS Chem. Biol..

[57] Pettersson M., Crews C. M. (2019). PROteolysis TArgeting Chimeras (PROTACs)—Past,
present and future. Drug Discovery Today: Technol..

[58] Verma R., Mohl D., Deshaies R. J. (2020). Harnessing
the Power of Proteolysis
for Targeted Protein Inactivation. Mol. Cell.

[59] Faust T. B., Donovan K. A., Yue H., Chamberlain P. P., Fischer E. S. (2021). Small-Molecule Approaches to Targeted
Protein Degradation. Annu. Rev. Cancer Biol..

[60] Berkley K., Zalejski J., Sharma N., Sharma A. (2025). Journey of PROTAC:
From Bench to Clinical Trial and Beyond. Biochemistry.

[61] Wang C., Zhang Y., Chen W., Wu Y., Xing D. (2024). New-generation
advanced PROTACs as potential therapeutic agents in cancer therapy. Mol. Cancer.

[62] Arndt C. M., Bitai J., Brunner J., Opatz T., Martinelli P., Gollner A., Sokol K. R., Krumb M. (2023). One-Pot Synthesis of
Cereblon Proteolysis Targeting Chimeras via Photoinduced C­(sp^2^)–C­(sp^3^) Cross Coupling and Amide Formation
for Proteolysis Targeting Chimera Library Synthesis. J. Med. Chem..

[63] Steiner A., Krieger J., Jones R., Böse D., Wang Y., Eggenweiler H.-M., Williams J. D., Kappe C. O. (2022). Photoredox
Csp^3^–Csp^2^ Reductive Cross-Couplings of
Cereblon Ligands for PROTAC Linker Exploration in Batch and Flow. ChemCatChem.

[64] Beckwith, R. E. J. ; Bonazzi, S. ; Cernijenko, A. ; Fazal, A. ; Tichkule, R. B. ; Visser, M. S. 3-(1-Oxoisoindolin-2-yl)piperidine-2,6-dione Derivatives and Uses Thereof. US 2,019,062,309 A1, 2019.

[65] Lovato K., Loskot D., Gelin C., Zhu L., Chaudhry C., Vellore N. A., Del Rosario A., Courtney T., Miller S., Cho J.-H. (2025). Reductive
C­(sp^2^)–C­(sp^3^) Coupling Protocol to Enable
Linker Exploration of Cereblon E3-Ligase
BRD4 Proteolysis-Targeting Chimeras. J. Med.
Chem..

[66] Hamby T. B., LaLama M. J., Sevov C. S. (2022). Controlling Ni redox states by dynamic
ligand exchange for electroreductive Csp^3^–Csp^2^ coupling. Science.

[67] Lin Q., Gong H., Wu F. (2022). Ni-Catalyzed
Reductive Coupling of
Heteroaryl Bromides with Tertiary Alkyl Halides. Org. Lett..

[68] Aguilar
Troyano F. J., Merkens K., Anwar K., Gómez-Suárez A. (2021). Radical-Based
Synthesis and Modification of Amino Acids. Angew.
Chem., Int. Ed..

[69] Twitty J. C., Hong Y., Garcia B., Tsang S., Liao J., Schultz D. M., Hanisak J., Zultanski S. L., Dion A., Kalyani D. (2023). Diversifying Amino Acids
and Peptides via Deaminative Reductive Cross-Couplings Leveraging
High-Throughput Experimentation. J. Am. Chem.
Soc..

[70] Kutchukian P. S., Dropinski J. F., Dykstra K. D., Li B., DiRocco D. A., Streckfuss E. C., Campeau L.-C., Cernak T., Vachal P., Davies I. W. (2016). Chemistry informer libraries: A chemoinformatics
enabled approach to evaluate and advance synthetic methods. Chem. Sci..

[71] Prieto
Kullmer C. N., Kautzky J. A., Krska S. W., Nowak T., Dreher S. D., MacMillan D. W. C. (2022). Accelerating reaction generality
and mechanistic insight through additive mapping. Science.

[72] Charboneau D. J., Barth E. L., Hazari N., Uehling M. R., Zultanski S. L. (2020). A Widely
Applicable Dual Catalytic System for Cross-Electrophile Coupling Enabled
by Mechanistic Studies. ACS Catal..

[73] Fu J., Lundy W., Chowdhury R., Twitty J. C., Dinh L. P., Sampson J., Lam Y.-H., Sevov C. S., Watson M. P., Kalyani D. (2023). Nickel-Catalyzed Electroreductive
Coupling of Alkylpyridinium
Salts and Aryl Halides. ACS Catal..

[74] Dong Z., MacMillan D. W. C. (2021). Metallaphotoredox-enabled
deoxygenative arylation of
alcohols. Nature.

[75] Bogdos M. K., Müller P., Morandi B. (2023). Structural Evidence for Aromatic
Heterocycle N–O Bond Activation via Oxidative Addition. Organometallics.

[76] Note: X7 and X11 were coupled with 1° NHP esters under the stoichiometric nickel conditions. See Reference 20.

[77] Dreher S. D., Krska S. W. (2021). Chemistry Informer Libraries: Conception, Early Experience,
and Role in the Future of Cheminformatics. Acc.
Chem. Res..

[78] Tran V. T., Kim N., Rubel C. Z., Wu X., Kang T., Jankins T. C., Li Z.-Q., Joannou M. V., Ayers S., Gembicky M. (2023). Structurally Diverse Bench-Stable Nickel(0) Pre-Catalysts: A Practical
Toolkit for In Situ Ligation Protocols. Angew.
Chem., Int. Ed..

[79] Su Z.-M., Deng R., Stahl S. S. (2024). Zinc and manganese redox potentials
in organic solvents and their influence on nickel-catalysed cross-electrophile
coupling. Nat. Chem..

[80] Feng C., Cunningham D. W., Easter Q. T., Blum S. A. (2016). Role of LiCl in
Generating Soluble Organozinc Reagents. J. Am.
Chem. Soc..

[81] Knochel P., Krasovskiy A. (2006). Convenient Titration Method for Organometallic Zinc,
Magnesium, and Lanthanide Reagents. Synthesis.

[82] Lin Q., Diao T. (2019). Mechanism of Ni-Catalyzed
Reductive 1,2-Dicarbofunctionalization
of Alkenes. J. Am. Chem. Soc..

[83] Lin Q., Fu Y., Liu P., Diao T. (2021). Monovalent Nickel-Mediated Radical
Formation: A Concerted Halogen-Atom Dissociation Pathway Determined
by Electroanalytical Studies. J. Am. Chem. Soc..

[84] Day C. S., Rentería-Gómez Á., Ton S. J., Gogoi A. R., Gutierrez O., Martin R. (2023). Elucidating electron-transfer events
in polypyridine nickel complexes for reductive coupling reactions. Nat. Catal..

[85] Raab T. J., Doyle A. G. (2025). Reactivity Studies of Bipyridine-Ligated
Nickel­(I)
and Nickel(0) Complexes Inform the Mechanism in Modern Cross-Coupling
Reactions. J. Am. Chem. Soc..

