# Supplementary material for: Cross-Electrophile Coupling of N‑Hydroxyphthalimide Esters with Aryl Bromides Using an Inner-Sphere Homogeneous Reductant
Source: J Am Chem Soc. 2026 Jan 8;148(2):2590–9. doi: 10.1021/jacs.5c18451 (PMC12833811; doi:10.1021/jacs.5c18451)

Cross Electrophile Coupling of *N*-Hydroxyphthalimide Esters With Aryl Bromides Using an Inner-Sphere, Homogeneous Reductant

Kasturi Ganguli[a], Alexandro R. Cruz[a], Justin B. Diccianni[b], Pablo García-Reynaga[c]*, and Daniel J. Weix[a]*

[a]University of Wisconsin, Madison, WI USA 53706
[b]Global Discovery Chemistry, Johnson & Johnson, 1400 McKean Road, Spring House, PA USA 19477
[c]Global Discovery Chemistry, Johnson & Johnson, 3210 Merryfield Row, San Diego, CA USA 92121

Supporting Information

Cross Electrophile Coupling of *N*-Hydroxyphthalimide Esters With Aryl Bromides Using an Inner-Sphere, Homogeneous Reductant 1

1. General Information 4
1.1 Reagents 4
Metals and reductants 4
Ligands 4
Solvents 4
Aryl Substrates 4
Other Reagents 5
1.2 Methods 5
NMR Spectroscopy 5
Gas Chromatography 5
GC/MS Analysis 5
High Resolution Mass Spectrometry (HR/MS) 6
Supercritical Fluid Chromatography Mass Spectrometry (SFC/MS) 6
Liquid Chromatography Mass Spectrometry (LC/MS) 6
Chromatography 6

2. General Procedures 6
2.1 General Procedure A for Reaction Optimization Between Aryl Bromides and N-Hydroxy Phthalimide Esters 6
2.2 General Procedure B for Decarboxylative Cross-Electrophile Coupling 7
2.3 General Procedure for Benchtop Reactions 8
2.3.1 General Procedure for Benchtop Reactions Under N_2 8
2.3.2 General Procedure for Benchtop Reactions Under Air 9
2.4 General Procedure C for the Synthesis of NHP Esters 10
2.4.1 Synthesis of 1-benzyl 4-(5-methyl-1,3-dioxoisoindolin-2-yl) piperidine-1,4-dicarboxylate (NHP ester-3) 11
2.4.2 Synthesis of 1-benzyl 4-(1,3-dioxoisoindolin-2-yl) 4-methylpiperidine-1,4-dicarboxylate (NHP ester-13) 11
2.5 General Procedure for the Synthesis of the Si-DHP reductants 12
2.5.1 Synthesis of TMS-DHP reductant 12
2.5.2 Synthesis of TMS-Me_4DHP reductant 13
2.5.3 Synthesis of TES-DHP reductant 13
2.5.4 Synthesis of TMS-DHB reductant 13
2.5.5 Synthesis of TBS-DHP reductant 14
Pictures of Si-DHP Synthesis Procedure 16
2.6 Procedure for the Synthesis of Informer Bromide X10 17
2.7 Procedure for One-Pot XEC and Amide-Bond Formation (3az) 18

3. Reaction Optimization 18
3.1 Effect of Additives on Test XEC Reaction 18
3.2 Effect of Reductant on Test XEC Reaction[a] 20
3.3 Effect of Nickel(II) Precursor on Test XEC Reaction.[a] 21
3.4 Effect of Reaction Concentration on Test XEC Reaction[a] 22
3.5 Effect of Reaction Temperature on Test XEC Reaction[a] 23
3.5.1 General Procedure for Examining Temperature With Activated Aryl Bromides 23
3.5.2 General Procedure for Examining Temperature With Deactivated Aryl Bromides 24
3.6 Effect of Solvents on Test XEC Reaction[a] 24
3.7 Effect of Catalyst Loading on Test XEC Reaction[a] 26
3.8 Flowchart for Choosing Starting Conditions and Reaction Optimization 27

4. Informer Bromide Comparison Data **28**

5. Mechanistic Studies **29**

5.1 Reactivity of TMS-DHP with Aryl Bromide[a] **29**

5.2 Reactivity of TMS-DHP with NHP Ester[a] **30**

5.2.1 Reduction of NHP Ester with Si-DHP in Toluene 30

5.2.2 Reduction of NHP Ester with Si-DHP in DMA 31

5.2.3 Reduction of NHP Ester with TMS-DHP In the Presence of Different Additives 31

5.3 Reduction of (dtbbpy)$NiCl_2$ With Various Chemical Reductants **32**

5.3.1 Reduction of (dtbbpy)$NiCl_2$ with TMS-DHP 33

5.3.2 Reduction of (dtbbpy)$NiCl_2$ with Zn/LiCl 33

5.3.3 Reduction of (dtbbpy)$NiCl_2$ with TDAE 34

5.4 Reduction Studies of (dtbbpy)$NiBr_2$ in the Presence of TMS-DHP or TMS -Me_4DHP **35**

5.4.1 Quantitation of Reduced Nickel with Iodocumene Trap 35

5.4.2 Quantitation of Reduced Nickel with Alkene and Cyclopentadieneone Traps. 36

5.4.2.1 Reduction with TMS-Me_4DHP Reductant 36

5.4.2.2 Reduction with TMS-DHP Reductant 37

5.5 Stoichiometric Reaction of (dtbbpy)Ni(COD) with NHP Ester 2a **37**

5.6 Stoichiometric Reaction of (dtbbpy)Ni^{II}(Ar)(NPhth) with NHP Ester 2a **38**

5.6.1 Synthesis of (dtbbpy)Ni^{II}(Ar)(NPhth) Complex 38

5.6.2 Stochiometric Reaction Between (dtbbpy)Ni^{II}(Ar)(NPhth) Complex and NHP Ester 2a 38

5.7 UV-Vis Study of the Reduction of Ni(II) to Ni(0) **39**

5.7.1 Dip-Probe Reactor Setup 39

5.7.2 Procedure 39

5.7.2.1 Reduction with TMS-DHP 40

5.7.2.2 Reduction with TES-DHP 41

5.7.2.3 Reduction with TMS-Me_4DHP 41

5.7.2.4 Reduction with TMS-DHB 42

5.8 Effect of Solvent on the Rate of the Cross-Electrophile Coupling Reaction **43**

5.8.1. Effect of Solvent on the Rate of XEC Between an Activated Aryl Bromide (1) and a 1° NHP Ester (2a) 43

5.8.2. Effect of Solvent on the Rate of XEC Between a Deactivated Aryl Bromide (4-Bromo anisole) and 1°, 2°, and Strained Ring 3° NHP Esters 44

6. Product Characterization **46**

7. References **76**

8. NMR Spectra **80**

1. General Information

1.1 Reagents

Metals and reductants

All metal catalysts and reductants, unless otherwise noted, were stored and handled in a nitrogen-filled glovebox. Nickel(II) bromide ethylene glycol dimethyl ether [$NiBr_2$(dme)] and Nickel(II) chloride ethylene glycol dimethyl ether [$NiCl_2$(dme)] were purchased from Millipore Sigma and used as received. Bis(1,5-cyclooctadiene)nickel(0) [$Ni(COD)_2$] was purchased from Strem, stored in the glovebox freezer (at –30 ºC), and used as received. The pre-formed (dtbbpy)$NiBr_2$ complex was synthesized following the reported procedure.[1]

Zinc flake (-325 mesh) and manganese powder (-325 mesh) were purchased from Alfa Aesar and stored inside the glovebox.

Zinc flake was activated with TMSCl following a literature procedure.[2] Mn powder was used as received.

Lithium was purchased as lithium granules (10 g bottle) from Sigma Aldrich and stored in the nitrogen glovebox. Note: the lithium would develop a lithium nitride coating typically between 1–3 months, so care should be taken to use the bottle as quickly as possible if stored inside the nitrogen glove box.

Tetrakis(dimethylamino)ethylene (TDAE) was purchased from Sigma Aldrich and stored in the glovebox freezer.

Bis(pinacolato)diboron (B_2pin_2) was purchased from Frontier Scientific and stored in the glovebox.

The organosilane reductants were synthesized following the synthetic procedures (*vide infra*) and stored in the glovebox.

Ligands

4,4´-Di-tert-butyl-2,2´-bipyridine (dtbbpy) was purchased from Ambeed, stored in a glovebox, and used as received.

2,6-bis(pyrazol-1-yl)pyridine (bpp) was purchased from Ambeed, stored in a glovebox, and used as received.

Solvents

Anhydrous *N*,*N*-dimethylacetamide (DMA), *N*,*N*-dimethylformamide (DMF), dimethylsulfoxide (DMSO), acetonitrile (MeCN), tetrahydrofuran (THF), and toluene were purchased from Sigma Aldrich, stored in a glovebox, and used as received.

Aryl Substrates

Most of the aryl bromides used in this study are known compounds. The aryl bromides 4-bromoacetanilide[3], 4-bromophenyl acetate[4] and *N*-(5-bromopyridin-3-yl)acetamide[3] were synthesized from the corresponding aryl amines or phenol by acylation using acetic anhydride, following the reported procedures.

The informer aryl bromides **X1**-**X13** (except **X10**) were purchased from Sigma Aldrich.

The informer bromide **X10** was synthesized by modifying a reported procedure[5] (**section 2.5**). Methyl 5-bromo-8-hydroxy-1,6-naphthyridine-7-carboxylate (1 g bottle) required for the synthesis of **X10** and the NHP ester-**20** (1-(1,3-dioxoisoindolin-2-yl) 4-methyl cyclohexane-1,4-dicarboxylate) were purchased from Enamine. 4-fluorobenzyl amine was purchased from Ambeed.

Other Reagents

Chlorotrimethylsilane (Me_3SiCl) was purchased from Alfa Aesar in a septum-sealed bottle. *Care must be taken to purge the glovebox atmosphere after use to preserve the copper oxygen scavenger of the glovebox, which reacts irreversibly with chlorosilanes.*

Chlorotriethylsilane (Et_3SiCl) and ^{t}butylchlorodimethylsilane ($^tBuMe_2SiCl$) were purchased from Acros Organics in septum-sealed bottles. *Care must be taken to purge the glovebox atmosphere after use to preserve the copper oxygen scavenger of the glovebox, which reacts irreversibly with chlorosilanes.*

Pyrazine and tetramethyl pyrazine were purchased from TCI America and stored in the glove box to avoid contamination with moisture.

Sodium carbonate (Na_2CO_3) was purchased from Sigma Aldrich and dried under vacuum at 200 mmHg/40 °C for 3 h before being transferred and stored in a glovebox.

Anhydrous sodium acetate (NaOAc) was purchased from JT Baker and dried under vacuum at 200 mmHg/40 °C for 3 h before being stored in a glovebox.

1,3,5-trimethoxybenzene was purchased from Sigma Aldrich and used as received.

1.2 Methods

NMR Spectroscopy

^{1}H and ^{13}C NMR spectra were acquired on 400 and 500 MHz Bruker Avance III or Oxford NMR instruments. ^{19}F-NMR spectra were acquired on a 400 MHz Avance spectrometer equipped with a BBFO probe (Bruker). In the absence of residual TMS in NMR solvent, NMR chemical shifts are reported in ppm and are referenced to the residual solvent peak (δ = 7.26 ppm, ^{1}H NMR, δ = 77.16 ppm, ^{13}C NMR for $CDCl_3$; δ = 7.16 ppm, ^{1}H NMR, δ = 128.62 ppm, ^{13}C NMR for C_6D_6; δ = 2.50 ppm, ^{1}H NMR, δ = 39.50 ppm, ^{13}C NMR for DMSO-D_6). For NMR solvents containing TMS, the NMR chemical shifts are referenced to the residual TMS peak at 0.0 ppm. We elected to report AA´BB´ systems present in para-disubtituted arenes as such instead of the more common, but incorrect, "m" or "d" designations. The measured value between the two prominent signals in this system is equal to $J_{AB} + J_{AB'}$ with the J_{AB} value being much larger than the $J_{AB'}$ value.

Gas Chromatography

GC analyses were performed on an Agilent 7890A GC equipped with dual DB-5 columns (20 m × 180 µm × 0.18 µm), dual FID detectors, and hydrogen as the carrier gas. A sample volume of 1 μL was injected at a temperature of 300 °C and a 100:1 split ratio. The initial inlet pressure was 20.3 psi but varied as the column flow was held constant at 1.8 mL/min for the duration of the run. The initial oven temperature of 50 °C was held for 0.46 min followed by a temperature ramp of 65 °C/min up to 300 °C. The total run time was 7.0 min and the FID temperature was 325 °C.

GC/MS Analysis

GC/MS analyses were performed on a Shimadzu GCMS-TQ8040 NX equipped with an Rxi-5MS column (30 m × 0.25 mm × 0.25 µm) with a triple quadrupole mass analyzer using helium as the carrier gas. The analysis method used in all cases was 1 µL injection of sample, an injection temp of 250 °C, and a 75:1 split ratio. The initial inlet pressure was 16.0 psi but varied as the column flow was held constant at 1.87 mL/min for the duration of the run. The interface temperature was held at 275 °C, and the ion source (EI^+, 30 eV) was held at 200 °C. The initial

oven temperature was at 50 °C for 1 min with the detector off, followed by a temperature ramp, with the detector on, to 300 °C at 20 °C/min. Total run time was 17.50 min.

High Resolution Mass Spectrometry (HR/MS)

Mass spectrometry data was collected on a Thermo Q Exactive™Plus (thermofisher.com) via flow injection with electrospray ionization or via ASAP-MS™ (asap-ms.com) by the chemistry mass spectrometry facility at the University of Wisconsin-Madison.

Supercritical Fluid Chromatography Mass Spectrometry (SFC/MS)

SFC/MS analyses were performed on a Waters Acquity UPC2 equipped with Acquity UPC2 PDA and Acquity QDa Detector. A Daicel Dcpack SFC-B column (4.6 mm ID × 150 mm L, 5 µm PS) was used for separations. The eluent was a mixture (90:10 CO_2/MeOH) with a flow rate of 2 mL/min at 40 °C with a ABPR at 1500 psi. A Chiralpak IC-3 column (3 mm ID × 150 mm L, 3 µm PS) was used for chiral separations. The eluent was a gradient from 90:10 CO_2/MeOH up to 60:40 CO_2/MeOH over 7 minutes, with a flow rate of 0.8 mL/min at 40 °C with the ABPR at 2200 psi.

Liquid Chromatography Mass Spectrometry (LC/MS)

UPLC-MS analyses were performed on a Waters Acquity UHPLC using a BEHC18 column (1.7 µm, 2.1 × 50 mm) with an Acquity PDA detector and Acquity QDA MS detector. MPA: 0.05% TFA in H2O; MPB: 100% ACN, starts from 1% B to 40% B for 0.5 minutes, then hold for 1.25 minutes, then increase to 65% B for 0.5 min, then hold for 1.1 minutes, then increase to 95% B over 0.15 minutes.

Chromatography

Chromatography was performed on Biotage Isolera One (detection at 254 nm and 280 nm, on Sfar Duo columns) or on a Teledyne Isco Rf-200 (detection at 254 nm and 280 nm, on Gold C18 Reversed Phase Columns, using water and acetonitrile eluent). Products were visualized by UV, $KMnO_4$ stain, or fractions were analyzed by GC, SFC/MS or LC/MS.

2. General Procedures

2.1 General Procedure A for Reaction Optimization Between Aryl Bromides and N-Hydroxy Phthalimide Esters

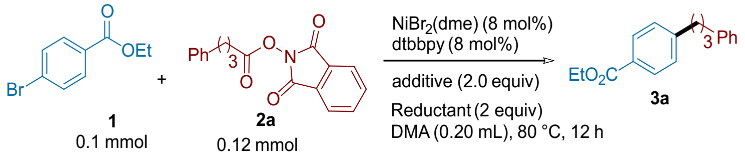

Reactions were set up in an N_2 filled glove box. A catalyst solution was prepared by charging an oven-dried 1-dram vial with a PTFE-coated stir bar, with $NiBr_2$(dme) (4.9 mg, 0.016 mmol), and dtbbpy (4.3 mg, 0.016 mmol). The solids were dissolved in DMA (400 μL) and allowed to stir at rt for 30 min. In a separate 1-dram vial charged with a PTFE-coated stir bar, the NHP ester **2a** (37.1 mg, 0.12 mmol, 1.2 equiv), the optional additive (0.20 mmol, 2.0 equiv), ethyl-4-

bromobenzoate (15.5 µL, 0.10 mmol, 1.0 equiv), and 1,3,5-trimethoxybenzene (16.8 mg, 0.10 mmol, 1.0 equiv as an internal standard) were added sequentially. Then 200 µL of the $NiBr_2$(dme)/dtbbpy prestirred solution (0.008 mmol, 8 mol%) was added. Finally, the corresponding reductant (0.20 mmol, 2.0 equiv) was added. The reaction vial was sealed with a screw cap fitted with PTFE-faced silicone septa and removed from the glovebox. The reaction was allowed to stir (1250 RPM) at the listed temperature for the specified time (between 4–12 h).

GC Analysis

The reaction was monitored by GC analysis by taking a 15 µL aliquot of the crude reaction mixture with an autopipette. The sample was filtered through a 2 cm silica plug in a Pasteur pipette. The silica plug was further washed with EtOAc (~1.8–2.0 mL) and collected in a GC vial. The sample was analyzed by GC-FID. Yields were determined based on the peak area of the analyte compared to 1,3,5-trimethoxybenzene as an internal standard.

2.2 General Procedure B for Decarboxylative Cross-Electrophile Coupling

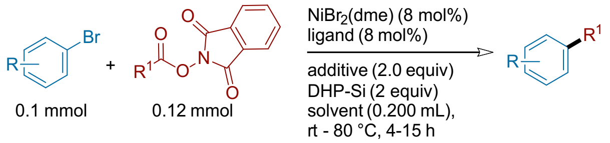

Reactions were set up in an N_2 filled glove box. The reactions were run between 0.1 mmol and 0.3 mmol scale for isolation. For a 0.1 mmol scale reaction, a catalyst solution was prepared by sequentially charging an oven dried scintillation vial with a PTFE-coated stir bar, $NiBr_2$(dme) (4.9 mg, 0.016 mmol) and the listed ligand (0.016 mmol for single ligands, dtbbpy or 2,6-bis(pyrazol-1-yl)pyridine; or, 0.008 mmol each of dtbbpy and 2,6-bis(pyrazol-1-yl)pyridine). The solids were dissolved in the specified solvent (400 µL, DMA, toluene, dioxane) and the contents were stirred for 30 min, resulting in a forest green (for DMA) to pale green (for toluene) solution. A separate oven-dried 1-dram vial with a PTFE-coated stir bar was charged with NHP ester (0.12 mmol, 1.2 equiv), aryl bromide (0.10 mmol, 1.0 equiv), and Na_2CO_3 or DIPEA (0.2 mmol, 2.0 equiv). Then, 200 µL of the Ni catalyst stock solution (in either DMA or toluene) was added. Lastly, the Si-DHP reductant (0.20 mmol, 2.0 equiv) was added, and the vial was sealed with a screw cap fitted with a PTFE-faced silicone septum before being removed from the glovebox. The contents of the reaction vessel were stirred (1250 RPM) at the set temperature (20–22 °C for room temperature or 40 °C and 80 °C for higher temperatures) for a time varying between 4 h and 15 h.

Isolation by Normal Phase Chromatography

The crude reaction mixture was diluted with EtOAc (~5 mL), and the contents filtered through a 2 cm silica plug in a thick-walled glass pipette. The filtrate was concentrated by rotary evaporation. The resulting material was dry loaded onto silica and purified by silica gel flash chromatography as outlined in **section 1.2**.

Isolation by Reverse Phase Chromatography

The reaction mixture was filtered through silica gel (a 4 cm silica plug in a thick-walled glass pipette) and washed with methanol (~5 mL). Thereafter, the filtrate was concentrated by

rotary evaporation. The resulting material was dissolved in DMSO (~1 mL) which was filtered through a cotton plug to remove any undissolved solid and the cotton plug was then washed with an additional DMSO (0.5 mL). The resulting clear filtrate (~1–1.5 mL) was loaded onto a C18 column, and the material was purified by reverse phase chromatography using H_2O and CH_3CN as the mobile phase as outlined in **section 1.2**. Typically, the product-containing fractions were confirmed by LC-MS analysis and the desired fractions (containing the product in CH_3CN/H_2O) were subsequently transferred to a 500 mL round bottom flask and concentrated in the rotary evaporator under reduced pressure to remove the acetonitrile. The concentrated fraction was dissolved in chloroform (~15 mL) and washed with brine solution (20 mL). The brine wash was in turn washed with chloroform (3 × 15 mL). The combined organic extract was dried over Na_2SO_4, filtered through a cotton plug in a powder funnel into a 250 mL round bottom flask, and concentrated in a rotary evaporator. The concentrated solution of chloroform (~2 mL) was transferred into a scintillation vial which was further dried to give the final product.

Note: for compounds containing acidic functional groups (for example, **3g**, **3r**) the aqueous extraction was done with HCl solution (1 M) instead of brine. For the PROTAC derivatives (**3h**, **3i**) having base sensitive functional groups or substrates that have weakly acidic functional groups (**X10** and compounds **3s**, **4m**), the aqueous extraction was performed with a saturated solution of ammonium chloride.

NMR Yields

For substrate **3aa** which was volatile and for **4g** which was difficult to separate from the by-products (phthalimide and Me_4-pyrazine), NMR yields were taken to quantify the yield of the product. In this case, 1,3,5-trimethoxybenzene (16.8 mg, 0.10 mmol, 1.0 equiv) was added as an internal standard after adding all reagents inside the glovebox. After the reaction was complete, the crude mixture was filtered through silica gel (4 cm), washed with EtOAc and the filtrate was concentrated by rotary evaporation. The organic layer (EtOAc) was subjected to an aqueous work up with cold saturated aqueous NaCl (to get rid of most of the DMA and salt impurities) and the aqueous layer was extracted with additional EtOAc (3×15 mL). The combined organic layers were dried over Na_2SO_4, filtered, and the solvent was removed under reduced pressure. The residue was diluted with $CDCl_3$ (~500 μL) and analyzed via NMR spectroscopy.

2.3 General Procedure for Benchtop Reactions

2.3.1 General Procedure for Benchtop Reactions Under N_2

$NiBr_2$(dme) and dtbbpy (8 mol% each) were weighed on the benchtop into a 1-dram vial (pre-stir vial), followed by the addition of 0.2 mL of dry solvent (DMA or toluene). The resulting solution was allowed to stir on the benchtop for 30 min at rt. In the meantime, the corresponding aryl bromide (0.10 mmol, 1.0 equiv), NHP ester (0.12 mmol, 1.2 equiv), Na_2CO_3 (0.2 mmol, 2.0 equiv), and 1,3,5-trimethoxybenze (16.8 mg, 0.10 mmol, 1.0 equiv as an internal standard) were weighed into a 1-dram vial (reaction vial) on the benchtop. The $NiBr_2$(dme)/dtbbpy solution was then added to the reactants in the second vial and the vial was fitted with a PTFE-faced silicone septa in black phenolic screw cap. A nitrogen needle inlet and a bleed needle outlet (open to air) was used to exchange the headspace in the reaction vial with N_2 for 5 min. With exclusion of oxygen and water, a 0.67 M solution of the TMS-DHP reductant was made by dissolving the reductant in the appropriate volume of dry, degassed solvent (DMA or toluene). The TMS-DHP solution (0.3 mL, 0.2 mmol, 2 equiv) was then transferred by syringe to the reaction vial using

standard syringe techniques. The vent needle was removed, and the vial top was sealed with electrical tape. The reaction was allowed to stir (1250 RPM) at 80 °C for 2 h (or 10 h for 4-bromo anisole). After that, the reaction was worked up by GC analysis as reported in SI **section 2.1**. The results of the benchtop reaction are summarized below.

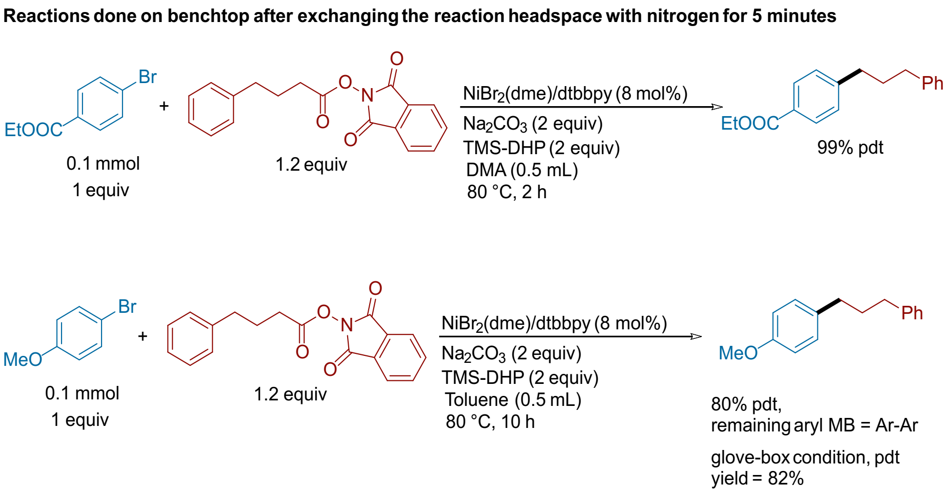

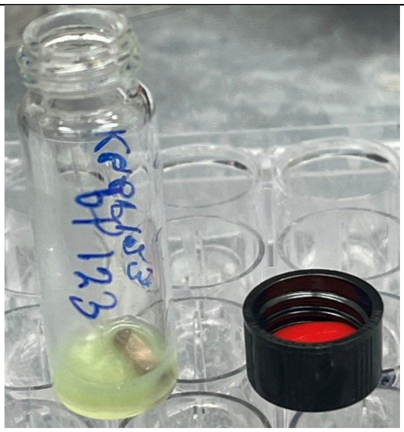

Image 1. Reaction vial after adding the Ni/L pre-stir to the reagents under air.

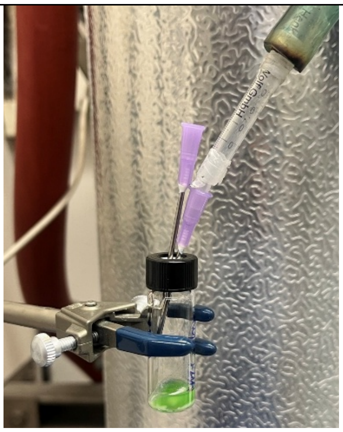

Image 2. Purging the reaction vial headspace with nitrogen inside the fume hood.

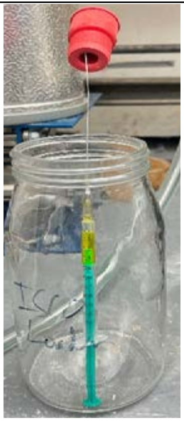

Image 3. The solution of TMS-DHP reductant in syringe for transfer.

2.3.2 General Procedure for Benchtop Reactions Under Air

For the reactions under air, the procedure was same as above, except that the reaction headspace was not purged with nitrogen before addition of the TMS-DHP reductant solution. In this case, we observed a slower rate of reaction (24 h instead of 2 h), however the outcome was similar (85% product with 2 equiv TMS-DHP and 94% product with 4 equiv TMS-DHP).

Reactions done under air, without exchanging the headspace with nitrogen

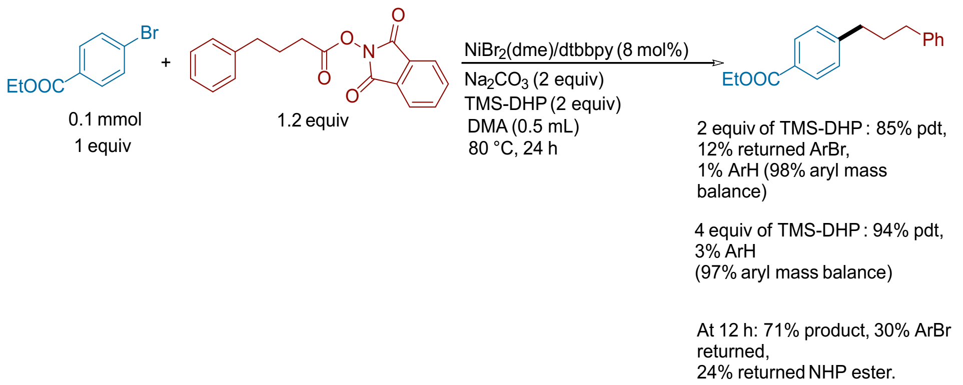

These results suggest that the experiments can be run on the benchtop, without a glovebox, using standard air-free techniques and a solution of the reductant.

2.4 General Procedure C for the Synthesis of NHP Esters

NHP esters **1–20** were prepared by a slight modification of the reported procedure (using DCC instead of DIC).[6] To a 250 mL round bottomed flask, with a magnetic stir bar was added the carboxylic acid (5.0 mmol, 1.0 equiv), *N*-hydroxyphthalimide (897.2 mg, 5.5 mmol, 1.1 equiv), and dichloromethane (~50 mL). To this solution was added *N,N*-dimethylaminopyridine (DMAP) (61.1 mg, 0.5 mmol, 0.1 equiv) and the solution was allowed to stir for 5 min. Thereafter, DCC (*N*,*N′*-dicyclohexylcarbodiimide) (1.13 g, 5.5 mmol, 1.1 equiv) was added to the solution and the flask was capped with a rubber septum affixed with a vent needle. The reaction mixture was allowed to stir at rt (20–25 °C) for ~12–15 h. Thereafter, the reaction mixture was filtered through a short pad of silica into a round bottom flask and the silica was rinsed with an additional ~50 mL of dichloromethane. The solvent was removed under reduced pressure on a rotary evaporator, and the crude reaction mixture was purified by silica gel column chromatography using ethyl acetate/ hexanes as eluent. The spectra of NHP esters **1**,[7] **2**,[7] **4**,[8] **5**,[6] **6**,[6] **7**,[6] **8**,[6] **9**,[9] **10**,[10] **11**,[6] **12**,[11] **14**,[12] **15**,[8] **16**,[13] **17**,[9] and **18**[14] matched with previous reports.

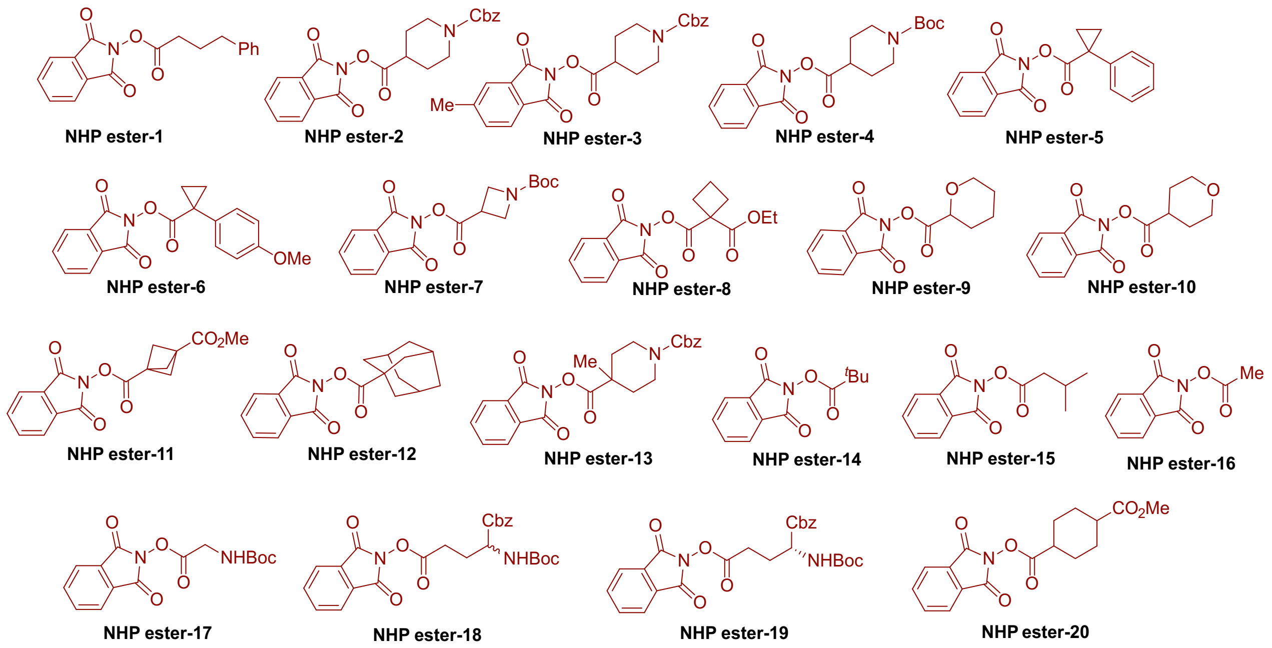

2.4.1 Synthesis of 1-benzyl 4-(5-methyl-1,3-dioxoisoindolin-2-yl) piperidine-1,4-dicarboxylate (NHP ester-3)

The title compound was prepared according to general procedure C using 1-(benzyloxycarbonyl)piperidine-4-carboxylic acid (1.32 g, 5.00 mmol, 1.0 equiv), *N*-hydroxyphthalimide (897.2 mg, 5.5 mmol, 1.1 equiv), *N,N*-dimethylaminopyridine (DMAP) (61.1 mg, 0.5 mmol, 0.1 equiv), DCC (*N,N′*-dicyclohexylcarbodiimide) (1.13 g, 5.5 mmol, 1.1 equiv) and dichloromethane (~50 mL). Purification of the crude material by column chromatography (gradient from 100% hexanes to 35–40% EtOAc/Hex) using 25 g silica afforded the title product (1.70 g, 4.0 mmol, 80%) as a yellow-white solid. **^{1}H NMR (500 MHz, $CDCl_3$)** δ 7.76 (d, *J* = 7.7 Hz, 1H), 7.68 (d, *J* = 1.4 Hz, 1H), 7.58–7.56 (m, 1H), 7.39 – 7.30 (m, 5H), 5.14 (s, 2H), 4.13–4.08 (m, 2H), 3.12 (t, *J* = 12.2 Hz, 2H), 2.93 (tt, *J* = 10.1, 4.0 Hz, 1H), 2.53 (s, 3H), 2.88–2.04 (m, 2H), 1.94–1.85 (m, 2H). **^{13}C{^{1}H} NMR (126 MHz, $CDCl_3$)** δ 170.7, 162.3, 162.2, 155.3, 146.5, 136.8, 135.4, 129.3, 128.7, 128.2, 128.1, 126.3, 124.7, 124.1, 67.4, 42.9, 38.5, 27.8 (br s), 22.3. **HRMS (ESI-MS)** m/z: $[M+H]^+$ calcd for $C_{23}H_{23}N_2O_6^+$, 423.1551, found 423.1545.

2.4.2 Synthesis of 1-benzyl 4-(1,3-dioxoisoindolin-2-yl) 4-methylpiperidine-1,4-dicarboxylate (NHP ester-13)

The title compound was prepared according to general procedure C using 1-(benzyloxycarbonyl)-4-methylpiperdine-4-carboxylic acid (1.39 g, 5.0 mmol, 1.0 equiv), *N*-hydroxyphthalimide (897.2 mg, 5.5 mmol, 1.1 equiv), *N,N*-dimethylaminopyridine (DMAP) (61.1 mg, 0.5 mmol, 0.1 equiv), DCC (*N,N′*-dicyclohexylcarbodiimide) (1.13 g, 5.5 mmol, 1.1 equiv) and dichloromethane (~12.5 mL). Purification of the crude material by column chromatography (0–100% EtOAc/Hex) using 25 g silica afforded the title product (950.0 mg, 2.25 mmol, 45%) as a pale white solid. **^{1}H NMR (500 MHz, $CDCl_3$)** δ 7.91–7.87 (m, 2H), 7.81–7.78 (m, 2H), 7.39–7.30 (m, 5H), 5.14 (s, 2H), 4.03 (s, 2H), 3.22 (s, 2H), 2.28 (d, *J* = 13.6 Hz, 2H), 1.57–1.54 (m, 2H), 1.49 (s, 3H). **^{13}C{^{1}H} NMR (126 MHz, $CDCl_3$)** δ 172.7, 162.1, 155.3, 136.9, 134.9, 129.1, 128.6, 128.1, 128.0, 124.1, 67.3, 41.9, 41.4, 35.0, 26.4. **HRMS (ESI-MS)** m/z: $[M+H]^+$ calcd for

$C_{23}H_{23}N_2O_6^+$, 423.1551, found 423.1549.

2.5 General Procedure for the Synthesis of the Si-DHP reductants

2.5.1 Synthesis of TMS-DHP reductant

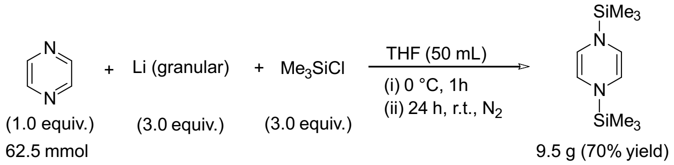

In an N_2 filled glove box, a 250 mL oven dried round bottom flask was charged with a teflon-coated stir bar, THF (30 mL), and chlorotrimethylsilane (TMSCl, 3 equiv, 187.5 mmol, 20.4 g, 24.8 mL). To this solution was added granular Li (3 equiv, 187.5 mmol, 1.30 g). The flask was sealed with a rubber septum, taken outside the glovebox, and fitted with a nitrogen balloon connected by a 1 mL plastic syringe. The flask was cooled to –78 °C in an ethanol-dry ice bath for about 10 min. Thereafter, with stirring (630–690 RPM), a solution of pyrazine (5.00 g, 62.5 mmol) in THF (20 mL) was added dropwise (via syringe) to the Li/TMSCl solution over the course of 1 hour at –78 °C. The resulting mixture was warmed to rt and stirred for an additional 24 h. After this time, additional electrical tape was wrapped around the flask and the septa and the flask was cycled back into the glovebox (the additional tape is to ensure the septa does come undone during the evacuation and refilling of the antechamber). To remove the unreacted Li metal and LiCl, the reaction was filtered directly through a 600 mL fritted glass filter funnel (medium porosity) into a 500 mL oven dried round bottom flask. The reaction flask was washed with an additional THF (10 mL) and filtered. The filtrate was concentrated under vacuum at rt (between 25–30 °C) inside the glove box, and the solid yellow residue was redissolved in minimum amount of diethyl ether (~15 mL). To remove excess LiCl, a second filtration was performed through a separate fritted glass (medium porosity) filter, and rinsed with additional diethyl ether (2×10 mL). The combined diethyl ether filtrate was concentrated under vacuum to ~10 mL total volume and transferred to two 20 mL scintillation vials, which were placed in a –30 °C freezer, where the reductant crystallized as yellow needles (after approximately 1 h). Thereafter, the ether was decanted out of the vials, and the crystals were dried with vacuum overnight to give the pure reductant (9.9 g, 70% yield). **^{1}H NMR (500 MHz, C_6D_6)** δ 4.67 (s, 4 H), –0.06 (s, 18 H). **^{13}C{^{1}H} NMR (126 MHz, C_6D_6)** δ 115.5, –1.7. The spectra matched those reported in the literature.[15]

Safety Note: Since an excess of Li (between 700 mg and 2.5 g) was used in these reactions, quenching of unreacted Li was performed inside a cleared fume hood following the guidelines reported in the literature.[16] The remaining Li would be slowly transferred from the fritted glass filter funnel to a bath of ethanol (~50–75 mL). Constant stirring with a metallic spatula was maintained to dissipate the heat generated from Li dissolution. Quenching was judged complete (approximately ~30–60 min depending on the amount of Li), upon formation of a white precipitate (indicative of lithium oxide/hydroxide in addition to lithium chloride) in the ethanol bath. Then, cold water was added dropwise with spatula stirring to ensure full quenching. The resulting clear solution would be disposed of according to local regulations. (For images related to filtration of Li inside the glovebox and quenching of lithium inside the fume hood, see **Image 8** among the pictures attached below).

2.5.2 Synthesis of TMS-Me4DHP reductant

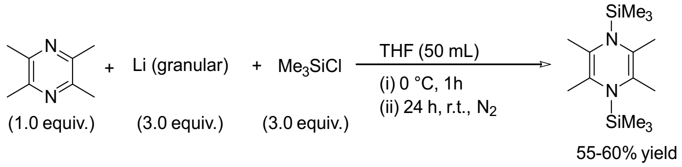

For the TMS-Me4DHP reductant (starting from tetramethylpyrazine 5.0 g, 36.7 mmol, 1 equiv instead of pyrazine, 760 mg Li and 14.0 mL of TMSCl), the synthesis followed the same procedure as **2.5.1**. For work up: the unreacted Li and precipitated LiCl were filtered inside the glove box through 600 mL fritted glass filter funnel (medium porosity) into a 500 mL oven dried round bottom flask. The flask was washed with additional THF (10 mL) and filtered into the round bottom flask. The THF solution was concentrated under vacuum at rt (between 25–30 °C), and the remaining white solid residue (containing LiCl) was dissolved in a minimum amount of diethyl ether (~15 mL) and filtered under vacuum (medium porosity). The residue was rinsed with another portion of diethyl ether (2×10 mL) and this was also passed through the filter. The resulting ether filtrate was concentrated to ~10 mL total volume under vacuum, transferred to a 20 mL scintillation vial, and dried under vacuum at rt (25–30 °C) overnight to give a near colorless solid (5.7–6.2 g, 55–60 % yield). The purity of the reductant was estimated by taking a quantitative ^{1}H NMR vs 1,3,5-trimethoxybenzene as the internal standard (96% pure). **^{1}H NMR (500 MHz, C_6D_6)** δ 1.69 (s, 12 H), 0.23 (s, 18 H). **^{13}C{^{1}H} NMR (126 MHz, C_6D_6)** δ 127.0, 19.1, 1.8. The spectra matched those reported in the literature.[15]

2.5.3 Synthesis of TES-DHP reductant

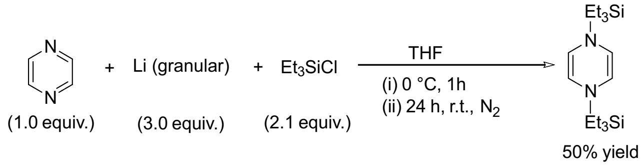

For the triethylsilyl reductant, the reaction procedure was the same as the TMS-DHP reductant except that a lesser amount of chlorotriethylsilane (19.8 g, 22.0 mL, 131.2 mmol, *2.1 equiv instead of 3.0 equiv*) was used. For work-up, the unreacted Li and precipitated LiCl were filtered inside the glove box through a 600 mL fritted glass filter funnel (medium porosity) into a 500 mL round bottom flask. The flask was washed with a additional THF (10 mL) and filtered into the round bottom flask. The THF solution was evaporated to dryness under vacuum to afford a brownish yellow, viscous solution containing undissolved LiCl as a white precipitate. This solution was dissolved in a minimal (~20 mL) amount of diethyl ether and then filtered through another 600 mL fritted glass filter funnel (medium porosity) into a 250 mL round bottom flask. The white precipitate was washed with diethyl ether (2×20 mL) to dissolve any leftover reductant (this step got rid of the LiCl precipitate while keeping the reductant in the ether filtrate). The filtrate was then concentrated under vacuum (~200 mmHg/rt), to ~20 mL and then transferred to a scintillation vial. The solution in the vial was dried under vacuum (~200 mmHg/rt, 10 h) to provide a viscous brownish-yellow liquid that was the pure TES-DHP reductant (9.7 g, 50% yield). **^{1}H NMR** (500 MHz, C_6D_6) δ 4.71 (s, 4 H), 0.99 (t, J = 5 Hz, 18 H), 0.50 (q, J = 5 Hz, 12 H). **^{13}C{^{1}H} NMR** (126 MHz, C_6D_6) δ 116.3, 7.1, 3.7.

2.5.4 Synthesis of TMS-DHB reductant

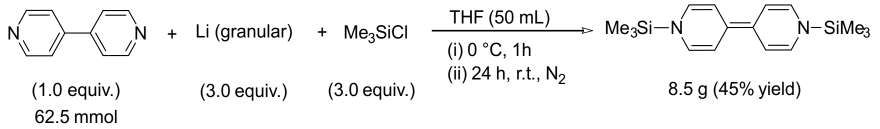

For the TMS-DHB reductant (starting from 4,4′-bipyridine instead of pyrazine), the synthesis followed the same procedure as above (for the TMS-DHP reductant). In an inert-atmosphere glovebox, a 250 mL round bottom flask was charged with a teflon-coated stir bar, THF (30 mL), and chlorotrimethylsilane (TMSCl, 3 equiv, 188 mmol, 20.4 g, 24.8 mL). To this solution was added granular Li (3 equiv, 188 mmol, 1.30 g). The flask was stoppered with a rubber septum, taken outside the glovebox, and fitted with a nitrogen balloon connected by a 1 mL plastic syringe. The flask was cooled in an ethanol-dry ice bath for about 10 min. Thereafter, with stirring, a solution of 4,4′-bipyridine (9.8 g, 62.5 mmol) in THF (20 mL) was added dropwise (via syringe) to the Li/TMSCl solution such that the external temperature (dry ice bath) was maintained below 0 °C (about 1 h addition time). The resulting mixture was allowed to warm to rt and was stirred for an additional 24 h at rt. [*Note: During addition of the 4,4′-bipyridine solution in THF the TMSCl/Li solution in THF turned into a deep purple color which sustained for up to ~2 h after the complete addition of the 4,4′-bipyridine. After ~12 h of stirring (690 RPM) at rt the reaction mixture had turned a brick red color*]. After this time, the flask was carefully sealed with electrical tape and moved back into the glovebox. To remove the unreacted Li metal and LiCl, the reaction was filtered directly through a 600 mL fritted glass filter funnel (medium porosity) into a 500 mL oven dried round bottom flask. The flask was washed with additional THF (10 mL) and filtered into the round bottom flask. The filtrate was then concentrated under high vacuum, and the bright red residue was dissolved in minimum amount (10–15 mL) of diethyl ether. To remove excess LiCl, a second filtration was performed through a separate fritted glass (medium porosity) filter and rinsed with additional diethyl ether (2×10 mL). The combined diethyl ether filtrate was concentrated with vacuum to ~10 mL total volume and transferred to two 20 mL scintillation vials, which were placed in a –30 °C freezer. The reductant crystallized as bright red needles upon standing at –30 °C for ~3 h. Thereafter, the ether solutions were decanted out of the scintillation vials and the crystals were dried overnight to give the pure reductant (8.5 g, 45% yield). **^{1}H NMR (500 MHz, C_6D_6)** δ 5.85–5.83 (m, 4 H), 5.76–5.74 (m, 4 H), –0.08 (s, 18 H). **^{13}C{^{1}H} NMR (126 MHz, C_6D_6)** δ 127.1, 110.1, 109.5, –1.4. The spectra matched those reported in the literature.[17] *Note: the TMS-DHB reductant was unstable at rt and was observed to decompose over a period of 1 week if stored at rt on a glove box shelf. Therefore, it was stored in the glove box freezer, at –30 ºC and away from light, where it was stable for a period of more than 3 months.*

2.5.5 Synthesis of TBS-DHP reductant

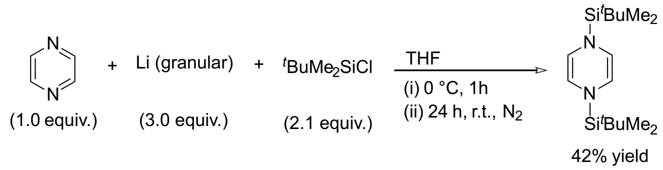

For the TBS-DHP (TBS = tert-butyldimethylsilyl) reductant, the reaction procedure was the same as the TES-DHP reductant except that tert-butyldimethylsilyl chloride (19.8 g, 131.2 mmol, *2.1 equiv instead of 3.0 equiv*) was used. For work-up, the unreacted Li and precipitated LiCl were filtered inside the glove box through a 600 mL fritted glass filter funnel (medium porosity) into a 500 mL round bottom flask, and the THF solution was evaporated to dryness to

result in a brownish yellow viscous solution containing undissolved LiCl as a white precipitate. To remove excess LiCl, this solution was dissolved in a minimal (~20 mL) amount of diethyl ether and filtered through another 600 mL fritted glass filter funnel (medium porosity) into a 250 mL round bottom flask. The residue on the filter funnel was washed with diethyl ether (2×20 mL) and filtered. The combined ether filtrate was concentrated under vacuum to ~20 mL volume and then transferred to two 20 mL scintillation vials. The solution in the scintillation vials was dried under vacuum (~ 10 h) to afford a viscous brown liquid that corresponded to the pure TBS-DHP reductant (8.2 g, 42 % yield). **^{1}H NMR (500 MHz, C_6D_6)** δ 4.72 (s, 4H), 1.03 (s, 18H), –0.08 (s, 12H). **^{13}C{^{1}H} NMR (126 MHz, C_6D_6)** δ 116.5, 26.6, 20.5, –6.8.

Pictures of Si-DHP Synthesis Procedure.

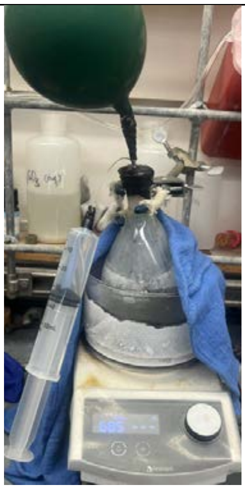

Image 1. Dropwise addition of pyrazine solution to TMSCl/Li in THF in the ethanol/dry ice bath.

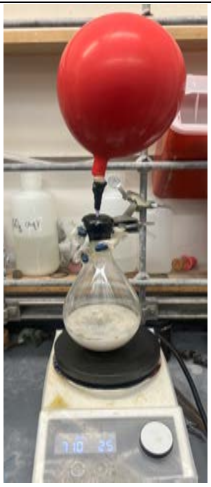

Image 2. Stirring of reaction mixture at rt for 24 h under nitrogen.

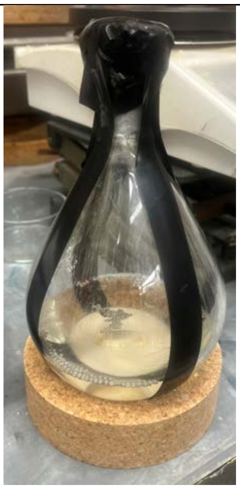

Image 3. Sealing the round bottom flask with electrical tape before taking inside the glovebox.

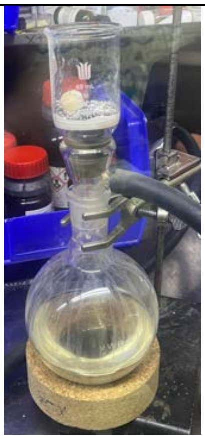

Image 4. Filtration set-up for separating the undissolved LiCl and Li from the reductant by filtration inside the glovebox.

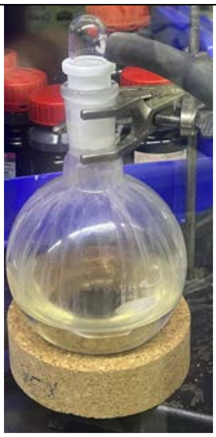

Image 5. Removing THF from the filtrate by connecting to the vacuum line inside the glove box.

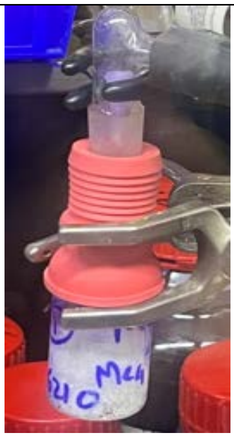

Image 6. The TMS-Me_4DHP reductant collected in a scintillation vial after drying inside the glove box.

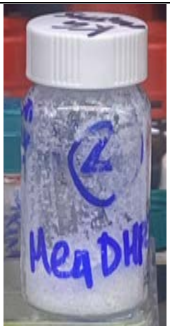

Image 7. The TMS-Me_4DHP reductant stored as a crystalline white solid inside the glovebox.

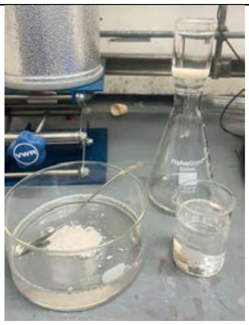

Image 8. Quenching unreacted lithium by slow addition into an ethanol bath kept inside a fume hood.

2.6 Procedure for the Synthesis of Informer Bromide X10

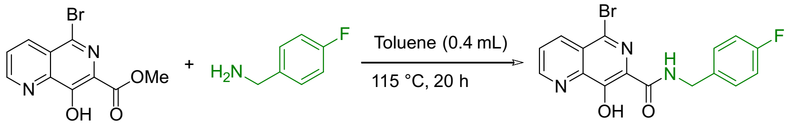

In the nitrogen filled glovebox, an oven dried 1-dram vial was charged with a PTFE-coated stir bar, methyl 5-bromo-8-hydroxy-1,6-naphthyridine-7-carboxylate (200 mg, 0.71 mmol, 1 equiv), 4-fluorobenzylamine (242.2 µL, 2.1 mmol, 3 equiv) and toluene (0.4 mL). The reaction vial was sealed with a screw cap fitted with PTFE-faced silicone septa and removed from the glovebox. The reaction was allowed to stir (1250 RPM) at 115 °C for 20 h. The vial was allowed to cool to rt, and the toluene was evaporated on a rotary evaporator. The solid residue was then dissolved in chloroform and washed with sat. aqueous NH_4Cl (20 mL). The aqueous layer was back-extracted with chloroform (3×15 mL). The combined chloroform extracts (~60 mL) were dried over Na_2SO_4, filtered into a 250 mL round bottom flask, and filtrate was evaporated to dryness using a rotary evaporator. The residue was dissolved in 1 mL DMSO and loaded (using liquid loading technique) onto a Gold C18 Reversed Phase Column (43 g) and purified by reverse-phase chromatography (water/acetonitrile mixture, gradient of 0.5% CH_3CN/H_2O–65% CH_3CN in H_2O, both containing 0.1% TFA). The pure product was isolated as a pale brown solid (180.6 mg, 68% yield). **^{1}H NMR (500 MHz, $CDCl_3$)** δ 13.30 (s, 1H), 9.20 (dd, J = 4.3, 1.6 Hz, 1H), 8.54 (dd, J = 8.5, 1.7 Hz, 1H), 8.16 (s, 1H), 7.73 (dd, J = 8.5, 4.2 Hz, 1H), 7.40–7.36 (m, 2H), 7.07 (m, 2H), 4.67 (d, J = 6.2 Hz, 2H). **^{13}C{^{1}H} NMR (126 MHz, $CDCl_3$)** δ 168.4, 163.5, 161.6, 155.2, 154.6, 144.5, 137.1, 133.3 (d, $J_{C\text{-}F}$ = 3.3 Hz), 131.0, 129.8 (d, $J_{C\text{-}F}$ = 8.2 Hz), 127.7, 125.7, 125.6, 115.9 (d, $J_{C\text{-}F}$ = 21.7 Hz), 42.7. **^{19}F{^{1}H} NMR (377 MHz, $CDCl_3$)** δ –114.4. The ^{1}H NMR data matched with that reported in the literature.[5]

HRMS-ESI (m/z): $[M+H]^+$ calcd for $C_{16}H_{12}BrFN_3O_2^+$, 376.0091, found 376.0086.

Note: Using 3 equiv of 4-fluorobenzylamine and a reaction time of ~20–24 h were found to be optimal for the formation of X10. A higher amount (4 equiv of 4-fluorobenzyl amine) and longer reaction times (> 24 h) led to the S_NAr product (by substitution of 4-fluorobenzyl amine at the C-Br bond) as a major side reaction. Using a lower amount (2 equiv of 4-fluorobenzyl amine) led to incomplete consumption of the aryl bromide starting material.

2.7 Procedure for One-Pot XEC and Amide-Bond Formation (3az)

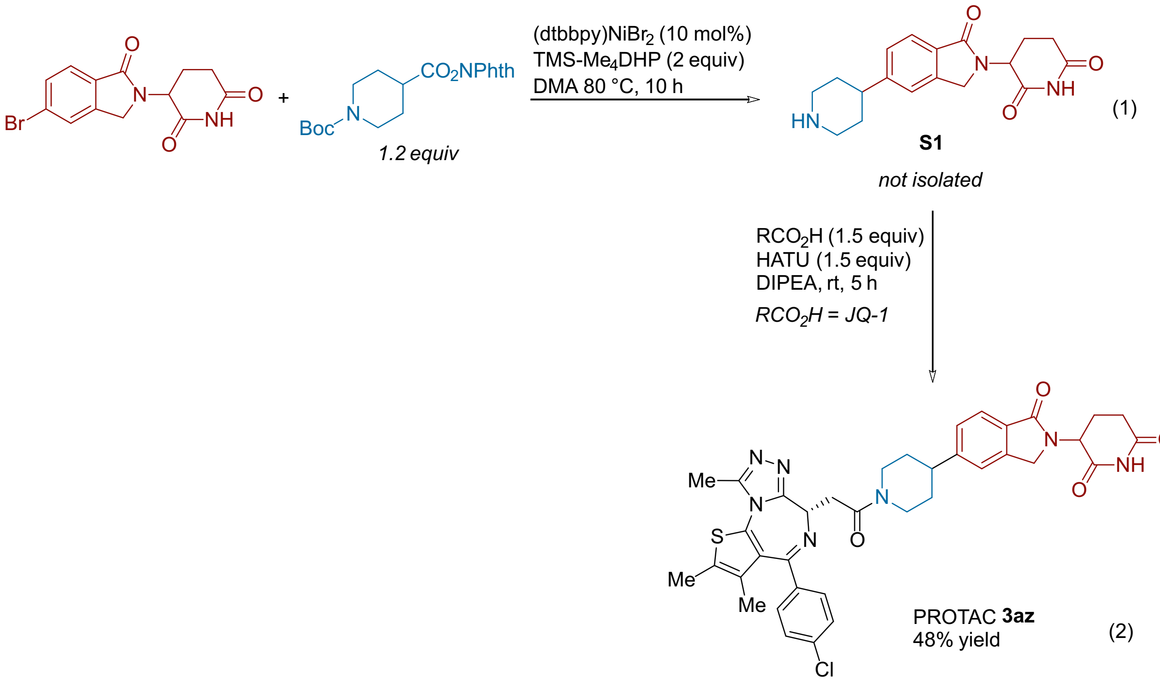

In the nitrogen filled glovebox, an oven dried 1-dram vial was charged with a PTFE-coated stir bar, 3-(5-bromo-1-oxoisoindolin-2-yl)piperidine-2,6-dione (16.2 mg, 0.05 mmol, 1 equiv), 1-(tert-butyl) 4-(1,3-dioxoisoindolin-2-yl) piperidine-1,4-dicarboxylate (28.1 mg, 0.075 mmol, 1.5 equiv), (dtbbpy)$NiBr_2$ (2.43 mg, 0.005 mol, 10 mol%) and DMA (0.5 mL, 0.1 M). Subsequently, TMS-Me_4DHP (28.3 mg, 0.10 mmol, 2 equiv) was added and the reaction vial was sealed with a screw cap fitted with PTFE-faced silicone septa and removed from the glovebox. The reaction was allowed to stir (1250 RPM) at 80 °C for 10 h. Afterwards, the vial was cooled to room temperature, and the reaction mixture was analyzed by LC-MS to confirm the formation of the amine (**S1**). By omitting base/activator, the Boc group could be deprotected during the XEC step (eq 1), presumably by the TMS-Br generated during nickel catalyst turnover. After that, diisopropylethylamine (20.7 mg, 27.9 µL, 0.16 mmol, 3.2 equiv), (6S)-4-(4-chlorophenyl)-2,3,9-trimethyl-6H-thieno[3,2-f][1,2,4]triazolo[4,3-a][1,4]diazepine-6-acetic acid (36.1 mg, 0.09 mmol, 1.8 equiv) and HATU (34.2 mg, 0.09 mmol, 1.8 equiv) were added sequentially to the crude reaction mixture. The vial was then capped and allowed to stir at room temperature (25 °C) for 3 h. After 3 h, the reaction mixture was analyzed by LC-MS to confirm complete consumption of the amine (**S1**) and formation of the product **3az**. The pure product **3az** was isolated as a white solid (19.7 mg, 48% yield over three steps) by reverse phase column chromatography (water/acetonitrile gradient).

3. Reaction Optimization

3.1 Effect of Additives on Test XEC Reaction

General Procedure A (section 2.1) was followed for the screening of additives. A catalyst stock solution was prepared by charging an oven-dried 1-dram vial with a PTFE-coated stir bar, $NiBr_2$(dme) (37.0 mg, 0.12 mmol, and dtbbpy (32.2 mg, 0.12 mmol). The solids were dissolved in DMA (3000 μL) and allowed to stir at rt for 30 min. In a separate 1-dram vial, the NHP ester **2a**

(37.1 mg, 0.12 mmol, 1.2 equiv), the additive (0.20 mmol, 2.0 equiv), ethyl-4-bromobenzoate (15.5 µL, 0.10 mmol, 1.0 equiv), 1,3,5-trimethoxybenzene (16.8 mg, 0.10 mmol, 1.0 equiv as an internal standard) were added sequentially. Then 200 µL of the $NiBr_2$(dme)/dtbbpy prestirred solution (0.008 mmol, 8 mol%) was added. Finally, TMS-DHP (45.3 mg, 0.20 mmol, 2.0 equiv) was added. The reaction vial was sealed with a screw cap fitted with PTFE-faced silicone septa and removed from the glovebox. The reaction was allowed to stir (1250 RPM) at room temperature (30 ºC) for 12 h. After 12 h, the reaction was analyzed by GC analysis as reported in **section 2.1**.

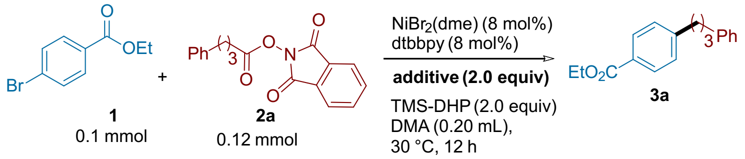

Entry	Additive	3a[a] (%)	1 returned (%)
1	none	53	46
2	Na_2CO_3	100	0
3	K_2CO_3	71	21 (2% Aryl-H)
4	Cs_2CO_3	26	52 (21% Aryl-H)
5	Li_2CO_3	93	10 (3% Aryl-H)
5	NaOAc	71	30
6	NaOPiv	0	100
7	Na-ethyl-*n*-hexanoate	0	100
8	CsF	89	14
9	CsOAc	9	83 (8% Aryl-H)
10	K-phthalimide	16	83
11	TMSBr	34	61
12	Bis(trimethylsilyl)acetamide	58	36
13	DIPEA	90	8

[a]Yields were determined by GC analysis calibrated against 1,3,5-trimethoxybenzene as an internal standard.

Note: With soluble bases such as Na-ethyl-n-hexanoate and CsOAc, a significant amount of the mixed anhydride was observed upon heating the NHP ester with just the base in DMA.

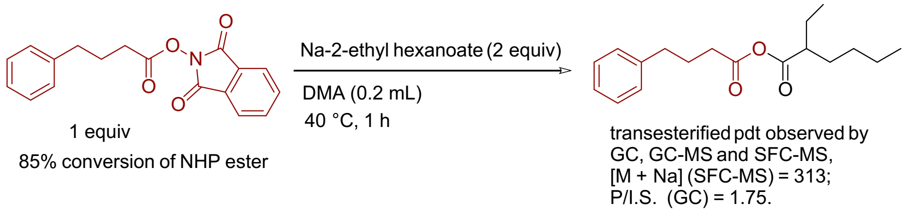

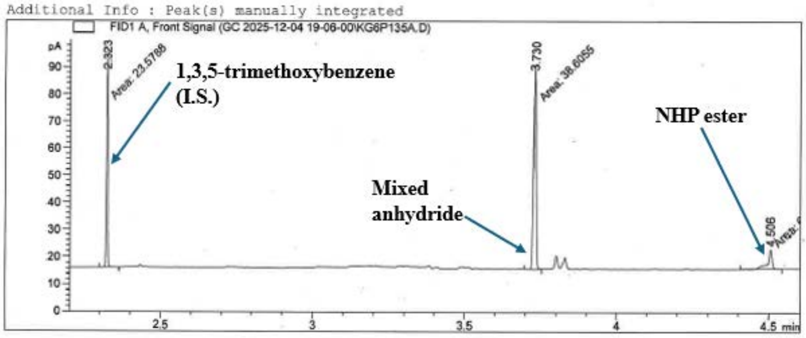

Peak #	RetTime [min]	Sig	Type	Area [pA*s]	Height [pA]	Area %
1	2.323	1	MM	23.57881	82.87549	34.20703
2	3.730	1	MM	38.60550	73.86254	56.00705
3	4.506	1	MM	6.74541	6.73374	9.78592

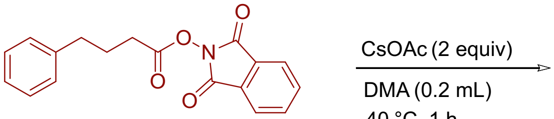

1 equiv

40% conversion of NHP ester

transesterified pdt observed by GC, GC-MS and SFC-MS,
[M]+ (GC-MS) = 206;
P/I.S. (GC) = 0.28.

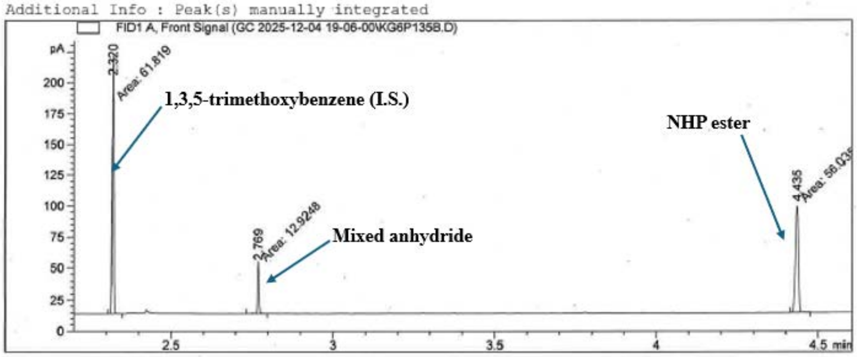

Peak #	RetTime [min]	Sig	Type	Area [pA*s]	Height [pA]	Area %
1	2.320	1	MM	61.81902	189.08833	47.26976
2	2.769	1	MM	12.92484	39.18350	9.88295
3	4.435	1	MM	56.03533	85.58899	42.84728

3.2 Effect of Reductant on Test XEC Reaction[a]

General Procedure A (section 2.1) was followed for the screening of reductants. A catalyst solution was prepared for each individual reaction, by charging an oven-dried 1-dram vial with a PTFE-coated stir bar, $NiBr_2$(dme) (4.9 mg, 0.016 mmol, 16 mol%), and dtbbpy (4.3 mg, 0.016 mmol, 16 mol%). The solids were dissolved in DMA (400 μL) and allowed to stir at rt for 30 min. In a separate 1-dram vial, the NHP ester **2a** (37.1 mg, 0.12 mmol, 1.2 equiv), ethyl-4-bromobenzoate (15.5 µL, 0.10 mmol, 1.0 equiv), 1,3,5-trimethoxybenzene (16.8 mg, 0.10 mmol, 1.0 equiv as an internal standard) were added sequentially [For the screens with Si-DHP/ Si-Me_4DHP/ Si-DHB reductants, Na_2CO_3 (21.2 mg, 0.20 mmol, 2.0 equiv) was added]. Then 200 µL of the $NiBr_2$(dme)/dtbbpy solution (0.008 mmol, 8 mol%) was transferred to reaction vial. Finally,

the corresponding reductant (0.20 mmol, 2.0 equiv) was added to the reaction vial. The reaction vial was sealed with a screw cap fitted with PTFE-faced silicone septa and removed from the glovebox. The reaction was allowed to stir (1250 RPM) at 80 ºC for 12 h. After 12 h, the reaction was worked up by GC analysis as reported in **section 2.1**.

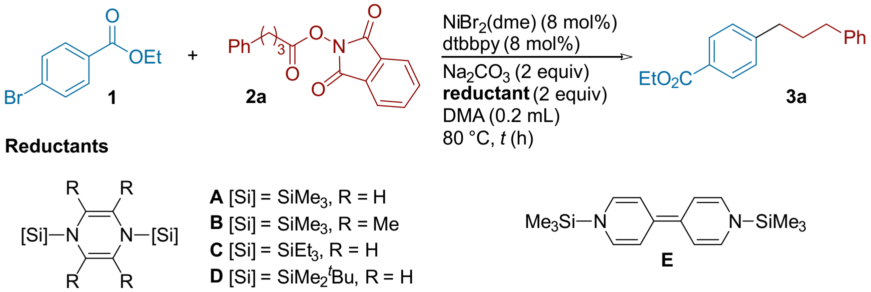

Entry	Reductant	Time	3a[b] (%)	Returned 1
1	**A**	12	100	0
2	**B**	12	100	0
3	**C**	12	82	18
4	**D**	12	10	77
5	**E**	12	24	9 (25% aryl dimer, 15% aryl-H)
6[b]	TDAE	12	8	90
7[c]	**A**	4	100	0
8[b, c]	B_2Pin_2	4	0	100
9[b]	Zn	12	82	18
10[b, d]	Mn	12	48	25
11	none	12	0	100

[a]Yields were determined by GC analysis calibrated against 1,3,5-trimethoxybenzene as an internal standard. [b]Na_2CO_3 not added. [c]Reaction run at 40 ºC. [d]10% aryl dimer was observed.

3.3 Effect of Nickel(II) Precursor on Test XEC Reaction.[a]

General Procedure A (section 2.1) was followed for screening of Ni(II) precursors. A catalyst solution was prepared by charging an oven-dried 1-dram vial with a PTFE-coated stir bar, the corresponding Ni(II) pre-catalyst (0.016 mmol, 16 mol%), and dtbbpy (4.3 mg, 0.016 mmol, 16 mol%). DMA (400 μL) was added, and the mixture was allowed to stir at 40 ºC for 60 min to ensure complete dissolution of the solids [*the NiI_2/dtbbpy pre-stir was initially insoluble in DMA but gradually dissolved upon heating at 40 ºC for 45 minutes, when it turned into a clear yellow solution*]. In a separate 1-dram vial, the NHP ester **2a** (37.1 mg, 0.12 mmol, 1.2 equiv), ethyl-4-bromobenzoate (15.5 µL, 0.10 mmol, 1.0 equiv), Na_2CO_3 (21.2 mg, 0.20 mmol, 2.0 equiv) and 1,3,5-trimethoxybenzene (16.8 mg, 0.10 mmol, 1.0 equiv as an internal standard) were added sequentially. Then 200 µL of the Ni(II)/dtbbpy solution (0.008 mmol, 8 mol%) was transferred to the reaction vial. Finally, TMS-DHP (45.3 mg, 0.20 mmol, 2.0 equiv) was added to the reaction vial. The reaction vial was sealed with a screw cap fitted with PTFE-faced silicone septa and removed from the glovebox. The reaction was allowed to stir (1250 RPM) at 40 ºC for 12 h. After 12 h, the reaction was worked up by GC analysis as reported in **section 2.1**.

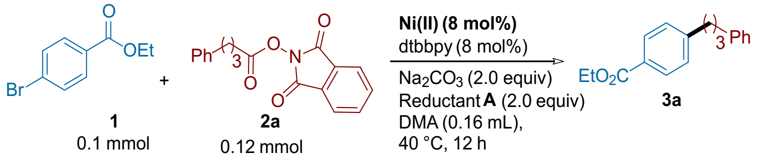

Entry	Ni(II) Precursor	3a (%)	Returned 1
1	$NiBr_2(dme)$	100	0
2	$NiCl_2(dme)$	100	0
3	NiI_2	98	0
4	$NiBr_2.3H_2O$	98	1 (1% Aryl-H)
5	$Ni(acac)_2$	3	94 (2% Aryl-H)
6	$Ni(OAc)_2.4H_2O$	81	18 (2% Aryl-H)
7[b]	$(dtbbpy)NiBr_2$	100	0

[a]Yields were determined by GC analysis calibrated against 1,3,5-trimethoxybenzene as an internal standard. [b]Using 8 mol% of the pre-formed $(dtbbpy)NiBr_2$ complex.

3.4 Effect of Reaction Concentration on Test XEC Reaction[a]

A catalyst solution was prepared by charging an oven-dried 1-dram vial with a PTFE-coated stir bar, $NiBr_2(dme)$ (18.5 mg, 0.06 mmol, 60 mol%), and dtbbpy (16.1 mg, 0.06 mmol, 60 mol%). DMA (1500 μL) was added, and the mixture was allowed to stir at 40 ºC for 30 min. In a separate 1-dram vial, the NHP ester **2a** (37.1 mg, 0.12 mmol, 1.2 equiv), ethyl-4-bromobenzoate (15.5 µL, 0.10 mmol, 1.0 equiv), Na_2CO_3 (21.2 mg, 0.20 mmol, 2.0 equiv) and 1,3,5-trimethoxybenzene (16.8 mg, 0.10 mmol, 1.0 equiv as an internal standard) were added sequentially. Then 200 µL of the $NiBr_2(dme)$/dtbbpy solution (0.008 mmol, 8 mol%) was transferred to the reaction vial, followed by DMA (x µL), depending on the concentration (x = 0 for 0.5 M, x = 200 µL for 0.25 M, x = 400 µL for 0.17 M, x = 600 µL for 0.125 M, x = 800 µL for 0.1 M and x = 1800 µL for 0.05 M). Finally, TMS-DHP (45.3 mg, 0.20 mmol, 2.0 equiv) was added to the reaction vial. The reaction vial was sealed with a screw cap fitted with PTFE-faced silicone septa and removed from the glovebox. The reaction was allowed to stir (1250 RPM) at 40 ºC for 12 h. After 12 h, the reaction was worked up by GC analysis as reported in **section 2.1**.

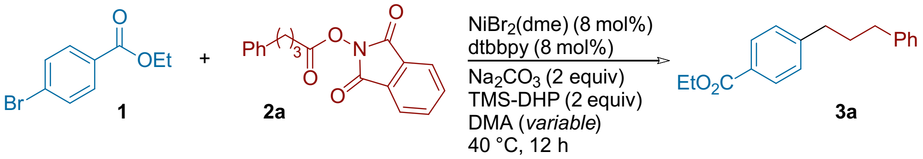

Entry	DMA amount (mL)	Conc. (M)	3a[b] (%)	Returned 1
1	0.200	0.50 M	100	0
2	0.400	0.25 M	100	0
3	0.600	0.17 M	100	0
4	0.800	0.125 M	100	0
5	1.000	0.100 M	100	0
6	2.000	0.050 M	97	3

[a]Reactions run on a 0.1 mmol scale in (x + 200) µL of DMA. [b]Yields were determined by GC analysis calibrated against 1,3,5-trimethoxybenzene as an internal standard.

3.5 Effect of Reaction Temperature on Test XEC Reaction[a]

3.5.1 General Procedure for Examining Temperature With Activated Aryl Bromides

General Procedure A (section 2.1) was followed for screening reaction temperature. A catalyst solution was prepared by charging an oven-dried 1-dram vial with a PTFE-coated stir bar, $NiBr_2$(dme) (12.3 mg, 0.04 mmol, 40 mol%), and dtbbpy (10.7 mg, 0.04 mmol, 40 mol%). DMA (1000 μL) was added and the mixture was allowed to stir at rt (20 ºC). In a separate 1-dram vial, the NHP ester **2a** (37.1 mg, 0.12 mmol, 1.2 equiv), ethyl-4-bromobenzoate (15.5 µL, 0.10 mmol, 1.0 equiv), Na_2CO_3 (21.2 mg, 0.20 mmol, 2.0 equiv) and 1,3,5-trimethoxybenzene (16.8 mg, 0.10 mmol, 1.0 equiv as an internal standard) were added sequentially. Then 200 µL of the $NiBr_2$(dme)/dtbbpy solution (0.008 mmol, 8 mol%) was transferred to the reaction vial. Finally, TMS-DHP (45.3 mg, 0.20 mmol, 2.0 equiv) was added to the reaction vial. The reaction vial was sealed with a screw cap fitted with PTFE-faced silicone septa and removed from the glovebox. The reaction was allowed to stir (1250 RPM) at the set temperature for 12 h. After 12 h, the reaction was worked up by GC analysis as reported in **section 2.1**.

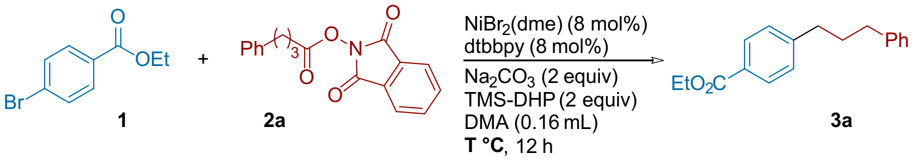

Entry	Temperature (°C)	3a (%)	Returned 1
1	20	94[b]	0 (3% ArH)
2	40	90	6
3	60	91	7
4	80	93	6

[a]Yields were determined by GC analysis calibrated against 1,3,5-trimethoxybenzene as an internal standard. [b]6 h.

3.5.2 General Procedure for Examining Temperature With Deactivated Aryl Bromides

For studying the effect of temperature on the XEC between a deactivated aryl bromide with the NHP ester, **General Procedure A (section 2.1)** was modified as follows. A catalyst solution was prepared by charging an oven-dried 1-dram vial with a PTFE-coated stir bar, $NiBr_2$(dme) (12.3 mg, 0.04 mmol, 40 mol%), and dtbbpy (10.7 mg, 0.04 mmol, 40 mol%). Toluene (1000 μL) was added, and the mixture was allowed to stir at rt (20 ºC). In a separate 1-dram vial, the NHP ester **2a** (37.1 mg, 0.12 mmol, 1.2 equiv), 4-bromo anisole (12.6 µL, 0.10 mmol, 1.0 equiv), Na_2CO_3 (21.2 mg, 0.20 mmol, 2.0 equiv) and 1,3,5-trimethoxybenzene (16.8 mg, 0.10 mmol, 1.0 equiv as an internal standard) were added sequentially. Then 200 µL of the $NiBr_2$(dme)/dtbbpy solution (0.008 mmol, 8 mol%) was transferred to the reaction vial. Finally, TMS-DHP (45.3 mg, 0.20 mmol, 2.0 equiv) was added to the reaction vial. The reaction vial was sealed with a screw cap fitted with PTFE-faced silicone septa and removed from the glovebox. The reaction was allowed to stir (1250 RPM) at the set temperature for the specified time. After the specified time (2 h, 6 h or 12 h), the reaction was worked up by GC analysis as reported in **section 2.1**. The selectivity for product formation increased at higher temperature (45% product at 40 °C, 65% product at 60 °C and 82% product at 80 °C).

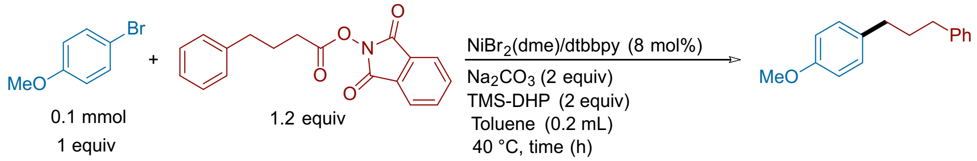

At 40 °C, 6 h:
34% pdt, 63%, ArBr returned, 3% ArH (100% MB);
15% NHP returned, 26% alkyl dimer, 20% TMS-ester

At 40 °C, 12 h:
45% pdt, 57%, ArBr returned (102% MB);
0% NHP returned, 28% alkyl dimer, 18% TMS-ester

At 60 °C, 6 h: 65% product.

At 80 °C, 2 h: 82% product.

3.6 Effect of Solvents on Test XEC Reaction[a]

General Procedure A (section 2.1) was followed for screening different solvents. A catalyst solution was prepared by charging an oven-dried 1-dram vial with a PTFE-coated stir bar, $NiBr_2$(dme) (4.9 mg, 0.016 mmol, 16 mol%), and dtbbpy (4.3 mg, 0.016 mmol, 16 mol%). The corresponding solvent (400 μL) was added, and the mixture was allowed to stir at 40 ºC for 30 min. In a separate 1-dram vial, the NHP ester **2a** (37.1 mg, 0.12 mmol, 1.2 equiv), ethyl-4-bromobenzoate (15.5 µL, 0.10 mmol, 1.0 equiv), Na_2CO_3 (21.2 mg, 0.20 mmol, 2.0 equiv) and 1,3,5-trimethoxybenzene (16.8 mg, 0.10 mmol, 1.0 equiv as an internal standard) were added sequentially. Then 200 µL of the $NiBr_2$(dme)/dtbbpy solution (0.008 mmol, 8 mol%) was transferred to the reaction vial. Finally, TMS-DHP (45.3 mg, 0.20 mmol, 2.0 equiv) was added to the reaction vial. The reaction vial was sealed with a screw cap fitted with PTFE-faced silicone septa and removed from the glovebox. The reaction was allowed to stir (1250 RPM) at 80 ºC for 12 h. After 12 h, the reaction was worked up by GC analysis as reported in **section 2.1**.

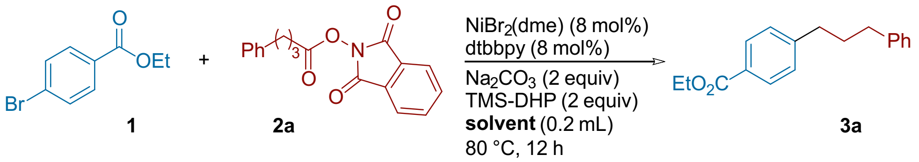

Entry	Solvent	3a[b] (%)	Returned 1[b]
1	1,4-dioxane[c]	79	0 (11% aryl dimer)
2	toluene	78	0 (9% aryl dimer)
3	1,2-dimethoxyethane	57	0 (22% aryl dimer)
4	THF[d]	81	0 (11% aryl dimer)
5	CH_3CN[c]	72	0 (11% aryl dimer, 3% aryl-H)
6	EtOAc	69	0 (16% aryl dimer)
7	^{i}PrOAc	78	0 (11% aryl dimer)
8	DMF	89	(1% aryl dimer)
9	DMSO	87	(2% aryl dimer)
10	DMA	100	0
11	NMP	100	0

[a]Reactions run on a 0.1 mmol scale in 200 µL of solvent. [b]Yields were determined by GC analysis calibrated against 1,3,5-trimethoxybenzene as an internal standard. [c]70 °C. [d]60 °C.

3.7 Effect of Catalyst Loading on Test XEC Reaction[a]

General Procedure A (section 2.1) was followed for screening the catalyst loading. A catalyst solution was prepared by charging an oven-dried 1-dram vial with a PTFE-coated stir bar, $NiBr_2$(dme) (6.2 mg, 0.020 mmol, 20 mol%), and dtbbpy (5.4 mg, 0.020 mmol, 20 mol%). The corresponding solvent (500 μL) was added and the mixture was allowed to stir at rt for 30 min. In a separate 1-dram vial, the NHP ester **2a** (37.1 mg, 0.12 mmol, 1.2 equiv), ethyl-4-bromobenzoate (15.5 µL, 0.10 mmol, 1.0 equiv), Na_2CO_3 (21.2 mg, 0.20 mmol, 2.0 equiv) and 1,3,5-trimethoxybenzene (16.8 mg, 0.10 mmol, 1.0 equiv as an internal standard) were added sequentially. Then y µL (y = 200 µL for 8 mol%, 125 µL for 5 mol%, 62.5 µL for 2.5 mol%, 25 µL for 1 mol%) of the $NiBr_2$(dme)/dtbbpy solution (0.00x mmol, x mol%) and (200-y) µL DMA were transferred to the reaction vial. Finally, TMS-DHP (45.3 mg, 0.20 mmol, 2.0 equiv) was added to the reaction vial. The reaction vial was sealed with a screw cap fitted with PTFE-faced silicone septa and removed from the glovebox. The reaction was allowed to stir (1250 RPM) at 40 °C for 12 h. After 12 h, the reaction was worked up by GC analysis as reported in **section 2.1**.

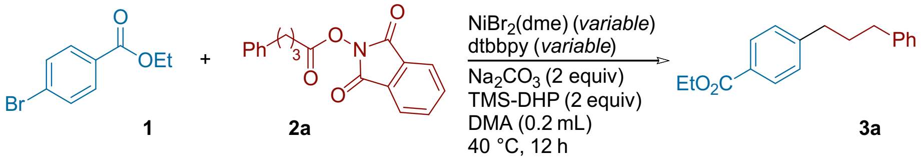

Entry	Conditions	3a[b] (%)	Returned 1
1	8 mol% catalyst	100	0
2	5 mol% catalyst	100	0
3	2.5 mol% catalyst	89	11
4	1 mol% catalyst	75	25
5	No $NiBr_2$(dme)	0	100
6	No dtbbpy	65	0 (12% aryl dimer, 6% aryl-H)

[a]Reactions run on a 0.1 mmol scale in 200 µL of solvent. [b]Yields were determined by GC analysis calibrated against 1,3,5-trimethoxybenzene as an internal standard.

3.8 Flowchart for Choosing Starting Conditions and Reaction Optimization

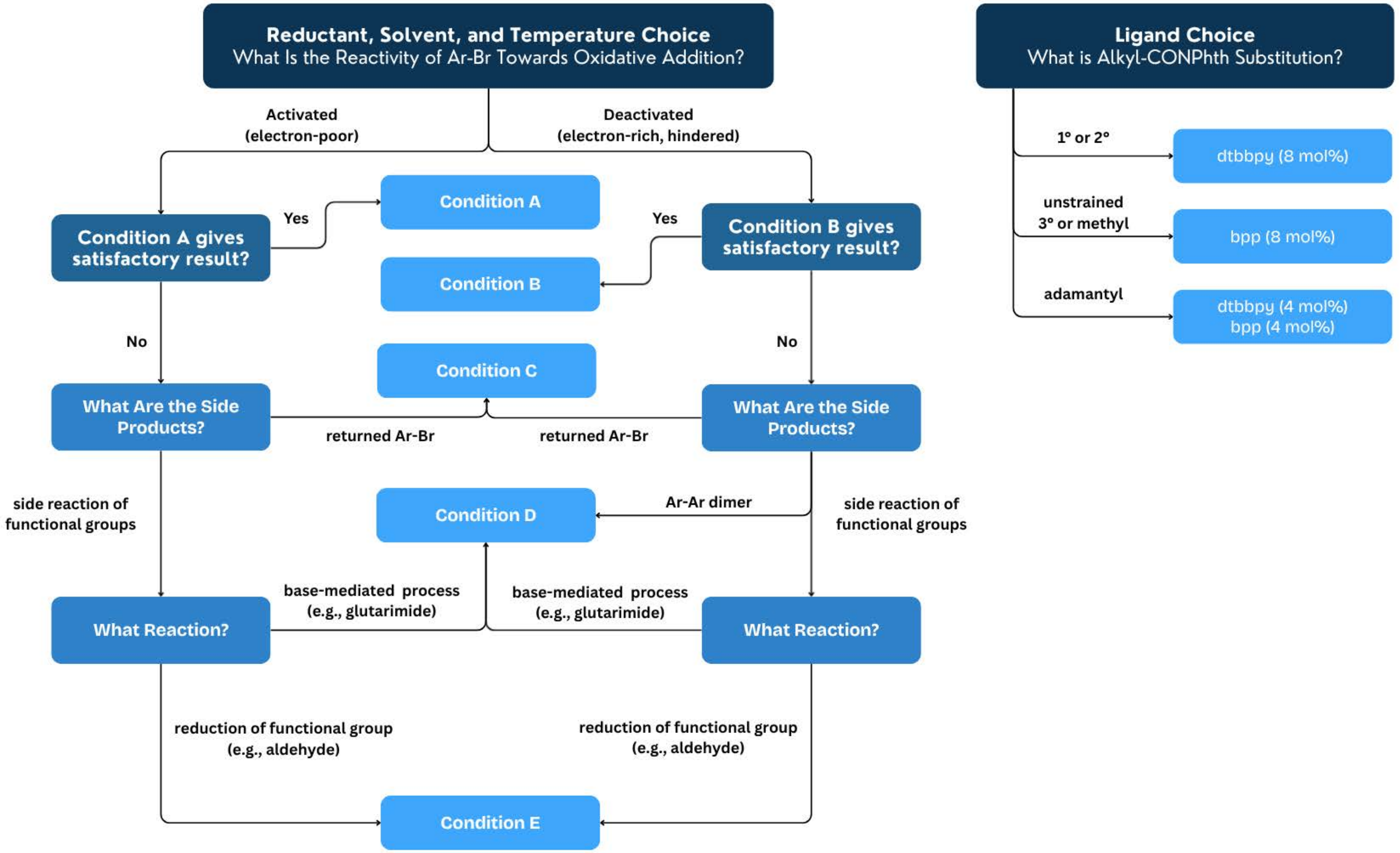

Catalyst choice. use ligands according to alkyl radical type (see flowchart above). Start with 8 mol% Ni/L. Try higher loading if yields do not improve according to flowchart (sometimes results in major improvement).

Condition A. TMS-DHP (2 equiv), Na_2CO_3 (2 equiv), DMA, 30 °C, 4 h

Condition B. TMS-Me_4DHP (2 equiv), Na_2CO_3 (2 equiv), toluene, 80 °C, 12 h

Condition C. TMS-DHP (2 equiv), Na_2CO_3 (2 equiv), toluene, 80 °C, 12 h

Condition D. TMS-Me_4DHP (2 equiv), DIPEA (2 equiv), toluene, 80 °C, 12 h

Condition E. TES-DHP (2 equiv), Na_2CO_3 (2 equiv), DMA, 40 °C, 4 h

Further optimization. Seek to balance rate of oxidative addition with rate of radical generation. There is very good solvent flexibility, but it alters rate of radical generation. Less polar solvent leads → slower radical generation. MeNHP for NHP → slower radical generation.

4. Informer Bromide Comparison Data

A - Entry 2

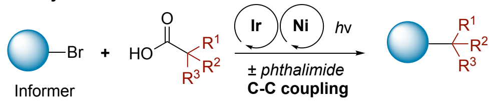

B - Entries 3 + 4

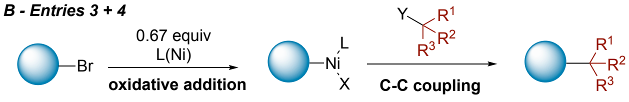

C - Entries 5 – 8

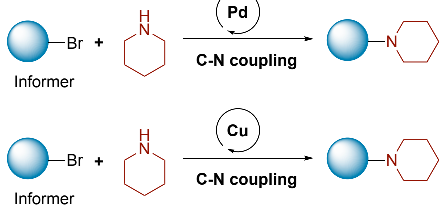

D - Entry 9

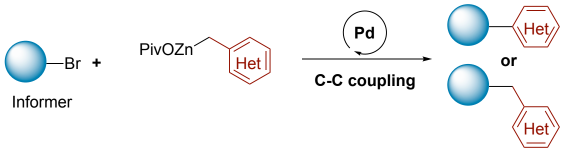

E - Entry 10

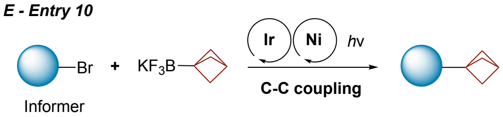

F - Entry 11

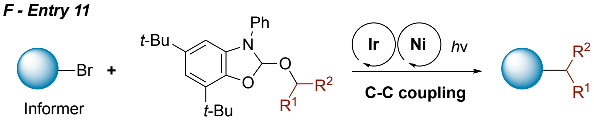

X1

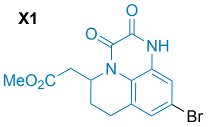

X2

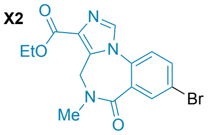

X3

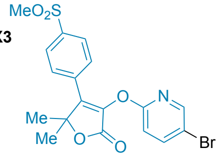

X4

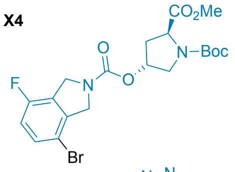

X5

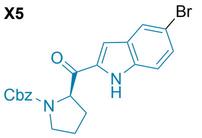

X6

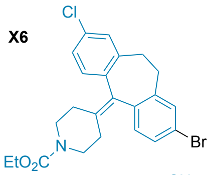

X7

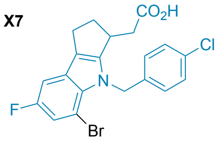

X8

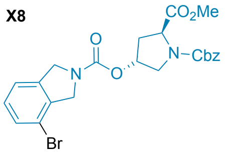

X9

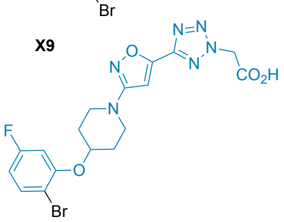

X10

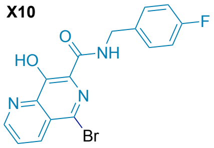

X11

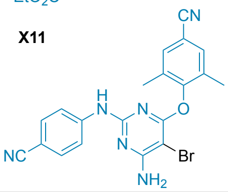

X12

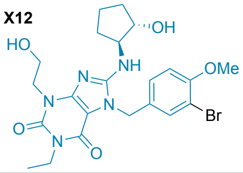

X13

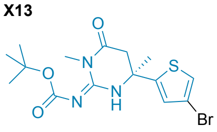

Entry	**Comparison with Published Informer Data**<br>Reaction Tested	X1	X2	X3	X4	X5	X6	X7	X8	X9	X10	X11	X12	X13	Avg. Yield (%)	Yields >20%	Yields >10%	DOI
1	**this study:** 10 mol% [Ni], **Si-DHP reductant**<br>Ar-Br + AlkylCO_2NPhth	**95**	72	39	**78**	81	72	0	66	0	34	0	68	42	49.8	10	10	this work
2	**A: metallaphotoredox:** 5 mol% [Ni], **1 mol% [Ir], hv**<br>Ar-Br + AlkylCO_2H	60	89	44	50	52	58	0	36	0	0	0	22	51	35.5	9	9	10.1126/science.abn1885
3	**B: two-step process:** 67 mol% Ni, **electrochem**<br>Ar-Br + AlkylCO_2NPhth	27	58	20	58	53	20	0	25	0	55	0	28	49	30.2	10	10	10.1038/s41557-024-01528-7
4	**B: two-step process:** 67 mol% Ni, **electrochem**<br>Ar-Br + AlkylCO_2NPhth	27	**98**	26	58	53	20	**27**	58	0	44	**20**	41	49	40.1	12	12	10.1038/s41557-024-01528-7
5	**C: amination**: 10 mol% [Pd] (1 condition)<br>Ar-Br + piperidine	16	46	0	11	27	37	0	0	0	3	0	**99**	4	18.7	4	6	10.1039/C5SC04751J
6	**C: amination**: 10 mol% [Pd] (8 conditions)<br>Ar-Br + piperidine	16	88	8	29	27	63	0	10	0	**90**	0	**99**	4	33.4	6	8	10.1039/C5SC04751J
7	**C: amination:** 25 mol% [Cu] (1 condition)<br>Ar-Br + piperidine	47	60	48	31	15	**89**	0	67	0	0	0	**93**	0	34.6	7	8	10.1039/C5SC04751J
8	**C: amination:** 25 mol% [Cu] (10 conditions)<br>Ar-Br + piperidine	47	60	**78**	53	15	**89**	0	67	0	3	0	**95**	0	39.0	7	8	10.1039/C5SC04751J
9	**D: Negishi coupling:** 3 mol% [Pd]<br>Ar-Br + BenzylZnOPiv	0	46	40	59	13	60	0	0	**42**	0	0	13	0	21.0	5	7	10.1002/anie.201604652
10	**E: metallaphotoredox:** 15 mol% [Ni], **10 mol% [Ir], hv**<br>Ar-Br + BCP-BF3K	58	89	20	y	y	61	0	y	0	0	y	y	0	28.5	4	4	10.1021/acs.orglett.0c00242
11	**F: metallaphotoredox:** 5 mol% [Ni], **1.5 mol% [Ir], hv**<br>Ar-Br + aminoacetal of 2° alkyl	57	90	**77**	**83**	**91**	81	**25**	**87**	0	32	**5**	54	**78**	58.5	11	11	10.1038/s41586-021-03920-6
				y = product observed by LC/MS, not quantitated														

A significant portion of the literature for informers reports LCAP or Area Under the Curve, uncalibrated. The data in this table is limited to calibrated yields, isolated yields, or yields by CAD. Literature data has shown that isolated yields can vary significantly from LCAP (e.g., differences of 2× for LCAP vs isolated yield for **X1**).[18]

Entry 1. This work. Conditions are best conditions for each substrate. Yields are isolated yields after purification.

Entry 2. Best results chosen among conditions examined.[19] Yields are by CAD detection, uncorrected, which has been reported to have a mean error of ±26% (e.g., 60% ± 15% yield for **X1**).[20] In this case all of them contained the additive phthalimide. Data found in supporting information Excel files. LCAP yields and some isolated yields without phthalimide were reported earlier by Merck.[18]

Entry 3. Best results among 2° radicals generated from NHP esters.[21] Yields estimated from a mix of isolated yields and LCAP data supplied in Supporting Information. Yields are for a two-step, one-pot process and are reported relative to the limiting coupling partner rather than with respect to nickel (as was done in the manuscript), which equates to a 67 mol% Ni catalyst loading. This change was to facilitate comparing with catalytic methods in this table and to account for the fact that some amount of turnover is observed in these reactions.

Entry 4. Best results among any alkyl coupling partners reported for a given aryl bromide informer (a mix of NHP ester, alkyl halide, and N-alkyl pyridinium; mix of 1°/2°/3° coupling partners).[21] Yields are isolated yields after purification. Yields are for a two-step, one-pot process and are reported relative to the limiting coupling partner rather than with respect to nickel (as was done in the manuscript), which equates to a 67 mol% Ni catalyst loading. This change was to facilitate comparing with catalytic methods in this table and to account for the fact that some amount of turnover is observed in these reactions.

Entry 5. Results from the best single set of conditions (out of eight tested) for all 13 aryl bromide informers for Pd-catalyzed amine arylation.[22] Yields are calibrated UPLC yields vs an internal standard.

Entry 6. Best yields for each given informer from among eight different conditions for Pd-catalyzed amine arylation.[22] Yields are calibrated UPLC yields vs an internal standard.

Entry 7. Results from the best single set of conditions (out of ten tested) for all 13 aryl bromide informers for Cu-catalyzed amine arylation.[22] Yields are calibrated UPLC yields vs an internal standard.

Entry 8. Best yields for each given informer from among ten different conditions for Cu-catalyzed amine arylation.[22] Yields are calibrated UPLC yields vs an internal standard.

Entry 9. Yields are the best yields among four conditions tested (in all cases XPhos Pd G3, THF, 50 °C, 18 h).[23] Yields are calibrated UPLC yields vs an internal standard.

Entry 10. Yields are isolated yields after purification.[24] In the table, "y" indicates that product was observed by UPLC, but no yield was obtained.

Entry 11. Yields are 1H NMR yields of the unpurified, isolated products.[25]

5. Mechanistic Studies

5.1 Reactivity of TMS-DHP with Aryl Bromide[a]

Reactions were set up in an N_2 filled glove box. A stock solution of catalyst was prepared by charging an oven-dried 1-dram vial with $NiBr_2$(dme) (4.9 mg, 0.016 mmol, 16 mol%), dtbbpy (4.3 mg, 0.016 mmol, 16 mol%) and 400 μL toluene, which was stirred at 40 ºC for 30 min. In a

separate 1-dram vial with a PTFE-coated stir bar, ethyl-4-bromobenzoate (15.5 µL, 0.10 mmol, 1.0 equiv), Na_2CO_3 (21.2 mg, 0.20 mmol, 2.0 equiv), 1,3,5-trimethoxybenzene (16.8 mg, 0.10 mmol, 1.0 equiv as an internal standard) were added sequentially. Then, either toluene (200 µL) or catalyst stock solution (200 µL) was added. Then TMS-DHP reductant (45.3 mg, 0.20 mmol, 2.0 equiv) was added. The reaction vial was sealed with a screw cap fitted with PTFE-faced silicone septa and removed from the glovebox. The reaction was allowed to stir (1250 RPM) at 80 °C for the specified time (18 h or 1 h). After 18 h (or 1 h), the reaction was worked up by GC analysis as reported in **section 2.1.**

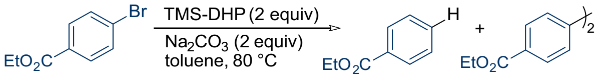

Entry	Conditions	Aryl-H	Aryl dimer	Returned ArBr
1	No catalyst, 18 h	0%	0%	100%
2	$NiBr_2$(dme)/dtbbpy (8 mol%), 1 h	0%	96%	0%

[a]Reactions run on a 0.1 mmol scale in 200 µL of solvent. Yields were determined by GC analysis calibrated against 1,3,5-trimethoxybenzene as an internal standard.

5.2 Reactivity of TMS-DHP with NHP Ester[a]

5.2.1 Reduction of NHP Ester with Si-DHP in Toluene

Reactions were set up in an N_2 filled glove box. A stock solution of catalyst was prepared by charging an oven-dried 1-dram vial with $NiBr_2$(dme) (4.9 mg, 0.016 mmol, 16 mol%), dtbbpy (4.3 mg, 0.016 mmol, 16 mol%) and 400 µL toluene, which was stirred at 40 ºC for 30 min. In another 1-dram vial with a PTFE-coated stir bar, NHP ester **2a** (30.9 mg, 0.10 mmol, 1.0 equiv), Na_2CO_3 (21.2 mg, 0.20 mmol, 2.0 equiv), 1,3,5-trimethoxybenzene (16.8 mg, 0.10 mmol, 1.0 equiv as an internal standard) were added sequentially. Then toluene (200 µL) or catalyst solution (200 µL) was added to the reaction vial. Lastly, the corresponding Si-DHP reductant (0.20 mmol, 2.0 equiv) was added. The reaction vial was sealed with a screw cap fitted with PTFE-faced silicone septa and removed from the glovebox. The reaction was allowed to stir (1250 RPM) at 80 ºC for the specified time (12 h or 1 h). After 18 h (or 1 h), the reaction was worked up by GC analysis as reported in **section 2.1**.

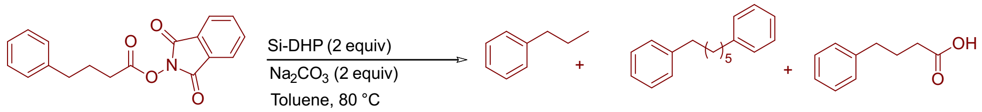

Entry	Conditions	Alkyl-H	Alkyl dimer	Alkyl-CO_2H	Returned NHP ester
1	TMS-DHP, 1 h	3%	0%	4%	88%
2	TMS-Me_4DHP, 12 h	10%	0%	3%	77%
3	$NiBr_2$(dme)/dtbbpy, Me_4DHP-TMS (8 mol%), 1 h	12%	54%	23%	0%

[a]Yields were determined by GC analysis calibrated against 1,3,5-trimethoxybenzene as an internal standard.

We also the above reaction (NHP ester **2a** and TMS-DHP) in toluene-d_8 and monitored the reaction by NMR (^{1}H and ^{13}C). Negligible consumption of NHP ester **2a** was observed after heating for 1 h at 80 °C. See spectra in Section 8.

Procedure: An oven dried 1-dram vial with a PTFE-coated stir bar was charged with the NHP ester **2a** (30.9 mg, 0.10 mmol, 1.0 equiv) and Na_2CO_3 (21.2 mg, 0.20 mmol, 2.0 equiv). Then toluene-d_8 (200 µL) was added to the reaction vial. Lastly, TMS-DHP reductant (45.3 mg, 0.20 mmol, 2.0 equiv) was added. The reaction vial was sealed with a screw cap fitted with PTFE-faced silicone septa and removed from the glovebox. The reaction was allowed to stir (1250 RPM) at 80 ºC for 1 h. After 1 h, the reaction was cooled down inside the glovebox antechamber for 15 min and subsequently taken inside the glovebox. The reaction mixture was passed through glass wool to remove the insoluble Na_2CO_3, transferred into an NMR tube, and diluted with toluene- d_8 (0.4 mL). The NMR tube was capped with a gas-tight white rubber septa cap and taken outside the glovebox. The ^{1}H and ^{13}C NMR of the reaction mixture matched with the corresponding NMR spectra of the pure substrate **2a** (figures S6 and S7, **Section 8**).

5.2.2 Reduction of NHP Ester with Si-DHP in DMA

Reactions were set up using the same procedure as above (**section 5.2.1**). The results are tabulated below.

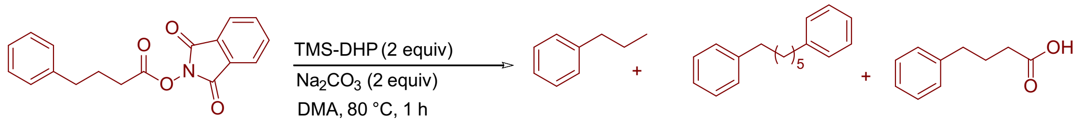

Entry	Conditions	Alkyl-H	Alkyl dimer	Alkyl-CO_2H	Returned NHP ester
1	TMS-DHP, 1 h	16%	0%	14%	0%[b]
2	$NiBr_2$(dme)/dtbbpy, (8 mol%), 1 h	0%	86%	14%	0%

[a]Yields were determined by GC analysis calibrated against 1,3,5-trimethoxybenzene as an internal standard. [b]The remaining alkyl mass balance (70%) was difficult to track by GC analysis.

Our data demonstrates that there is direct reduction of the NHP ester by TMS-DHP reductant in DMA at 80 °C.

5.2.3 Reduction of NHP Ester with TMS-DHP In the Presence of Different Additives

Reactions were set up in an N_2 filled glove box. A stock solution of catalyst was prepared by charging an oven-dried 1-dram vial with $NiBr_2$(dme) (4.9 mg, 0.016 mmol, 16 mol%), dtbbpy (4.3 mg, 0.016 mmol, 16 mol%) and 400 µL DMA which was stirred at 40 ºC for 30 min. In another 1-dram vial with a PTFE-coated stir bar, the corresponding NHP ester (0.10 mmol, 1.0 equiv), addtive(s) (0.15 mmol or 0.20 mmol, 1.5 equiv or 2.0 equiv), 1,3,5-trimethoxybenzene (16.8 mg, 0.10 mmol, 1.0 equiv as an internal standard) were added sequentially. Then solvent (DMA or toluene, 200 µL) or catalyst solution in DMA (200 µL) was added to the reaction vial. Lastly, the TMS-DHP reductant (45.3 mg, 0.20 mmol, 2.0 equiv) was added. The reaction vial was sealed with a screw cap fitted with PTFE-faced silicone septa and removed from the glovebox. The reaction was allowed to stir (1250 RPM) at the specified temperature (40 °C or 80 °C) for 1 h. After 1 h, the reaction was worked up by GC analysis as reported in **section 2.1**. The results are presented in the tables below.

Reaction with NHP ester of 4-phenylbutyric acid

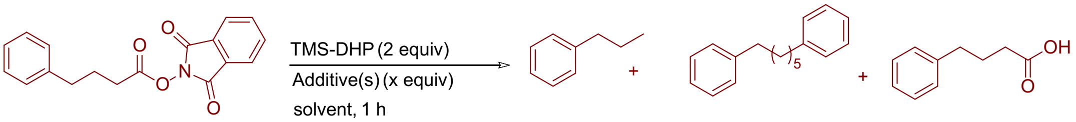

Entry	Additive	Alkyl-H	Alkyl dimer	Alkyl-CO_2H	Returned NHP ester
1	No additive, DMA, 40 °C	13%	0%	14%	0%[b]
2	NaBr (2 equiv), Na_2CO_3 (2 equiv) in DMA, 80 °C	18%	0%	14%	0%[b]
3	NaBr (2 equiv), Na_2CO_3 (2 equiv) in toluene, 80 °C	0%	0%	5%	89%
4	TMS-Br (1.5 equiv) in DMA, 40 °C	0%	0%	4%	83%
5	$NiBr_2$(dme)/dtbbpy (8 mol%) in DMA, 40 °C	0%	90%	10%	0%

[a]Yields were determined by GC analysis calibrated against 1,3,5-trimethoxybenzene as an internal standard. [b]The remaining alkyl mass balance (70%) was difficult to track by GC analysis.

Reaction with NHP ester of 2-phenylpropionic acid

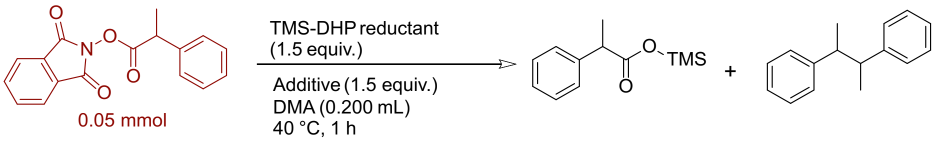

Entry	Additive	Alkyl-H	Alkyl dimer	Alkyl-CO_2H	Returned NHP ester
1	TMS-Br (1.5 equiv) in DMA, 40 °C	0%	0%	4%	100%
2	$NiBr_2$(dme)/dtbbpy (8 mol%), 40 °C	0%	0.32 (Pdt/I.S.)	0.06 (Pdt/I.S.)	0%

Discussion: While Yang and Reisman observed that TMS-Br accelerated reduction of NHP esters by TDAE,[26] we observe that TMS-Br slows reduction by TMS-DHP. For this purpose, we chose to study the effect of TMS-Br on the NHP esters of 4-phenyl butyric acid (the key acid in our study) and 2-phenylpropionic acid (the key substrate in the Yang and Reisman study). In both cases, we observed mostly returned NHP ester in the presence of TMS-DHP and excess TMS-Br. However, we observed complete conversion of the NHP esters to the alkyl dimer (major product) within an hour when we excluded TMS-Br but added 8 mol% $NiBr_2$(dme)/dtbbpy and 1.5 equiv TMS-DHP in DMA. This suggests that the mechanism of activation of the NHP ester is different for the two homogeneous reductants (TDAE versus TMS-DHP).

5.3 Reduction of (dtbbpy)$NiCl_2$ With Various Chemical Reductants

Entry	Conditions	(dtbbpy)Ni(COD)	$Ni(COD)_2$	Total Ni(0)
1	TMS-DHP, NaOAc (2 equiv)	72%	24%	96%
2	Zn, LiCl (10 equiv)	52%	12%	64%
3	TDAE	0%	0%	0%

5.3.1 Reduction of (dtbbpy)NiCl$_2$ with TMS-DHP

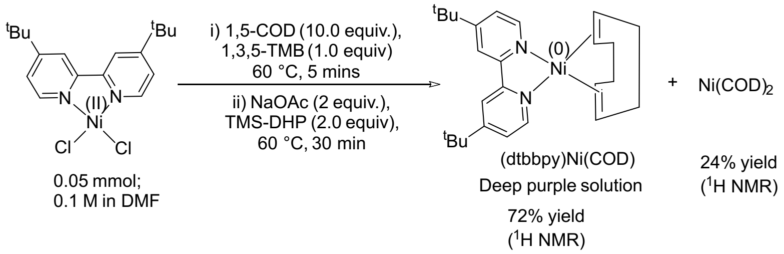

Reactions were set up in an N_2 filled glove box. In a 1-dram vial with a PTFE-coated stir bar, $NiCl_2$(dme) (11.0 mg, 0.05 mmol, 1.0 equiv) and dtbbpy (13.4 mg, 0.05 mmol, 1.0 equiv) were added, followed by DMF (500 µL). The resultant green solution was stirred at 60 ºC for 30 min. Thereafter, 1,5-cyclooctadiene (61 µL, 0.5 mmol, 10.0 equiv) and 1,3,5-trimethoxybenzene (8.7 mg, 0.05 mmol, 1.03 equiv) were added to the vial and the mixture was stirred for 5 min at 60 ºC. Then, NaOAc (8.2 mg, 0.1 mmol, 2.0 equiv) was added followed by TMS-DHP (22.6 mg, 0.1 mmol, 2.0 equiv). The reaction vial was sealed with a screw cap fitted with PTFE-faced silicone septa and stirred (1250 RPM) for 30 min at 60 ºC inside the glovebox. The reaction mixture immediately changed color to a deep brown upon adding TMS-DHP and gradually developed a deep purple color characteristic of (dtbbpy)Ni(cod) within 15 min. After 30 min, the vial was cooled down over 10 mins, the cap was opened and the solution was filtered through a 0.2 µm syringe filter into a 1-dram vial, to get rid of any undissolved residue. The resultant deep purple solution (in DMF) was transferred to a J-Young NMR tube, taken outside the glove box and submitted for a no-D quantitative ^{1}H NMR with solvent suppression (suppression of the DMF peaks). The relaxation time was set to 30 s and number of scans to 16 to get accurate peak integrations. The spectrum was automatically phase and baseline corrected to provide a stable baseline. The crude ^{1}H NMR showed peaks characteristic of (dtbbpy)Ni(cod) and Ni(cod)$_2$(**Figure S1**, **section 8**), which was confirmed by comparison with a previous report.[27]

Upon integrating the peaks at δ 9.86 (1.40, 2H) and δ 3.63 (2.99, 4H) for (dtbbpy)Ni(cod)] versus δ 6.10 (3, 3H) [for 1,3,5-TMB], the ^{1}H NMR yield of (dtbbpy)Ni(cod) was determined to be 72%.

Upon integrating the peaks at δ 4.27 (1.92, 8H) and δ 2.10 (1.94, 8H) for Ni^0(cod)$_2$ versus δ 6.10 (3, 3H) [for 1,3,5-TMB], the ^{1}H NMR yield of Ni^0(cod)$_2$ was determined to be 24%.

5.3.2 Reduction of (dtbbpy)NiCl$_2$ with Zn/LiCl

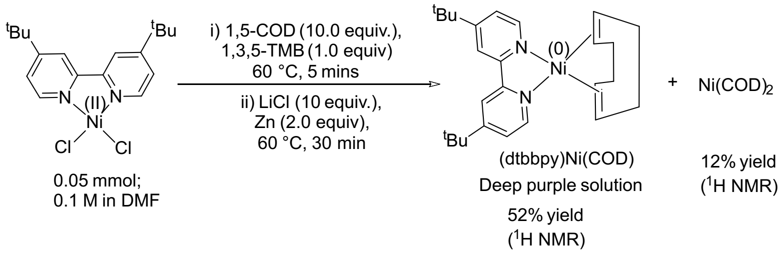

A procedure similar to **4.3.1** was used. Reactions were set up in an N_2 filled glove box. In a 1-dram vial with a PTFE-coated stir bar, $NiCl_2$(dme) (11.0 mg, 0.05 mmol, 1.0 equiv) and dtbbpy (13.4 mg, 0.05 mmol, 1.0 equiv) were added, followed by DMF (500 µL). The resultant green solution was stirred at 60 ºC for 30 min. Thereafter, 1,5-cyclooctadiene (61 µL, 0.5 mmol, 10.0 equiv) and 1,3,5-trimethoxybenzene (8.7 mg, 0.05 mmol, 1.03 equiv) were added to the vial and

the mixture was stirred for 5 min at 60 ºC. Then, LiCl (21.2 mg, 0.5 mmol, 10.0 equiv) was added followed by activated Zn dust (6.5 mg, 0.1 mmol, 2.0 equiv). The reaction vial was sealed with a screw cap fitted with PTFE-faced silicone septa and stirred (1250 RPM) for 30 min at 60 ºC inside the glovebox. [The reaction mixture gradually changed color from forest green to black to deep purple, characteristic of (dtbbpy)Ni(cod) within 30 min]. After 30 min, the vial was cooled down over 10 mins, the cap was opened and the solution was filtered through a 0.2 μm syringe filter into a 1-dram vial, to get rid of any undissolved residue. The resultant deep purple solution (in DMF) was transferred to a J-Young NMR tube, taken outside the glove box and submitted for a no-D quantitative ^{1}H NMR with solvent suppression (suppression of the DMF peaks). The relaxation time was set to 30 seconds and number of scans to 16 to get accurate peak integrations. The spectrum was automatically phase and baseline corrected to provide a stable baseline. The crude ^{1}H NMR showed peaks characteristic of (dtbbpy)Ni(cod) and Ni(cod)$_2$ (**Figure S2**, **section 8**), which was confirmed by comparison with a previous report.[27]

Upon integrating the peaks at δ 9.86 (1.03, 2H) and δ 3.63 (2.10, 4H) [for (dtbbpy)Ni(cod)] versus δ 6.10 (3, 3H) [for 1,3,5-TMB], the ^{1}H NMR yield of (dtbbpy)Ni(cod) was determined to be 52%.

Upon integrating the peaks at δ 4.27 (0.94, 8H) Ni^0(cod)$_2$ versus δ 6.10 (3, 3H) [for 1,3,5-TMB], the ^{1}H NMR yield of Ni^0(cod)$_2$ was determined to be 12%.

5.3.3 Reduction of (dtbbpy)NiCl$_2$ with TDAE

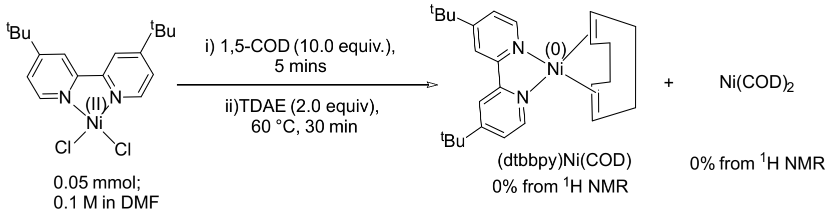

A procedure similar to **4.3.1** was used. Reactions were set up in an N_2 filled glove box. In a 1-dram vial with a PTFE-coated stir bar, NiCl$_2$(dme) (11.0 mg, 0.05 mmol, 1.0 equiv) and dtbbpy (13.4 mg, 0.05 mmol, 1.0 equiv) were added, followed by DMF (500 μL). The resultant green solution was stirred at 60 ºC for 30 min. Thereafter, 1,5-cyclooctadiene (61 μL, 0.5 mmol, 10.0 equiv) and 1,3,5-trimethoxybenzene (8.7 mg, 0.05 mmol, 1.03 equiv) were added to the vial and the mixture was stirred for 5 min at 60 ºC. Then TDAE (23.3 μL, 0.1 mmol, 2.0 equiv) was added and the reaction vial was sealed with a screw cap fitted with PTFE-faced silicone septa and stirred (1250 RPM) for 30 min at 60 ºC inside the glovebox. [The reaction mixture gradually changed color from forest green to brown in 30 min, however brown solution did not change color even when heating at 60 ºC for 24 h]. After 30 min, the vial was cooled down over 10 mins, the cap was opened and the solution was filtered through a 0.2 μm syringe filter into a 1-dram vial, to get rid of any undissolved residue. The resultant deep purple solution (in DMF) was transferred to a J-Young NMR tube, taken outside the glove box and submitted for a no-D quantitative ^{1}H NMR with solvent suppression (suppression of the DMF peaks). The relaxation time was set to 30 seconds and number of scans to 16 to get accurate peak integrations. The spectrum was automatically phase and baseline corrected to provide a stable baseline. The crude ^{1}H NMR showed broad paramagnetic peaks representative of Ni(II) and/or Ni(I) species present in solution. However, no peaks characteristic of (dtbbpy)Ni(cod) or Ni(cod)$_2$ were observed.

5.4 Reduction Studies of (dtbbpy)$NiBr_2$ in the Presence of TMS-DHP or TMS -Me_4DHP

5.4.1 Quantitation of Reduced Nickel with Iodocumene Trap

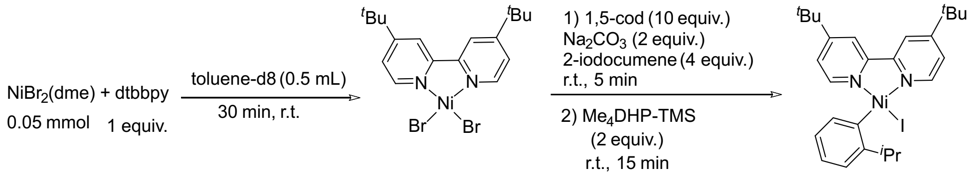

complex-1

characterized by ^{1}H NMR
60% NMR yield wrto 1,3,5-TMB;
UV-Vis absorbance at λ = 512 nm

Reactions were set up in an N_2 filled glove box. In a 1-dram vial with a PTFE-coated stir bar, $NiBr_2$(dme) (30.9 mg, 0.1 mmol, 1.0 equiv) and dtbbpy (26.8 mg, 0.1 mmol, 1.0 equiv) were added, followed by toluene-d_8 (500 µL). The resulting orange suspension was stirred at rt (30 ºC) for 30 min when the solution changed to a pale green suspension. Thereafter, 1,5-cyclooctadiene (130 µL, 1.0 mmol, 10.0 equiv), Na_2CO_3 (21.2 mg, 0.2 mmol, 2.0 equiv), 2-iodocumene (63.9 µL, 0.4 mmol, 4.0 equiv) and 1,3,5-trimethoxybenzene (13.9 mg, 0.08 mmol, 0.8 equiv) were added to the vial and the mixture was stirred for 5 min at 30 ºC. Then TMS-Me_4DHP (56.5 mg, 0.2 mmol, 2.0 equiv) was added, the reaction vial was sealed with a screw cap fitted with PTFE-faced silicone septa and stirred (1250 RPM) for 15 min at 30 ºC inside the glovebox. The reaction mixture changed to a deep red color upon stirring for 5 min. After 15 min, the stirring was stopped, the cap was opened and the solution was filtered through a 0.2 µm syringe filter into a 1-dram vial, to get rid of any undissolved residue. The resultant deep red solution (in toluene-d_8) was transferred to a J-Young NMR tube, taken outside the glove box and submitted for a quantitative ^{1}H NMR (d1 = 30 s). The crude ^{1}H NMR showed peaks that matched peaks observed for the isolated (dtbbpy)Ni(2-cumenyl)(I) in toluene-d_8 (**Figure S3**, **section 8** and *vide infra*).

^{1}H NMR (500 MHz, toluene-d_8) δ 9.71 (d, J = 5.0 Hz, 1H), 7.85 (d, J = 10.0 Hz, 1H), 7.36 (d, J = 15.0 Hz, 2H), 6.96–6.94 (m, 2H, overlap with toluene peaks in toluene-d_8), 6.85–6.83 (m, 2H, overlap with toluene peaks in toluene-d_8), 6.64 (d, J = 5.0 Hz, 1H), 6.25 (d, J = 6.1 Hz, 1H), 5.27–5.22 (m, 1H, overlaps with the 1,5-COD peak), 1.48 (d, J = 5.0 Hz, 3H), 1.19 (d, J = 5.0 Hz, 3H), 0.98 (s, 9H), 0.87 (s, 9H). UV-Vis(toluene): λ_{max} = 512 nm.

The pre-formed (dtbbpy)Ni(2-cumene)(I) complex was synthesized following the literature procedure.[27] The complex was sampled in toluene-d_8 for a ^{1}H NMR and showed the following peaks (**Figure S4**, **section 8**): **^{1}H NMR (500 MHz, toluene-d_8)** δ 9.94 (d, J = 5.0 Hz, 1H), 8.15 (d, J = 5.0 Hz, 1H), 7.29 (d, J = 10.0 Hz, 2H), 7.17 (d, J = 5.0 Hz, 2H), 7.07 (d, J = 5.0 Hz, 2H; merges with residual toluene peak in toluene-d_8), 6.59 (d, J = 10.0 Hz, 1H), 6.24 (d, J = 5.0 Hz, 1H), 5.61 (m, 1H), 1.73 (d, J = 5.0 Hz, 3H), 1.38 (d, J = 5.0 Hz, 3H), 1.00 (s, 9H), 0.89 (s, 9H). UV-Vis(toluene): λ_{max} = 512 nm.

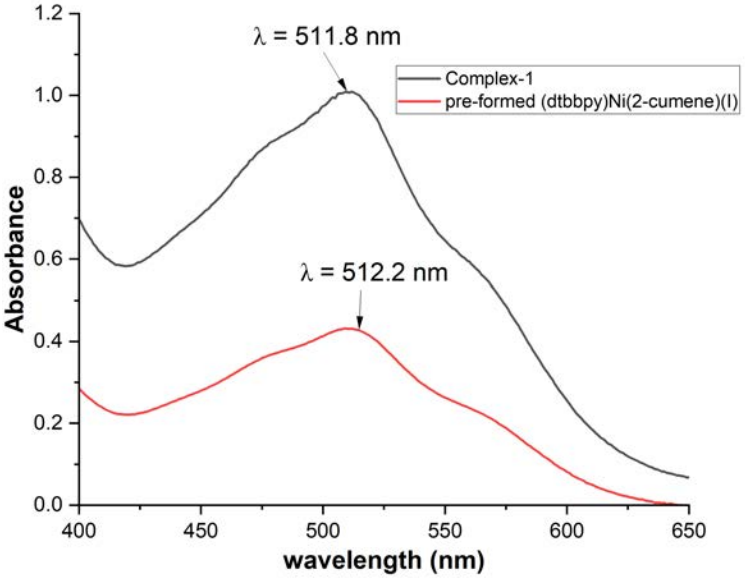

5.4.2 Quantitation of Reduced Nickel with Alkene and Cyclopentadieneone Traps.

5.4.2.1 Reduction with TMS-Me$_4$DHP Reductant

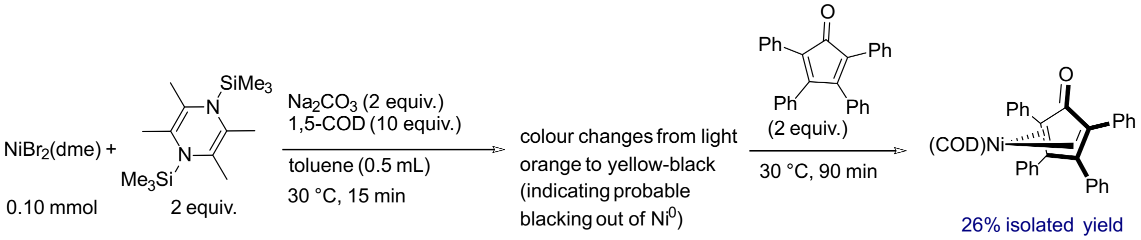

Reactions were set up in an N_2 filled glove box. In a 1-dram vial with a PTFE-coated stir bar, $NiBr_2$(dme) (30.9 mg, 0.1 mmol, 1.0 equiv), Na_2CO_3 (21.2 mg, 0.2 mmol, 2.0 equiv) and 1,5-cyclooctadiene (613.3 µL, 5.0 mmol, 50.0 equiv) were added, followed by toluene (500 µL). The pale orange suspension was stirred at rt (30 ºC) for 5 min and then TMS-Me$_4$DHP (56.5 mg, 0.2 mmol, 2.0 equiv) was added. The resulting mixture was stirred at rt for 15 min, resulting in a black solution. Thereafter, tetraphenylcyclopentadienone (76.9 mg, 0.2 mmol, 2.0 equiv) was added and the mixture was stirred (1250 RPM) for 90 min at 30 ºC. After 90 min, the stirring was stopped and the vial was taken outside the glove box where it was filtered through a 2 cm silica plug into a scintilation vial, to get rid of any undissolved residue. The silica plug was further washed with dichloromethane (2×3 mL). The resultant filtrate was dried down in a rotary evaporator and then dichloromethane (5 mL) was added followed by silica gel. The silica slurry was dried down in a rotary evaporator and purified by normal phase silica gel column chromatography (on a Sfar Duo 10 g column) using 100% hexanes (12 CV), 0–55% EtOAc/ hexanes (12–30 CV; the product peak separated at 37–40% EtOAc/hexanes). The product was isolated as a brown-black solid [cod)Ni(cpd)] in a 26% crude yield (**Figure S5**, **section 8**). **^{1}H NMR (500 MHz, CDCl$_3$)** δ 7.72–7.70 (m, 4H), 7.29 (t, J = 7.5 Hz, 4H), 7.25–7.22 (m, 2H), 7.20–7.17 (m, 2H), 7.08 (t, J = 7.5 Hz, 4H), 6.87 (d, J = 10.0 Hz, 4H), 4.31–4.26 (m, 2H), 4.09–4.04 (m, 2H), 2.79–2.72 (m, 2H), 2.53–2.45 (m, 2H), 2.37–2.29 (m, 2H), 2.10–2.04 (m, 2H). The NMR data matched the reported spectrum.[28] **HRMS (ESI-MS)** m/z: $[M+H]^+$ calcd for $[C_{37}H_{32}NiO+H]^+$ 551.1879; found 551.1877.

5.4.2.2 Reduction with TMS-DHP Reductant

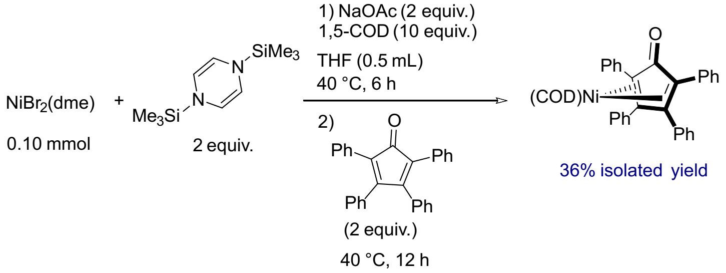

Reactions were set up in an N_2 filled glove box. In a 1-dram vial with a PTFE-coated stir bar, $NiBr_2$(dme) (30.9 mg, 0.1 mmol, 1.0 equiv), NaOAc (16.4 mg, 0.2 mmol, 2.0 equiv) and 1,5-cyclooctadiene (122.6 µL, 1.0 mmol, 10.0 equiv) were added, followed by THF (500 µL). The pale orange suspension was stirred at rt (30 ºC) for 5 min and then TMS-DHP (45.3 mg, 0.2 mmol, 2.0 equiv) was added. The resulting mixture was stirred at 40 ºC for 6 h when the solution changed to a black suspension. Thereafter, tetraphenylcyclopentadienone (76.9 mg, 0.2 mmol, 2.0 equiv) was added and the mixture was stirred (1250 RPM) for 12 h at 40 ºC. After 12 h, the stirring was stopped, the vial was cooled down to rt and taken outside the glove box where it was filtered through a 2 cm silica plug into a scin vial, to get rid of any undissolved residue. The silica plug was further washed with dichloromethane (2×3 mL). The resultant filtrate was dried down in a rotary evaporator and then dichloromethane (5 mL) was added followed by silica gel. The silica slurry was dried down in a rotary evaporator and purified by normal phase silica gel column chromatography (on a Sfar Duo 10 g column) using 100% hexanes (12 CV), 0–55% EtOAc/ hexanes (12–30 CV; the product peak separated at 37–40% EtOAc/hexanes). The product was isolated as a brown-black solid in a 36% isolated yield. The NMR data matched the reported spectrum[28] and the spectrum for (cod)Ni(cpd) obtained in section **5.4.2.1**.

5.5 Stoichiometric Reaction of (dtbbpy)Ni(COD) with NHP Ester 2a

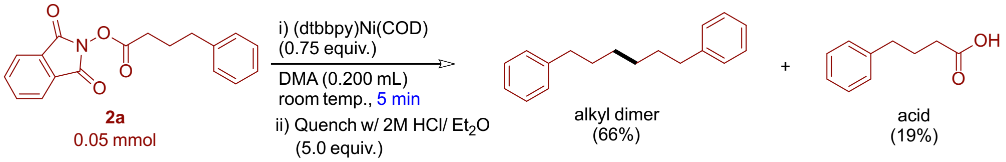

In an N_2 glovebox a scintillation vial was charged with a stir bar, $Ni(COD)_2$ (10.3 mg, 0.0375 mmol, 0.75 equiv, 75 mol%) and dtbbpy (10.1 mg, 0.0375 mmol, 0.75 equiv, 75 mol%). DMA (200 µL was added resulting in a dark purple solution that was allowed to stir at rt for 2 h inside the glovebox. Thereafter, the NHP ester **2a** (15.5 mg, 0.05 mmol, 1.0 equiv) was added, followed by 1,3,5-trimethoxybenzene (8.4 mg, 0.05 mmol, 1.0 equiv) as an internal standard. Then the reaction was allowed to stir at rt for 5 min as it changed color from deep purple to dark reddish brown. To this solution, HCl in Et_2O (500 µL of a 1 M solution of HCl in Et_2O) was added dropwise and the mixture was stirred for 5 minutes when the deep red solution turned into a pale yellow color. The resulting solution was filtered through a 2 cm celite plug and further washed with EtOAc (~1.8–2.0 mL) and collected in a GC vial. The sample was analyzed by GC using our standard method and the yields were determined based on the peak area of the analyte compared to 1,3,5-trimethoxybenzene as an internal standard. The alkyl dimer was formed in 66% yield with respect to NHP ester and 88% yield with respect to [Ni^0] (by GC).

5.6 Stoichiometric Reaction of (dtbbpy)Ni^{II}(Ar)(NPhth) with NHP Ester 2a

5.6.1 Synthesis of (dtbbpy)Ni^{II}(Ar)(NPhth) Complex

In an N_2 glovebox a scintillation vial was charged with a stir bar, Ni(COD)$_2$ (138 mg, 0.5 mmol, 1.0 equiv) and dtbbpy (134 mg, 0.5 mmol, 1.0 equiv). THF (1 mL was added resulting in a dark purple solution that was allowed to stir at rt for 12 h inside the glovebox. Thereafter, ethyl-4-bromobenzoate (458 mg, 327 µL, 2.0 mmol, 4.0 equiv) was added and the reaction was allowed to stir at rt for an additional 30 min as it changed color to dark reddish orange. To this solution, potassium phthalimide (93 mg, 0.5 mmol, 1.0 equiv) was added and as the solution was stirred at rt for 3 h, the reddish orange solution changed to a canary yellow color. Subsequently, pentane (18 mL) was added, and the mixture was stirred for 5 minutes to crash out a yellow solid which was filtered through a 10-micron PE fritted filter. The solid on the filter was subsequently washed with pentane (2×5 mL), transferred into a pre-weighed scintillation vial and dried under vacuum overnight (~ 12 h) to give a yellow solid as the desired product (274 mg, 88% yield). Further removal of THF under reduced pressure was unsuccessful. The complex showed a 95 wt% purity after drying under vacuum (containing 5 wt% residual THF).

^{1}H NMR (500 MHz, CDCl$_3$) δ 7.98 (d, *J* = 5.0 Hz, 2H), 7.92 (d, *J* = 5.0 Hz, 1H), 7.82 (br s, 1H), 7.77 (br s, 1H), 7.57 (d, *J* = 5.0 Hz, 4H), 7.42–7.40 (m, 2H), 7.34 (d, *J* = 5.0 Hz, 1H), 7.23 (d, *J* = 5.0 Hz, 1H), 7.11–7.09 (m, 1H), 4.27 (q, *J* = 10.0 Hz, 2H), 1.36 (s, 9H), 1.35 (s, 9H), 1.32 (t, *J* = 10.0 Hz, 3H).

^{13}C{^{1}H} NMR (126 MHz, CDCl$_3$) δ 180.2, 168.3, 166.2, 164.0, 163.5, 156.0, 153.1, 152.1, 148.7, 137.3, 135.9, 131.4, 125.9, 125.3, 123.9, 123.5, 120.8, 117.2, 117.0, 60.3, 35.6, 35.5, 30.4, 30.3, 14.5.

5.6.2 Stochiometric Reaction Between (dtbbpy)Ni^{II}(Ar)(NPhth) Complex and NHP Ester 2a

In an N_2 glovebox a scintillation vial was charged with a stir bar, (dtbbpy)Ni(Ar)(NPhth) complex (10.0 mg, 0.016 mmol, 1.0 equiv), the NHP ester **2a** (5.9 mg, 0.019 mmol, 1.2 equiv, *or*, 9.9 mg, 0.032 mmol, 2 equiv) was added, followed by 1,3,5-trimethoxybenzene (~2.7 mg, 0.016 mmol, 1.0 equiv) as an internal standard. After that, DMA (200 µL) was added, followed by TMS-DHP (7.2 mg. 0.032, 2.0 equiv; *in case of entries 1 and 2*). The reaction vial was sealed with a screw cap fitted with a PTFE faced silicone septum before being removed from the glovebox. The contents of the reaction vessel were stirred (1250 RPM) at room temperature (20–22 °C) for 2 h. Afterwards, 30 µL of the reaction mixture was filtered through a 2 cm celite plug and further washed with EtOAc (~1.8–2.0 mL) and collected in a GC vial. The sample was analyzed by GC using our standard method and the yields were determined based on the peak area of the analyte compared to 1,3,5-trimethoxybenzene as the internal standard.

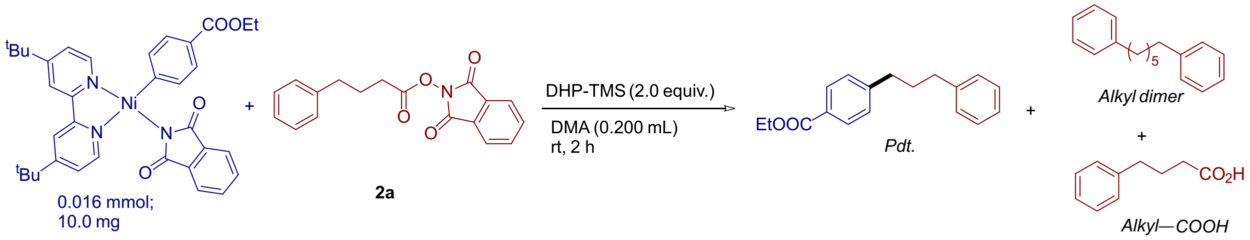

Entry	Conditions	Pdt	Alkyl dimer	Alkyl-CO_2H
1	2.0 equiv **2a**	73%	72%	12%
2	1.2 equiv **2a**	69%	31%	14%
3	TMS-DHP *not added*, 2.0 equiv **2a**, 80 ºC	4%	0%[a]	0%

[a]NHP ester completely returned (96%).

5.7 UV-Vis Study of the Reduction of Ni(II) to Ni(0)

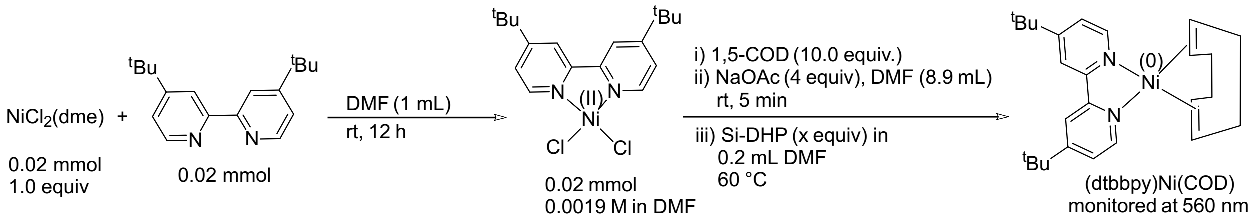

From the UV-Vis kinetic data, the rate of formation of (dtbbpy)Ni(cod) with different Si-DHP reductants followed the order: TMS-DHB >> TMS-DHP > TES-DHP ≈ TMS-Me_4DHP.

5.7.1 Dip-Probe Reactor Setup

Reactions were carried out in 24-mL scintillation vials equipped with septum caps featuring PTFE lined silicone SURE-LINK™ septa (ChemGlass), allowing for perforation of the caps with needles to introduce gas/samples. A corer was used to bore a hole into the septum cap, ensuring a gas-tight seal between the UV-Vis dip probe and the septum of the cap. The UV-Vis dip probe (2 mm path length, Agilent, part number 7910035700 for the body and 7910036000 for the 2 mm path length tip) was connected via fiber optic cables to a Cary 60 spectrophotometer. Before each experiment, the 100% transmission spectrum was collected in DMF used in the subsequent experiment, and the 0% transmission was collected after blocking the sample beam from entering the detector.

5.7.2 Procedure

In an N_2 glovebox a 20 mL scintillation vial was charged with a stir bar, $NiCl_2$(dme) (4.4 mg, 0.02 mmol, 1.0 equiv) and dtbbpy (5.4 mg, 0.02 mmol, 1.0 equiv). Degassed DMF (1 mL) was added resulting in a deep green solution that was allowed to stir at rt (30 ºC) for 12 h inside the glovebox. Thereafter, 1,5-cyclooctadiene (330.8 mg, 375 µL, 3.0 mmol, 152.9 equiv), NaOAc (6.6 mg, 0.08 mmol, 4.0 equiv) and DMF (8900 µL) were added, and the reaction mixture was allowed to stir at rt for an additional 5 min. To a separate 1-dram vial, the reductant was weighed out and dissolved in 200 µL of the specific solvent (see below for details) [*Total volume of the solution = 10.475 mL*]. The 1-dram vial was then sealed with a septa cap and taken outside the glovebox. Side by side, the 24 mL vial (containing the Ni/L pre-stir) was sealed with a PTFE-lined cap perforated with a stainless-steel dip probe. Then the sealed vial with the attached dip probe and the sealed 1-dram vial having the reductant solution were taken outside the glove box. The 24 mL scintillation vial with the attached dip probe was then placed on a Pie-block pre-heated at 60 ºC and the stirring was set to 900 rpm. To the stirring dispersion, 200 µL of the reductant solution

was added via a gas tight syringe, ensuring that the dip probe opening was fully submerged after complete addition of the reductant solution. Immediately after complete addition, the spectral acquisition sequence was initiated, ensuring the reaction was shielded from ambient light throughout the duration of the experiment. The absorbance at 560 nm, which is characteristic of (dtbbpy)Ni^0(cod), was monitored with time.

Note: Because of the extreme sensitivity of (dtbbpy)Ni^0(cod), the reaction was run in the presence of excess cod (152.9 equiv) and in freeze-pump-thawed DMF to stabilize this intermediate.

Data Analysis

Initial concentration of (dtbbpy)Ni(II)Cl_2 in solution = 0.02 mmol/ 10.475 mL = 1.9 mM. The absorbance at 560 nm[29] (characteristic of (dtbbpy)Ni(cod); ε_{560} = 9350 M^{-1} cm^{-1} in DMF) was monitored vs time. The concentration of (dtbbpy)Ni(cod) could be determined by the Beer-Lambert law:

$A = \varepsilon \times C \times \ell$

A = absorbance in AU, C = concentration in M, ℓ = path-length in cm, ε = extinction coefficient in M^{-1} cm^{-1}.

5.7.2.1 Reduction with TMS-DHP

Procedure **5.7.2** was followed. TMS-DHP (18.1 mg, 0.08 mmol, 4.0 equiv) was dissolved in DMF (200 μL) and added to the reaction vial by syringe at 60 ºC. The spectra were collected from 200 nm to 800 nm at a scan rate of 600 nm min^{-1} every 1.0 min for 60 min, followed by every 3 min for an additional 120 min and then every 5 min for the next 180 min (run time = 6 h).

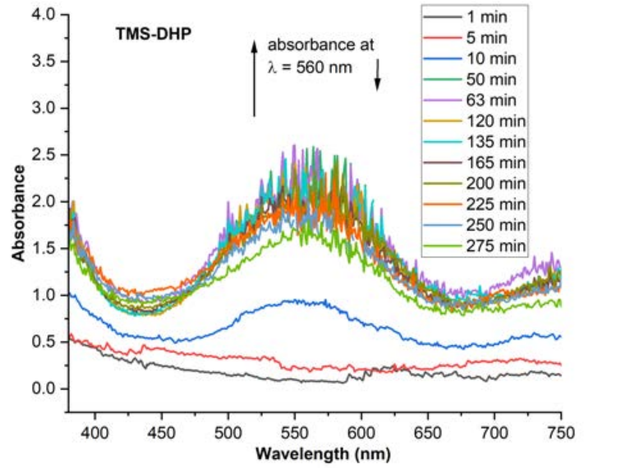

Image 1. Absorbance versus wavelength plot of TMS-DHP reductant.

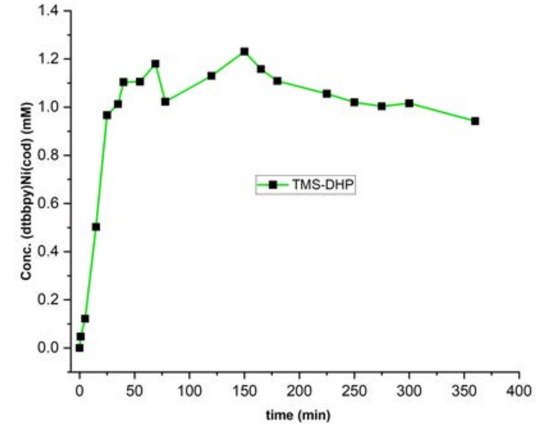

Image 2. Concentration of (dtbbpy)Ni(cod) plotted against time for TMS-DHP reductant.

The formation of (dtbbpy)Ni(cod) commenced at t ~ 15 min. The concentration of (dtbbpy)Ni(cod) at 69 min was found to be 1.1 mM (absorbance at 69 min = 2.04), which corresponds to 57% yield of (dtbbpy)Ni(cod). The maximum absorbance (A_{max}) was observed at t = 150 min; A_{max} (at 150 min) = 2.3022. After 150 min, the concentration of (dtbbpy)Ni(cod) gradually decreased, likely due to decomposition in solution (formation of nickel black observed).

Concentration of (dtbbpy)Ni(cod) at $A_{max} = C_{max} = A_{max} / (\varepsilon \times \ell) = [2.3022 / (9350 \times 0.2)] \times 1000$ mM = 1.2 mM

Maximum %yield of (dtbbpy)Ni^0(cod) = (1.2 / 1.9) × 100 = 65%

5.7.2.2 Reduction with TES-DHP

The typical procedure **5.7.2** was followed. TES-DHP (24.9 mg, 0.08 mmol, 4.0 equiv) was dissolved in DMF (200 μL) and added to the reaction vial by syringe at 60 ºC. The spectra were collected from 800 nm to 200 nm at a scan rate of 600 nm min^{-1} every 1.0 min for 60 min, followed by every 3 min for an additional 60 min and then every 5 min for the next 240 min (run time = 6 h). At this time we do not understand the origin of the observed induction period (about 150 min).

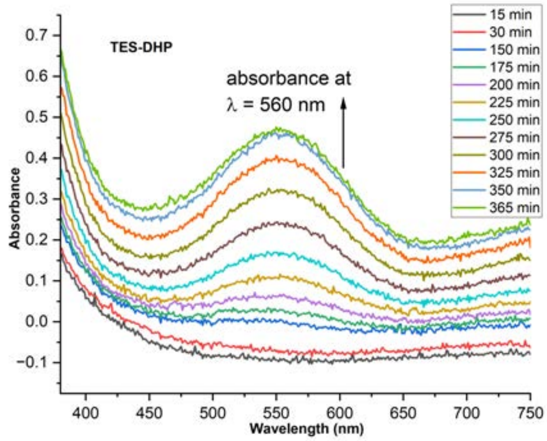

Image 3. Absorbance versus wavelength plot of TES-DHP reductant.

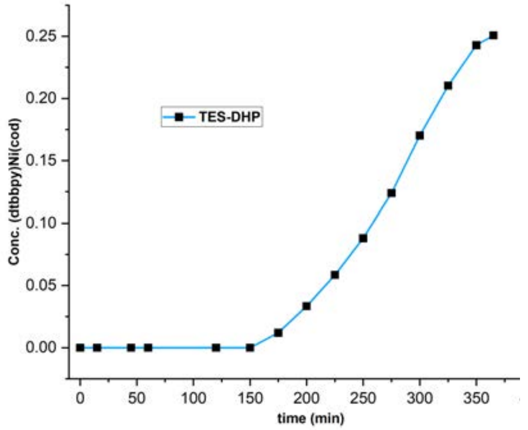

Image 4. Concentration of (dtbbpy)Ni(cod) plotted against time for TES-DHP reductant.

The formation of (dtbbpy)Ni(cod) commenced at t ~ 150 min. Even after it commenced, the reduction was slower than TMS-DHP (*vide supra*). The maximum absorbance (A_{max} at 560 nm) was recorded at t = 360 min (210 min after initiation). The run was stopped at 360 minutes, so this is not a reflection of the maximum conc. of (dtbbpy)Ni(cod) that could be obtained from this reductant. A_{max} (at 360 min) = 0.4688.

Concentration of (dtbbpy)Ni(cod) at $A_{max} = C_{max}$ = 0.2 mM;

Maximum %yield of (dtbbpy)Ni^0(cod) = (0.2 / 1.9) × 100 = 13%

5.7.2.3 Reduction with TMS-Me_4DHP

The typical procedure **5.7.2** was followed. TMS-Me_4DHP (45.2 mg, 0.16 mmol, 8.0 equiv) was dissolved in a 1:1 mixture of toluene and DMF (1:1, total 200 μL) and added to the reaction vial by syringe at 60 ºC. For the TMS-Me_4DHP reductant, spectra were collected from 800 nm to 200 nm at a scan rate of 600 nm min^{-1} every 3.0 min for 120 min, followed by every 5 min for an additional 420 min and then every 10 min for the next 180 min (run time = 12 h). With 4.0 equiv of TMS-Me_4DHP, the formation of Ni(0) could not be detected over time and we observed formation of nickel black. Upon increasing the reductant amount to 8.0 equiv, the formation of

(dtbbpy)Ni(cod) could be monitored from the appearance of the characteristic UV-Vis peak at λ = 560 nm. Again, we observed an induction period and in addition this reductant exhibited what appears to be autocatalytic behavior.

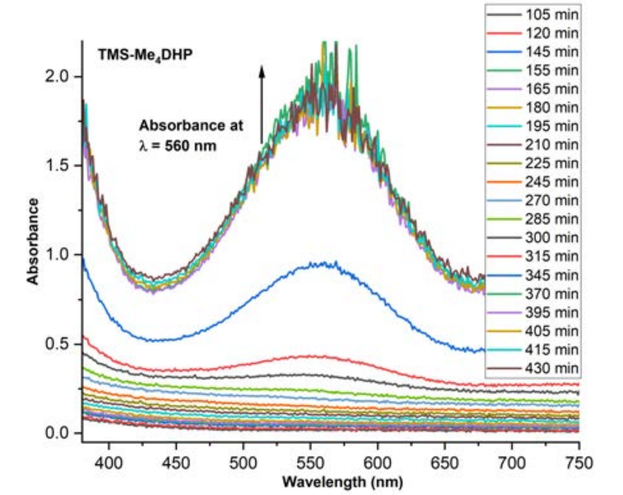

Image 5. Absorbance versus wavelength plot of TMS-Me4DHP reductant.

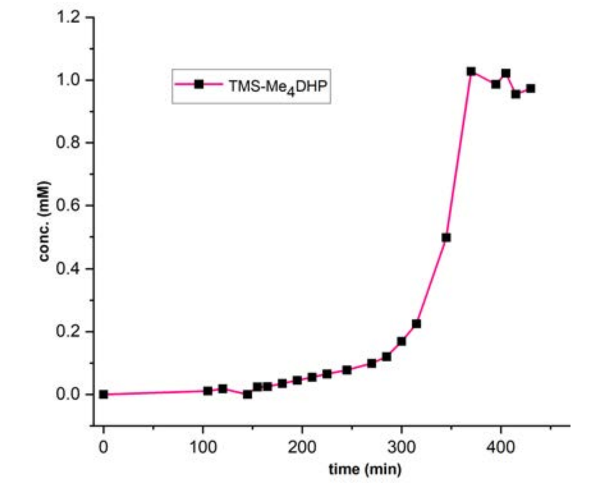

Image 6. Concentration of (dtbbpy)Ni(cod) plotted against time for TMS-Me4DHP reductant.

The formation of (dtbbpy)Ni(cod) commenced at t ~ 155 min and the overall rate of formation of (dtbbpy)Ni(cod) was significantly slower compared to TMS-DHP (*vide supra*). The maximum absorbance (A_{max} at 560 nm) was recorded at t ~ 370 min and the conc. of (dtbbpy)Ni(cod) from this point onward did not significantly change until the end of the experiment; A_{max} (at 369 min) = 1.9220. We note that once initiation occurred, the maximum rate observed is similar to TMS-DHP and the ultimate yield was similar as well.

Concentration of (dtbbpy)Ni(cod) at A_{max} = C_{max} = 1.0 mM;

Maximum %yield of (dtbbpy)Ni^0(cod) = (1.0 / 1.9) × 100 = 54%

5.7.2.4 Reduction with TMS-DHB

The typical procedure **5.7.2** was followed. TMS-DHB (6.1 mg, 0.02 mmol, 1.0 equiv) was dissolved in DMF (200 µL) and added to the reaction vial by syringe at 30 ºC. For the TMS-DHB reductant, spectra were collected from 800 nm to 200 nm at a scan rate of 600 nm min^{-1} every 30 s for the initial 30 min, followed by every 1.0 min for the next 30 min and then every 3 min for the next 60 min, then every 5 minutes for the next 240 min (run time = 6 h). The reaction temperature was maintained at 30 ºC throughout this experiment. The reduction of Ni(II) to Ni(0) was extremely fast with TMS-DHB. In presence of 1.0 equiv TMS-DHB at 30 °C, the formation of (dtbbpy)Ni(0)(cod) commenced within 30 seconds. After reaching a maximum absorbance at 5 min, the concentration of (dtbbpy)Ni(0)(cod) started to steadily decrease in solution and the deep purple color turned into brick red followed by nickel blacking out after some time.

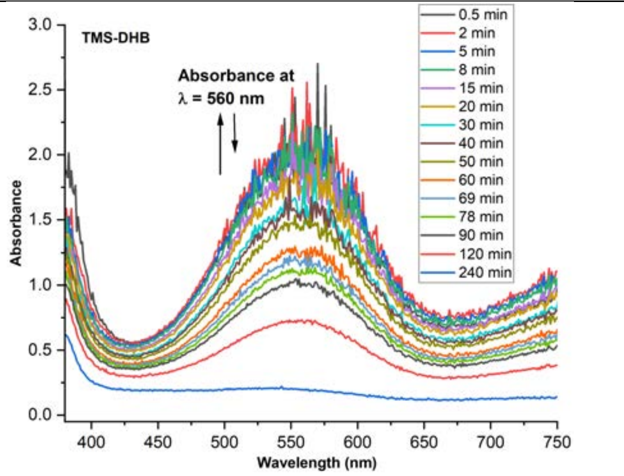

Image 7. Absorbance versus wavelength plot of TMS-DHB reductant.

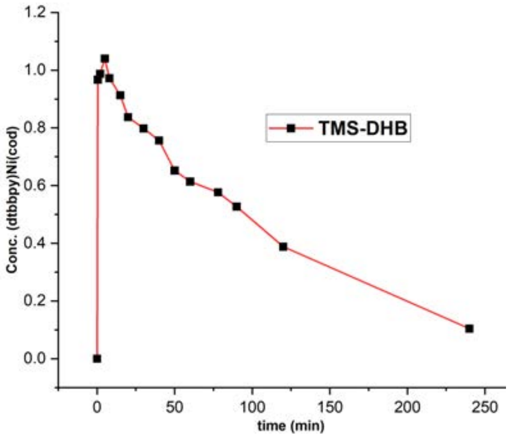

Image 8. Concentration of (dtbbpy)Ni(cod) plotted against time for TMS-DHB reductant.

The formation of (dtbbpy)Ni(cod) commenced at t ~ 30 seconds and the overall rate of formation of (dtbbpy)Ni(cod) was significantly faster compared to TMS-DHP (*vide supra*). The maximum absorbance (A_{max} at 560 nm) was recorded at t = 5 min and the conc. of (dtbbpy)Ni(cod) after this point showed a steady decrease over time; A_{max} (at 5 min) = 1.9455.

Concentration of (dtbbpy)Ni(cod) at $A_{max} = C_{max} = 1.0$ mM;

Maximum %yield of (dtbbpy)Ni^0(cod) = (1.0 / 1.9) × 100 = 55%

5.8 Effect of Solvent on the Rate of the Cross-Electrophile Coupling Reaction

5.8.1. Effect of Solvent on the Rate of XEC Between an Activated Aryl Bromide (1) and a 1° NHP Ester (2a)

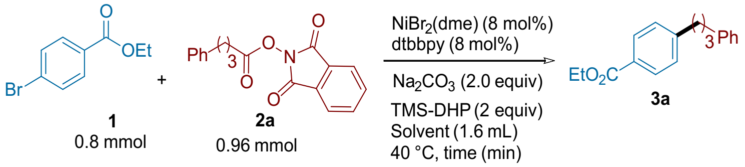

In an N_2 filled glove box, a catalyst solution was prepared by charging an oven-dried 1-dram vial with a PTFE-coated stir bar, $NiBr_2$(dme) (19.8 mg, 0.064 mmol, 8 mol%), and dtbbpy (17.2 mg, 0.064 mmol, 8 mol%). The solids were dissolved in the corresponding solvent (1000 μL of DMA or THF or toluene) and allowed to stir at 40 °C for 30 min. In a separate 1-dram vial, the NHP ester **2a** (296.9 mg, 0.96 mmol, 1.2 equiv), Na_2CO_3 (169.6 mg, 1.6 mmol, 2.0 equiv), ethyl-4-bromobenzoate (130.6 µL, 0.80 mmol, 1.0 equiv), 1,3,5-trimethoxybenzene (134.6 mg, 0.80 mmol, 1.0 equiv as an internal standard) were added sequentially. Then 1000 µL of the $NiBr_2$(dme)/dtbbpy prestirred solution was added to the reaction mixture followed by rinsing the prestir vial with an additional 600 µL solvent and transferring it to the reaction vial. Finally, the TMS-DHP reductant (362.4 mg, 1.6 mmol, 2.0 equiv) was added to the reaction mixture and the reaction vial was sealed with a screw cap fitted with PTFE-faced silicone septa and removed from

the glovebox. The reaction was allowed to stir (1250 RPM) at 40 °C and aliquots (10 μL) were withdrawn from the reaction vial at specific time intervals followed by work up and GC analysis following the general procedure listed in **section 2.1**.

(a) Reaction profile in DMA

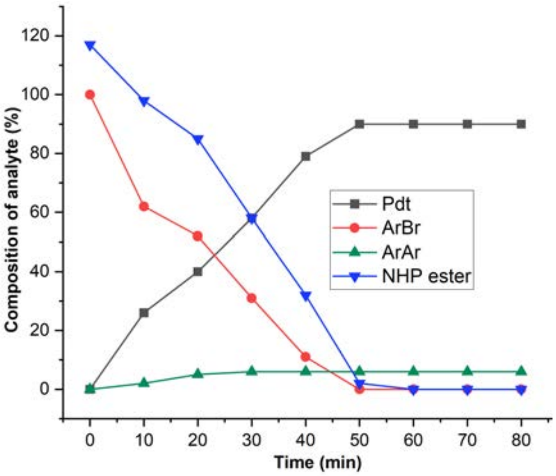

(b) Reaction profile in THF

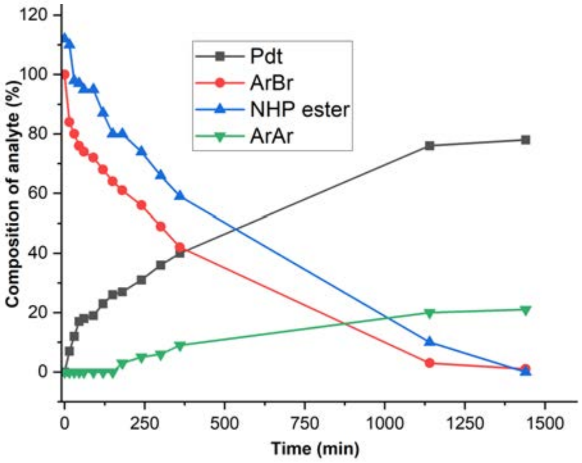

(c) Reaction profile in toluene

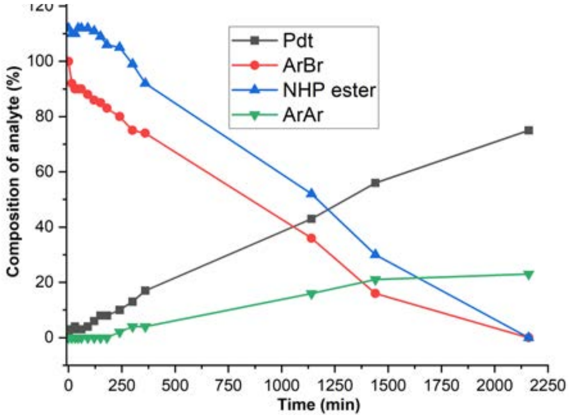

Solvent	Time (min)	3a (%)*	1 (%)
DMA	10	26	62
	20	40	52
	50	90	0
THF	150	26	64
	360	40	42
	1140	76	3
Toluene	360	17	74
	1140	43	36
	2160	76	0

*The remaining aryl mass balance corresponds to the aryl dimer.

5.8.2. Effect of Solvent on the Rate of XEC Between a Deactivated Aryl Bromide (4-Bromo anisole) and 1°, 2°, and Strained Ring 3° NHP Esters

In a N_2 filled glove box, a catalyst solution was prepared by charging an oven-dried 1-dram vial with a PTFE-coated stir bar, $NiBr_2$(dme) (19.8 mg, 0.064 mmol, 8 mol%), and dtbbpy (17.2 mg, 0.064 mmol, 8 mol%). The solids were dissolved in the corresponding solvent (1600 μL of DMA or toluene) and allowed to stir at 40 °C for 30 min. The reactions for different time points were set up in parallel as follows. In separate 1-dram vials, the corresponding NHP ester (0.12

mmol, 1.2 equiv), Na_2CO_3 (169.6 mg, 1.6 mmol, 2.0 equiv), 4-bromo anisole (12.5 µL, 0.10 mmol, 1.0 equiv), 1,3,5-trimethoxybenzene (16.8 mg, 0.10 mmol, 1.0 equiv as an internal standard) were added sequentially. Then 200 µL of the $NiBr_2$(dme)/dtbbpy solution was added to the reaction mixture. Finally, the Si-DHP reductant (0.20 mmol, 2.0 equiv) was added to the reaction mixture and the reaction vial was sealed with a screw cap fitted with PTFE-faced silicone septa and removed from the glovebox. The reaction vials were allowed to stir (1250 RPM) at 60 °C for the specified times. Thereafter, aliquots (10 µL) were withdrawn from the reaction vial followed by work up and GC analysis following the general procedure listed in **section 2.1**.

Discussion: In a polar solvent (such as DMA) there was a significantly faster rate of NHP ester conversion (complete consumption within 10 minutes for 1°, 2°, and strained ring 3° NHP esters) to form the alkyl dimer as a major product with minor amounts of alkyl-H and TMS-ester of the acid. This led to a major amount of returned aryl bromide. Conclusion: too-fast alkyl radical formation is responsible for lower yields with electron-rich aryl bromides in DMA.

For reactions in toluene, the conversion of NHP ester slowed down significantly for all three (1°, 2°, and strained ring 3°) NHP esters and this matched well with the slower oxidative addition of 4-bromo anisole, thus giving improved product yield and selectivity. The outcomes of condition-A versus condition-B or condition-C (from **section 3.8**) tested on 4-bromo anisole versus 1°, 2°, and strained ring 3° NHP esters are listed below.

Reaction outcome for the XEC between 4-bromoanisole versus 1°, 2° and strained ring 3° NHP esters in DMA, at 10 min

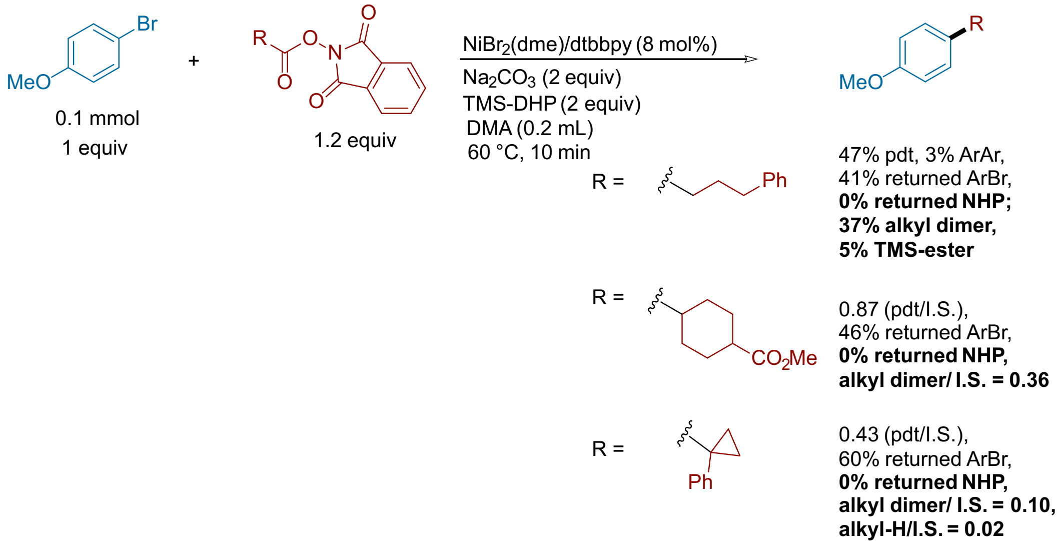

Time points for the XEC between 4-bromoanisole versus 1°, 2° and strained ring 3° NHP esters in toluene

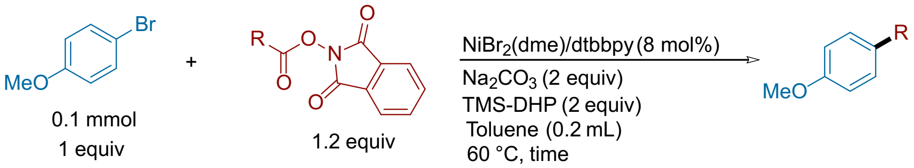

R	*at 10 min*	*at 60 min*	*at 6 h*
R = 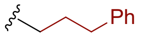	33% pdt, 1% ArAr, 69% returned ArBr, **83% returned NHP, 4% alkyl dimer**	51% pdt, 1% ArAr, 48% returned ArBr, **15% returned NHP, 21% alkyl dimer**	65% pdt, 18% ArAr, 0% returned ArBr, **0% returned NHP, 21% alkyl dimer**
R = 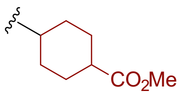	0.71 (pdt/I.S.)[a], 59% returned ArBr, **75% returned NHP, 0% alkyl dimer**	1.60 (pdt/I.S.)[a], 6% ArAr, 0% returned ArBr, **0% returned NHP, 0% alkyl dimer**	
R = 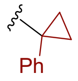	0.24 (pdt/I.S.), 74% returned ArBr, **60% returned NHP, alkyl dimer/I.S. = 0.04**	0.60 (pdt/I.S.), 27% returned ArBr, **13% returned NHP, alkyl dimer/I.S. = 0.04**	0.63 (pdt/I.S.), 4% ArAr, 15% returned ArBr, **0% returned NHP**

*[a]Reactions with the 2° NHP ester in toluene were performed in the presence of TMS-Me$_4$DHP reductant for a direct comparison between the general conditions in DMA (using TMS-DHP reductant) versus the conditions in toluene under which we isolated the product **3ah** in 50% yield.*

6. Product Characterization

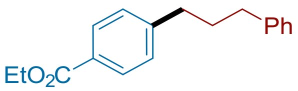

Ethyl-4-(3-phenylpropyl)benzoate (3a)

General Procedure 2.2 was followed using ethyl-4-bromobenzoate (32.6 µL, 45.8 mg, 0.2 mmol, 1 equiv), 1,3-dioxoisoindolin-2-yl-4-phenylbutanoate (74.2 mg, 0.24 mmol, 1.2 equiv), Na_2CO_3 (42.2 mg, 0.4 mmol, 2 equiv) as the additive, and TMS-DHP (90.6 mg, 0.4 mmol, 2 equiv) as the reductant in DMA (0.4 mL) for 4 h at 30 °C. **3a** (45.4 mg, 0.17 mmol, 85%) was isolated as a colorless oil following column chromatography (gradient from 100% hexanes to 8% EtOAc/hexanes). **^{1}H NMR** (500 MHz, $CDCl_3$) δ 7.93 (AA'BB', $J_{AB+AB'}$ = 10.1 Hz, 2H), 7.26–7.23 (m, 4H), 7.16–7.13 (m, 3H), 4.32 (q, J = 7.0 Hz, 2H), 2.66 (t, J = 7.6 Hz, 2H), 2.61 (t, J = 7.4 Hz, 2H), 1.97–1.90 (m, 2H), 1.35 (t, J = 7.1 Hz, 3H). **^{13}C{^{1}H} NMR** (126 MHz, $CDCl_3$) δ 166.8, 147.8, 142.0, 129.8, 128.54, 128.48, 128.3, 126.0, 60.9, 35.5, 35.4, 32.7, 14.5. Characterization data matched those reported in the literature.[30]

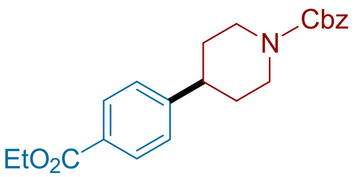

Benzyl-4-(4-(ethoxycarbonyl)phenyl)piperidine-1-carboxylate (3b)

General Procedure 2.2 was followed using ethyl-4-bromobenzoate (32.6 µL, 45.8 mg, 0.2 mmol, 1 equiv), 1-benzyl-4-(1,3-dioxoisoindolin-2-yl)piperidine-1,4-dicarboxylate (98.0 mg, 0.24 mmol, 1.2 equiv), Na_2CO_3 (42.2 mg, 0.4 mmol, 2 equiv) as the additive, and TMS-DHP (90.6 mg, 0.4 mmol, 2 equiv) as the reductant in DMA (0.4 mL) for 4 h at 30 °C. **3b** (50.1 mg, 0.14 mmol, 67%) was isolated as a colorless oil following column chromatography (gradient from 100% hexanes to 20% EtOAc/hexanes).
^{1}H NMR (500 MHz, $CDCl_3$) δ 7.99 (AA'BB', $J_{AB+AB'}$ = 8.4 Hz, 2H), 7.40–7.30 (m, 5H), 7.25 (AA'BB', $J_{AB+AB'}$ = 8.4 Hz, 2H), 5.16 (s, 2H), 4.42–4.26 (m, 4H), 2.89 (br s, 2H), 2.73 (tt, J = 15 Hz, J = 5.0 Hz, 1H), 1.86–1.77 (m, 2H), 1.69–1.62 (m, 2H), 1.38 (t, J = 7.5 Hz, 3H). **^{13}C{^{1}H} NMR (126 MHz, $CDCl_3$)** δ 166.8, 155.4, 150.7, 136.9, 130.0, 128.9, 128.6, 128.1, 128.0, 126.9, 67.2, 60.9, 44.6, 42.8, 32.9 (br s), 14.4. **HRMS (ESI-MS)** m/z: $[M+H]^+$ calcd for $C_{22}H_{26}NO_4^+$, 368.1856, found 368.1853.

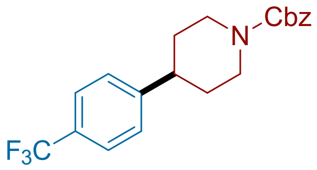

Benzyl-4-(4-(trifluoromethyl)phenyl)piperidine-1-carboxylate (3c)

General Procedure 2.2 was followed using 4-trifluoromethylphenylbromide (14.0 µL, 22.5 mg, 0.1 mmol, 1 equiv), 1-benzyl-4-(1,3-dioxoisoindolin-2-yl)piperidine-1,4-dicarboxylate (49.0 mg, 0.12 mmol, 1.2 equiv), Na_2CO_3 (21.2 mg, 0.2 mmol, 2 equiv) as the additive, and TMS-DHP (45.3 mg, 0.2 mmol, 2 equiv) as the reductant in DMA (0.2 mL) for 4 h at 30 °C. **3c** (28.7 mg, 0.08 mmol, 79%) was isolated as a colorless oil following column chromatography (gradient from 100% hexanes to 20% EtOAc/hexanes).
^{1}H NMR (500 MHz, $CDCl_3$) δ 7.57 (AA'BB', $J_{AB+AB'}$ = 8.4 Hz, 2H), 7.40–7.32 (m, 5H), 7.31 (AA'BB', $J_{AB+AB'}$ = 8.4 Hz, 2H), 5.17 (s, 2H), 4.36 (br s, 2H), 2.93–2.87 (m, 2H), 2.74 (tt, J = 15 Hz, J = 5H, 1H), 1.86–1.84 (m, 2H), 1.70–1.62 (m, 2H). **^{13}C{^{1}H} NMR** (126 MHz, $CDCl_3$) δ 155.4, 149.6, 136.9, 134.4, 128.9 (q, $J_{C\text{-}F}$ = 32.8 Hz), 128.6, 128.2, 127.3, 125.6 (q, $J_{C\text{-}F}$ = 3.75 Hz), 124.3 (q, $J_{C\text{-}F}$ = 272.2 Hz), 67.3, 44.6, 42.7, 33.0. **^{19}F{^{1}H} NMR** (377 MHz, $CDCl_3$) δ –62.36. Characterization data matched those reported in the literature.[31]

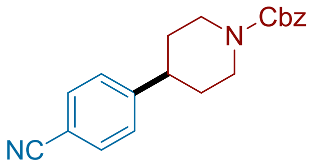

Benzyl-4-(4-cyanophenyl)piperidine-1-carboxylate (3d)

General Procedure 2.2 was followed using 4-bromobenzenecarbonitrile (18.0 mg, 0.1 mmol, 1 equiv), 1-benzyl-4-(1,3-dioxoisoindolin-2-yl)piperidine-1,4-dicarboxylate (49.0 mg, 0.12 mmol, 1.2 equiv), Na_2CO_3 (21.2 mg, 0.2 mmol, 2 equiv) as the additive, and TMS-DHP (45.3 mg, 0.2 mmol, 2 equiv) as the reductant in DMA (0.2 mL) for 4 h at 30 °C. **3d** (25.9 mg, 0.08 mmol, 81%) was isolated as a colorless oil following column chromatography (gradient from 100% hexanes to 20% EtOAc/hexanes).
^{1}H NMR (500 MHz, $CDCl_3$) δ 7.59 (AA'BB', $J_{AB+AB'}$ = 8.2 Hz, 2H), 7.39–7.31 (m, 5H), 7.29 (AA'BB', $J_{AB+AB'}$ = 8.3 Hz, 2H), 5.16 (s, 2H), 4.35 (br s, 2H), 2.91–2.86 (m, 2H), 2.74 (tt, J = 15.0 Hz, J = 5H, 1H), 1.85–1.82 (m, 2H), 1.67–1.59 (m, 2H). **^{13}C{^{1}H} NMR (126 MHz, $CDCl_3$)** δ

155.2, 150.8, 136.8, 132.5, 128.5, 128.1, 128.0, 127.9, 127.6, 118.9, 110.4, 67.2, 44.3, 42.8, 32.6. Characterization data matched those reported in the literature.[32]

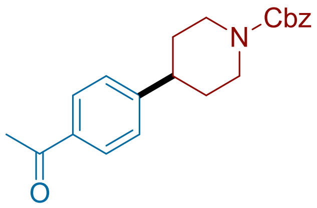

Benzyl-4-(4-acetylphenyl)piperidine-1-carboxylate (3e)

General Procedure 2.2 was followed using 4-bromoacetophenone (19.9 mg, 0.1 mmol, 1 equiv), 1-benzyl-4-(1,3-dioxoisoindolin-2-yl)piperidine-1,4-dicarboxylate (49.0 mg, 0.12 mmol, 1.2 equiv), Na_2CO_3 (21.2 mg, 0.2 mmol, 2 equiv) as the additive, and TMS-DHP (45.3 mg, 0.2 mmol, 2 equiv) as the reductant in DMA (0.2 mL) for 4 h at 30 °C. **3d** (27.7 mg, 0.08 mmol, 82%) was isolated as a colorless oil following column chromatography (gradient from 100% hexanes to 25% EtOAc/hexanes). **^{1}H NMR (500 MHz, $CDCl_3$)** δ 7.90 (AA'BB', $J_{AB+AB'}$, J = 8.4 Hz, 2H), 7.38–7.31 (m, 5H), 7.28 (AA'BB', $J_{AB+AB'}$, J = 8.2 Hz, 2H), 5.16 (s, 2H), 4.35 (br s, 2H), 2.90 (br s, 2H), 2.74 (tt, J = 15 Hz, J = 5H, 1H), 2.58 (s, 3H), 1.86–1.84 (m, 2H), 1.70–1.64 (m, 2H). **^{13}C{^{1}H} NMR (125 MHz, $CDCl_3$)** δ 197.7, 155.3, 151.0, 136.8, 135.6, 128.8, 128.5, 128.1, 128.0, 127.0, 67.2, 44.5, 42.7, 32.8, 26.6. Characterization data matched those reported in the literature.[31]

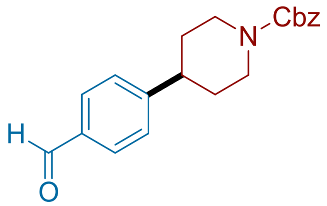

Benzyl-4-(4-formylphenyl)piperidine-1-carboxylate (3f)

General Procedure 2.2 was followed using 4-bromobenzaldehyde (37.0 mg, 0.2 mmol, 1 equiv), 1-benzyl-4-(1,3-dioxoisoindolin-2-yl)piperidine-1,4-dicarboxylate (98.0 mg, 0.24 mmol, 1.2 equiv), Na_2CO_3 (42.2 mg, 0.4 mmol, 2 equiv) as the additive, and TES-DHP (124.2 mg, 0.4 mmol, 2 equiv) as the reductant in DMA (0.4 mL) for 4 h at 40 °C. **3f** (49.2 mg, 0.15 mmol, 76%) was isolated as a colorless oil following reverse phase chromatography (gradient from 5% CH_3CN/H_2O to 85% CH_3CN/H_2O). **^{1}H NMR (500 MHz, $CDCl_3$)** δ 9.98 (s, 1H), 7.84–7.82 (AA'BB', $J_{AB+AB'}$, J = 8.1 Hz, 2H), 7.40–7.35 (m, 6H), 7.34–7.31 (m, 1H), 5.16 (s, 2H), 4.36 (s, 2H), 2.90–2.79 (m, 2H), 2.77 (tt, J = 12.2, 3.6 Hz, 1H), 1.88–1.85 (m, 2H), 1.71–1.63 (m, 2H). **^{13}C{^{1}H} NMR (126 MHz, $CDCl_3$)** δ 191.8, 155.3, 152.6, 136.8, 135.0, 130.2, 128.5, 128.1, 128.0, 127.5, 67.2, 44.4, 42.9, 32.7. **HRMS (ESI-MS)** m/z: $[M+H]^+$ calcd for $C_{20}H_{22}NO_3^+$, 324.1594, found 324.1590. Characterization data matched those reported in the literature.[31]

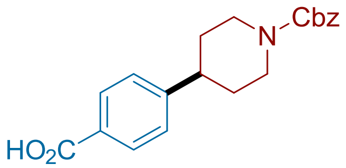

4-(1-((Benzyloxy)carbonyl)piperidin-4-yl)benzoic acid (3g)

General Procedure 2.2 was slightly modified for the synthesis of **3g**. An oven dried 1-dram vial was charged with a PTFE-coated stir bar, 4-bromobenzoic acid (40.2, 0.2 mmol, 1 equiv), *N*,*O*-bis(trimethylsilyl)acetamide (N,O-Bis(trimethylsilyl)acetamide, 97.8 µL, 81.4 mg, 0.4 mmol, 2 equiv) and toluene (0.1 mL). The reaction mixture was stirred at 40 °C for 30 minutes inside the glovebox. Subsequently, 1-benzyl-4-(1,3-dioxoisoindolin-2-yl)piperidine-1,4-dicarboxylate (98.0 mg, 0.24 mmol, 1.2 equiv), Na_2CO_3 (42.4 mg, 0.4 mmol, 2 equiv) and TMS-Me_4DHP (113.0 mg, 0.4 mmol, 2 equiv) were added followed by additional toluene (0.3 mL). The reaction mixture was sealed with a PTFE-faced silicone septa and taken outside the glovebox where it was allowed to stir (1250 RPM) at 80 °C heated for 10 h. **3g** (42.8 mg, 0.13 mmol, 63%) was isolated as a pale brown solid following reverse phase chromatography (water/acetonitrile mixture, gradient of 5% CH_3CN/H_2O-72% CH_3CN in H_2O, both containing 0.1% TFA).
^{1}H NMR (400 MHz, $CDCl_3$) δ 8.06 (d, AA'BB', $J_{AB+AB'}$ = 8.4 Hz, 2H), 7.41–7.32 (m, 5H), 7.30 (AA'BB', $J_{AB+AB'}$ = 8.4 Hz, 2H), 5.18 (s, 2H), 4.37 (br s, 2H), 2.92 (br s, 2H), 2.77 (tt, *J* = 12.1, 3.6 Hz, 1H), 1.89–1.85 (m, 2H), 1.70–1.66 (m, 2H). **^{13}C{^{1}H} NMR (126 MHz, $CDCl_3$)** δ 171.6, 155.4, 151.7, 136.8, 130.6, 128.5, 128.1, 128.0, 127.7, 127.0, 67.3, 44.5, 42.8, 32.8. **HRMS-ESI (m/z)**: $[M+Na]^+$ calcd for $C_{20}H_{21}NO_4Na^+$, 362.1363, found 362.1355.

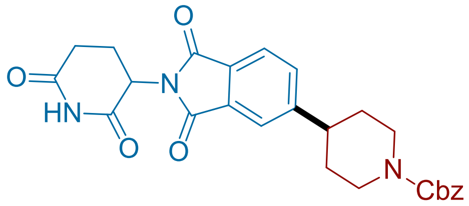

Benzyl 4-(2-(2,6-dioxopiperidin-3-yl)-1,3-dioxoisoindolin-5-yl)piperidine-1-carboxylate (3h)
Safety Note: derivatives of thalidomide and lenalidomide can have serious effects on human health at very low exposure levels. The derivatives should be handled with the same level of caution using suitable safety procedures.

General Procedure 2.2 was followed using 5-bromo-2-(2,6-dioxopiperidin-3-yl)isoindoline-1,3-dione (67.4 mg, 0.2 mmol, 1 equiv), 1-benzyl-4-(1,3-dioxoisoindolin-2-yl)piperidine-1,4-dicarboxylate (98.0 mg, 0.24 mmol, 1.2 equiv), DIPEA (51.7 mg, 69.7 µL, 0.4 mmol, 2 equiv) as the additive and TMS-Me_4DHP (113.0 mg, 0.4 mmol, 2 equiv) as the reductant in DMA (0.4 mL) for 10 h at 80 °C. **3h** (57.1 mg, 0.12 mmol, 60%) was isolated as a pale yellow solid following reverse phase chromatography (water/acetonitrile mixture, gradient of 5% CH_3CN/H_2O–72% CH_3CN in H_2O, both containing 0.1% TFA). **^{1}H NMR (400 MHz, $CDCl_3$)** δ 8.27 (s, 1H), 7.81 (d, *J* = 7.7 Hz, 1H), 7.72 (d, *J* = 1.5 Hz, 1H), 7.57 (dd, *J* = 7.8, 1.5 Hz, 1H), 7.39–7.31 (m, 5H), 5.16 (s, 2H), 4.97 (dd, *J* = 7.1, 5.3 Hz, 1H), 4.37 (brs, 2H), 2.95–2.71 (m, 6H), 2.16–2.11 (m, 1H), 1.87 (d, *J* = 13.0 Hz, 2H), 1.72–1.64 (m, 2H). **^{13}C{^{1}H} NMR (126 MHz, $CDCl_3$)** δ 171.0, 168.1, 167.4, 167.2, 155.3, 153.4, 136.8, 133.2, 132.5, 130.1, 128.7, 128.2, 128.1, 124.2, 122.3, 67.4, 49.5, 44.4, 43.2, 32.9, 31.5, 22.8. **HRMS-ESI (m/z)**: $[M+Na]^+$ calcd for $C_{26}H_{25}N_3O_6Na^+$, 498.1636, found 498.1628.

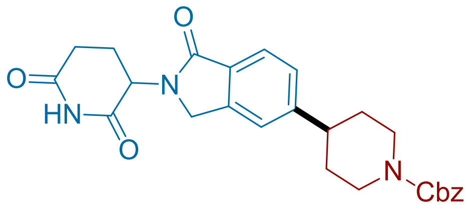

Benzyl-4-(2-(2,6-dioxopiperidin-3-yl)-1-oxoisoindolin-5-yl)piperidine-1-carboxylate (3i)
Safety Note: derivatives of thalidomide and lenalidomide can have serious effects on human health at very low exposure levels. The derivatives should be handled with the same level of caution using suitable safety procedures.

General Procedure 2.2 was followed using 3-(5-bromo-1-oxoisoindolin-2-yl)piperidine-2,6-dione (64.6 mg, 0.2 mmol, 1 equiv), 1-benzyl-4-(1,3-dioxoisoindolin-2-yl)piperidine-1,4-dicarboxylate (98.0 mg, 0.24 mmol, 1.2 equiv), DIPEA (51.7 mg, 69.7 µL, 0.4 mmol, 2 equiv) as the additive and TMS-Me$_4$DHP (113.0 mg, 0.4 mmol, 2 equiv) as the reductant in DMA (0.4 mL) for 10 h at 80 °C. **3h** (87.7 mg, 0.19 mmol, 95%) was isolated as a pale yellow solid following reverse phase chromatography (water/acetonitrile mixture, gradient of 5% CH_3CN/H_2O–100% CH_3CN in H_2O, both containing 0.1% TFA). **^{1}H NMR (500 MHz, DMSO-D$_6$)** δ 10.97 (s, 1H), 7.65 (d, J = 7.8 Hz, 1H), 7.48 (s, 1H), 7.40–7.38 (m, 5H), 7.35–7.31 (m, 1H), 5.12–5.09 (m, 3H), 4.41 (d, J = 17.2 Hz, 1H), 4.29 (d, J = 17.1 Hz, 1H), 4.20–4.06 (m, 2H), 2.95–2.86 (m, 4H), 2.62–2.57 (m, 1H), 2.39 (qd, J = 13.2, 4.5 Hz, 1H), 2.01–1.95 (m, 1H), 1.81–1.78 (m, 2H), 1.58 (qd, J = 12.6, 4.2 Hz, 2H). **^{13}C{^{1}H} NMR (126 MHz, DMSO-D$_6$)** δ 172.8, 171.0, 168.0, 154.4, 149.9, 142.5, 137.0, 129.9, 128.4, 127.8, 127.6, 126.9, 123.0, 121.7, 66.2, 51.5, 47.1, 44.0, 41.8, 32.6, 31.2, 22.5. **HRMS-ESI (m/z)**: $[M+NH_4]^+$ calcd for $C_{26}H_{31}N_4O_5^+$, 479.2289, found 479.2281.

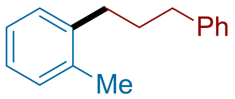

1-Methyl-2-(3-phenylpropyl)benzene (3j)

General Procedure 2.2 was followed using 2-bromotoluene (36.1 µL, 51.3 mg, 0.3 mmol, 1 equiv), 1,3-dioxoisoindolin-2-yl-4-phenylbutanoate (111.4 mg, 0.36 mmol, 1.2 equiv), Na_2CO_3 (63.6 mg, 0.6 mmol, 2 equiv) as the additive, and TMS-DHP (135.9 mg, 0.6 mmol, 2 equiv) as the reductant in 1,4-dioxane (0.6 mL) for 10 h at 70 °C. **3j** (32.2 mg, 0.153 mmol, 51%) was isolated as a colorless oil following column chromatography (100% hexanes). **^{1}H NMR (500 MHz, CDCl$_3$)** δ 7.29–7.26 (m, 2H), 7.22–7.16 (m, 3H), 7.13–7.07 (m, 4H), 2.70 (t, J = 7.8 Hz, 2H), 2.63 (t, J = 7.8 Hz, 2H), 2.26 (s, 3H), 1.94–1.88 (m, 2H). **^{13}C{^{1}H} NMR (126 MHz, CDCl$_3$)** δ 142.3, 140.5, 135.9, 130.1, 128.8, 128.4, 128.3, 125.87, 125.86, 125.7, 35.8, 32.8, 31.7, 19.2. Characterization data matched those reported in the literature.[7]

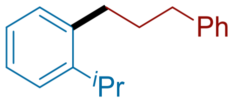

1-Isopropyl-2-(3-phenylpropyl)benzene (3k)

General Procedure 2.2 was followed using 2-bromocumene (45.9 µL, 59.7 mg, 0.3 mmol, 1 equiv), 1,3-dioxoisoindolin-2-yl-4-phenylbutanoate (111.4 mg, 0.36 mmol, 1.2 equiv), Na_2CO_3 (63.6 mg, 0.6 mmol, 2 equiv) as the additive, and TMS-DHP (135.9 mg, 0.6 mmol, 2 equiv) as the reductant in 1,4-dioxane (0.6 mL) for 10 h at 70 °C. **3k** (41.5 mg, 0.17 mmol, 58%) was isolated as a colorless oil following column chromatography (100% hexanes). **^{1}H NMR (500 MHz, CDCl$_3$)** δ 7.29–7.26 (m, 3H), 7.20–7.15 (m, 4H), 7.13–7.09 (m, 2H), 3.09 (septet, J = 6.9 Hz, 1H), 2.69–2.66 (m, 4H), 1.94–1.89 (m, 2H), 1.20 (d, J = 6.9 Hz, 6H). **^{13}C{^{1}H} NMR (126 MHz, CDCl$_3$)** δ 146.6, 142.3, 139.0, 129.3, 128.4, 128.3, 126.3, 125.8, 125.7, 125.5, 35.9, 33.2, 32.4, 28.5, 24.0. Characterization data matched those reported in the literature.[33]

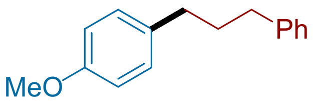

Methoxy-4-(3-phenylpropyl)benzene (3m)

General Procedure 2.2 was followed using 4-bromoanisole (25.1 µL, 37.4 mg, 0.2 mmol, 1 equiv), 1,3-dioxoisoindolin-2-yl-4-phenylbutanoate (74.2 mg, 0.24 mmol, 1.2 equiv), Na_2CO_3 (21.2 mg, 0.2 mmol, 2 equiv) as the additive, and TMS-DHP (45.3 mg, 0.2 mmol, 2 equiv) as the reductant in toluene (0.4 mL) for 10 h at 80 °C. **3m** (31.2 mg, 0.08 mmol, 69%) was isolated as a colorless oil following column chromatography (gradient from 100% hexanes to 10% EtOAc/hexanes). **^{1}H NMR (500 MHz, $CDCl_3$)** δ 7.27 (t, J = 7.5 Hz, 2H), 7.18–7.17 (m, 3H), 7.09 (AA'BB', $J_{AB+AB'}$, J = 8.3 Hz, 2H), 6.82 (AA'BB', $J_{AB+AB'}$, J = 8.3 Hz, 2H), 3.77 (s, 3H), 2.63 (t, J = 7.6 Hz, 2H), 2.59 (t, J = 7.6 Hz, 2H), 1.92 (pentet, J = 7.6 Hz, 2H). **^{13}C{^{1}H} NMR (126 MHz, $CDCl_3$)** δ 157.7, 142.4, 134.4, 129.3, 128.4, 128.3, 125.7, 113.7, 55.2, 35.4, 34.5, 33.2. Characterization data matched those reported in the literature.[34]

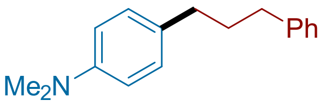

***N*,*N*-dimethyl-4-(3-phenylpropyl)aniline (3n)**

General Procedure 2.2 was followed using 4-bromo-*N*,*N*-dimethylaniline (20.0 mg, 0.1 mmol, 1 equiv), 1,3-dioxoisoindolin-2-yl-4-phenylbutanoate (37.1 mg, 0.12 mmol, 1.2 equiv), Na_2CO_3 (21.2 mg, 0.2 mmol, 2 equiv) as the additive, and TMS-DHP (45.3 mg, 0.2 mmol, 2 equiv) as the reductant in toluene (0.2 mL) for 10 h at 80 °C. **3n** (16.0 mg, 0.067 mmol, 67%) was isolated as a colorless oil following column chromatography (gradient from 100% hexanes to 8% EtOAc/hexanes). **^{1}H NMR (500 MHz, $CDCl_3$)** δ 7.28–7.25 (m, 2H), 7.19–7.15 (m, 3H), 7.06 (AA'BB', $J_{AB+AB'}$, J = 8.6 Hz, 2H), 6.70 (AA'BB', $J_{AB+AB'}$, J = 8.6 Hz, 2H), 2.90 (s, 6H), 2.65 (t, J = 7.5 Hz, 2H), 2.57 (t, J = 7.5 Hz, 2H), 1.95–1.89 (m, 2H). **^{13}C{^{1}H} NMR (125 MHz, $CDCl_3$)** δ 149.0, 142.6, 130.5, 129.0, 128.5, 128.2, 125.6, 113.0, 41.0, 35.4, 34.4, 33.2. Characterization data matched those reported in the literature.[35]

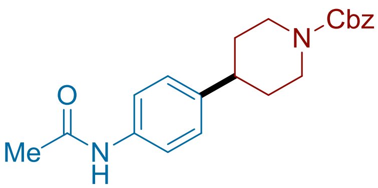

Benzyl-4-(4-acetamidophenyl)piperidine-1-carboxylate (3o)

General Procedure 2.2 was followed using *N*-(4-bromophenyl)acetamide (21.4 mg, 0.1 mmol, 1 equiv), 1-benzyl-4-(1,3-dioxoisoindolin-2-yl)piperidine-1,4-dicarboxylate (49.0 mg, 0.12 mmol, 1.2 equiv), Na_2CO_3 (21.2 mg, 0.2 mmol, 2 equiv) as the additive, and TMS-DHP (45.3 mg, 0.2 mmol, 2 equiv) as the reductant in toluene (0.2 mL) for 10 h at 80 °C. **3o** (23.3 mg, 0.066 mmol, 66%) was isolated as a colorless oil following column chromatography (gradient from 100% hexanes to 65% EtOAc/hexanes). **^{1}H NMR (500 MHz, $CDCl_3$)** δ 7.41 (AA'BB', $J_{AB+AB'}$, J = 8.5 Hz, 2H), 7.37–7.34 (m, 3H), 7.33–7.29 (m, 2H), 7.13 (AA'BB', $J_{AB+AB'}$, J = 8.5 Hz, 2H), 5.15 (s, 2H), 4.31(br s, 2H), 2.87 (br s, 2H), 2.63 (tt, J = 10.0 Hz, J = 5.0 Hz, 1H), 2.15 (s, 3H), 1.80 (d, J = 13.0 Hz, 2H), 1.61–1.56 (m, 3H). **^{13}C{^{1}H} NMR (125 MHz, $CDCl_3$)** δ 168.4, 155.4, 141.7, 137.0, 136.3, 128.6, 128.1, 128.0, 127.3, 120.4, 67.2, 44.7, 42.1, 33.2, 24.6. **HRMS-ESI (m/z)**: $[M+Na]^+$ calcd for $C_{21}H_{24}N_2O_3Na^+$, 375.1679, found 375.1671.

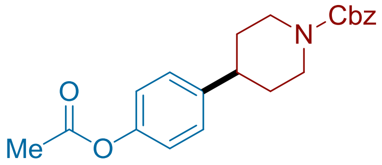

Benzyl-4-(4-acetoxyphenyl)piperidine-1-carboxylate (3p)

General Procedure 2.2 was followed using 4-bromophenyl acetate (43.0 mg, 0.2 mmol, 1 equiv), 1-benzyl-4-(1,3-dioxoisoindolin-2-yl)piperidine-1,4-dicarboxylate (98.0 mg, 0.24 mmol, 1.2 equiv), Na_2CO_3 (42.4 mg, 0.4 mmol, 2 equiv) as the additive, and TMS-DHP (90.6 mg, 0.4 mmol, 2 equiv) as the reductant in toluene (0.4 mL) for 10 h at 80 °C. **3p** (57.2 mg, 0.16 mmol, 81%) was isolated as a white solid following reverse phase chromatography (water/acetonitrile mixture, gradient of 5% CH_3CN/H_2O–85% CH_3CN in H_2O). **^{1}H NMR (500 MHz, $CDCl_3$)** δ 7.43–7.37 (m, 4H), 7.37–7.34 (m, 1H), 7.23 (AA'BB', $J_{AB+AB'}$ = 7.5 Hz, 2H), 7.05 (d, AA'BB', $J_{AB+AB'}$ = 7.5 Hz, 2H), 5.19 (s, 2H), 4.36 (br s, 2H), 2.91 (br s, 2H), 2.71 (tt, J = 12.2, 3.6 Hz, 1H), 2.31 (s, 3H), 1.88–1.85 (m, 2H), 1.69–1.64 (m, 2H). **^{13}C{^{1}H} NMR (125 MHz, $CDCl_3$)** δ 168.5, 154.2, 148.0, 141.9, 135.8, 127.4, 126.9, 126.8, 126.6, 120.4, 66.0, 43.5, 40.9, 32.0, 20.1. **HRMS-ESI (m/z)**: $[M+H]^+$ calcd for $C_{21}H_{24}NO_4^+$, 354.1700, found 354.1696.

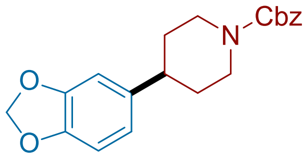

Benzyl-4-(benzo[d][1,3]dioxol-5-yl)piperidine-1-carboxylate (3q)

General Procedure 2.2 was followed using 1,2-(methylenedioxy)-4-bromobenzene (12.1 µL, 20.1 mg, 0.1 mmol, 1 equiv), 1-benzyl-4-(1,3-dioxoisoindolin-2-yl)piperidine-1,4-dicarboxylate (49.0 mg, 0.12 mmol, 1.2 equiv), Na_2CO_3 (21.2 mg, 0.2 mmol, 2 equiv) as the additive, and TMS-DHP (45.3 mg, 0.2 mmol, 2 equiv) as the reductant in toluene (0.2 mL) for 10 h at 80 °C. **3q** (20.7 mg, 0.061 mmol, 61%) was isolated as a colorless oil following column chromatography (gradient from 100% hexanes to 25% EtOAc/hexanes). **^{1}H NMR (500 MHz, $CDCl_3$)** δ 7.39–7.30 (m, 5H), 6.74 (d, J = 7.9 Hz, 1H), 6.68 (d, J = 1.7 Hz, 1H), 6.64 (dd, J = 8.0, 1.8 Hz, 1H), 5.92 (s, 2H), 5.15 (s, 2H), 4.31 (s, 2H), 2.85 (s, 2H), 2.59 (tt, J = 12.1, 3.6 Hz, 1H), 1.80 (d, J = 12.9 Hz, 2H), 1.57 (s, 2H). **^{13}C{^{1}H} NMR (125 MHz, $CDCl_3$)** δ 155.3, 147.7, 146.0, 139.7, 137.0, 128.5, 128.0, 127.9, 119.5, 108.3, 107.2, 100.9, 67.1, 44.6, 42.4, 33.4. **HRMS-ESI (m/z)**: $[M+H]^+$ calcd for $C_{20}H_{22}NO_4^+$, 340.1543, found 340.1539.

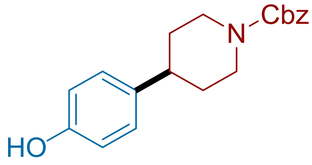

Benzyl-4-(4-hydroxyphenyl)piperidine-1-carboxylate (3r)

General Procedure 2.2 was followed using 4-bromophenol (17.3, 0.1 mmol, 1 equiv), 1-benzyl-4-(1,3-dioxoisoindolin-2-yl)piperidine-1,4-dicarboxylate (49.0 mg, 0.12 mmol, 1.2 equiv), Na_2CO_3 (21.2 mg, 0.2 mmol, 2 equiv) as the additive, and TMS-Me_4DHP (56.5 mg, 0.2 mmol, 2 equiv) as the reductant in toluene (0.2 mL) for 10 h at 80 °C. **3r** (30.9 mg, 0.099 mmol, 99%) was isolated as a white solid following reverse phase chromatography (water/acetonitrile mixture,

gradient of 5% CH_3CN/H_2O–72% CH_3CN in H_2O). **^{1}H NMR (500 MHz, $CDCl_3$)** δ 7.39–7.30 (m, 5H), 7.03 (AA'BB', $J_{AB+AB'}$ = 8.5 Hz, 2H), 6.79 (AA'BB', $J_{AB+AB'}$ = 8.5 Hz, 2H), 6.10 (br s, 1H), 5.17 (s, 2H), 4.31 (br s, 2H), 2.91–2.85 (m, 2H), 2.60 (tt, J = 12.2, 3.6 Hz, 1H), 1.81 (d, J = 13.1 Hz, 2H), 1.58 (qd, J = 12.8, 4.3 Hz, 2H). **^{13}C{^{1}H} NMR (126 MHz, $CDCl_3$)** δ 155.6, 154.6, 137.5, 136.8, 128.6, 128.2, 128.0, 127.8, 115.5, 67.4, 44.8, 41.8, 33.5. Characterization data matched those reported in the literature.[36]

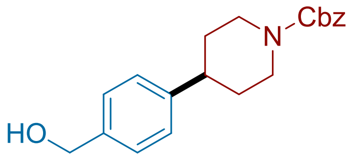

Benzyl-4-(4-(hydroxymethyl)phenyl)piperidine-1-carboxylate (3s)

General Procedure 2.2 was followed using 4-bromobenzyl alcohol (37.4, 0.2 mmol, 1 equiv), 1-benzyl-4-(1,3-dioxoisoindolin-2-yl)piperidine-1,4-dicarboxylate (98.0 mg, 0.24 mmol, 1.2 equiv), Na_2CO_3 (42.4 mg, 0.4 mmol, 2 equiv) as the additive, and TMS-Me$_4$DHP (113.0 mg, 0.4 mmol, 2 equiv) as the reductant in toluene (0.4 mL) for 10 h at 80 °C. **3s** (39.0 mg, 0.12 mmol, 60%) was isolated as a white solid following reverse phase chromatography (water/acetonitrile mixture, gradient of 5% CH_3CN/H_2O–62% CH_3CN in H_2O). **^{1}H NMR (500 MHz, $CDCl_3$)** δ 7.42–7.35 (m, 4H), 7.34–7.31 (m, 3H), 7.19 (AA'BB', $J_{AB+AB'}$= 8.2 Hz, 2H), 5.15 (s, 2H), 4.66 (s, 2H), 4.32 (br s, 2H), 2.88 (br s, 2H), 2.67 (tt, J = 12.2, 3.6 Hz, 1H), 2.08 (br s, 1H), 1.83 (d, J = 12.8 Hz, 2H), 1.63 (qd, J = 12.8, 4.3 Hz, 2H). **^{13}C{^{1}H} NMR (126 MHz, $CDCl_3$)** δ 155.4, 145.1, 139.3, 136.9, 128.6, 128.1, 128.0, 127.4, 127.0, 67.2, 65.1, 44.7, 42.4, 33.2. Characterization data matched those reported in the literature.[31]

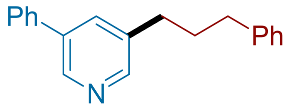

3-Phenyl-5-(3-phenylpropyl)pyridine (3t)

General Procedure 2.2 was followed using 3-bromo-5-phenylpyridine (70.2 mg, 0.3 mmol, 1 equiv), 1,3-dioxoisoindolin-2-yl-4-phenylbutanoate (111.4 mg, 0.36 mmol, 1.2 equiv), Na_2CO_3 (63.6 mg, 0.6 mmol, 2 equiv) as the additive, and TMS-DHP (135.9 mg, 0.6 mmol, 2 equiv) as the reductant in DMA (0.6 mL) for 10 h at 80 °C. **3t** (73.4 mg, 0.27 mmol, 89% yield from ^{1}H NMR) was isolated as a colorless oil following column chromatography (gradient from 100% hexanes to 30% EtOAc/hexanes; the product contained 3% aryl-dimer that was inseparable). **^{1}H NMR (500 MHz, $CDCl_3$)** δ 8.71 (d, J = 2.2 Hz, 1H), 8.46 (d, J = 2.3 Hz, 1H), 7.69 (t, J = 2.2 Hz, 1H), 7.61–7.58 (m, 2H), 7.51–7.46 (m, 2H), 7.43–7.39 (m, 1H), 7.33–7.29 (m, 2H), 7.23–7.20 (m, 3H), 2.75–2.70 (m, 4H), 2.07–2.01 (m, 2H). **^{13}C{^{1}H} NMR (126 MHz, $CDCl_3$)** δ 148.8, 146.0, 141.7, 138.1, 137.4, 136.4, 134.4, 129.15, 129.10, 128.5, 128.1, 127.3, 126.0, 35.4, 32.7, 32.5. **HRMS-ESI (m/z)**: $[M+H]^+$ calcd for $C_{20}H_{20}N^+$, 274.1590, found 274.1589.

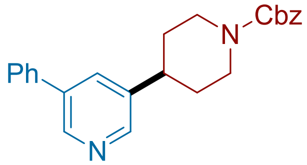

Benzyl-4-(5-phenylpyridin-3-yl)piperidine-1-carboxylate (3u)

General Procedure 2.2 was followed using 3-bromo-5-phenylpyridine (46.8 mg, 0.2 mmol, 1 equiv), 1-benzyl-4-(1,3-dioxoisoindolin-2-yl)piperidine-1,4-dicarboxylate (98.0 mg, 0.24 mmol, 1.2 equiv), Na_2CO_3 (42.4 mg, 0.4 mmol, 2 equiv) as the additive, and TMS-DHP (90.6 mg, 0.4 mmol, 2 equiv) as the reductant in toluene (0.4 mL) for 10 h at 80 °C. **3u** (56.6 mg, 0.15 mmol, 76% yield from ^{1}H NMR) was isolated as a white solid following reverse phase chromatography (water/acetonitrile mixture, gradient of 5% CH_3CN/H_2O–80% CH_3CN in H_2O; the product contained 5% aryl dimer that was inseparable). **^{1}H NMR (500 MHz, CDCl$_3$)** δ 8.70 (d, J = 2.2 Hz, 1H), 8.46 (d, J = 2.2 Hz, 1H), 7.67 (t, J = 2.2 Hz, 1H), 7.57 – 7.54 (m, 2H), 7.49–7.46 (m, 2H), 7.42–7.40 (m, 1H), 7.39–7.30 (m, 5H), 5.17 (s, 2H), 4.38 (s, 2H), 2.93 (s, 2H), 2.79 (tt, J = 12.2, 3.6 Hz, 1H), 1.90 (d, J = 12.8 Hz, 2H), 1.76–1.68 (m, 2H). **^{13}C{^{1}H} NMR (126 MHz, CDCl$_3$)** δ 155.4, 147.6, 146.7, 140.6, 138.0, 136.9, 132.7, 129.2, 128.6, 128.25, 128.16, 128.08, 127.3, 67.3, 44.6, 40.3, 33.0. **HRMS-ESI (m/z)**: $[M+H]^+$ calcd for $C_{24}H_{25}N_2O_2^+$, 373.1910, found 373.1903.

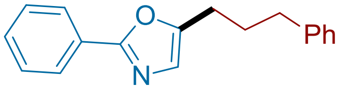

2-Phenyl-5-(3-phenylpropyl)oxazole (3v)

General Procedure 2.2 was followed using 5-bromo-2-phenyloxazole (22.4 mg, 0.1 mmol, 1 equiv), 1,3-dioxoisoindolin-2-yl-4-phenylbutanoate (37.1 mg, 0.12 mmol, 1.2 equiv), Na_2CO_3 (21.2 mg, 0.2 mmol, 2 equiv) as the additive, and TMS-DHP (45.3 mg, 0.2 mmol, 2 equiv) as the reductant in DMA (0.2 mL) for 10 h at 80 °C. **3v** (19.8 mg, 0.075 mmol, 75%) was isolated as a colorless oil following column chromatography (gradient from 100% hexanes to 20% EtOAc/hexanes). **^{1}H NMR (500 MHz, CDCl$_3$)** δ 7.98–7.96 (m, 2H), 7.42–7.36 (m, 3H), 7.28–7.25 (m, 2H), 7.18–7.15 (m, 3H), 6.82 (s, 1H), 2.73–2.67 (m, 4H), 2.01 (p, J = 7.5 Hz, 2H). **^{13}C{^{1}H} NMR (125 MHz, CDCl$_3$)** δ 160.9, 152.8, 141.6, 130.1, 128.9, 128.62, 128.59, 127.9, 126.2, 126.1, 124.0, 35.3, 29.4, 25.2. Characterization data matched those reported in the literature.[37]

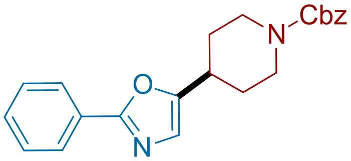

Benzyl 4-(2-phenyloxazol-5-yl)piperidine-1-carboxylate (3w)

General Procedure 2.2 was followed using 5-bromo-2-phenyloxazole (44.8 mg, 0.2 mmol, 1 equiv), 1-benzyl-4-(1,3-dioxoisoindolin-2-yl)piperidine-1,4-dicarboxylate (98.0 mg, 0.24 mmol, 1.2 equiv), Na_2CO_3 (42.4 mg, 0.4 mmol, 2 equiv) as the additive, and TMS-Me$_4$DHP (113.0 mg, 0.4 mmol, 2 equiv) as the reductant in toluene (0.4 mL) for 10 h at 80 °C. **3w** (39.9 mg, 0.11 mmol, 55%) was isolated as a white solid following reverse phase chromatography (water/acetonitrile mixture, gradient of 5% CH_3CN/H_2O – 82% CH_3CN in H_2O). **^{1}H NMR (500 MHz, CDCl$_3$)** δ 8.04–8.02 (m, 2H), 7.48–7.46 (m, 3H), 7.42–7.34 (m, 5H), 6.88 (br s, 1H), 5.19 (s, 2H), 4.28 (br s, 2H), 3.05–2.94 (m, 3H), 2.10–2.07 (m, 2H), 1.77–1.68 (m, 2H). **^{13}C{^{1}H} NMR (125 MHz, CDCl$_3$)** δ 159.7, 154.12, 154.07, 135.7, 129.0, 127.6, 127.4, 126.9, 126.8, 126.5, 124.9, 121.5, 66.1, 42.4, 32.4, 28.9. **HRMS-ESI (m/z)**: $[M+H]^+$ calcd for $C_{22}H_{23}N_2O_3^+$, 363.1703, found 363.1696.

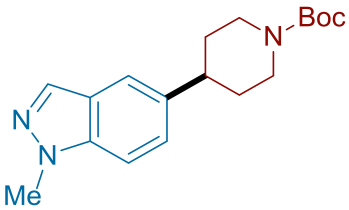

***tert*-Butyl 4-(1-methyl-1H-indazol-5-yl)piperidine-1-carboxylate** (**3x**)

General Procedure 2.2 was followed using 5-bromo-1-methyl-1H-indazole (42.2 mg, 0.2 mmol, 1 equiv), 1-benzyl-4-(1,3-dioxoisoindolin-2-yl)piperidine-1,4-dicarboxylate (98.0 mg, 0.24 mmol, 1.2 equiv), Na_2CO_3 (42.4 mg, 0.4 mmol, 2 equiv) as the additive, and TMS-Me_4DHP (113.0 mg, 0.4 mmol, 2 equiv) as the reductant in toluene (0.4 mL) for 10 h at 80 °C. **3x** (31.5 mg, 0.10 mmol, 50%) was isolated as a pale yellow oil following reverse phase chromatography (water/acetonitrile mixture, gradient of 5% CH_3CN/H_2O–70% CH_3CN in H_2O). **^{1}H NMR (500 MHz, $CDCl_3$)** δ 7.92 (d, J = 1.0 Hz, 1H), 7.64 (d, J = 8.3 Hz, 1H), 7.18 (s, 1H), 7.02 (dd, J = 8.4, 1.4 Hz, 1H), 4.28 (br s, 2H), 4.05 (s, 3H), 2.86–2.77 (m, 3H), 1.91–1.88 (m, 2H), 1.71 (qd, J = 12.5, 4.3 Hz, 2H), 1.49 (s, 9H). **^{13}C{^{1}H} NMR (126 MHz, $CDCl_3$)** δ 153.8, 143.5, 139.2, 131.4, 121.7, 119.9, 119.5, 105.0, 78.4, 43.4, 42.1, 34.2, 32.3, 27.4. **HRMS-ESI (m/z)**: [M+H]$^+$ calcd for $C_{18}H_{26}N_3O_2$, 316.2019, found 316.2019.

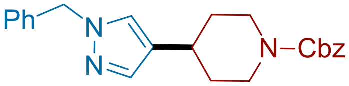

Benzyl 4-(1-benzyl-1H-pyrazol-4-yl)piperidine-1-carboxylate (**3y**)

General Procedure 2.2 was followed using 1-benzyl-4-bromo-1H-pyrazole (59.3 mg, 0.25 mmol, 1 equiv), 1-benzyl-4-(1,3-dioxoisoindolin-2-yl)piperidine-1,4-dicarboxylate (126.7 mg, 0.30 mmol, 1.2 equiv), DIPEA (87.1 μL, 64.6 mg, 0.50 mmol, 2 equiv) as the additive, and TMS-Me_4DHP (141.3 mg, 0.50 mmol, 2 equiv) as the reductant in toluene (0.5 mL) for 10 h at 80 °C. **3y** (37.5 mg, 0.10 mmol, 40% yield from ^{1}H NMR) was isolated as a pale yellow solid following reverse phase chromatography (water/acetonitrile mixture, gradient of 5% CH_3CN/H_2O–76% CH_3CN in H_2O; the product contained 11% alkyl-H (benzyl piperidine-1-carboxylate) that was inseparable. **^{1}H NMR (500 MHz, $CDCl_3$)** δ 7.38 (s, 1H), 7.36–7.29 (m, 9H), 7.19 (s, 1H), 7.15 (s, 1H), 5.25 (s, 2H), 5.13 (s, 2H), 4.20 (br s, 2H), 3.44 (t, J = 5.4 Hz, 1H), 2.90–2.85 (m, 2H), 2.64 (tt, J = 11.7, 3.7 Hz, 1H), 1.89–1.83 (m, 2H), 1.60–1.57 (m, 1H). **^{13}C{^{1}H} NMR (126 MHz, $CDCl_3$)** δ 154.2, 136.3, 135.8, 135.6, 127.7, 127.4, 126.92, 126.86, 126.77, 126.70, 126.6, 125.0, 65.9, 54.9, 43.1, 31.2, 23.3. **HRMS-ESI (m/z)**: [M+H]$^+$ calcd for $C_{23}H_{26}N_3O_2^+$, 376.2020, found 376.2017.

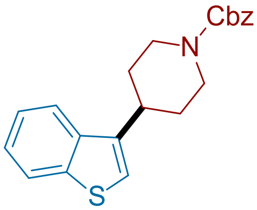

Benzyl 4-(benzo[b]thiophen-3-yl)piperidine-1-carboxylate (**3z**)

General Procedure 2.2 was followed using 3-bromothianaphthene (26.2 μL, 42.6 mg, 0.2 mmol, 1 equiv), 1-benzyl 4-(1,3-dioxoisoindolin-2-yl)piperidine-1,4-dicarboxylate (98.0 mg, 0.24 mmol, 1.2 equiv), Na_2CO_3 (42.4 mg, 0.4 mmol, 2 equiv) as the additive, and TMS-Me_4DHP (113.0

mg, 0.4 mmol, 2 equiv) as the reductant in toluene (0.4 mL) for 10 h at 80 °C. **3z** (53.4 mg, 0.152 mmol, 76%) was isolated as a light pink oil following column chromatography (gradient from 100% hexanes to 35% EtOAc/hexanes). **^{1}H NMR (500 MHz, CDCl$_3$)** δ 7.88 (d, *J* = 7.6, 1H), 7.78 (d, *J* = 7.7, 1H), 7.42 – 7.32 (m, 7H), 7.09 (s, 1H), 5.19 (s, 2H), 4.38 (br s, 2H), 3.11 (tt, *J* = 12.1, 3.5 Hz, 1H), 3.05–2.98 (m, 2H), 2.09 – 2.05 (m, 2H), 1.76–1.69 (m, 2H). **^{13}C{^{1}H} NMR (126 MHz, CDCl$_3$)** δ 155.4, 140.8, 140.4, 138.2, 137.0, 128.6, 128.1, 128.0, 124.4, 124.0, 123.2, 121.6, 120.2, 67.2, 44.7, 36.2, 32.3. **HRMS-ESI (m/z)**: [M+H]$^+$ calcd for $C_{21}H_{22}NO_2S^+$, 352.1366, found 352.1363.

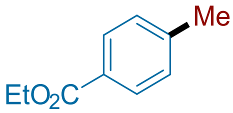

Ethyl 4-methylbenzoate (3aa)

General Procedure 2.2 was followed using 1-bpp (8 mol%) as the ligand instead of dtbbpy, ethyl-4-bromobenzoate (49 µL, 68.7 mg, 0.3 mmol, 1 equiv), 1,3-dioxoisoindolin-2-yl acetate (92.3 mg, 0.45 mmol, 1.5 equiv), Na_2CO_3 (63.6 mg, 0.6 mmol, 2 equiv) as the additive, and TMS-DHP (135.9 mg, 0.6 mmol, 2 equiv) as the reductant in DMA (0.6 mL) for 10 h at 80 °C. **3aa** (8.6 mg, 0.057 mmol, 19% yield) was isolated as a colorless oil following column chromatography (100% hexanes). Measured crude ^{1}H NMR yield of 51% using 1,3,5-trimethoxybenzene as internal standard. The discrepancy between isolated and NMR yield is due to the volatile nature of the product (bp 235 °C). **^{1}H NMR (500 MHz, CDCl$_3$)** δ 7.86 (AA'BB', $J_{AB+AB'}$, *J* = 8.5 Hz, 2H), 7.17 (AA'BB', $J_{AB+AB'}$, *J* = 8.5 Hz, 2H), 4.29 (q, *J* = 7.2 Hz, 2H), 2.34 (s, 3H), 1.32 (t, *J* = 7.1 Hz, 3H). **^{13}C{^{1}H} NMR (126 MHz, CDCl$_3$)** δ 165.7, 142.4, 128.5, 128.0, 126.8, 59.7, 20.6, 13.3. Characterization data matched those reported in the literature.[38]

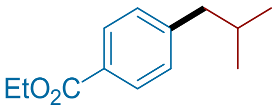

Ethyl 4-isobutylbenzoate (3ab)

General Procedure 2.2 was followed using ethyl-4-bromobenzoate (49 µL, 68.7 mg, 0.3 mmol, 1 equiv), 1,3-dioxoisoindolin-2-yl-3-methylbutanoate (89.0 mg, 0.36 mmol, 1.2 equiv), Na_2CO_3 (63.6 mg, 0.6 mmol, 2 equiv) as the additive, and TMS-DHP (135.9 mg, 0.6 mmol, 2 equiv) as the reductant in DMA (0.6 mL) for 10 h at 80 °C. **3ab** (46.4 mg, 0.225 mmol, 75%) was isolated as a colorless oil following column chromatography (100% hexanes). **^{1}H NMR (500 MHz, CDCl$_3$)** δ 7.95 (AA'BB', $J_{AB+AB'}$, *J* = 8.5 Hz, 2H), 7.20 (AA'BB', $J_{AB+AB'}$, *J* = 8.5 Hz, 2H), 4.36 (q, *J* = 7.1 Hz, 2H), 2.52 (d, *J* = 7.2 Hz, 2H), 1.89 (septet, *J* = 6.7 Hz, 1H), 1.38 (t, *J* = 7.1 Hz, 3H), 0.90 (d, *J* = 6.6 Hz, 6H). **^{13}C{^{1}H} NMR (126 MHz, CDCl$_3$)** δ 166.9, 147.3, 129.6, 129.2, 128.2, 60.9, 45.6, 30.3, 22.5, 14.5. Characterization data matched those reported in the literature.[39]

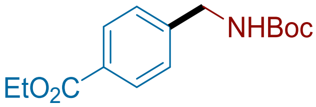

Ethyl 4-(((tert-butoxycarbonyl)amino)methyl)benzoate (3ac)

General Procedure 2.2 was followed using ethyl-4-bromobenzoate (49 µL, 68.7 mg, 0.3 mmol, 1 equiv), 1,3-dioxoisoindolin-2-yl (tert-butoxycarbonyl)glycinate (115.3 mg, 0.36 mmol, 1.2 equiv), Na_2CO_3 (63.6 mg, 0.6 mmol, 2 equiv) as the additive, and TMS-DHP (135.9 mg, 0.6

mmol, 2 equiv) as the reductant in DMA (0.6 mL) for 10 h at 80 °C. **3ac** (67.0 mg, 0.24 mmol, 79%) was isolated as a colorless oil following column chromatography (gradient from 100% hexanes to 40% EtOAc/hexanes). **^{1}H NMR (500 MHz, CDCl$_3$)** δ 7.97 (AA'BB', $J_{AB+AB'}$, J = 8.3 Hz, 2H), 7.32 (AA'BB', $J_{AB+AB'}$, J = 8.3 Hz, 2H), 5.03 (br s, 1H), 4.37– 4.32 (m, 4H), 1.44 (s, 9H), 1.37 (t, J = 7.1 Hz, 3H). **^{13}C{^{1}H} NMR (126 MHz, CDCl$_3$)** δ 166.5, 156.0, 144.3, 129.9, 129.6, 127.2, 79.8, 61.0, 44.4, 28.5, 14.4. Characterization data matched those reported in the literature.[40]

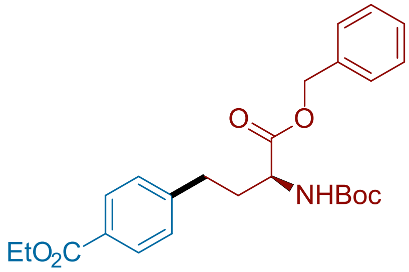

Ethyl (S)-4-(4-(benzyloxy)-3-((tert-butoxycarbonyl)amino)-4-oxobutyl)benzoate (3ad)

General Procedure 2.2 was followed using ethyl-4-bromobenzoate (16.3 µL, 22.9 mg, 0.1 mmol, 1 equiv), 1-benzyl-5-(1,3-dioxoisoindolin-2-yl) (tert-butoxycarbonyl)-L-glutamate (57.9 mg, 0.12 mmol, 1.2 equiv), Na_2CO_3 (21.2 mg, 0.2 mmol, 2 equiv) as the additive, and TMS-DHP (45.3 mg, 0.2 mmol, 2 equiv) as the reductant in DMA (0.2 mL) for 10 h at 80 °C. **3ad** (27.8 mg, 0.063 mmol, 63%) was isolated as an off-white solid following column chromatography, having >98% ee (gradient from 100% hexanes to 25% EtOAc/hexanes). **^{1}H NMR (500 MHz, CDCl$_3$)** δ 7.93 (AA'BB', $J_{AB+AB'}$, J = 8.2 Hz, 2H), 7.39–7.32 (m, 5H), 7.16 (AA'BB', $J_{AB+AB'}$, J = 8.2 Hz, 2H), 5.20 (d, J = 12.2 Hz, 1H), 5.13–5.09 (m, 2H), 4.41–4.34 (m, 3H), 2.73–2.60 (m, 2H), 2.18–2.12 (m, 1H), 1.98–1.91 (m, 1H), 1.44 (s, 9H), 1.38 (t, J = 7.1 Hz, 3H). **^{13}C{^{1}H} NMR (126 MHz, CDCl$_3$)** δ 172.3, 166.5, 155.3, 146.1, 135.3, 129.8, 128.7, 128.6, 128.5, 128.42, 128.38, 80.1, 67.2, 60.8, 53.2, 34.1, 31.6, 28.3, 14.3. **HRMS-ESI (m/z)**: $[M+Na]^+$ calcd for $C_{25}H_{31}NO_6Na^+$, 464.2044, found 464.2038. **SFC-MS:** (Chiralpak IC-3 column (3 mm ID x 150 mm), 2200 psi, 40 ºC, 10% MeOH in CO_2 to 40% MeOH in CO_2, v=0.8 mL/min, λ = 254 nm): t_R [min] = 4.483 (99.03%), 5.013 (0.97%), >98% ee.

Upon using the racemic NHP ester, 1-benzyl-5-(1,3-dioxoisoindolin-2-yl) (tert-butoxycarbonyl)glutamate, (±)-**3ad** was isolated in 68% isolated yield.

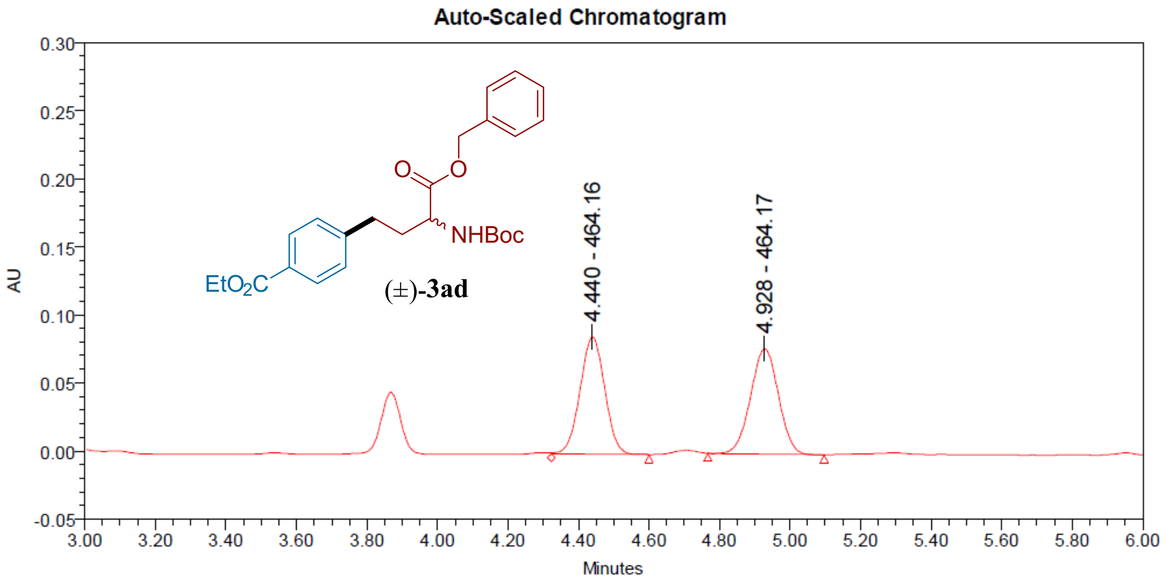

Peak Results

	Name	RT	Area	Height	Amount	Units	% Area
1		4.440	417830	85974			49.53
2		4.928	425779	77287			50.47

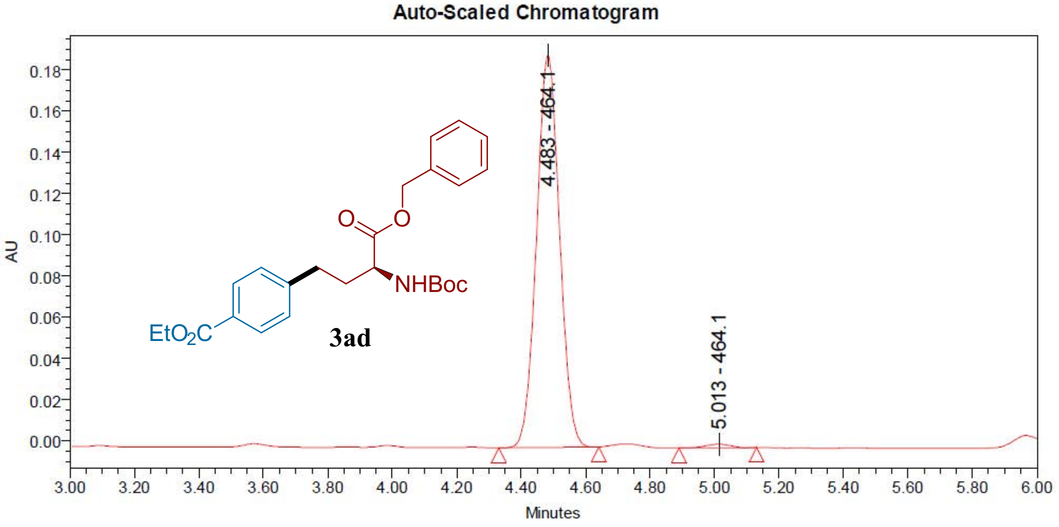

Peak Results

	Name	RT	Area	Height	Amount	Units	% Area
1		4.483	942399	190341			99.03
2		5.013	9261	1775			0.97

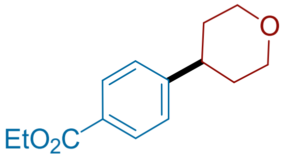

Ethyl 4-(tetrahydro-2H-pyran-4-yl)benzoate (3ae)

General Procedure 2.2 was followed using ethyl-4-bromobenzoate (49.0 µL, 68.7 mg, 0.3 mmol, 1 equiv), 1,3-dioxoisoindolin-2-yl tetrahydro-2H-pyran-4-carboxylate (99.1 mg, 0.36 mmol, 1.2 equiv), Na_2CO_3 (63.6 mg, 0.6 mmol, 2 equiv) as the additive, and TMS-DHP (135.9 mg, 0.6 mmol, 2 equiv) as the reductant in DMA (0.6 mL) for 10 h at 80 °C. **3ae** (56.9 mg, 0.24 mmol, 81%) was isolated as a colorless oil following column chromatography (gradient from 100% hexanes to 10% EtOAc/hexanes). **^{1}H NMR (500 MHz, $CDCl_3$)** δ 7.96 (AA'BB', $J_{AB+AB'}$, J = 8.4 Hz, 2H), 7.25 (AA'BB', $J_{AB+AB'}$, J = 8.4 Hz, 2H), 4.33 (q, J = 7.1 Hz, 2H), 4.04 (dd, J = 10.5, 4.3 Hz, 2H), 3.49 (td, J = 11.6, 2.5 Hz, 2H), 2.78 (tt, J = 11.7, 4.2 Hz, 1H), 1.83–1.71 (m, 4H), 1.34 (t, J = 7.1 Hz, 3H). **^{13}C{^{1}H} NMR (126 MHz, $CDCl_3$)** δ 166.6, 151.1, 130.0, 128.8, 126.8, 68.3, 60.9, 41.7, 33.7, 14.4. Characterization data matched those reported in the literature.[39]

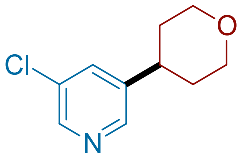

3-Chloro-5-(tetrahydro-2H-pyran-4-yl)pyridine (3af)

General Procedure 2.2 was followed using 3-bromo-5-chloropyridine (38.5 mg, 0.2 mmol, 1 equiv), 1,3-dioxoisoindolin-2-yl tetrahydro-2H-pyran-4-carboxylate (66.1 mg, 0.24 mmol, 1.2 equiv), Na_2CO_3 (42.4 mg, 0.4 mmol, 2 equiv) as the additive, and TMS-DHP (90.6 mg, 0.4 mmol, 2 equiv) as the reductant in DMA (0.2 mL) for 10 h at 80 °C. **3af** (32.8 mg, 0.17 mmol, 83%) was isolated as a colorless oil following reverse phase chromatography (water/acetonitrile mixture, gradient of 5% CH_3CN/H_2O–100% CH_3CN in H_2O). **^{1}H NMR (500 MHz, $CDCl_3$)** δ 8.44 (d, J = 2.4 Hz, 1H), 8.38 (d, J = 2.0 Hz, 1H), 7.52 (t, J = 2.1 Hz, 1H), 4.12–4.08 (m, 2H), 3.56–3.51 (m, 2H), 2.84–2.78 (m, 1H), 1.82–1.76 (m, 4H). **^{13}C{^{1}H} NMR (126 MHz, $CDCl_3$)** δ 146.9, 146.8, 142.3, 134.1, 132.2, 68.1, 38.9, 33.5. Characterization data matched those reported in the literature.[41]

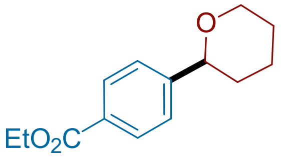

Ethyl 4-(tetrahydro-2H-pyran-2-yl)benzoate (3ag)

General Procedure 2.2 was followed using ethyl-4-bromobenzoate (49.0 µL, 68.7 mg, 0.3 mmol, 1 equiv), 1,3-dioxoisoindolin-2-yl tetrahydro-2H-pyran-2-carboxylate (99.1 mg, 0.36 mmol, 1.2 equiv), Na_2CO_3 (63.6 mg, 0.6 mmol, 2 equiv) as the additive, and TMS-DHP (135.9 mg, 0.6 mmol, 2 equiv) as the reductant in DMA (0.6 mL) for 10 h at 80 °C. **3ag** (64.7 mg, 0.28 mmol, 92%) was isolated as a colorless oil following column chromatography (gradient from 100% hexanes to 10% EtOAc/hexanes). **^{1}H NMR (500 MHz, $CDCl_3$)** δ 8.00 (AA'BB', $J_{AB+AB'}$, J = 8.4 Hz, 2H), 7.40 (AA'BB', $J_{AB+AB'}$, J = 8.4 Hz, 2H), 4.38–4.33 (m, 3H), 4.16–4.12 (m, 1H), 3.60 (td, J = 11.6, 2.5 Hz, 1H), 1.96–1.90 (m, 1H), 1.85–1.81 (m, 1H), 1.73–1.64 (m, 2H), 1.61–1.48 (m, 2H), 1.38 (t, J = 7.2 Hz, 3H). **^{13}C{^{1}H} NMR (126 MHz, $CDCl_3$)** δ 166.6, 148.5, 129.7, 129.4, 125.7, 79.6, 69.0, 60.9, 34.3, 25.9, 24.0, 14.4. Characterization data matched those reported in the literature.[42]

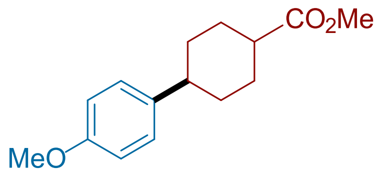

Methyl 4-(4-methoxyphenyl)cyclohexane-1-carboxylate (**3ah**)

General Procedure 2.2 was followed using 4-bromoanisole (25.1 µL, 37.4 mg, 0.2 mmol, 1 equiv), 1-(1,3-dioxoisoindolin-2-yl) 4-methyl cyclohexane-1,4-dicarboxylate (79.5 mg, 0.24 mmol, 1.2 equiv), Na_2CO_3 (42.4 mg, 0.4 mmol, 2 equiv) as the additive, and TMS-Me_4DHP (113.0 mg, 0.4 mmol, 2 equiv) as the reductant in toluene (0.4 mL) for 10 h at 80 °C. **3ah** (25.3 mg, 0.10 mmol, 50%) was isolated as a colorless oil (1:1 d.r.) following column chromatography (gradient from 100% hexanes to 4% EtOAc/hexanes). **^{1}H NMR (500 MHz, $CDCl_3$)** δ 7.12 (AA'BB', $J_{AB+AB'}$, J = 8.7 Hz, 2H), 6.86–6.82 (m, 2H), 3.79 (s, 1.5 H, diastereomer), 3.78 (s, 1.5 H, diastereomer), 3.72 (s, 1.5 H, diastereomer), 3.69 (s, 1.5 H, diastereomer), 2.72–2.69 (m, 0.5 H), 2.53–2.44 (m, 1H), 2.35 (tt, J = 12.2, 3.6 Hz, 0.5 H), 2.26–2.21 (m, 1H), 2.12–2.07 (m, 1H), 1.98–1.93 (m, 1H), 1.78–1.73 (m, 1H), 1.65–1.57 (m, 3H), 1.49–1.40 (m, 1H). **^{13}C{^{1}H} NMR (126 MHz, $CDCl_3$)** δ 176.6, 175.7, 158.0, 157.9, 139.4, 139.2, 127.8, 127.7, 113.9, 113.8, 55.37, 55.36, 51.7, 51.6, 43.1, 42.8, 42.7, 39.0, 33.6, 30.8, 29.5, 27.7. Both ^{1}H and ^{13}C NMR spectra showed a 1:1 mixture of the two diastereomers. Characterization data matched those reported in the literature.[41]

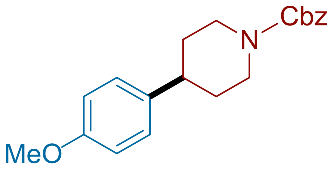

Benzyl 4-(4-methoxyphenyl)piperidine-1-carboxylate (**3ai**)

General Procedure 2.2 was followed using 4-bromoanisole (25.1 µL, 37.4 mg, 0.2 mmol, 1 equiv), 1-benzyl 4-(1,3-dioxoisoindolin-2-yl)piperidine-1,4-dicarboxylate (98.0 mg, 0.24 mmol, 1.2 equiv), Na_2CO_3 (42.4 mg, 0.4 mmol, 2 equiv) as the additive, and TMS-Me_4DHP (113.0 mg, 0.4 mmol, 2 equiv) as the reductant in toluene (0.4 mL) for 10 h at 80 °C. **3ai** (47.0 mg, 0.14 mmol, 72%) was isolated as a colorless oil following column chromatography (gradient from 100% hexanes to 17% EtOAc/hexanes). **^{1}H NMR (500 MHz, $CDCl_3$)** δ 7.39–7.35 (m, 4H), 7.32–7.29 (m, 1H), 7.10 (AA'BB', $J_{AB+AB'}$, J = 8.6 Hz, 2H), 6.84 (AA'BB', $J_{AB+AB'}$, J = 8.6 Hz, 2H), 5.15 (s, 2H), 4.31 (br s, 2H), 3.78 (s, 3H), 2.86 (br s, 2H), 2.61 (tt, J = 12.2, 3.6 Hz, 1H), 1.81 (br d, J = 11.4, 2H), 1.64–1.56 (m, 2H). **^{13}C{^{1}H} NMR (126 MHz, $CDCl_3$)** δ 158.2, 155.4, 137.9, 137.1, 128.6, 128.1, 128.0, 127.7, 114.0, 67.2, 55.4, 44.8, 41.8, 33.5. Characterization data matched those reported in the literature.[43]

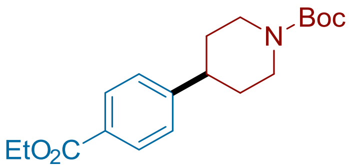

***tert*-Butyl-4-(4-(ethoxycarbonyl)phenyl)piperidine-1-carboxylate** (**3aj**)

General Procedure 2.2 was followed using ethyl-4-bromobenzoate (16.3 µL, 22.9 mg, 0.1 mmol, 1 equiv), 1-(tert-butyl)-4-(1,3-dioxoisoindolin-2-yl)piperidine-1,4-dicarboxylate (44.9 mg, 0.12 mmol, 1.2 equiv), Na_2CO_3 (21.2 mg, 0.2 mmol, 2 equiv) as the additive, and TMS-DHP (45.3

mg, 0.2 mmol, 2 equiv) as the reductant in DMA (0.2 mL) for 4 h at 30 °C. **3aj** (27.0 mg, 0.08 mmol, 81%) was isolated as a colorless oil following column chromatography (gradient from 100% hexanes to 12% EtOAc/hexanes). **^{1}H NMR (500 MHz, $CDCl_3$)** δ 7.97 (AA'BB', $J_{AB+AB'}$ = 8.0 Hz, 2H), 7.25 (AA'BB', $J_{AB+AB'}$ = 8.4 Hz, 2H), 4.34 (q, J = 7.1 Hz, 2H), 4.24 (s, 2H), 2.79 (t, J = 13.1 Hz, 2H), 2.69 (tt, J = 12.2, 3.6 Hz, 1H), 1.95–1.74 (m, 2H), 1.61 (qd, J = 12.5, 4.3 Hz, 2H), 1.47 (s, 9H), 1.36 (t, J = 7.1 Hz, 3H). **^{13}C{^{1}H} NMR (126 MHz, $CDCl_3$)** δ 166.6, 154.9, 151.0, 129.9, 128.8, 126.9, 79.6, 60.9, 44.3, 42.9, 33.0, 28.6, 14.4. Characterization data matched those reported in the literature.[30]

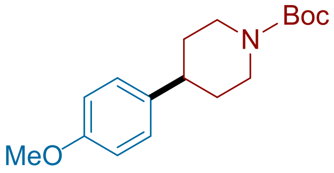

***tert*-Butyl-4-(4-methoxyphenyl)piperidine-1-carboxylate (3ak)**

General Procedure 2.2 was followed using 4-bromoanisole (25.1 µL, 37.4 mg, 0.2 mmol, 1 equiv), 1-(tert-butyl) 4-(1,3-dioxoisoindolin-2-yl)piperidine-1,4-dicarboxylate (89.8 mg, 0.12 mmol, 1.2 equiv), Na_2CO_3 (42.4 mg, 0.4 mmol, 2 equiv) as the additive, and TMS-Me_4DHP (113.0 mg, 0.4 mmol, 2 equiv) as the reductant in toluene (0.4 mL) for 10 h at 80 °C. **3ak** (39.0 mg, 0.134 mmol, 67%) was isolated as a colorless oil following column chromatography (gradient from 100% hexanes to 6% EtOAc/hexanes). **^{1}H NMR (500 MHz, $CDCl_3$)** δ 7.12 (AA'BB', $J_{AB+AB'}$, J = 8.6 Hz, 2H), 6.85 (AA'BB', $J_{AB+AB'}$, J = 8.7 Hz, 2H), 4.23 (s, 2H), 3.79 (s, 3H), 2.79 (t, J = 13.1 Hz, 2H), 2.59 (tt, J = 12.2, 3.6 Hz, 1H), 1.81–1.77 (m, 2H), 1.62–1.54 (m, 2H), 1.48 (s, 9H). **^{13}C NMR{^{1}H} (126 MHz, $CDCl_3$)** δ 158.2, 155.0, 138.1, 127.7, 114.0, 79.5, 55.4, 44.6, 42.0, 33.5, 28.6. Characterization data matched those reported in the literature.[44]

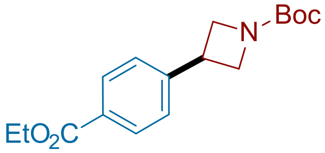

***tert*-Butyl-3-(4-(ethoxycarbonyl)phenyl)azetidine-1-carboxylate (3al)**

General Procedure 2.2 was followed using ethyl-4-bromobenzoate (49.0 µL, 68.7 mg, 0.3 mmol, 1 equiv), 1-(tert-butyl)-3-(1,3-dioxoisoindolin-2-yl) azetidine-1,3-dicarboxylate (129.3 mg, 0.36 mmol, 1.2 equiv), Na_2CO_3 (63.6 mg, 0.6 mmol, 2 equiv) as the additive, and TMS-DHP (135.9 mg, 0.6 mmol, 2 equiv) as the reductant in DMA (0.6 mL) for 10 h at 80 °C. **3al** (34.8 mg, 0.11 mmol, 38%) was isolated as a colorless oil following column chromatography (gradient from 100% hexanes to 12% EtOAc/hexanes). **^{1}H NMR (500 MHz, $CDCl_3$)** δ 8.01 (AA'BB', $J_{AB+AB'}$, J = 8.2 Hz, 2H), 7.36 (AA'BB', $J_{AB+AB'}$, J = 8.2 Hz, 2H), 4.39–4.32 (m, 4H), 3.96 (dd, J = 8.6, 5.9 Hz, 2H), 3.77 (tt, J = 8.7, 5.9 Hz, 1H), 1.46 (s, 9H), 1.38 (t, J = 7.1 Hz, 3H). **^{13}C{^{1}H} NMR (126 MHz, $CDCl_3$)** δ 166.4, 156.5, 147.5, 130.1, 129.4, 126.9, 79.8, 61.1, 56.4, 33.6, 28.5, 14.5. Characterization data matched those reported in the literature.[39]

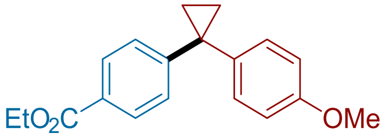

Ethyl 4-(1-(4-methoxyphenyl)cyclopropyl)benzoate (3am)

General Procedure 2.2 was followed using ethyl-4-bromobenzoate (32.6 µL, 45.8 mg, 0.2 mmol, 1 equiv), 1,3-dioxoisoindolin-2-yl-1-(4-methoxyphenyl)cyclopropane-1-carboxylate (80.9 mg, 0.24 mmol, 1.2 equiv), Na_2CO_3 (42.2 mg, 0.4 mmol, 2 equiv) as the additive, and TMS-DHP (90.6 mg, 0.4 mmol, 2 equiv) as the reductant in DMA (0.4 mL) for 4 h at 80 °C. **3am** (39.7 mg, 0.13 mmol, 67%) was isolated as a white solid following column chromatography (gradient from 100% hexanes to 15% EtOAc/hexanes). **^{1}H NMR (500 MHz, $CDCl_3$)** δ 7.90 (AA'BB', $J_{AB+AB'}$, J = 8.7 Hz, 2H), 7.21–7.17 (m, 4H), 6.83 (AA'BB', $J_{AB+AB'}$, J = 8.7 Hz, 2H), 4.34 (q, J = 7.1 Hz, 2H), 3.79 (s, 3H), 1.36 (t, J = 7.1 Hz, 3H), 1.34–1.26 (m, 4H). **^{13}C{^{1}H} NMR (126 MHz, $CDCl_3$)** δ 166.7, 158.3, 151.9, 136.8, 130.4, 129.6, 128.0, 127.5, 114.0, 60.9, 55.4, 29.4, 17.1, 14.5. **HRMS-ESI (m/z)**: $[M+H]^+$ calcd for $C_{18}H_{20}O_3^+$, 297.1485, found 297.1480.

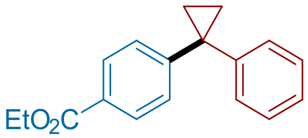

Ethyl 4-(1-phenylcyclopropyl)benzoate (3an)

General Procedure 2.2 was followed using ethyl-4-bromobenzoate (32.6 µL, 45.8 mg, 0.2 mmol, 1 equiv), 1,3-dioxoisoindolin-2-yl-1-phenylcyclopropane-1-carboxylate (73.7 mg, 0.24 mmol, 1.2 equiv), Na_2CO_3 (42.2 mg, 0.4 mmol, 2 equiv) as the additive, and TMS-DHP (90.6 mg, 0.4 mmol, 2 equiv) as the reductant in DMA (0.4 mL) for 4 h at 80 °C. **3an** (27.7 mg, 0.10 mmol, 52%) was isolated as a colorless oil following column chromatography (gradient from 100% hexanes to 18% EtOAc/hexanes). **^{1}H NMR (500 MHz, $CDCl_3$)** δ 7.85 (AA'BB', $J_{AB+AB'}$, J = 10.4 Hz, 2H), 7.22–7.11 (m,7H), 4.28 (q, J = 7.1 Hz, 2H), 1.30–1.24 (m, 7H). **^{13}C{^{1}H} NMR (126 MHz, $CDCl_3$)** δ 166.7, 151.4, 144.81, 129.7, 128.9, 128.6, 128.2, 128.0, 126.5, 60.9, 30.1, 17.1, 14.5. **HRMS-ESI (m/z)**: $[M+H]^+$ calcd for $C_{18}H_{19}O_2^+$, 267.1380, found 267.1377.

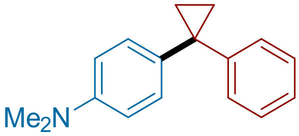

***N,N*-Dimethyl-4-(1-phenylcyclopropyl)aniline (3ao)**

General Procedure 2.2 was followed using 4-bromo-*N*,*N*-dimethylaniline (20.0 mg, 0.1 mmol, 1 equiv), 1,3-dioxoisoindolin-2-yl-1-phenylcyclopropane-1-carboxylate (36.9 mg, 0.12 mmol, 1.2 equiv), Na_2CO_3 (21.2 mg, 0.2 mmol, 2 equiv) as the additive, and TMS-DHP (45.3 mg, 0.2 mmol, 2 equiv) as the reductant in toluene (0.2 mL) for 10 h at 80 °C. **3ao** (7.9 mg, 0.033 mmol, 33%) was isolated as a colorless oil following column chromatography (gradient from 100% hexanes to 15% EtOAc/hexanes). **^{1}H NMR (400 MHz, $CDCl_3$)** δ 7.25–7.13 (m, 7H), 6.67 (AA'BB', $J_{AB+AB'}$, J = 8.8 Hz, 2H), 2.91 (s, 6H), 1.26–1.21 (m, 4H). **^{13}C{^{1}H} NMR (125 MHz, $CDCl_3$)** δ 148.0, 145.7, 132.6, 128.5, 127.1, 126.8, 124.4, 111.6, 39.7, 28.0, 15.2. Characterization data matched those reported in the literature.[6]

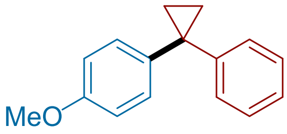

1-Methoxy-4-(1-phenylcyclopropyl)benzene (3ap)

General Procedure 2.2 was followed using 4-bromoanisole (25.2 µL, 37.4 mg, 0.2 mmol, 1 equiv), 1,3-dioxoisoindolin-2-yl-1-phenylcyclopropane-1-carboxylate (73.8 mg, 0.24 mmol, 1.2 equiv), Na_2CO_3 (42.4 mg, 0.4 mmol, 2 equiv) as the additive, and TMS-DHP (90.6 mg, 0.4 mmol, 2 equiv) as the reductant in toluene (0.4 mL) for 10 h at 80 °C. **3ap** (28.3 mg, 0.13 mmol, 63%) was isolated as a colorless oil following column chromatography (gradient from 100% hexanes to 10% EtOAc/hexanes). **^{1}H NMR (500 MHz, $CDCl_3$)** δ 7.26–7.23 (m, 2H), 7.20–7.13 (m, 5H), 6.82 (AA'BB', $J_{AB+AB'}$, J = 8.8 Hz, 2H), 3.78 (s, 3H), 1.26–1.23 (m, 4H). **^{13}C{^{1}H} NMR (125 MHz, $CDCl_3$)** δ 158.0, 146.4, 137.9, 130.0, 128.3, 128.0, 125.9, 113.8, 55.4, 29.3, 16.4. Characterization data matched those reported in the literature.[6]

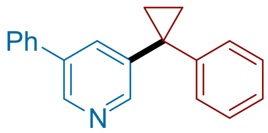

3-Phenyl-5-(1-phenylcyclopropyl)pyridine (**3aq**)

General Procedure 2.2 was followed using 3-bromo-5-phenylpyridine (46.8 mg, 0.2 mmol, 1 equiv), 1,3-dioxoisoindolin-2-yl-1-phenylcyclopropane-1-carboxylate (73.8 mg, 0.24 mmol, 1.2 equiv), DIPEA (69.6 µL, 51.6 mg, 0.4 mmol, 2 equiv) as the additive, and TMS-Me_4DHP (113.0 mg, 0.4 mmol, 2 equiv) as the reductant in toluene (0.4 mL) for 10 h at 80 °C. **3aq** (36.9 mg, 0.14 mmol, 68%) was isolated as a pale yellow oil following column chromatography (gradient from 100% hexanes to 18% EtOAc/hexanes). **^{1}H NMR (500 MHz, $CDCl_3$)** δ 8.66 (d, J = 2.1 Hz, 1H), 8.49 (d, J = 2.5 Hz, 1H), 7.71 (t, J = 2.3 Hz, 1H), 7.55 – 7.52 (m, 2H), 7.47–7.44 (m, 2H), 7.40–7.37 (m, 1H), 7.31–7.25 (m, 4H), 7.22–7.19 (m, 1H), 1.41–1.35 (m, 4H). **^{13}C{^{1}H} NMR (125 MHz, $CDCl_3$)** δ 147.7, 144.8, 143.3, 140.0, 136.8, 135.0, 133.3, 127.9, 127.4, 127.3, 126.9, 126.1, 125.3, 26.9, 14.5. **HRMS–ESI (m/z)**: $[M+H]^+$ calcd for $C_{20}H_{18}N^+$, 272.1434, found 272.1430.

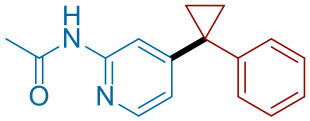

***N*-(4-(1-phenylcyclopropyl)pyridin-2-yl)acetamide** (**3ar**)

General Procedure 2.2 was followed using *N*-(4-bromopyridin-2-yl)acetamide (43.0 mg, 0.2 mmol, 1 equiv), 1,3-dioxoisoindolin-2-yl-1-phenylcyclopropane-1-carboxylate (73.8 mg, 0.24 mmol, 1.2 equiv), Na_2CO_3 (42.4 mg, 0.4 mmol, 2 equiv) as the additive, and TMS-Me_4DHP (113.0 mg, 0.4 mmol, 2 equiv) as the reductant in toluene (0.4 mL) for 10 h at 80 °C. **3ar** (16.1 mg, 0.064 mmol, 32%) was isolated as a white solid following reverse phase chromatography (water/acetonitrile mixture, gradient of 5% CH_3CN/H_2O–60% CH_3CN in H_2O), the . **^{1}H NMR (500 MHz, $CDCl_3$)** δ 8.42 (s, 1H), 7.99 (d, J = 5.4 Hz, 1H), 7.91 (br s, 1H), 7.29–7.25 (m, 4H), 7.22–7.19 (m, 1H), 6.65 (dd, J = 5.4, 1.7 Hz, 1H), 2.10 (s, 3H), 1.35–1.33 (m, 4H). **^{13}C{^{1}H} NMR (126 MHz, $CDCl_3$)** δ 168.8, 158.5, 151.9, 147.3, 143.4, 129.8, 128.7, 127.0, 119.3, 111.7, 29.8, 24.8, 17.6. Characterization data matched those reported in the literature.[41]

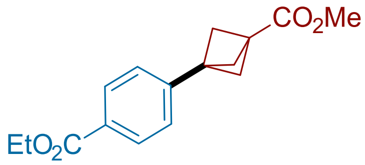

Methyl 3-(4-(ethoxycarbonyl)phenyl)bicyclo[1.1.1]pentane-1-carboxylate (3as)

General Procedure 2.2 was followed using ethyl-4-bromobenzoate (32.6 µL, 45.8 mg, 0.2 mmol, 1 equiv), 1-(1,3-dioxoisoindolin-2-yl)-3-methyl bicyclo[1.1.1]pentane-1,3-dicarboxylate (75.7 mg, 0.24 mmol, 1.2 equiv), Na_2CO_3 (42.2 mg, 0.4 mmol, 2 equiv) as the additive, and TMS-DHP (90.6 mg, 0.4 mmol, 2 equiv) as the reductant in DMA (0.4 mL) for 10 h at 80 °C. **3as** (24.1 mg, 0.088 mmol, 44%) was isolated as a white solid following column chromatography (gradient from 100% hexanes to 15% EtOAc/hexanes). **^{1}H NMR (500 MHz, $CDCl_3$)** δ 7.98 (AA'BB', $J_{AB+AB'}$, J = 8.2 Hz, 2H), 7.26 (AA'BB', $J_{AB+AB'}$, J = 8.2 Hz, 2H), 4.37 (q, J = 7.1 Hz, 2H), 3.72 (s, 3H), 2.35 (s, 6H), 1.39 (t, J = 7.1 Hz, 3H). **^{13}C{^{1}H} NMR (126 MHz, $CDCl_3$)** δ 170.6, 166.6, 144.7, 129.7, 129.3, 126.2, 61.1, 53.6, 51.9, 41.8, 37.2, 14.5. Characterization data matched those reported in the literature.[24]

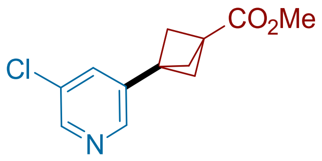

Methyl 3-(5-chloropyridin-3-yl)bicyclo[1.1.1]pentane-1-carboxylate (3at)

General Procedure 2.2 was followed using 3-bromo-5-chloropyridine (38.5 mg, 0.2 mmol, 1 equiv), 1-(1,3-dioxoisoindolin-2-yl)-3-methyl bicyclo[1.1.1]pentane-1,3-dicarboxylate (75.7 mg, 0.24 mmol, 1.2 equiv), Na_2CO_3 (42.2 mg, 0.4 mmol, 2 equiv) as the additive, and TMS-Me_4DHP (113.0 mg, 0.4 mmol, 2 equiv) as the reductant in DMA (0.4 mL) for 10 h at 80 °C. **3at** (19.5 mg, 0.082 mmol, 41% yield from ^{1}H NMR) was isolated as a white solid following column chromatography (gradient from 100% hexanes to 15% EtOAc/hexanes). The product contained 2% phthalimide that was inseparable. **^{1}H NMR (500 MHz, $CDCl_3$)** δ 8.46 (d, J = 2.4 Hz, 1H), 8.34 (d, J = 1.9 Hz, 1H), 7.49 (t, J = 2.1 Hz, 1H), 3.72 (s, 3H), 2.37 (s, 6H). **^{13}C{^{1}H} NMR (126 MHz, $CDCl_3$)** δ 170.0, 147.4, 145.9, 136.4, 133.8, 132.0, 53.6, 52.0, 39.5, 37.7. Characterization data matched those reported in the literature.[45]

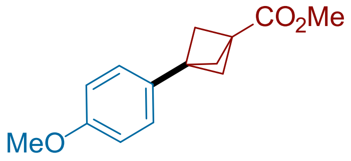

Methyl 3-(4-methoxyphenyl)bicyclo[1.1.1]pentane-1-carboxylate (3au)

General Procedure 2.2 was followed using 4-bromoanisole (31.4 µL, 46.8 mg, 0.25 mmol, 1 equiv), 1-(1,3-dioxoisoindolin-2-yl)-3-methyl bicyclo[1.1.1]pentane-1,3-dicarboxylate (94.5 mg, 0.30 mmol, 1.2 equiv), Na_2CO_3 (53.0 mg, 0.5 mmol, 2 equiv) as the additive, and TMS-Me_4DHP (141.3 mg, 0.5 mmol, 2 equiv) as the reductant in toluene (0.5 mL) for 10 h at 80 °C. **3au** (30.2 mg, 0.13 mmol, 52%) was isolated as a white solid following column chromatography (gradient from 100% hexanes to 15% EtOAc/hexanes). **^{1}H NMR (500 MHz, $CDCl_3$)** δ 7.14 (AA'BB', $J_{AB+AB'}$, J = 8.5 Hz, 2H), 6.85 (AA'BB', $J_{AB+AB'}$, J = 8.5 Hz, 2H), 3.79 (s, 3H), 3.71 (s, 3H), 2.29 (s, 6H). **^{13}C{^{1}H} NMR (126 MHz, $CDCl_3$)** δ 170.9, 158.8, 132.1, 127.3, 113.8, 55.4, 53.6, 51.8, 41.5, 37.0. Characterization data matched those reported in the literature.[6]

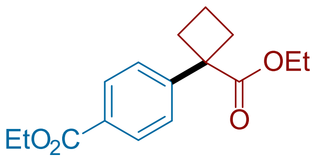

Ethyl 4-(1-(ethoxycarbonyl)cyclobutyl)benzoate (3av)

General Procedure 2.2 was followed using ethyl-4-bromobenzoate (49.0 µL, 68.7 mg, 0.3 mmol, 1 equiv), 1-(1,3-dioxoisoindolin-2-yl)-1-ethyl cyclobutane-1,1-dicarboxylate (114.2 mg, 0.36 mmol, 1.2 equiv), Na_2CO_3 (63.6 mg, 0.6 mmol, 2 equiv) as the additive, and TMS-DHP (135.9 mg, 0.6 mmol, 2 equiv) as the reductant in DMA (0.6 mL) for 10 h at 80 °C. **3av** (31.5 mg, 0.11 mmol, 38%) was isolated as a white solid following column chromatography (gradient from 100% hexanes to 15% EtOAc/hexanes). **^{1}H NMR (500 MHz, $CDCl_3$)** δ 8.00 (AA'BB', $J_{AB+AB'}$, J = 8.5 Hz, 2H), 7.36 (AA'BB', $J_{AB+AB'}$, J = 8.5 Hz, 2H), 4.37 (q, J = 7.1 Hz, 2H), 4.10 (q, J = 7.1 Hz, 2H), 2.88–2.83 (m, 2H), 2.54–2.48 (m, 2H), 2.12–2.04 (m, 1H), 1.92–1.84 (m, 1H), 1.39 (t, J = 7.1 Hz, 3H), 1.16 (t, J = 7.1 Hz, 3H). **^{13}C{^{1}H} NMR (126 MHz, $CDCl_3$)** δ 175.2, 166.5, 148.9, 129.5, 128.8, 126.3, 61.1, 60.9, 52.6, 32.4, 16.7, 14.4, 14.0. **HRMS-ESI (m/z)**: $[M+H]^+$ calcd for $C_{16}H_{21}O_4^+$, 277.1434, found 277.1430.

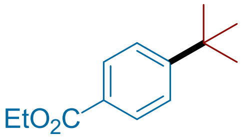

Ethyl 4-(*tert*-butyl)benzoate (3aw)

General Procedure 2.2 was followed using 1-bpp (8 mol%) as the ligand instead of dtbbpy, ethyl-4-bromobenzoate (49 µL, 68.7 mg, 0.3 mmol, 1 equiv), 1,3-dioxoisoindolin-2-yl pivalate (111.3 mg, 0.45 mmol, 1.5 equiv), Na_2CO_3 (63.6 mg, 0.6 mmol, 2 equiv) as the additive, and TMS-DHP (135.9 mg, 0.6 mmol, 2 equiv) as the reductant in DMA (0.6 mL) for 10 h at 80 °C. **3aw** (21.4 mg, 0.10 mmol, 34% yield from ^{1}H NMR) was isolated as a colorless oil following column chromatography (100% hexanes). The product contained 17% aryl bromide (ethyl-4-bromobenzoate) that was inseparable. **^{1}H NMR (500 MHz, $CDCl_3$)** δ 8.00 (AA'BB', $J_{AB+AB'}$, J = 8.5 Hz, 2H), 7.45 (AA'BB', $J_{AB+AB'}$, J = 8.5 Hz, 2H), 4.36 (q, J = 7.1 Hz, 2H), 1.38 (t, J = 7.2 Hz, 3H), 1.33 (s, 9H). **^{13}C{^{1}H} NMR (126 MHz, $CDCl_3$)** δ 166.8, 156.6, 129.5, 127.9, 125.4, 60.9, 35.2, 31.3, 14.5. Characterization data matched those reported in the literature.[46] We verified that the ratio of **3aw** to the isomerized product **3ab** was 34:1 (97% **3aw**, 3% **3ab**). observed by comparing quantitative GC-FID chromatograms (see below).

Additional Info : Peak(s) manually integrated

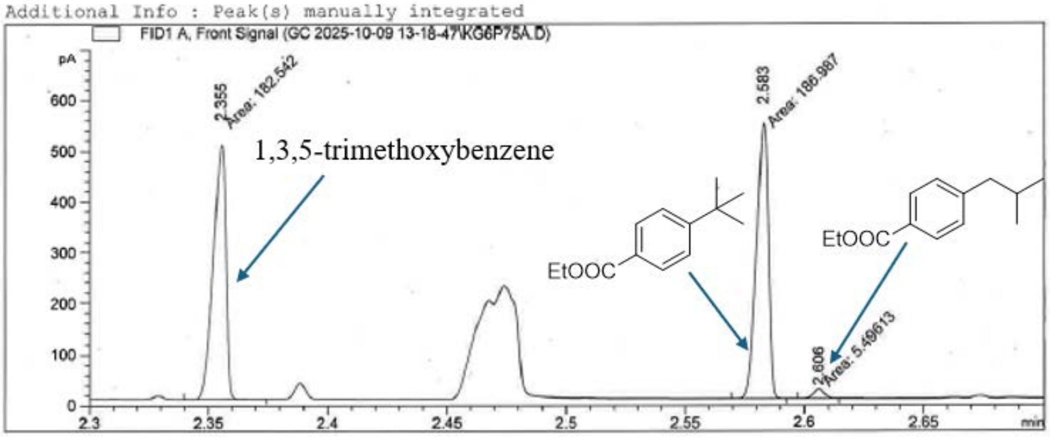


```
=====================================================================
                          Area Percent Report
=====================================================================

Sorted By             :      Retention Time
Multiplier            :      1.0000
Dilution              :      1.0000
Do not use Multiplier & Dilution Factor with ISTDs

Signal 1: FID1 A, Front Signal

Peak RetTime Sig Type    Area       Height      Area
  #   [min]              [pA*s]     [pA]         %
----|-------|---|----|----------|----------|--------|
   1   2.355   1  MM    182.54158  537.42572 48.67451
   2   2.583   1  MM    186.98729  568.83734 49.85995
   3   2.606   1  MM      5.49613   17.83577  1.46554
```

Additional Info : Peak(s) manually integrated

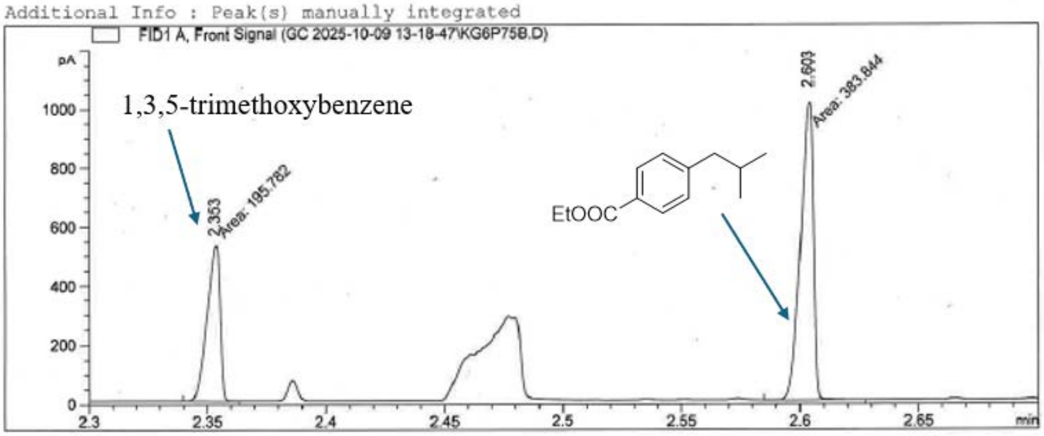


```
=====================================================================
                          Area Percent Report
=====================================================================

Sorted By             :      Retention Time
Multiplier            :      1.0000
Dilution              :      1.0000
Do not use Multiplier & Dilution Factor with ISTDs

Signal 1: FID1 A, Front Signal

Peak RetTime Sig Type    Area       Height      Area
  #   [min]              [pA*s]     [pA]         %
----|-------|---|----|----------|----------|--------|
   1   2.353   1  MM    195.78152  540.82202 33.77725
   2   2.603   1  MM    383.84384 1048.11743 66.22275
```

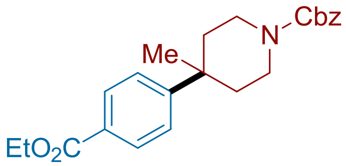

N-benzyloxycarbonyl-4-(p-ethoxycarbonylphenyl)-4-methylpiperidine (**3ax**)

General Procedure 2.2 was followed using 1-bpp (8 mol%) as the ligand instead of dtbbpy, ethyl-4-bromobenzoate (49 µL, 68.7 mg, 0.3 mmol, 1 equiv), 1-benzyl-4-(1,3-dioxoisoindolin-2-yl)-4-methylpiperidine-1,4-dicarboxylate (190.1 mg, 0.45 mmol, 1.2 equiv), Na_2CO_3 (63.6 mg, 0.6 mmol, 2 equiv) as the additive, and TMS-DHP (135.9 mg, 0.6 mmol, 2 equiv) as the reductant in DMA (0.6 mL) for 10 h at 80 °C. **3ax** (29.7 mg, 0.078 mmol, 26%) was isolated as a white solid following column chromatography (gradient from 100% hexanes to 15% EtOAc/hexanes). ^{1}H NMR (500 MHz, $CDCl_3$) δ 8.01 (AA'BB', $J_{AB+AB'}$, J = 8.6 Hz, 2H), 7.39 (AA'BB', $J_{AB+AB'}$, J = 8.6 Hz, 2H), 7.36–7.29 (m, 5H), 5.13 (s, 2H), 4.38 (q, J = 7.1 Hz, 2H), 3.59 (ddd, J = 13.5, 7.7, 3.5 Hz, 2H), 3.45 (ddd, J = 13.4, 7.7, 3.6 Hz, 2H), 2.18–2.07 (m, 2H), 1.75–1.71 (m, 2H), 1.39 (t, J = 7.1 Hz, 3H), 1.27 (s, 3H). **^{13}C{^{1}H} NMR (126 MHz, $CDCl_3$)** δ 166.6, 155.5, 153.3, 137.0, 130.0, 128.6, 128.4, 128.1, 128.0, 125.8, 67.2, 61.0, 40.8, 37.03, 36.63, 28.9, 14.5. **HRMS-ESI (m/z)**: $[M+H]^+$ calcd for $C_{23}H_{28}NO_4^+$, 382.2013, found 382.2007.

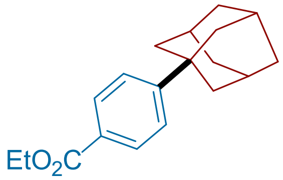

Ethyl 4-(1-adamantyl)benzoate (**3ay**)

General Procedure 2.2 was followed using a mixture of 1-bpp (4 mol%) and dtbbpy (4 mol%) as the ligand instead of only dtbbpy, ethyl 4-bromobenzoate (32.6 µL, 45.8 mg, 0.2 mmol, 1 equiv), 1,3-dioxoisoindolin-2-yl (3r,5r,7r)-adamantane-1-carboxylate (78.0 mg, 0.24 mmol, 1.2 equiv), Na_2CO_3 (42.4 mg, 0.4 mmol, 2 equiv) as the additive, and TMS-DHP (90.6 mg, 0.4 mmol, 2 equiv) as the reductant in DMA (0.4 mL) for 10 h at 80 °C. **3ay** (16.0 mg, 0.056 mmol, 28%) was isolated as a white solid following column chromatography (gradient from 100% hexanes to 15% EtOAc/hexanes). Upon using 20 mol% $NiBr_2$(dme), 10 mol% dtbbpy and 10 mol% 1-bpp under similar conditions, **3ay** was obtained in 48% isolated yield by ^{1}H NMR (27.3 mg, 0.096 mmol). **^{1}H NMR (500 MHz, $CDCl_3$)** δ 7.93 (AA'BB', $J_{AB+AB'}$, J = 8.7 Hz, 2H), 7.36 (AA'BB', $J_{AB+AB'}$, J = 8.7 Hz, 2H), 4.29 (q, J = 7.2 Hz, 2H), 2.05–2.03 (m, 3H), 1.86 (d, J = 2.9 Hz, 6H), 1.76–1.67 (m, 6H), 1.31 (t, J = 7.1 Hz, 3H). **^{13}C{^{1}H} NMR (126 MHz, $CDCl_3$)** δ 166.7, 156.5, 129.4, 127.8, 124.9, 60.7, 42.9, 36.70, 36.65, 28.8, 14.4. Characterization data matched those reported in the literature.[46]

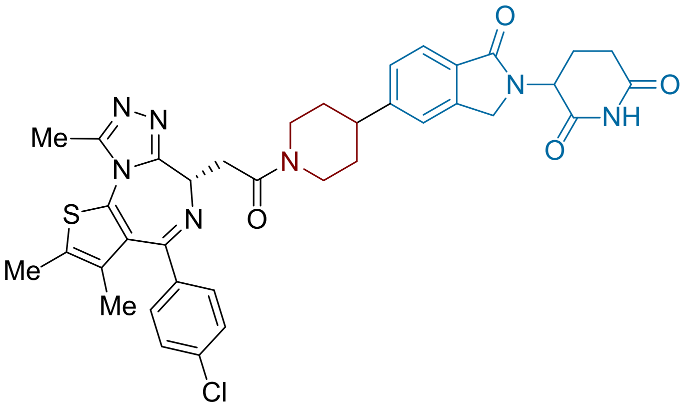

3-(5-(1-(2-((S)-4-(4-chlorophenyl)-2,3,9-trimethyl-6H-thieno[3,2-f][1,2,4]triazolo[4,3-a][1,4]diazepin-6-yl)acetyl)piperidin-4-yl)-1-oxoisoindolin-2-yl)piperidine-2,6-dione (3az)

General Procedure 2.6 was followed. **^{1}H NMR (600 MHz, DMSO-D$_6$)** δ 10.97 (s, 1H), 7.68–7.66 (m, 1H), 7.53–7.42 (m, 6H), 5.12–5.09 (m, 2H), 4.65–4.62 (m, 1H), 4.55 (br d, J = 12.6 Hz, 1H), 4.45 – 4.42 (m, 1H), 4.33–4.29 (m, 2H), 3.71–3.65 (m, 1H), 3.48–3.43 (m, 1H), 3.27 (br t, *J* = 13.2 Hz, 1H), 3.02–2.97 (m, 1H), 2.94–2.88 (m, 1H), 2.74–2.67 (m, 1H), 2.63–2.59 (m, 4H), 2.42–2.36 (m, 4H), 2.00–1.98 (m, 1H), 1.92 (br d, J = 12.3 Hz, 1H), 1.89–1.81 (m, 1H), 1.64 (s, 3H), 1.54–1.49 (m, 1H). **^{13}C-APT{^{1}H} NMR (150 MHz, DMSO-D$_6$)** δ. 173.0, 171.2, 168.1, 163.3, 155.4, 150.1, 142.7, 136.8, 135.5, 132.2, 131.1, 130.4, 130.1, 130.0, 128.6, 127.1, 123.2, 121.9, 54.3, 51.2, 47.3, 45.8, 42.2, 34.8, 32.8, 31.4, 22.7, 14.1, 12.8, 11.4. Characterization data matched those reported in the literature.[47]

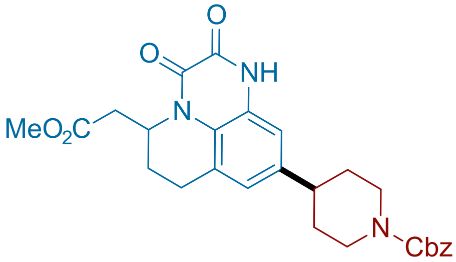

Benzyl 4-(5-(2-methoxy-2-oxoethyl)-2,3-dioxo-2,3,6,7-tetrahydro-1H,5H-pyrido[1,2,3-de]quinoxalin-9-yl)piperidine-1-carboxylate (**4a**)

General Procedure 2.2 was followed using NiBr$_2$(dme) (10 mol%) and dtbbpy (10 mol%) for the catalyst pre-stir, the informer bromide **X1** (42.7 mg, 0.1 mmol, 1 equiv), 1-benzyl 4-(5-methyl-1,3-dioxoisoindolin-2-yl) piperidine-1,4-dicarboxylate (NHP ester-3, 50.7 mg, 0.12 mmol, 1.2 equiv), Na$_2$CO$_3$ (21.2 mg, 0.2 mmol, 2 equiv) as the additive, and TMS-Me$_4$DHP (56.5 mg, 0.2 mmol, 2 equiv) as the reductant in a mixture of toluene (0.2 mL) and DMA (0.2 mL) for 15 h at 80 °C. **4a** (46.8 mg, 0.095 mmol, 95%) was isolated as a light brown solid following column chromatography (gradient from 100% hexanes to 100% EtOAc/hexanes, followed by 100% DCM to 30% MeOH/DCM).**^{1}H NMR (500 MHz, CDCl$_3$)** δ 11.75 (s, 1H), 7.40–7.34 (m, 4H), 7.33–7.30 (m, 1H), 7.04 (s, 1H), 6.87 (s, 1H), 5.44–5.40 (m, 1H), 5.16 (s, 2H), 4.35 (br s, 2H), 3.72 (s, 3H), 3.04–2.95 (m, 1H), 2.88 (br s, 1H), 2.85–2.80 (m, 3H), 2.70 (tt, *J* = 12.1, 3.6 Hz, 1H), 2.59–2.54 (m, 1H), 2.34–2.29 (m, 1H), 2.00 (tt, *J* = 14.0, 4.7 Hz, 1H), 1.82 (d, *J* = 12.9 Hz, 2H), 1.67–1.60 (m, 3H). **^{13}C{^{1}H} NMR (126 MHz, CDCl$_3$)** δ 170.6, 155.8, 155.4, 154.1, 142.2, 137.0, 128.6, 128.13, 128.08, 124.93, 124.90, 122.9, 121.4, 113.6, 67.3, 52.2, 47.9, 44.6, 42.0, 35.4, 33.1, 23.4, 22.0. **HRMS-ESI (m/z)**: $[M+H]^+$ calcd for $C_{27}H_{30}N_3O_6^+$, 492.2129, found 492.2125.

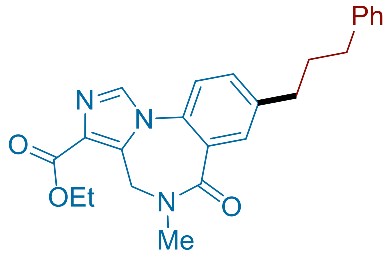

Ethyl 5-methyl-6-oxo-8-(3-phenylpropyl)-5,6-dihydro-4H-benzo[f]imidazo[1,5-a][1,4]diazepine-3-carboxylate (4b)

General Procedure 2.2 was followed using $NiBr_2$(dme) (10 mol%) and dtbbpy (10 mol%) for the catalyst pre-stir, the informer bromide **X2** (36.4 mg, 0.1 mmol, 1 equiv), 1,3-dioxoisoindolin-2-yl-4-phenylbutanoate (37.1 mg, 0.12 mmol, 1.2 equiv), Na_2CO_3 (21.2 mg, 0.2 mmol, 2 equiv) as the additive, and TMS-DHP (45.3 mg, 0.2 mmol, 2 equiv) as the reductant in DMA (0.4 mL) for 12 h at 80 °C. **4b** (32.7 mg, 0.081 mmol, 81%) was isolated as a white solid following column chromatography (gradient from 100% hexanes to 100% EtOAc/hexanes, followed by 100% EtOAc to 10% ^{i}PrOH/EtOAc). **^{1}H NMR (500 MHz, $CDCl_3$)** δ 7.89–7.87 (m, 2H), 7.44–7.42 (m, 1H), 7.34–7.27 (m, 3H), 7.21–7.18 (m, 3H), 5.18 (s, 1H), 4.46–4.37 (m, 3H), 3.25 (s, 3H), 2.75 (t, J = 7.8 Hz, 2H), 2.69 (t, J = 7.6 Hz, 2H), 2.02 (pentet, J = 7.7 Hz, 2H), 1.45 (t, J = 7.2 Hz, 3H). **^{13}C{^{1}H} NMR (126 MHz, $CDCl_3$)** δ 166.8, 163.2, 143.3, 141.7, 135.7, 135.0, 132.8, 132.4, 130.1, 129.0, 128.7, 128.5, 126.1, 121.9, 61.0, 42.5, 35.9, 35.4, 34.8, 32.6, 14.5. **HRMS-ESI (m/z)**: $[M+H]^+$ calcd for $C_{24}H_{26}N_3O_3^+$, 404.1969, found 404.1965.

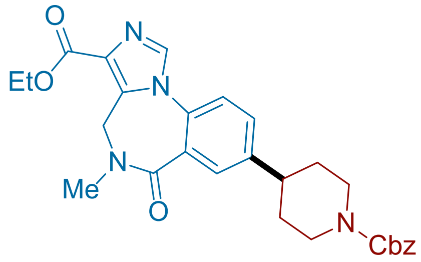

Ethyl 8-(1-((benzyloxy)carbonyl)piperidin-4-yl)-5-methyl-6-oxo-5,6-dihydro-4H-benzo[f]imidazo[1,5-a][1,4]diazepine-3-carboxylate (4c)

General Procedure 2.2 was followed using $NiBr_2$(dme) (10 mol%) and dtbbpy (10 mol%) for the catalyst pre-stir, the informer bromide **X2** (36.4 mg, 0.1 mmol, 1 equiv), 1-benzyl 4-(1,3-dioxoisoindolin-2-yl)piperidine-1,4-dicarboxylate (49.0 mg, 0.12 mmol, 1.2 equiv), Na_2CO_3 (21.2 mg, 0.2 mmol, 2 equiv) as the additive, and TMS-DHP (45.3 mg, 0.2 mmol, 2 equiv) as the reductant in DMA (0.4 mL) for 12 h at 80 °C. **4c** (36.2 mg, 0.072 mmol, 72%) was isolated as a white solid following column chromatography (gradient from 100% hexanes to 100% EtOAc/hexanes). **^{1}H NMR (500 MHz, $CDCl_3$)** δ 7.90 (d, J = 2.1 Hz, 1H), 7.87 (s, 1H), 7.46 (dd, J = 8.3, 2.2 Hz, 1H), 7.39–7.31 (m, 6H), 5.16 (s, 2H), 4.45–4.43 (m, 2H), 4.37 (s, 2H), 3.25 (s, 3H), 2.91 (s, 2H), 2.81 (tt, J = 12.2, 3.6 Hz, 1H), 1.90 (d, J = 13.0 Hz, 2H), 1.68 (tt, J = 12.4, 7.1 Hz, 2H), 1.65–1.56 (m, 2H), 1.45 (t, J = 7.2 Hz, 3H). **^{13}C{^{1}H} NMR (126 MHz, $CDCl_3$)** δ 166.6, 163.1, 155.3, 146.3, 136.8, 135.6, 134.9, 131.05, 130.97, 130.4, 129.2, 128.7, 128.5, 128.1, 128.0, 122.1, 67.2, 61.0, 44.4, 42.4, 42.1, 35.9, 32.8, 14.4. **HRMS-ESI (m/z)**: $[M+H]^+$ calcd for $C_{28}H_{31}N_4O_5^+$, 503.2289, found 503.2285.

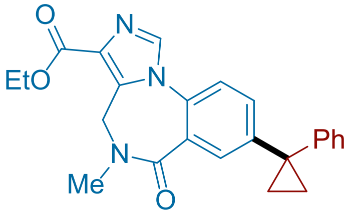

Ethyl 5-methyl-6-oxo-8-(1-phenylcyclopropyl)-5,6-dihydro-4H-benzo[f]imidazo[1,5-a][1,4]diazepine-3-carboxylate (4d)

General Procedure 2.2 was followed using $NiBr_2$(dme) (10 mol%) and dtbbpy (10 mol%) for the catalyst pre-stir, the informer bromide **X2** (36.4 mg, 0.1 mmol, 1 equiv), 1,3-dioxoisoindolin-2-yl-1-phenylcyclopropane-1-carboxylate (36.8 mg, 0.12 mmol, 1.2 equiv), Na_2CO_3 (21.2 mg, 0.2 mmol, 2 equiv) as the additive, and TMS-DHP (45.3 mg, 0.2 mmol, 2 equiv) as the reductant in DMA (0.4 mL) for 12 h at 80 °C. **4d** (36.1 mg, 0.09 mmol, 90%) was isolated as a white solid following reverse phase chromatography (water/acetonitrile mixture, gradient of 5% CH_3CN/H_2O–65% CH_3CN in H_2O). **^{1}H NMR (500 MHz, $CDCl_3$)** δ 7.86 (d, J = 2.2 Hz, 1H), 7.79 (s, 1H), 7.39 (dd, J = 8.3, 2.2 Hz, 1H), 7.29–7.23 (m, 5H), 7.20–7.17 (m, 1H), 5.12 (s, 1H), 4.40–4.31 (m, 3H), 3.19 (s, 3H), 1.40 (t, J = 7.2 Hz, 3H), 1.30–1.25 (m, 4H). **^{13}C{^{1}H} NMR (126 MHz, $CDCl_3$)** δ 166.7, 163.2, 147.1, 144.2, 135.7, 135.0, 132.8, 131.5, 129.9, 129.1, 129.0, 128.7, 126.8, 121.8, 61.0, 42.5, 35.9, 29.9, 16.5, 14.5. **HRMS-ESI (m/z)**: $[M+H]^+$ calcd for $C_{24}H_{24}N_3O_3^+$, 402.1812, found 402.1808.

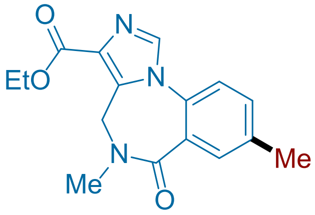

Ethyl 5,8-dimethyl-6-oxo-5,6-dihydro-4H-benzo[f]imidazo[1,5-a][1,4]diazepine-3-carboxylate (4e)

General Procedure 2.2 was followed using $NiBr_2$(dme) (10 mol%), a mixture of 1-bpp (5 mol%) and dtbbpy (5 mol%) as the ligand instead of only dtbbpy, the informer bromide **X2** (36.4 mg, 0.1 mmol, 1 equiv), 1,3-dioxoisoindolin-2-yl acetate (61.4 mg, 0.20 mmol, 2.0 equiv), Na_2CO_3 (21.2 mg, 0.2 mmol, 2 equiv) as the additive, and TMS-DHP (45.3 mg, 0.2 mmol, 2 equiv) as the reductant in DMA (0.4 mL) for 12 h at 80 °C. **4e** (20.1 mg, 0.067 mmol, 67%) was isolated as a white solid following reverse phase chromatography (gradient from 5%–50% CH_3CN/H_2O). **^{1}H NMR (400 MHz, $CDCl_3$)** δ 7.79 (s, 2H), 7.36 (dd, J = 8.2, 2.1 Hz, 1H), 7.24 (d, J = 8.2 Hz, 1H), 5.10 (s, 1H), 4.36–4.29 (m, 3H), 3.17 (s, 3H), 2.39 (s, 3H), 1.37 (t, J = 7.1 Hz, 3H). **^{13}C{^{1}H} NMR (101 MHz, $CDCl_3$)** δ 166.8, 163.2, 139.0, 135.7, 135.0, 133.5, 132.9, 129.8, 128.9, 128.7, 121.8, 61.0, 42.5, 35.9, 21.1, 14.5. **HRMS-ESI (m/z)**: $[M+H]^+$ calcd for $C_{16}H_{18}N_3O_3^+$, 300.1342, found 300.1340. Characterization data matched those reported in the literature.[48]

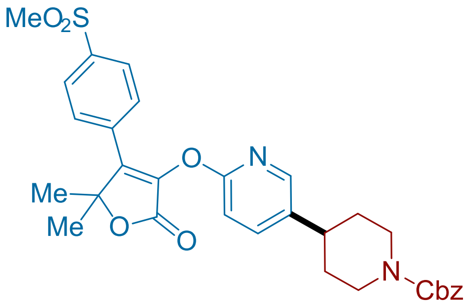

Benzyl 4-(6-((5,5-dimethyl-4-(4-(methylsulfonyl)phenyl)-2-oxo-2,5-dihydrofuran-3-yl)oxy)pyridin-3-yl)piperidine-1-carboxylate (**4f**)

General Procedure 2.2 was followed using $NiBr_2$(dme) (10 mol%) and dtbbpy (10 mol%) for the catalyst pre-stir, the informer bromide **X3** (21.9 mg, 0.05 mmol, 1 equiv), 1-benzyl 4-(1,3-dioxoisoindolin-2-yl)piperidine-1,4-dicarboxylate (24.5 mg, 0.06 mmol, 1.2 equiv), Na_2CO_3 (10.6 mg, 0.1 mmol, 2 equiv) as the additive, and TMS-Me_4DHP (28.3 mg, 0.1 mmol, 2 equiv) as the reductant in toluene (0.2 mL) for 15 h at 80 °C. **4f** (11.5 mg, 0.020 mmol, 40%) was isolated as a pale yellow solid following reverse phase chromatography (gradient from from 5%–86% CH_3CN/H_2O). **^{1}H NMR (500 MHz, $CDCl_3$)** δ 7.92–7.89 (m, 3H), 7.69–7.68 (m, 2H), 7.46 (dd, J = 8.5, 2.5 Hz, 1H), 7.30–7.24 (m, 5H), 6.88 (d, J = 8.4 Hz, 1H), 5.08 (s, 2H), 4.26 (br s, 2H), 2.99 (s, 3H), 2.80 (br s, 2H), 2.59 (tt, J = 12.2, 3.6 Hz, 1H), 1.75 (d, J = 13.1 Hz, 2H), 1.69 (s, 6H), 1.56–1.49 (m, 2H). **^{13}C{^{1}H} NMR (126 MHz, $CDCl_3$)** δ 164.9, 159.0, 154.2, 147.2, 144.7, 140.4, 137.3, 137.0, 135.8, 135.7, 134.0, 128.0, 127.5, 127.04, 126.93, 126.89, 109.9, 83.3, 66.2, 43.40, 43.36, 38.4, 31.9, 25.4. **HRMS-ESI (m/z)**: $[M+Na]^+$ calcd for $C_{31}H_{32}N_2O_7SNa^+$, 599.1822, found 599.1826.

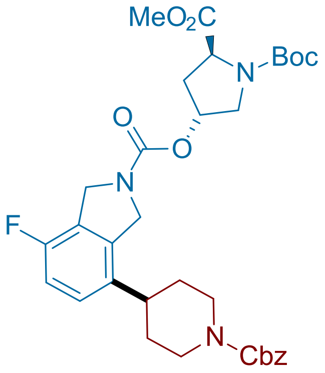

1-(*tert*-butyl) 2-methyl (2S,4R)-4-((4-(1-((benzyloxy)carbonyl)piperidin-4-yl)-7-fluoroisoindoline-2-carbonyl)oxy)pyrrolidine-1,2-dicarboxylate (**4g**)

General Procedure 2.2 was followed using $NiBr_2$(dme) (10 mol%) and dtbbpy (10 mol%) for the catalyst pre-stir, the informer bromide **X4** (24.4 mg, 0.05 mmol, 1 equiv), 1-benzyl 4-(1,3-dioxoisoindolin-2-yl)piperidine-1,4-dicarboxylate (24.5 mg, 0.06 mmol, 1.2 equiv), Na_2CO_3 (10.6 mg, 0.1 mmol, 2 equiv) as the additive, and TMS-Me_4DHP (28.3 mg, 0.1 mmol, 2 equiv) as the reductant in toluene (0.2 mL) for 15 h at 80 °C. The yield of **4g** was determined from ^{1}H NMR of the unpurified material (1,3,5-trimethoxybenzene as the internal standard, 6.3 mg, 0.0375 mmol, 0.75 equiv with respect to **X4**) and was found to be 78%. **^{1}H NMR (500 MHz, $CDCl_3$)** δ 7.39–7.33 (m, 5H), 7.09–7.05 (m, 1H), 6.97–6.92 (m, 1H), 5.33 (s, 1H), 5.17–5.15 (m, 2H), 4.77–4.76 (m, 2H), 4.70–4.66 (m, 2H), 4.53–4.34 (m, 3H), 3.96–3.75 (m, 5H, overlaps with the 1,3,5-trimethoxybenzene peak), 3.67–3.45 (m, 1H), 2.95–2.86 (m, 2H), 2.57 (td, J = 10.2, 4.5 Hz, 1H),

2.27–2.21 (m, 1H), 1.74 (br s, 2H), 1.62 (br s, 2H), 1.46–1.43 (m, 9H). **^{13}C{^{1}H} NMR (126 MHz, CDCl$_3$)** δ 173.2, 173.0, 155.39, 155.36, 153.96, 153.86, 136.9, 136.1, 132.8, 130.4, 129.2, 128.3, 128.1, 128.0, 126.7, 123.5, 123.4, 114.9, 93.05, 80.7, 74.0, 73.3, 67.3, 58.2, 57.8, 52.3, 52.0, 51.5, 50.2, 49.7, 44.7, 44.6, 39.1, 37.1, 36.1, 32.5, 30.7, 28.5, 28.37, 28.4. Mixture of diastereomers was observed. **HRMS-ESI (m/z)**: $[M+NH_4]^+$ calcd for $C_{33}H_{44}FN_4O_8^+$, 643.3138, found 643.3142.

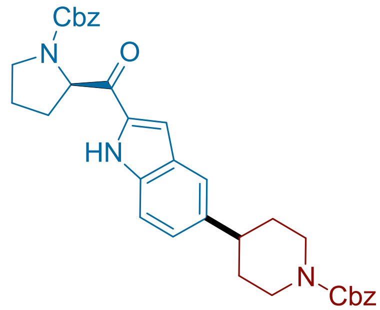

Benzyl 4-(2-(((benzyloxy)carbonyl)-D-prolyl)-1H-indol-5-yl)piperidine-1-carboxylate (4h)

General Procedure 2.2 was followed using $NiBr_2$(dme) (10 mol%) and dtbbpy (10 mol%) for the catalyst pre-stir, the informer bromide **X5** (21.4 mg, 0.05 mmol, 1 equiv), 1-benzyl 4-(5-methyl-1,3-dioxoisoindolin-2-yl) piperidine-1,4-dicarboxylate (NHP ester-3, 25.3 mg, 0.06 mmol, 1.2 equiv), Na_2CO_3 (10.6 mg, 0.1 mmol, 2 equiv) as the additive, and TMS-Me_4DHP (28.3 mg, 0.1 mmol, 2 equiv) as the reductant in toluene (0.2 mL) for 15 h at 80 °C. **4h** (23.1 mg, 0.041 mmol, 81%) was isolated as a pale yellow solid following reverse phase chromatography (gradient from 5% CH_3CN/H_2O to 75% CH_3CN/H_2O). **^{1}H NMR (500 MHz, CDCl$_3$)** δ 9.60–9.37 (m, 1H, mixture of rotamers), 8.27–8.09 (m, 1H, mixture of rotamers), 7.65 (d, J = 3.1 Hz, 1H), 7.40–7.37 (m, 5H), 7.36–7.34 (m, 1H), 7.33–7.29 (m, 2H), 7.17–7.09 (m, 1H), 7.07–7.03 (m, 1H), 6.97–6.94 (m, 1H), 5.25–5.13 (m, 3H), 5.11–5.08 (m, 1H), 5.01–4.95 (m, 1H), 4.33 (br s, 2H), 3.79–3.69 (m, 1H), 3.65–3.58 (m, 1H), 2.88 (br s, 2H), 2.84–2.67 (m, 1H), 2.30– 2.20 (m, 1H), 2.10–1.98 (m, 2H), 1.96–1.81 (m, 3H), 1.78–1.62 (m, 3H). **^{13}C{^{1}H} NMR (126 MHz, CDCl$_3$)** δ 194.5, 193.8, 155.35, 155.33, 155.2, 154.7, 140.4, 139.9, 136.94, 136.92, 136.8, 136.5, 135.1, 132.0, 131.3, 128.5, 128.03, 128.0, 127.95, 127.86, 127.7, 127.6, 126.5, 126.3, 125.9, 122.9, 122.7, 120.2, 119.7, 114.8, 114.7, 111.63, 111.56, 111.68, 67.10, 67.08, 67.0, 62.5, 62.3, 47.5, 47.0, 44.8, 42.9, 42.7, 33.8, 33.73, 33.69, 33.5, 31.9, 30.8, 24.3, 23.7. Mixture of diastereomers was observed. Products derived from this informer bromide (X5) have previously been reported to exist as a mixture of rotamers.[21] **HRMS-ESI (m/z)**: $[M+Na]^+$ calcd for $C_{34}H_{35}N_3O_5Na^+$, 588.2469, found 588.2463.

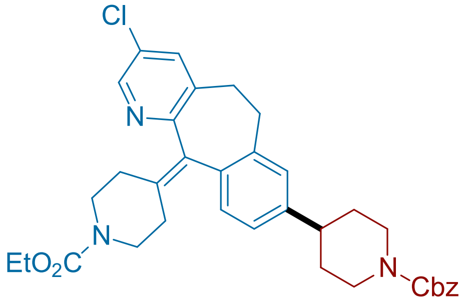

Benzyl 4-(3-chloro-11-(1-(ethoxycarbonyl)piperidin-4-ylidene)-6,11-dihydro-5H-benzo[5,6]cyclohepta[1,2-b]pyridin-8-yl)piperidine-1-carboxylate (4i)

General Procedure 2.2 was followed using $NiBr_2$(dme) (10 mol%) and dtbbpy (10 mol%) for the catalyst pre-stir, the informer bromide **X6** (46.2 mg, 0.10 mmol, 1 equiv), 1-benzyl 4-(1,3-

dioxoisoindolin-2-yl)piperidine-1,4-dicarboxylate (49.0 mg, 0.12 mmol, 1.2 equiv), Na_2CO_3 (21.2 mg, 0.2 mmol, 2 equiv) as the additive, and TMS-DHP (45.3 mg, 0.2 mmol, 2 equiv) as the reductant in DMA (0.2 mL) for 15 h at 80 °C. **4i** (43.4 mg, 0.072 mmol, 72%) was isolated as a white solid following column chromatography (gradient from 100% hexanes to 100% EtOAc/hexanes). **^{1}H NMR (500 MHz, $CDCl_3$)** δ 8.17 (d, J = 2.2 Hz, 1H), 7.31–7.23 (m, 5H), 7.16 (d, J = 2.2 Hz, 1H), 7.10 (d, J = 2.1 Hz, 1H), 7.07–7.02 (m, 2H), 5.08 (s, 2H), 4.26 (br s, 2H), 4.06 (q, J = 7.1 Hz, 2H), 3.77–3.70 (m, 2H), 3.35–3.21 (m, 2H), 3.08–3.03 (m, 2H), 2.80–2.74 (m, 3H), 2.73–2.68 (m, 1H), 2.58 (tt, J = 11.8, 4.1 Hz, 1H), 2.44–2.39 (m, 1H), 2.30–2.21 (m, 3H), 1.77–1.73 (m, 2H), 1.59–1.54 (m, 2H), 1.18 (t, J = 7.1 Hz, 3H). **^{13}C{^{1}H} NMR (126 MHz, $CDCl_3$)** δ 154.5, 154.2, 154.1, 144.5, 138.6, 138.2, 137.0, 136.6, 135.8, 134.7, 132.9, 132.0, 131.9, 129.4, 127.9, 127.5, 127.0, 126.9, 125.2, 66.1, 60.3, 43.8, 43.4, 38.7, 31.8, 30.7, 30.6, 29.5, 13.7. **HRMS-ESI (m/z)**: $[M+H]^+$ calcd for $C_{35}H_{39}ClN_3O_4^+$, 600.2624, found 600.2620.

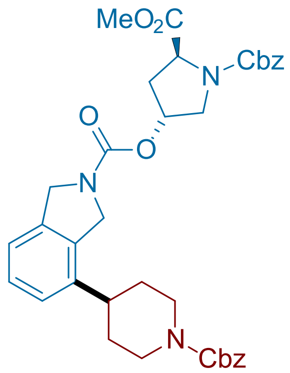

1-Benzyl 2-methyl (2S,4R)-4-((4-(1-((benzyloxy)carbonyl)piperidin-4-yl)isoindoline-2-carbonyl)oxy)pyrrolidine-1,2-dicarboxylate (4k)

General Procedure 2.2 was followed using $NiBr_2$(dme) (10 mol%) and dtbbpy (10 mol%) for the catalyst pre-stir, the informer bromide **X8** (25.2 mg, 0.05 mmol, 1 equiv), 1-benzyl 4-(1,3-dioxoisoindolin-2-yl)piperidine-1,4-dicarboxylate (24.5 mg, 0.06 mmol, 1.2 equiv), Na_2CO_3 (10.6 mg, 0.1 mmol, 2 equiv) as the additive, and TMS-Me_4DHP (28.3 mg, 0.1 mmol, 2 equiv) as the reductant in toluene (0.2 mL) for 15 h at 80 °C. **4k** (21.2 mg, 0.033 mmol, 66%) was isolated as a white solid following reverse phase chromatography (water/acetonitrile mixture, gradient of 5% CH_3CN/H_2O - 98% CH_3CN in H_2O). **^{1}H NMR (500 MHz, $CDCl_3$)** δ 7.44–7.30 (m, 11H), 7.17–7.11 (m, 2H), 5.39 (s, 1H), 5.29–5.07 (m, 4H), 4.77 (s, 2H), 4.68–4.60 (m, 2H), 4.58–4.50 (m, 1H), 4.39 (s, 2H), 3.94–3.84 (m, 1H), 3.81 (d, J = 3.5 Hz, 2H), 3.60 (s, 1H), 2.99–2.91 (m, 2H), 2.67–2.50 (m, 2H), 2.37–2.26 (m, 1H), 1.82–1.80 (m, 2H), 1.70 (br s, 3H). **^{13}C{^{1}H} NMR (126 MHz, $CDCl_3$)** δ 172.8, 172.6, 172.6, 155.42, 155.38, 155.1, 155.0, 154.4, 154.1, 154.0, 140.4, 137.0, 136.96, 136.91, 136.6, 136.5, 136.4, 134.5, 134.35, 134.30, 128.64, 128.59, 128.3, 128.2, 128.14, 128.12, 128.07, 128.05, 124.5, 120.8, 120.7, 73.8, 73.7 (73.78, 73.71), 73.1, 73.0, 67.5 (67.59, 67.53, 67.48), 67.3, 58.3, 58.2, 58.0, 53.1 (53.15, 53.08), 52.8, 52.6, 52.5, 52.4, 51.7, 44.7, 44.6, 40.0, 39.6, 37.3, 37.2, 36.2, 32.2, 29.8. Mixture of rotamers was observed. Products derived from this informer bromide (X5) have previously been reported to exist as a mixture of rotamers.[21, 33] **HRMS-ESI (m/z)**: $[M+H]^+$ calcd for $C_{36}H_{40}N_3O_8^+$, 642.2810, found 642.2802.

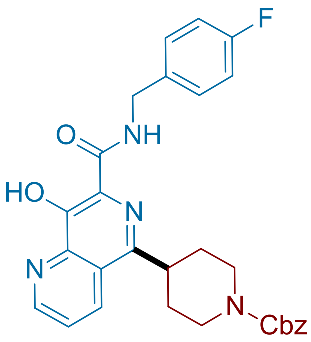

Benzyl 4-(7-((4-fluorobenzyl)carbamoyl)-8-hydroxy-1,6-naphthyridin-5-yl)piperidine-1-carboxylate (4m)

General Procedure 2.2 was slightly modifying for synthesizing **4m**. An oven dried 1-dram vial was charged with a PTFE-coated stir bar, informer bromide **X10** (37.6 mg, 0.10 mmol, 1 equiv), *N*,*O*-bis(trimethylsilyl)acetamide (BSA, 48.9 µL, 40.7 mg, 0.2 mmol, 2 equiv) and toluene (100 µL). The reaction mixture was stirred at 40 °C for 30 minutes inside the glovebox. Subsequently, 1-benzyl-4-(1,3-dioxoisoindolin-2-yl)piperidine-1,4-dicarboxylate (49.0 mg, 0.12 mmol, 1.2 equiv), Na_2CO_3 (21.2 mg, 0.2 mmol, 2 equiv) and the $NiBr_2$(dme)/dtbbpy pre-stir (10 mol%, in 200 µL toluene) were added, followed by TMS-Me_4DHP (56.5 mg, 0.2 mmol, 2 equiv) and additional toluene (100 µL). The reaction mixture was sealed with a PTFE-faced silicone septa and taken outside the glovebox where it was allowed to stir (1250 RPM) at 80 °C heated for 15 h. **4m** (17.5 mg, 0.034 mmol, 34% yield) was isolated as a pale yellow solid following reverse phase chromatography (water/acetonitrile mixture, gradient of 5% CH_3CN/H_2O–75% CH_3CN in H_2O, both containing 0.1% TFA). Product **4m** contained unknown impurities that were inseparable. Therefore, the yield of **4m** was determined by quantitative ^{1}H NMR (with respect to 1,3,5-trimethoxybenzene as an internal standard) and was found to be 34% yield, with 84% purity. **^{1}H NMR (500 MHz, $CDCl_3$)** δ 13.03 (s, 1H), 9.10 (dd, *J* = 4.2, 1.6 Hz, 1H), 8.39 (dd, *J* = 8.7, 1.6 Hz, 1H), 8.29 (t, *J* = 6.5 Hz, 1H), 7.57 (dd, *J* = 8.6, 4.2 Hz, 1H), 7.40–7.33–7.28 (m, 6H), 7.01–6.97 (m, 2H), 5.09 (s, 2H), 4.63 (d, *J* = 6.4 Hz, 2H), 4.38–4.32 (m, 2H), 3.48–3.42 (m, 1H), 2.95 (br s, 2H), 2.01–1.88 (m, 4H). **$^{13}C\{^1H\}$ NMR (126 MHz, $CDCl_3$)** δ 169.7, 163.4, 161.5, 155.4, 153.5 (d, $J_{C\text{-}F}$ = 3.3 Hz), 152.7, 143.7, 136.8, 133.8 (d, $J_{C\text{-}F}$ = 3.2 Hz), 132.7, 129.5 (d, $J_{C\text{-}F}$ = 8.2 Hz), 128.7, 128.2, 128.1, 124.5 (d, $J_{C\text{-}F}$ = 30.4 Hz), 124.1, 115.8 (d, $J_{C\text{-}F}$ = 21.5 Hz), 67.4, 44.3, 42.4, 38.9, 31.5. **$^{19}F\{^1H\}$ NMR** (377 MHz, $CDCl_3$) δ –114.74. **HRMS-ESI (m/z)**: $[M+H]^+$ calcd for $C_{29}H_{28}FN_4O_4^+$, 515.2089, found 515.2082.

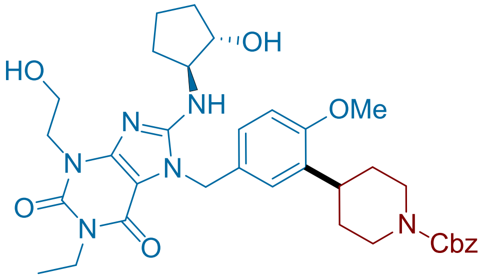

Benzyl 4-(5-((1-ethyl-8-(((1S,2S)-2-hydroxycyclopentyl)amino)-3-(2-hydroxyethyl)-2,6-dioxo-1,2,3,6-tetrahydro-7H-purin-7-yl)methyl)-2-methoxyphenyl)piperidine-1-carboxylate (4o)

General Procedure 2.2 was followed using using $NiBr_2$(dme) (10 mol%) and dtbbpy (10 mol%) for the catalyst pre-stir, the informer bromide **X12** (26.1 mg, 0.05 mmol, 1 equiv), 1-benzyl

4-(1,3-dioxoisoindolin-2-yl)piperidine-1,4-dicarboxylate (24.5 mg, 0.06 mmol, 1.2 equiv), DIPEA (17.4 µL mg, 12.9 mg, 0.1 mmol, 2 equiv) as the additive, and TMS-Me_4DHP (28.3 mg, 0.1 mmol, 2 equiv) as the reductant in toluene (0.2 mL) for 15 h at 80 °C. **4o** (22.5 mg, 0.034 mmol, 68%) was isolated as a white solid following reverse phase chromatography (water/acetonitrile mixture, gradient of 5%–63% CH_3CN in H_2O). **^{1}H NMR (500 MHz, $CDCl_3$)** δ 7.31–7.23 (m, 5H), 7.04–7.02 (m, 2H), 6.77 (d, *J* = 8.2 Hz, 1H), 5.19 (s, 2H), 5.07 (s, 2H), 4.24 (br s, 2H), 4.18– 4.15 (m, 2H), 4.14 (d, *J* = 4.4 Hz, 1H), 4.00 (q, *J* = 7.0 Hz, 2H), 3.86 (t, *J* = 5.0 Hz, 2H), 3.82–3.78 (m, 1H), 3.75 (s, 3H), 3.66 (septet, *J* = 4.6 Hz, 1H), 3.03 (tt, *J* = 12.1, 3.4 Hz, 1H), 2.88–2.81 (m, 2H), 2.07–2.00 (m, 1H), 1.95–1.87 (m, 1H), 1.72–1.65 (m, 4H), 1.60–1.47 (m, 4H), 1.24–1.21 (m, 1H), 1.18 (t, *J* = 7.0 Hz, 3H). **^{13}C{^{1}H} NMR (126 MHz, $CDCl_3$)** δ 157.0, 155.5, 154.0, 153.2, 152.0, 147.5, 137.0, 134.9, 128.6, 128.1, 128.0, 126.9, 126.6, 126.2, 111.2, 103.4, 79.6, 67.2, 63.1, 61.8, 55.6, 47.1, 46.3, 44.9, 36.6, 35.5, 33.0, 31.7, 29.8, 21.4, 13.5. **HRMS-ESI (m/z)**: $[M+H]^+$ calcd for $C_{35}H_{45}N_6O_7^+$, 661.3344, found 661.3343.

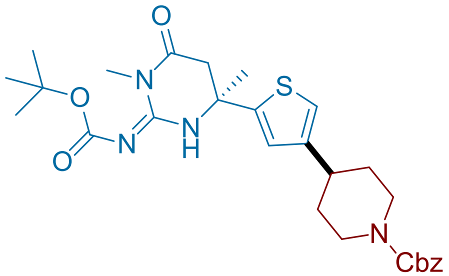

Benzyl (S,Z)-4-(5-(2-((tert-butoxycarbonyl)imino)-1,4-dimethyl-6-oxohexahydropyrimidin-4-yl)thiophen-3-yl)piperidine-1-carboxylate (4p)

General Procedure 2.2 was followed with a slight modification for synthesizing **4p**. The informer bromide **X12** (40.2 mg, 0.10 mmol, 1 equiv), 1-benzyl 4-(1,3-dioxoisoindolin-2-yl)piperidine-1,4-dicarboxylate (49.0 mg, 0.12 mmol, 1.2 equiv), Na_2CO_3 (21.2 mg, 0.2 mmol, 2 equiv) and $NiBr_2$(dme)/dtbbpy pre-stir (0.01 mmol, 10 mol% in 400 µL toluene) were added sequentially followed by TMS-Me_4DHP (56.5 mg, 0.2 mmol, 2 equiv). The reaction mixture was heated at 80 °C for 15 h. After that, the reaction vial was taken back inside the glove box and allowed cool under the nitrogen atmosphere. Then *N*,*N*-Diisopropylethylamine (26.2 µL, 19.4 mg, 0.15 mmol, 1.5 equiv) was added to the reaction mixture followed by di-*tert*-butyl dicarbonate (230.0 µL, 218.3 mg, 1.0 mmol, 10.0 equiv). Then the vial was sealed with a screw cap, taken outside the glove box and allowed to stir at 40 °C for 12 h. This step was done to reprotect any guanidine nitrogens that had lost their -Boc group during the coupling reaction. Ultimately **4p** (22.6 mg, 0.042 mmol, 42%) was isolated as an off-white solid following reverse phase chromatography (water/acetonitrile mixture, gradient of 5% CH_3CN/H_2O–98% CH_3CN in H_2O). The product had 16% of the -Boc deprotected product (benzyl (S)-4-(5-(2-imino-1,4-dimethyl-6-oxohexahydropyrimidin-4-yl)thiophen-3-yl)piperidine-1-carboxylate), that was inseparable. **^{1}H NMR (500 MHz, $CDCl_3$)** δ 10.3 (br s, 1H), 7.37–7.31 (m, 5H), 6.84 (m, 1H), 6.76 (s, 1H), 5.14 (s, 2H), 4.27 (br s, 2H), 3.26 (m, 3H), 3.13–3.09 (m, 1H), 2.93–2.85 (m, 3H), 2.68 (tt, *J* = 11.9, 4.0 Hz, 1H), 1.92–1.87 (m, 2H), 1.78–1.65(m, 5H), 1.53 (s, 9H). **^{13}C{^{1}H} NMR (126 MHz, $CDCl_3$)** δ 167.5, 164.0, 157.6, 155.4, 148.2, 146.7, 137.0, 128.6, 128.0, 123.5, 119.0, 80.1, 67.2, 53.4, 46.0, 44.4, 37.9, 32.8, 30.2, 28.7, 28.4. **HRMS-ESI (m/z)**: $[M+H]^+$ calcd for $C_{28}H_{37}N_4O_5S^+$, 541.2479, found 541.2466.

7. References

(1) Schirmer, T. E.; Wimmer, A.; Weinzierl, F. W. C.; König, B. Photo–nickel dual catalytic benzoylation of aryl bromides. *Chem Commun* **2019**, *55*, 10796–10799, 10.1039/C9CC04726C.
(2) Everson, D. A.; Jones, B. A.; Weix, D. J. Replacing Conventional Carbon Nucleophiles with Electrophiles: Nickel-Catalyzed Reductive Alkylation of Aryl Bromides and Chlorides. *J. Am. Chem. Soc.* **2012**, *134*, 6146–6159.
(3) Patel, P.; Borah, G. Synthesis of oxindole from acetanilide via Ir(III)-catalyzed C–H carbenoid functionalization. *Chem Commun* **2017**, *53*, 443–446, 10.1039/C6CC08788D.
(4) Shim, Y. S.; Kim, K. C.; Chi, D. Y.; Lee, K.-H.; Cho, H. Formylchromone derivatives as a novel class of protein tyrosine phosphatase 1B inhibitors. *Bioorg. Med. Chem. Lett.* **2003**, *13*, 2561–2563.
(5) Zeng, L.-F.; Wang, Y.; Kazemi, R.; Xu, S.; Xu, Z.-L.; Sanchez, T. W.; Yang, L.-M.; Debnath, B.; Odde, S.; Xie, H.; et al. Repositioning HIV-1 Integrase Inhibitors for Cancer Therapeutics: 1,6-Naphthyridine-7-carboxamide as a Promising Scaffold with Drug-like Properties. *J. Med. Chem.* **2012**, *55*, 9492–9509.
(6) Salgueiro, D. C.; Chi, B. K.; Guzei, I. A.; García-Reynaga, P.; Weix, D. J. Control of Redox-Active Ester Reactivity Enables a General Cross-Electrophile Approach to Access Arylated Strained Rings. *Angew. Chem. Int. Ed.* **2022**, *61*, e202205673.
(7) Huihui, K. M. M.; Caputo, J. A.; Melchor, Z.; Olivares, A. M.; Spiewak, A. M.; Johnson, K. A.; DiBenedetto, T. A.; Kim, S.; Ackerman, L. K. G.; Weix, D. J. Decarboxylative Cross-Electrophile Coupling of N-Hydroxyphthalimide Esters with Aryl Iodides. *J. Am. Chem. Soc.* **2016**, *138*, 5016–5019.
(8) Chan, C.-M.; Xing, Q.; Chow, Y.-C.; Hung, S.-F.; Yu, W.-Y. Photoredox Decarboxylative C(sp^3)–N Coupling of α-Diazoacetates with Alkyl N-Hydroxyphthalimide Esters for Diversified Synthesis of Functionalized N-Alkyl Hydrazones. *Org. Lett.* **2019**, *21*, 8037–8043.
(9) Brals, J.; McGuire, T. M.; Watson, A. J. B. A Chemoselective Polarity-Mismatched Photocatalytic C(sp^3)−C(sp^2) Cross-Coupling Enabled by Synergistic Boron Activation. *Angew. Chem. Int. Ed.* **2023**, *62*, e202310462.
(10) Guerrero, I.; Tan, E. Y. K.; Liu, Y.; Edwards, L. J.; Chiba, S. Photoinduced Alkylation of Diazines with N-(Acyloxy)phthalimides in the Presence of Triethylamine. *Synthesis* **2023**, *56*, 3261–3276.
(11) Webb, E. W.; Park, J. B.; Cole, E. L.; Donnelly, D. J.; Bonacorsi, S. J.; Ewing, W. R.; Doyle, A. G. Nucleophilic (Radio)Fluorination of Redox-Active Esters via Radical-Polar Crossover Enabled by Photoredox Catalysis. *J. Am. Chem. Soc.* **2020**, *142*, 9493–9500.
(12) Liu, L.; Pan, N.; Sheng, W.; Su, L.; Liu, L.; Dong, J.; Zhou, Y.; Yin, S.-F. Visible Light-Induced Regioselective Decarboxylative Alkylation of the C(sp^2)−H Bonds of Non-Aromatic Heterocycles. *Adv. Synth. Catal.* **2019**, *361*, 4126–4132.
(13) Chen, J.-Q.; Huang, J.; Yao, J.; Wang, T.; Feng, Y.; Zhou, X.; Yang, M.; Wu, J. Photoinduced Concerted Fragmentation of Alkyl N-Phthalimidoyl Oxalates: Mechanisms and Applications. *Org. Lett.* **2025**, *27*, 427–432.
(14) Qin, T.; Malins, L. R.; Edwards, J. T.; Merchant, R. R.; Novak, A. J. E.; Zhong, J. Z.; Mills, R. B.; Yan, M.; Yuan, C.; Eastgate, M. D.; et al. Nickel-Catalyzed Barton Decarboxylation and Giese Reactions: A Practical Take on Classic Transforms. *Angew. Chem. Int. Ed.* **2017**, *56*, 260–265.

(15) Parsutkar, M. M.; Moore, C. E.; RajanBabu, T. V. Activator-free single-component Co(I)-catalysts for regio- and enantioselective heterodimerization and hydroacylation reactions of 1,3-dienes. New reduction procedures for synthesis of [L]Co(I)-complexes and comparison to in situ generated catalysts. *Dalton Trans.* **2022**, *51*, 10148–10159, 10.1039/D2DT01484J.
(16) Schröder, I.; Kolodziej, C. M.; Moreno, J. A.; Merlic, C. A. Lessons Learned─Explosion and Fires Resulting from Quenching Lithium, Lithium Nitride, and Sodium. *ACS Chem. Health Saf.* **2024**, *31*, 473–481.
(17) Waghamare, A. B.; Raut, R. K.; Patel, N.; Majumdar, M. Role of N, N′-Diboryl-4, 4′-bipyridinylidene in the Transition-Metal-Free Borylation of Aryl Halides and Direct C−H Arylation of Unactivated Benzene. *Eur. J. Inorg. Chem.* **2022**, *2022*, e202200089.
(18) Zhang, R.; Li, G.; Wismer, M.; Vachal, P.; Colletti, S. L.; Shi, Z.-C. Profiling and Application of Photoredox $C(sp^3)$–$C(sp^2)$ Cross-Coupling in Medicinal Chemistry. *ACS Med. Chem. Lett.* **2018**, *9*, 773–777.
(19) Prieto Kullmer, C. N.; Kautzky, J. A.; Krska, S. W.; Nowak, T.; Dreher, S. D.; MacMillan, D. W. C. Accelerating reaction generality and mechanistic insight through additive mapping. *Science* **2022**, *376*, 532–539.
(20) Souza, L. W.; Ricke, N. D.; Chaffin, B. C.; Fortunato, M. E.; Jiang, S.; Soylu, C.; Caya, T. C.; Lau, S. H.; Wieser, K. A.; Doyle, A. G.; et al. Applying Active Learning toward Building a Generalizable Model for Ni-Photoredox Cross-Electrophile Coupling of Aryl and Alkyl Bromides. *J. Am. Chem. Soc.* **2025**, *147*, 18747–18759.
(21) Dinh, L. P.; Starbuck, H. F.; Hamby, T. B.; LaLama, M. J.; He, C. Q.; Kalyani, D.; Sevov, C. S. Persistent organonickel complexes as general platforms for Csp^2–Csp^3 coupling reactions. *Nat. Chem.* **2024**, *16*, 1515–1522.
(22) Kutchukian, P. S.; Dropinski, J. F.; Dykstra, K. D.; Li, B.; DiRocco, D. A.; Streckfuss, E. C.; Campeau, L.-C.; Cernak, T.; Vachal, P.; Davies, I. W.; et al. Chemistry informer libraries: a chemoinformatics enabled approach to evaluate and advance synthetic methods. *Chem. Sci.* **2016**, *7*, 2604–2613, 10.1039/C5SC04751J.
(23) Greshock, T. J.; Moore, K. P.; McClain, R. T.; Bellomo, A.; Chung, C. K.; Dreher, S. D.; Kutchukian, P. S.; Peng, Z.; Davies, I. W.; Vachal, P.; et al. Synthesis of Complex Druglike Molecules by the Use of Highly Functionalized Bench-Stable Organozinc Reagents. *Angew. Chem. Int. Ed.* **2016**, *55*, 13714–13718.
(24) VanHeyst, M. D.; Qi, J.; Roecker, A. J.; Hughes, J. M. E.; Cheng, L.; Zhao, Z.; Yin, J. Continuous Flow-Enabled Synthesis of Bench-Stable Bicyclo[1.1.1]pentane Trifluoroborate Salts and Their Utilization in Metallaphotoredox Cross-Couplings. *Org. Lett.* **2020**, *22*, 1648–1654.
(25) Dong, Z.; MacMillan, D. W. C. Metallaphotoredox-enabled deoxygenative arylation of alcohols. *Nature* **2021**, *598*, 451–456.
(26) Turro, R. F.; Wahlman, J. L. H.; Tong, Z. J.; Chen, X.; Yang, M.; Chen, E. P.; Hong, X.; Hadt, R. G.; Houk, K. N.; Yang, Y.-F.; et al. Mechanistic Investigation of Ni-Catalyzed Reductive Cross-Coupling of Alkenyl and Benzyl Electrophiles. *J. Am. Chem. Soc.* **2023**, *145*, 14705–14715.
(27) Biswas, S.; Weix, D. J. Mechanism and Selectivity in Nickel-Catalyzed Cross-Electrophile Coupling of Aryl Halides with Alkyl Halides. *J. Am. Chem. Soc.* **2013**, *135*, 16192–16197.
(28) Tran, V. T.; Kim, N.; Rubel, C. Z.; Wu, X.; Kang, T.; Jankins, T. C.; Li, Z.-Q.; Joannou, M. V.; Ayers, S.; Gembicky, M.; et al. Structurally Diverse Bench-Stable Nickel(0) Pre-Catalysts: A Practical Toolkit for In Situ Ligation Protocols. *Angew. Chem. Int. Ed.* **2023**, *62*, e202211794.

(29) Huang, L.; Ackerman, L. K. G.; Kang, K.; Parsons, A. M.; Weix, D. J. LiCl-Accelerated Multimetallic Cross-Coupling of Aryl Chlorides with Aryl Triflates. *J. Am. Chem. Soc.* **2019**, *141*, 10978–10983.
(30) Akana-Schneider, B. D.; Mouat, J. M.; Zhang, S.; Akana, M. E.; Wu, B.; Weix, D. J. Translation of Nickel-Catalyzed C(sp^2)–C(sp^3) Cross-Electrophile Coupling to Non-Amide Solvents. *Org. Lett.* **2025**, *27*, 4310–4315.
(31) Hughes, J. M. E.; Fier, P. S. Desulfonylative Arylation of Redox-Active Alkyl Sulfones with Aryl Bromides. *Org. Lett.* **2019**, *21*, 5650–5654.
(32) Lin, E.; Wang, J. Z.; Mao, E.; Tsang, S.; Carsch, K. M.; Prieto Kullmer, C. N.; McNamee, R. E.; Long, J. R.; Le, C. C.; MacMillan, D. W. C. Aryl Acid-Alcohol Cross-Coupling: C(sp^3)–C(sp^2) Bond Formation from Nontraditional Precursors. *J. Am. Chem. Soc.* **2025**, *147*, 14905–14914.
(33) Charboneau, D. J.; Barth, E. L.; Hazari, N.; Uehling, M. R.; Zultanski, S. L. A Widely Applicable Dual Catalytic System for Cross-Electrophile Coupling Enabled by Mechanistic Studies. *ACS Catal.* **2020**, *10*, 12642–12656.
(34) Kim, S.; Goldfogel, M. J.; Ahern, B. N.; Salgueiro, D. C.; Guzei, I. A.; Weix, D. J. Nickel-Catalyzed Cross-Electrophile Coupling of Aryl Triflates with Alkyl Halides: Mechanism-Informed Design of More General Conditions. *J. Am. Chem. Soc.* **2025**, *147*, 2616–2625.
(35) Liu, J.-H.; Yang, C.-T.; Lu, X.-Y.; Zhang, Z.-Q.; Xu, L.; Cui, M.; Lu, X.; Xiao, B.; Fu, Y.; Liu, L. Copper-Catalyzed Reductive Cross-Coupling of Nonactivated Alkyl Tosylates and Mesylates with Alkyl and Aryl Bromides. *Chem. Eur. J.* **2014**, *20*, 15334–15338.
(36) Peter A; James, S. Bifunctional Compounds Capable of Degrading Androgen Receptors. US WO2024189488A1, **2024**.
(37) Yu, X.; Chen, K.; Wang, Q.; Zhang, W.; Zhu, J. Synthesis of 2,5-disubstituted oxazoles via cobalt(III)-catalyzed cross-coupling of N-pivaloyloxyamides and alkynes. *Chem. Commun.* **2018**, *54*, 1197–1200, 10.1039/C7CC08611C.
(38) Jiang, Q.; Zhao, A.; Xu, B.; Jia, J.; Liu, X.; Guo, C. PIFA-Mediated Esterification Reaction of Alkynes with Alcohols via Oxidative Cleavage of Carbon Triple Bonds. *J. Org. Chem.* **2014**, *79*, 2709–2715.
(39) Yang, K.; Lu, J.; Li, L.; Luo, S.; Fu, N. Electrophotochemical Metal-Catalyzed Decarboxylative Coupling of Aliphatic Carboxylic Acids. *Chem. Eur. J.* **2022**, *28*, e202202370.
(40) Nielsen, S. D.; Smith, G.; Begtrup, M.; Kristensen, J. L. Synthesis and Application of a New Fluorous-Tagged Ammonia Equivalent. *Chem. Eur. J.* **2010**, *16*, 4557–4566.
(41) Palkowitz, M. D.; Laudadio, G.; Kolb, S.; Choi, J.; Oderinde, M. S.; Ewing, T. E.-H.; Bolduc, P. N.; Chen, T.; Zhang, H.; Cheng, P. T. W.; et al. Overcoming Limitations in Decarboxylative Arylation via Ag–Ni Electrocatalysis. *J. Am. Chem. Soc.* **2022**, *144*, 17709–17720.
(42) Zhao, H.; Jose, A. T.; Asany, A.; Khan, S. M.; Biscoe, M. R. Pd-Catalyzed Arylation of Secondary α-Alkoxytricyclohexylstannanes. *Org. Lett.* **2022**, *24*, 8714–8718.
(43) Herath, A.; Molteni, V.; Pan, S.; Loren, J. Generation and Cross-Coupling of Organozinc Reagents in Flow. *Org. Lett.* **2018**, *20*, 7429–7432.
(44) Cardinale, L.; Beutner, G. L.; Bemis, C. Y.; Weix, D. J.; Stahl, S. S. Non-Innocent Role of Sacrificial Anodes in Electrochemical Nickel-Catalyzed C(sp^2)–C(sp^3) Cross-Electrophile Coupling. *J. Am. Chem. Soc.* **2024**, *146*, 32249–32254.
(45) Laudadio, G.; Neigenfind, P.; Péter, Á.; Rubel, C. Z.; Emmanuel, M. A.; Oderinde, M. S.; Ewing, T. E.-H.; Palkowitz, M. D.; Sloane, J. L.; Gillman, K. W.; et al. Nickel-Electrocatalytic Decarboxylative Arylation to Access Quaternary Centers. *Angew. Chem. Int. Ed.* **2024**, *63*, e202314617.

(46) Conboy, A.; Greaney, M. F. Synthesis of benzenes from pyridines via N to C switch. *Chem* **2024**, *10*, 1940–1949.
(47) Arndt, C. M.; Bitai, J.; Brunner, J.; Opatz, T.; Martinelli, P.; Gollner, A.; Sokol, K. R.; Krumb, M. One-Pot Synthesis of Cereblon Proteolysis Targeting Chimeras via Photoinduced C(sp^2)–C(sp^3) Cross Coupling and Amide Formation for Proteolysis Targeting Chimera Library Synthesis. *J. Med. Chem.* **2023**, *66*, 16939–16952.
(48) Liu, W.; Mulhearn, J.; Hao, B.; Cañellas, S.; Last, S.; Gómez, J. E.; Jones, A.; De Vera, A.; Kumar, K.; Rodríguez, R.; et al. Enabling Deoxygenative C(sp^2)-C(sp^3) Cross-Coupling for Parallel Medicinal Chemistry. *ACS Med. Chem. Lett.* **2023**, *14*, 853–859.

8. NMR Spectra

Figure S1. ^{1}H NMR (500 MHz) for *in-situ* Ni(II) Reduction with TMS-DHP in protic DMF (with solvent suppression)

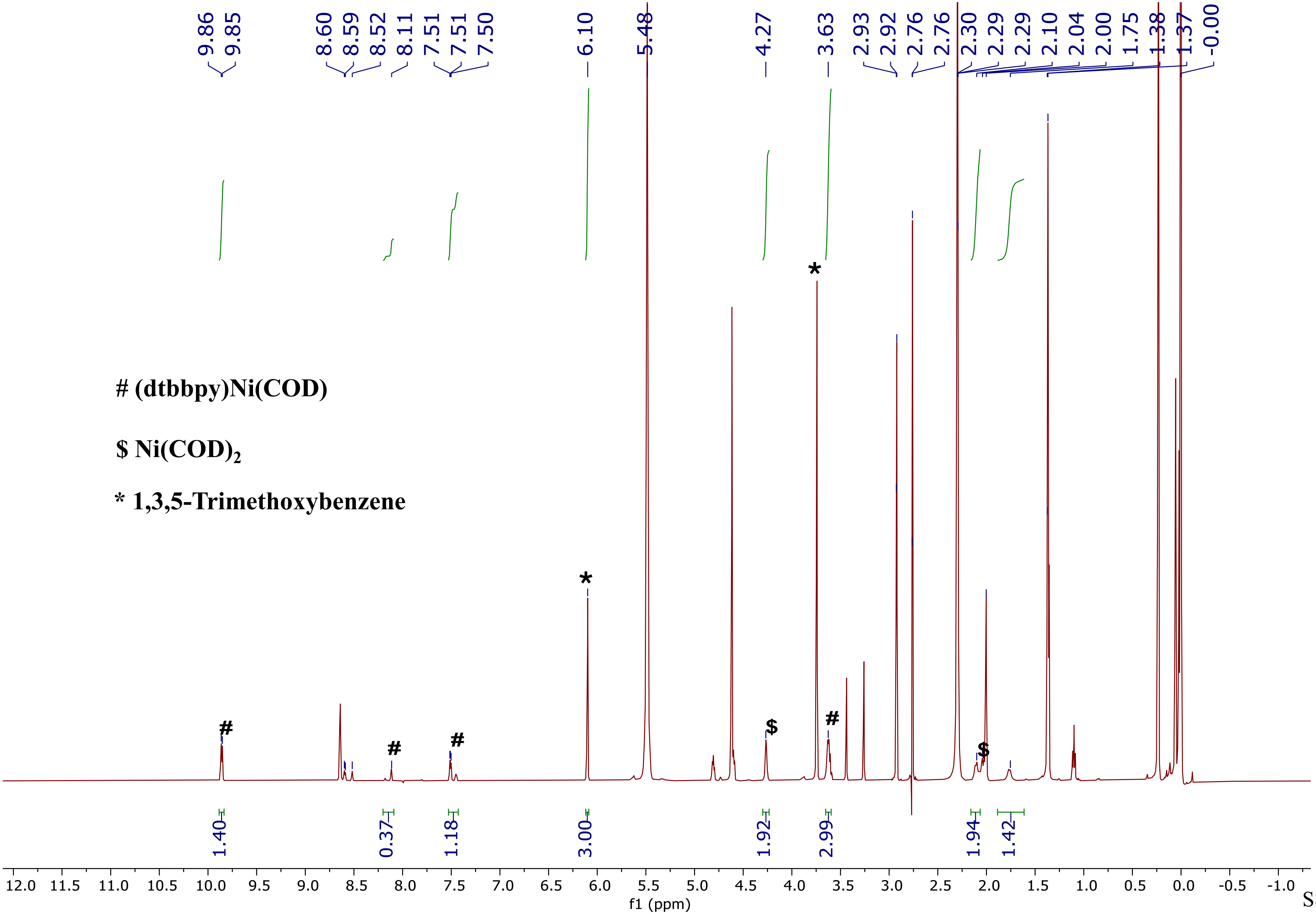

Figure S2. ^{1}H NMR (500 MHz) for *in-situ* Ni(II) Reduction with Zn in protic DMF (with solvent suppression)

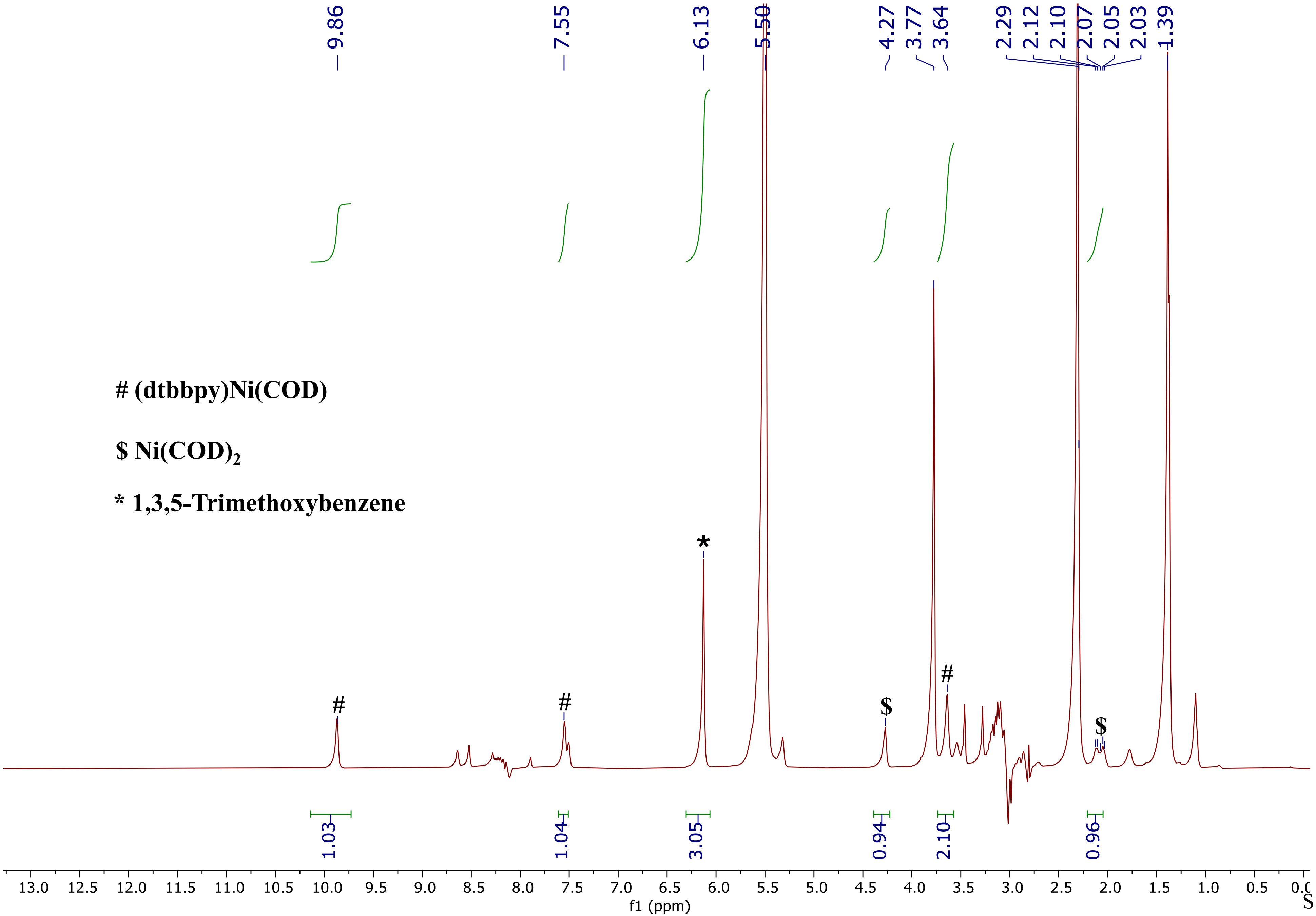

Figure S3. ^{1}H NMR (500 MHz, toluene-D_8) of the reaction mixture after reduction of Ni(II) with TMS-Me_4DHP in the presence of 2-Iodocumene.

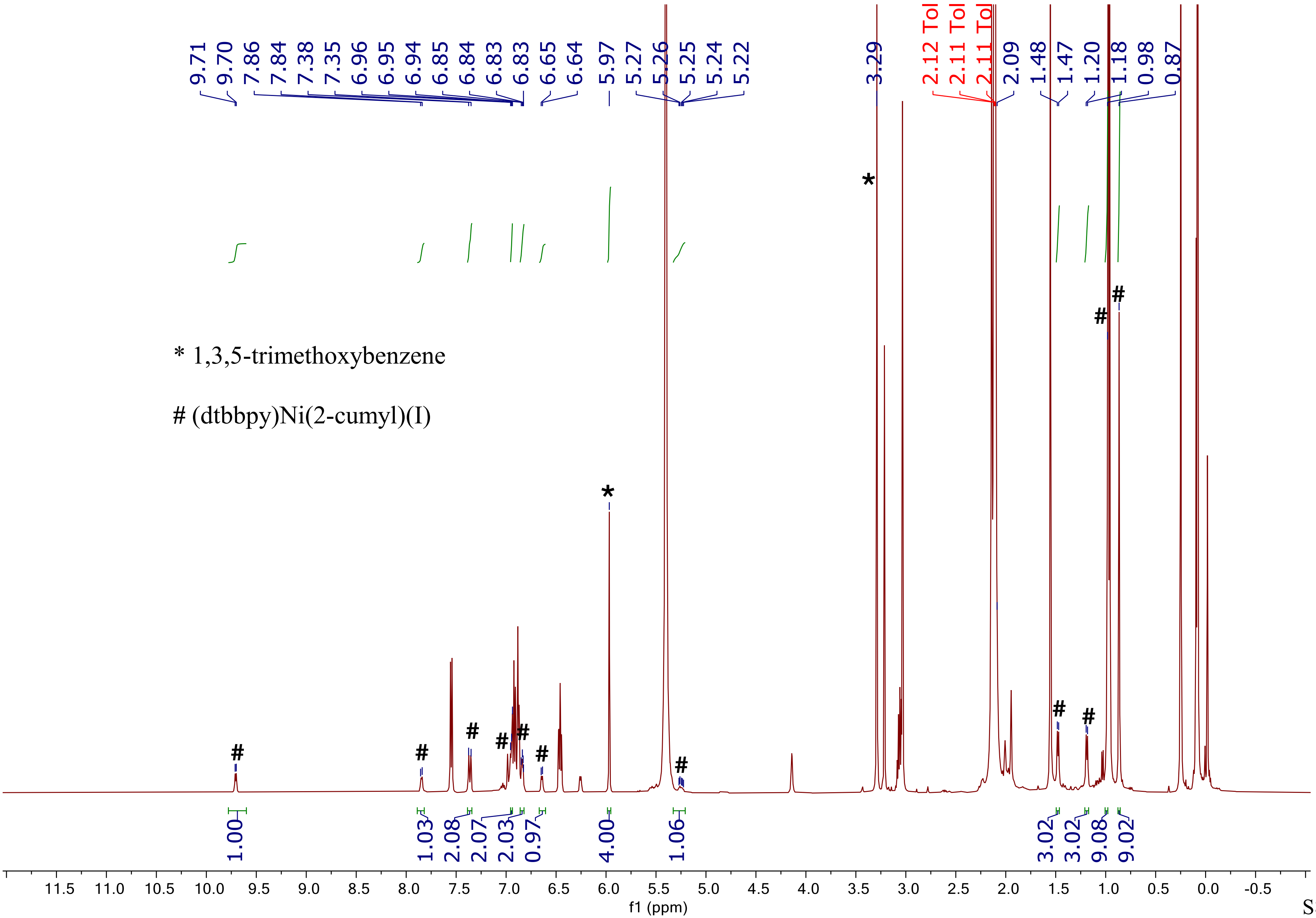

Figure S4. Quantitative ^{1}H NMR (500 MHz, toluene-D_8) of the isolated (dtbbpy)Ni(2-cumenyl)(I) complex.

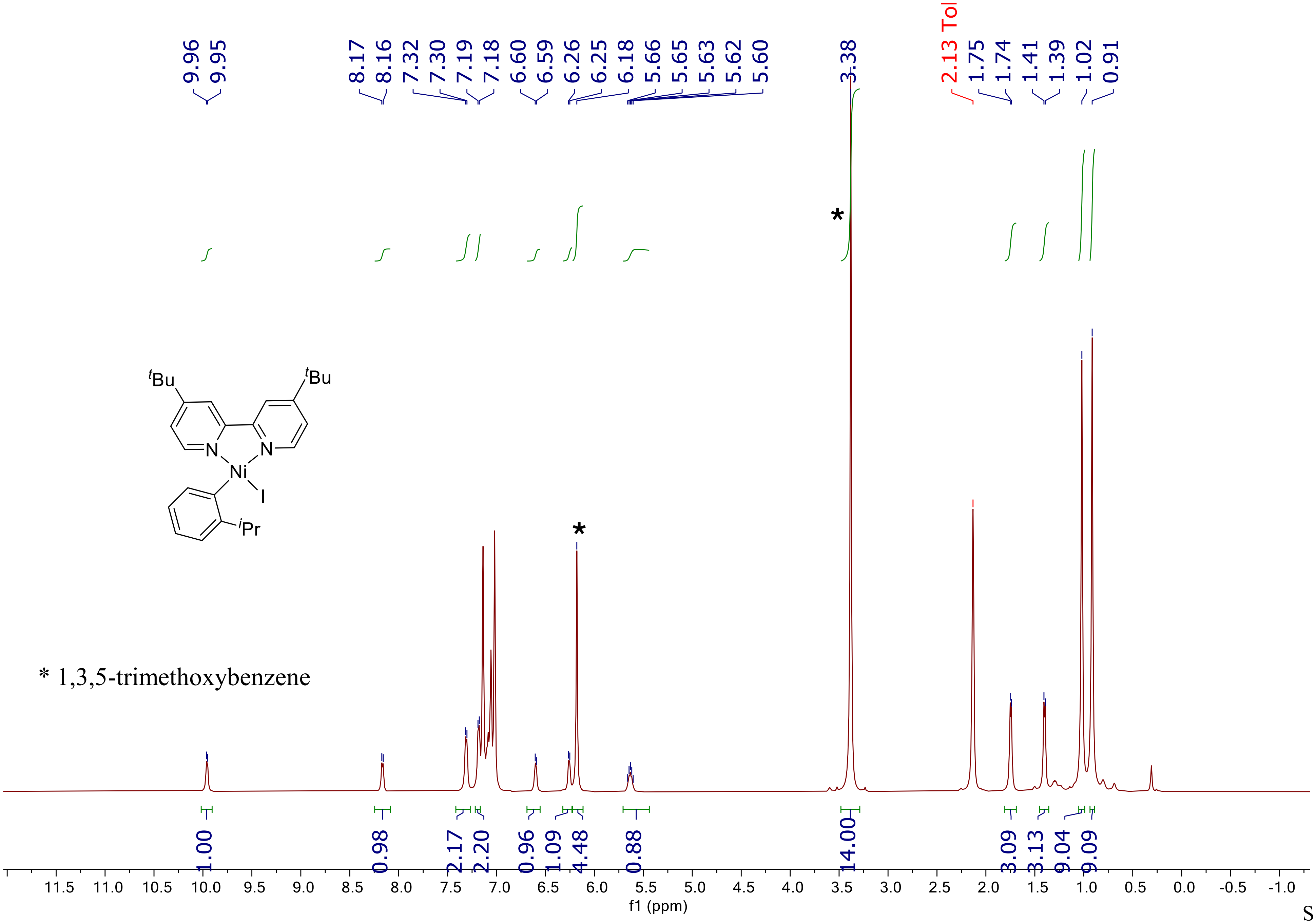

^{1}H NMR (500 MHz, toluene-d_8) of the in-situ (above) versus isolated (below) complex (dtbbpy)Ni(2-cumenyl)(I).

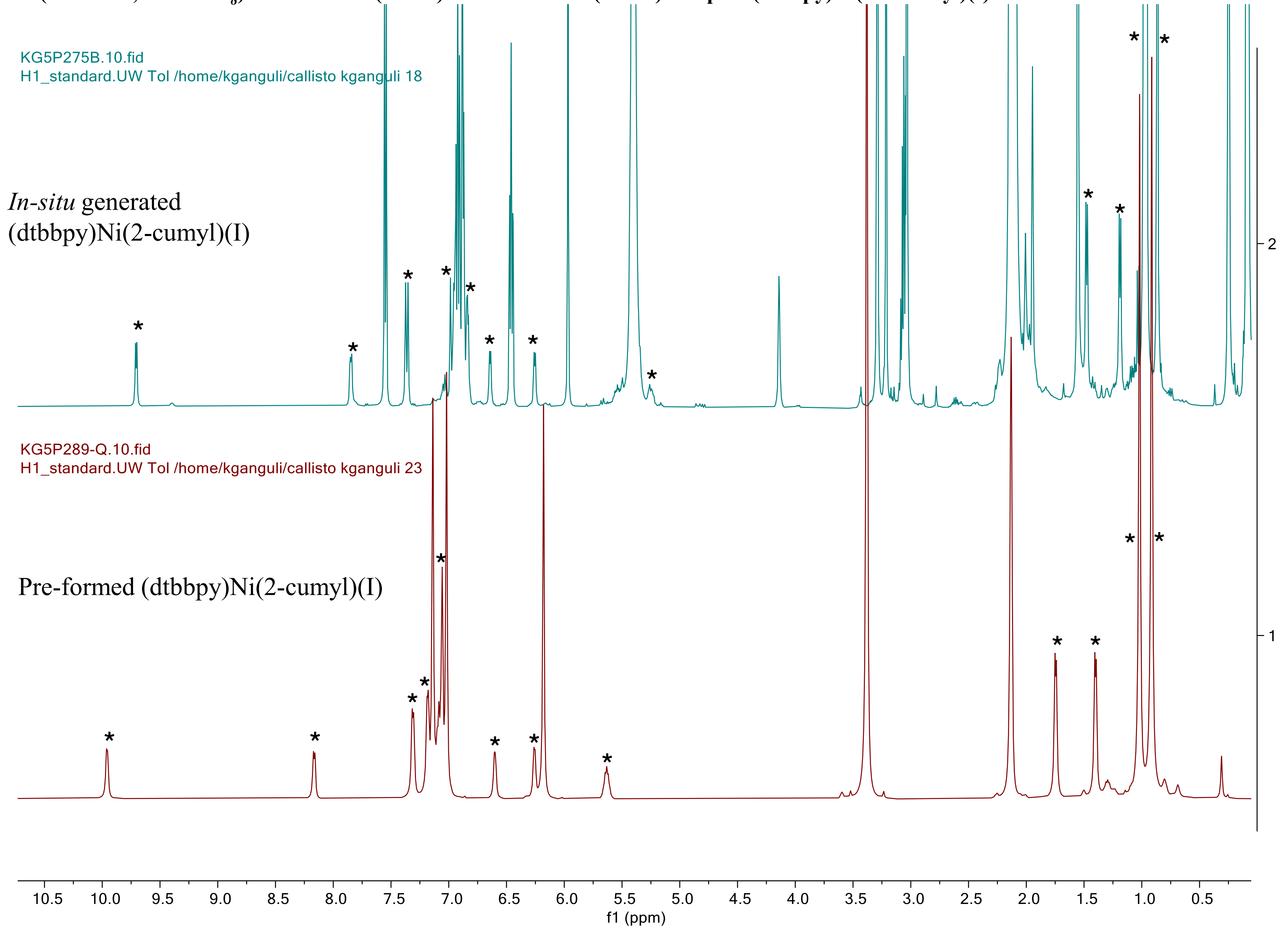

Figure S5. ^{1}H NMR (500 MHz, $CDCl_3$) of the isolated [(cod)Ni(cpd)] complex.

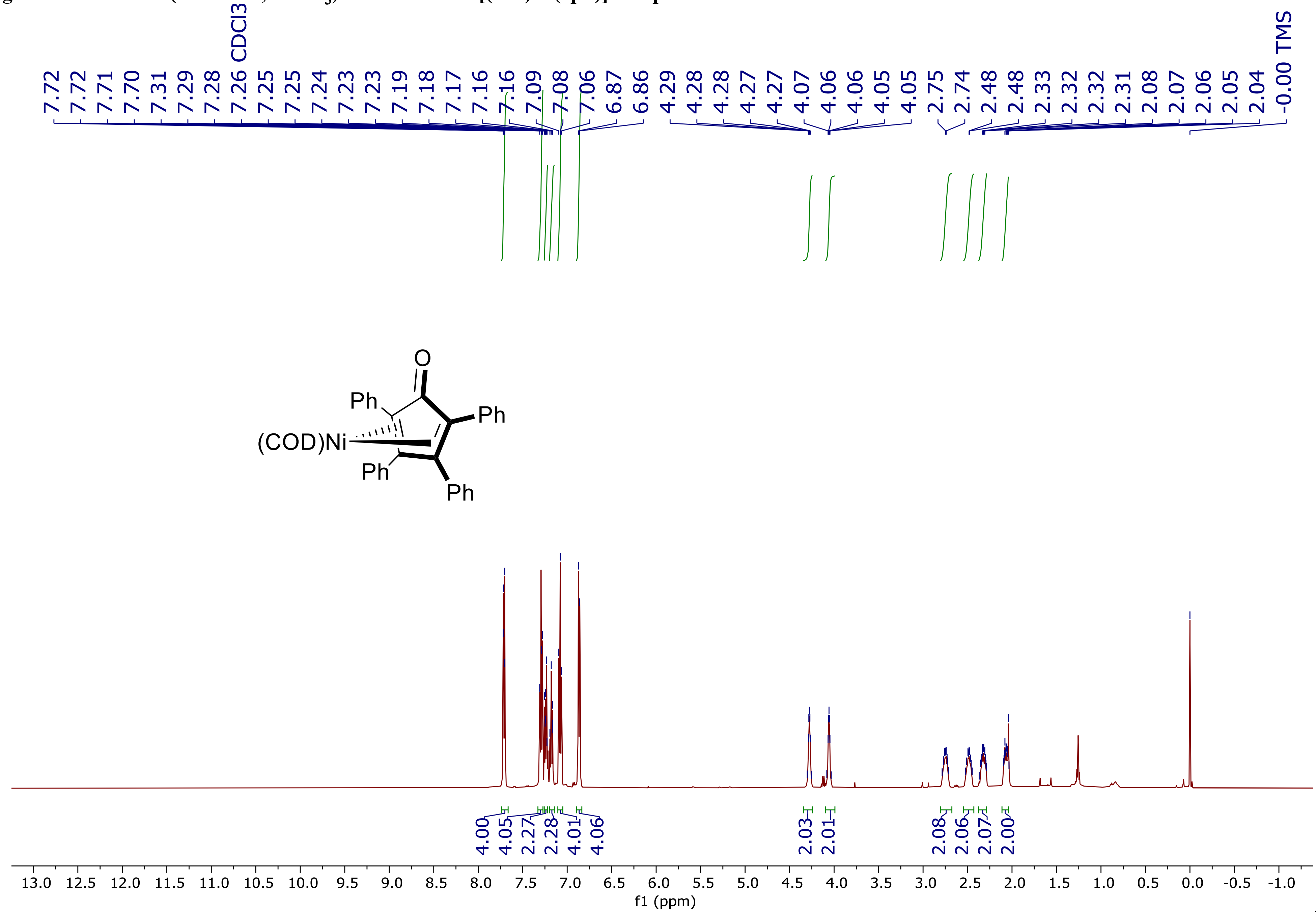

Figure S6. ^{1}H NMR (500 MHz, toluene-d_8) of the pure substrate 2a (above) versus reaction mixture of 2a + TMS-DHP + Na_2CO_3 (below).

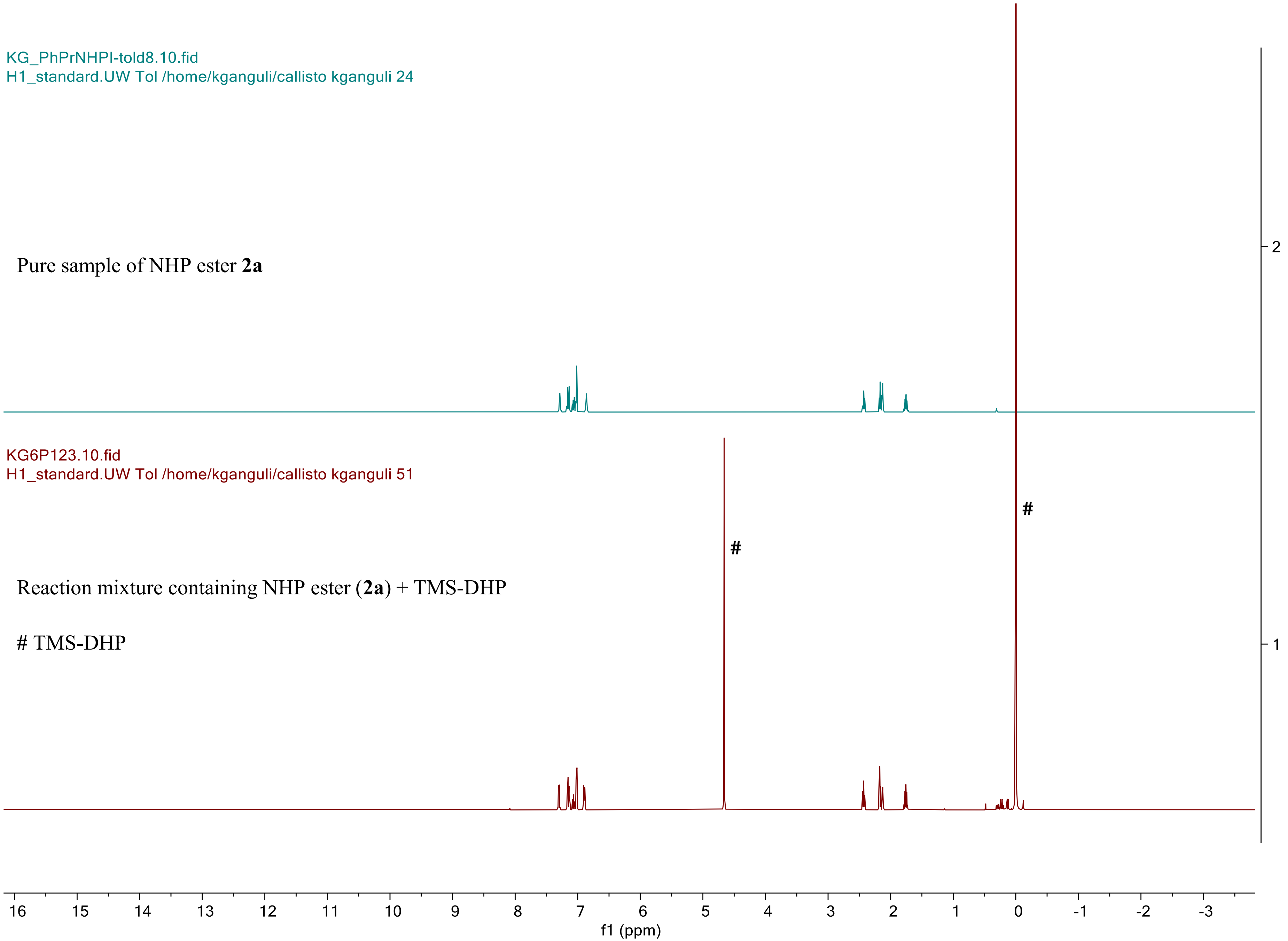

Figure S7. $^{13}C\{^{1}H\}$ **NMR (126 MHz, toluene-d_8) of reaction mixture of 2a + TMS-DHP + Na_2CO_3 (above) versus the pure substrate 2a (below).**

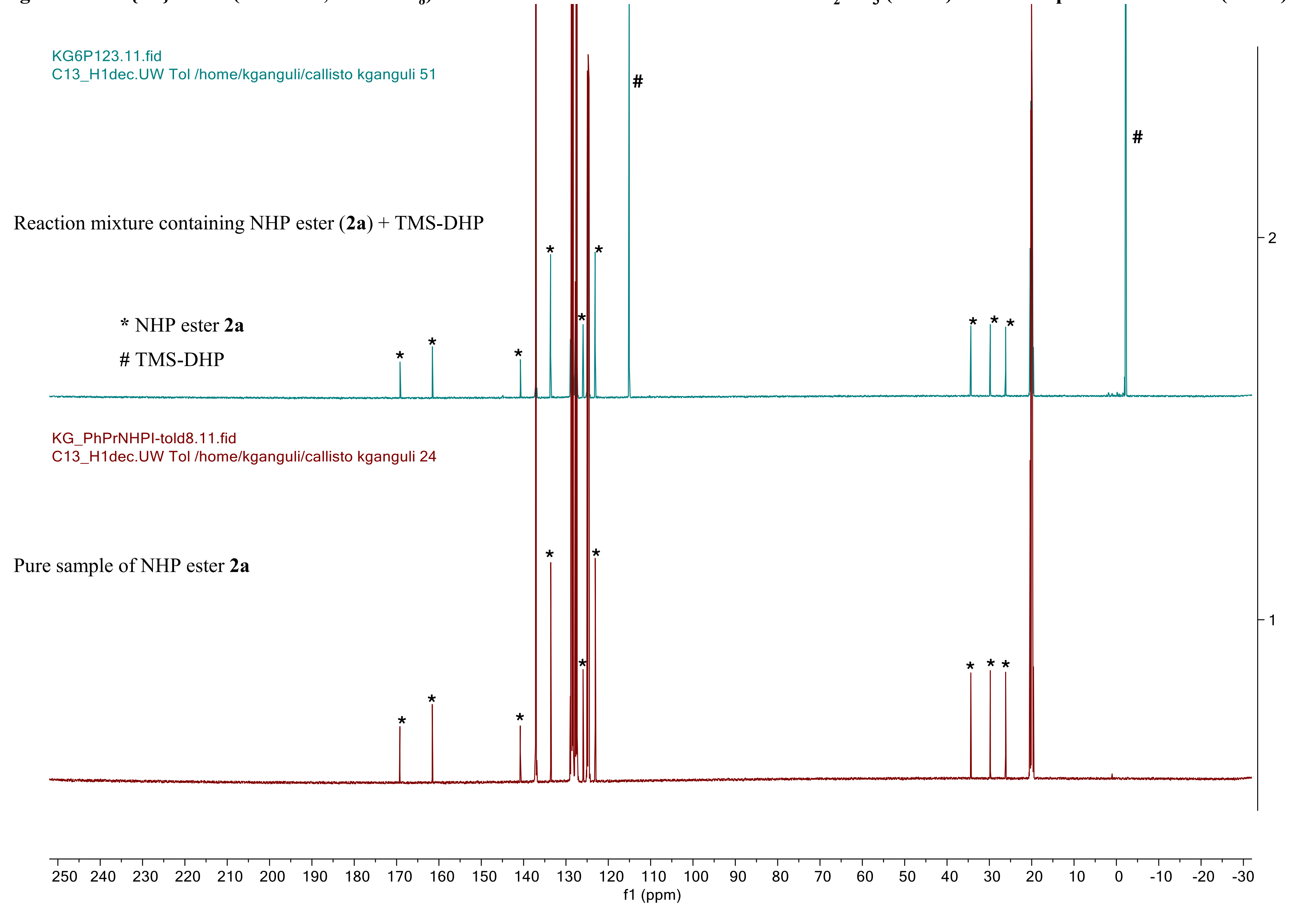

^{1}H NMR (500 MHz) in $CDCl_3$) of the isolated (dtbbpy)Ni(Ar)(NPhth) complex.

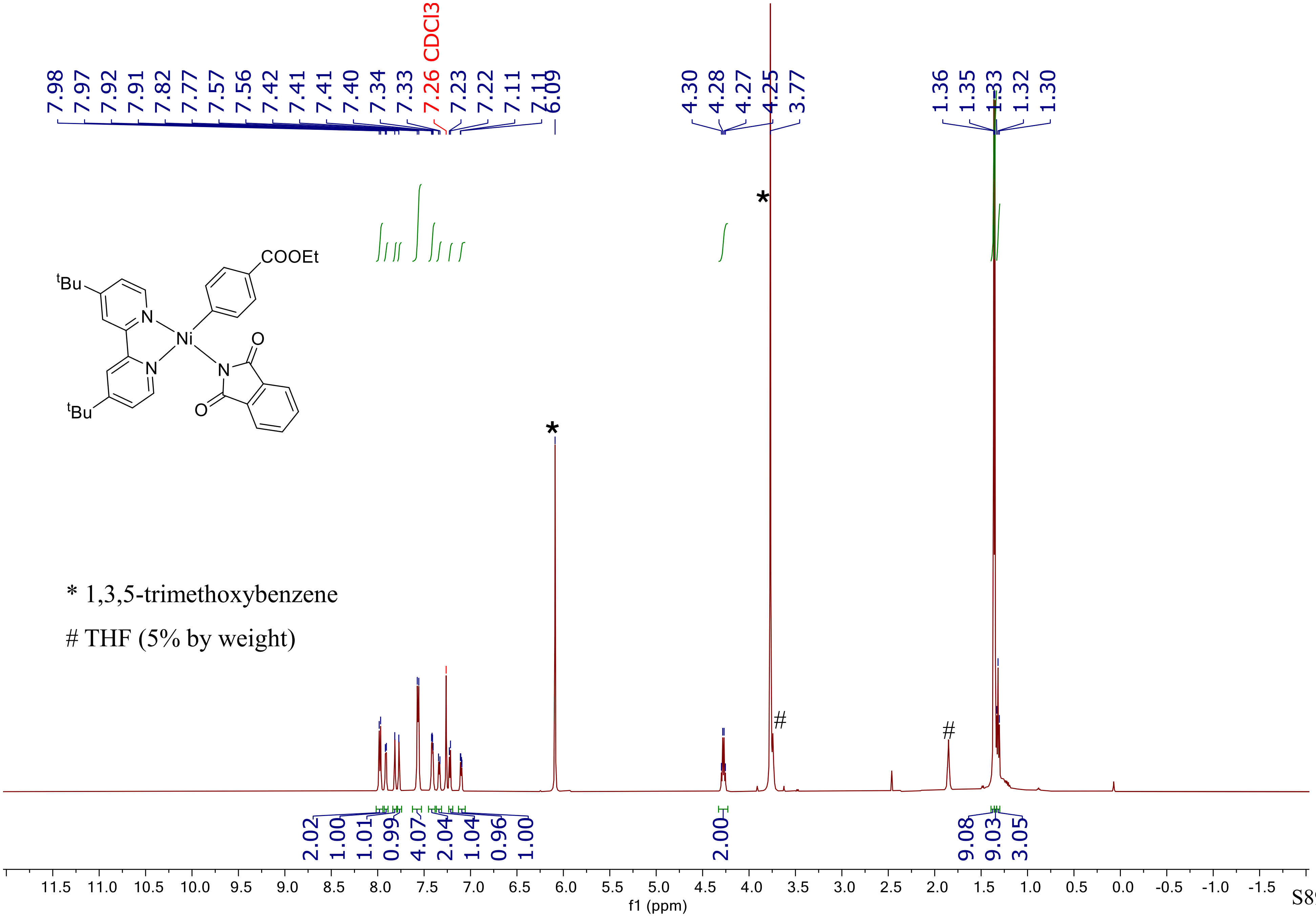

$^{13}C\{^{1}H\}$ NMR (126 MHz) in $CDCl_3$) of the isolated (dtbbpy)Ni(Ar)(NPhth) complex.

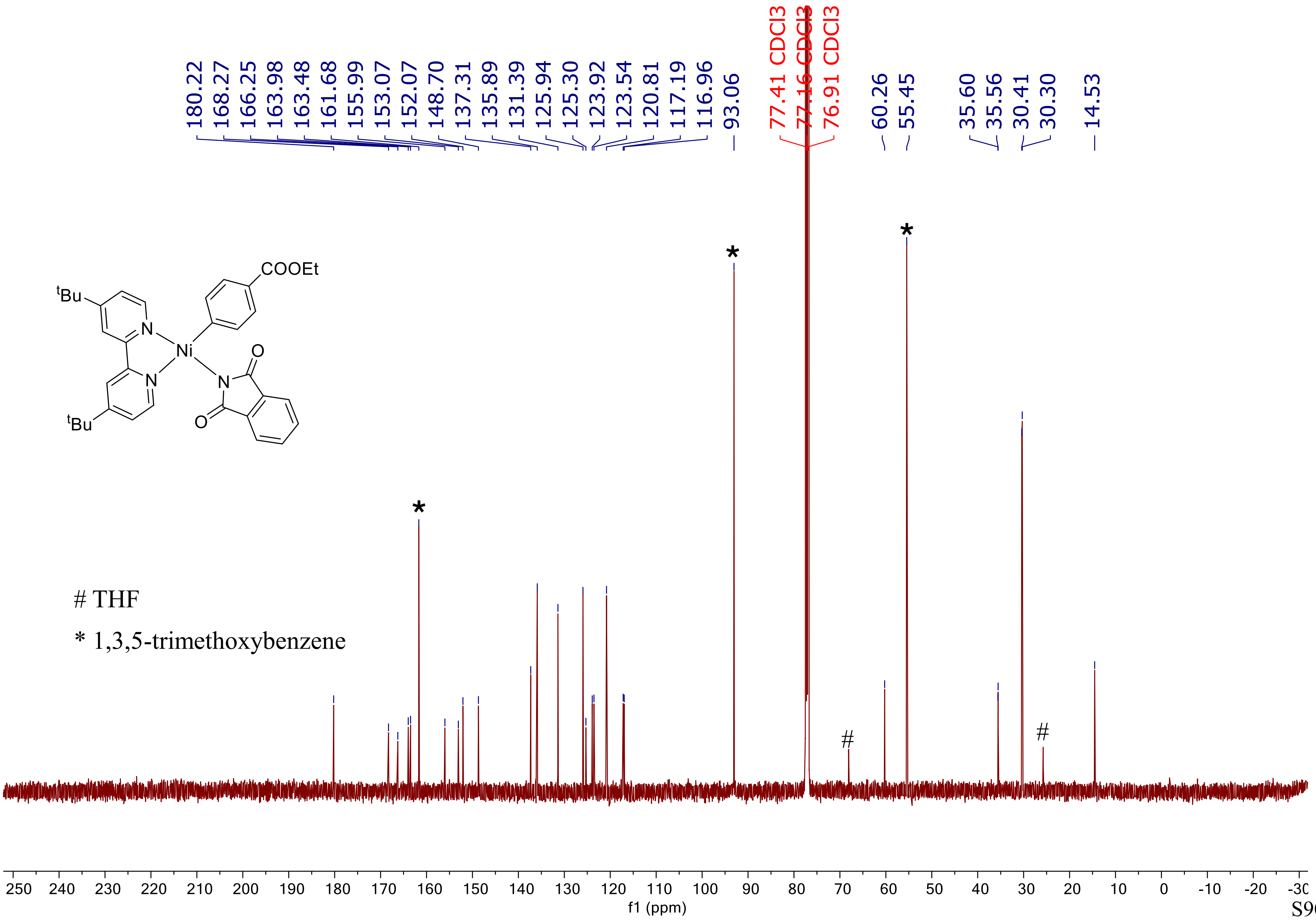

^{1}H NMR (500 MHz) in $CDCl_3$

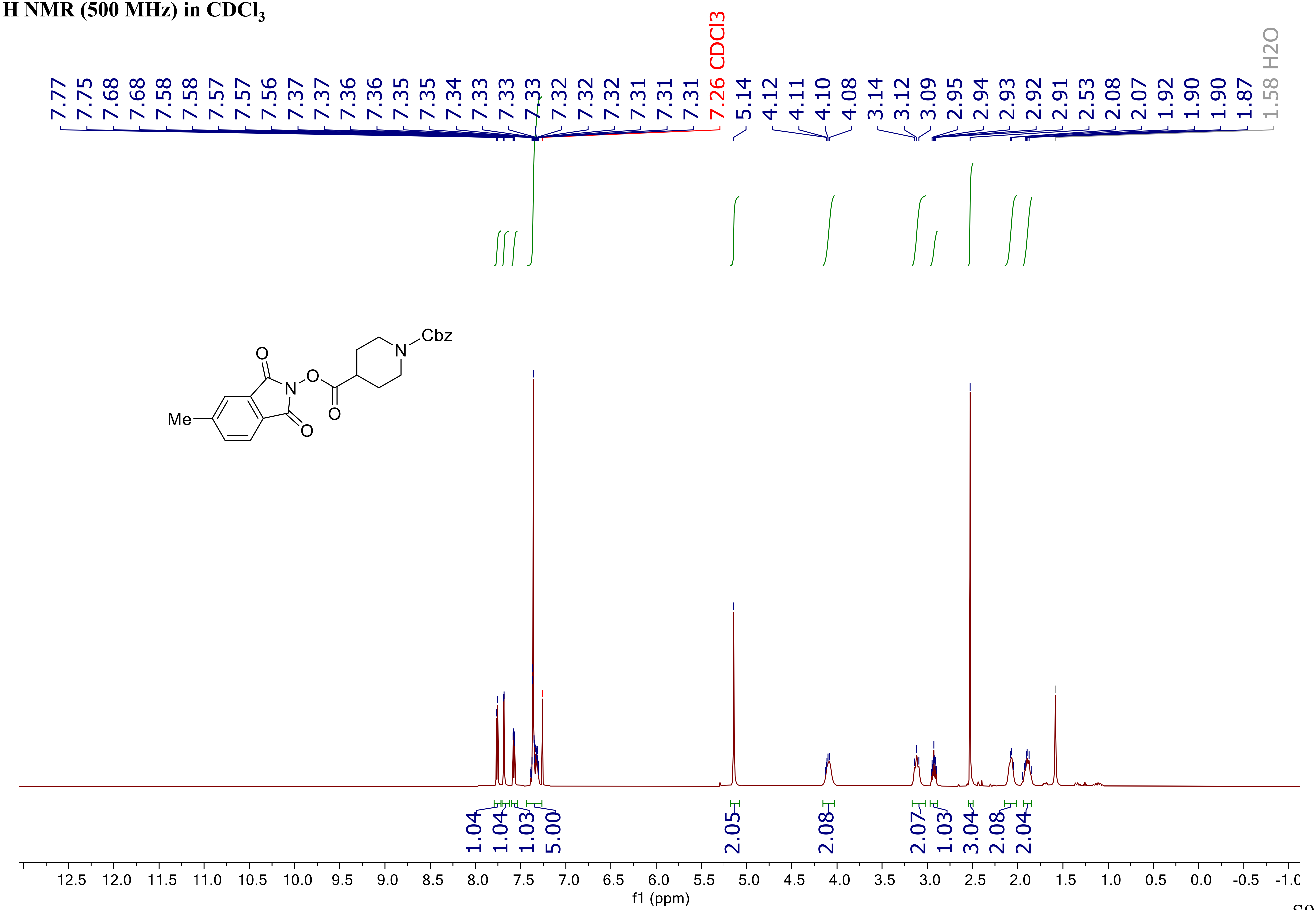

$^{13}C\{^{1}H\}$ NMR (126 MHz) in $CDCl_3$

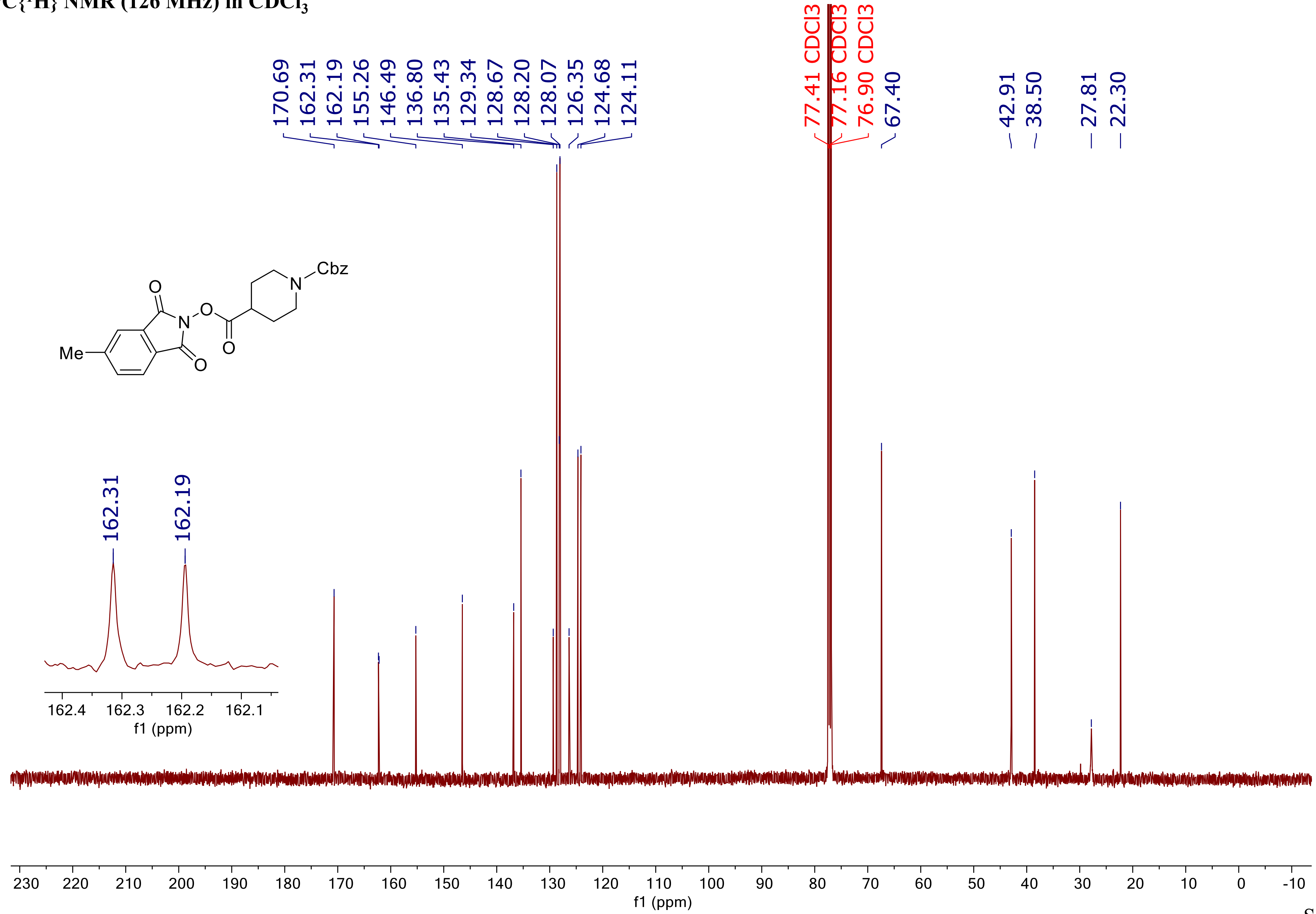

^{1}H NMR (500 MHz) in $CDCl_3$

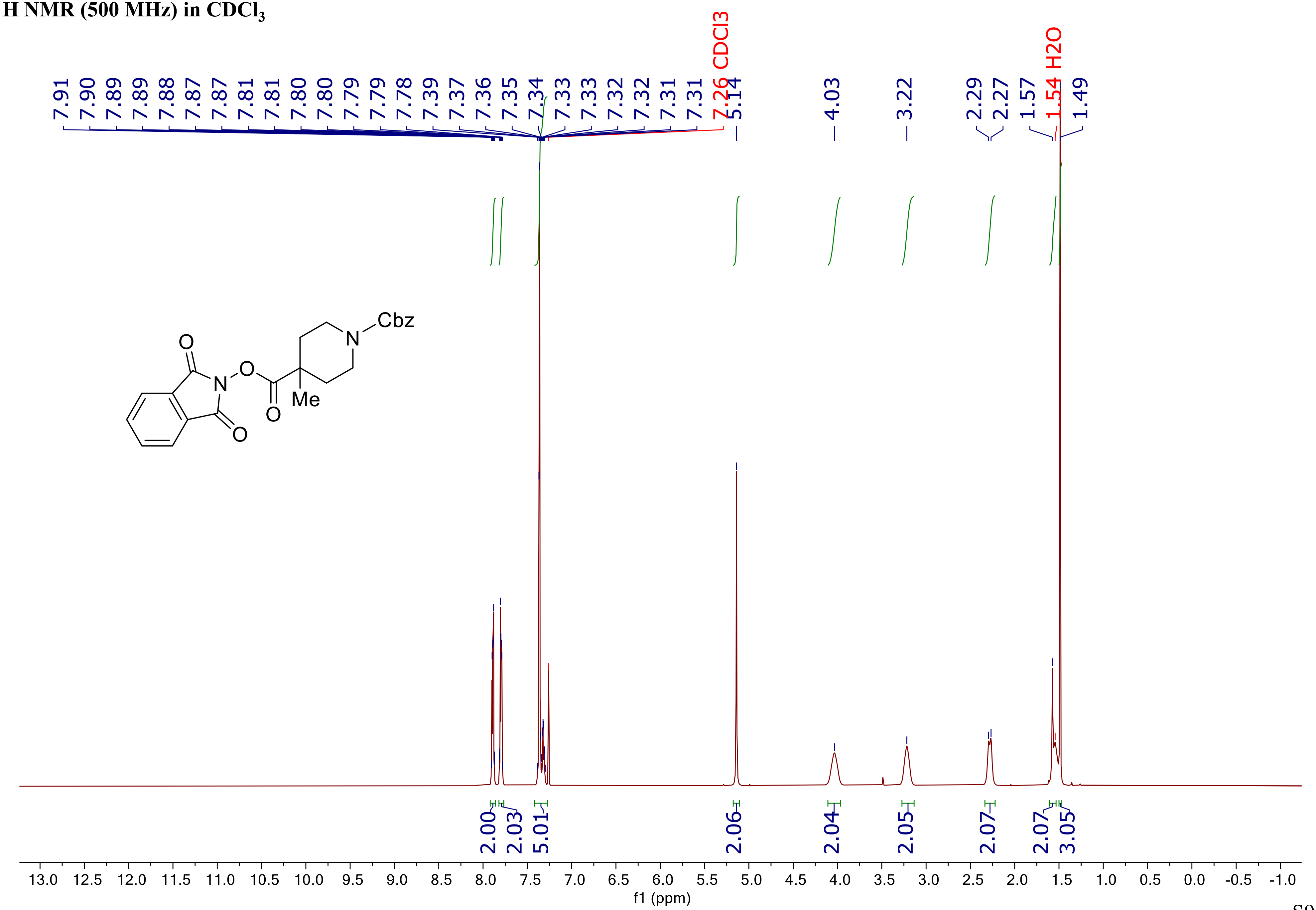

$^{13}C\{^{1}H\}$ NMR (126 MHz) in $CDCl_3$

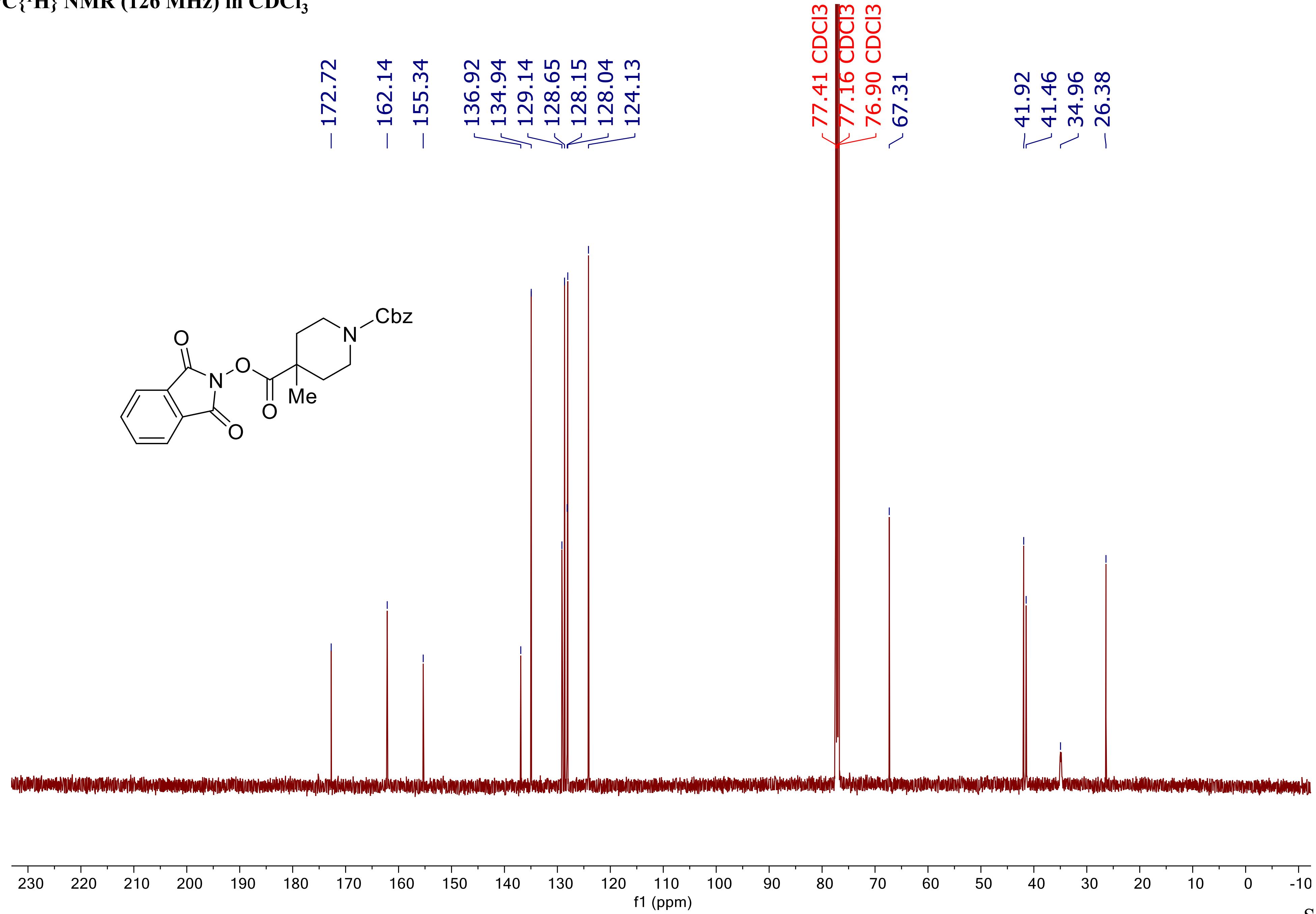

^{1}H NMR (500 MHz) in C_6D_6

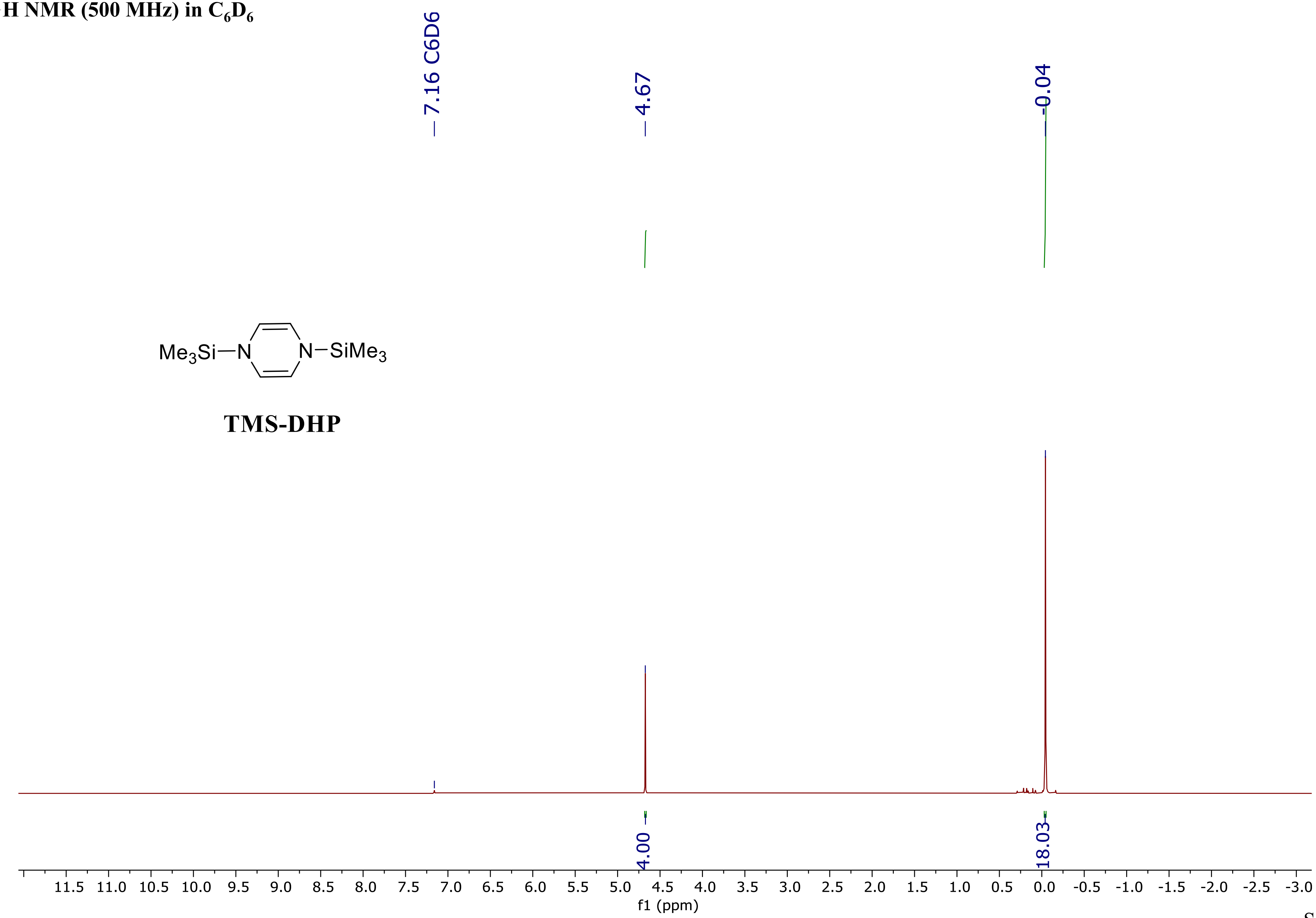

TMS-DHP

$^{13}C\{^{1}H\}$ NMR (126 MHz) in C_6D_6

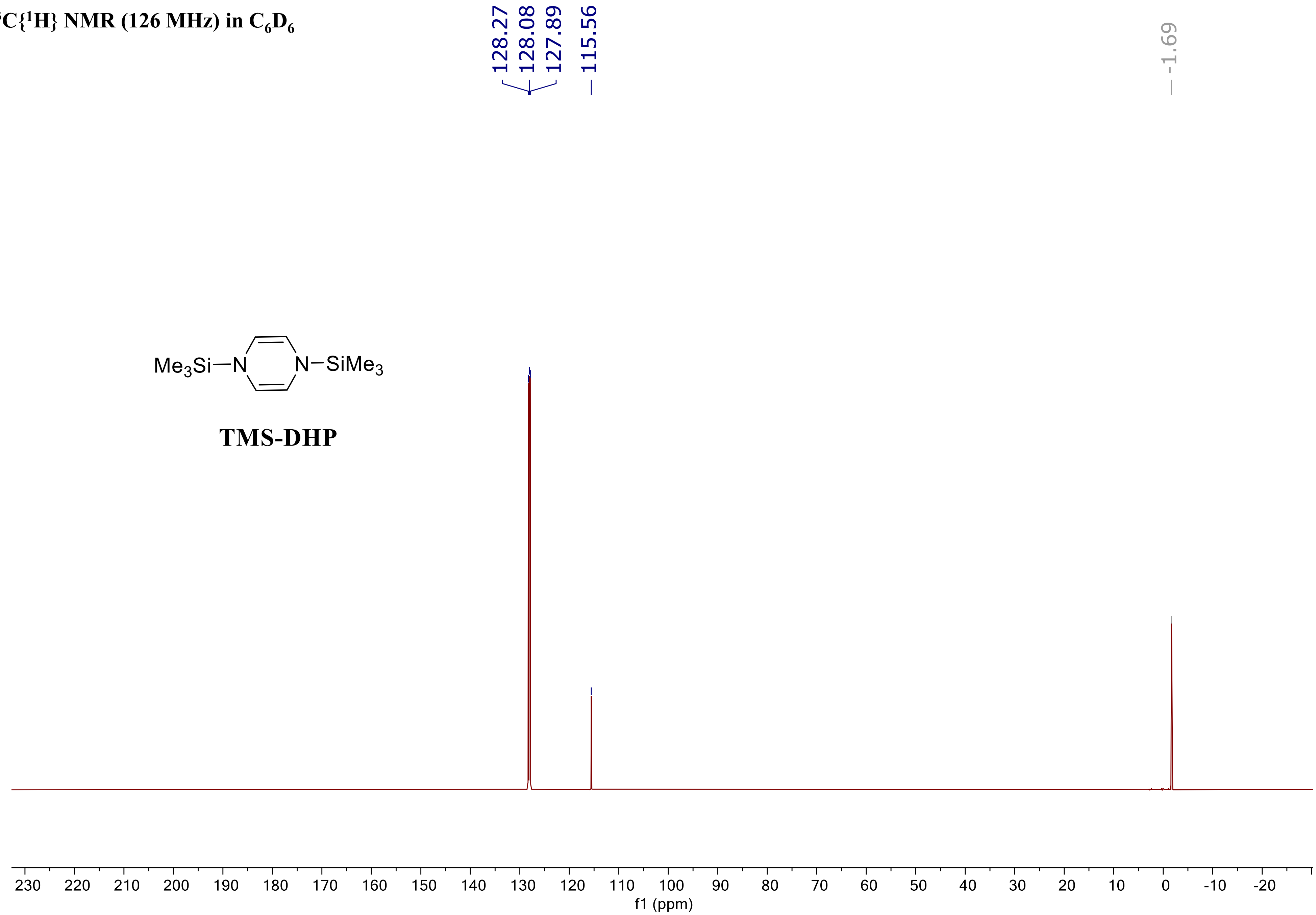

^1H NMR (500 MHz) in C_6D_6

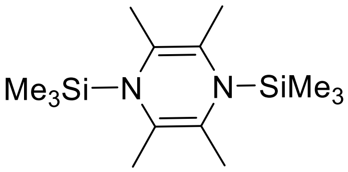

TMS-Me$_4$DHP

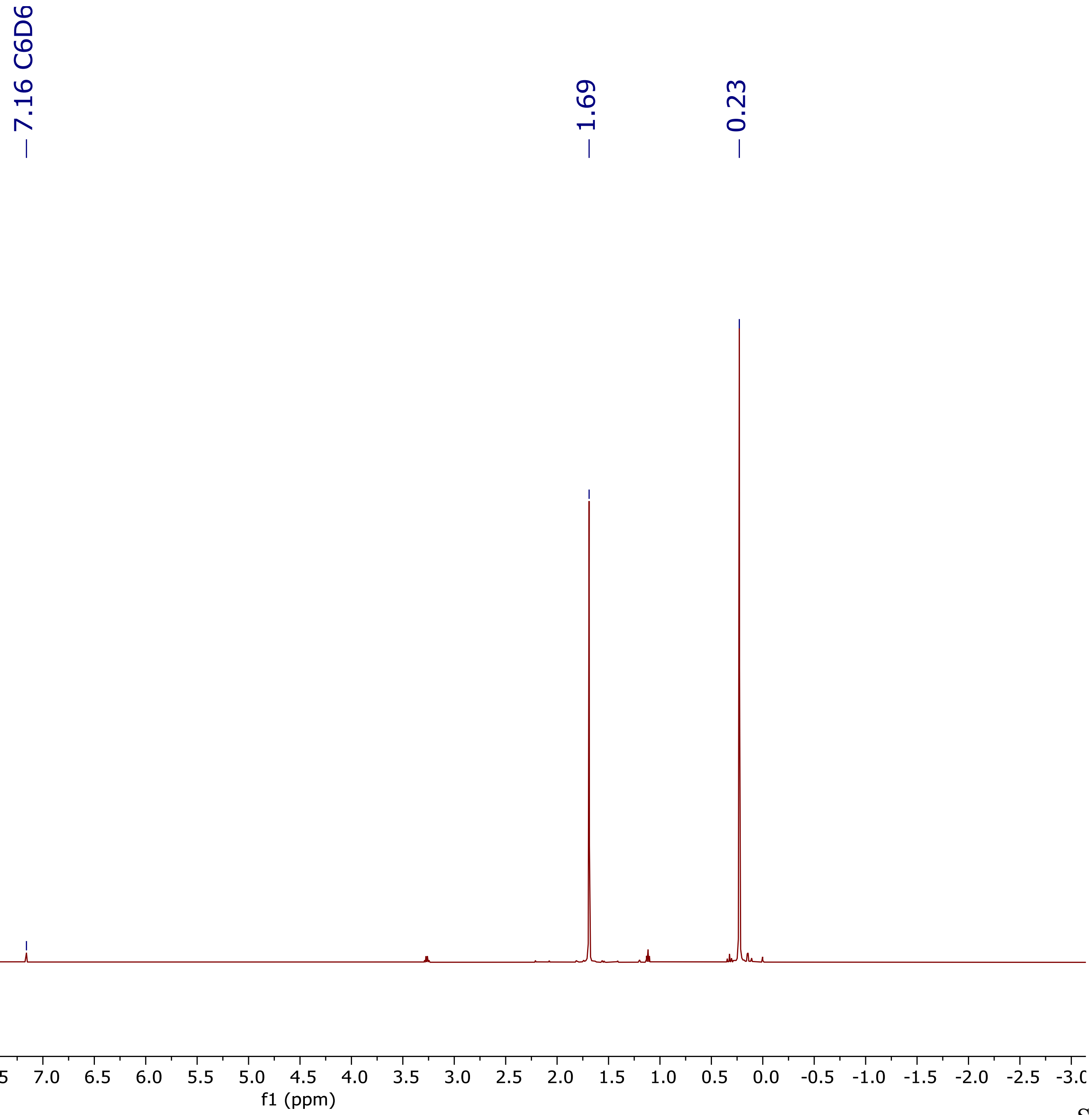

$^{13}C\{^{1}H\}$ NMR (126 MHz) in C_6D_6

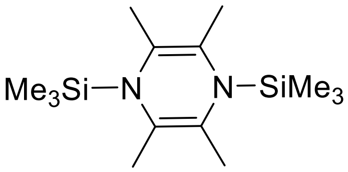

TMS-Me$_4$DHP

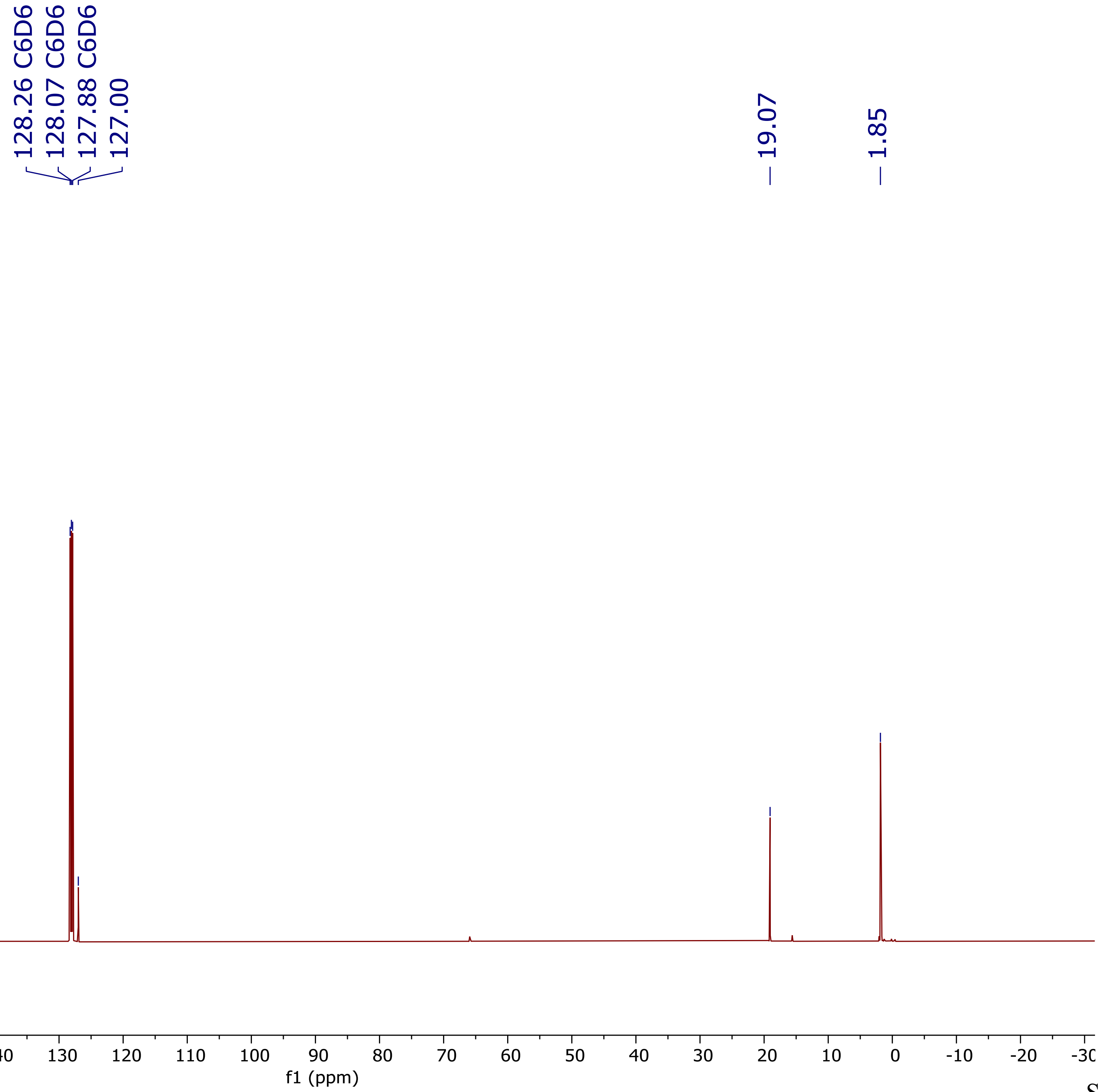

^{1}H NMR (500 MHz) in C_6D_6

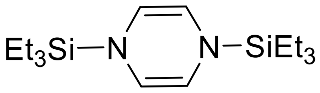

TES-DHP

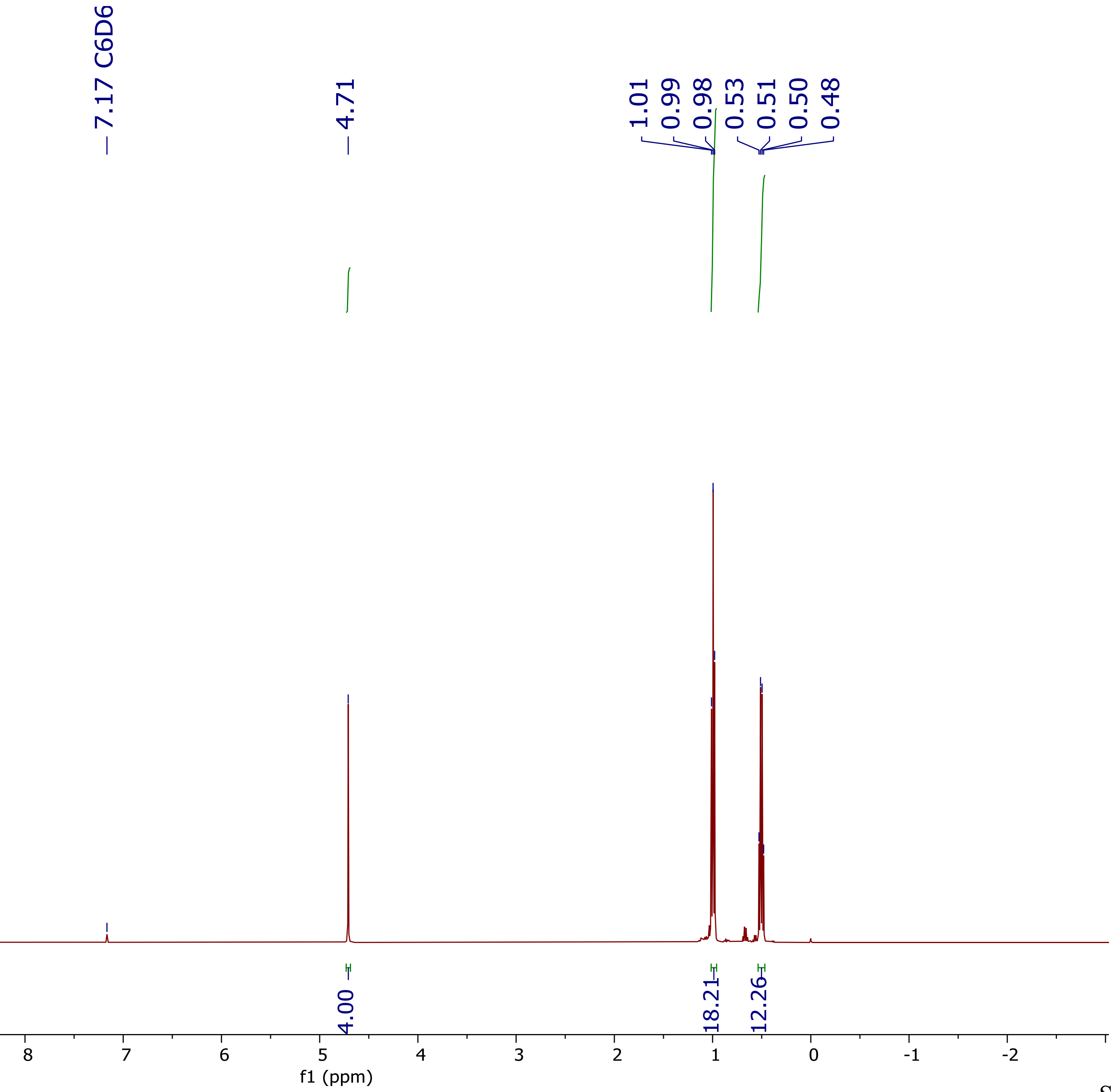

$^{13}C\{^1H\}$ NMR (126 MHz) in C_6D_6

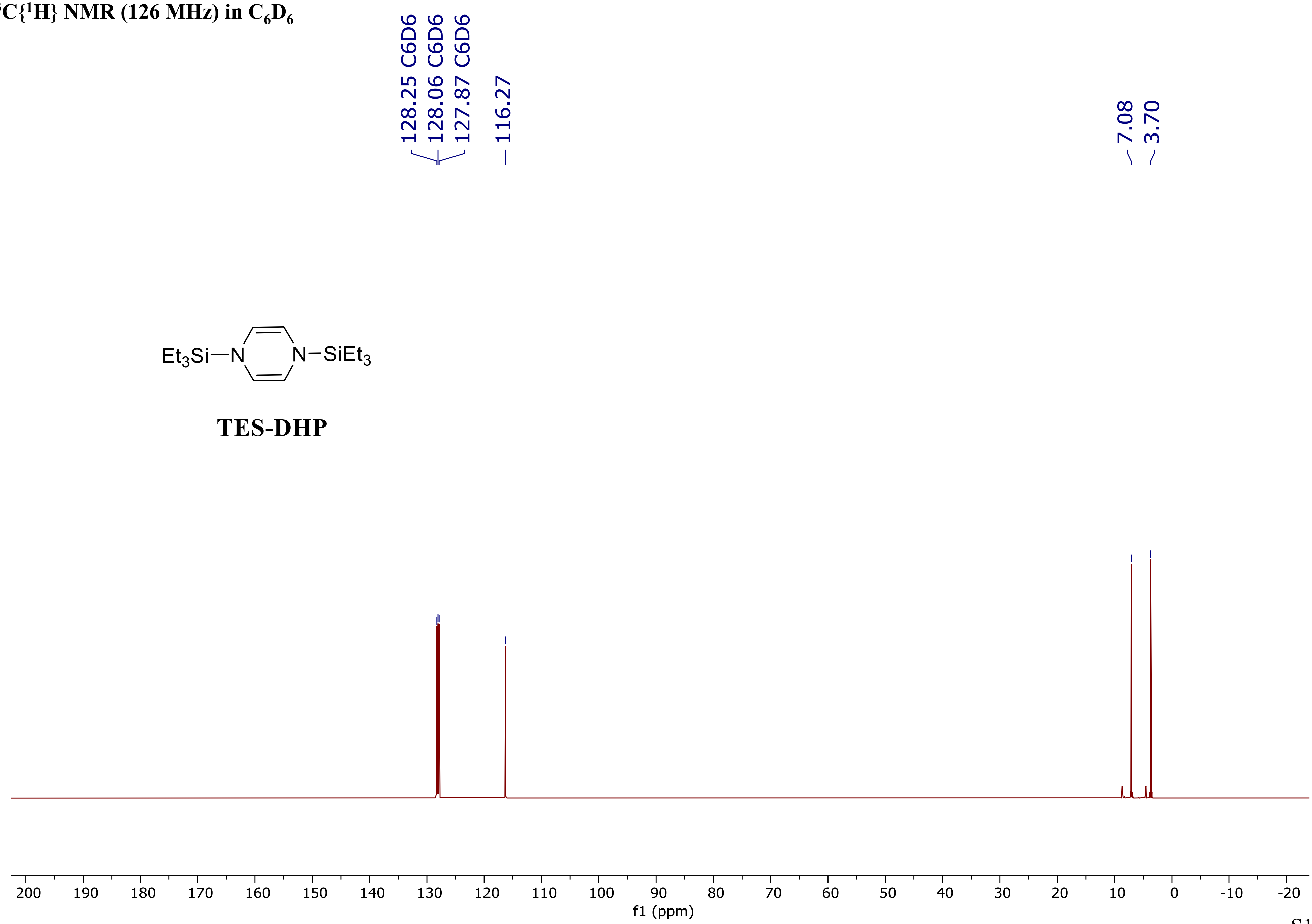

^{1}H NMR (500 MHz) in C_6D_6

TMS-DHB

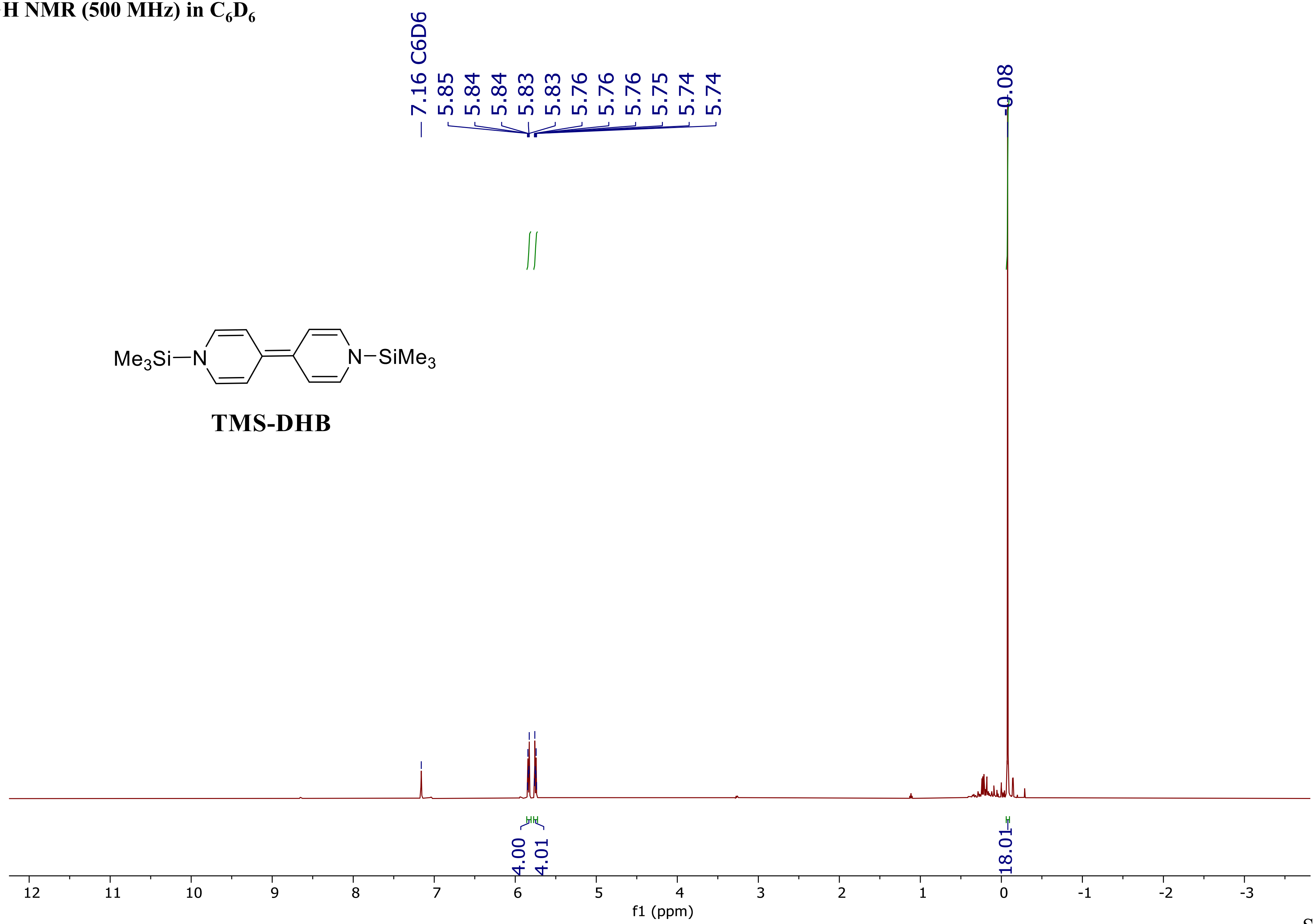

$^{13}C\{^1H\}$ NMR (126 MHz) in C_6D_6

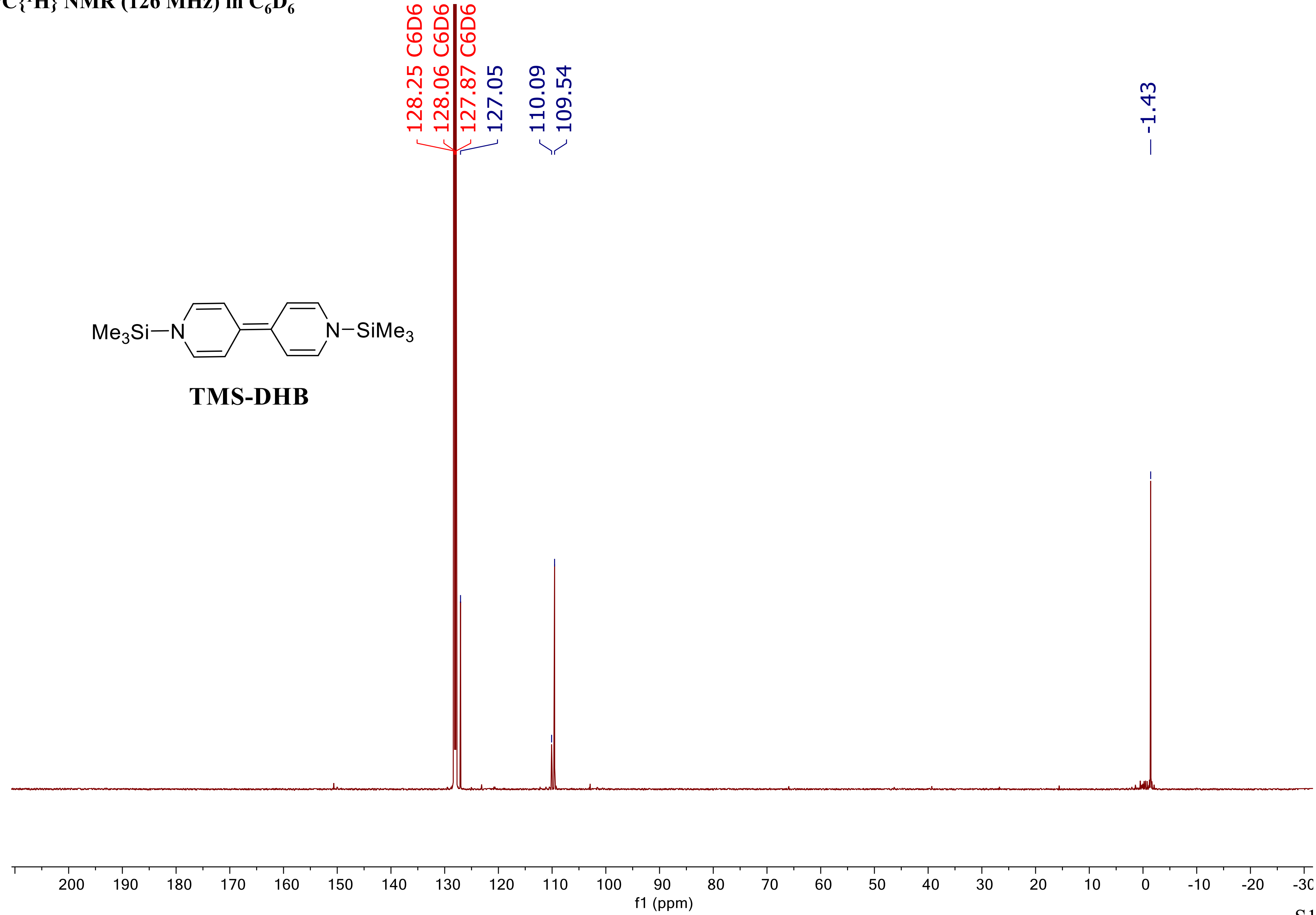

^{1}H NMR (500 MHz) in C_6D_6

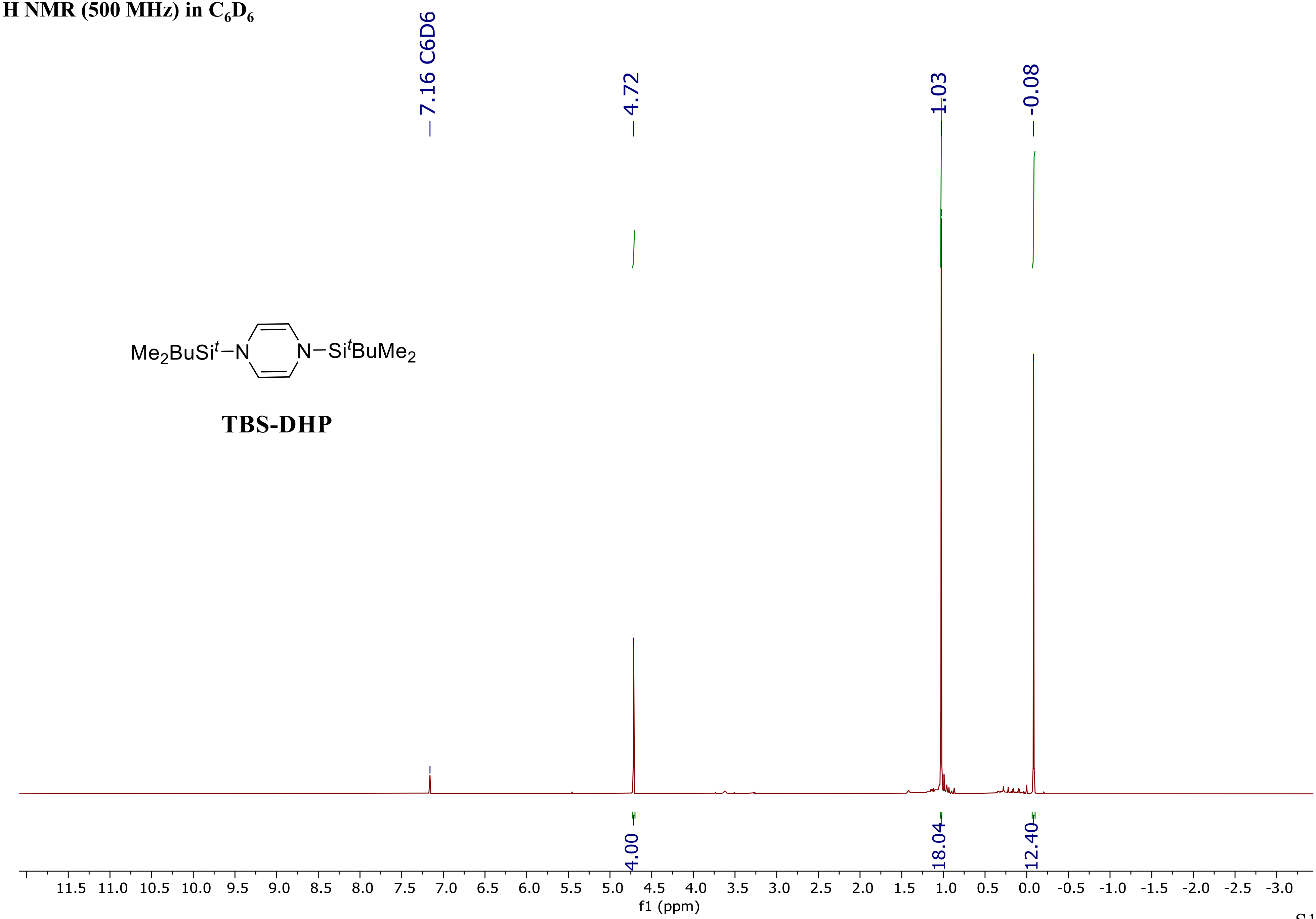

$^{13}C\{^{1}H\}$ NMR (126 MHz) in C_6D_6

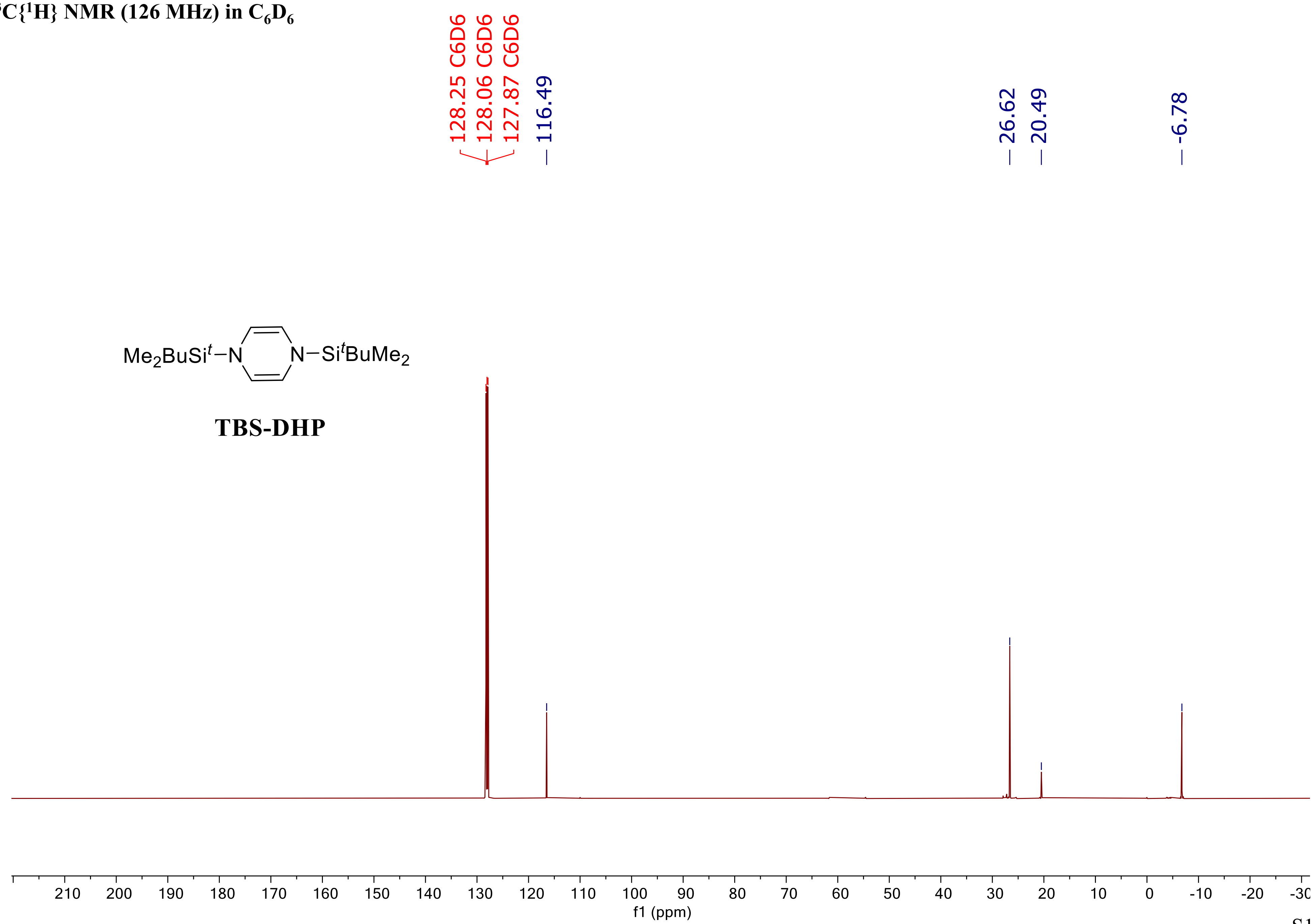

^{1}H NMR of X10 (500 MHz) in $CDCl_3$

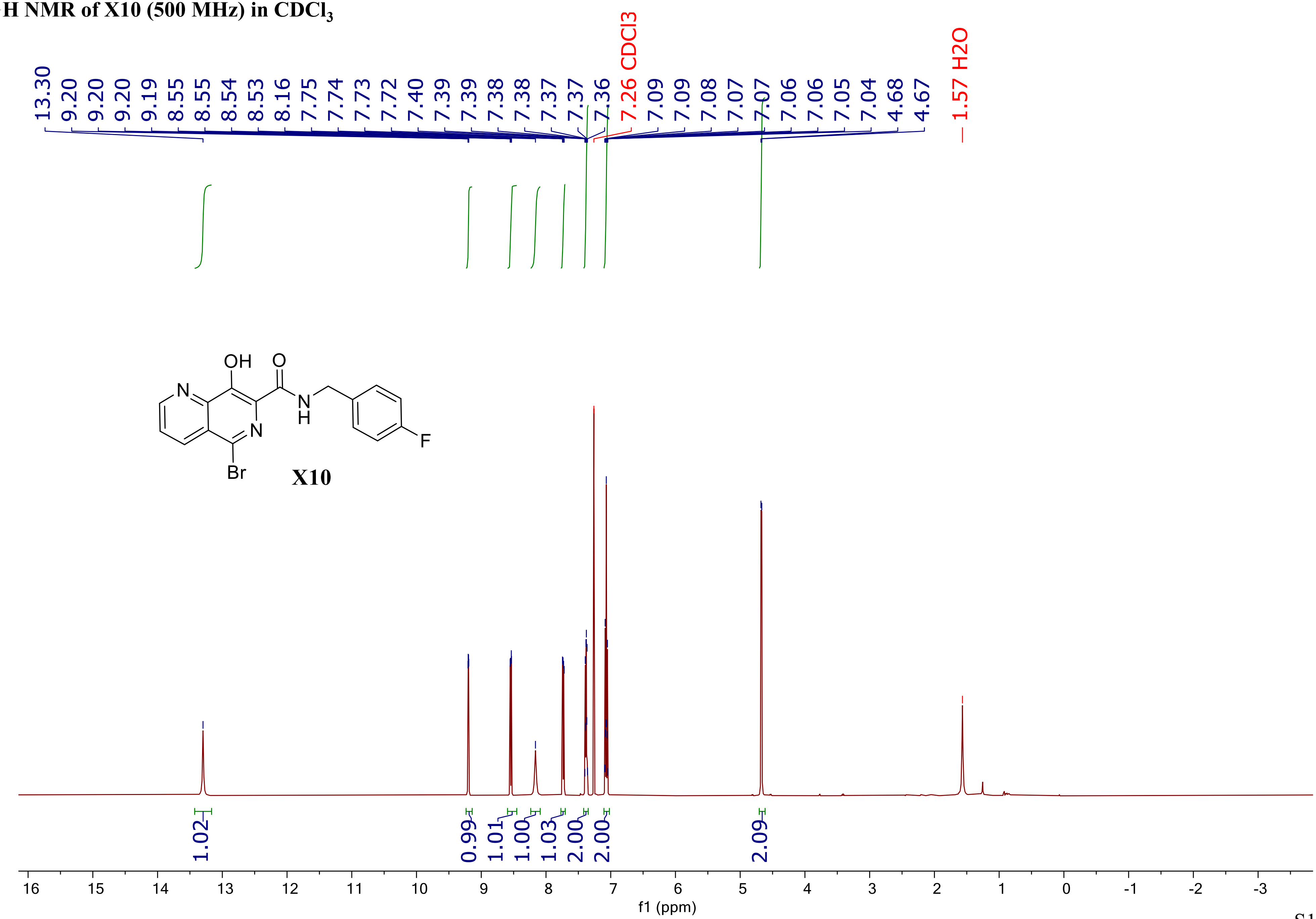

$^{13}C\{^{1}H\}$ NMR of X10 (126 MHz) in $CDCl_3$

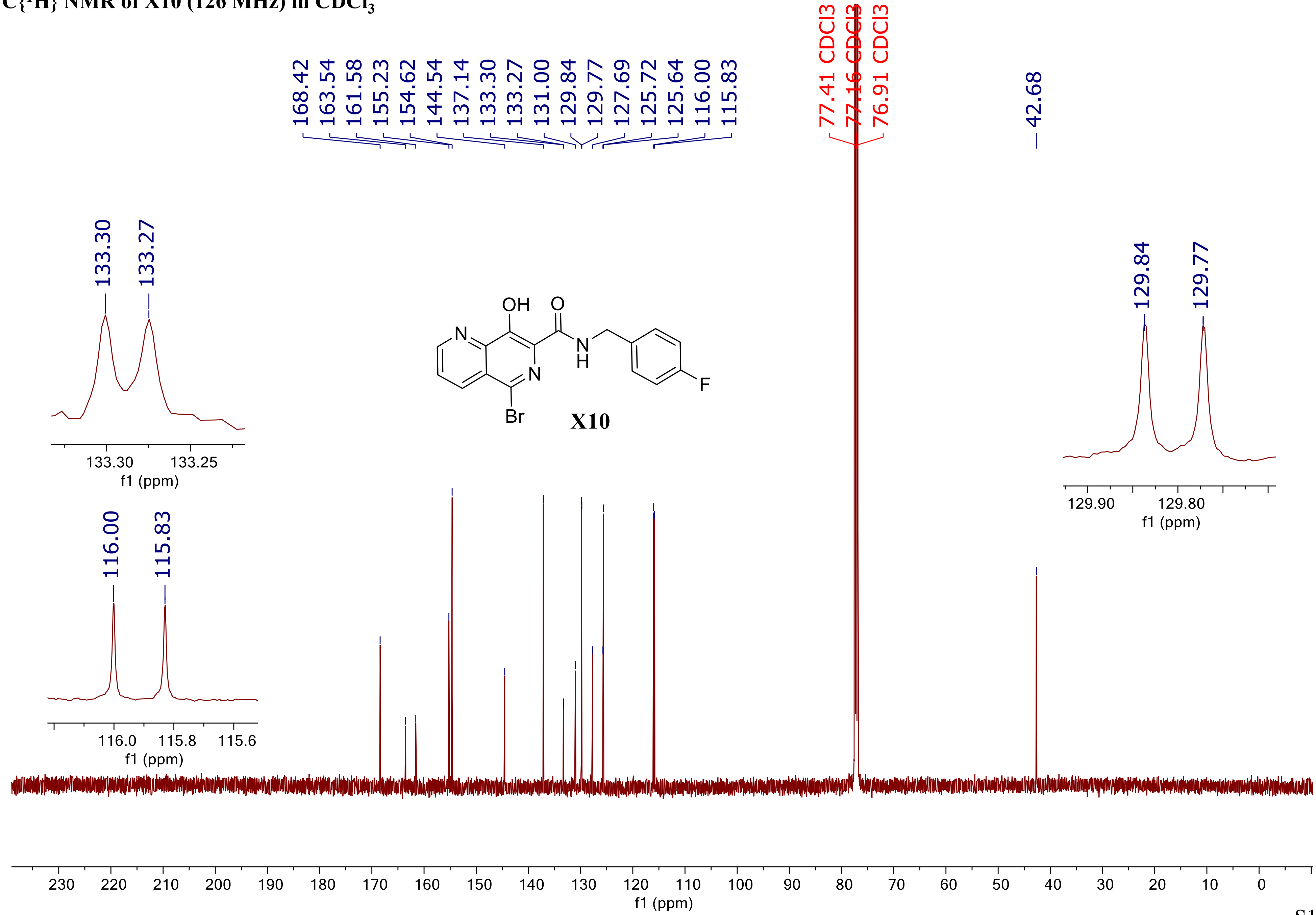

$^{19}F\{^{1}H\}$ NMR of X10 (377 MHz) in $CDCl_3$

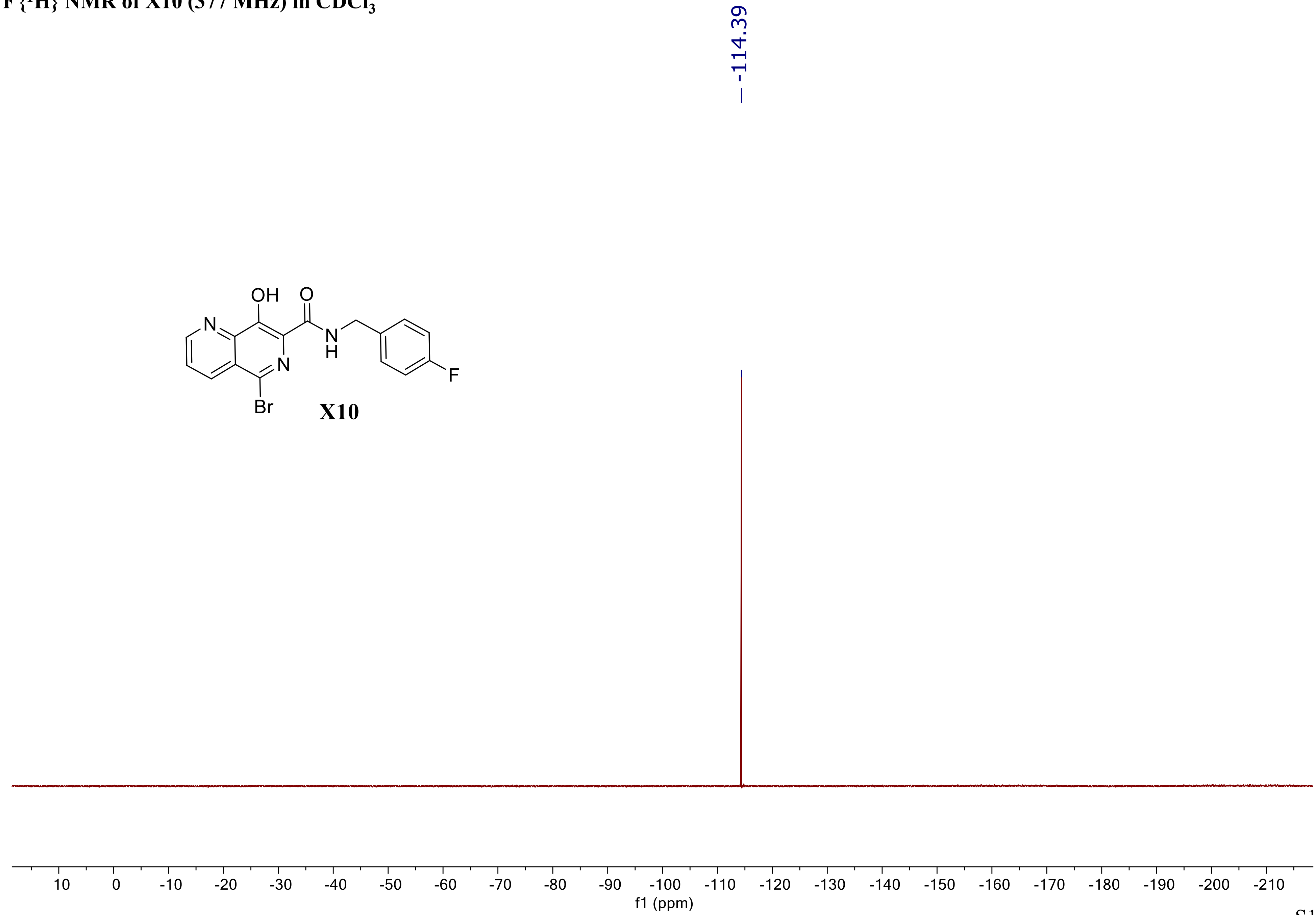

^{1}H NMR (500 MHz) of 3a in $CDCl_3$

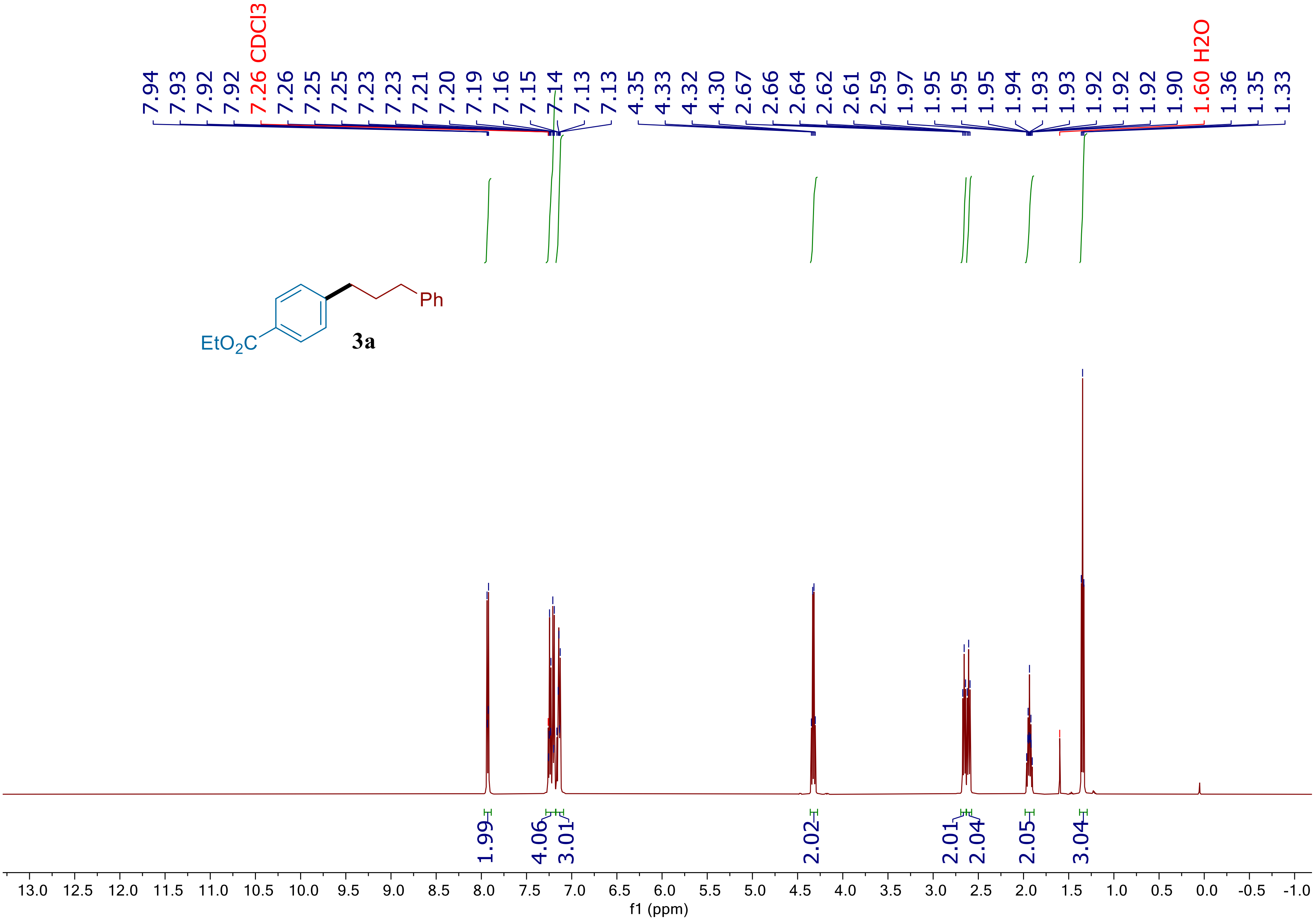

$^{13}C\{^{1}H\}$ NMR (126 MHz) of 3a in $CDCl_3$

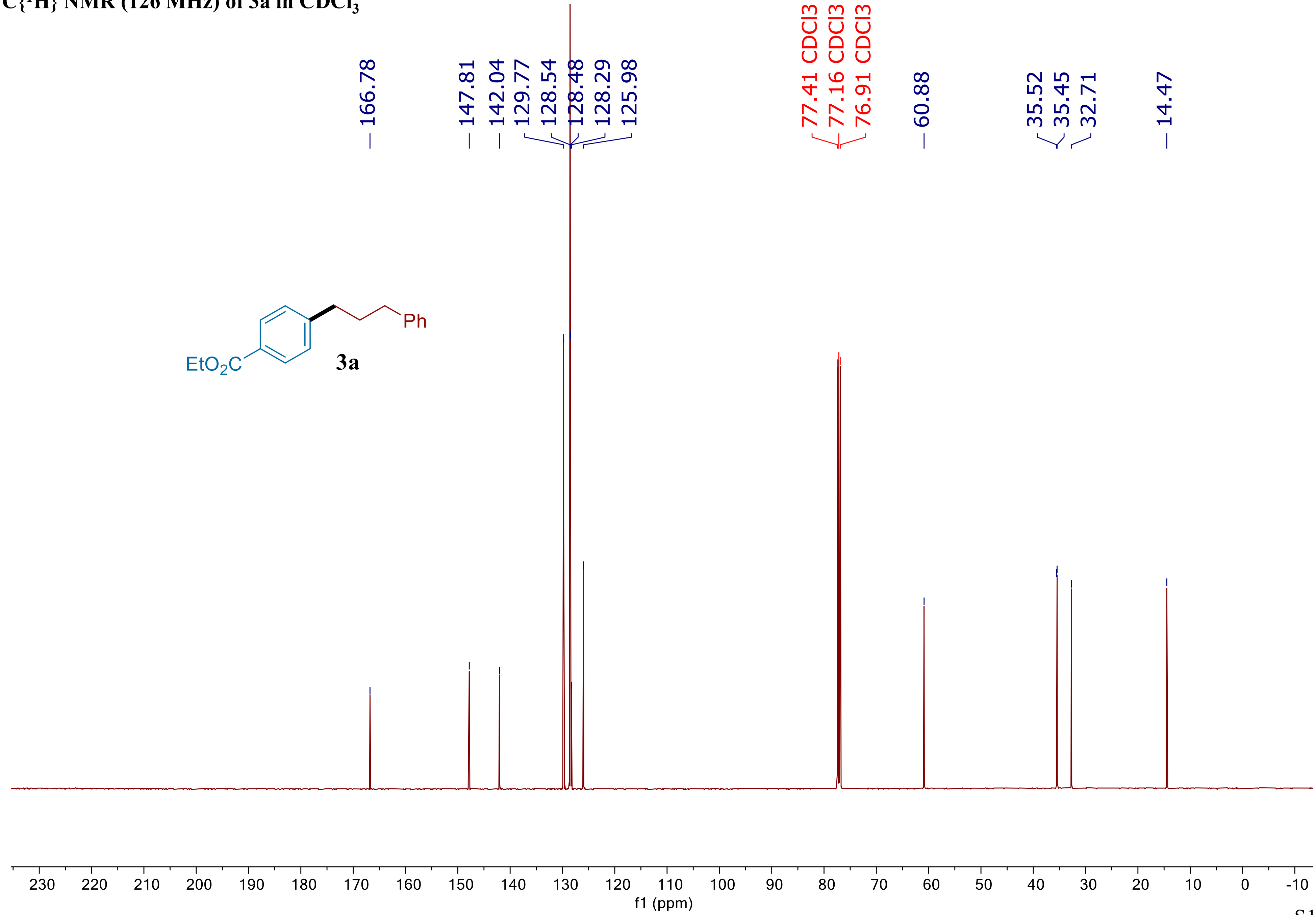

^{1}H NMR (500 MHz) of 3b in $CDCl_3$

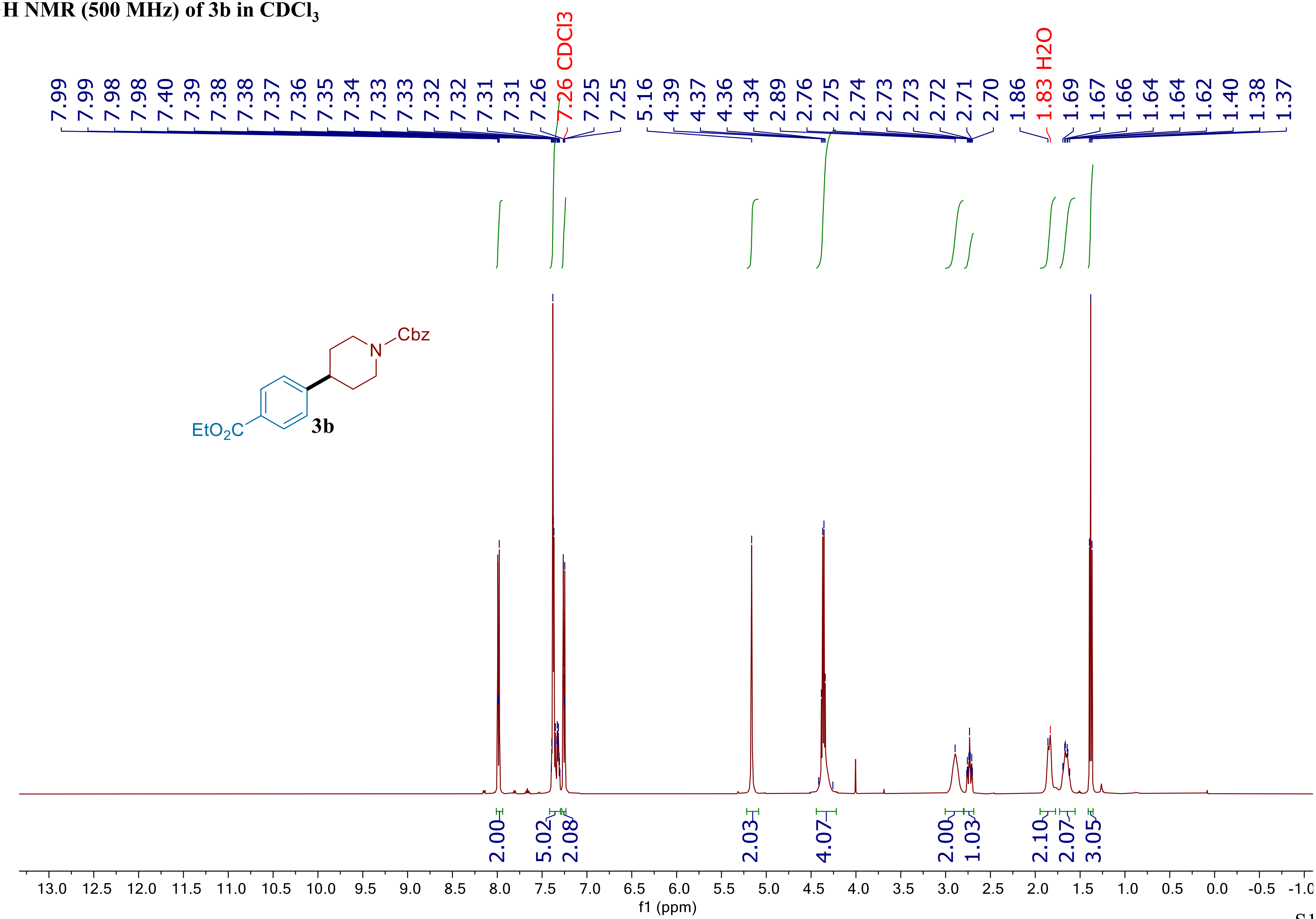

$^{13}C\{^{1}H\}$ NMR (126 MHz) of 3b in $CDCl_3$

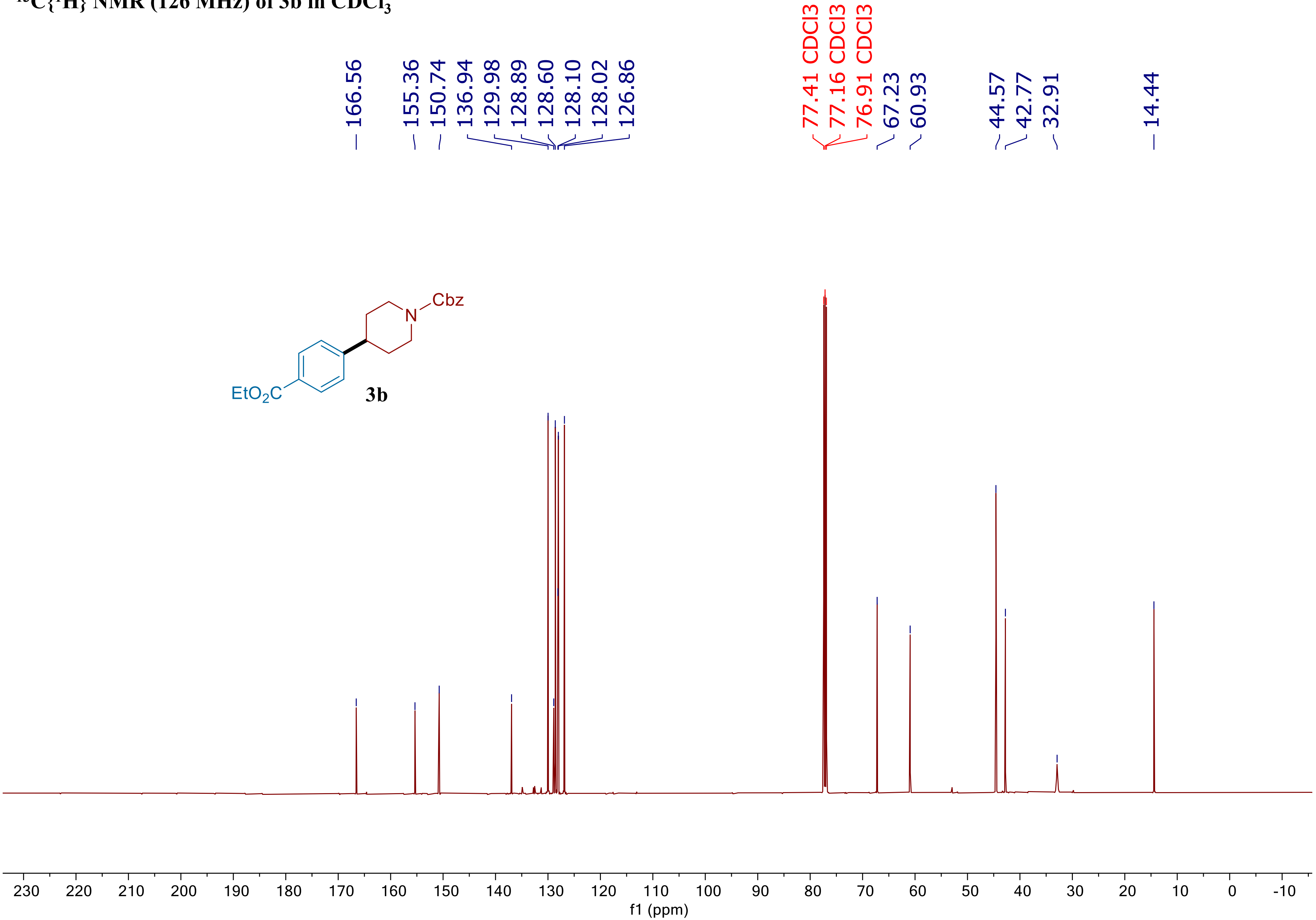

^{1}H NMR (500 MHz) of 3c in $CDCl_3$

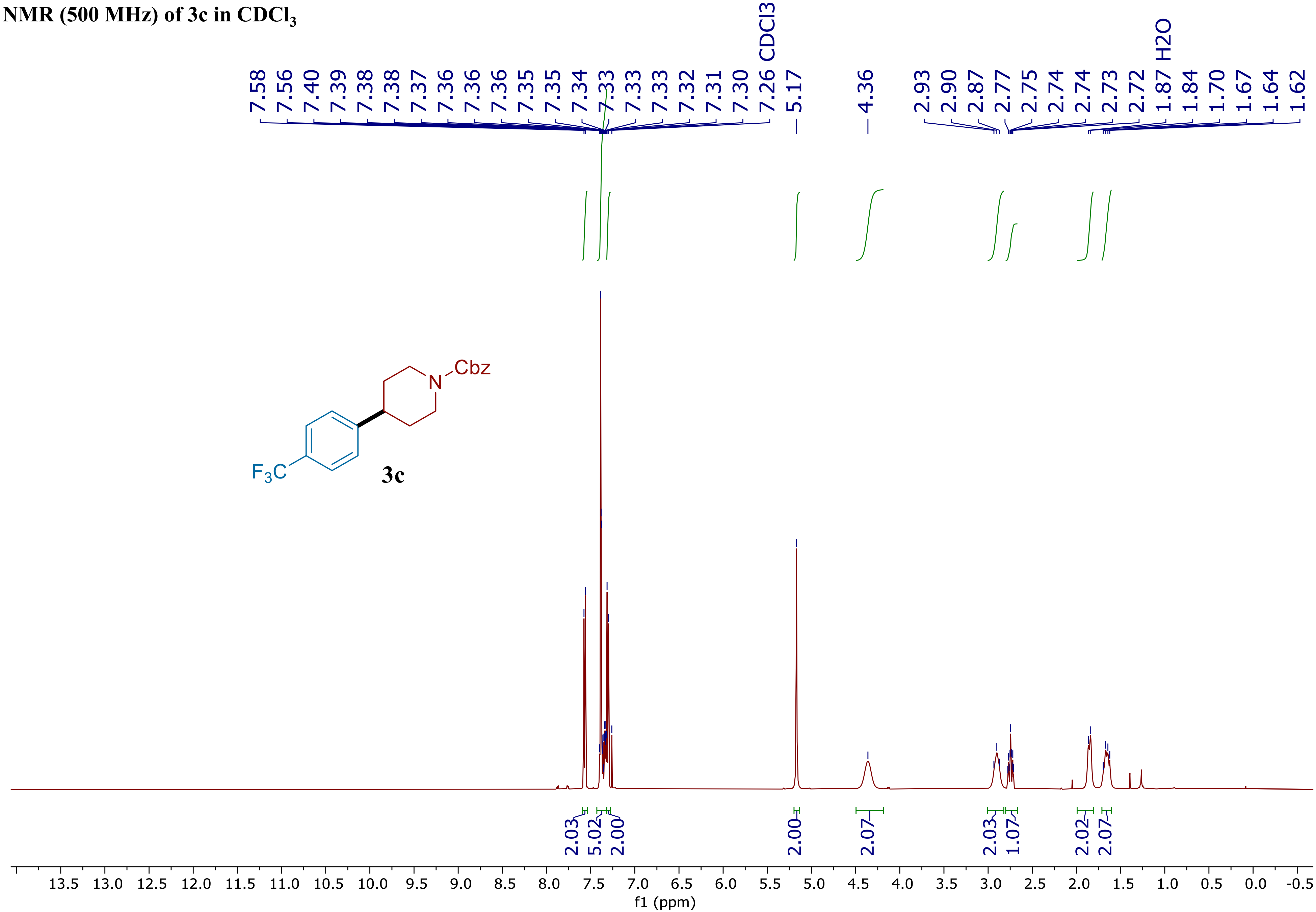

$^{13}C\{^1H\}$ NMR (126 MHz) of 3c in $CDCl_3$

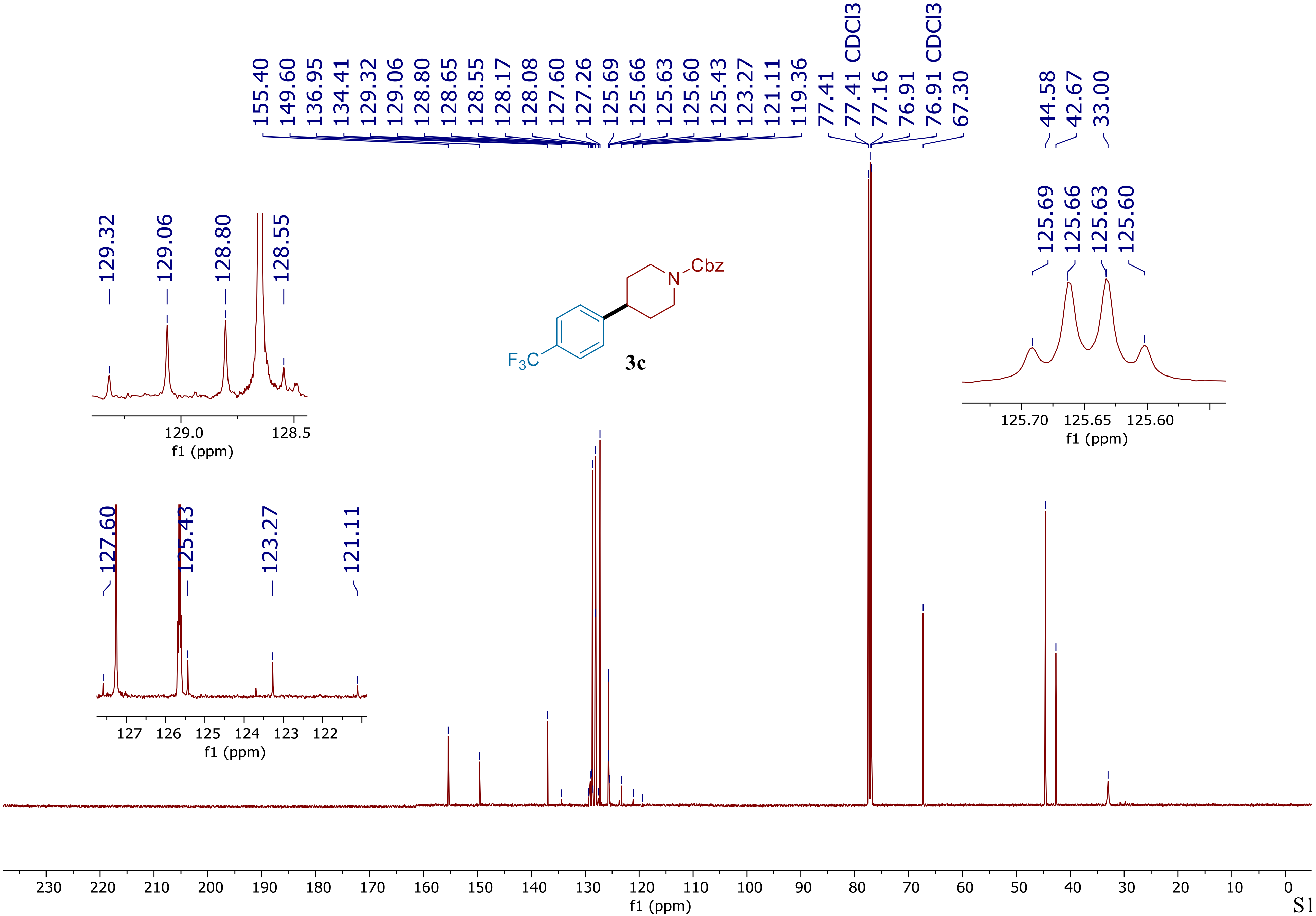

$^{19}F\{^{1}H\}$ NMR (377 MHz) of 3c in $CDCl_3$

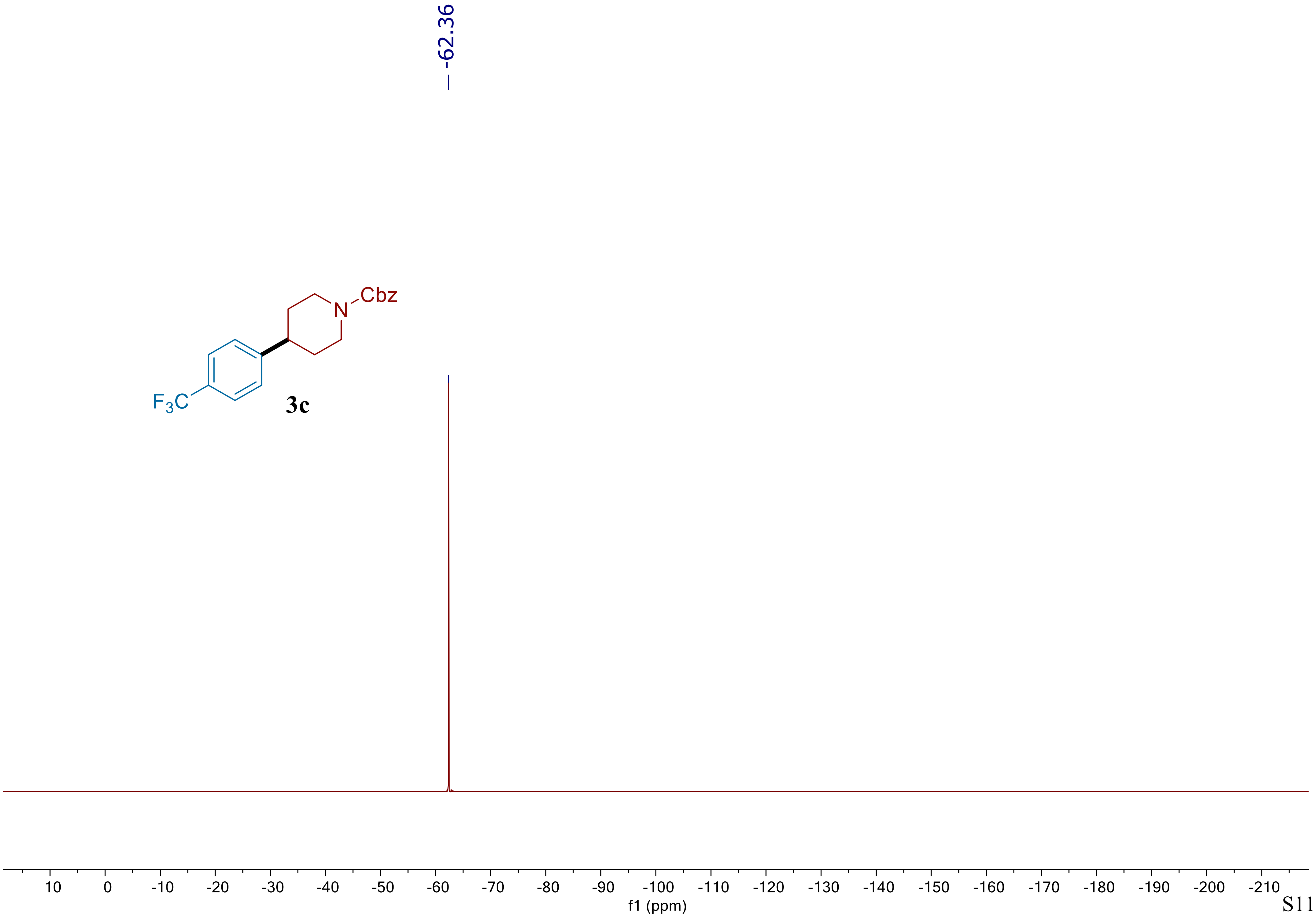

^{1}H NMR (500 MHz) of 3d in $CDCl_3$

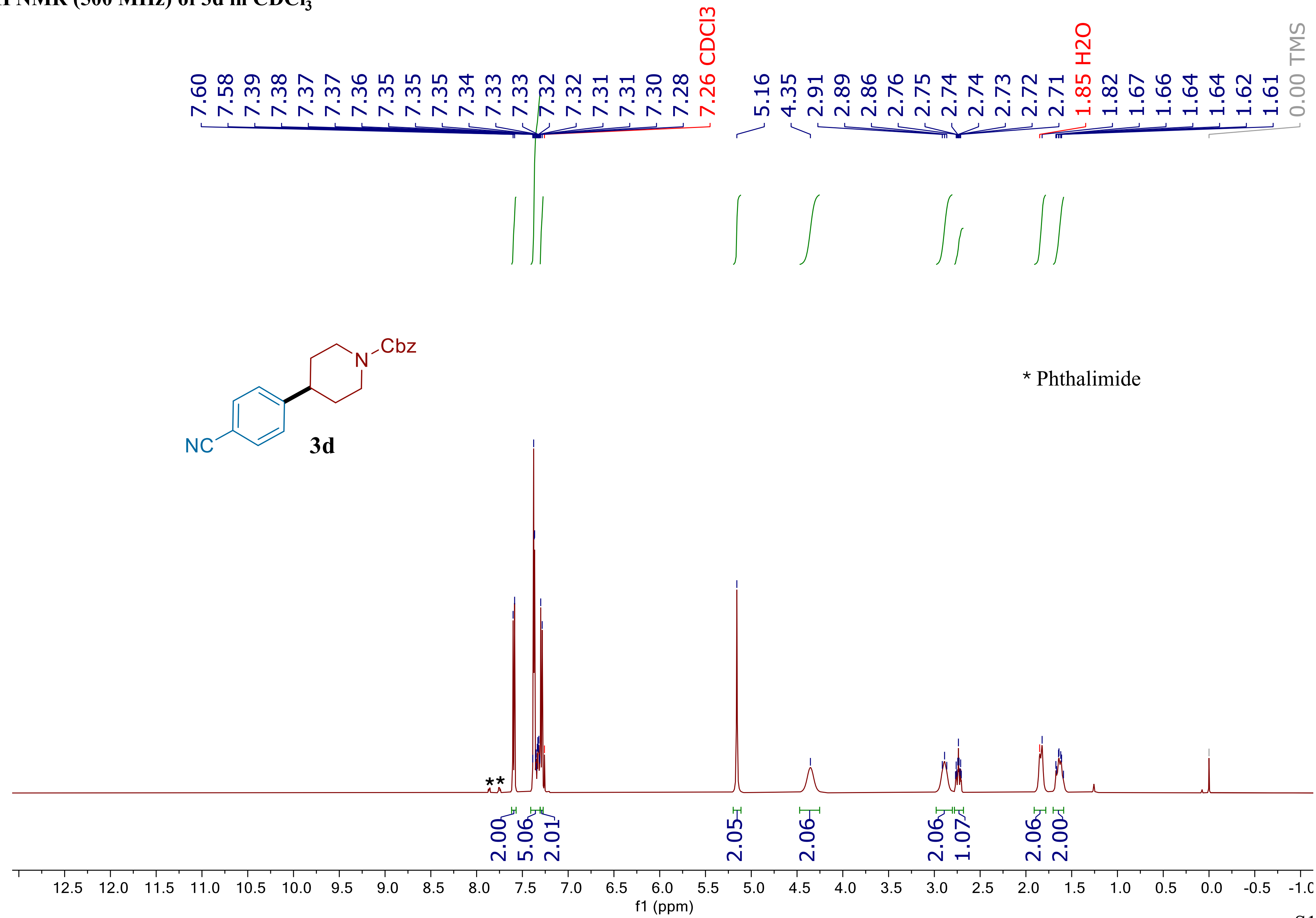

$^{13}C\{^{1}H\}$ NMR (126 MHz) of 3d in $CDCl_3$

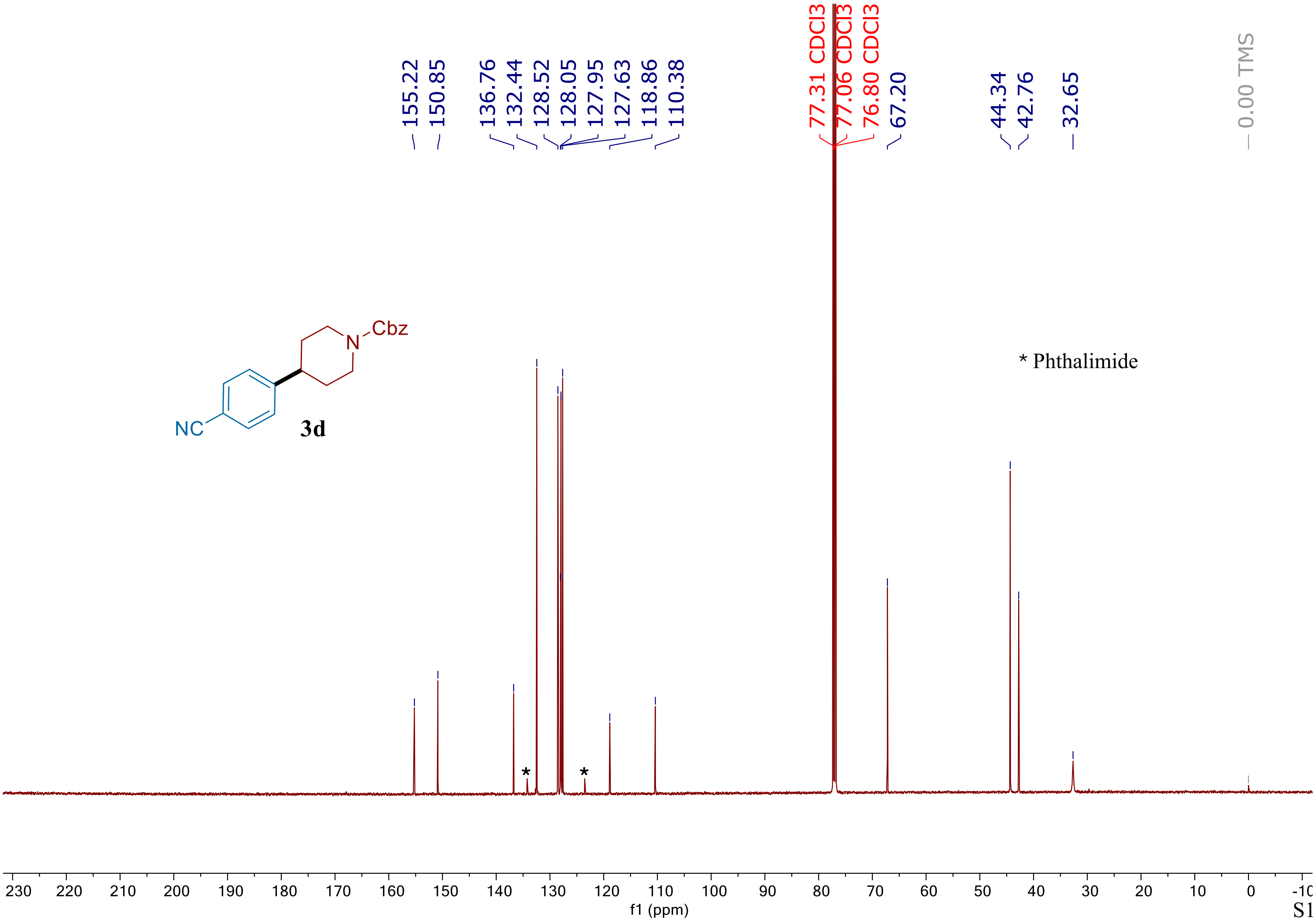

^{1}H NMR (500 MHz) of 3e in $CDCl_3$

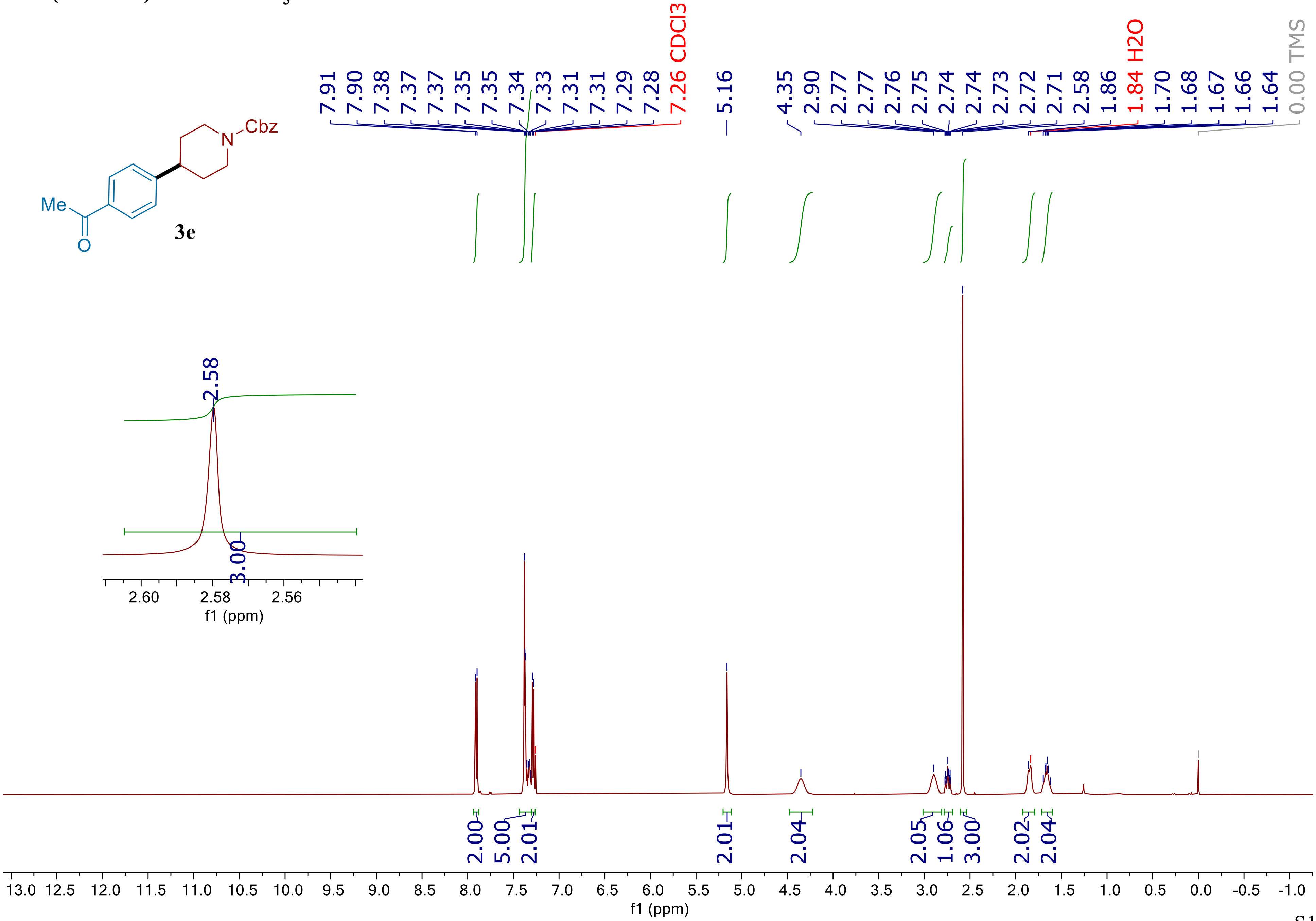

$^{13}C\{^1H\}$ NMR (126 MHz) of 3e in $CDCl_3$

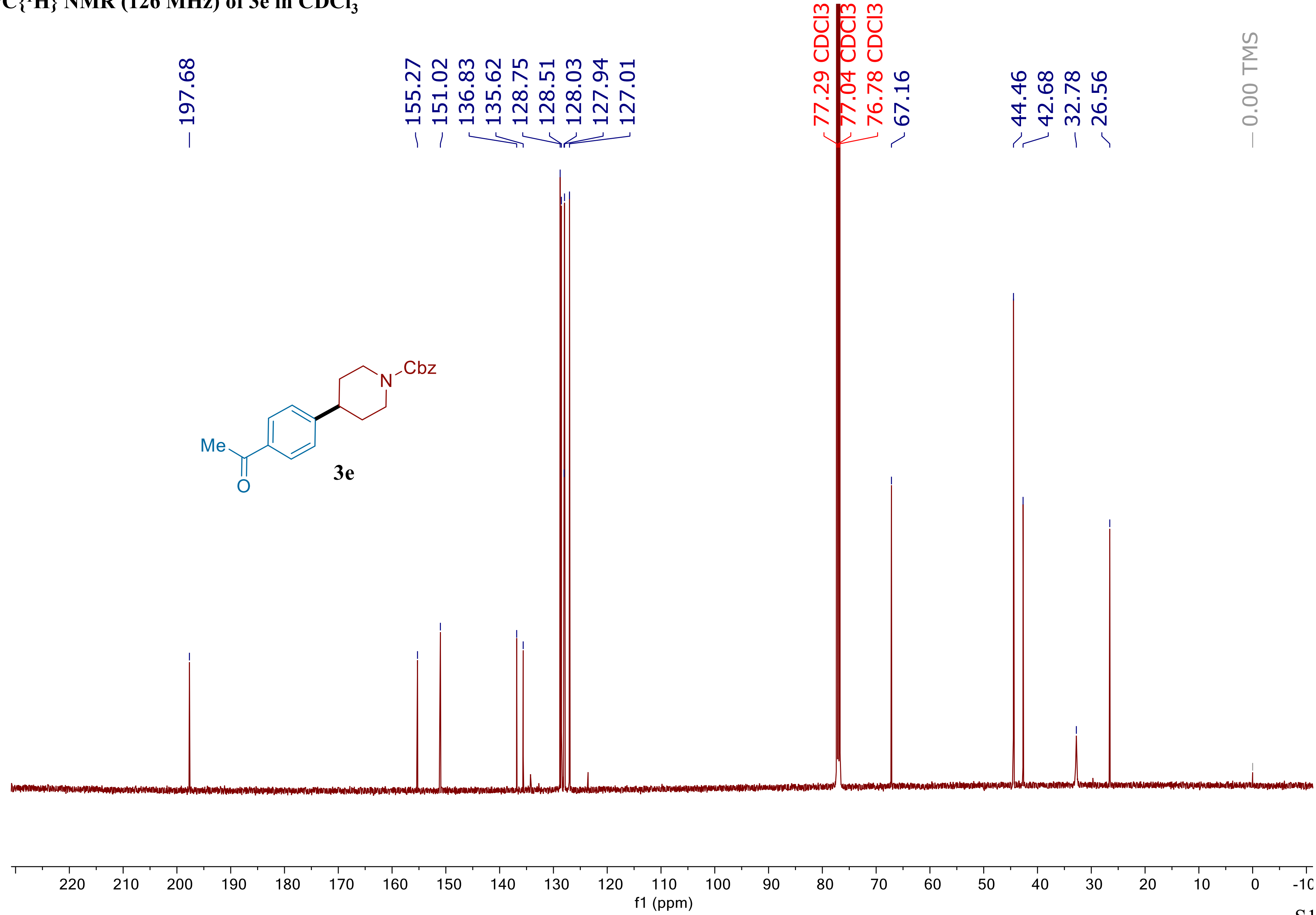

^{1}H NMR (500 MHz) of 3f in $CDCl_3$

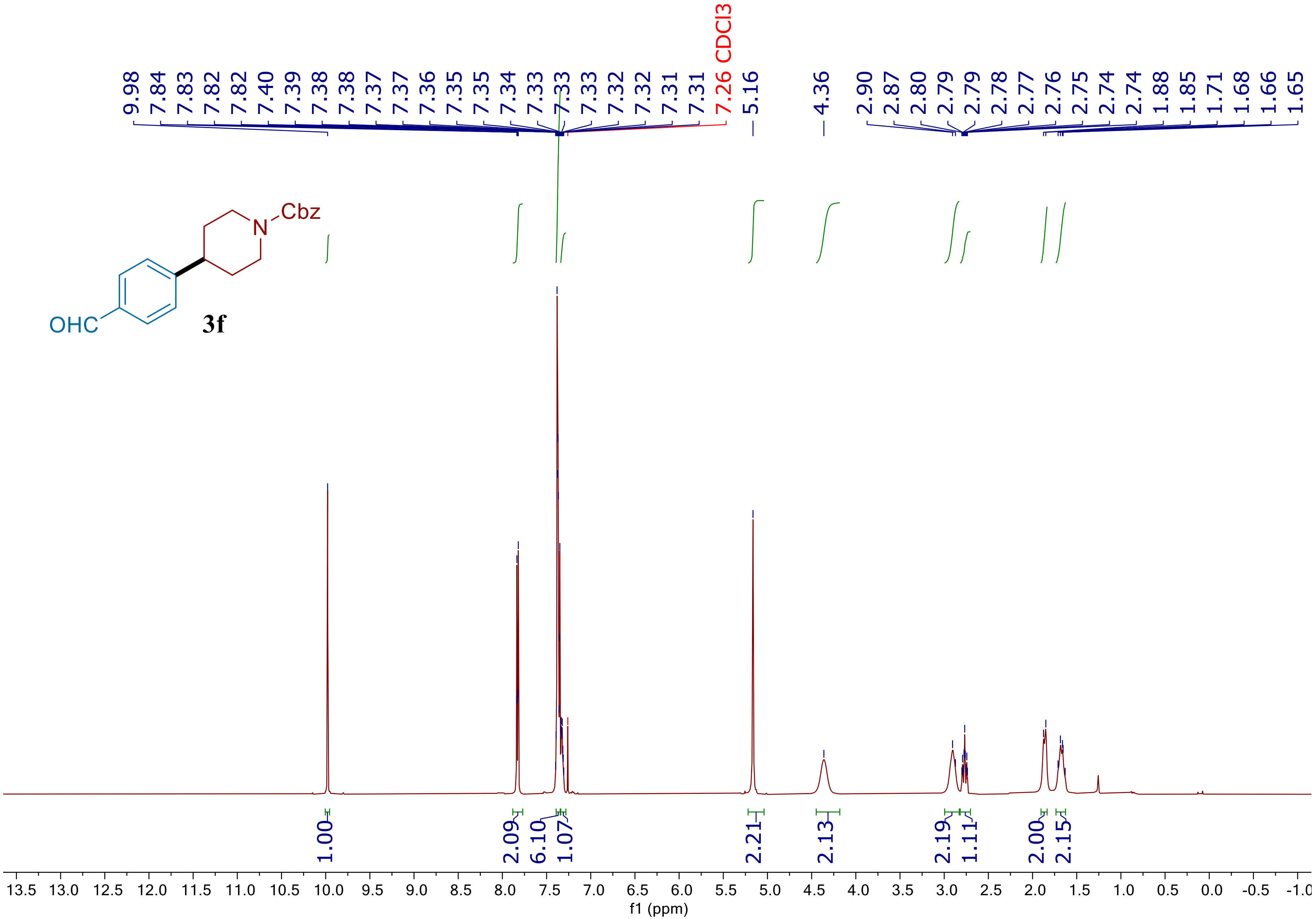

$^{13}C\{^1H\}$ NMR (126 MHz) of 3f in $CDCl_3$

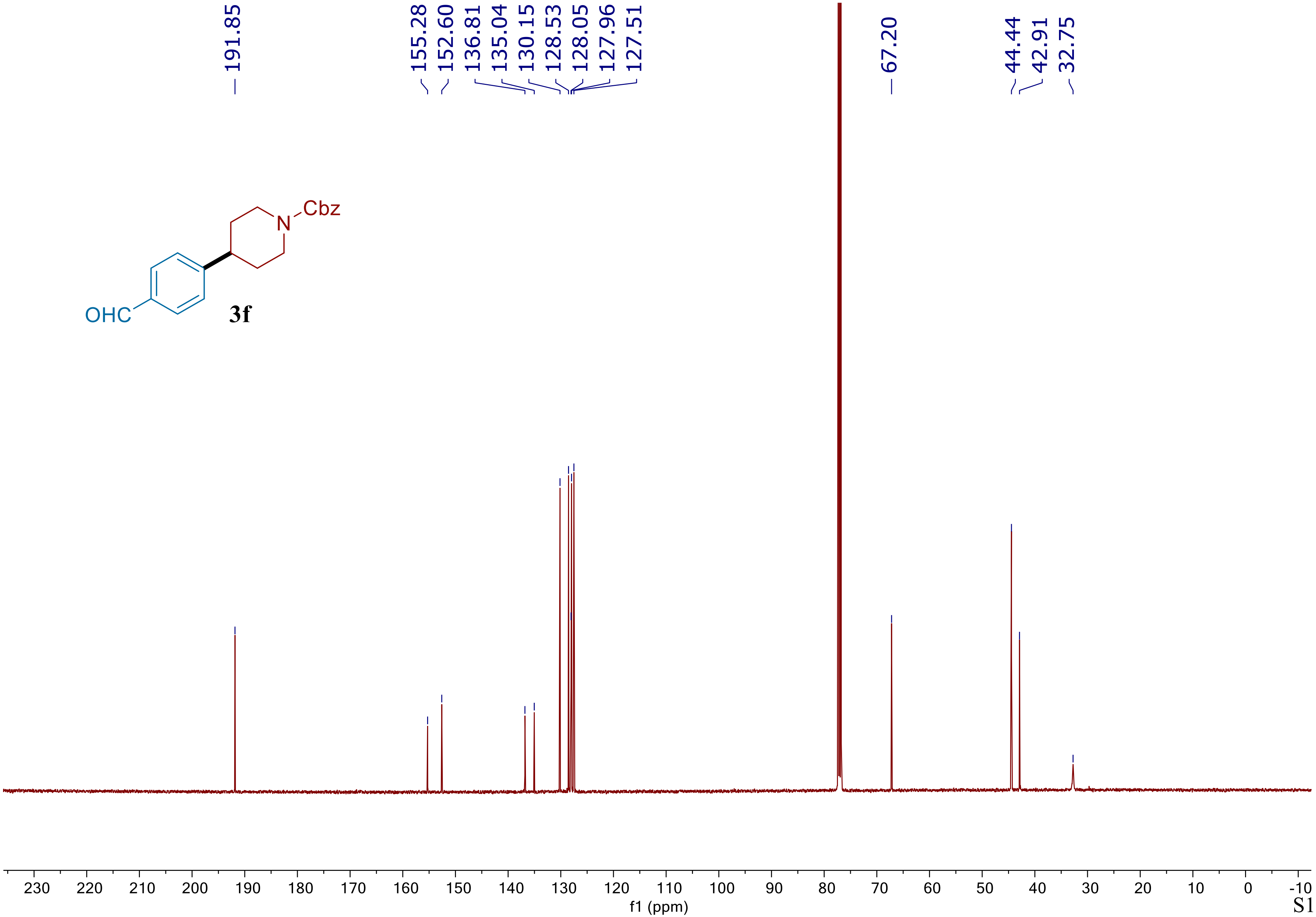

^{1}H NMR (400 MHz) of 3g in $CDCl_3$

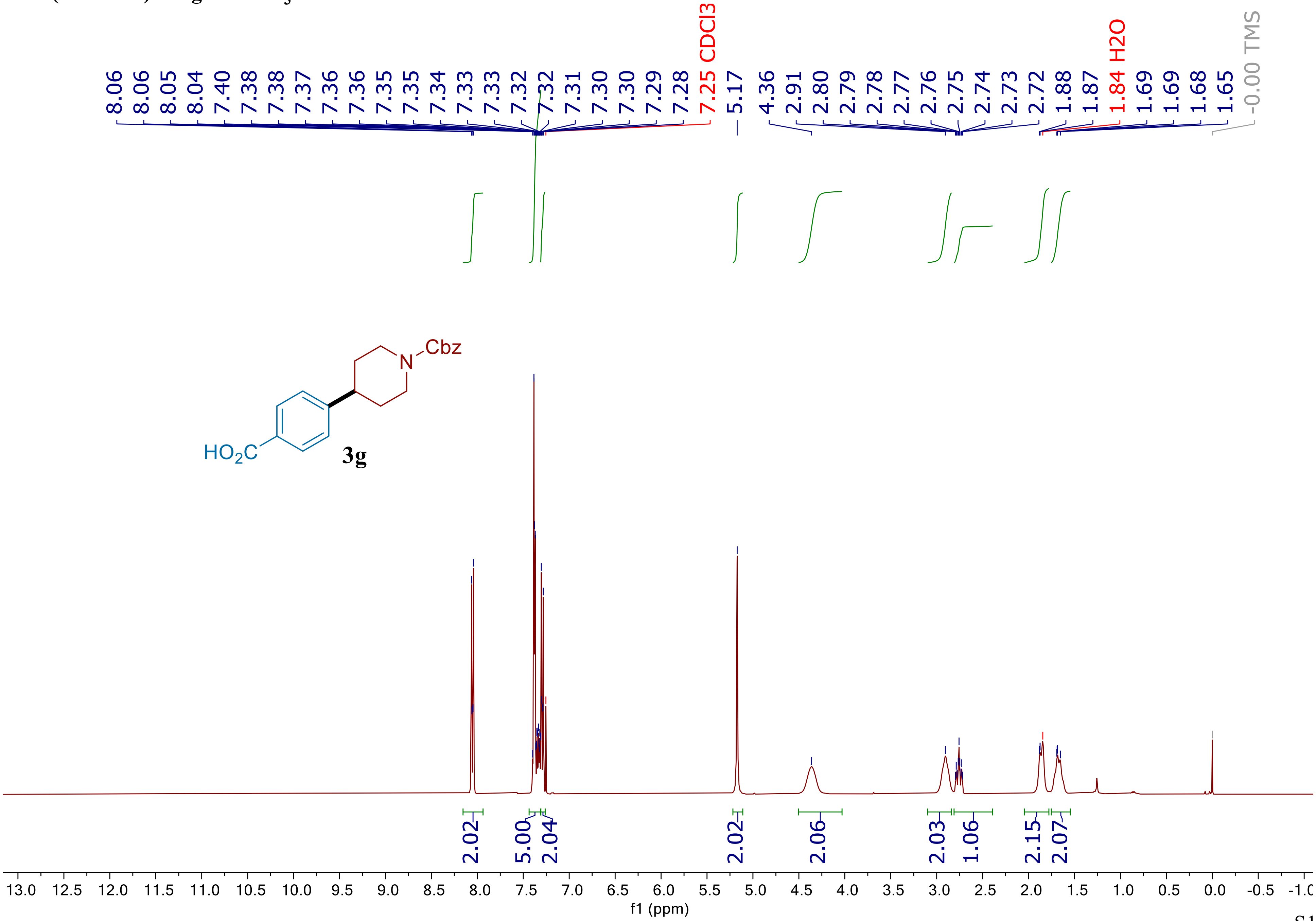

$^{13}C\{^{1}H\}$ NMR (126 MHz) of 3g in $CDCl_3$

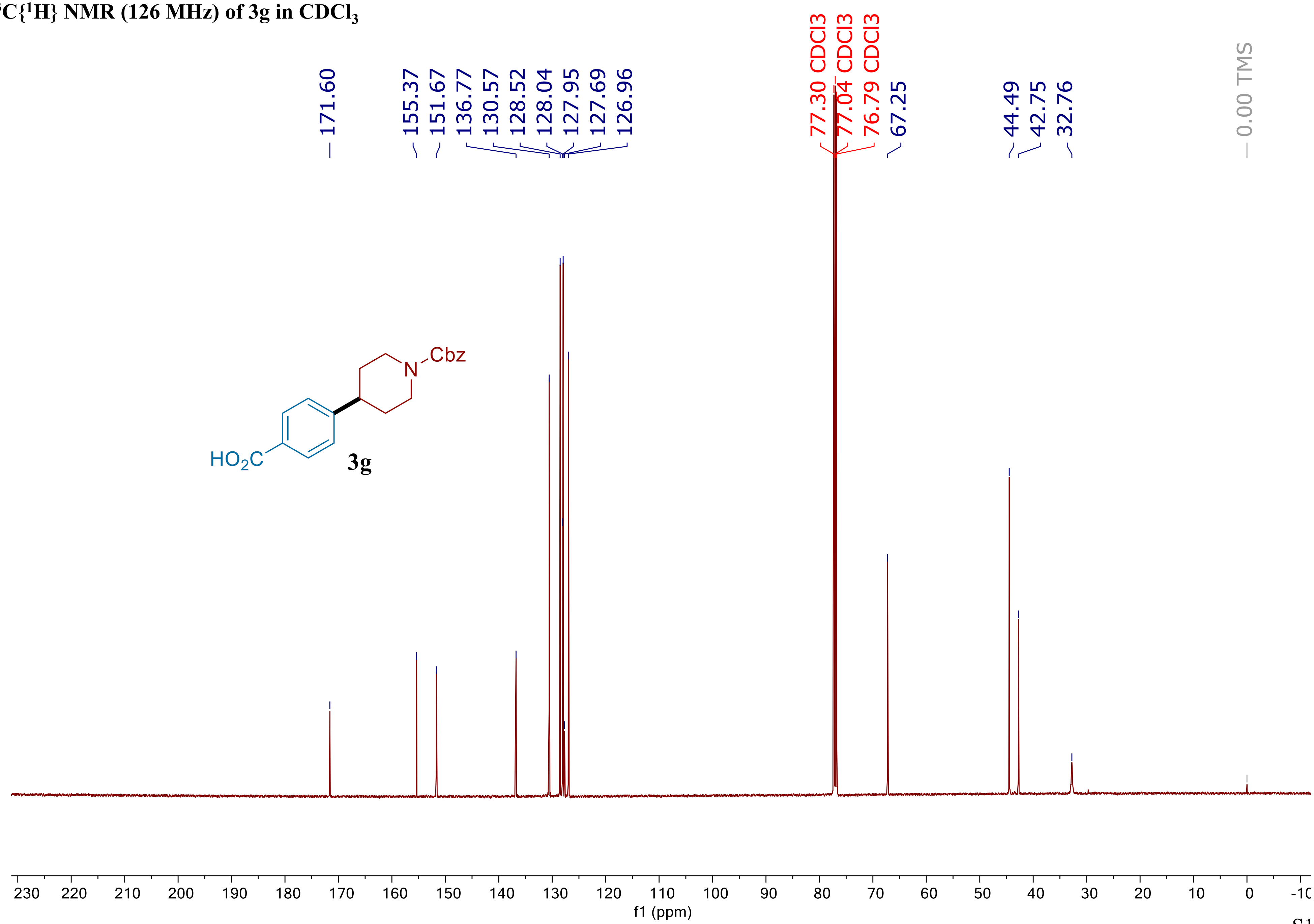

^{1}H NMR (400 MHz) of 3h in $CDCl_3$

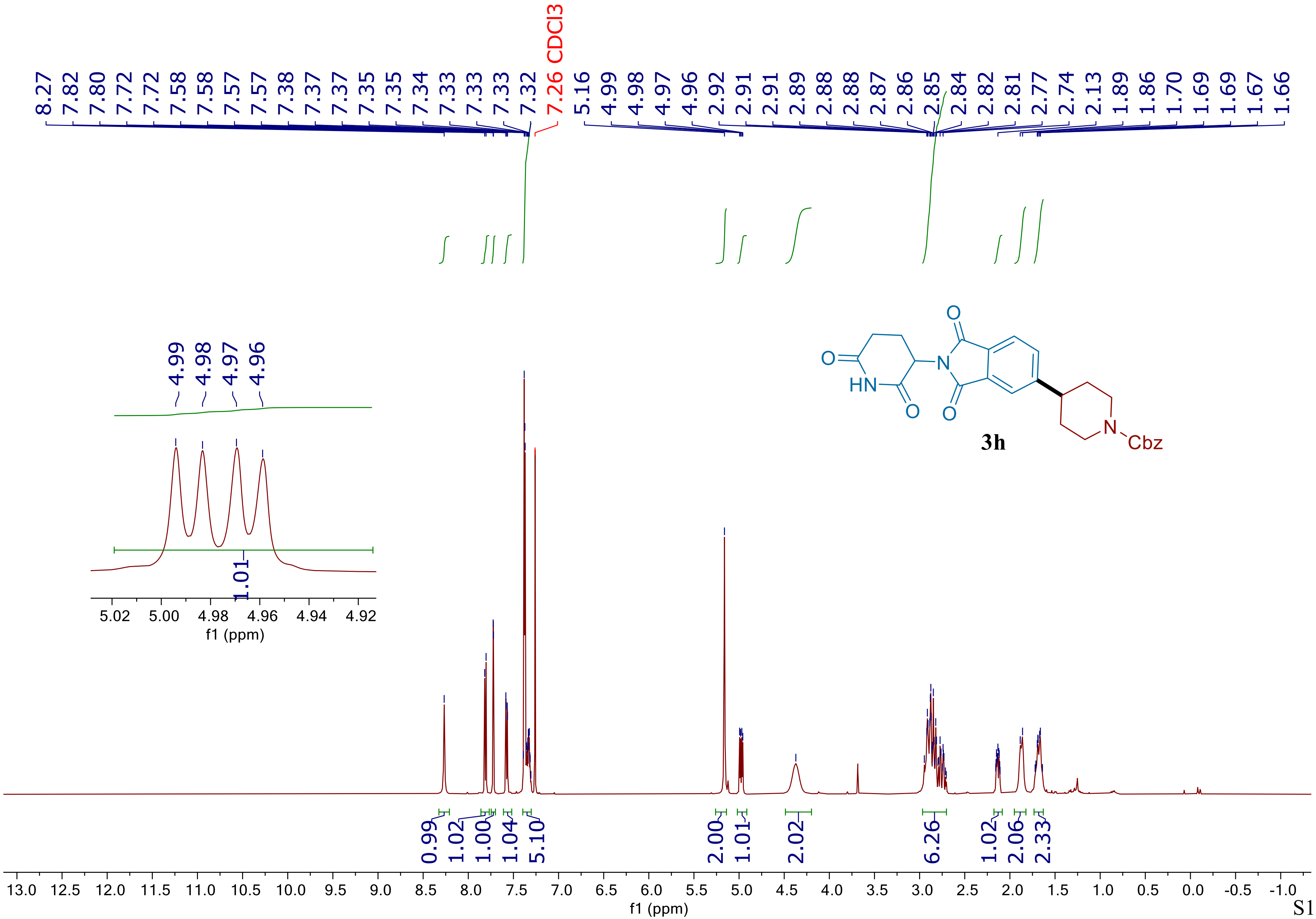

$^{13}C\{^1H\}$ NMR of 3h (126 MHz) in $CDCl_3$

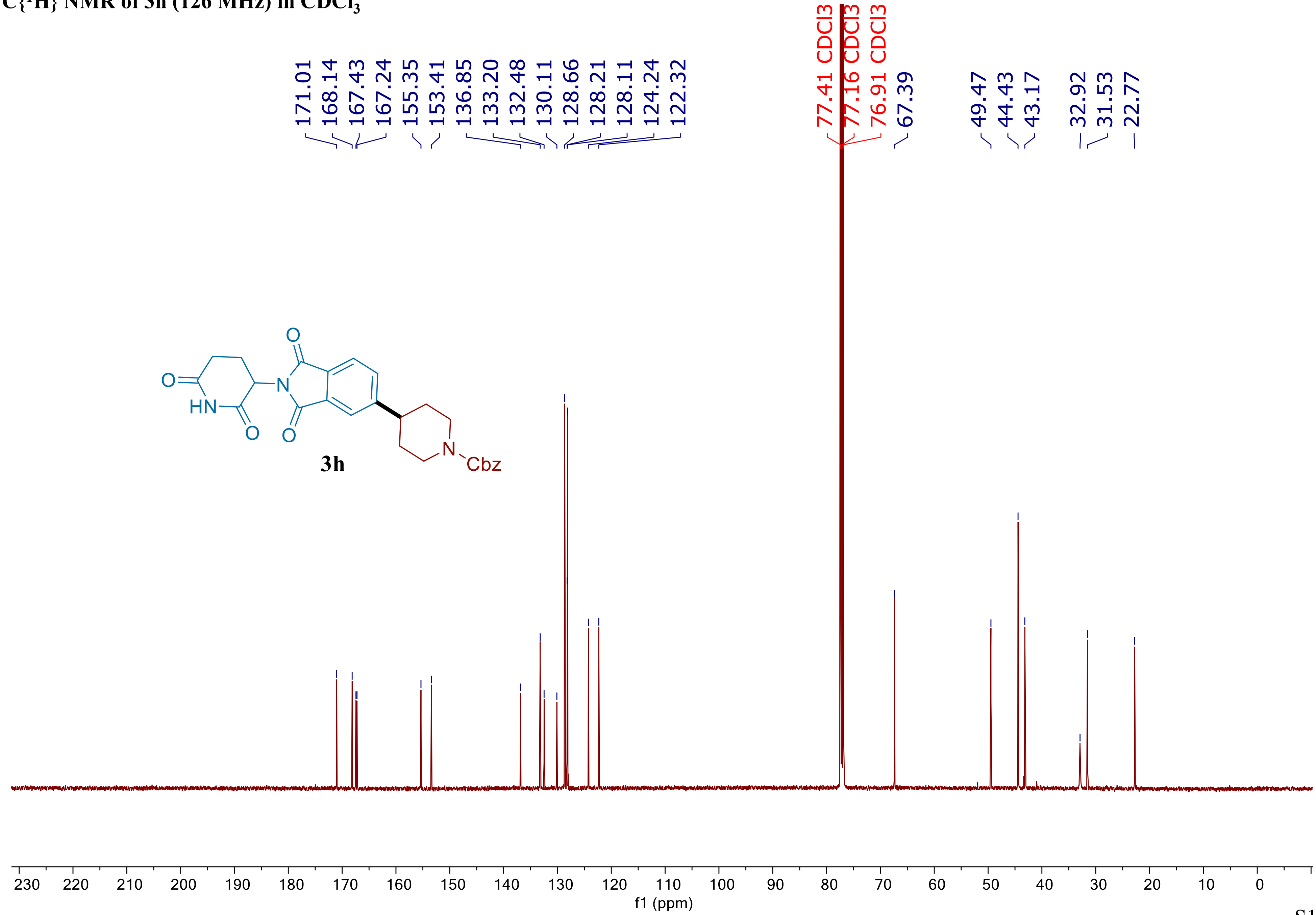

^{1}H NMR (500 MHz) of 3i in DMSO-D$_6$

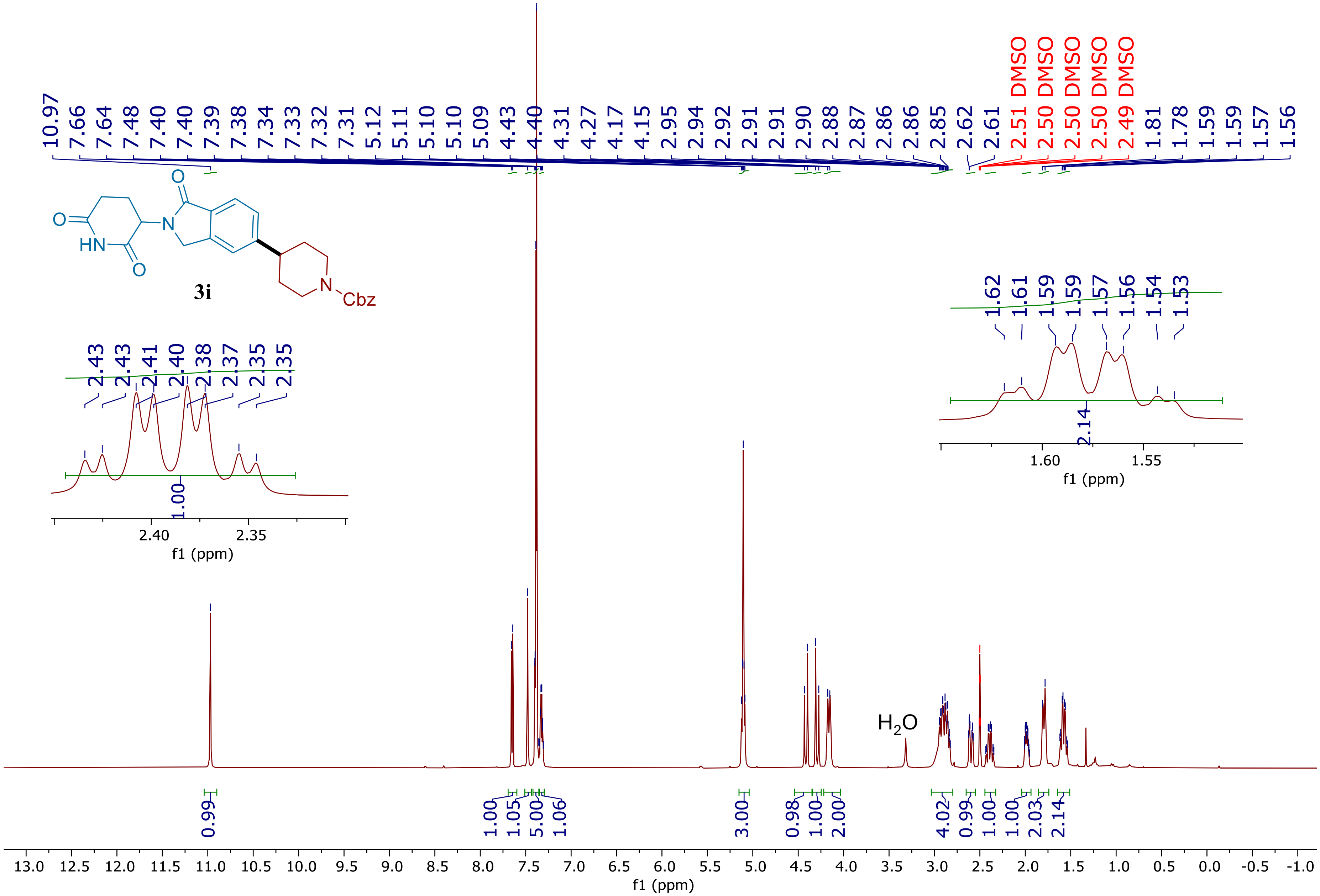

$^{13}C\{^{1}H\}$ NMR (126 MHz) of 3i in DMSO-D_6

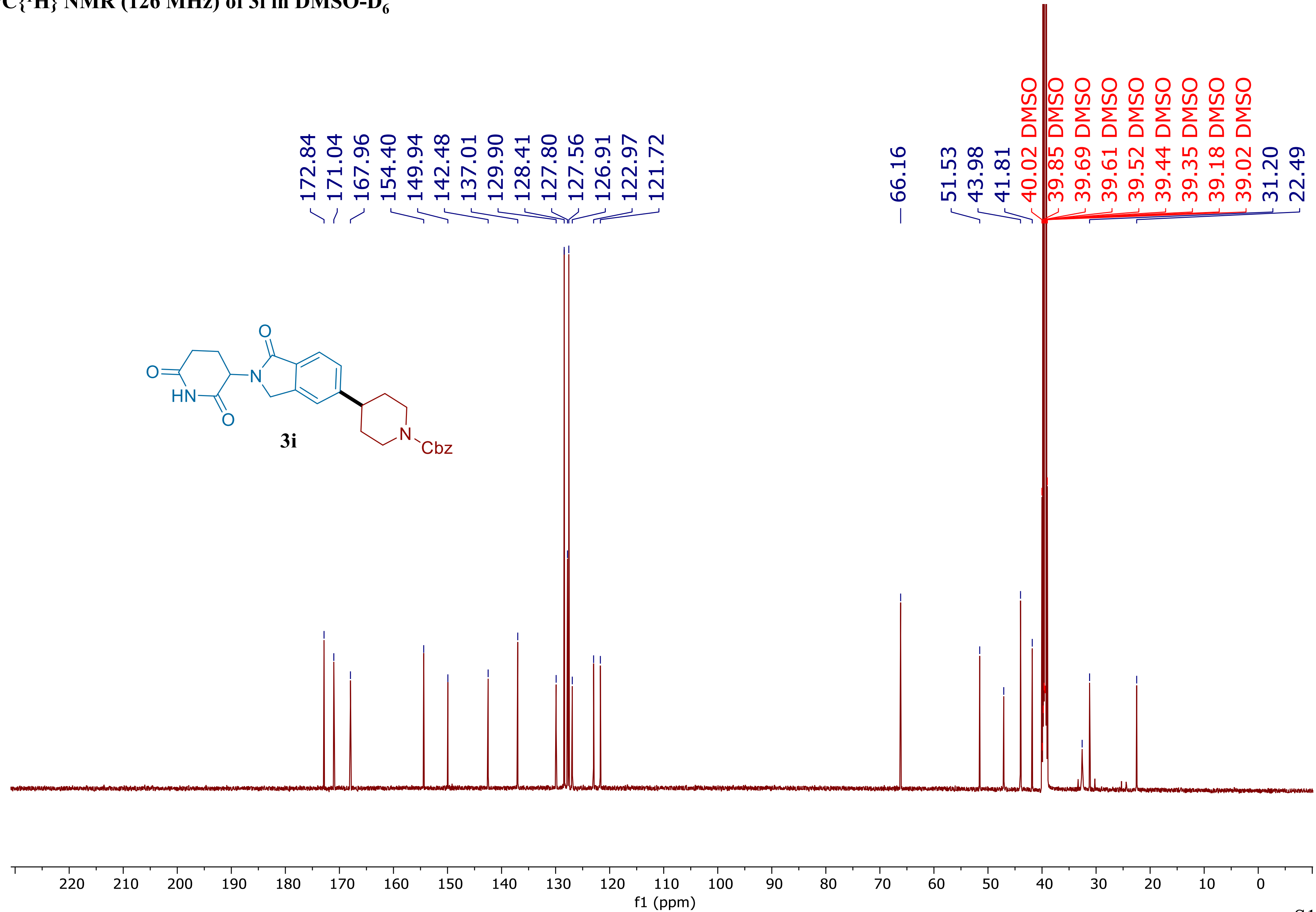

^{1}H NMR (500 MHz) of 3j in $CDCl_3$

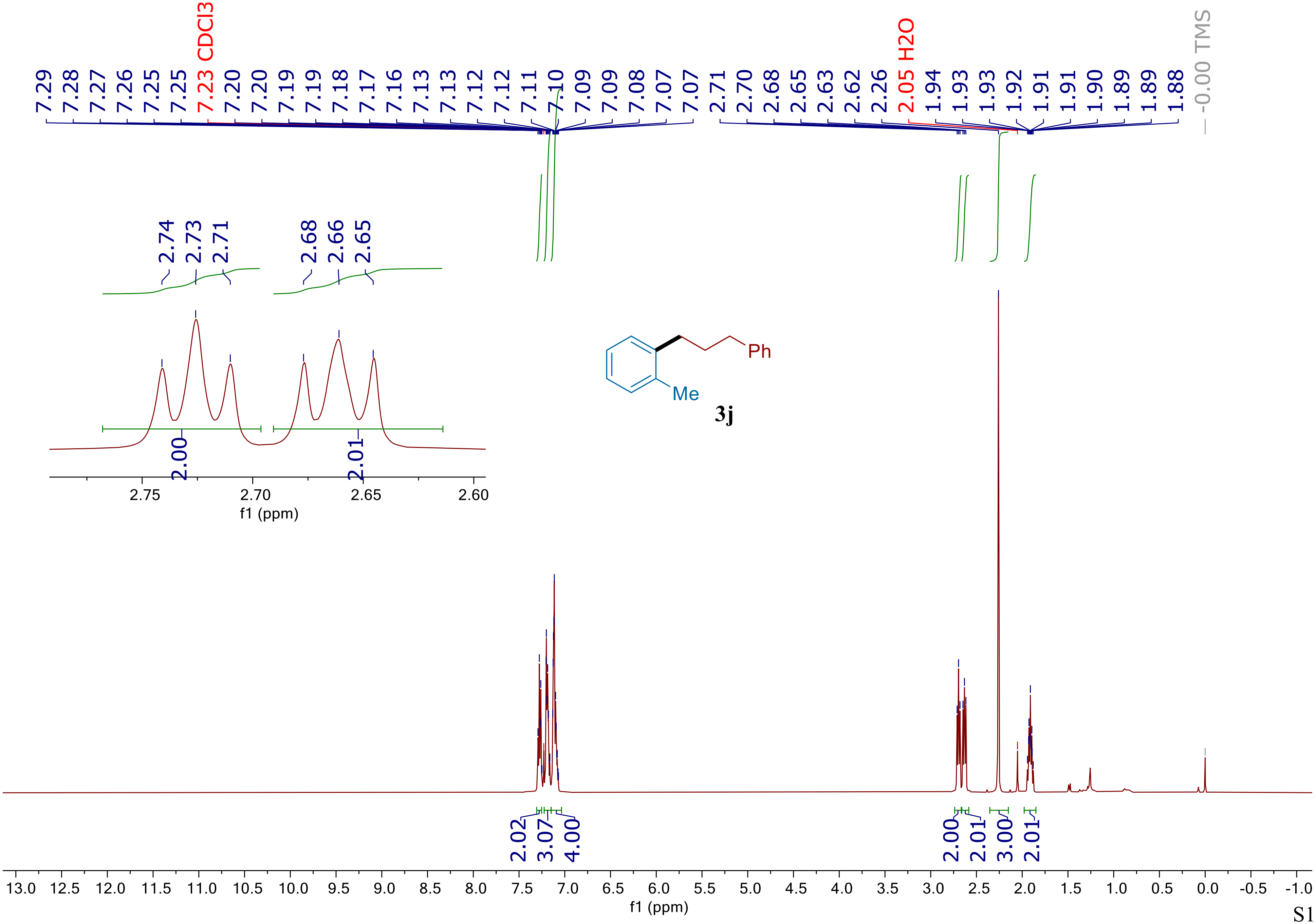

$^{13}C\{^{1}H\}$ NMR (126 MHz) of 3j in $CDCl_3$

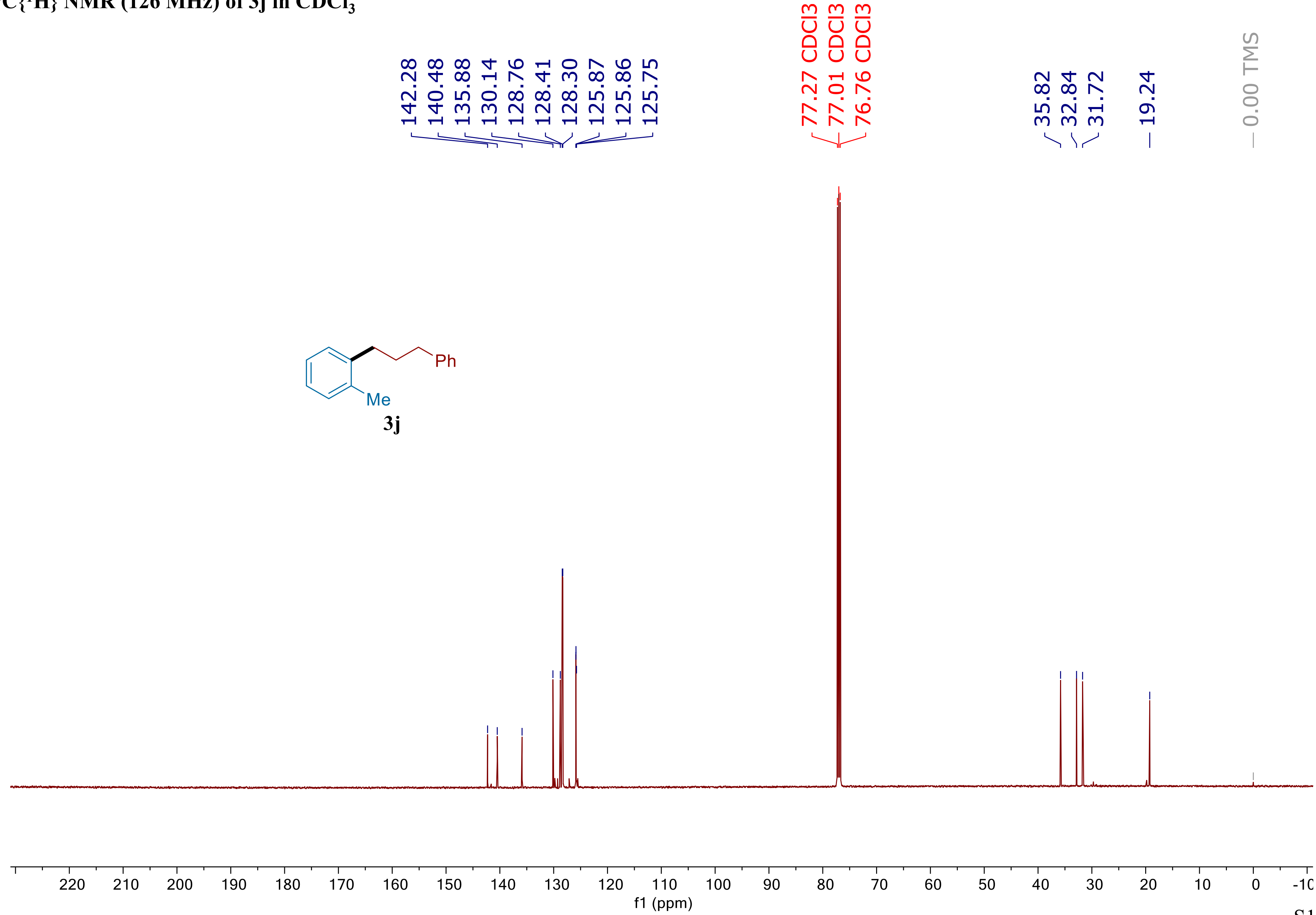

3j

^{1}H NMR (500 MHz) of 3k in $CDCl_3$

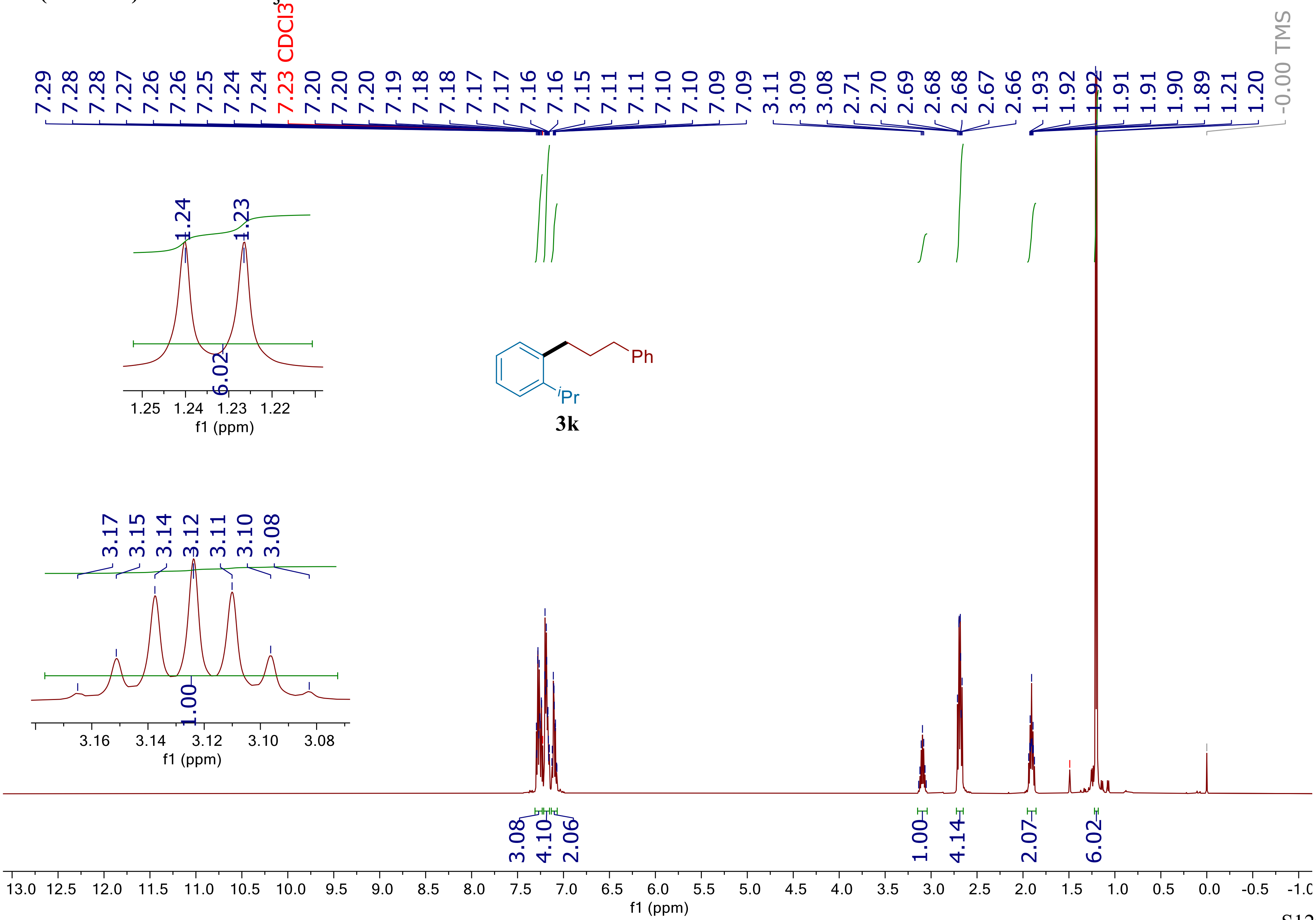

$^{13}C\{^{1}H\}$ NMR (126 MHz) of 3k in $CDCl_3$

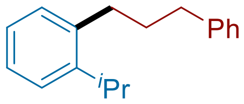

3k

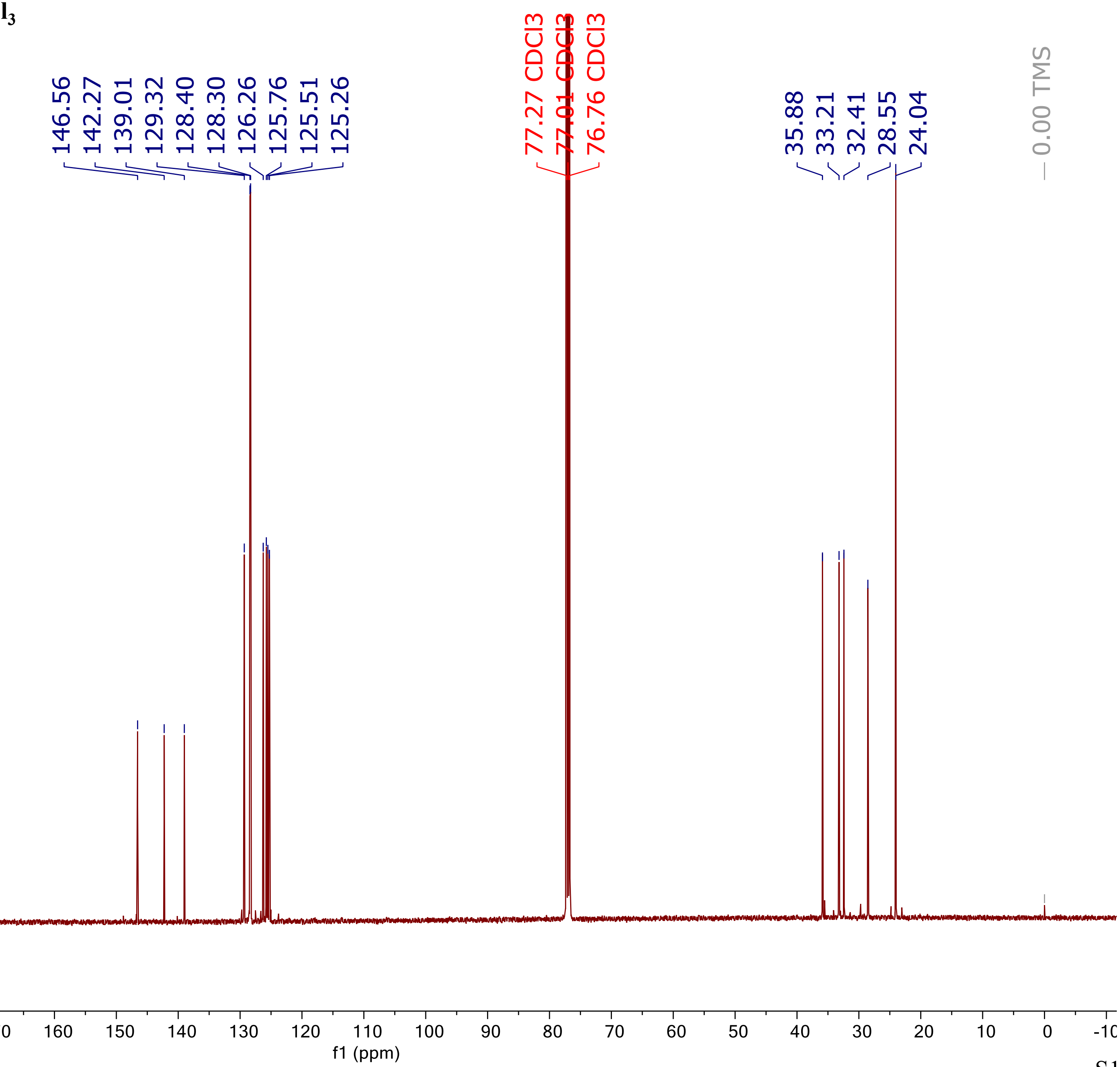

^1H NMR (500 MHz) of 3m in $CDCl_3$

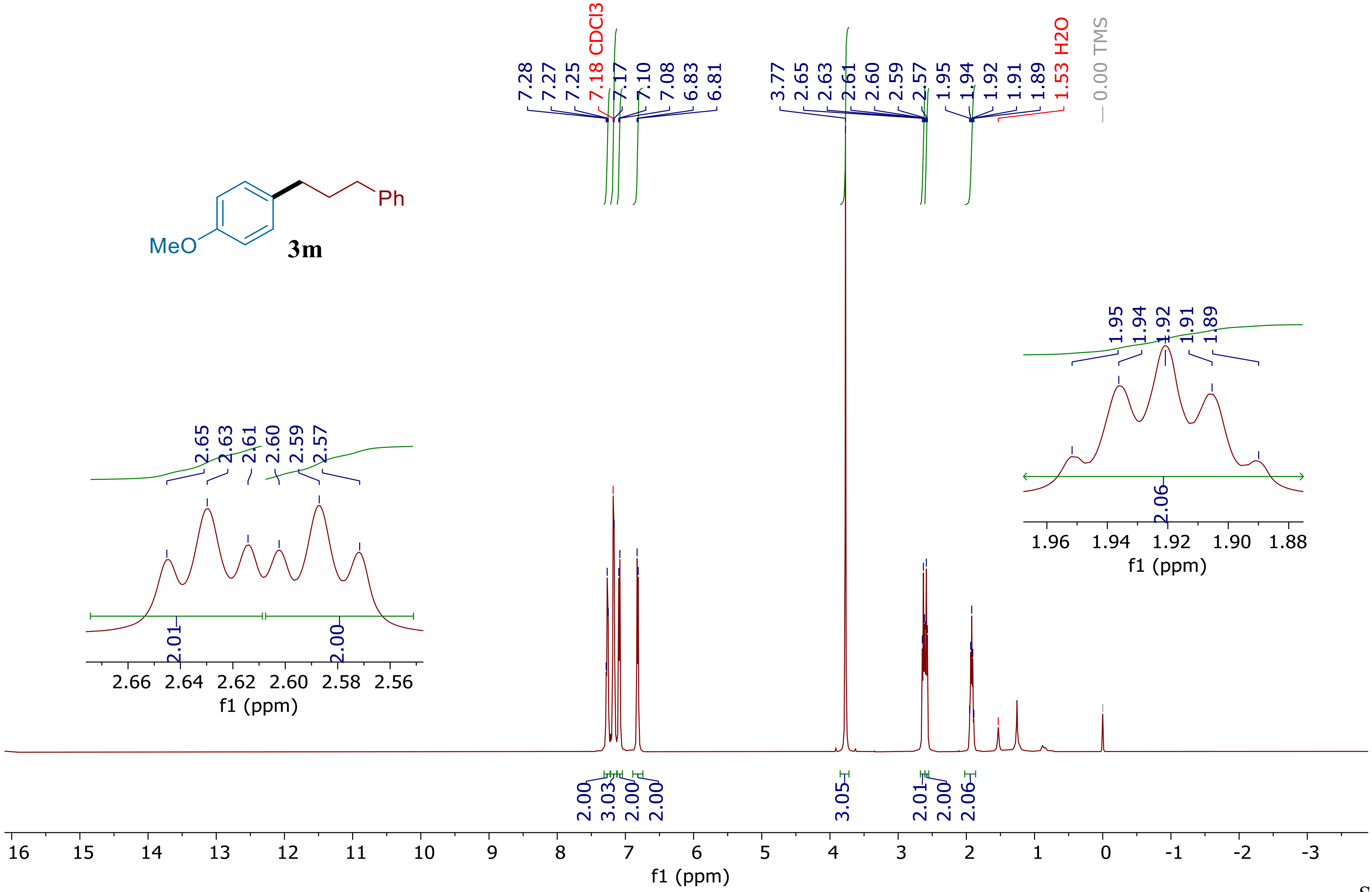

$^{13}C\{^1H\}$ NMR (126 MHz) of 3m in $CDCl_3$

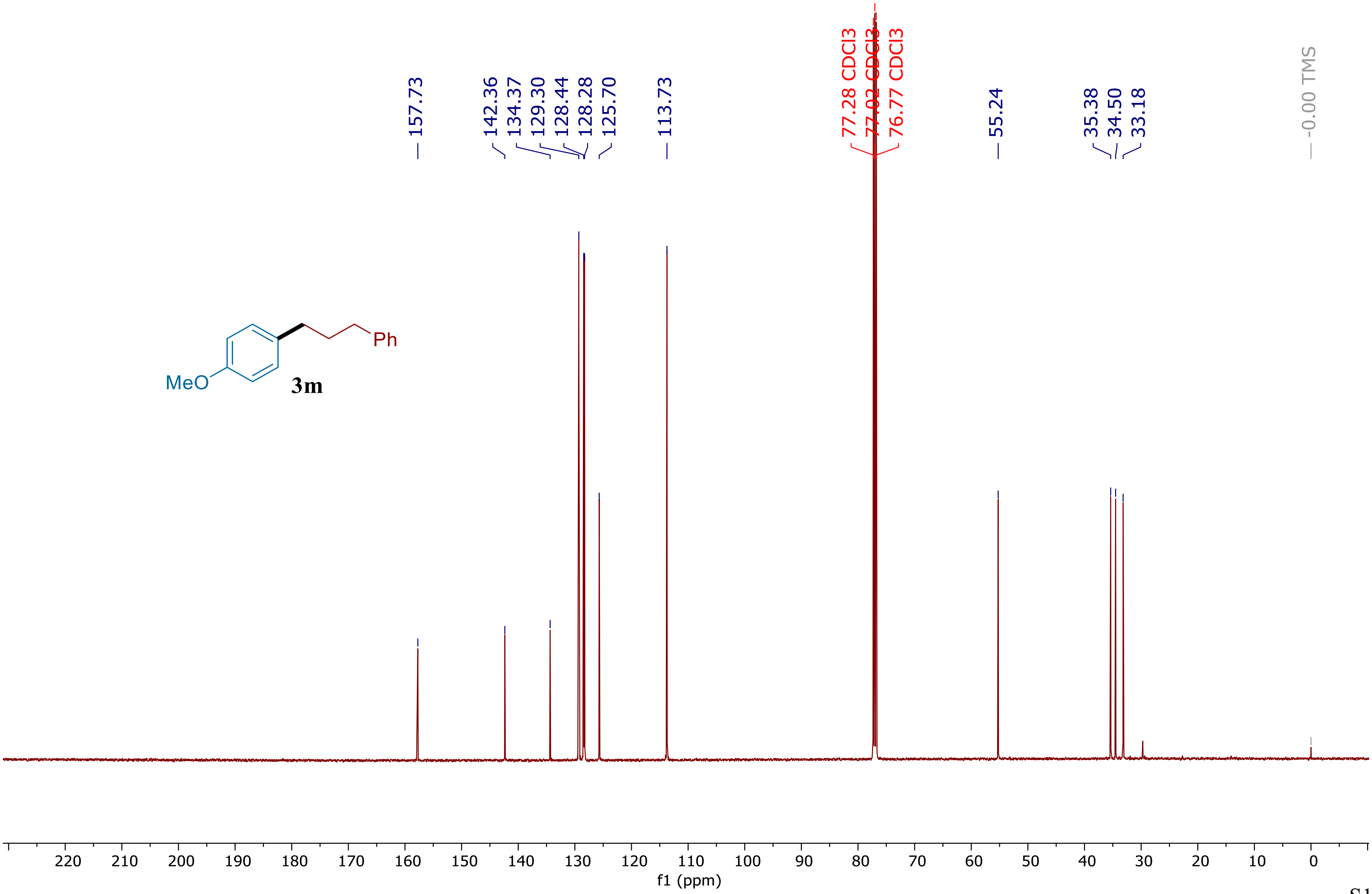

^{1}H NMR (500 MHz) of 3n in $CDCl_3$

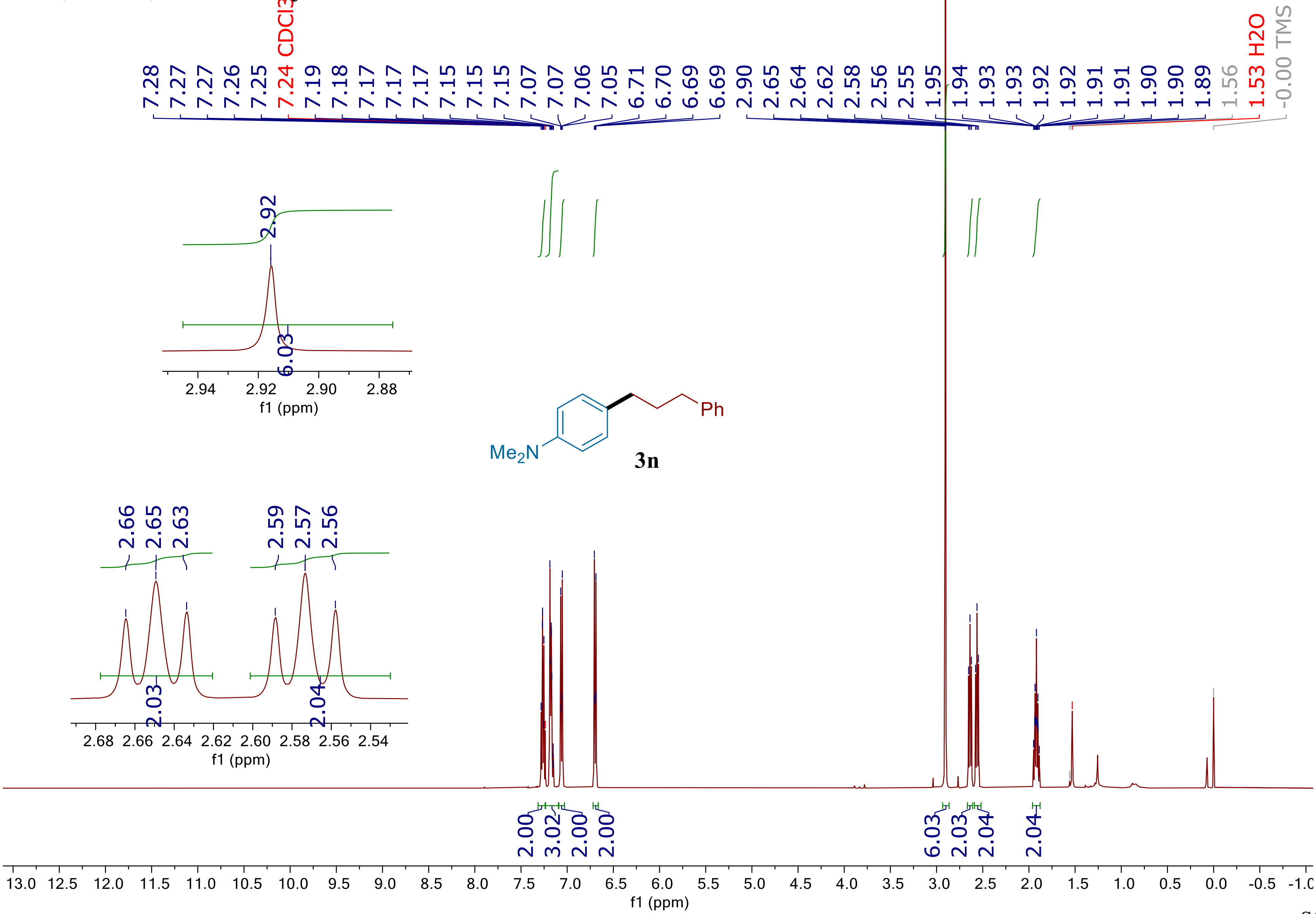

$^{13}C\{^{1}H\}$ NMR (126 MHz) of 3n in $CDCl_3$

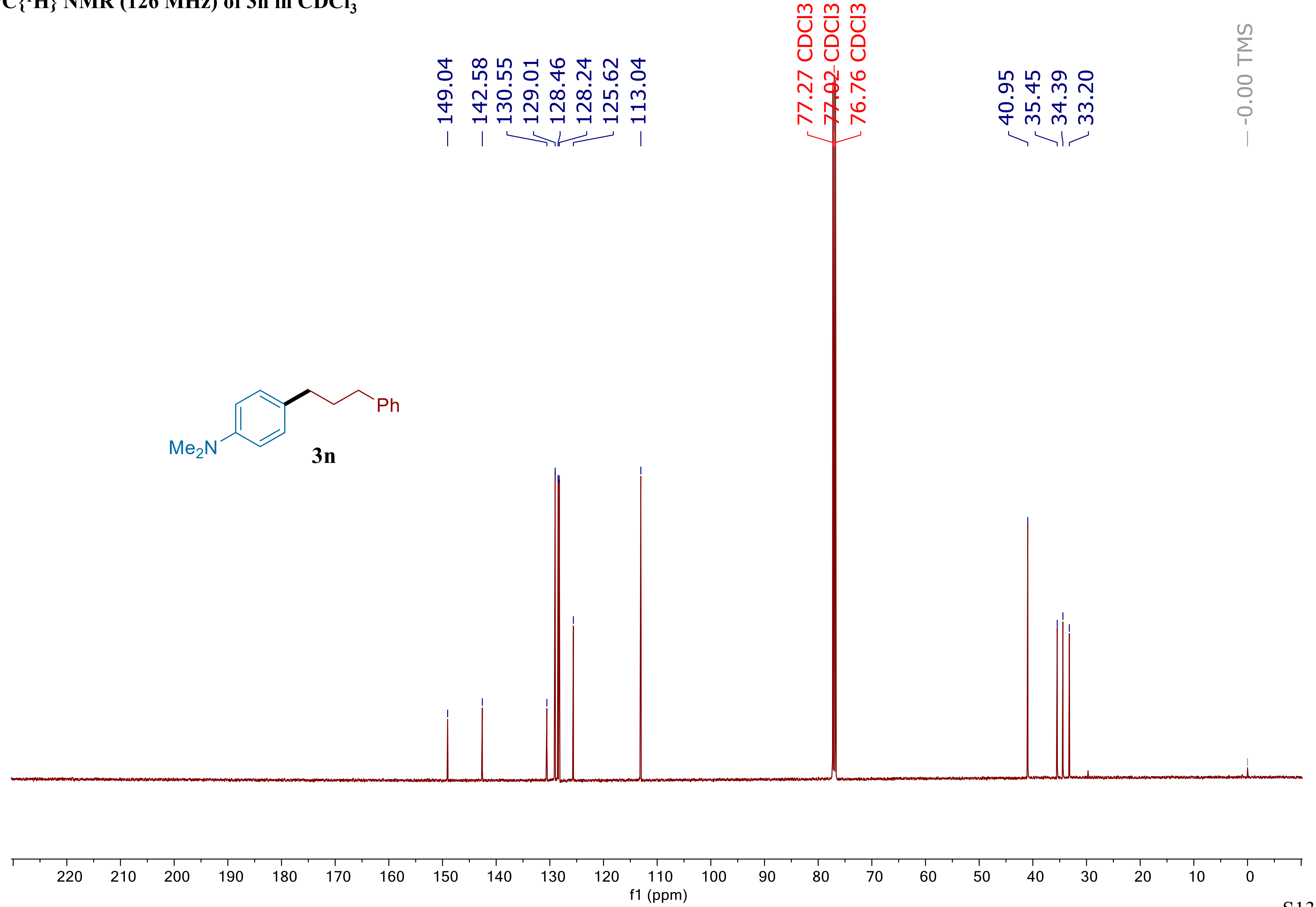

^{1}H NMR (500 MHz) of 3o in $CDCl_3$

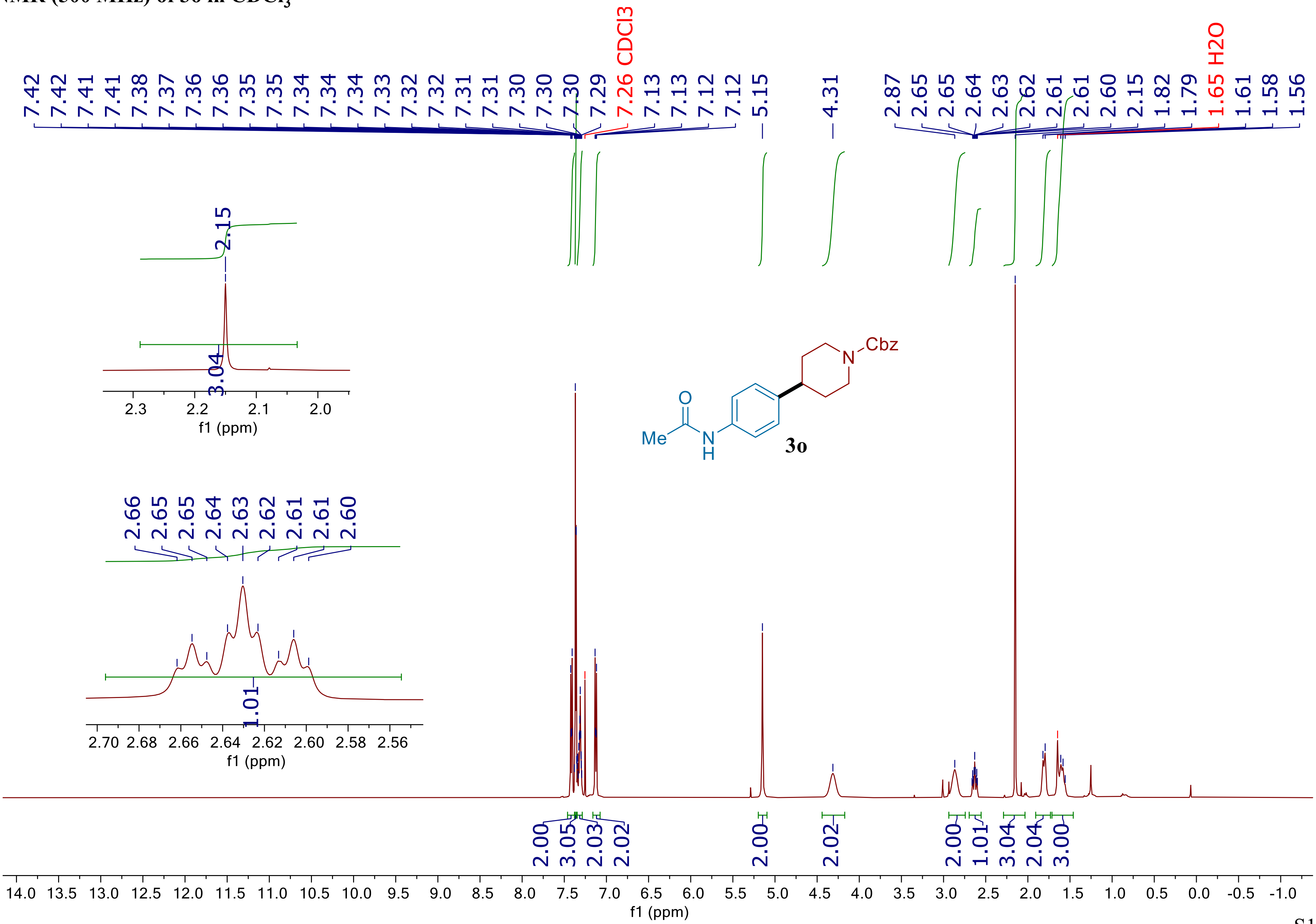

$^{13}C\{^{1}H\}$ NMR (126 MHz) of 3o in $CDCl_3$

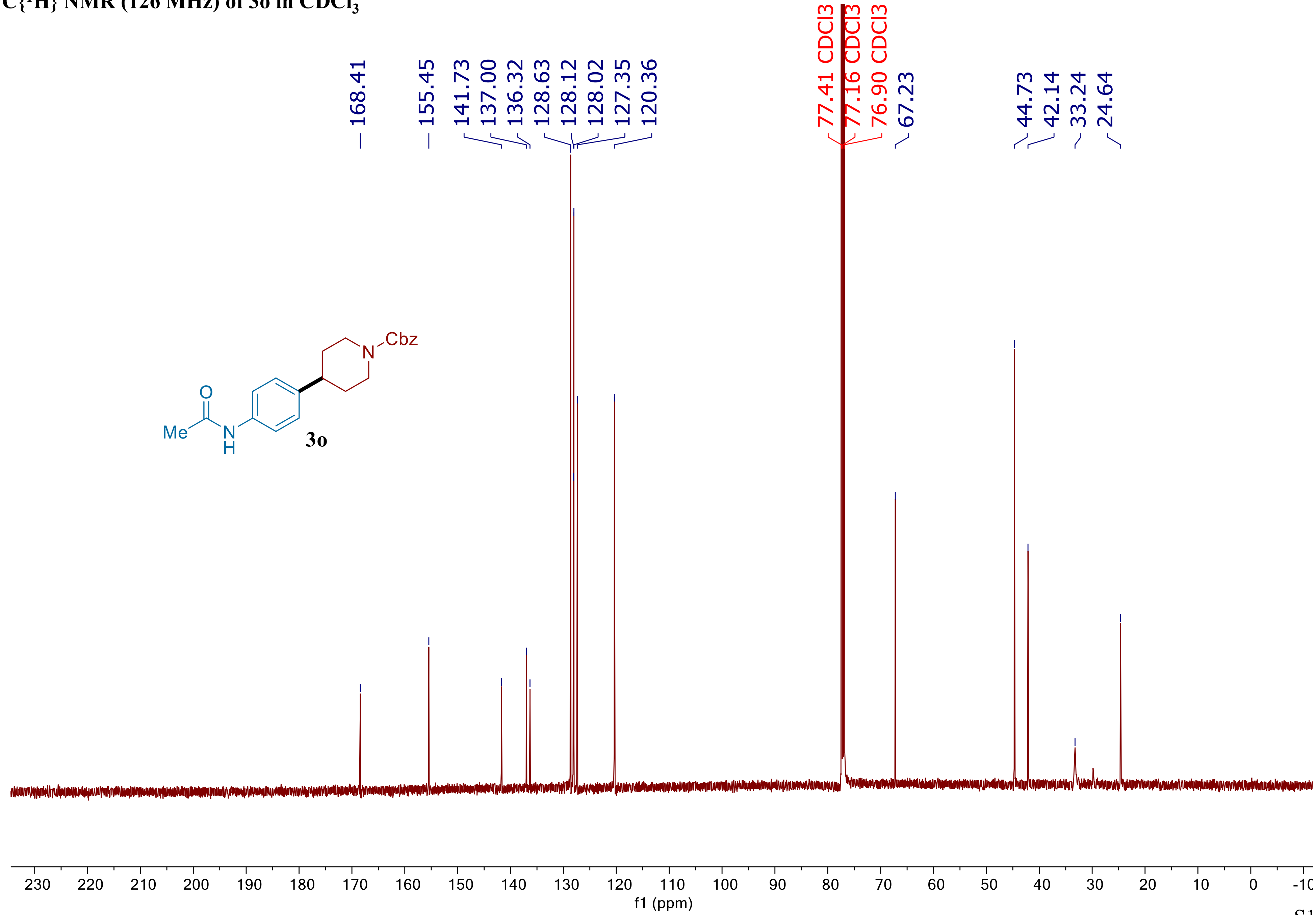

^{1}H NMR (500 MHz) of 3p in $CDCl_3$

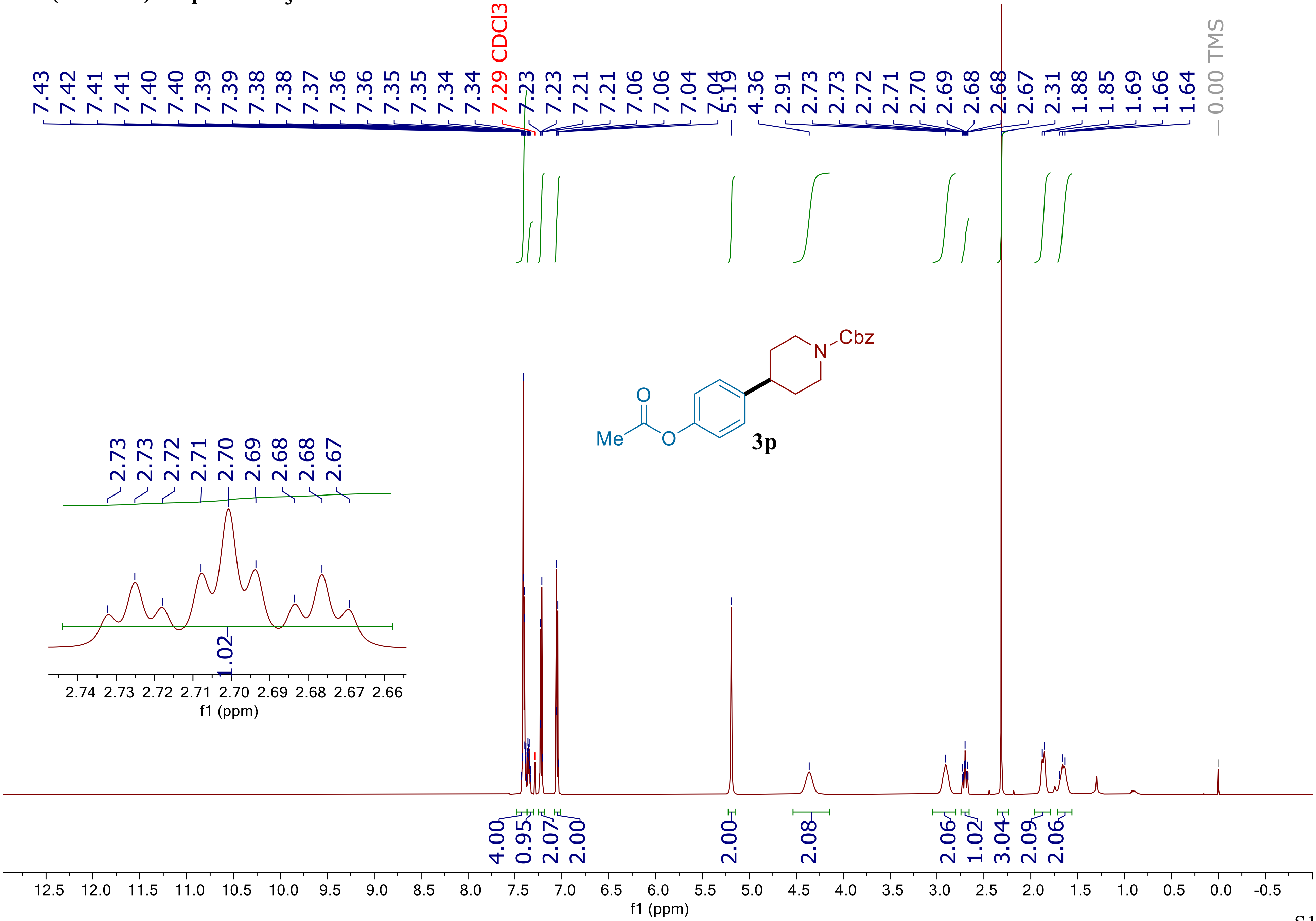

$^{13}C\{^1H\}$ NMR (126 MHz) of 3p in $CDCl_3$

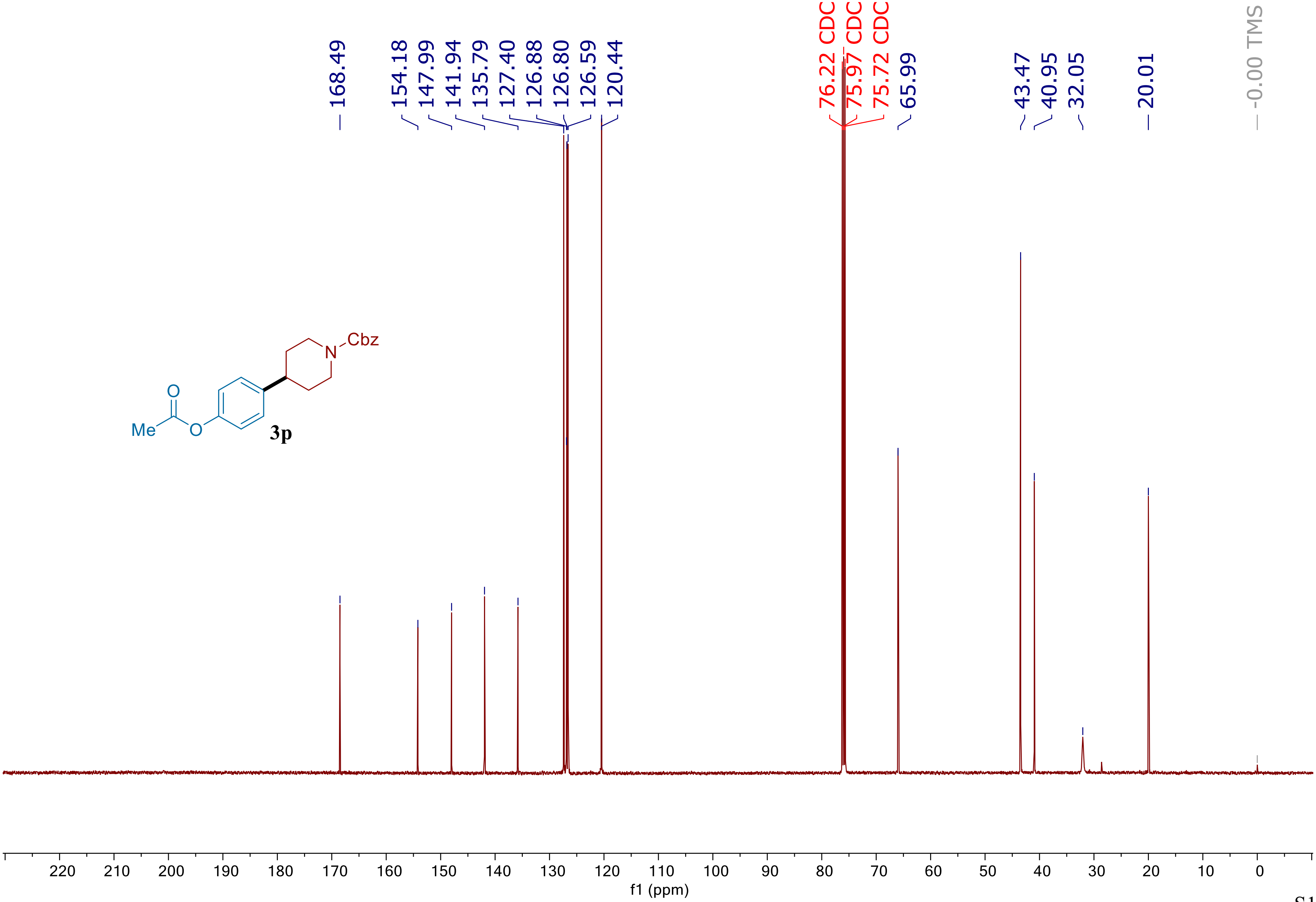

3p

^{1}H NMR (500 MHz) of 3q in $CDCl_3$

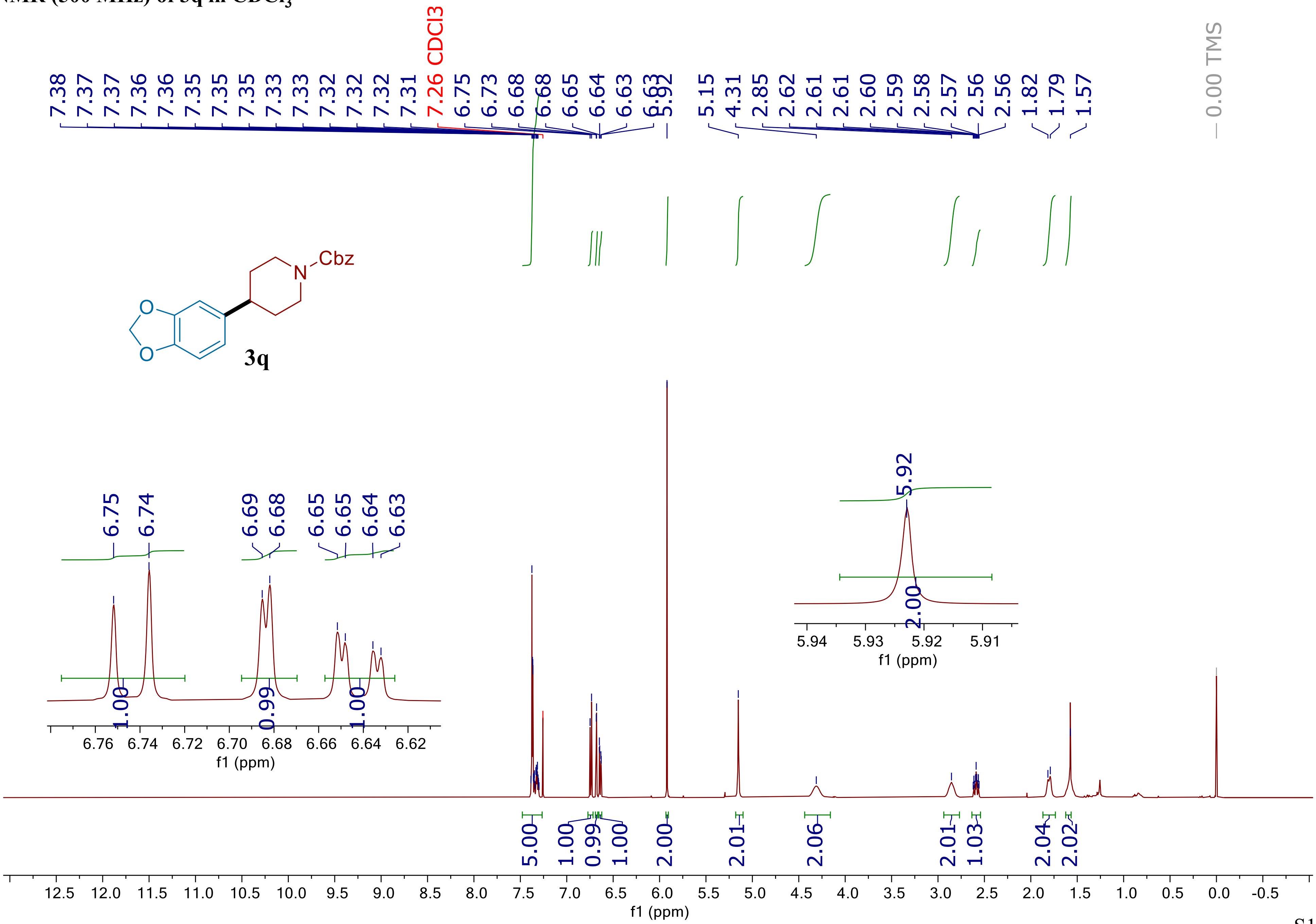

$^{13}C\{^1H\}$ NMR (126 MHz) of 3q in $CDCl_3$

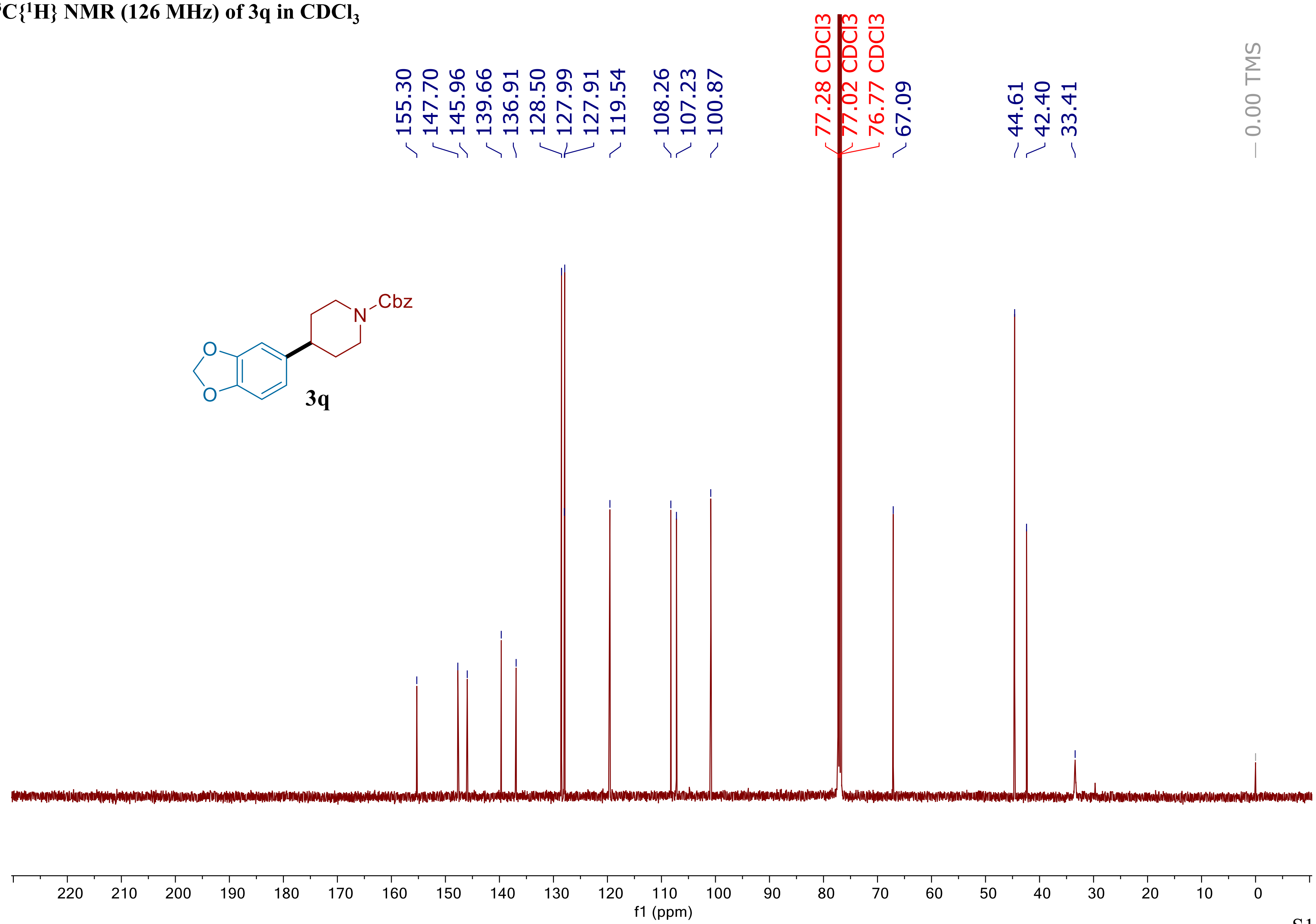

^{1}H NMR (500 MHz) of 3r in $CDCl_3$

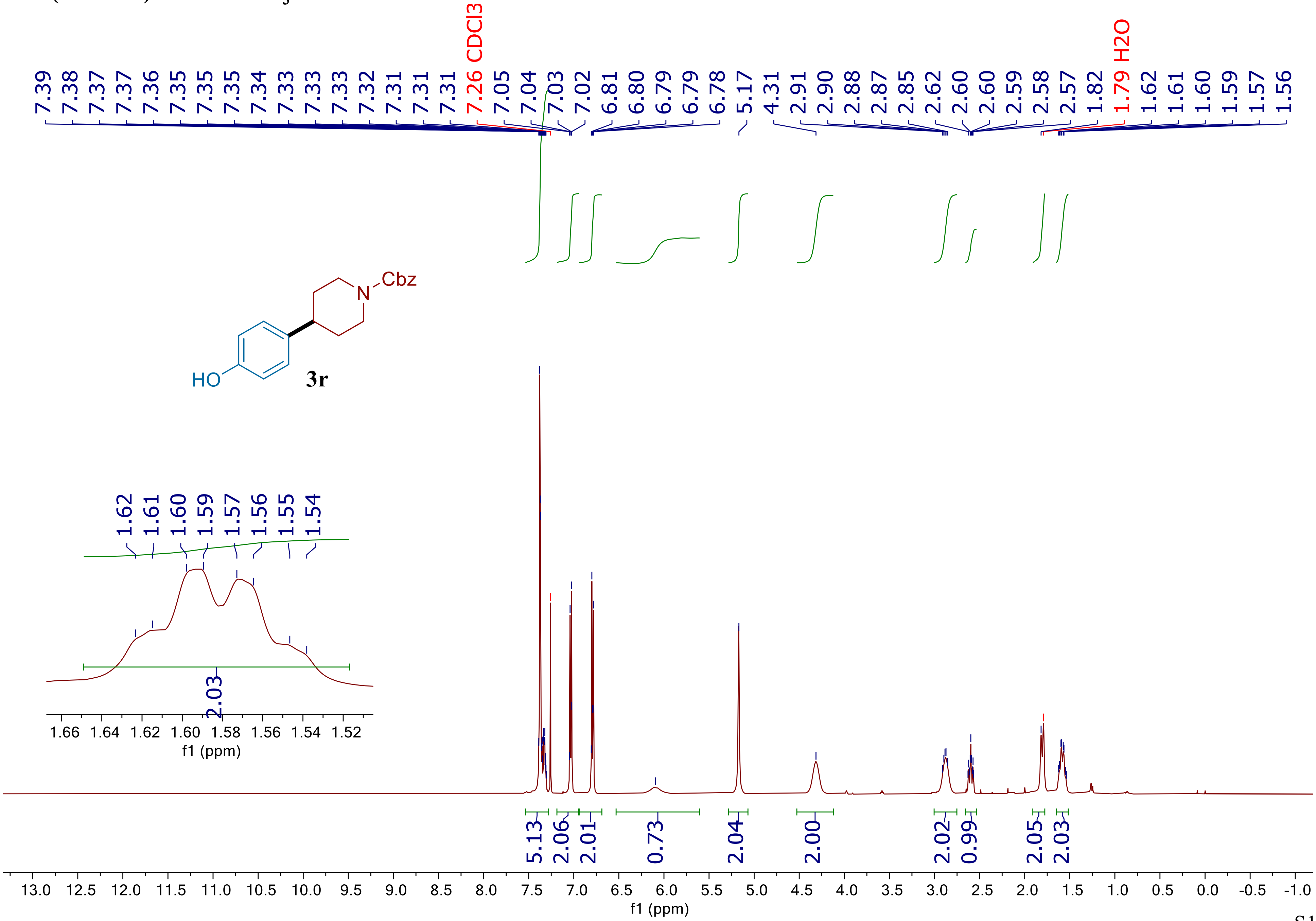

$^{13}C\{^1H\}$ NMR (126 MHz) of 3r in $CDCl_3$

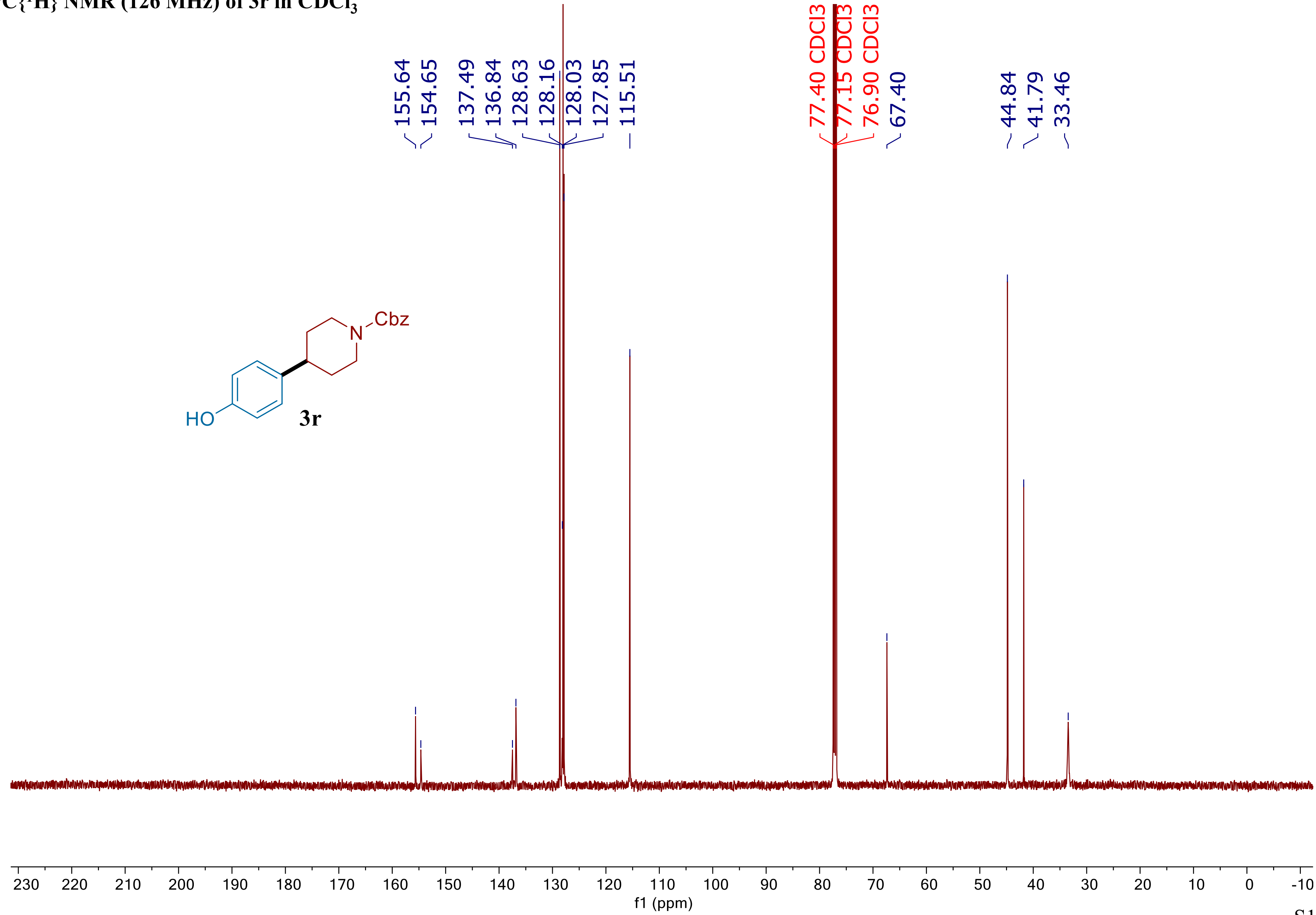

^1H NMR (500 MHz) of 3s in $CDCl_3$

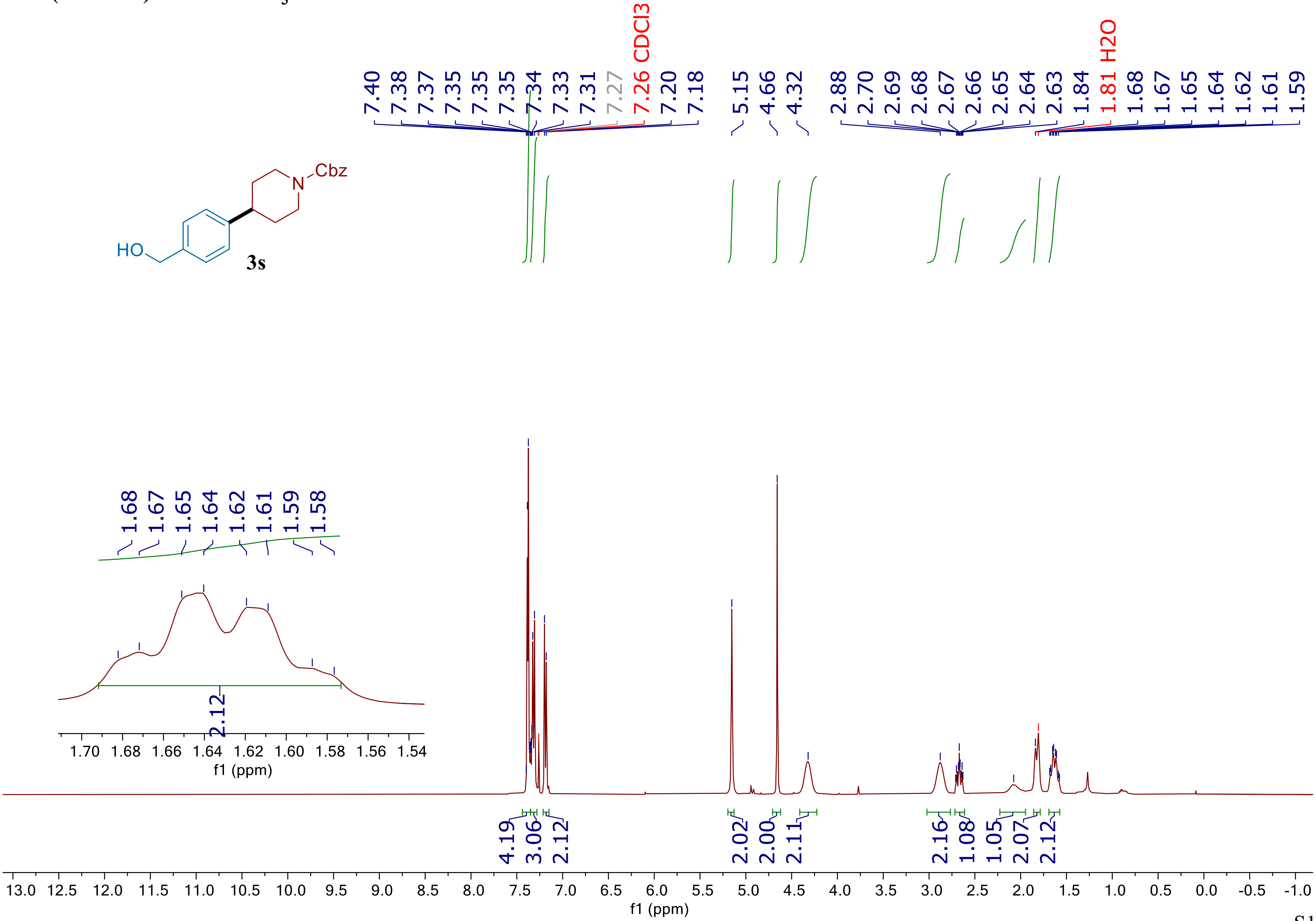

$^{13}C\{^1H\}$ NMR (126 MHz) of 3s in $CDCl_3$

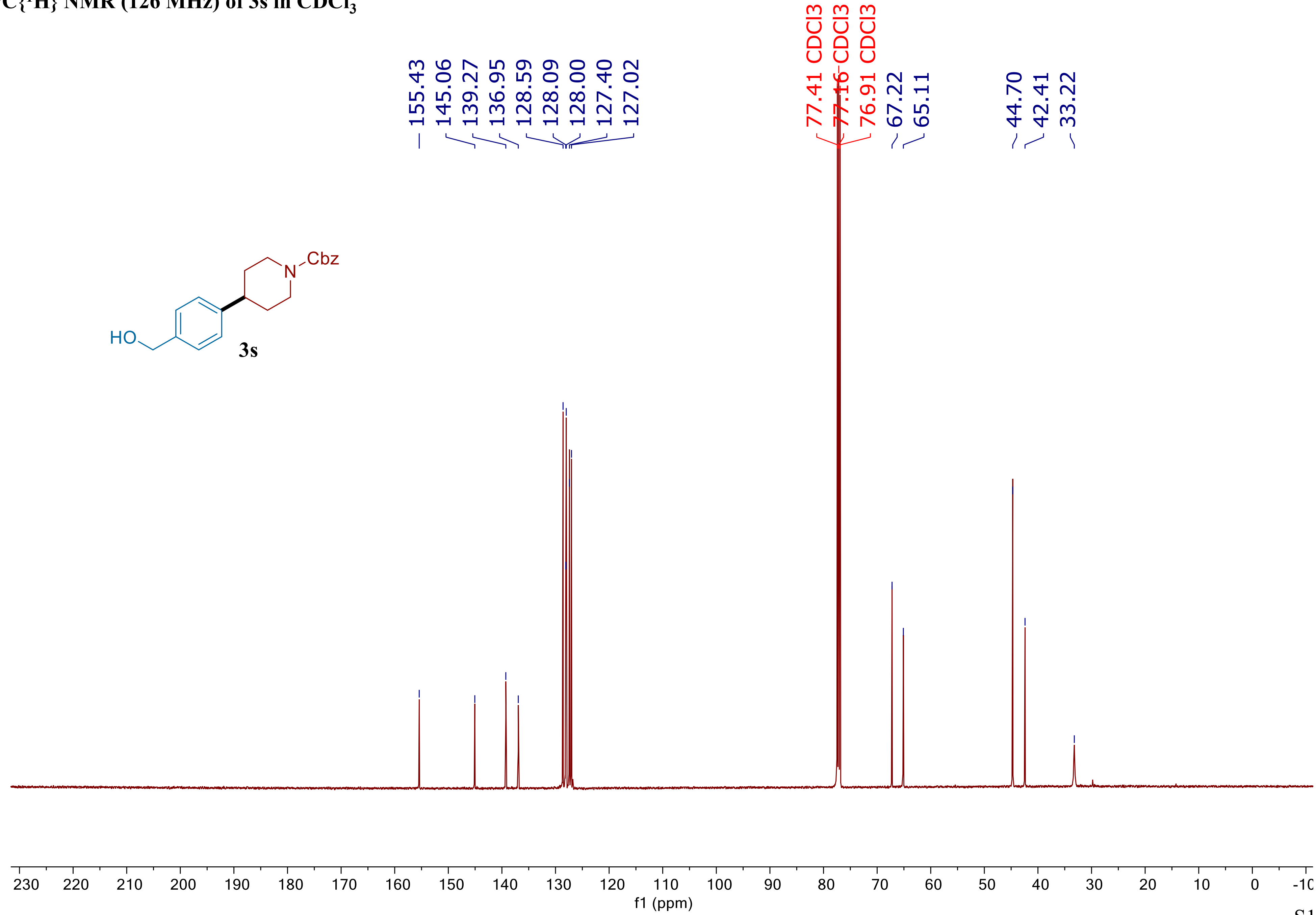

3s

^{1}H NMR (500 MHz) of 3t in $CDCl_3$

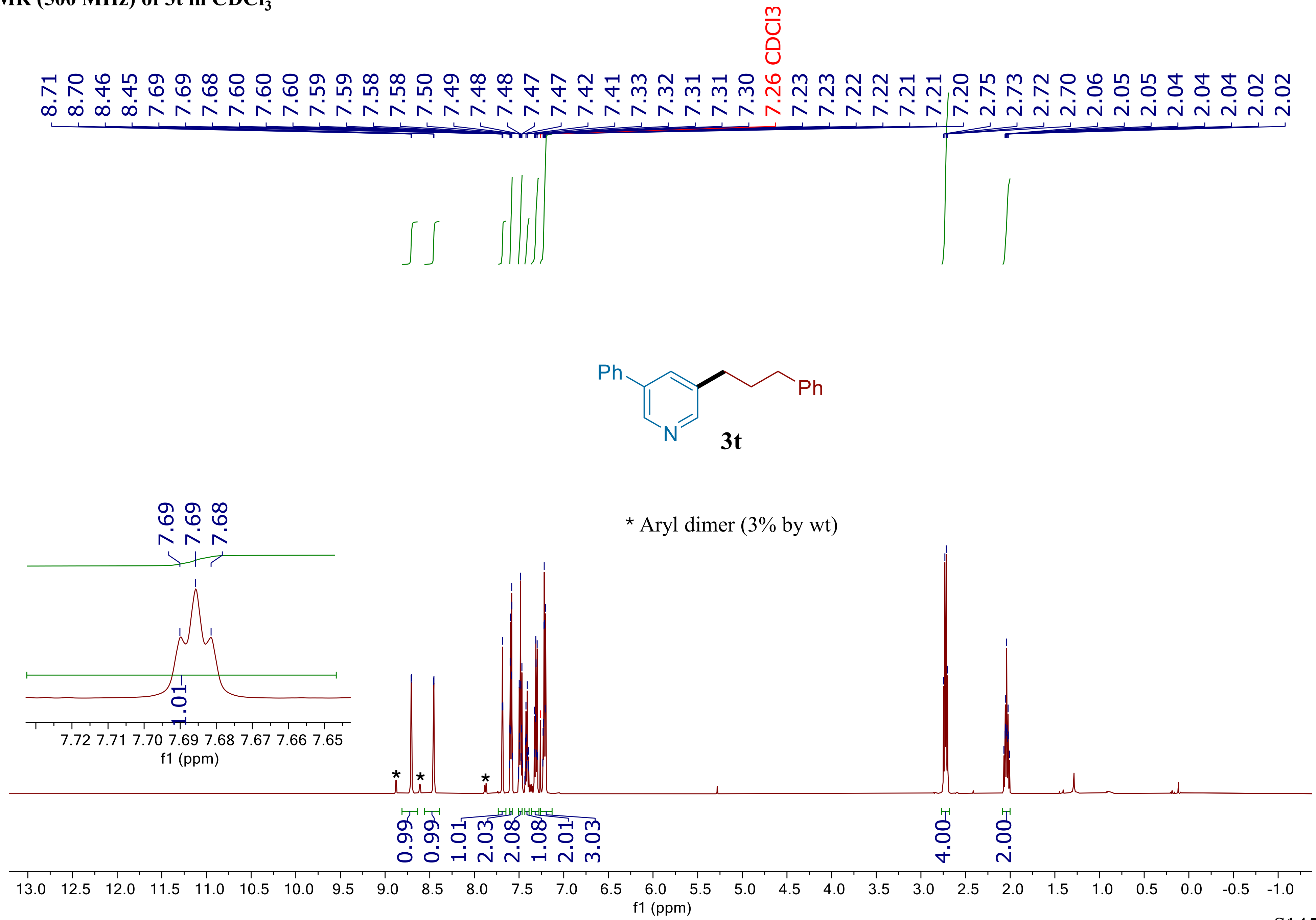

$^{13}C\{^{1}H\}$ NMR (126 MHz) of 3t in $CDCl_3$

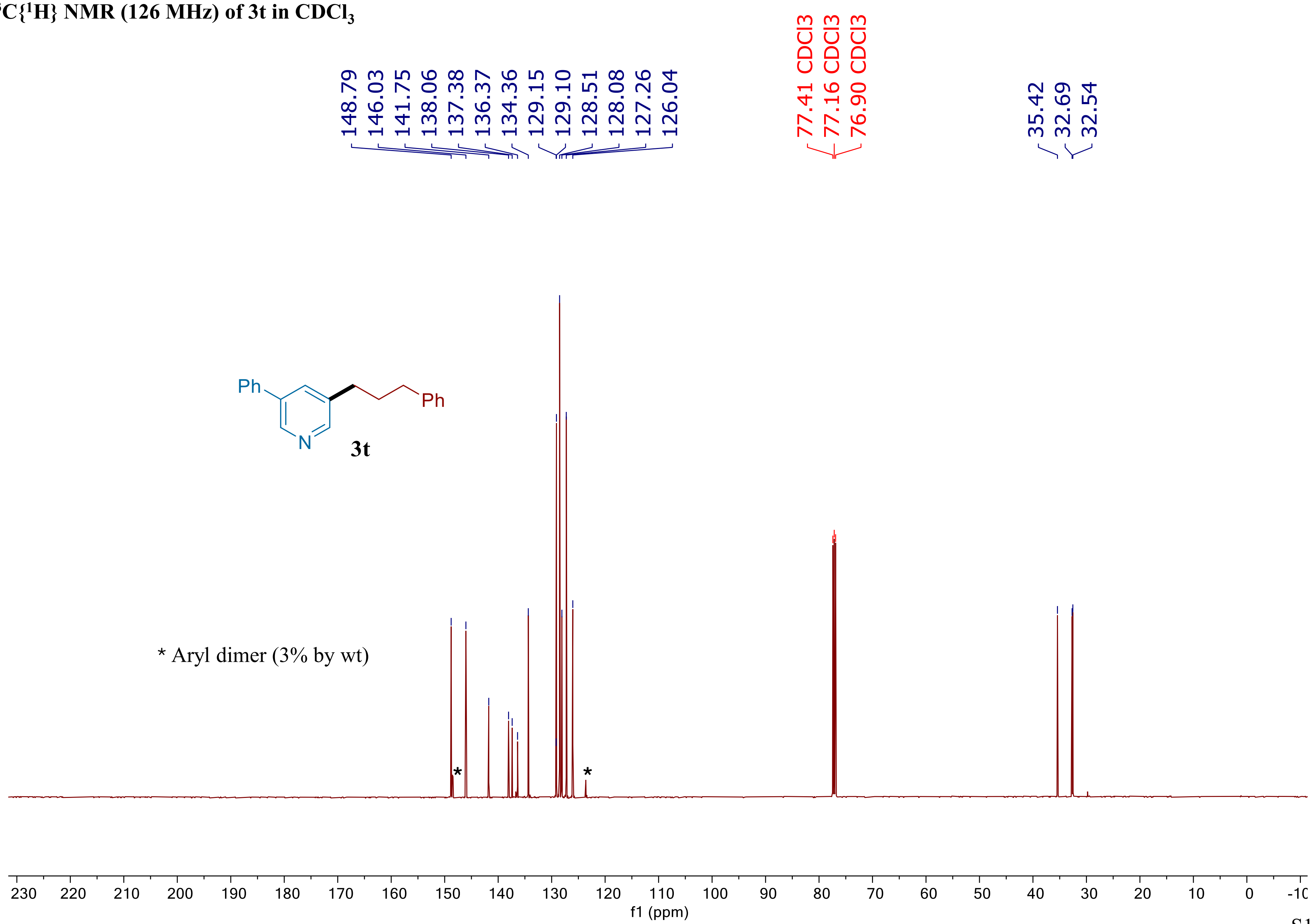

^{1}H NMR (500 MHz) of 3u in $CDCl_3$

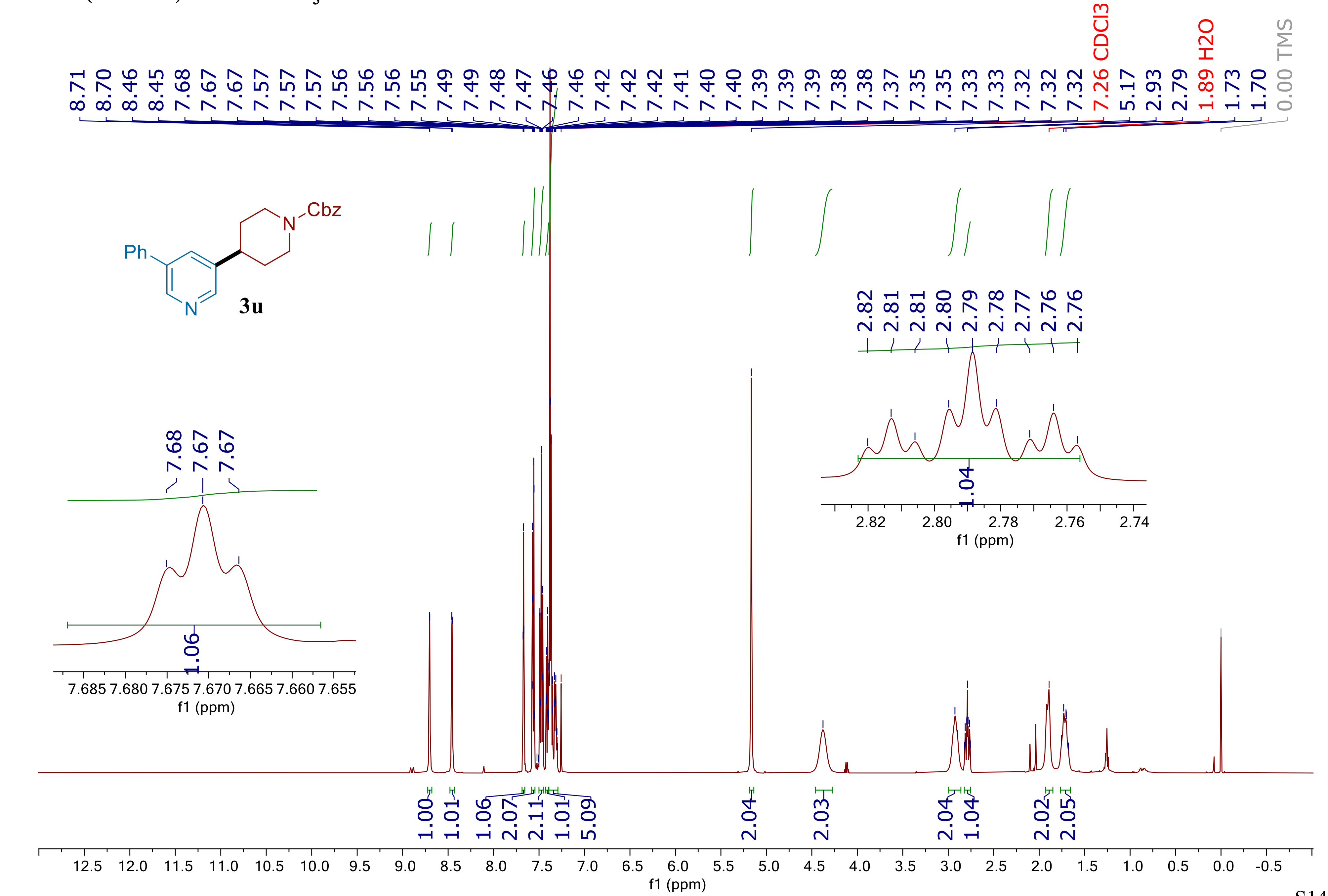

$^{13}C\{^{1}H\}$ NMR (126 MHz) of 3u in $CDCl_3$

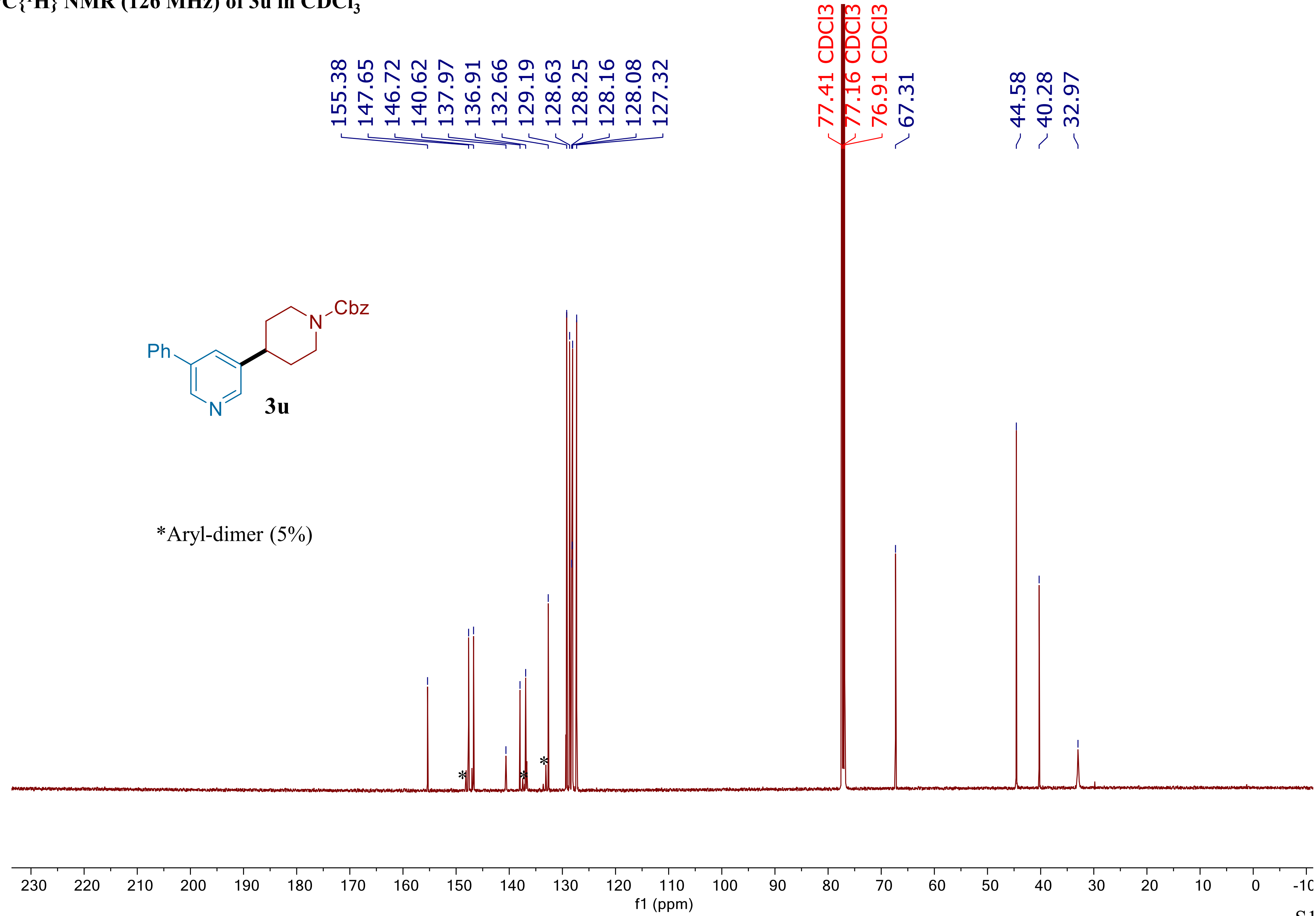

^{1}H NMR (500 MHz) of 3v in $CDCl_3$

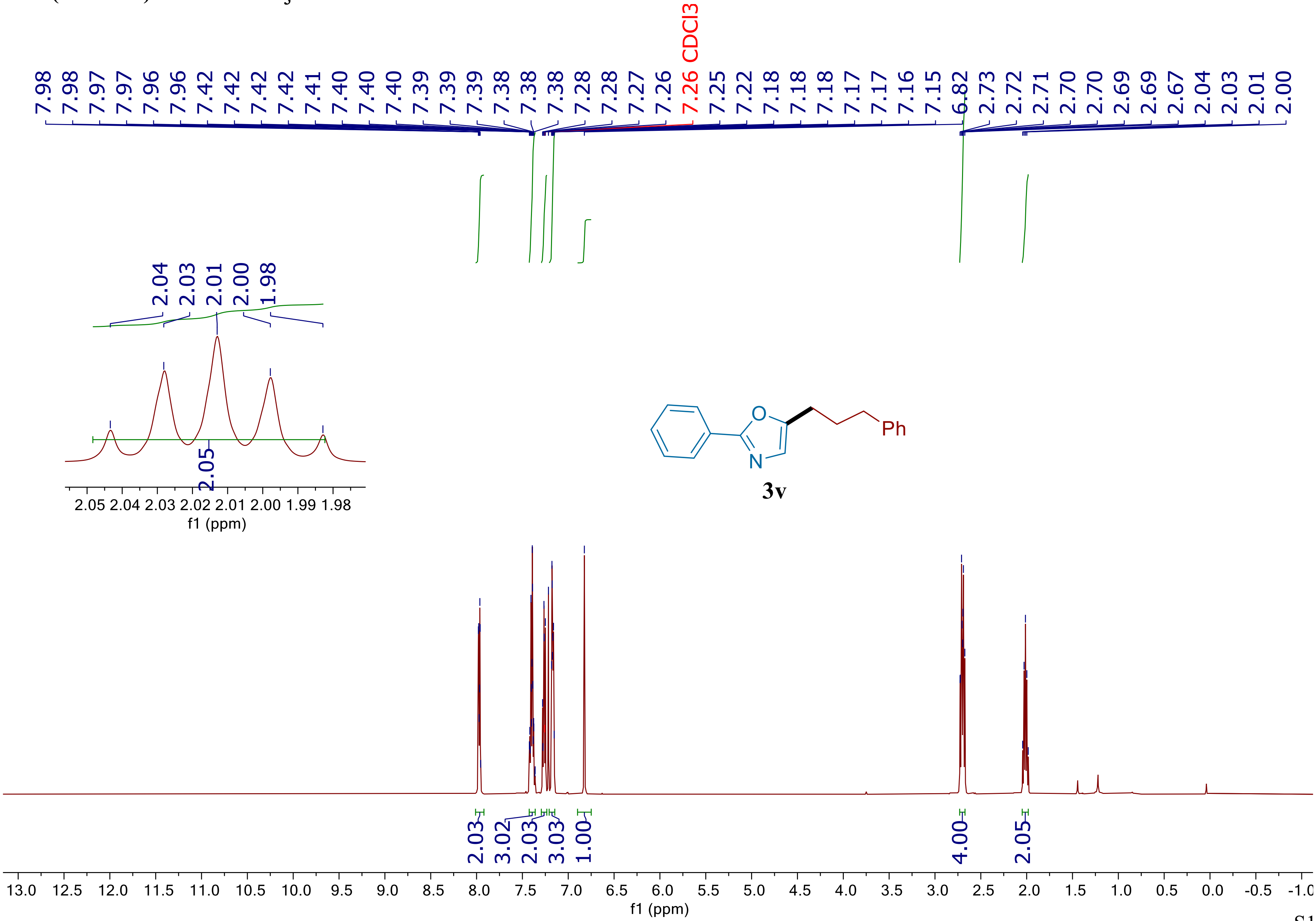

3v

$^{13}C\{^{1}H\}$ NMR (126 MHz) of 3v in $CDCl_3$

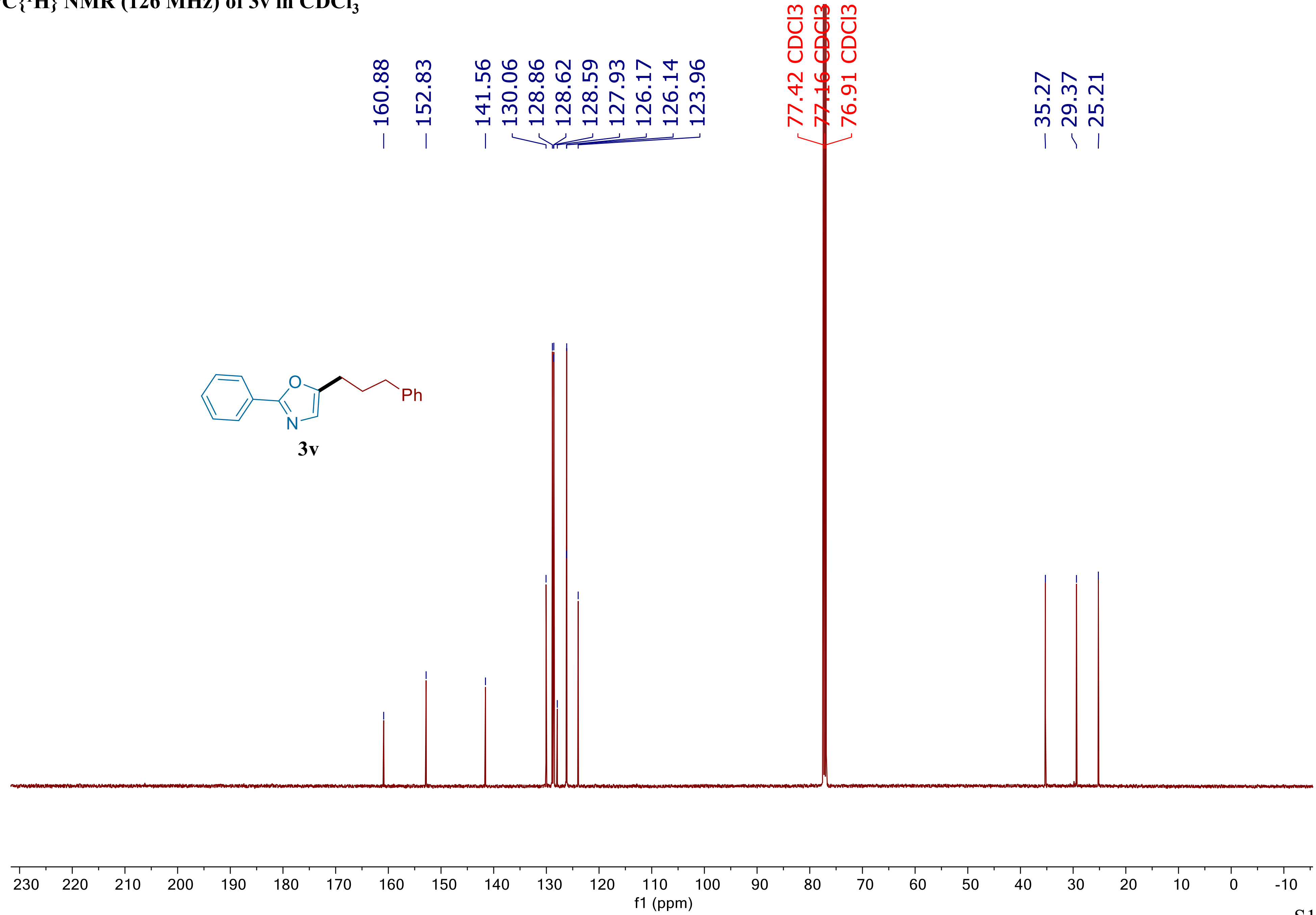

^{1}H NMR (500 MHz) of 3w in $CDCl_3$

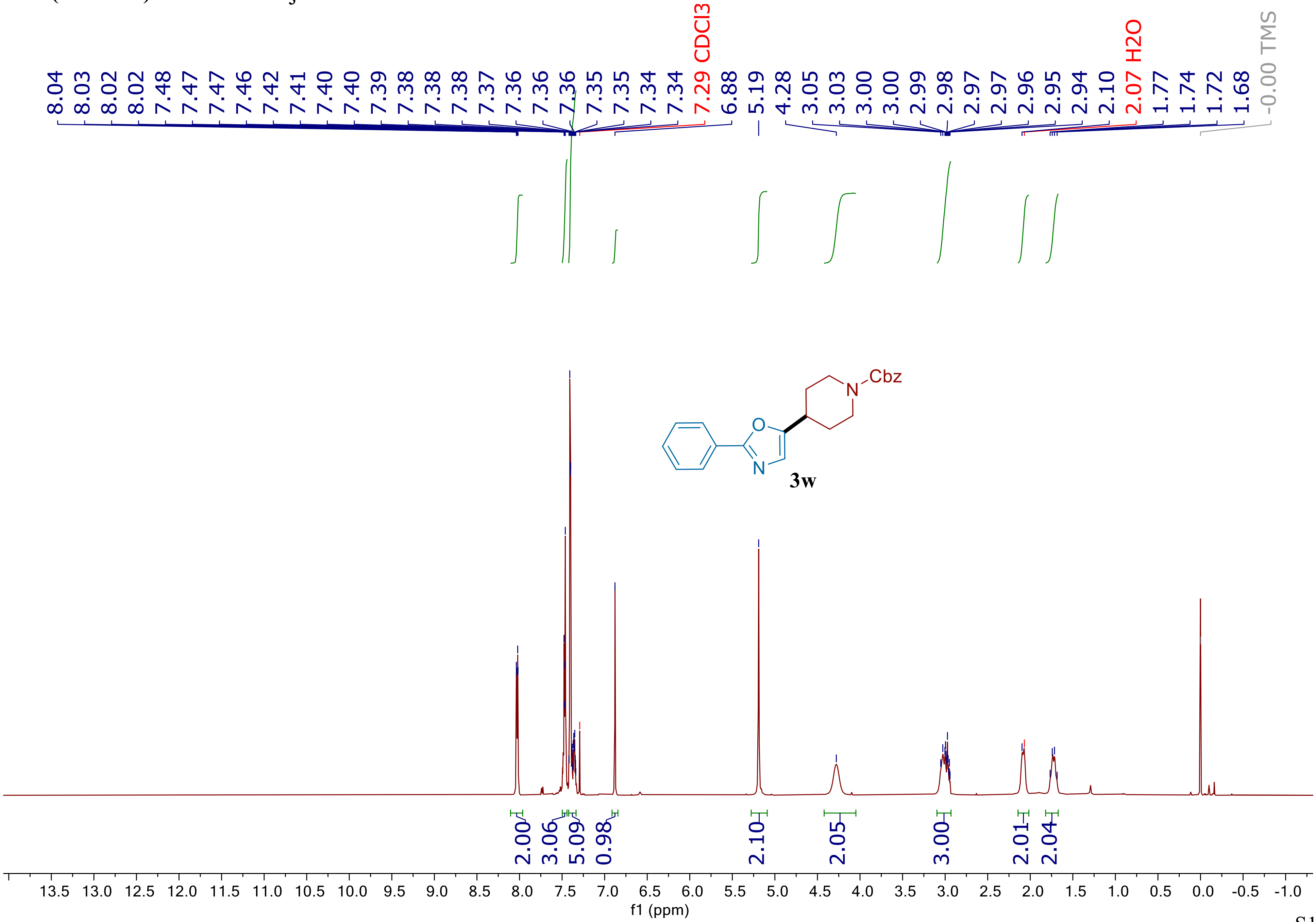

$^{13}C\{^1H\}$ NMR of 3w (125 MHz) in $CDCl_3$

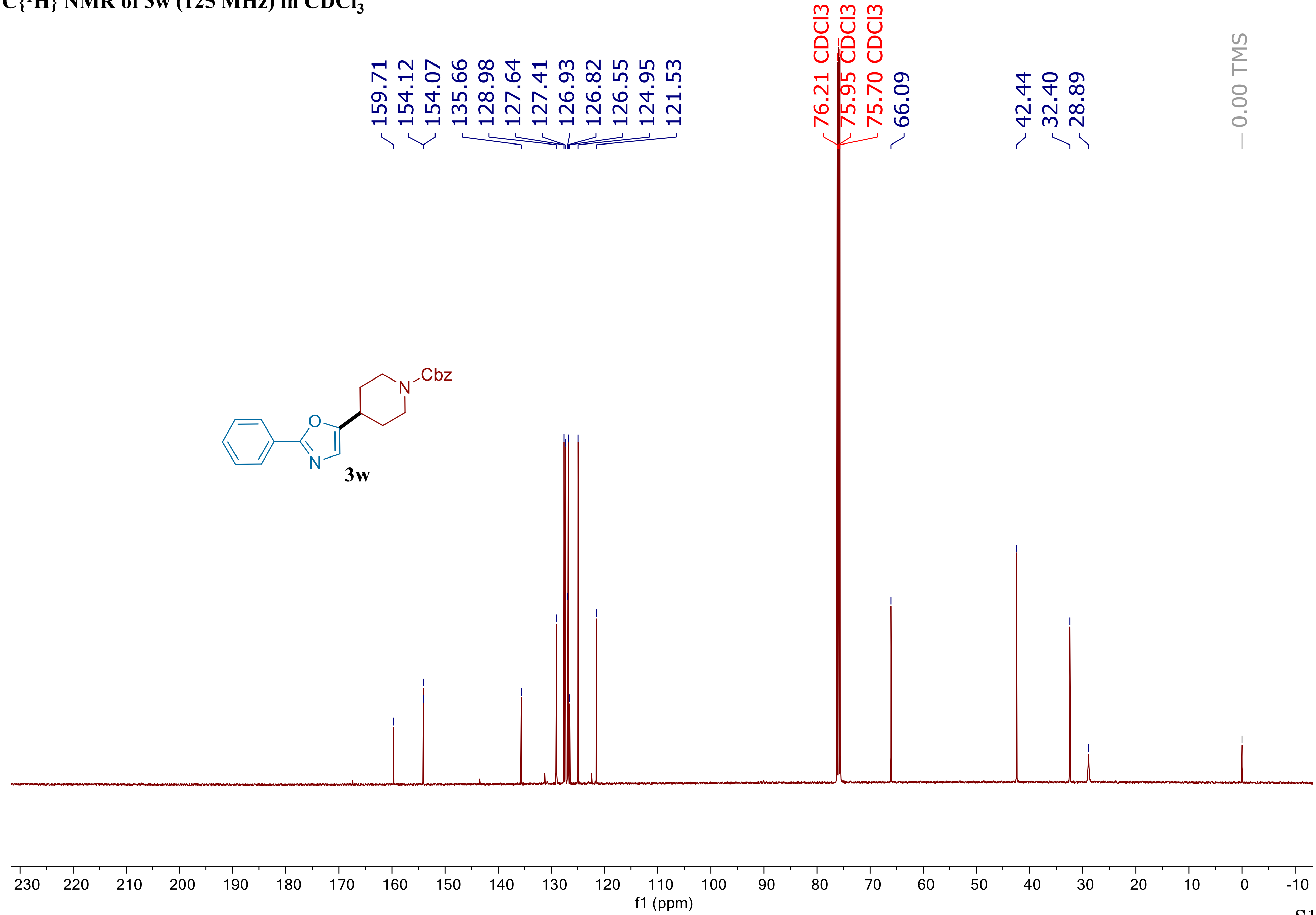

^{1}H NMR (500 MHz) of 3x in $CDCl_3$

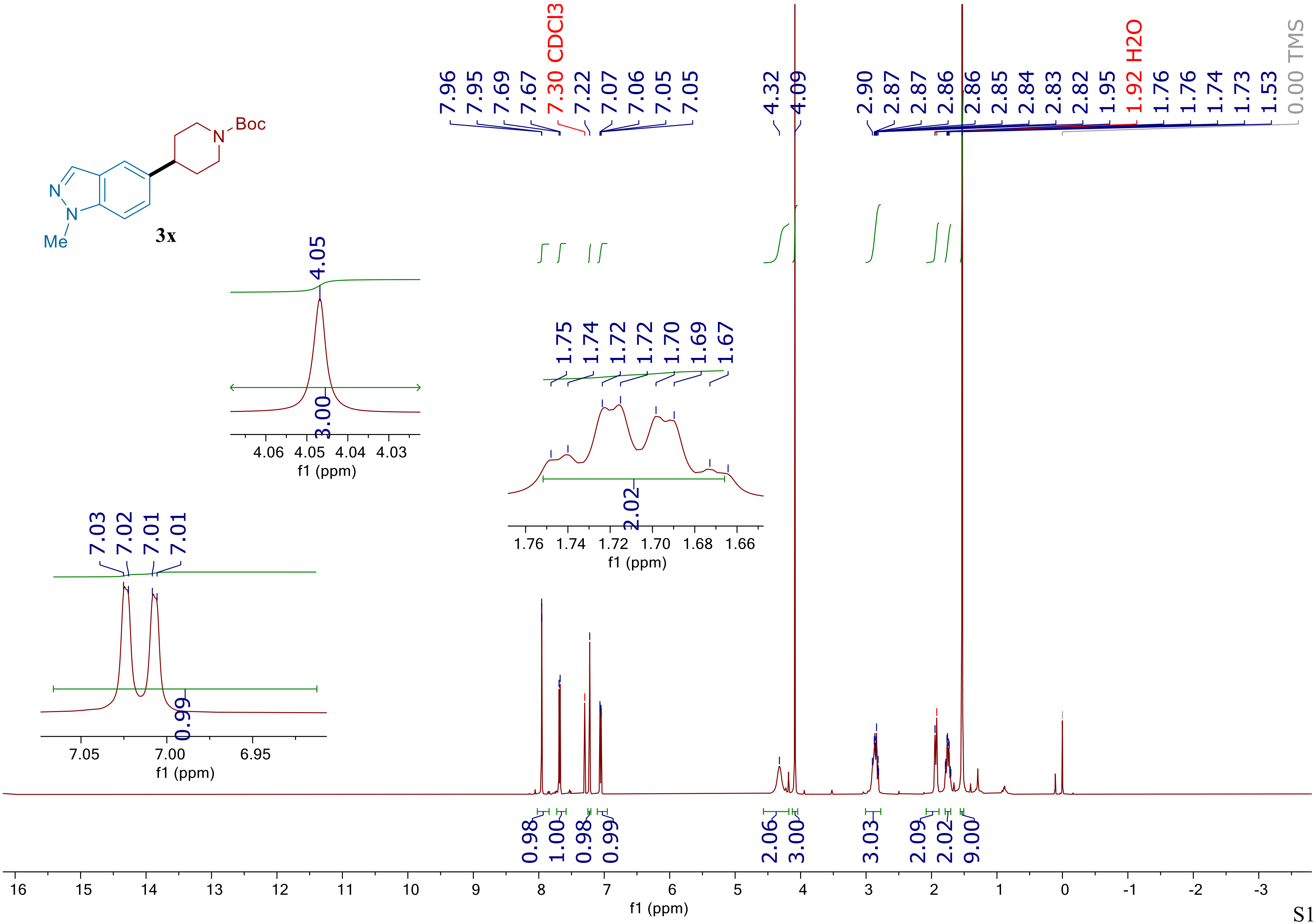

3x

$^{13}C\{^1H\}$ NMR (126 MHz) of 3x in $CDCl_3$

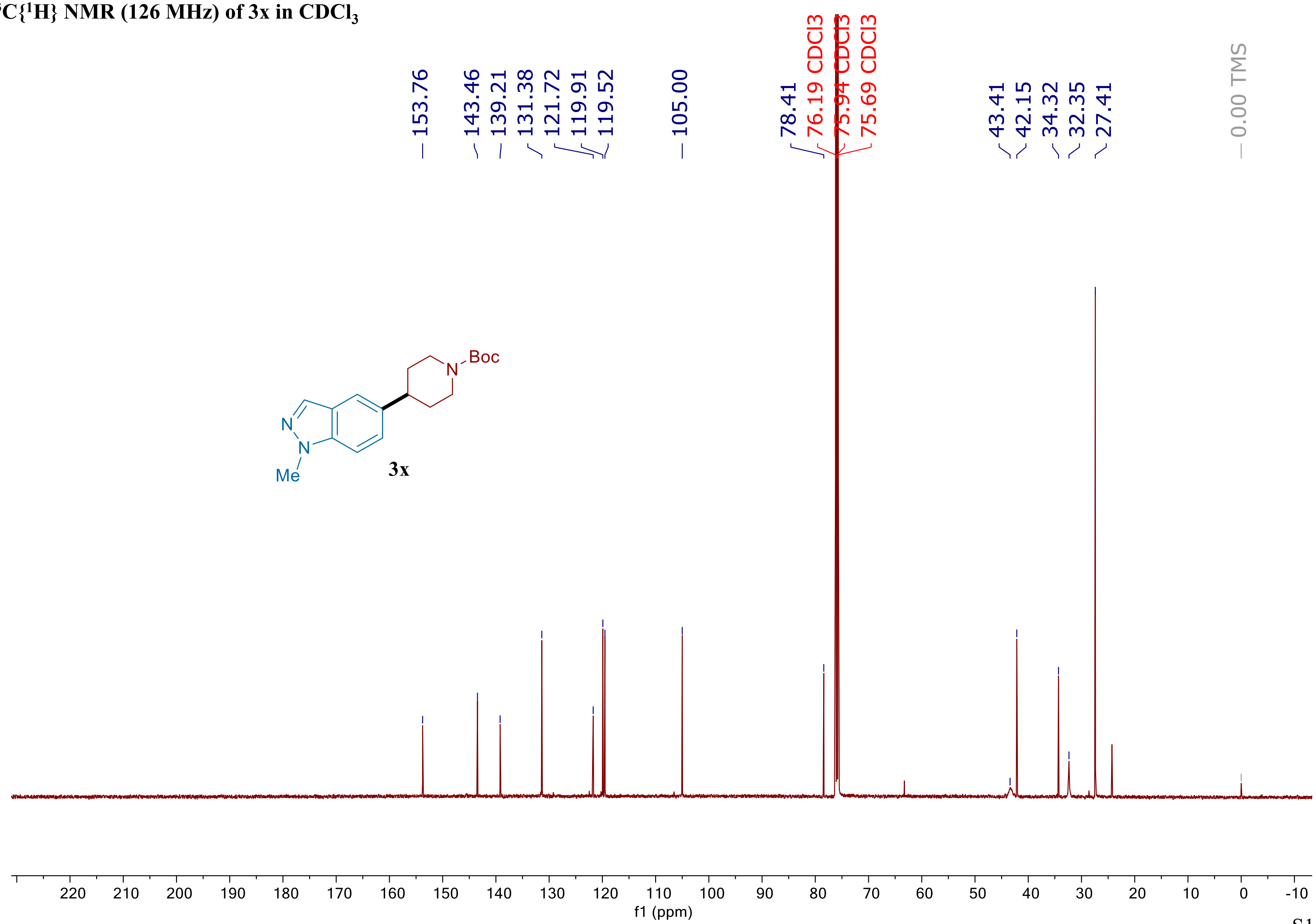

^{1}H NMR (500 MHz) of 3y in $CDCl_3$

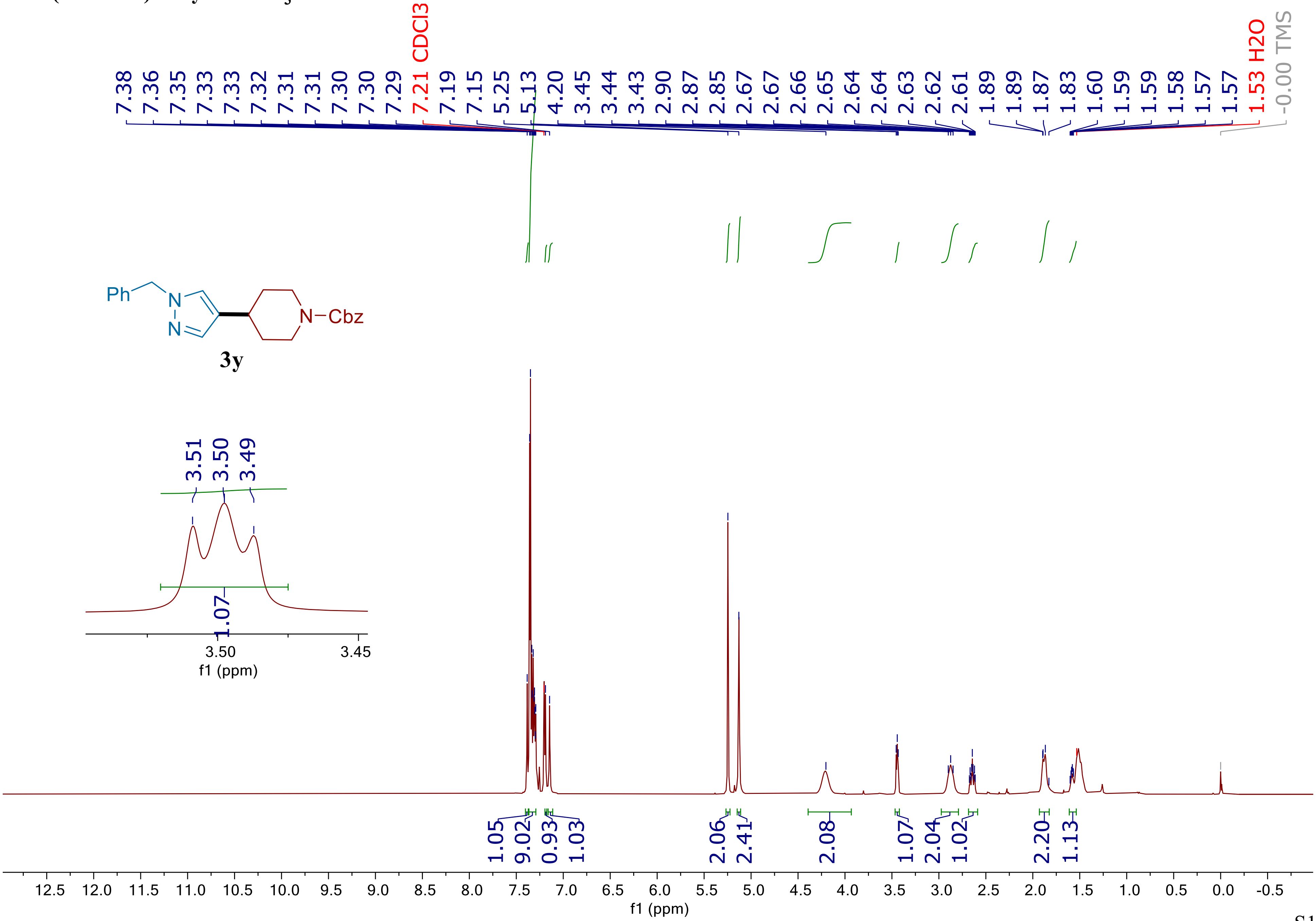

$^{13}C\{^1H\}$ NMR (126 MHz) of 3y in $CDCl_3$

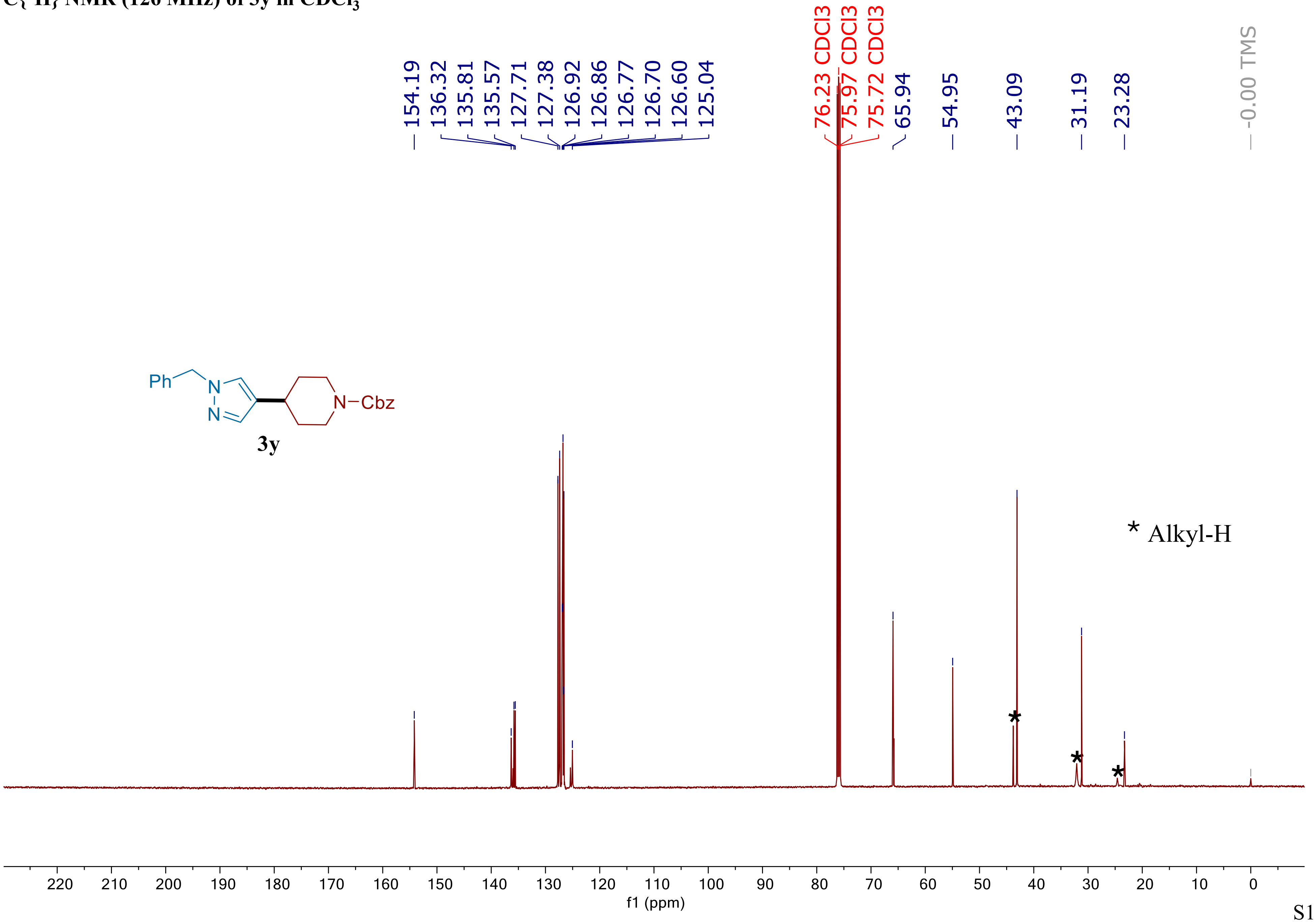

^{1}H NMR (500 MHz) of 3z in $CDCl_3$

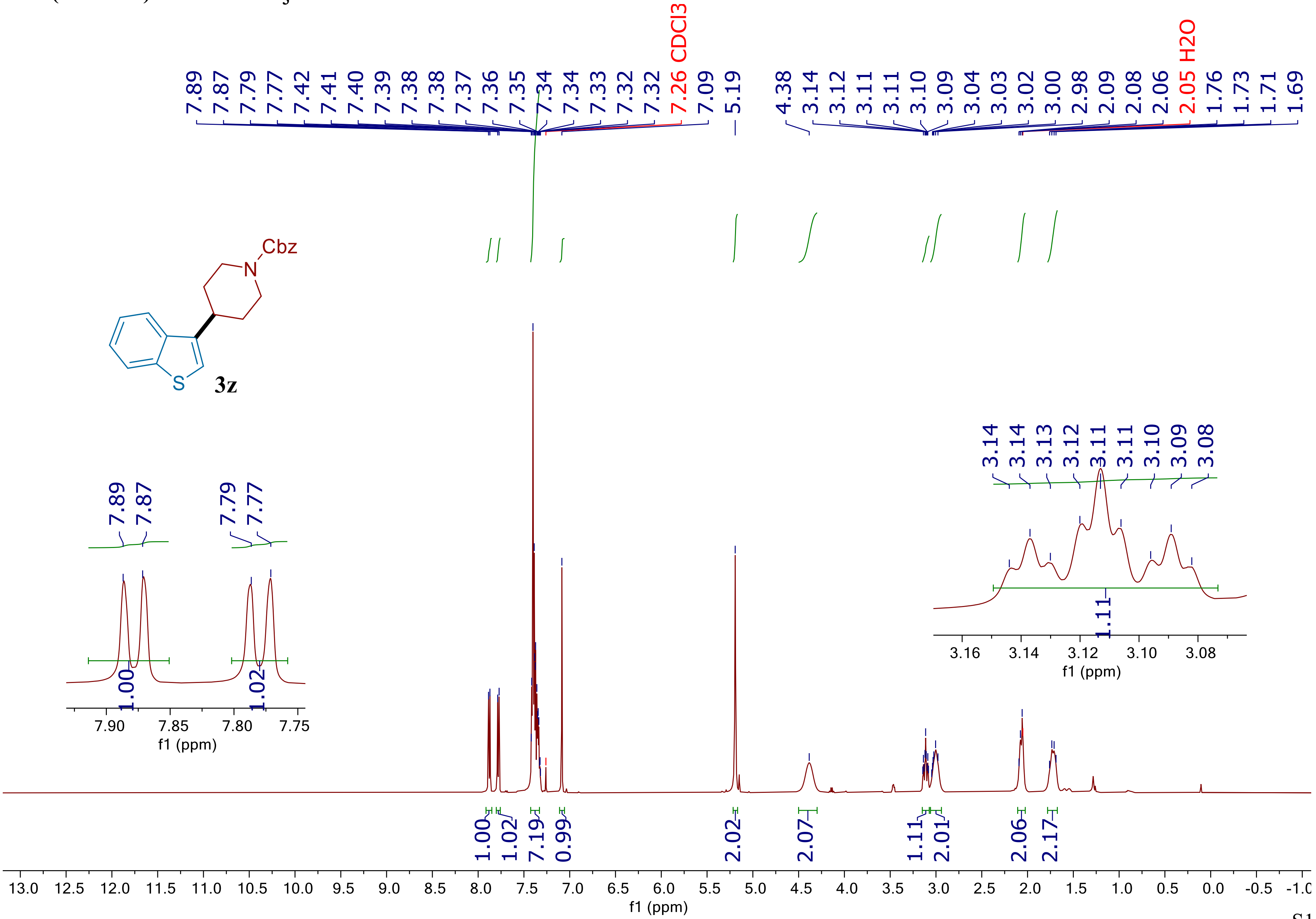

$^{13}C\{^{1}H\}$ NMR (126 MHz) of 3z in $CDCl_3$

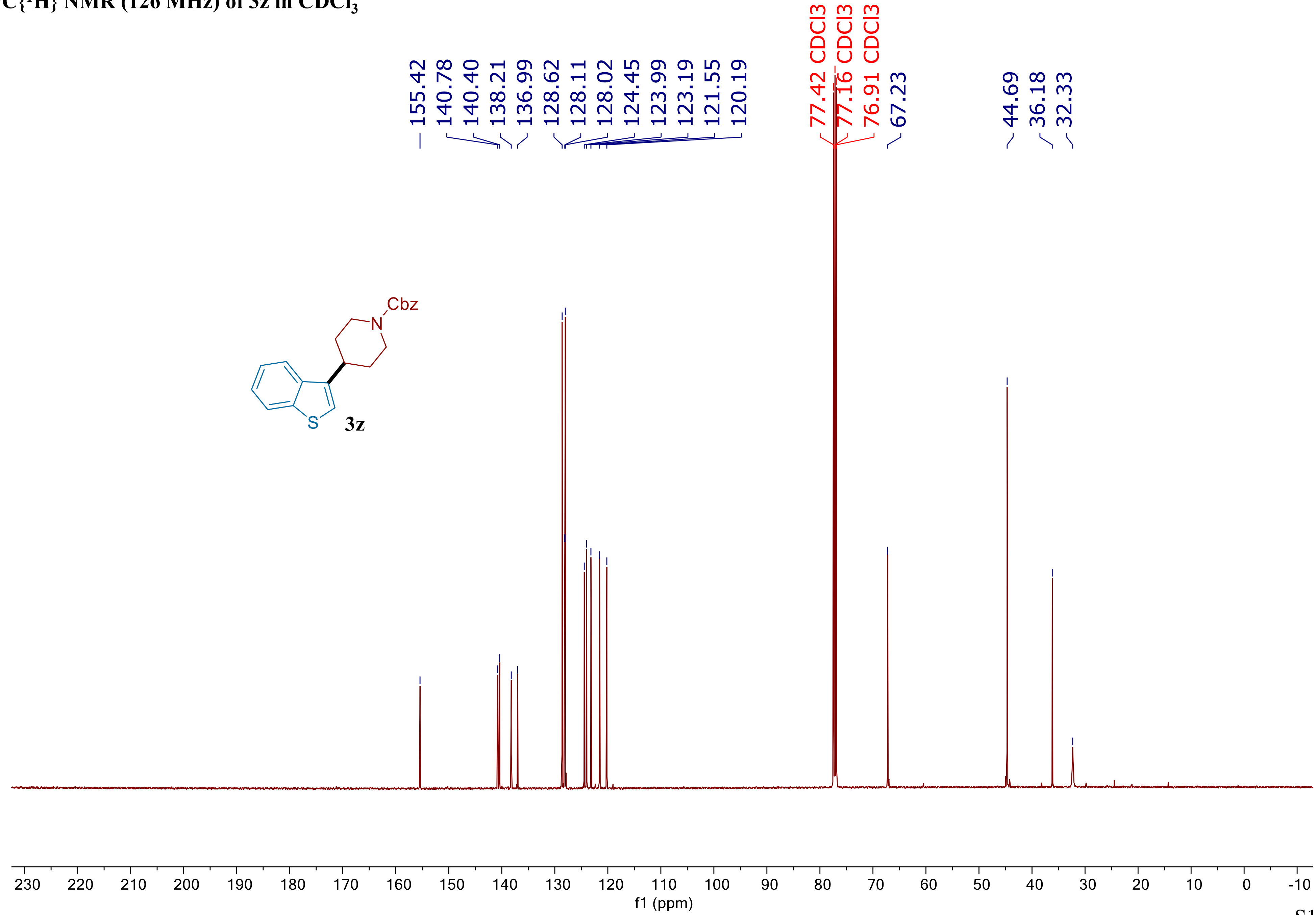

^{1}H NMR (500 MHz) of 3aa in $CDCl_3$

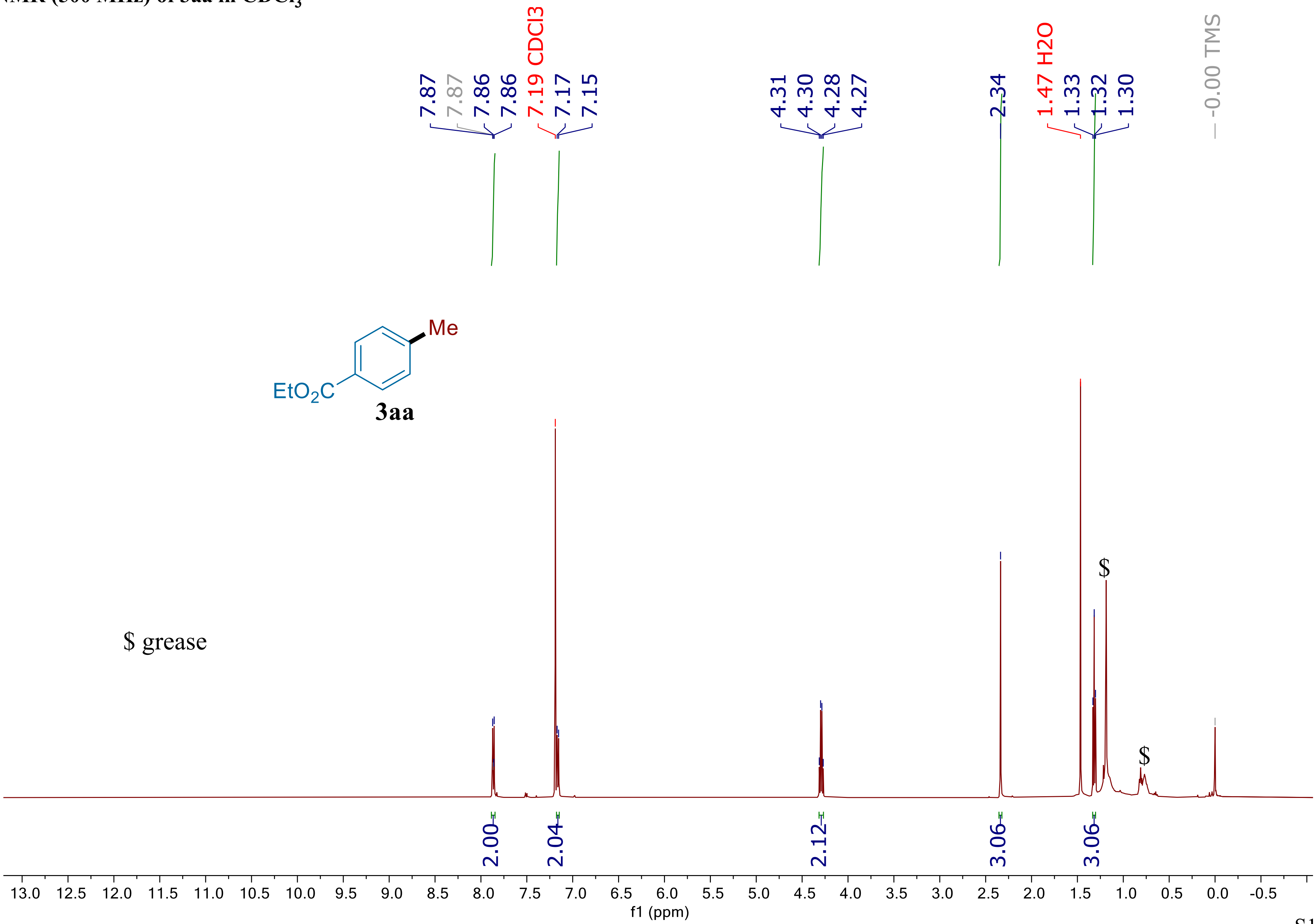

$^{13}C\{^{1}H\}$ NMR (126 MHz) of 3aa in $CDCl_3$

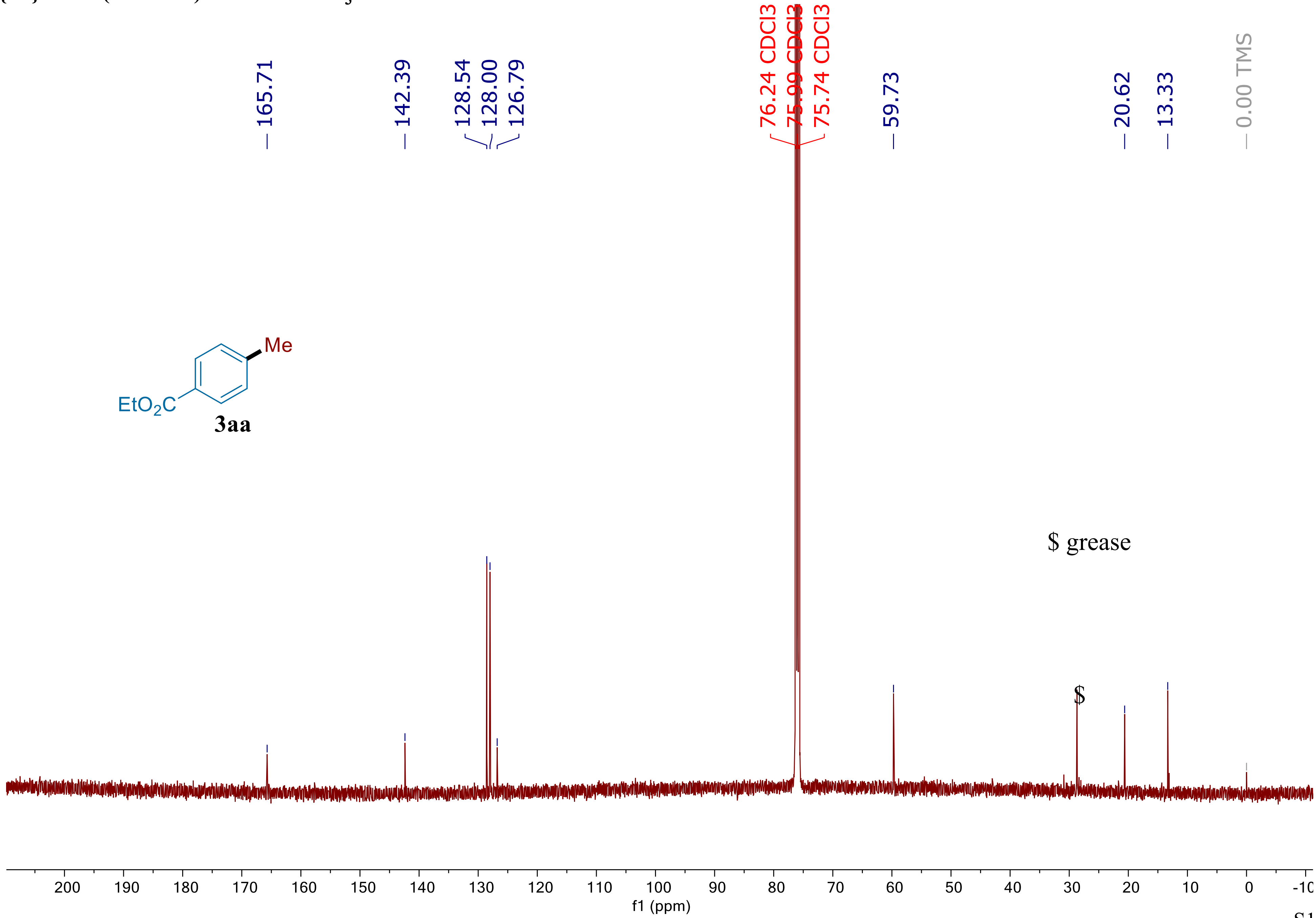

^{1}H NMR (500 MHz) of 3ab in $CDCl_3$

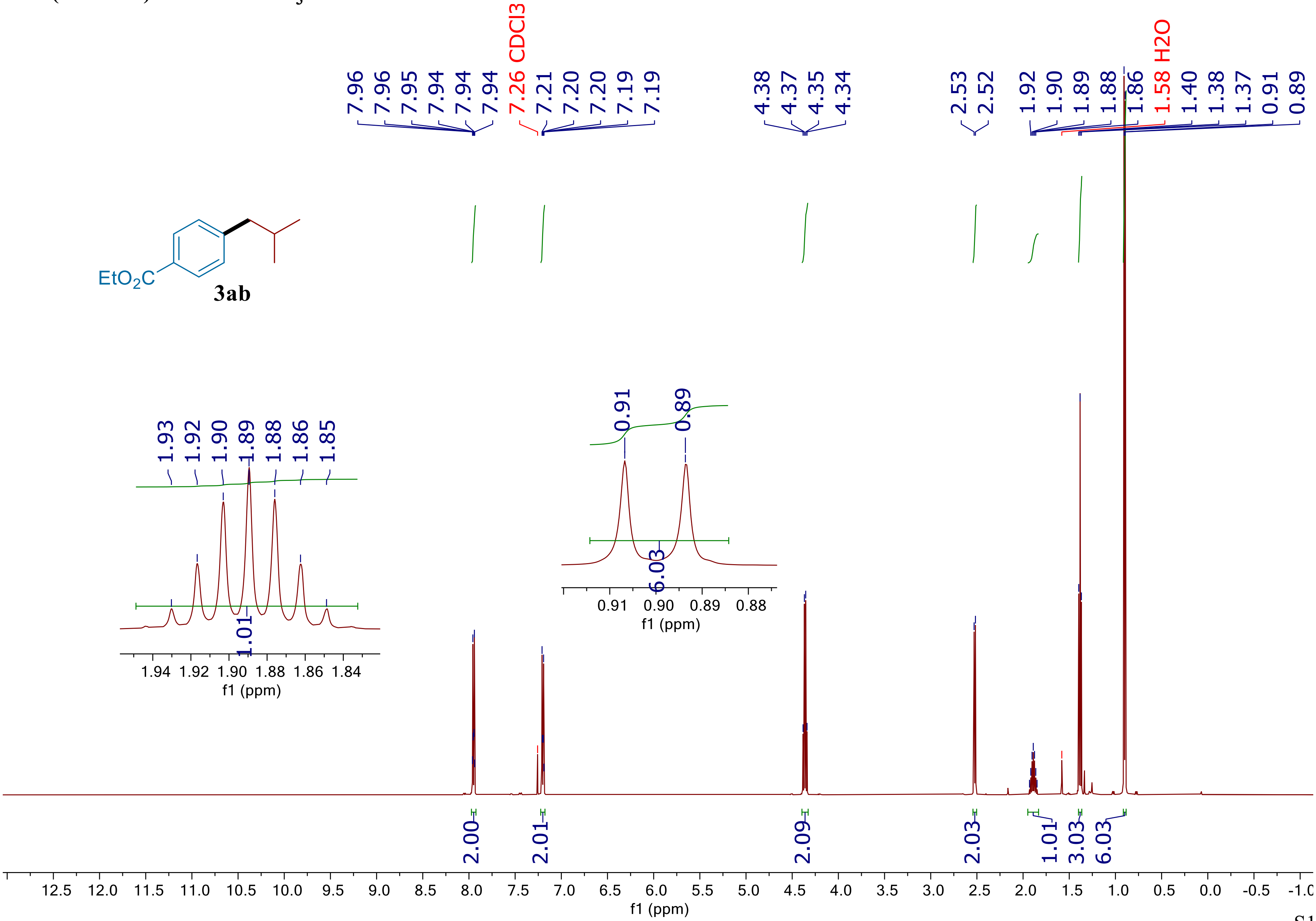

$^{13}C\{^{1}H\}$ NMR (126 MHz) of 3ab in $CDCl_3$

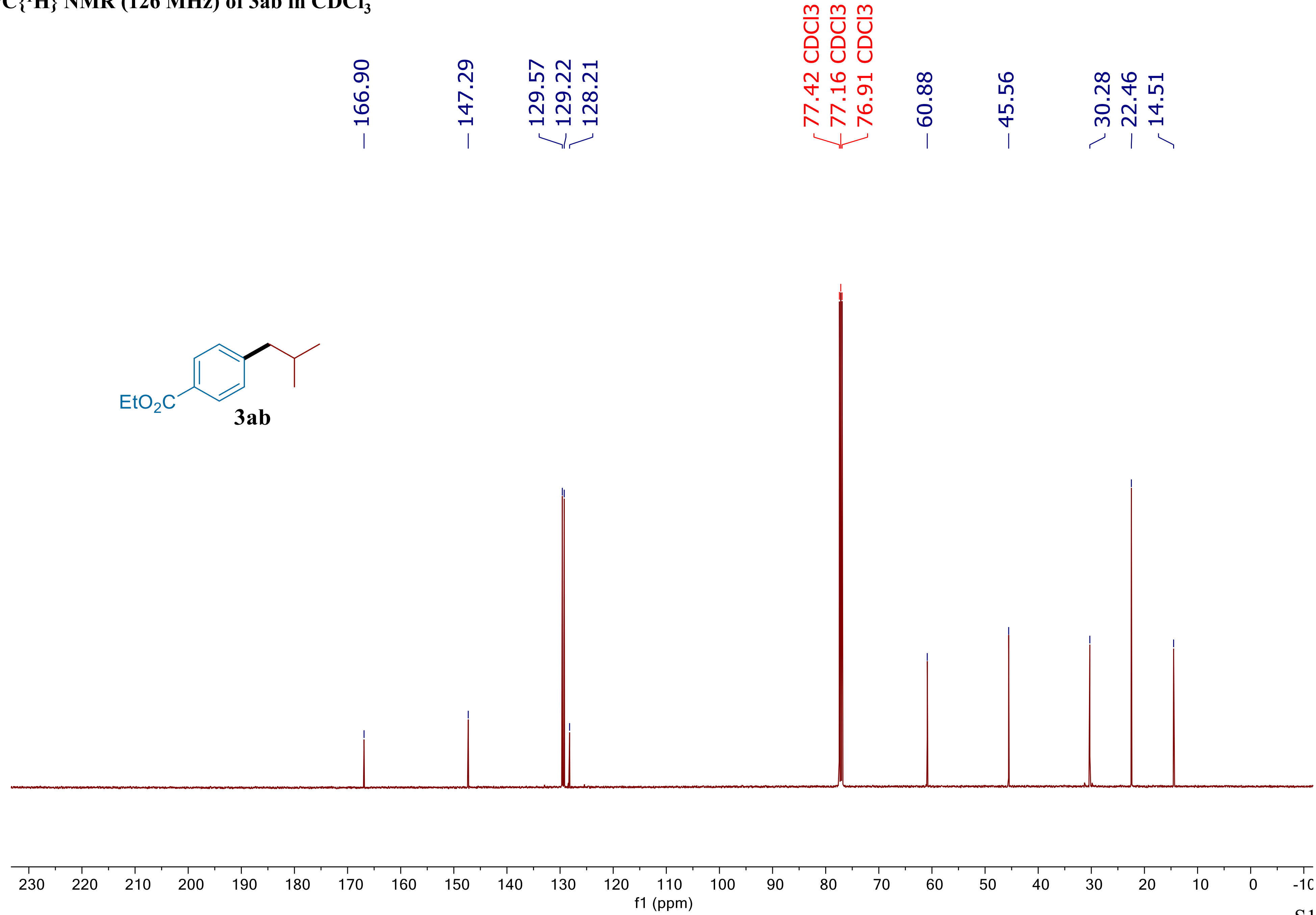

3ab

^1H NMR (500 MHz) of 3ac in $CDCl_3$

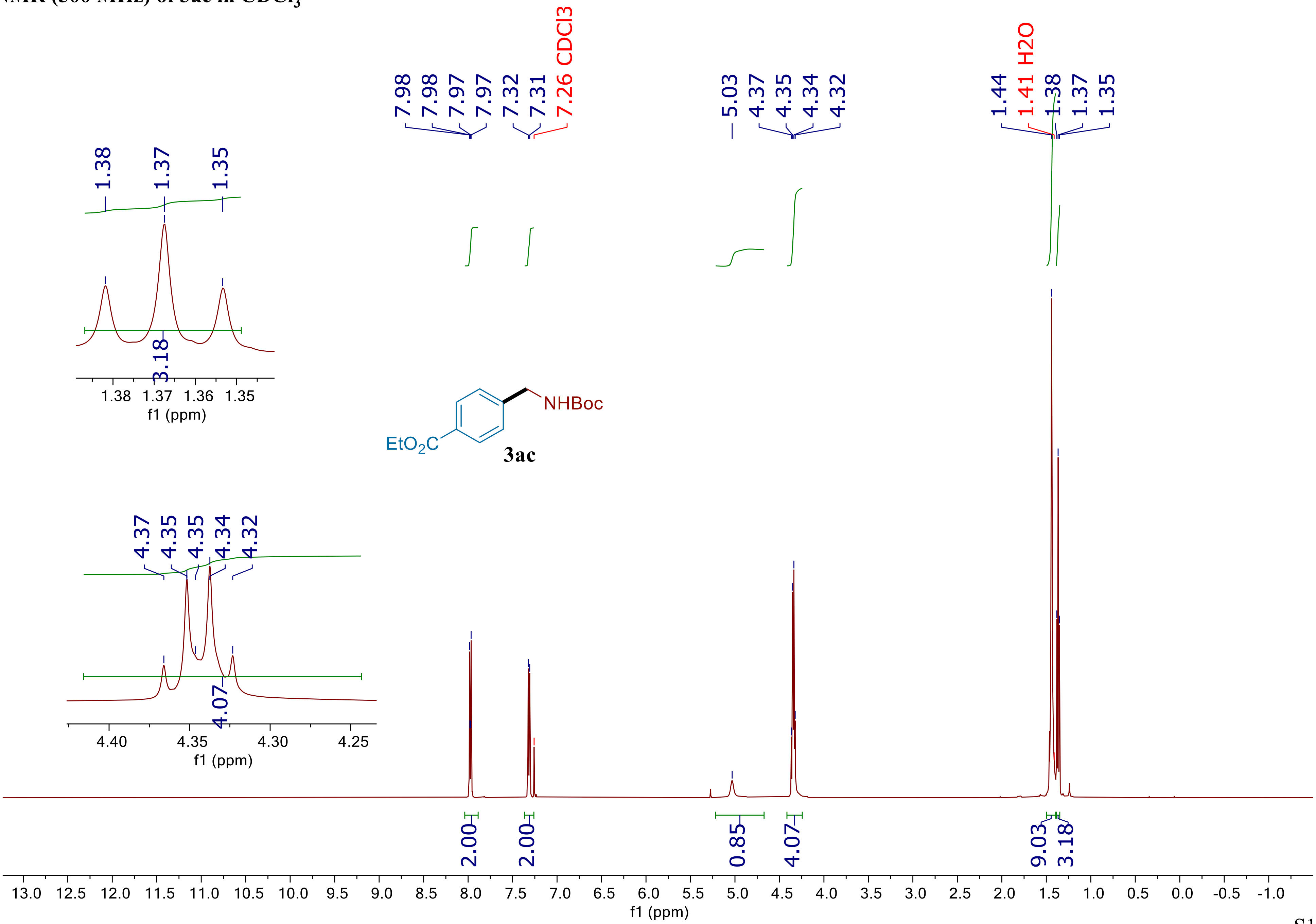

$^{13}C\{^{1}H\}$ NMR (126 MHz) of 3ac in $CDCl_3$

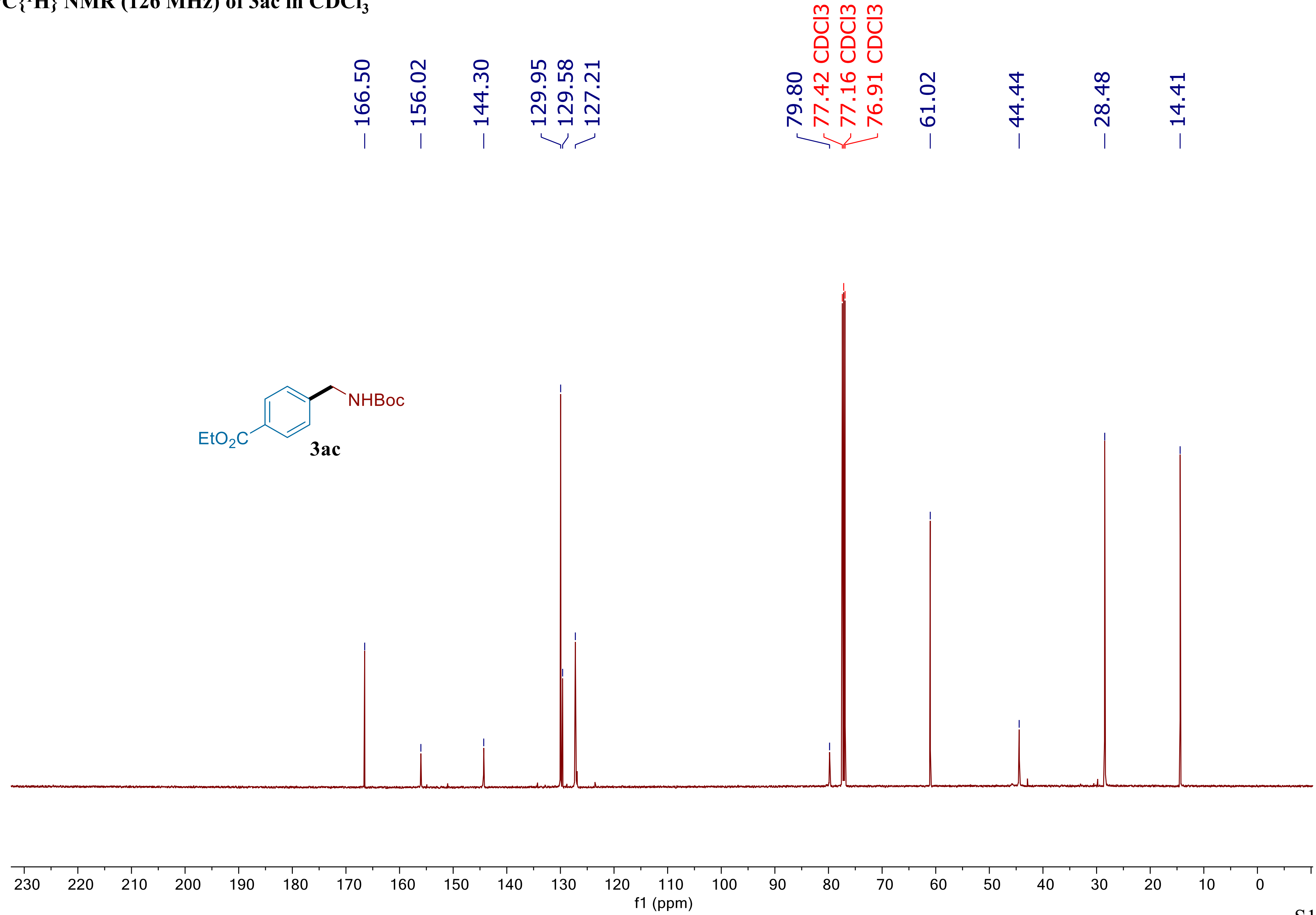

^{1}H NMR (500 MHz) of 3ad in $CDCl_3$

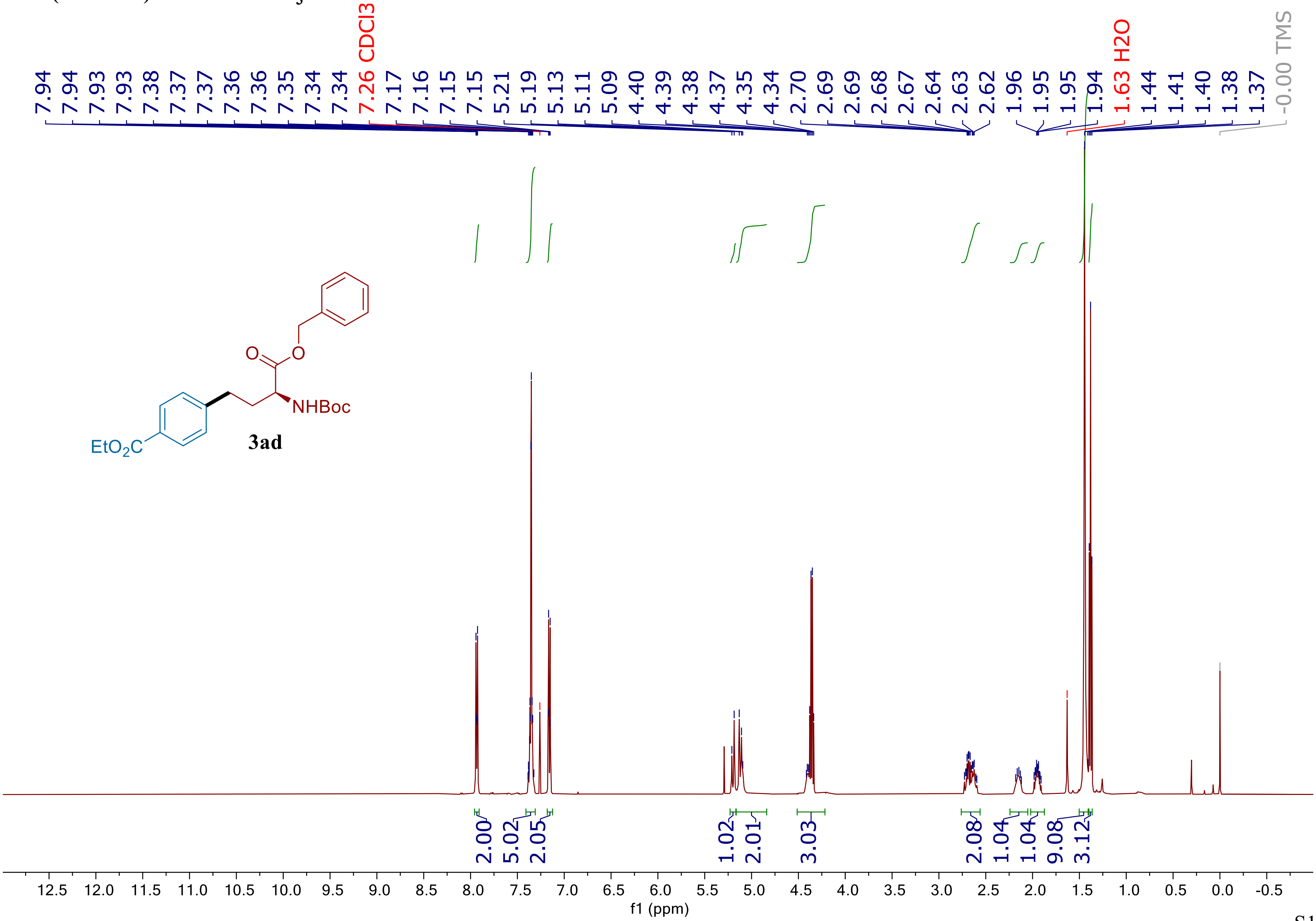

$^{13}C\{^{1}H\}$ NMR (126 MHz) of 3ad in $CDCl_3$

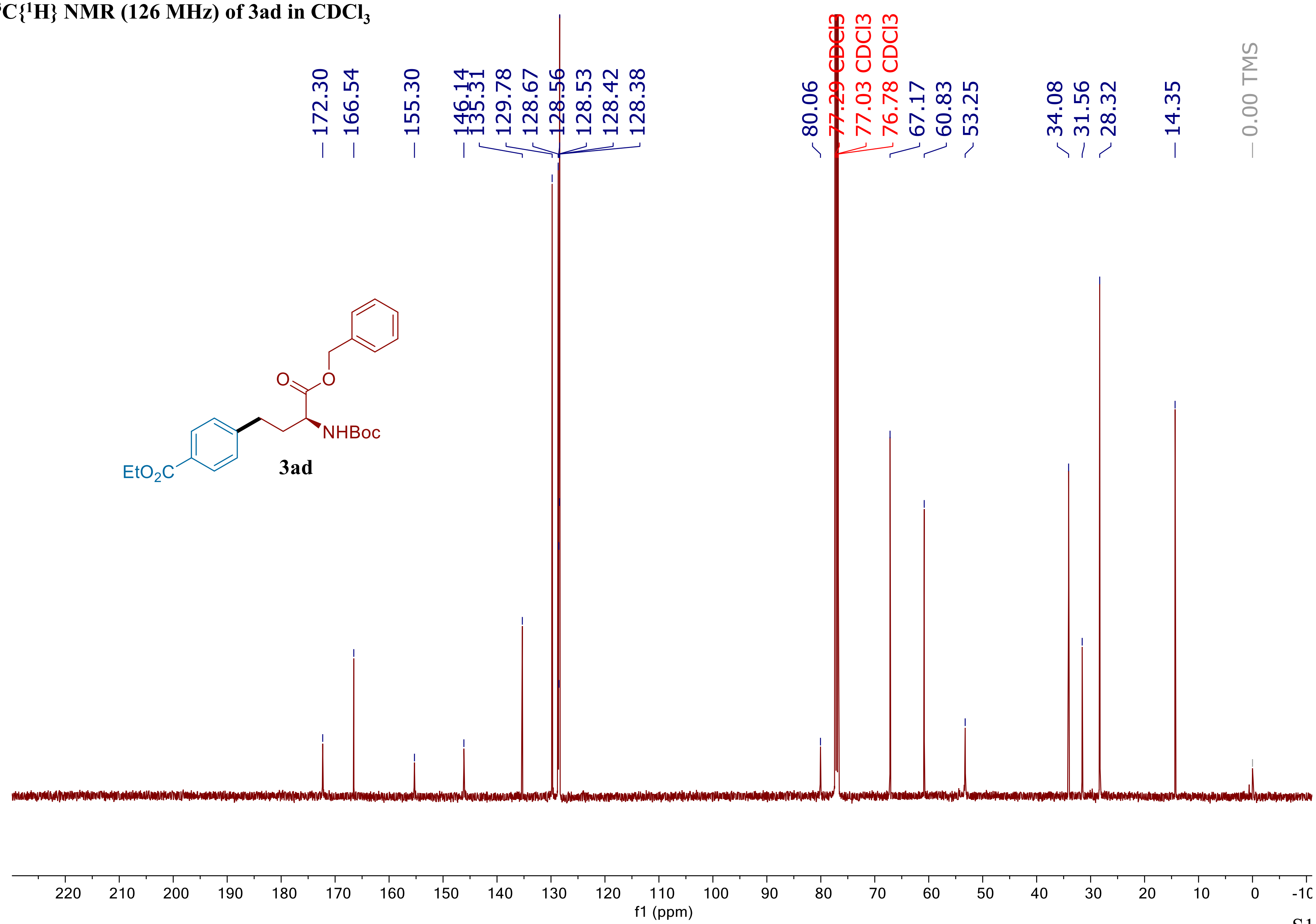

^{1}H NMR (500 MHz) of 3ae in $CDCl_3$

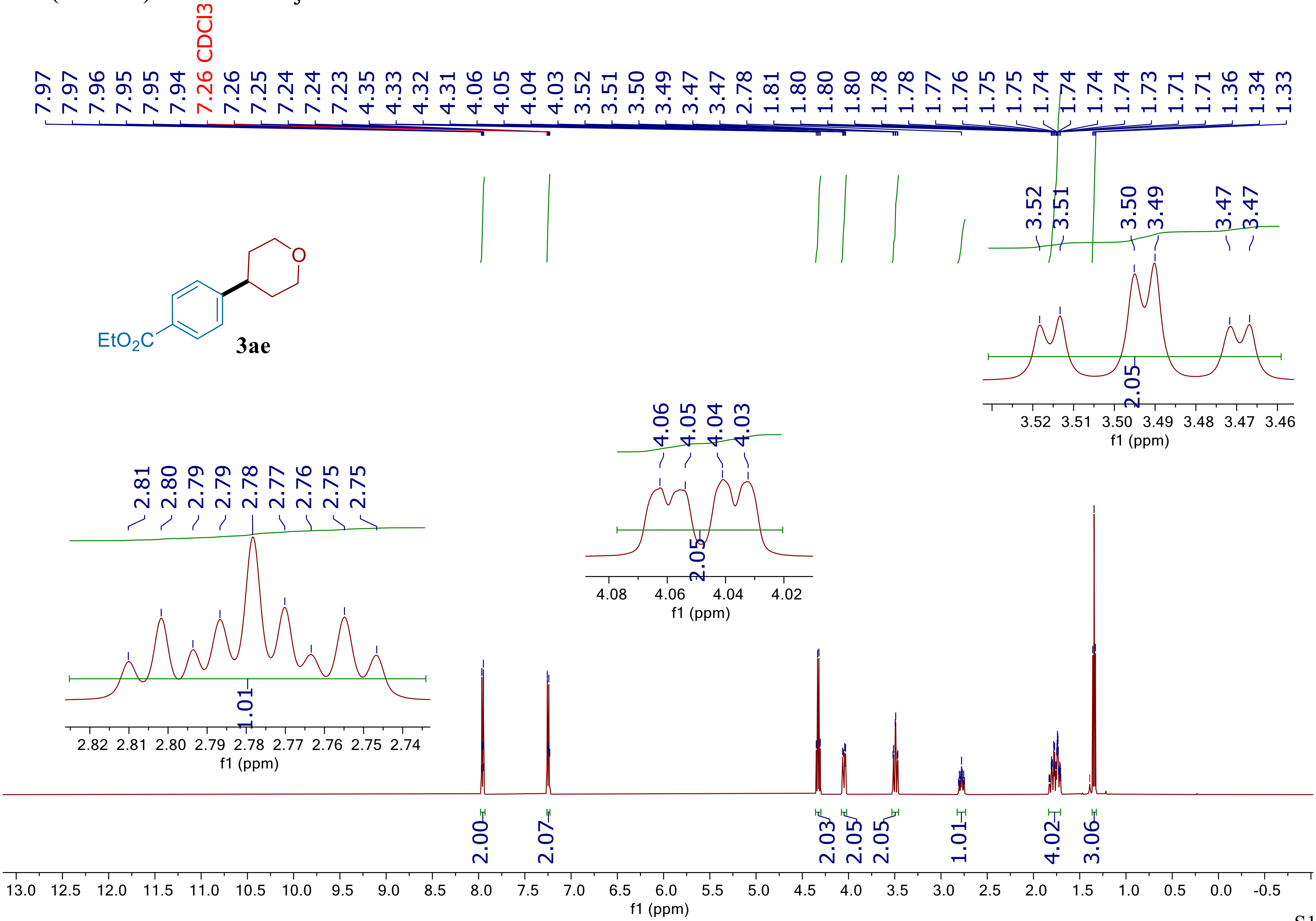

$^{13}C\{^{1}H\}$ NMR (126 MHz) of 3ae in $CDCl_3$

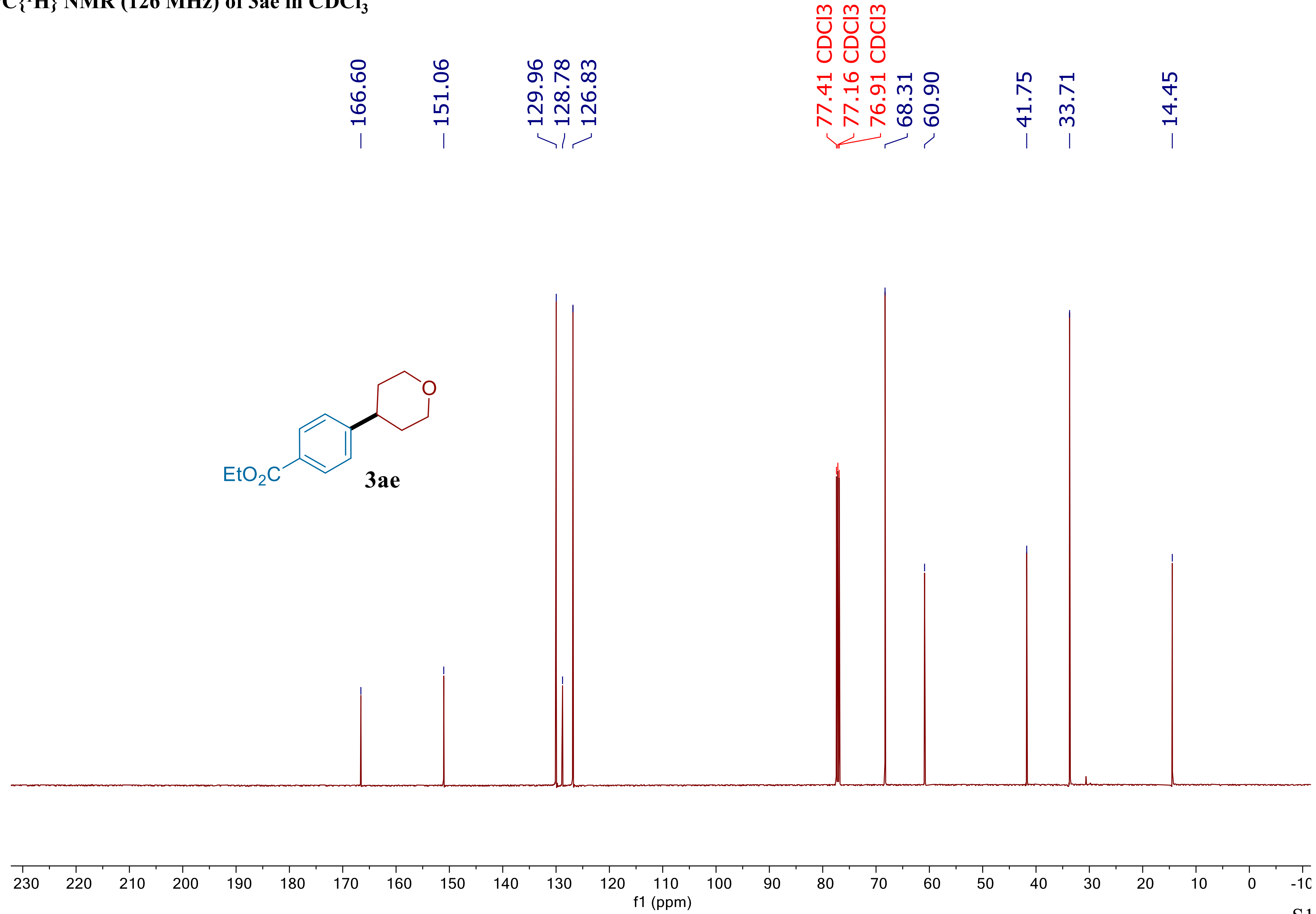

^1H NMR (500 MHz) of 3af in $CDCl_3$

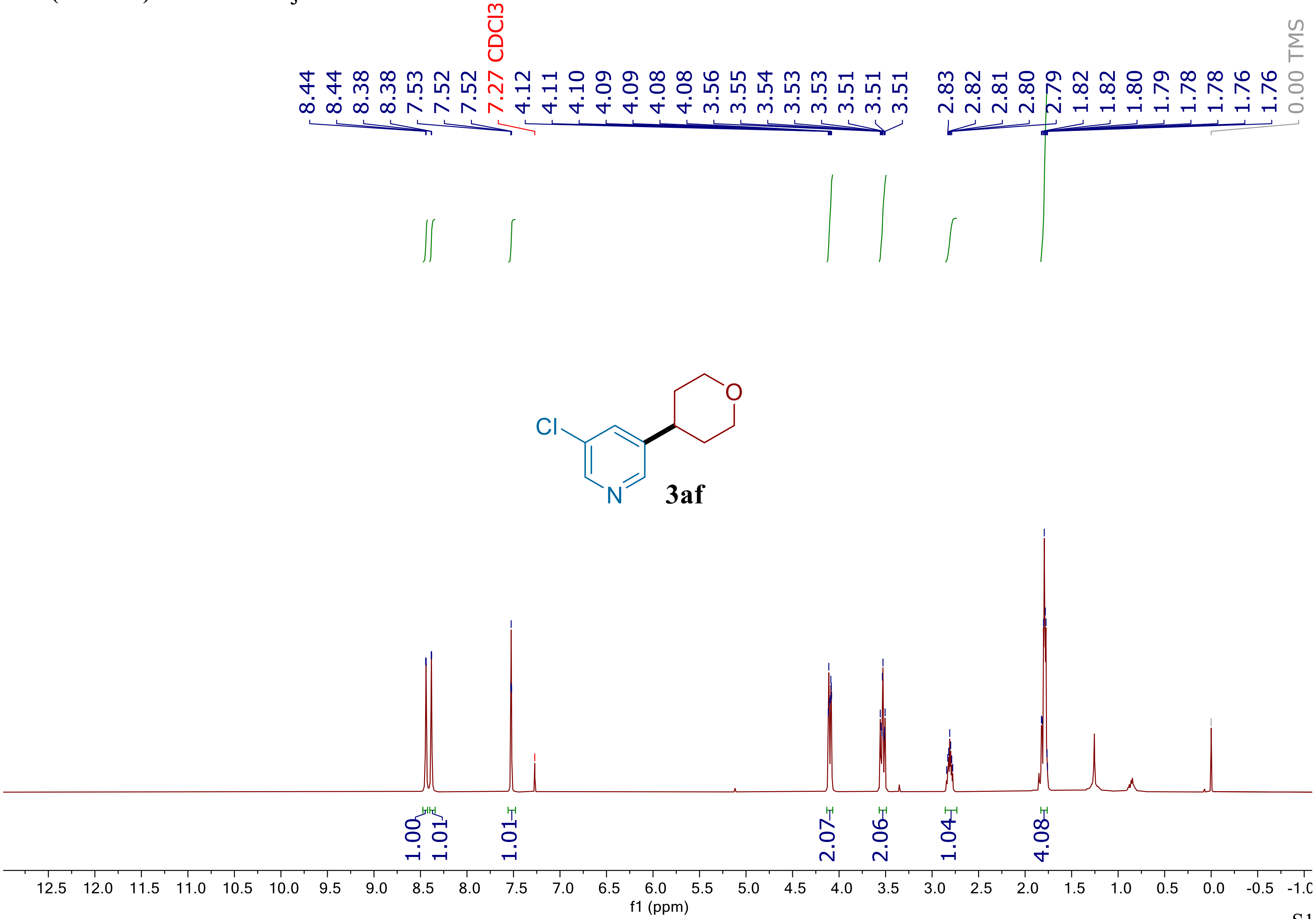

$^{13}C\{^{1}H\}$ NMR (126 MHz) of 3af in $CDCl_3$

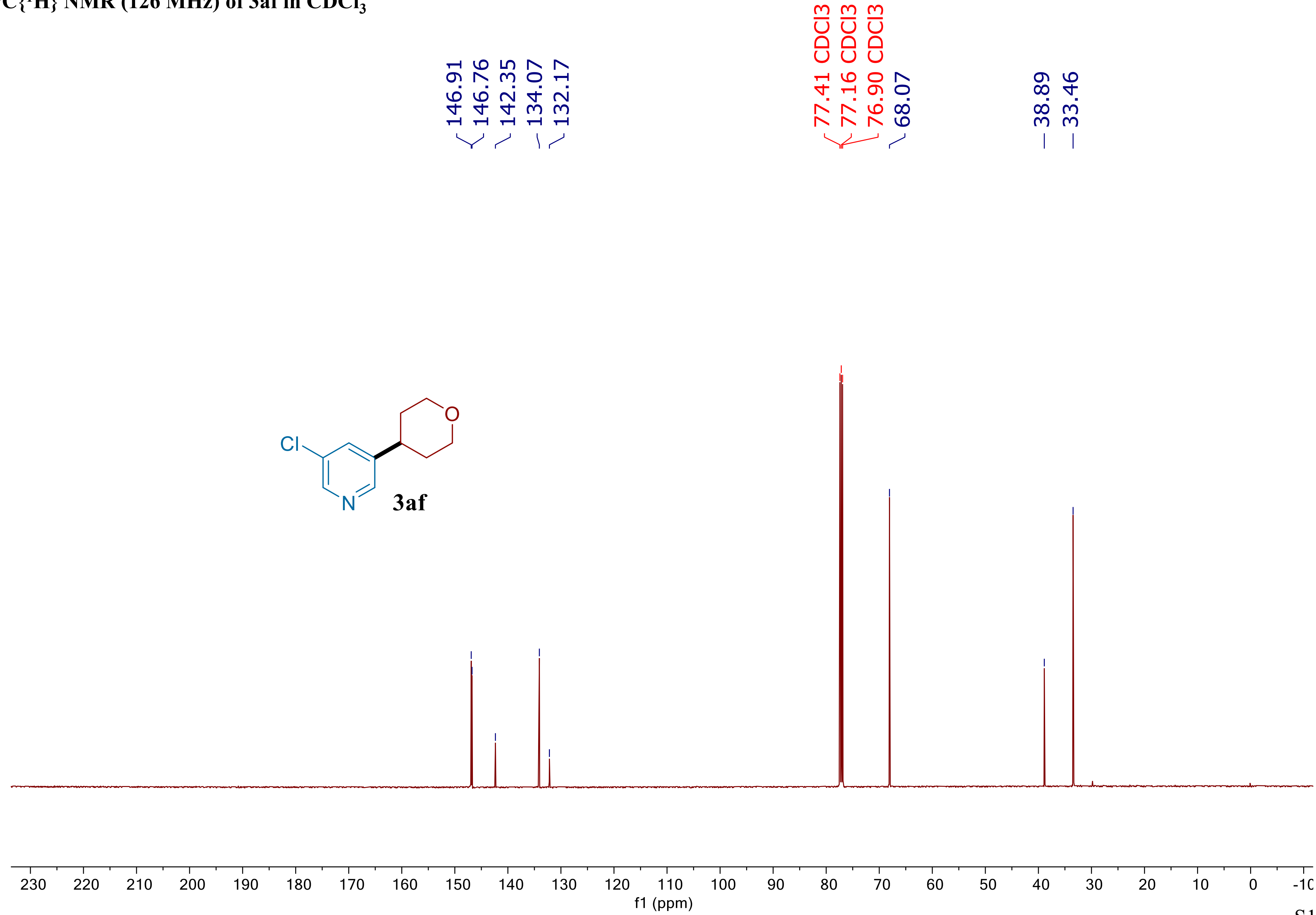

^{1}H NMR (500 MHz) of 3ag in $CDCl_3$

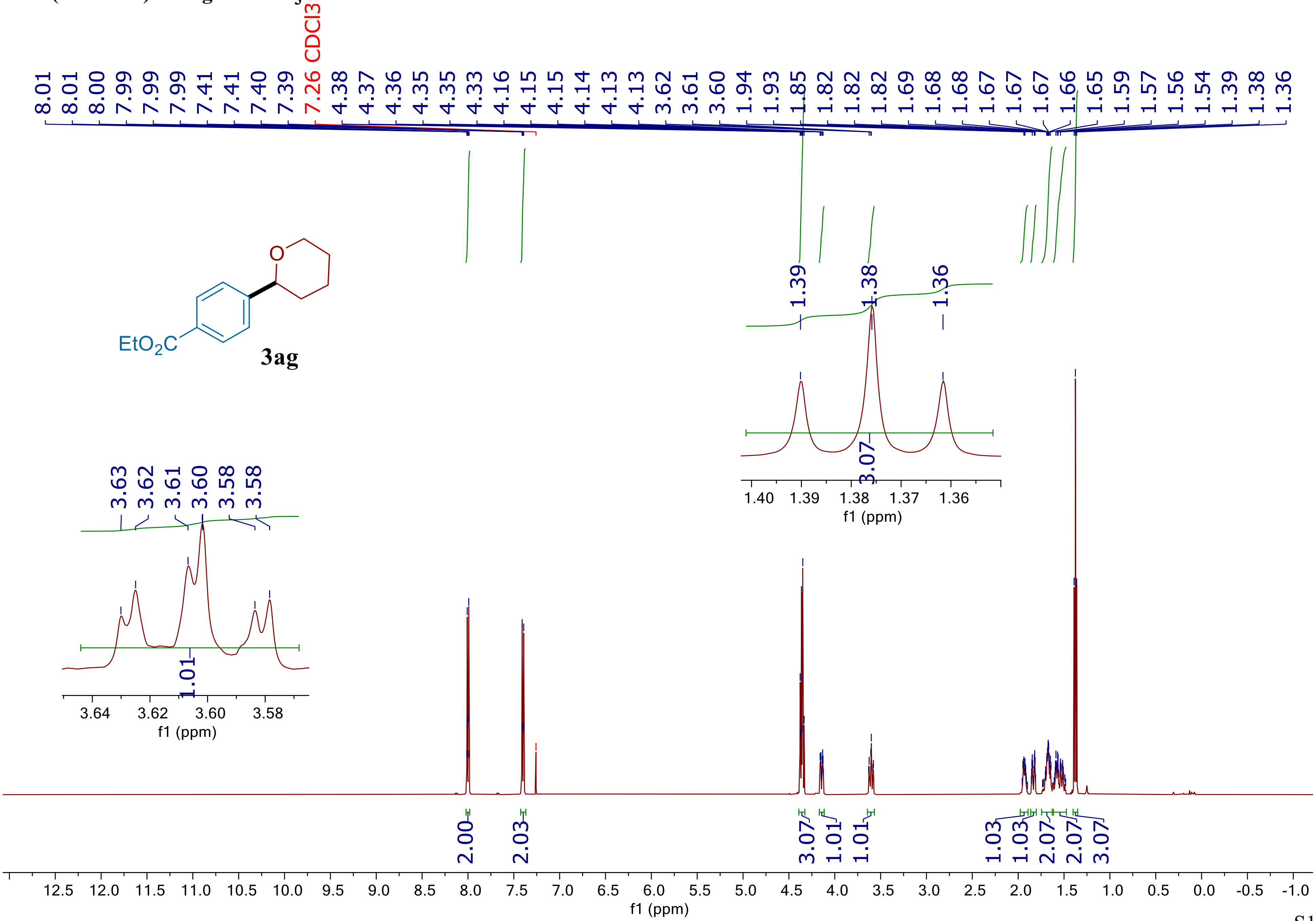

$^{13}C\{^{1}H\}$ NMR (126 MHz) of 3ag in $CDCl_3$

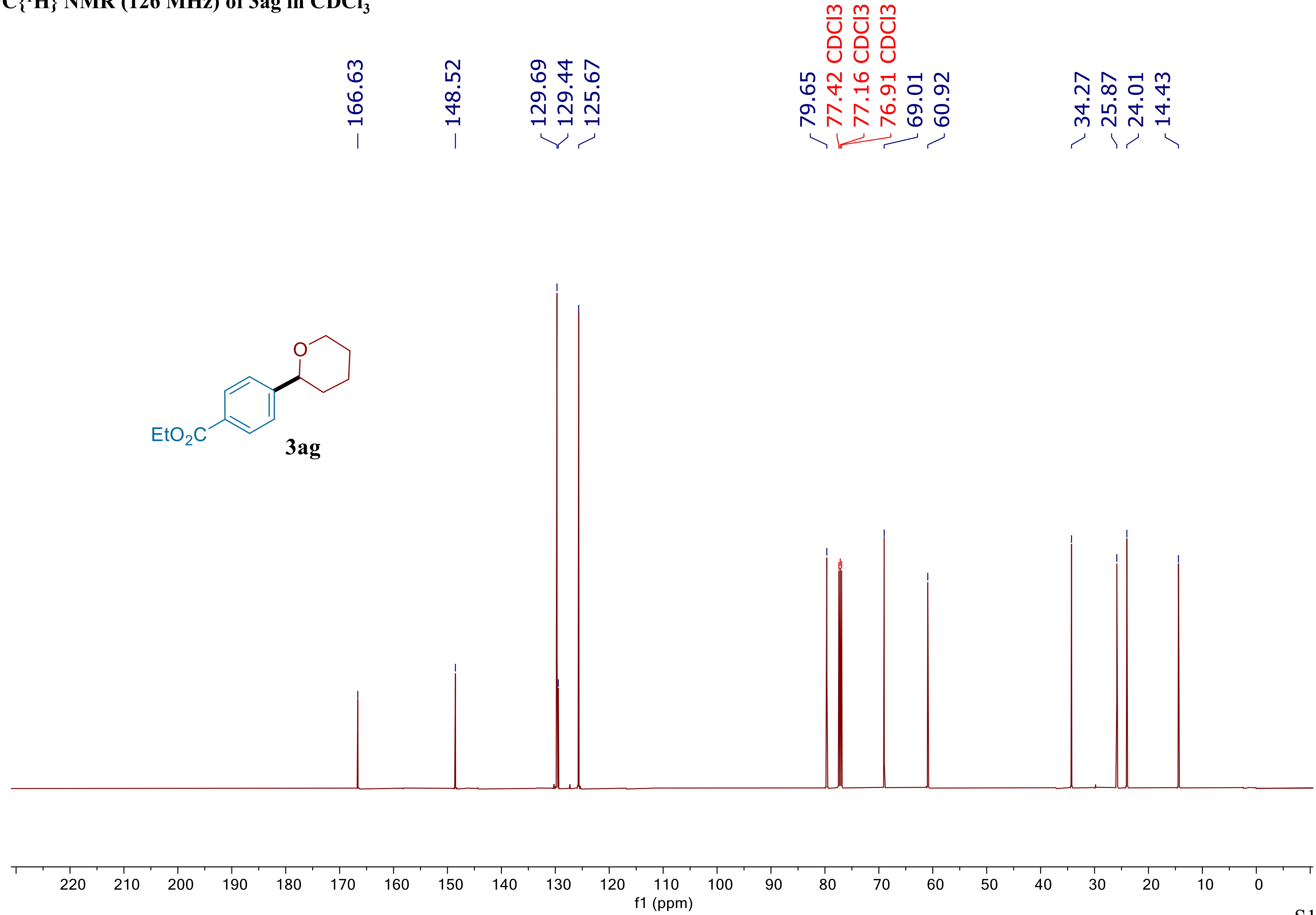

^{1}H NMR (500 MHz) of 3ah in $CDCl_3$

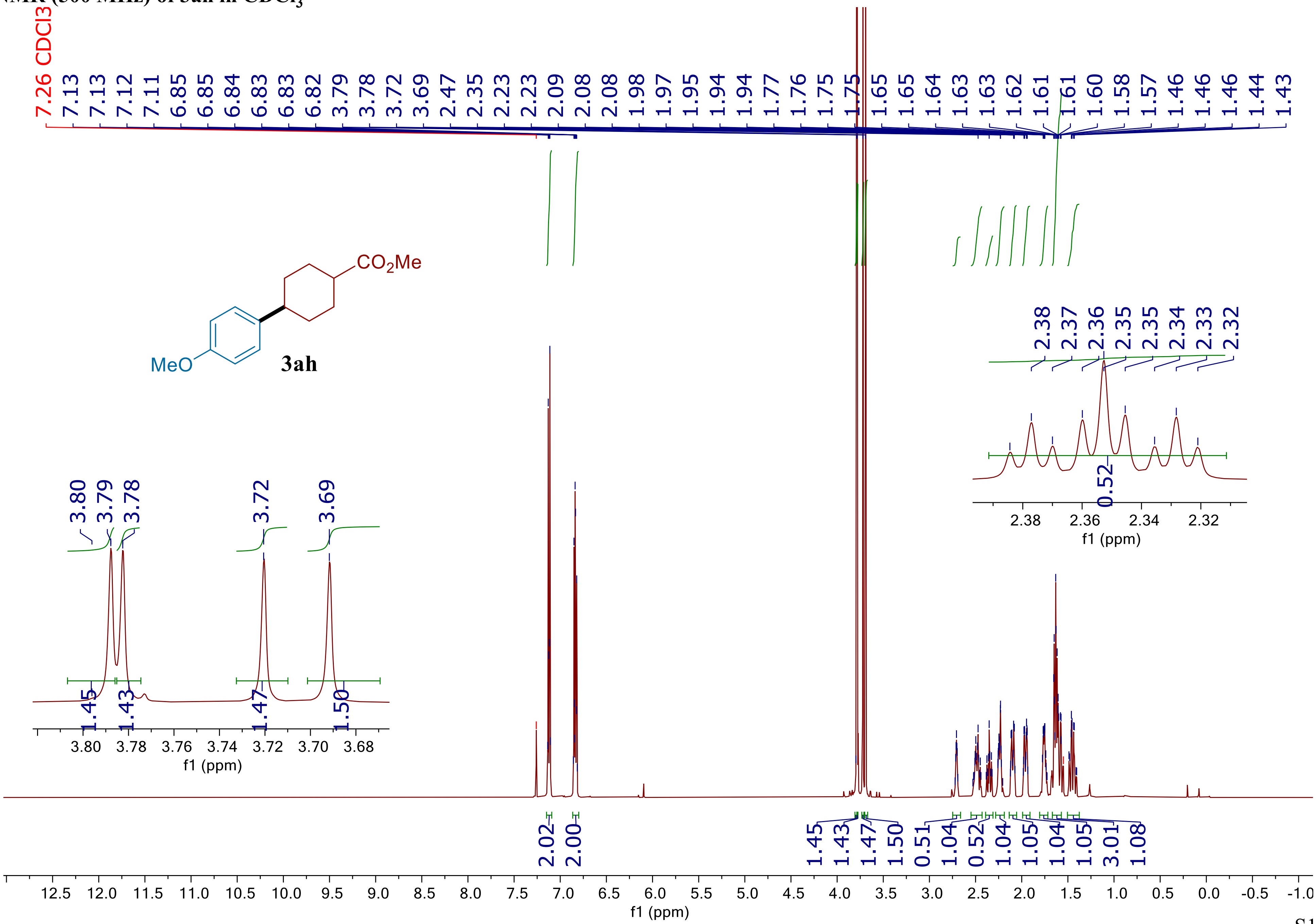

$^{13}C\{^{1}H\}$ NMR (126 MHz) of 3ah in $CDCl_3$

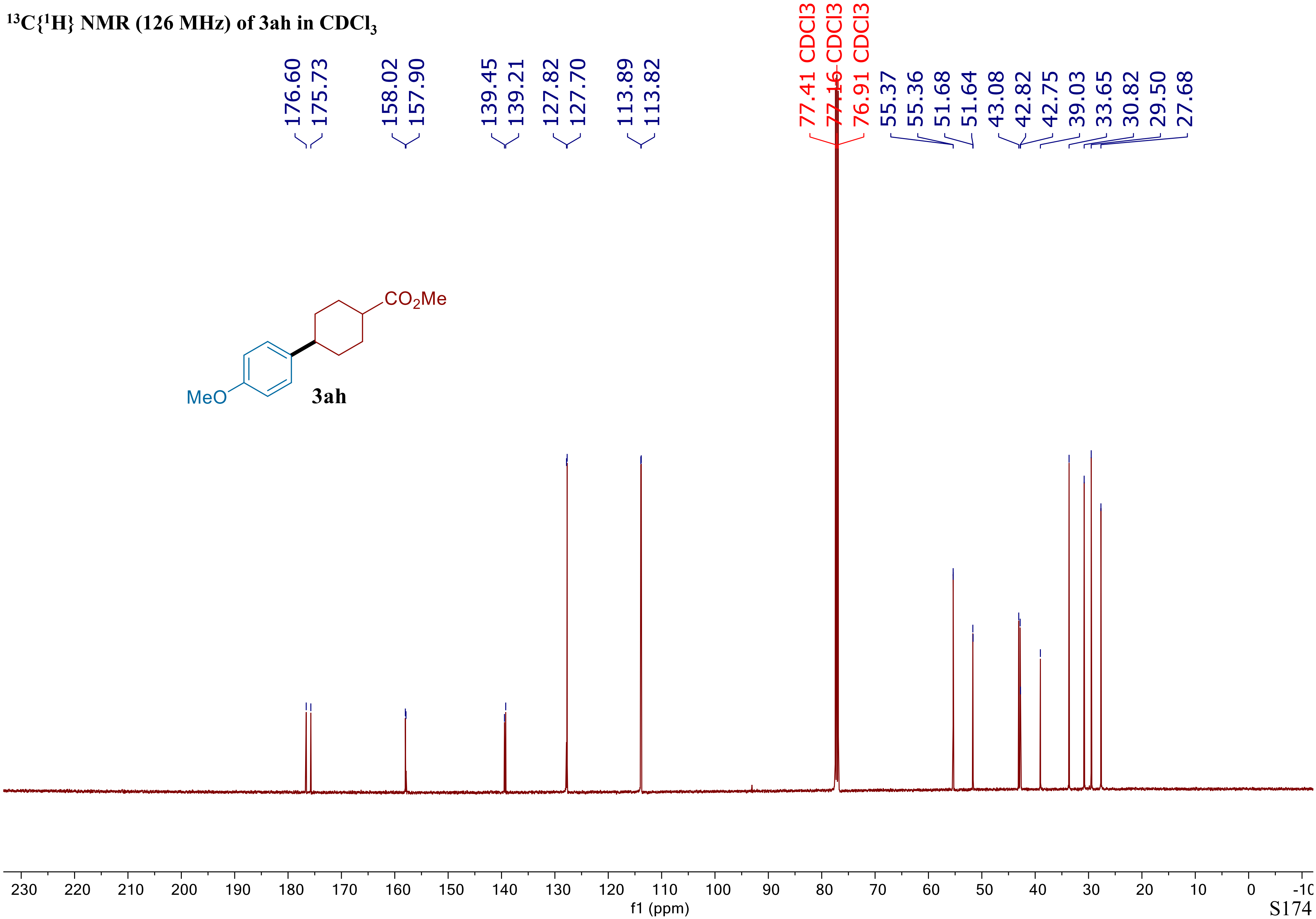

^{1}H NMR (500 MHz) of 3ai in $CDCl_3$

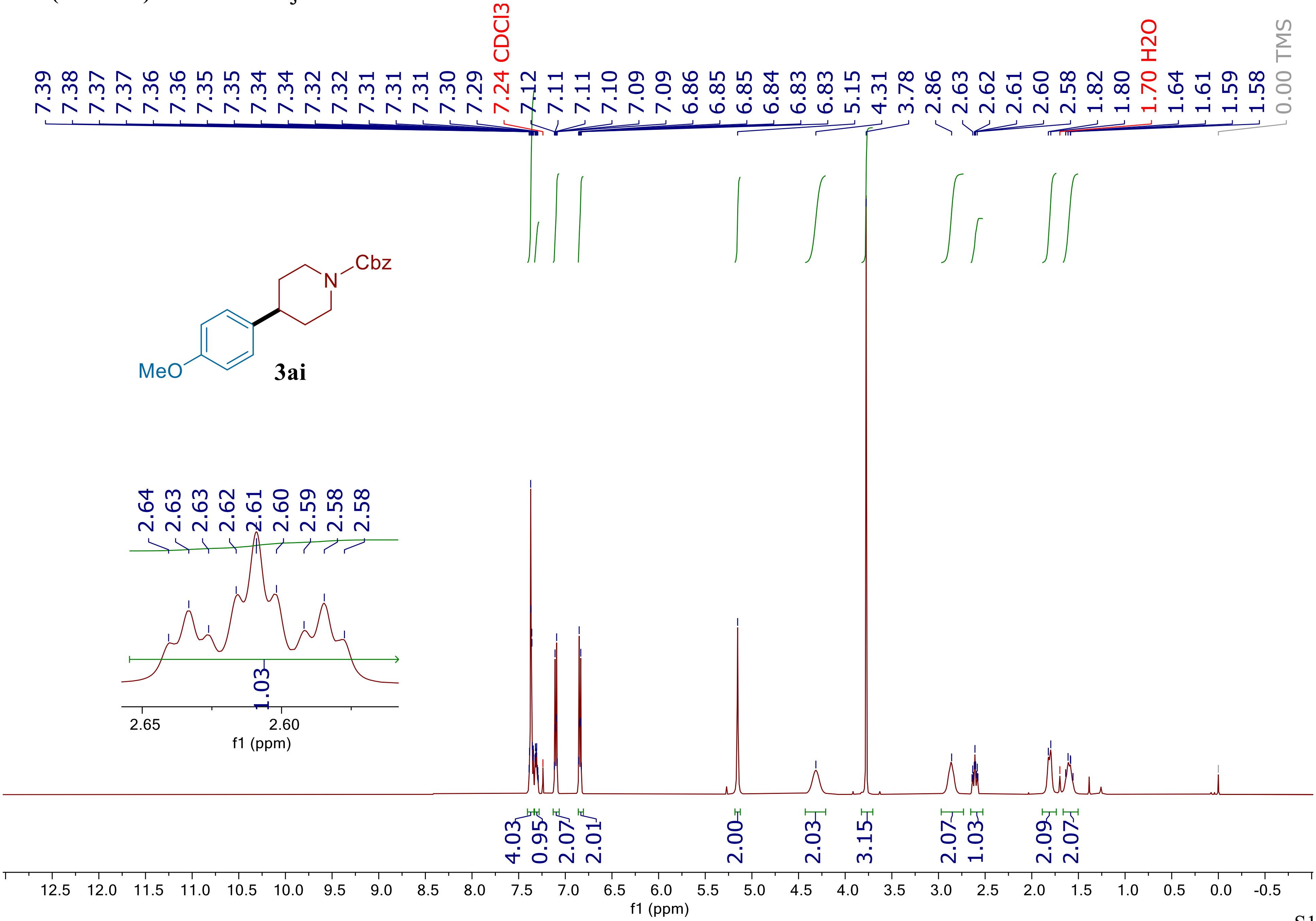

$^{13}C\{^{1}H\}$ NMR (126 MHz) of 3ai in $CDCl_3$

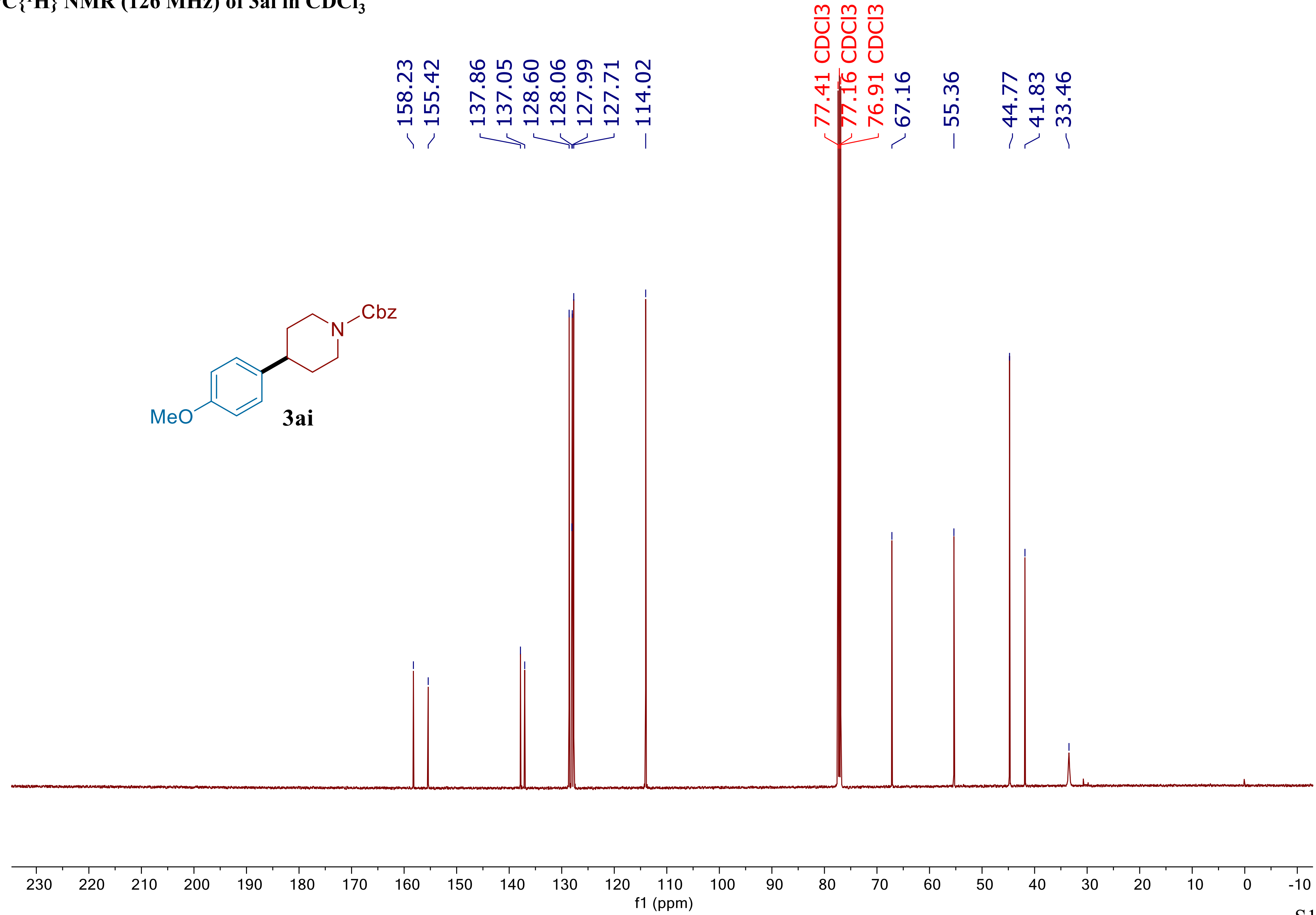

^1H NMR (500 MHz) of 3aj in $CDCl_3$

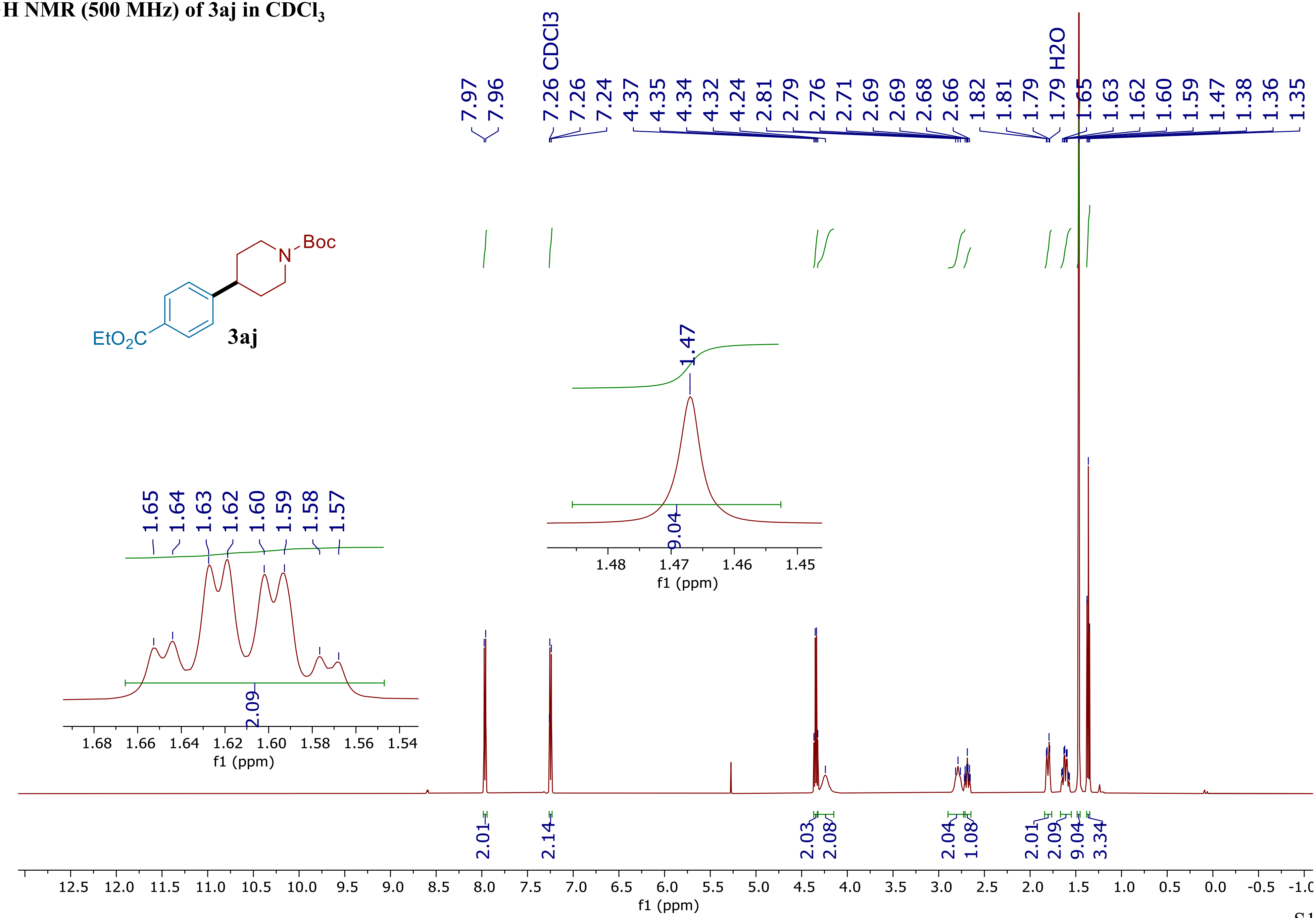

$^{13}C\{^1H\}$ NMR (126 MHz) of 3aj in $CDCl_3$

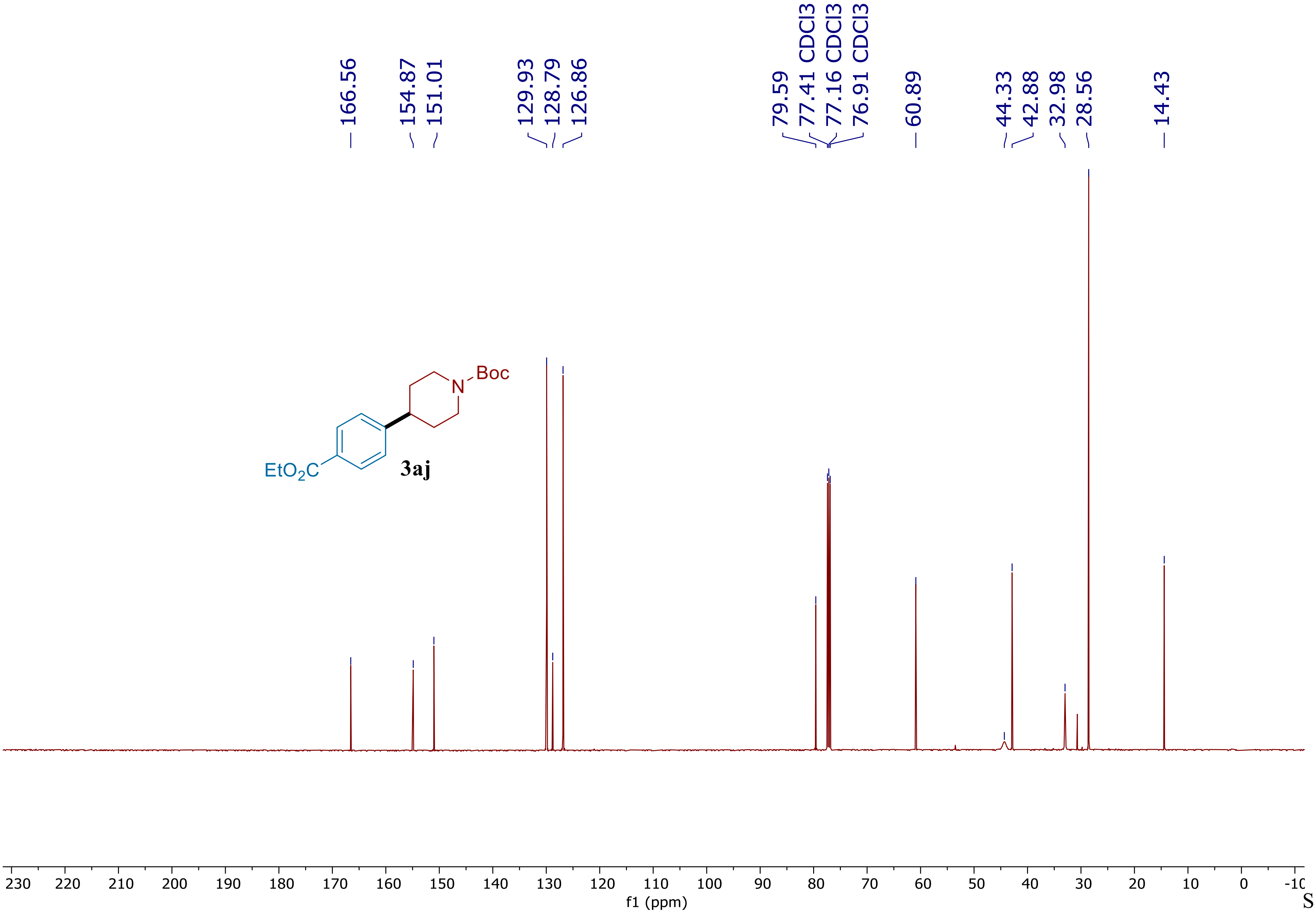

^{1}H NMR (500 MHz) of 3ak in $CDCl_3$

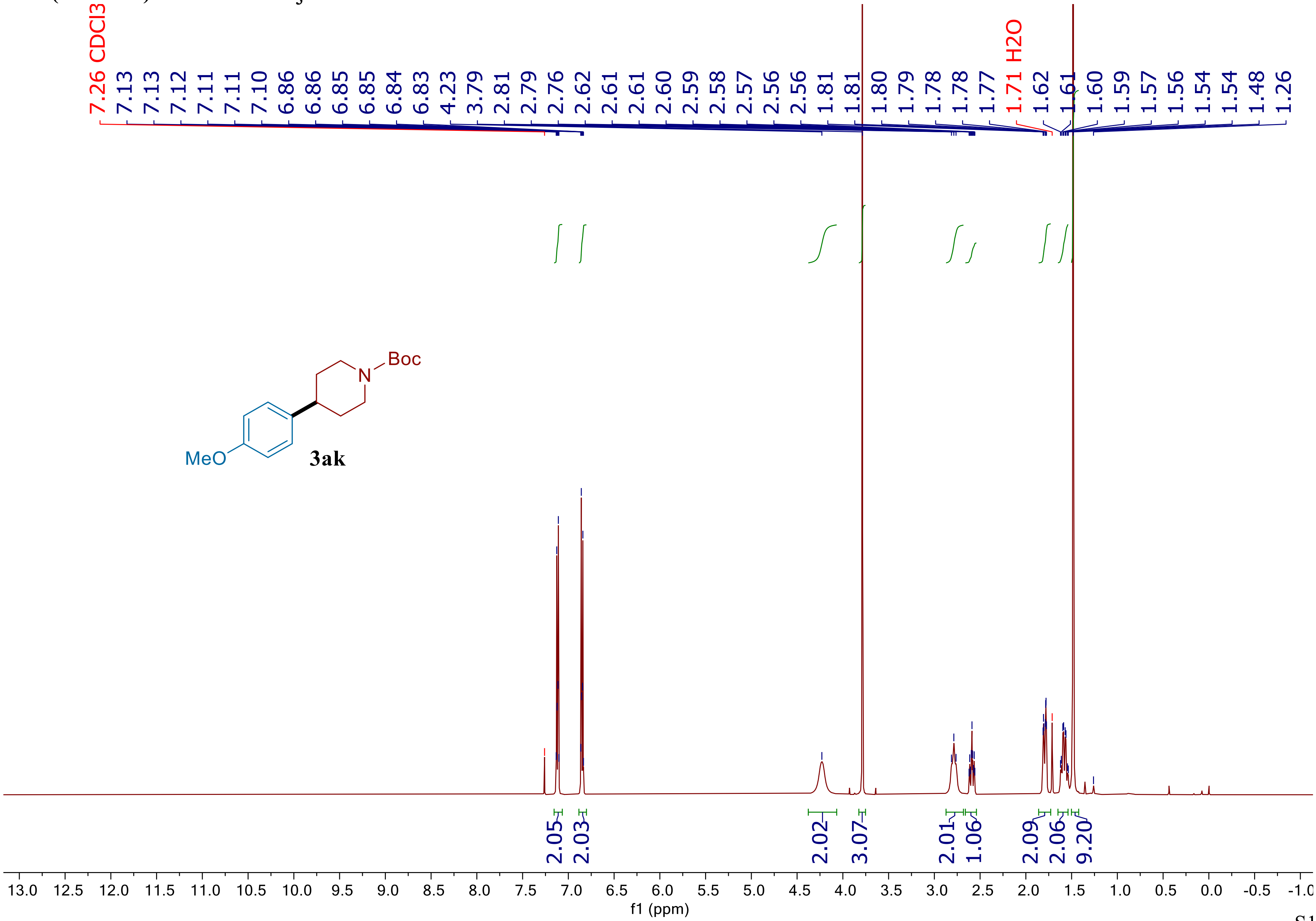

$^{13}C\{^{1}H\}$ NMR (126 MHz) of 3ak in $CDCl_3$

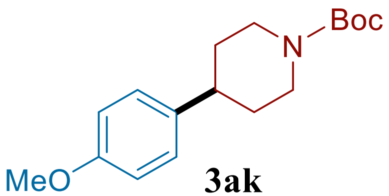

3ak

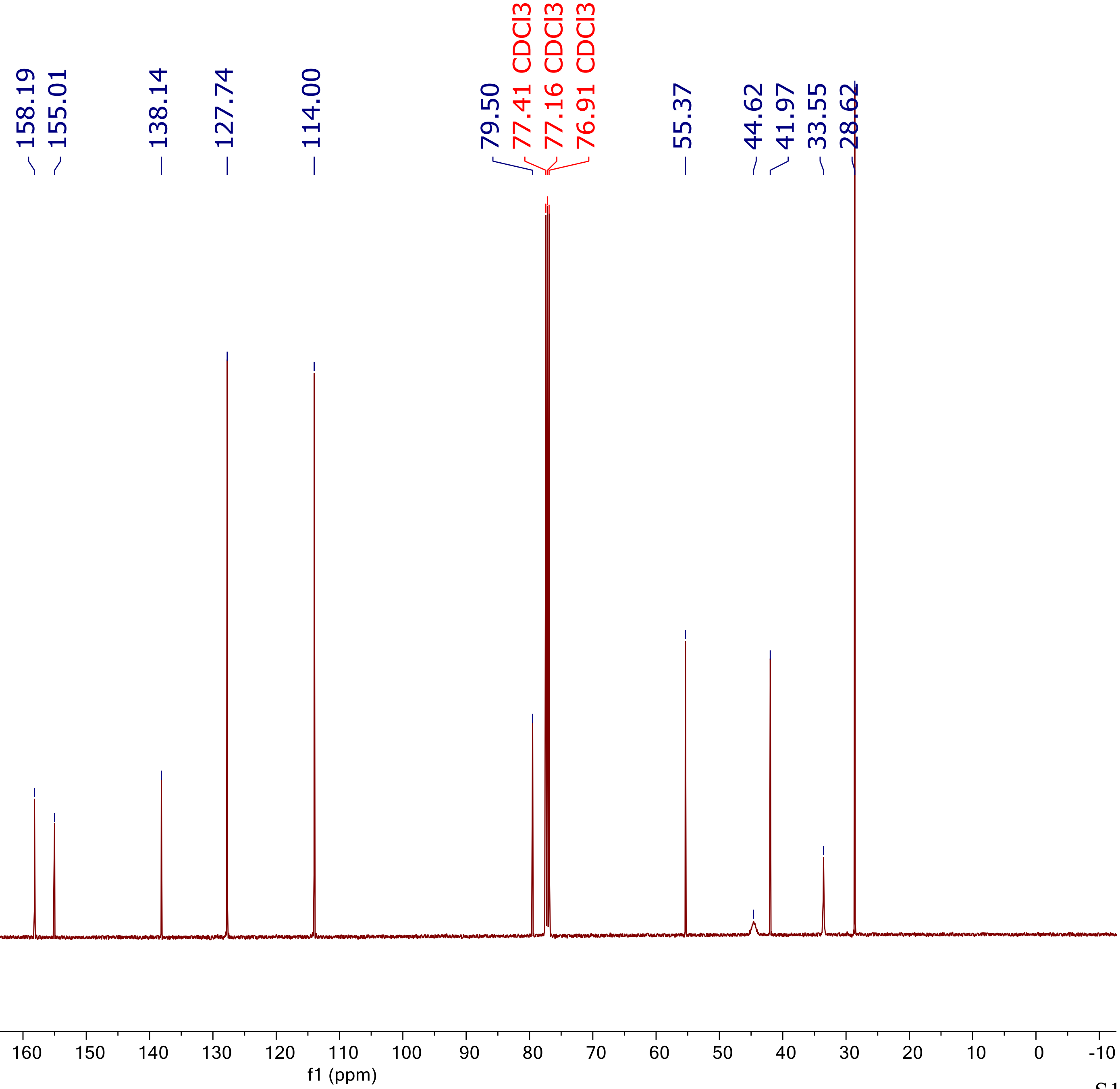

^{1}H NMR (500 MHz) of 3al in $CDCl_3$

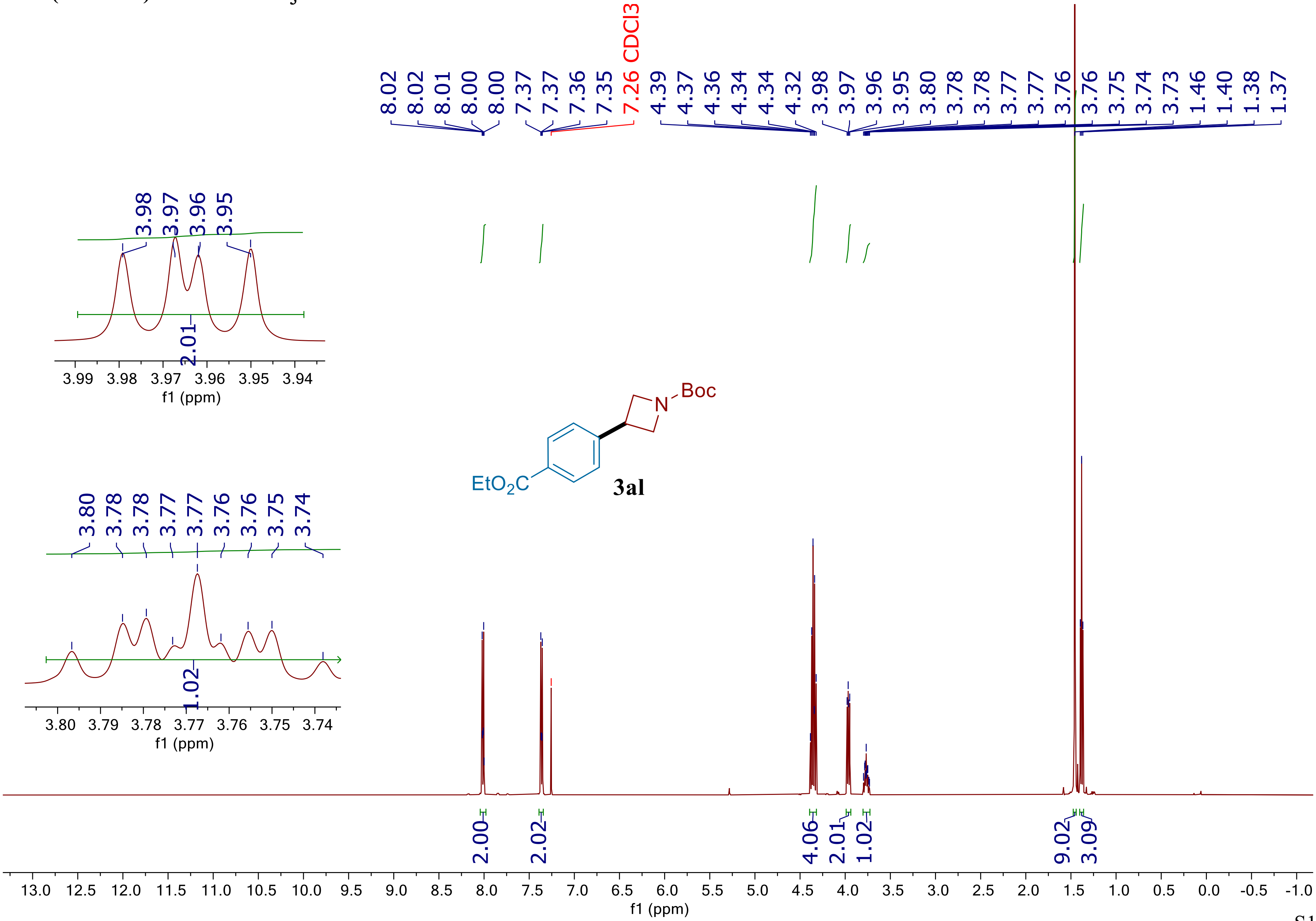

$^{13}C\{^{1}H\}$ NMR (126 MHz) of 3al in $CDCl_3$

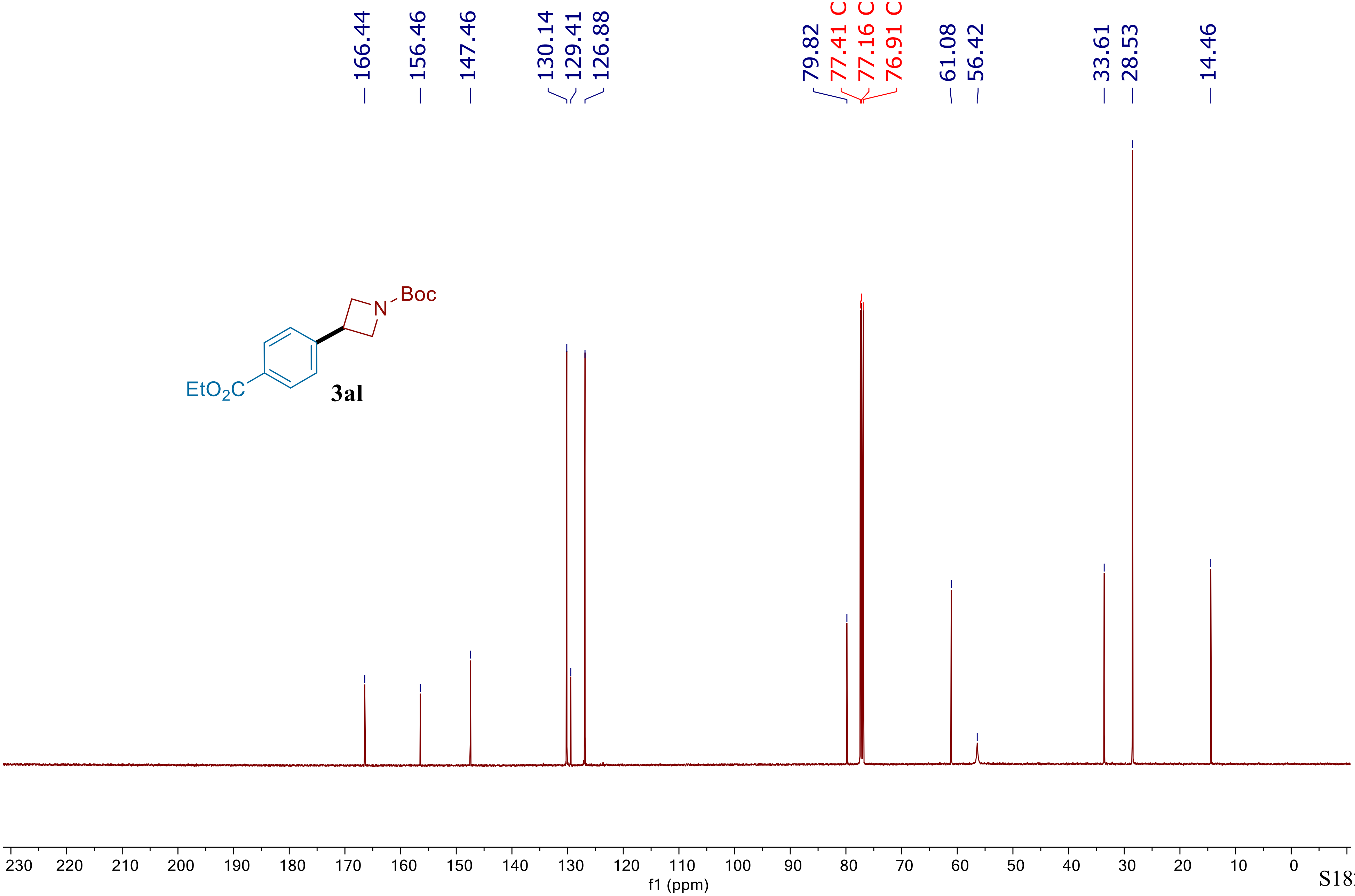

^{1}H NMR (500 MHz) of 3am in CDCl$_3$

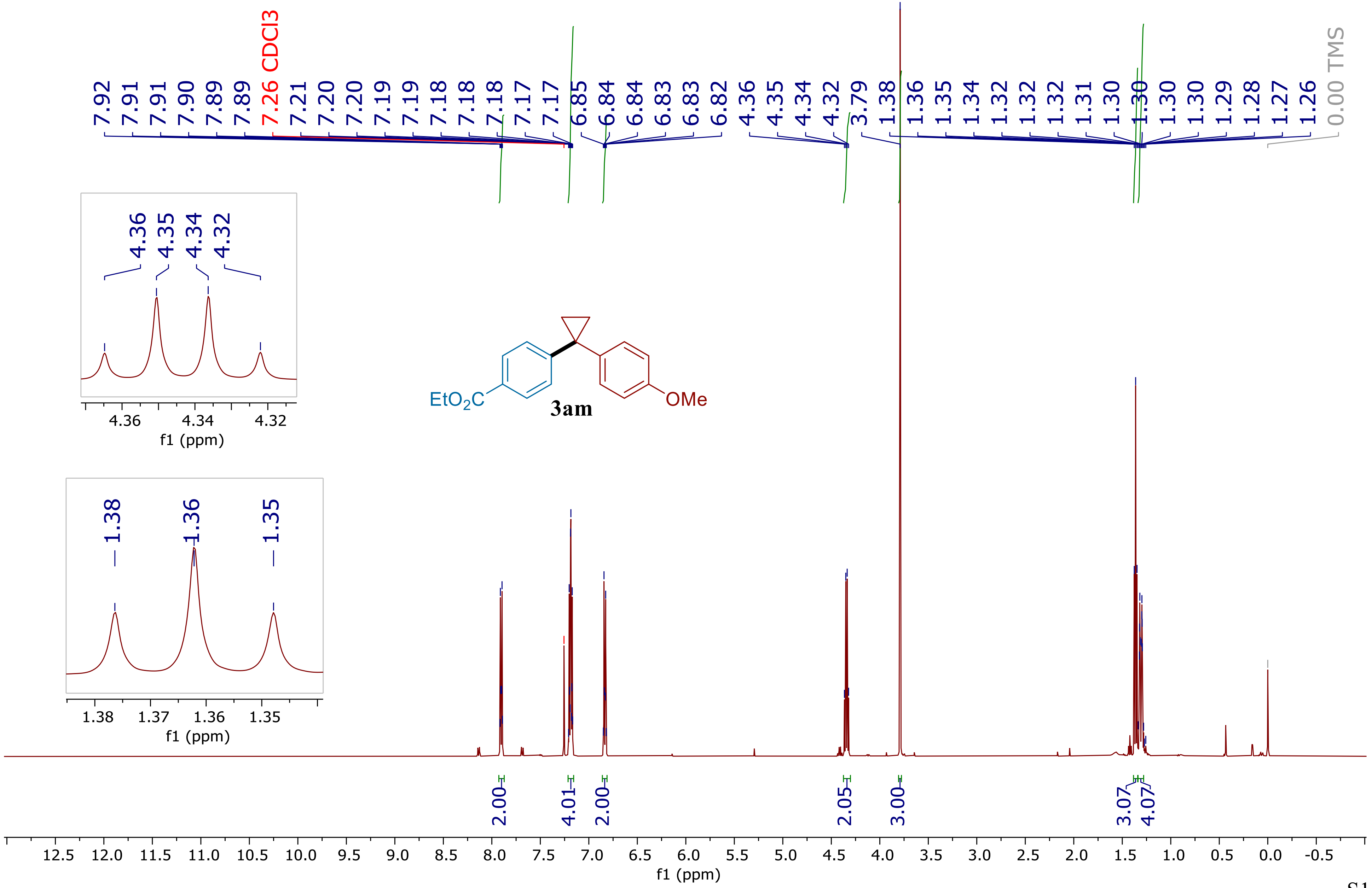

$^{13}C\{^{1}H\}$ NMR (126 MHz) of **3am** in $CDCl_3$

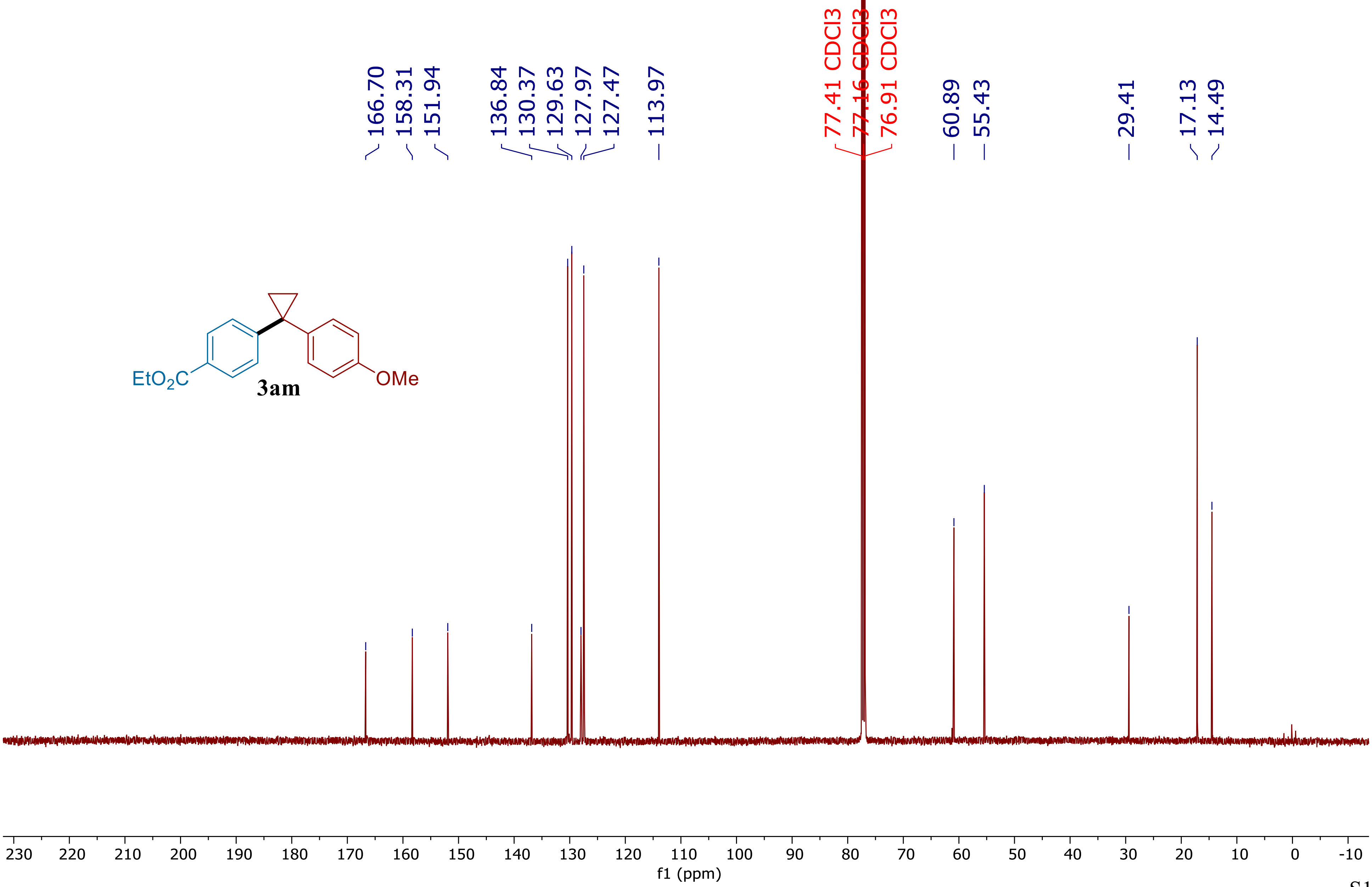

^1H NMR (500 MHz) of 3an in $CDCl_3$

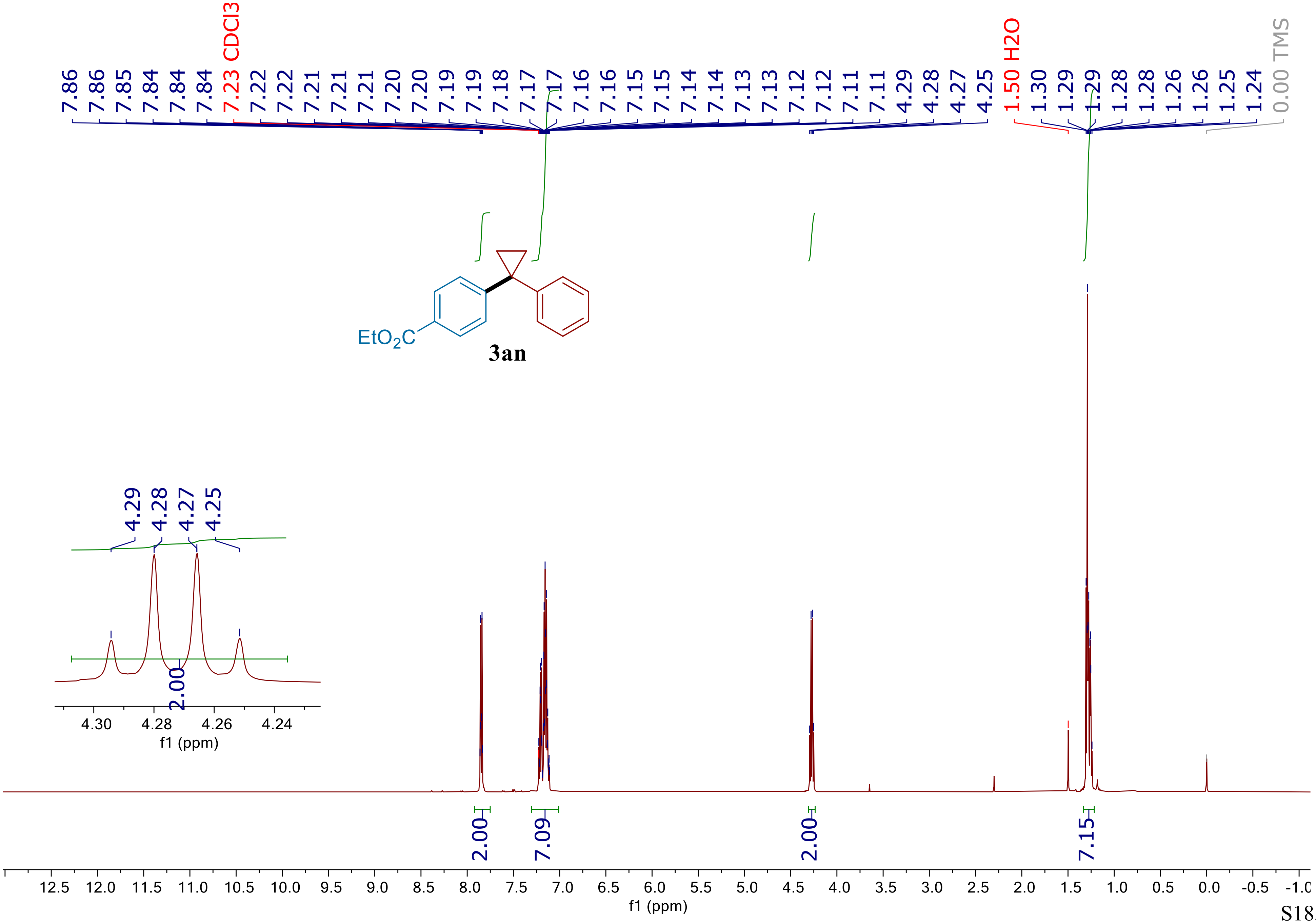

$^{13}C\{^{1}H\}$ NMR (126 MHz) of 3an in $CDCl_3$

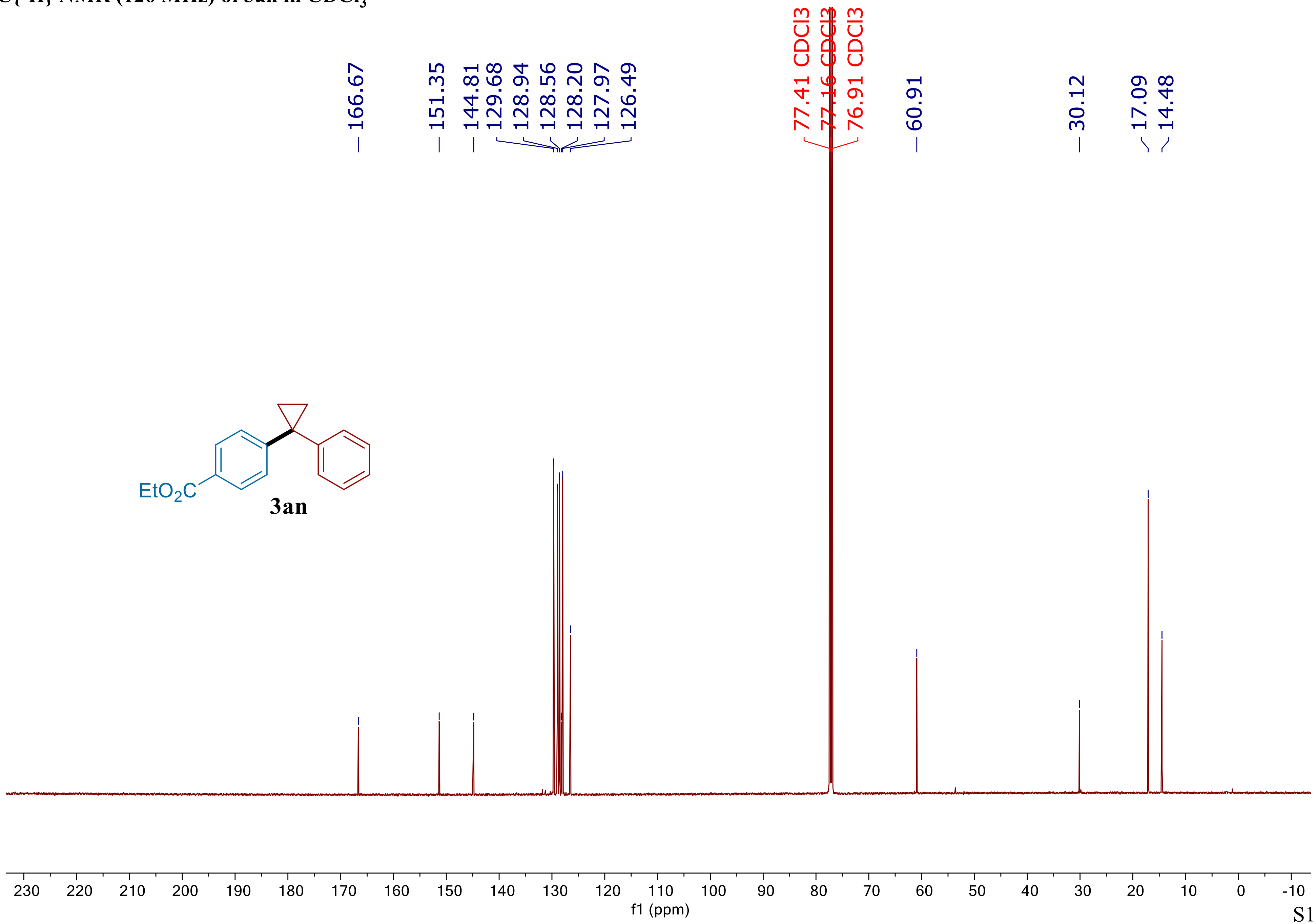

^{1}H NMR (400 MHz) of 3ao in $CDCl_3$

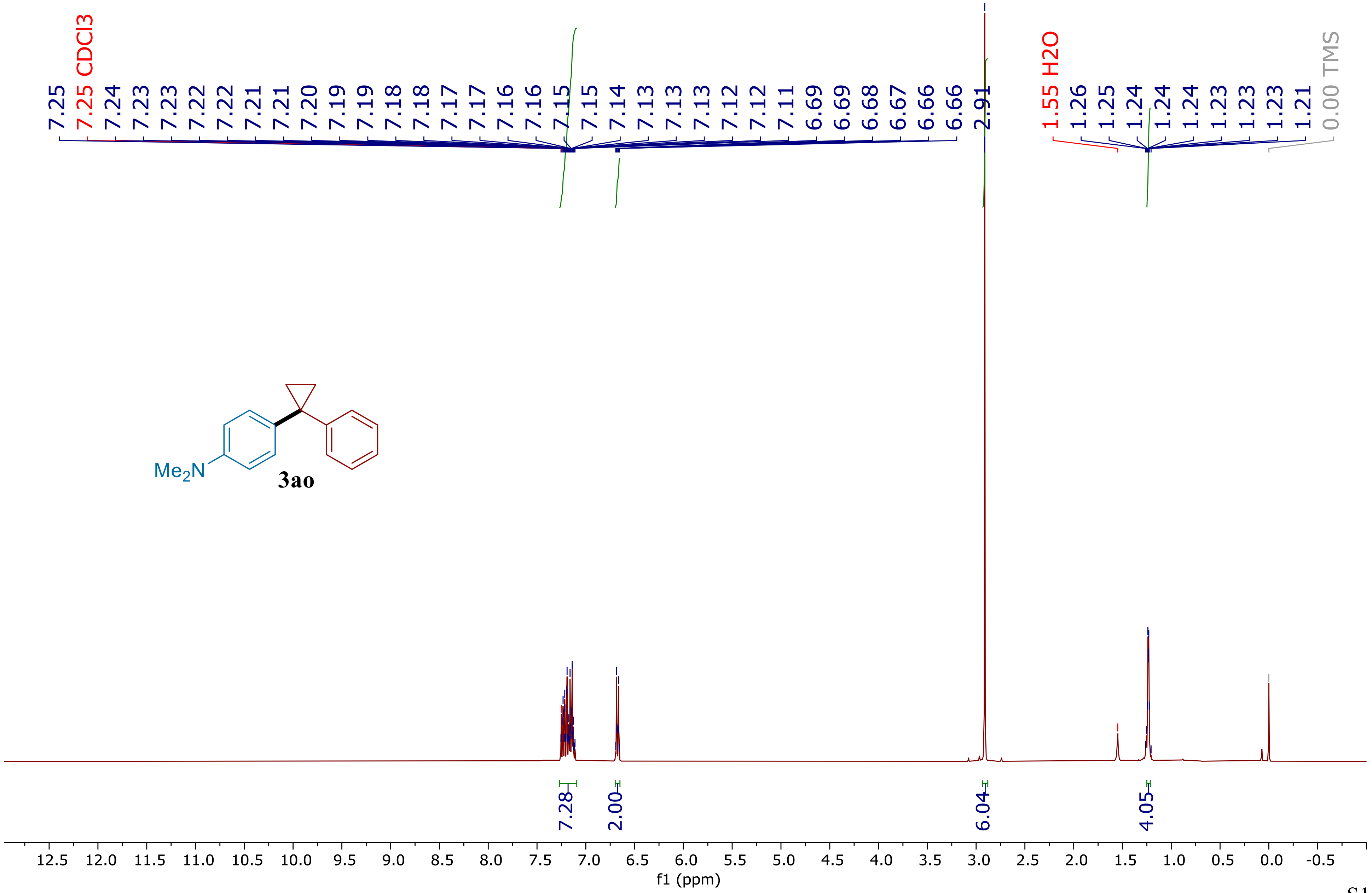

$^{13}C\{^1H\}$ NMR of 3ao (125 MHz) in $CDCl_3$

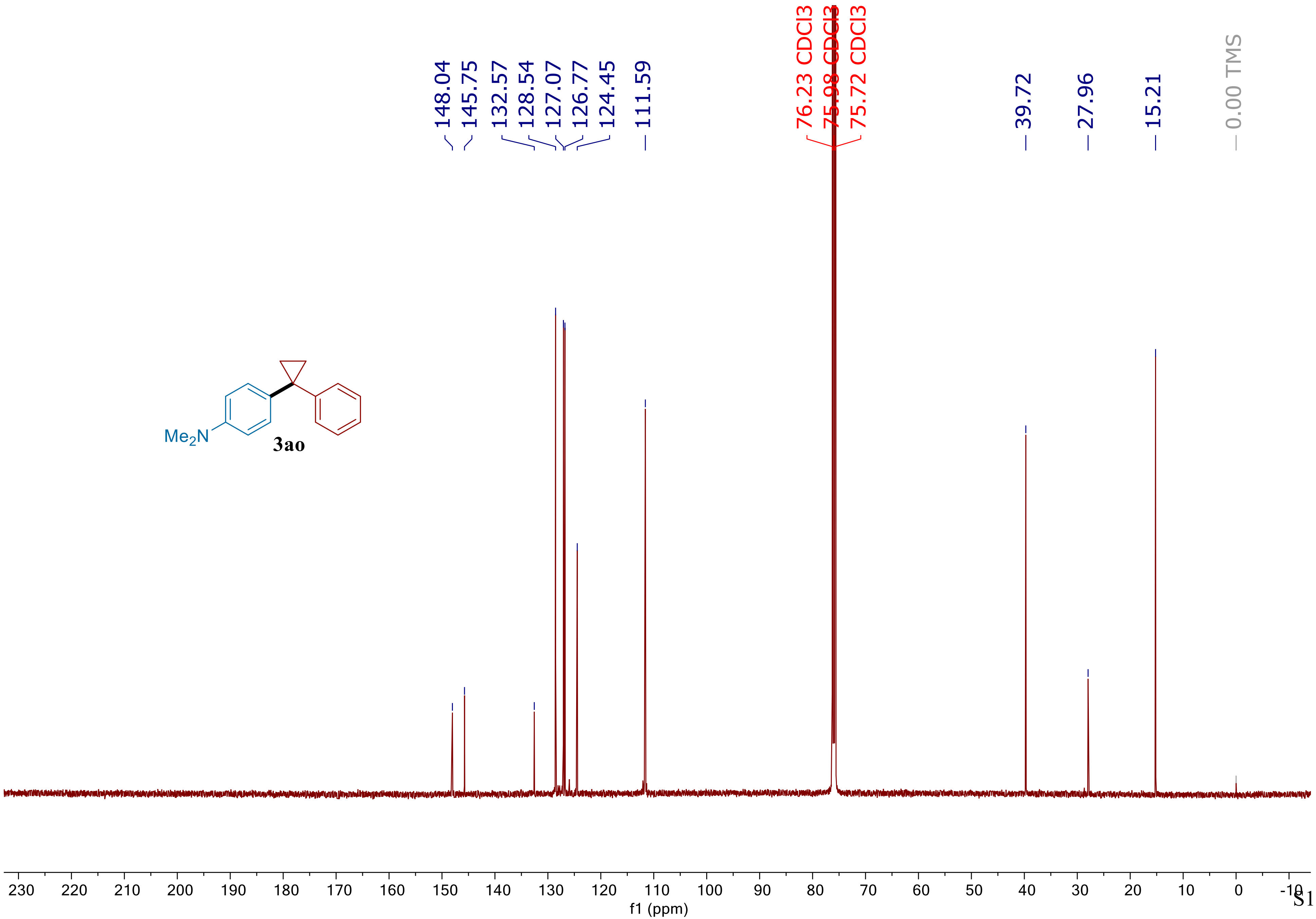

^{1}H NMR of 3ap (500 MHz) in $CDCl_3$

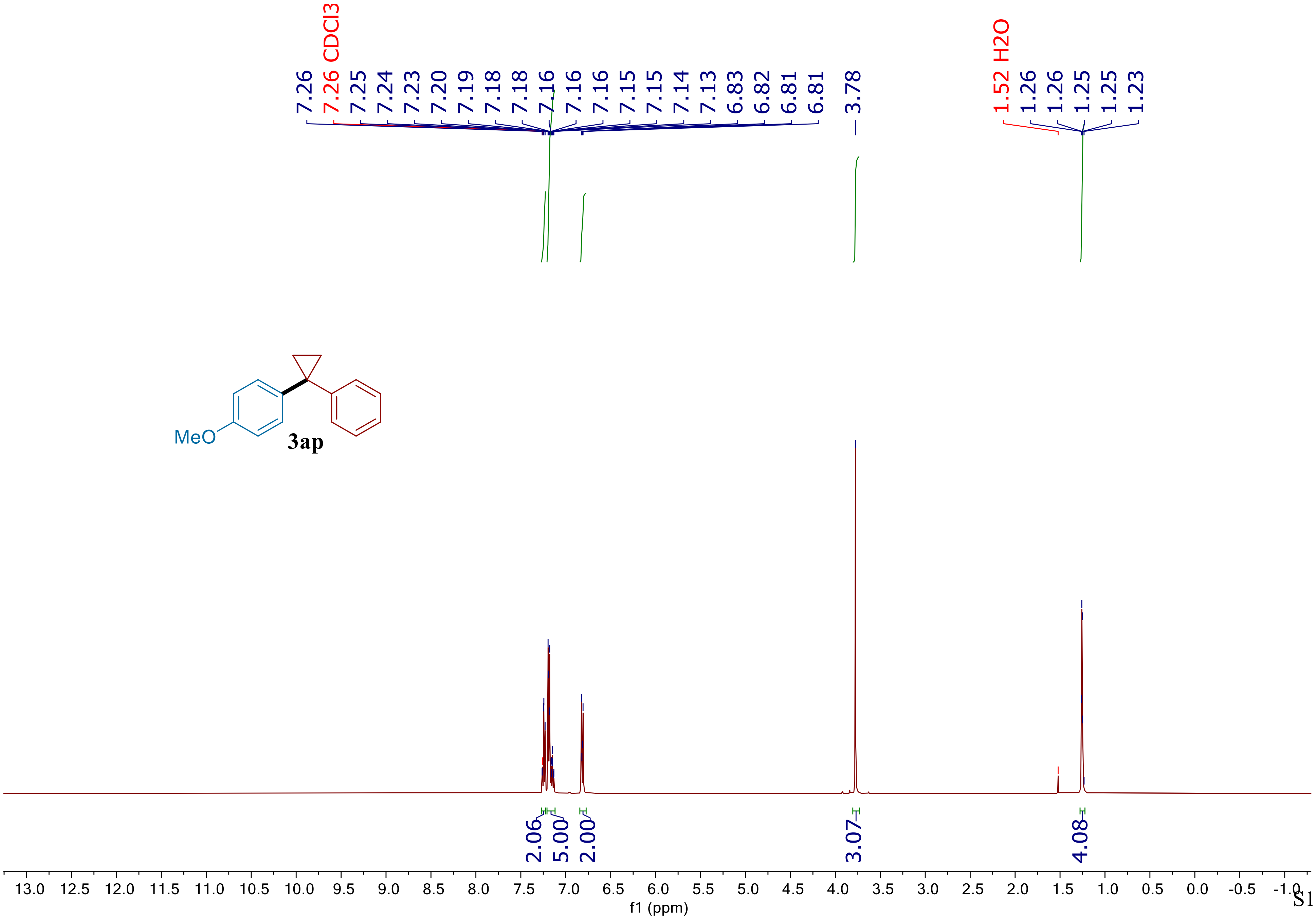

$^{13}C\{^{1}H\}$ NMR of 3ap (125 MHz) in $CDCl_3$

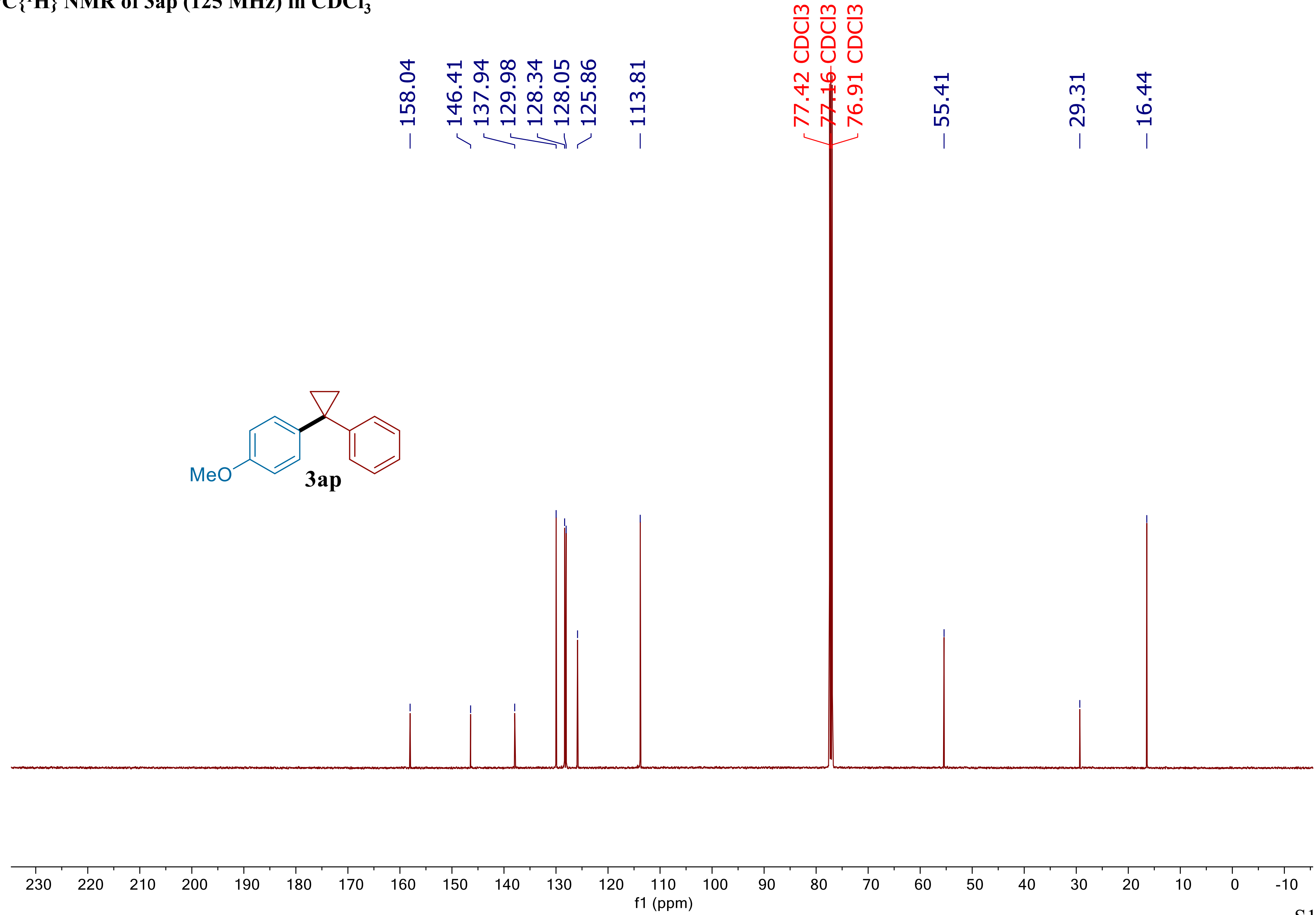

^{1}H NMR (500 MHz) of 3aq in $CDCl_3$

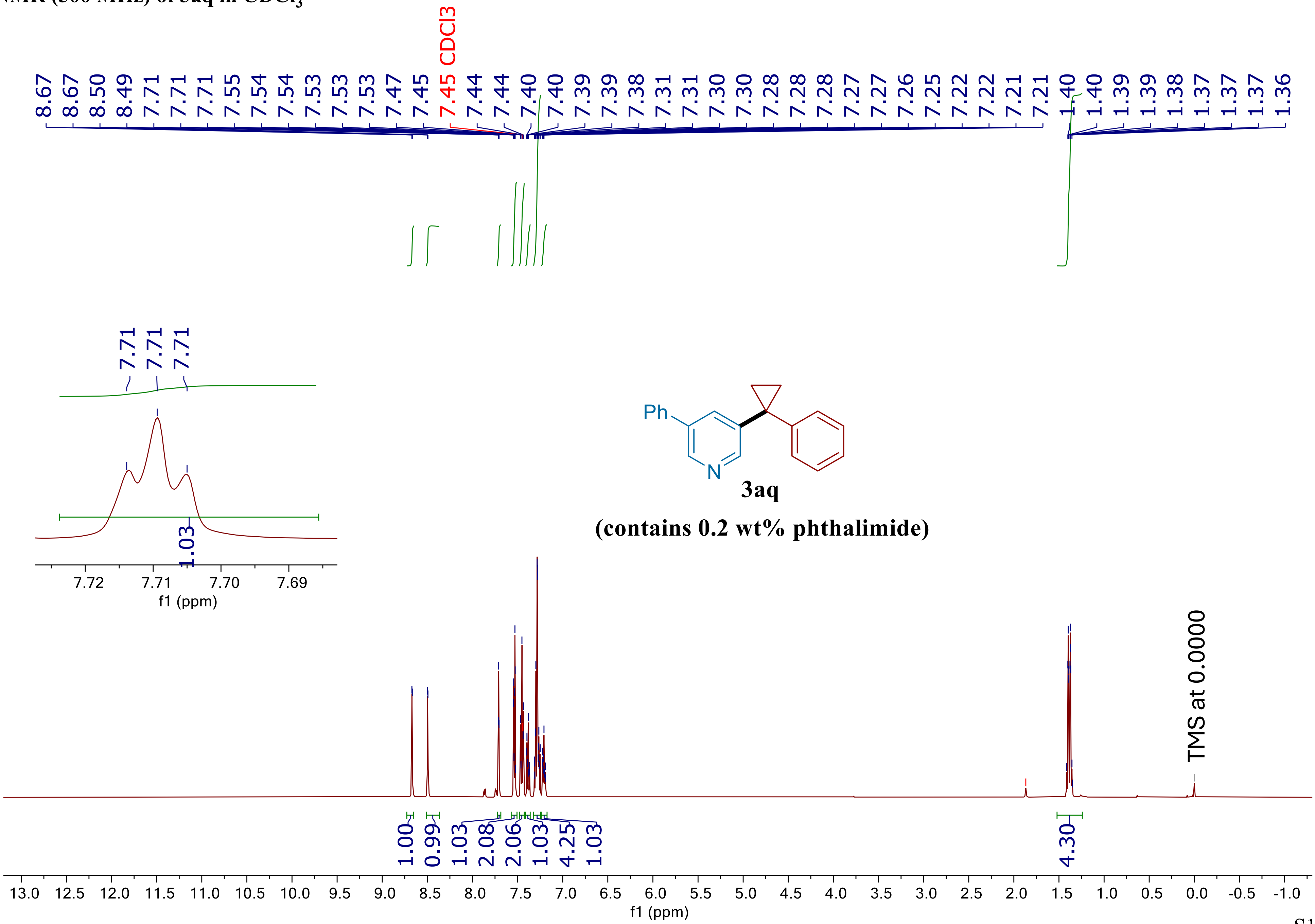

3aq

(contains 0.2 wt% phthalimide)

$^{13}C\{^{1}H\}$ NMR of 3aq (125 MHz) in $CDCl_3$

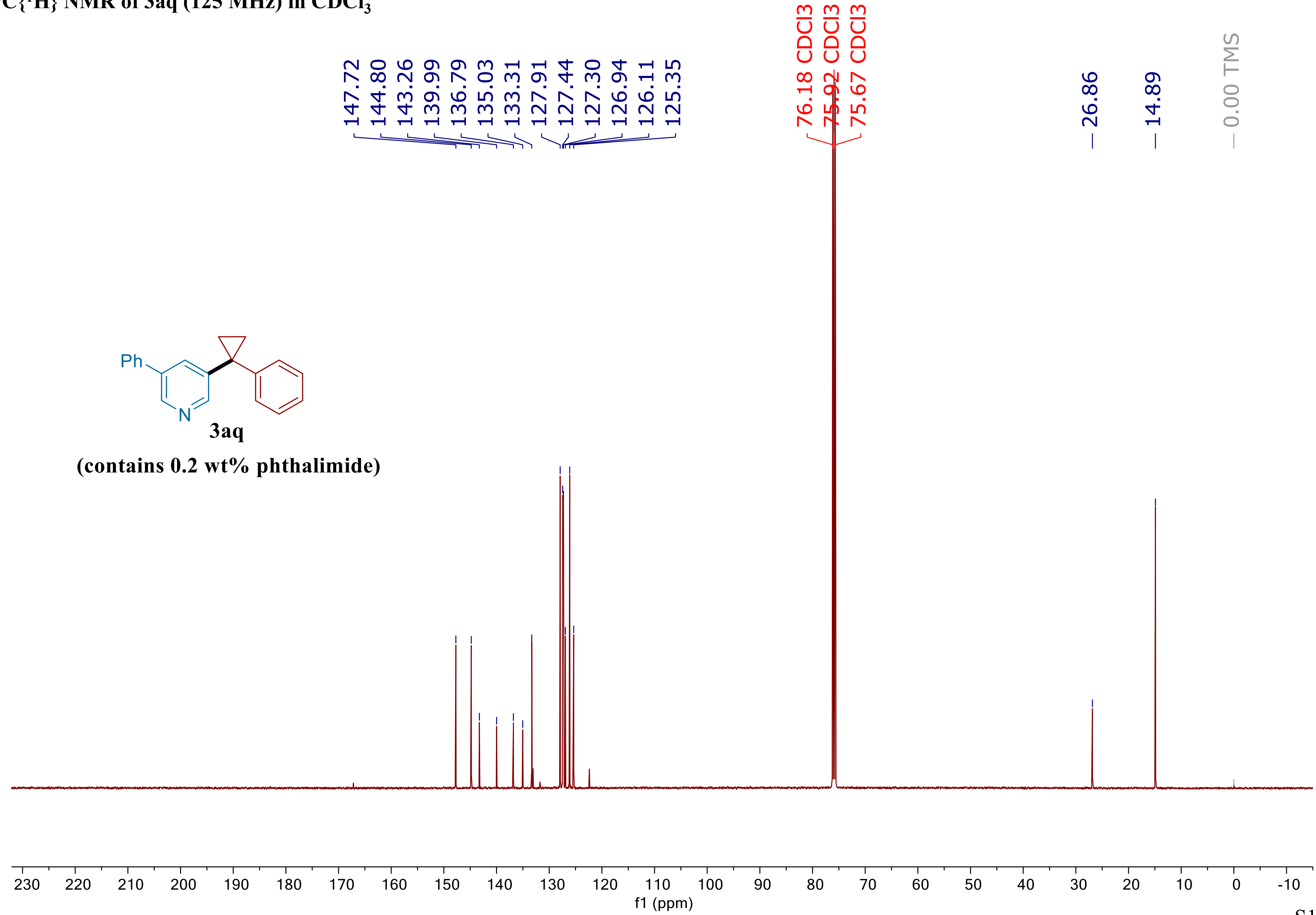

3aq

(contains 0.2 wt% phthalimide)

^1H NMR (500 MHz) of 3ar in $CDCl_3$

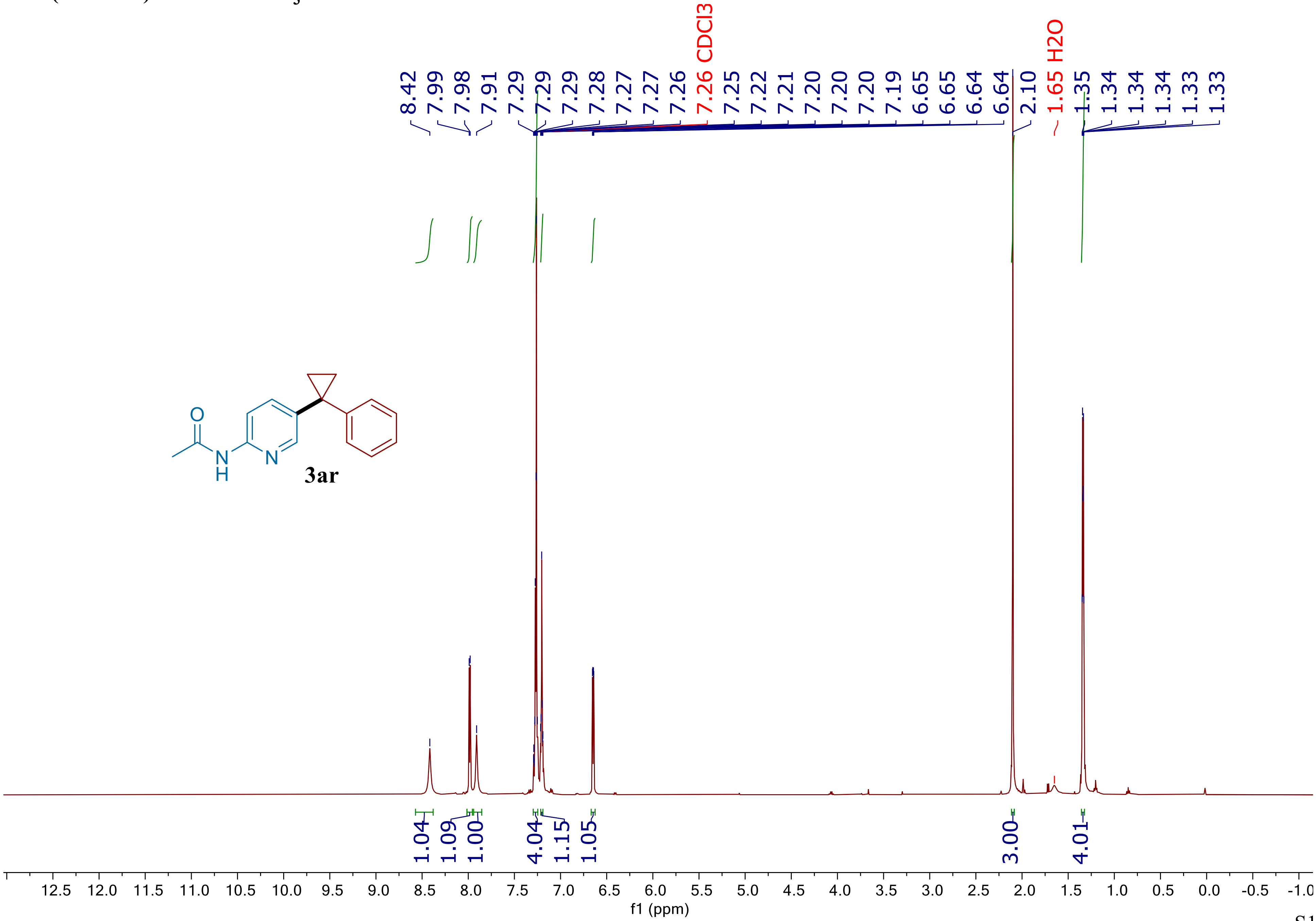

$^{13}C\{^{1}H\}$ NMR (126 MHz) of 3ar in $CDCl_3$

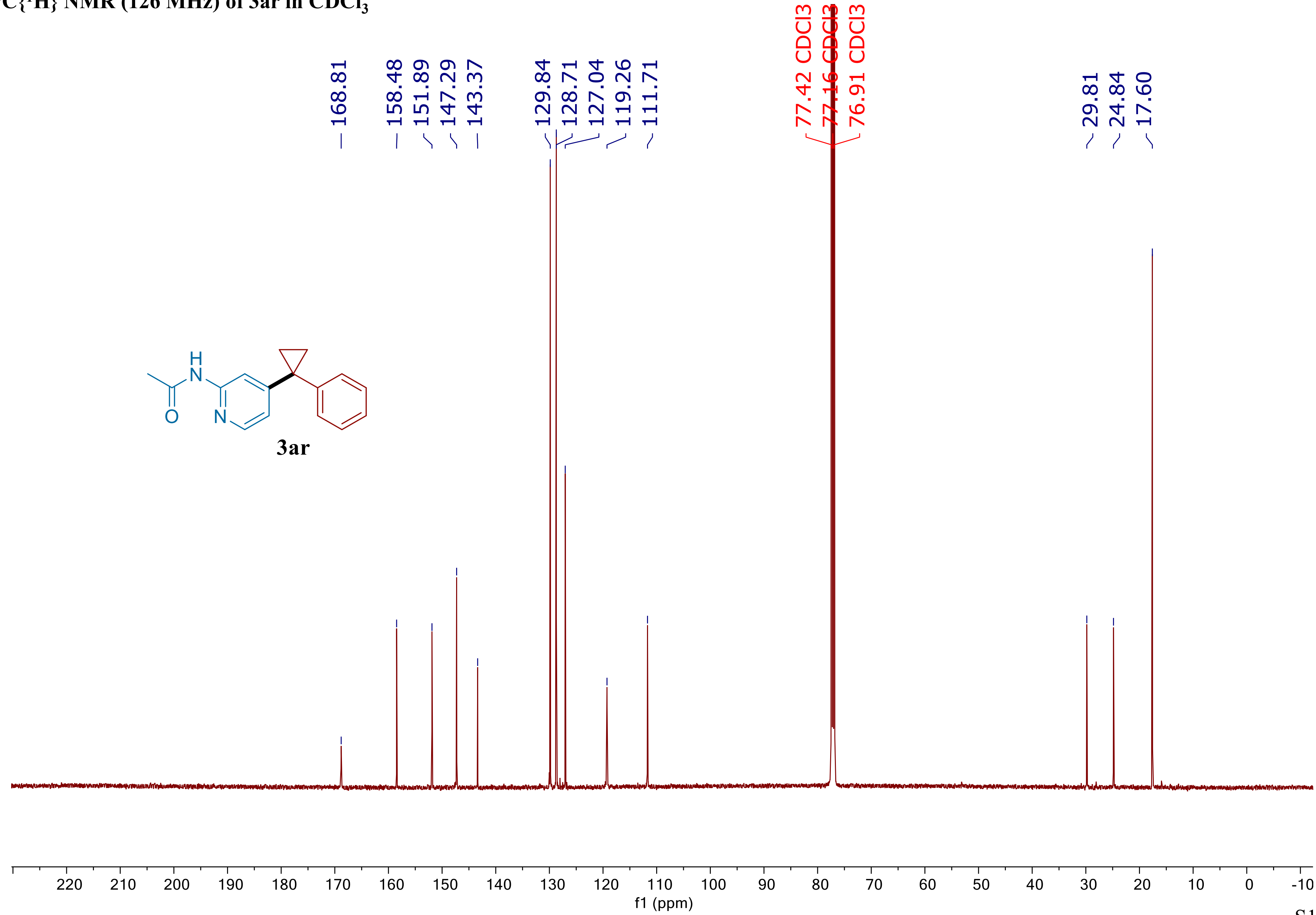

^{1}H NMR (500 MHz) of 3as in $CDCl_3$

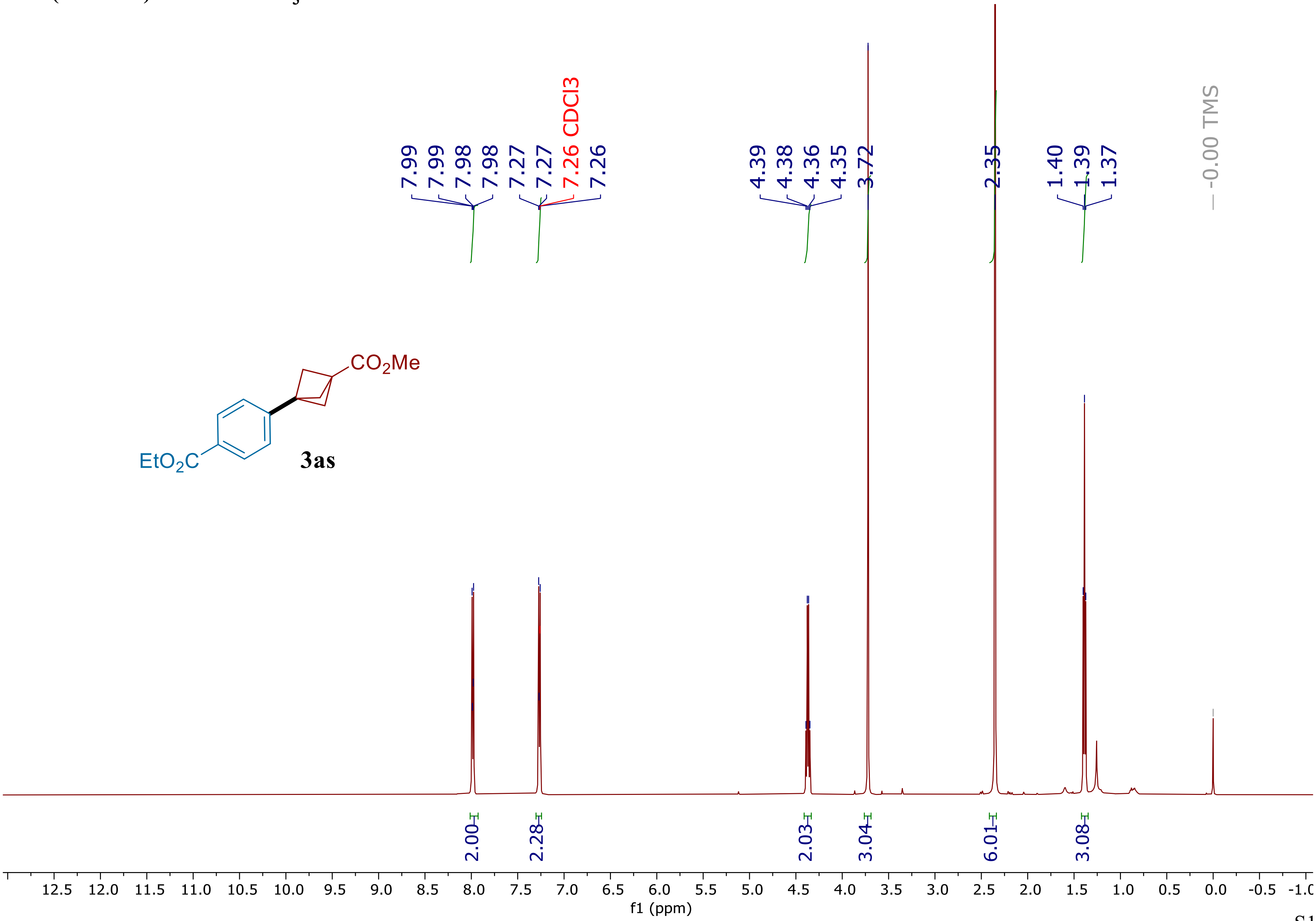

$^{13}C\{^1H\}$ NMR (126 MHz) of 3as in $CDCl_3$

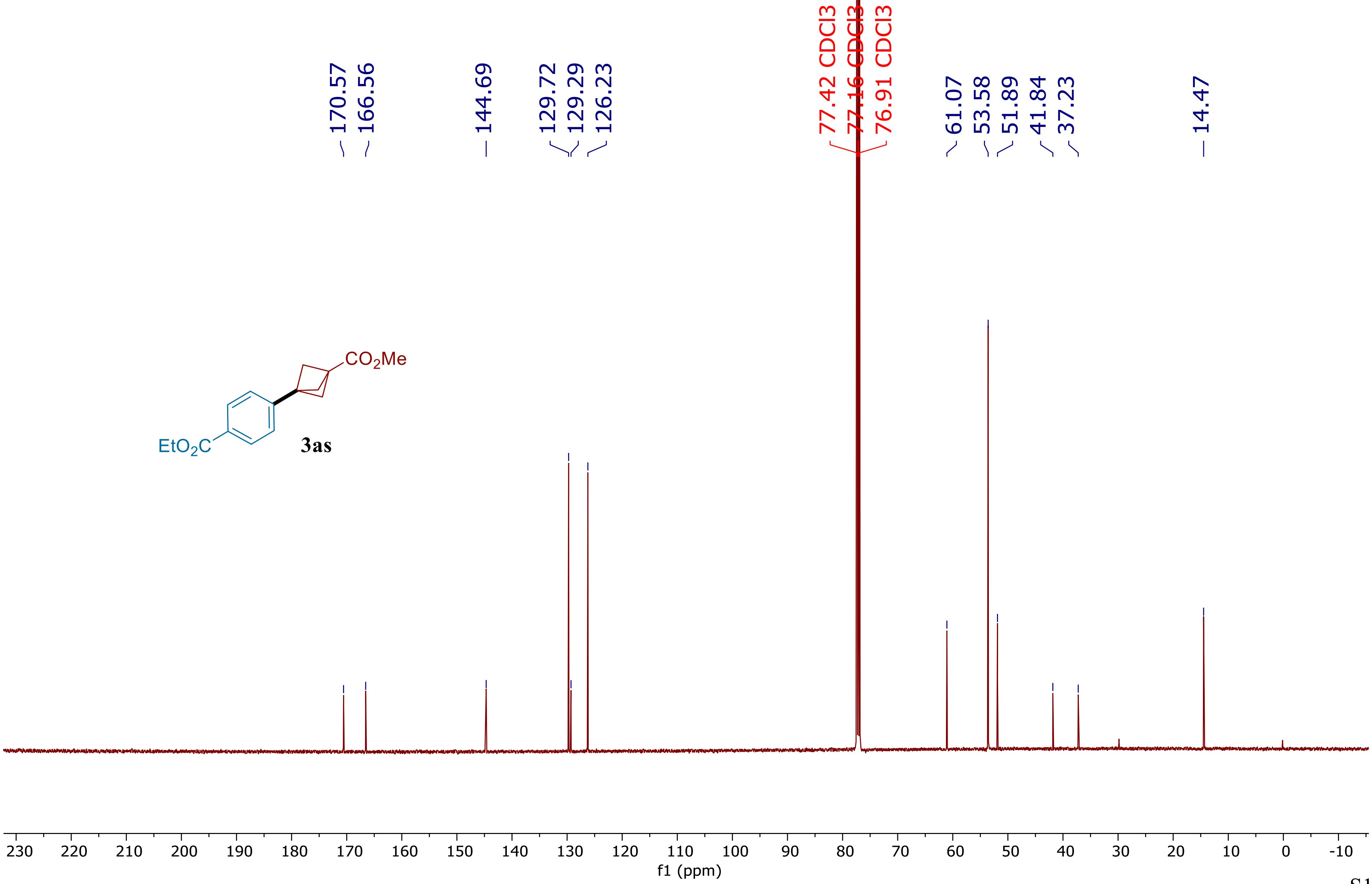

^{1}H NMR (500 MHz) of 3at in $CDCl_3$

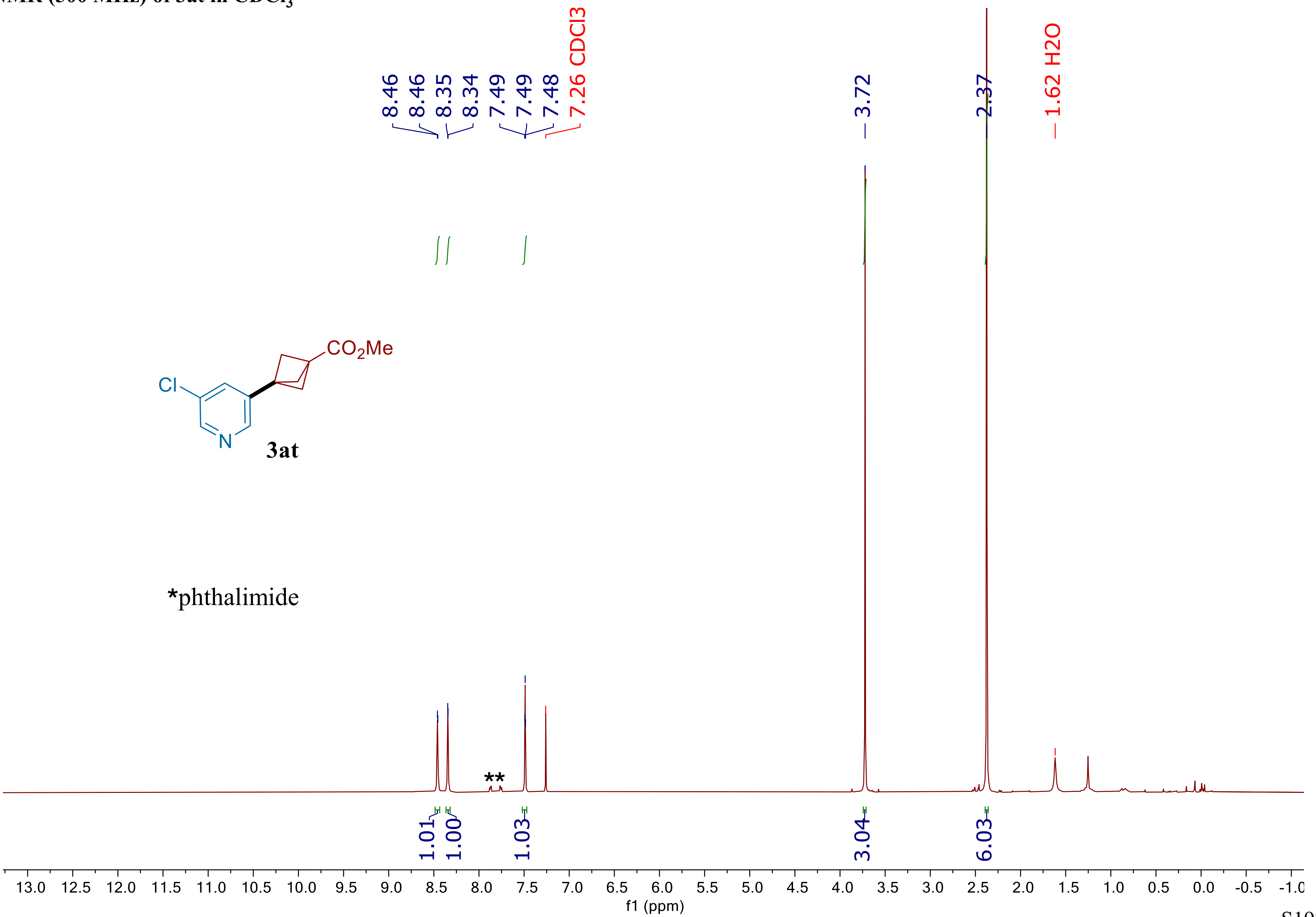

$^{13}C\{^{1}H\}$ NMR (126 MHz) of 3at in $CDCl_3$

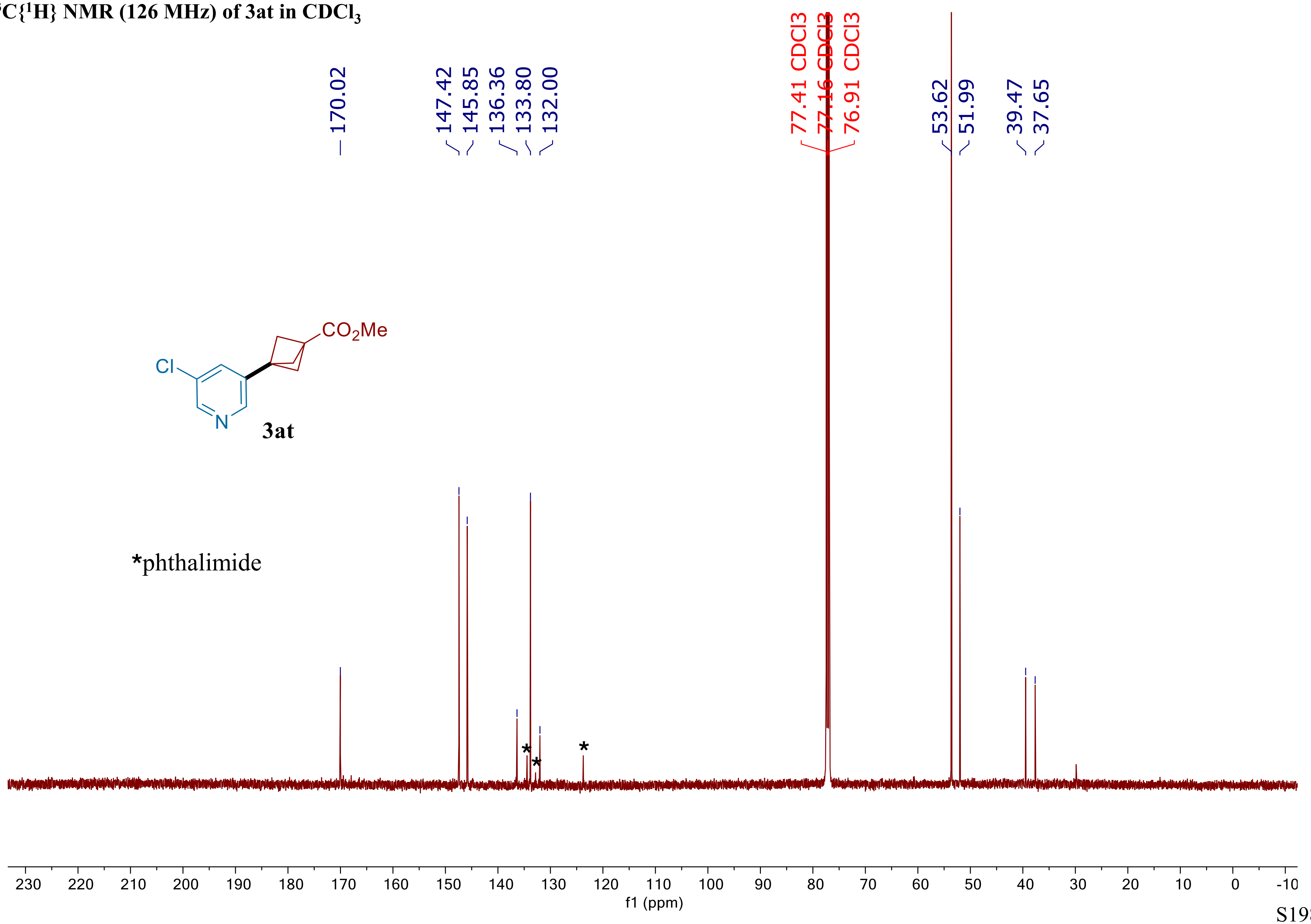

^1H NMR (500 MHz) of 3au in $CDCl_3$

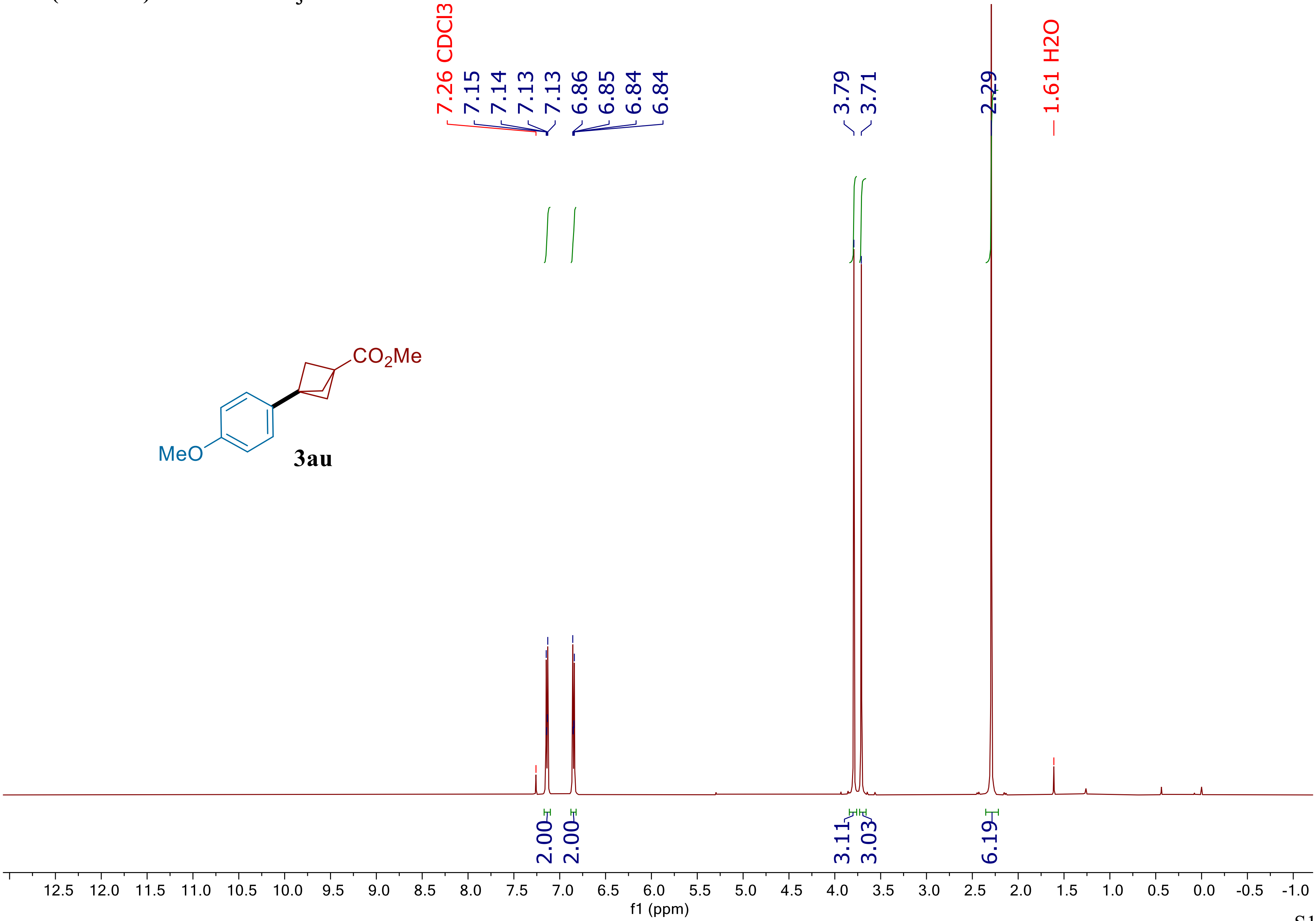

$^{13}C\{^{1}H\}$ NMR (126 MHz) of 3au in $CDCl_3$

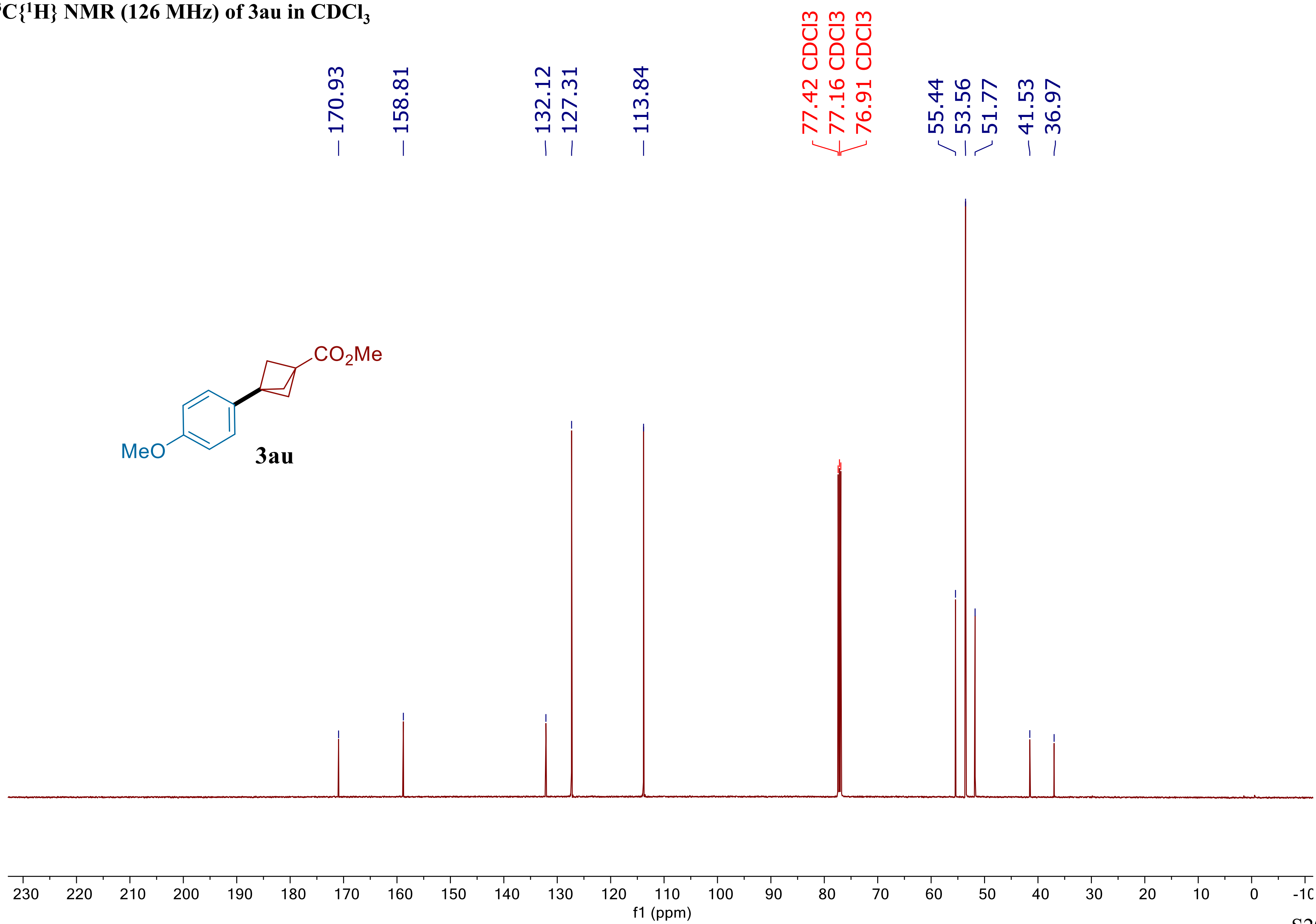

3au

^{1}H NMR (500 MHz) of 3av in $CDCl_3$

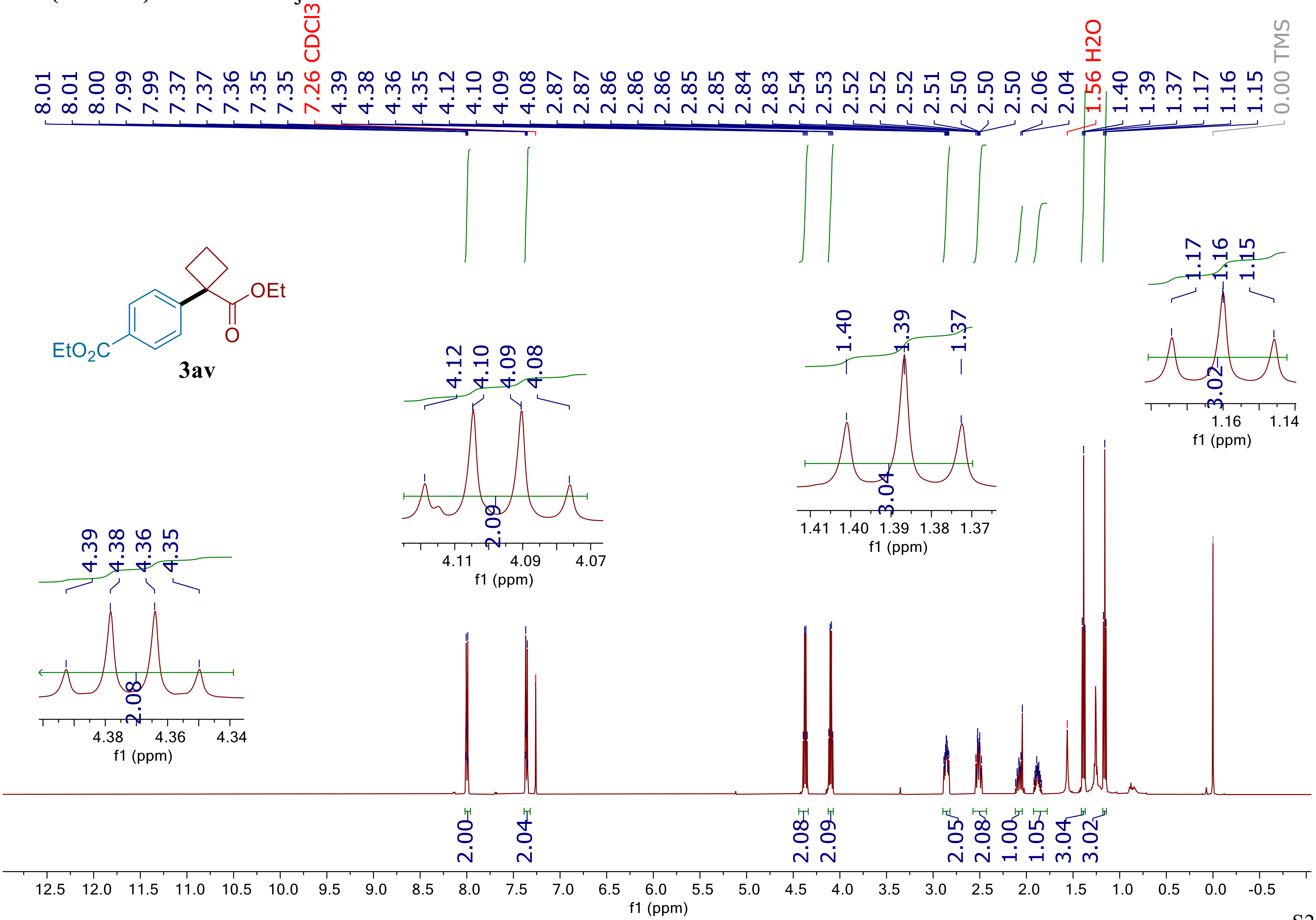

$^{13}C\{^1H\}$ NMR (126 MHz) of 3av in $CDCl_3$

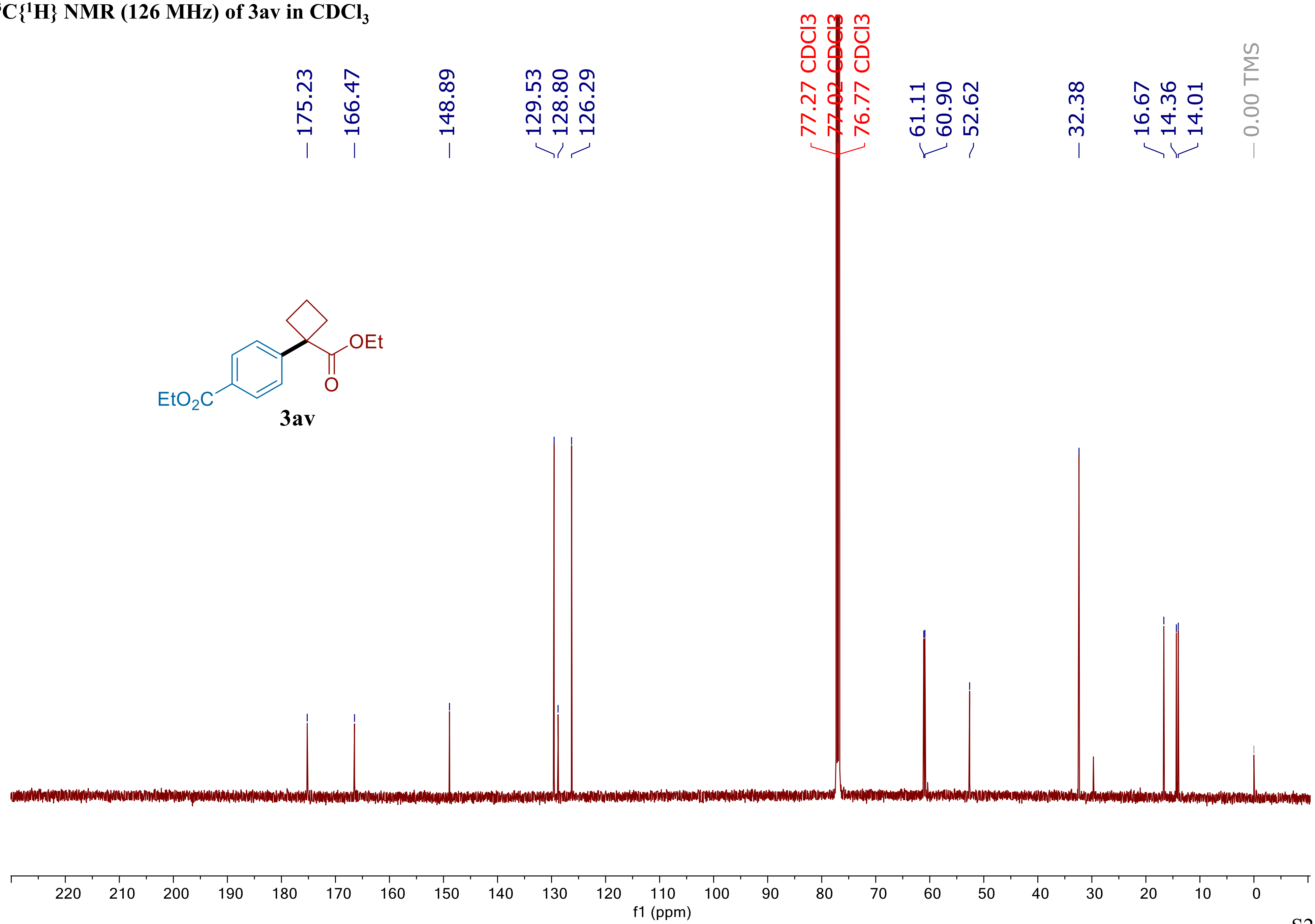

^{1}H NMR (500 MHz) of 3aw in $CDCl_3$

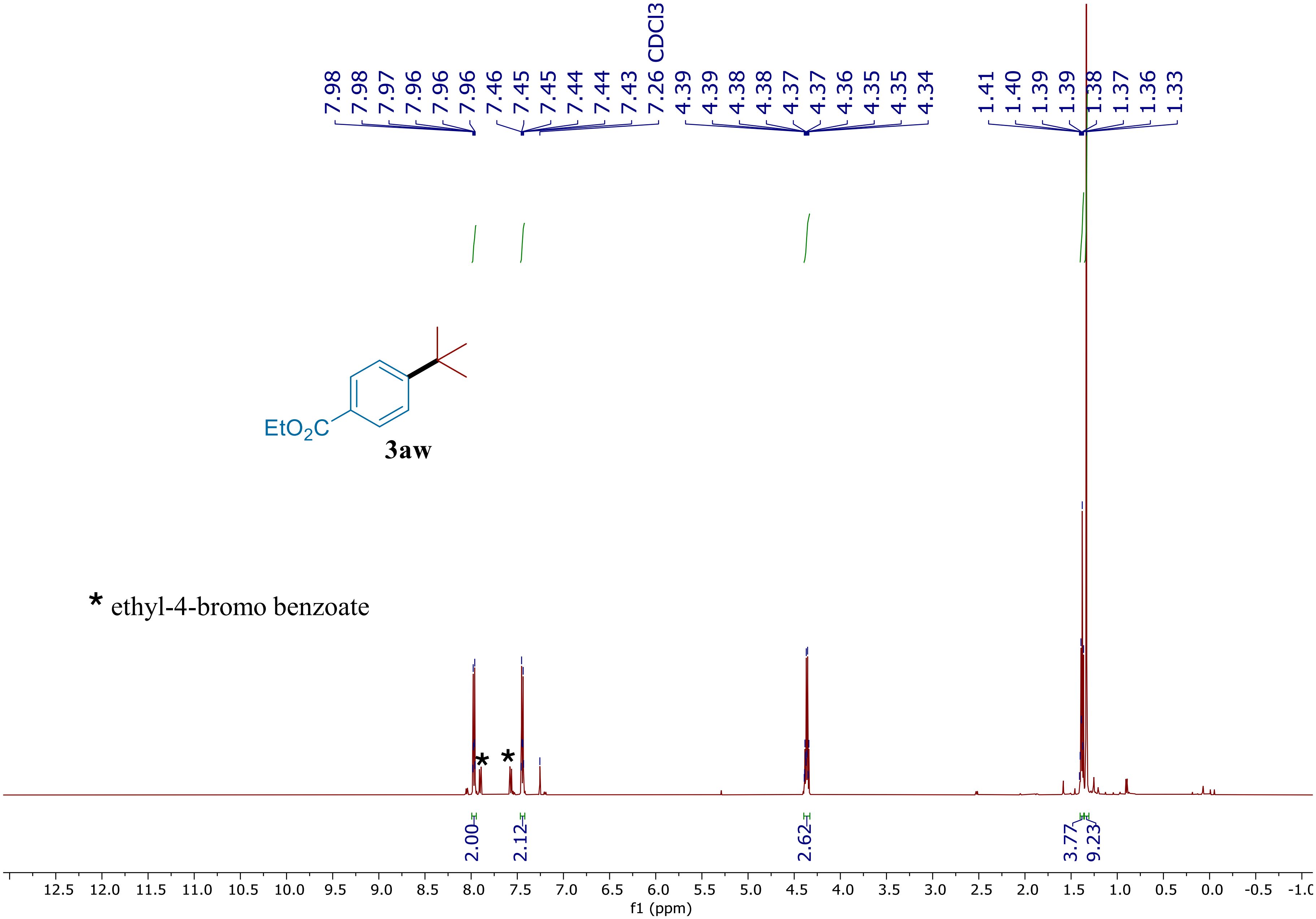

$^{13}C\{^{1}H\}$ NMR (126 MHz) of 3aw in $CDCl_3$

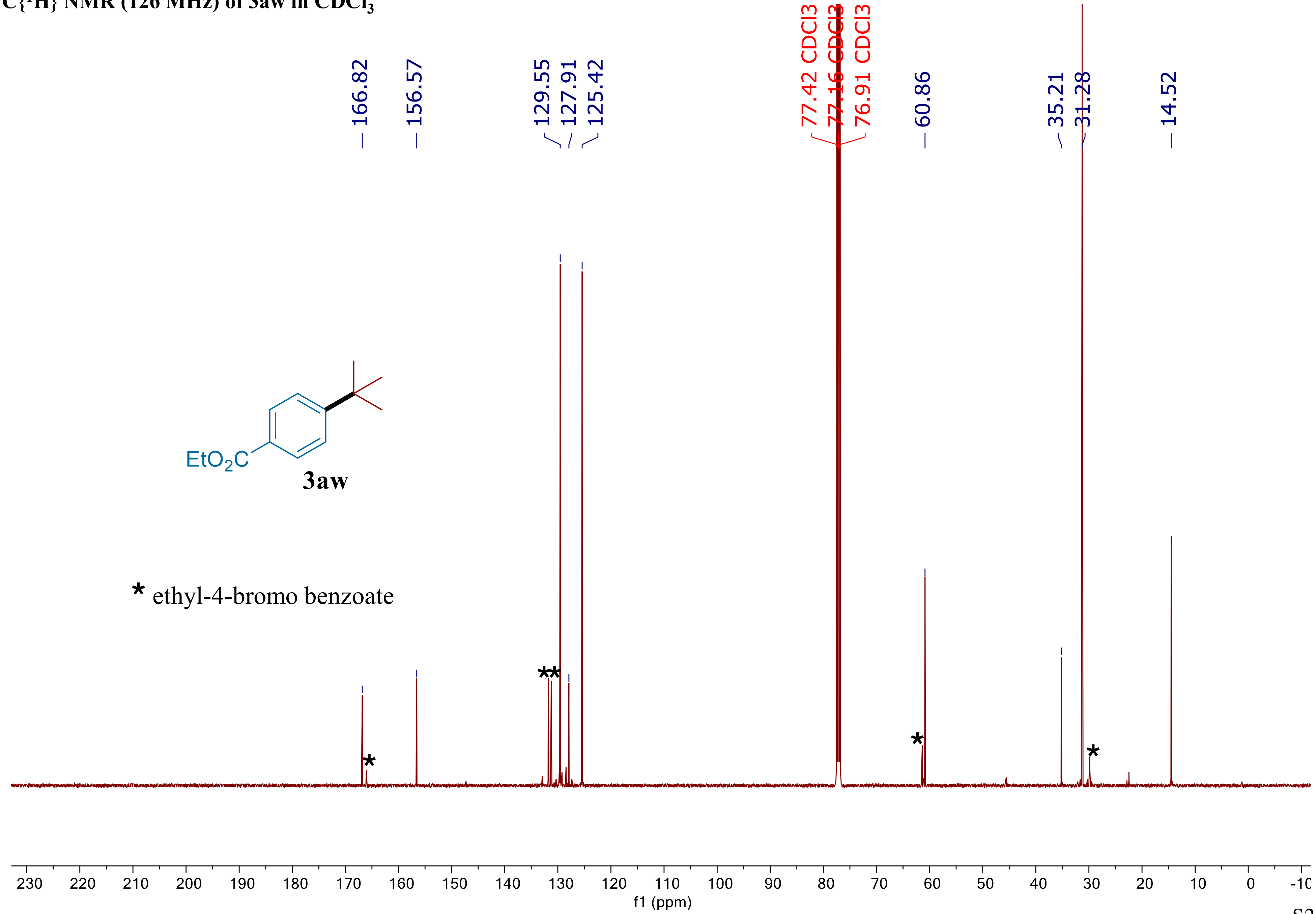

^{1}H NMR (500 MHz) of 3ax in $CDCl_3$

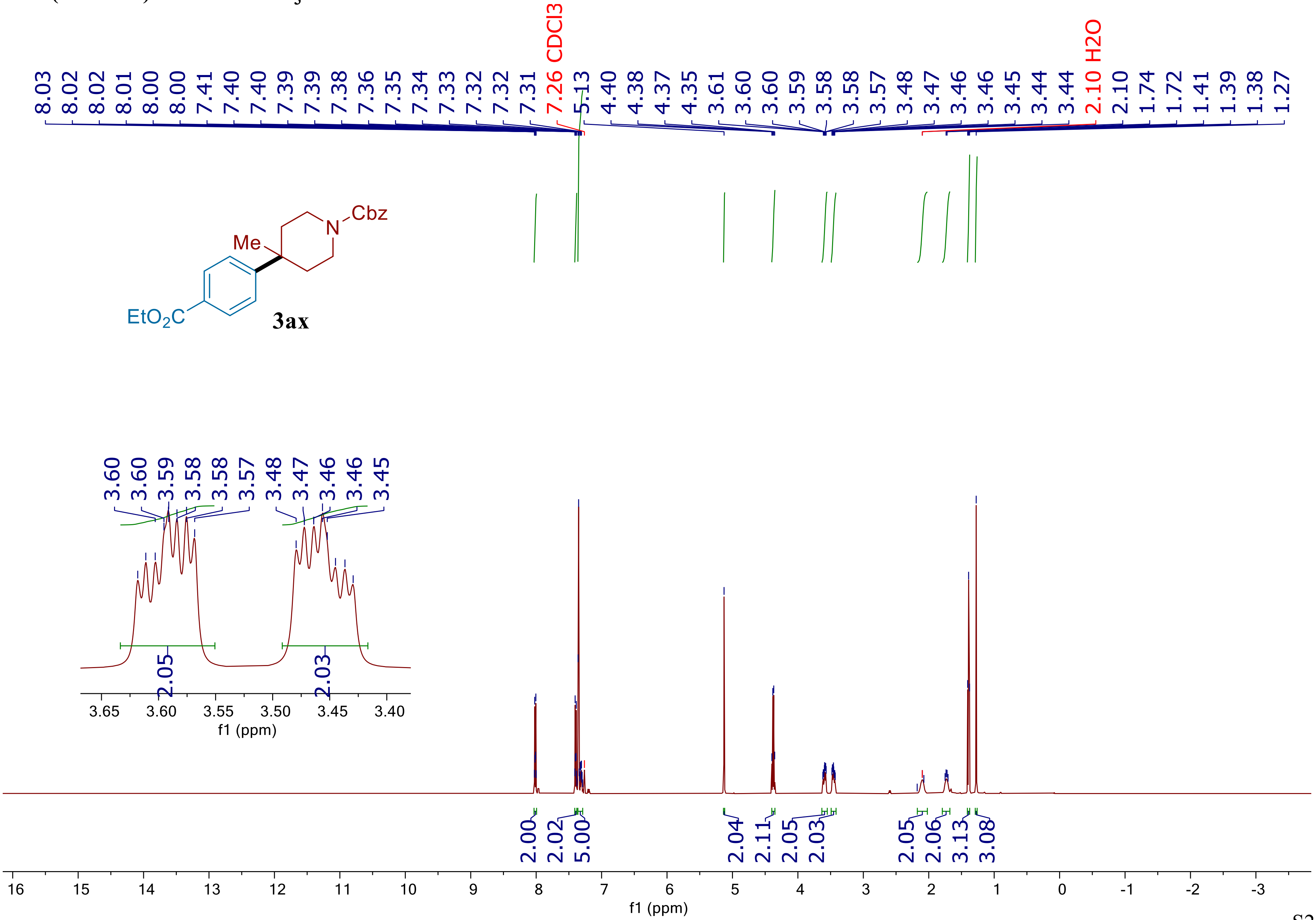

$^{13}C\{^{1}H\}$ NMR (126 MHz) of 3ax in $CDCl_3$

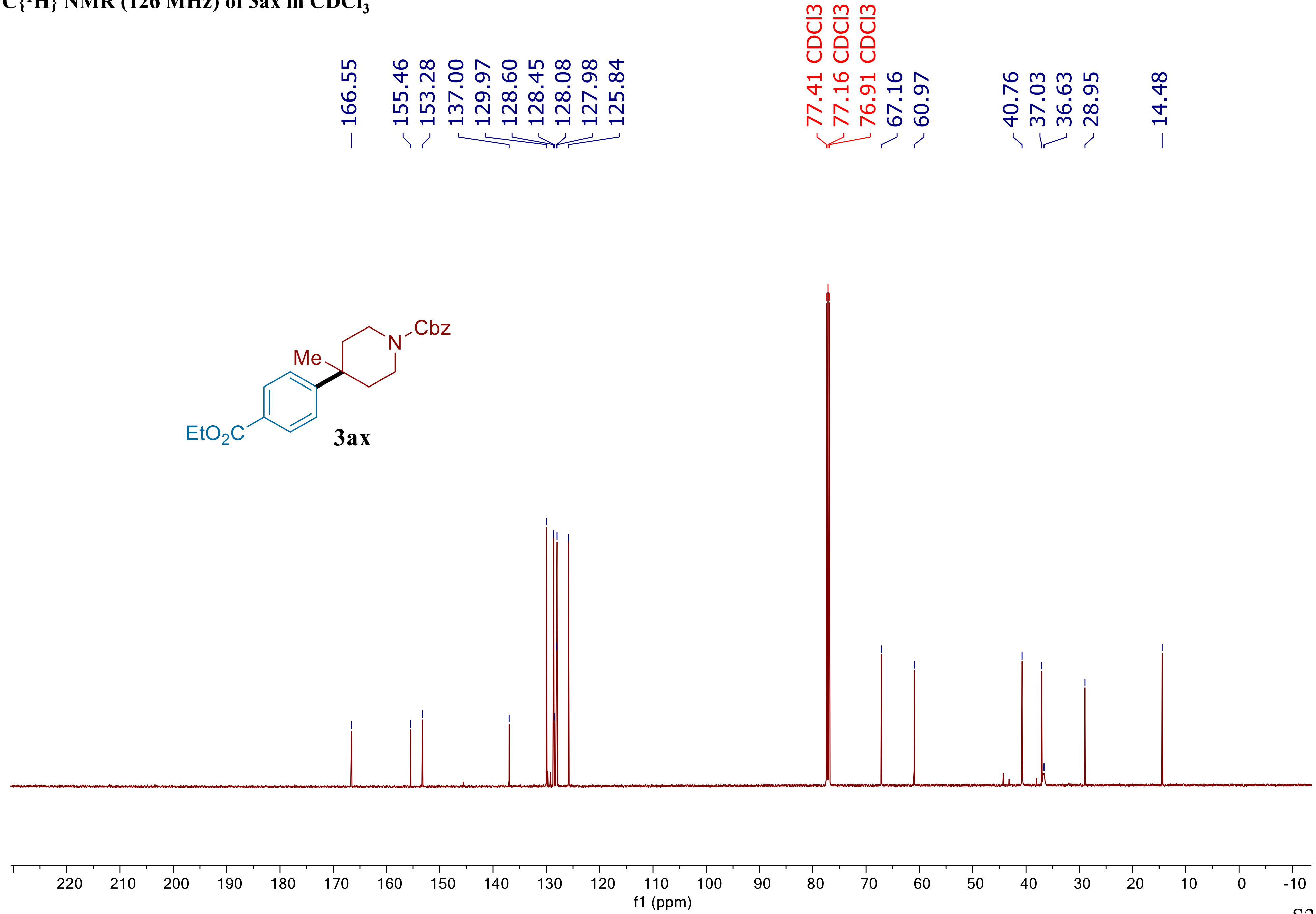

^{1}H NMR (500 MHz) of 3ay in $CDCl_3$

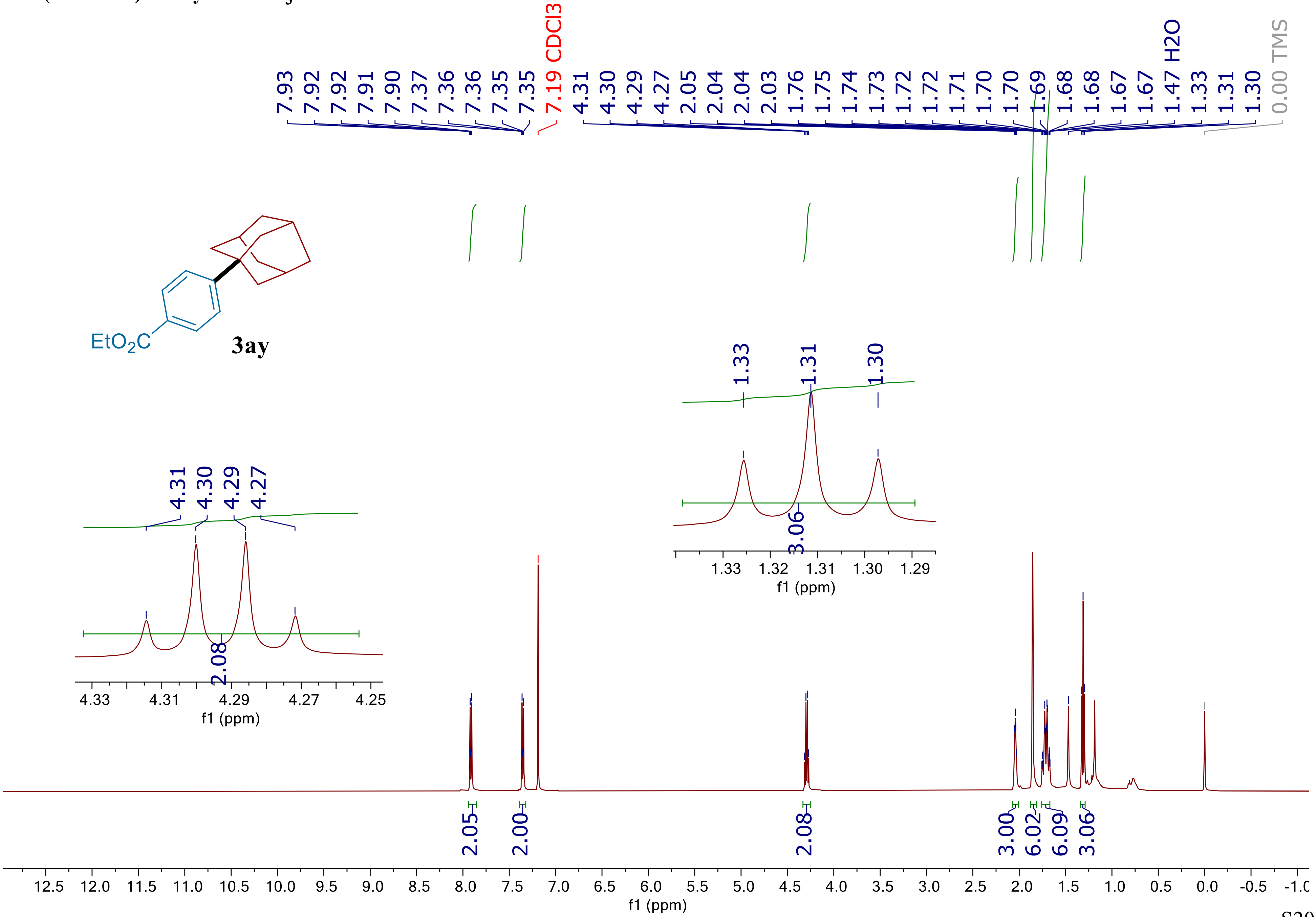

$^{13}C\{^{1}H\}$ NMR (126 MHz) of 3ay in $CDCl_3$

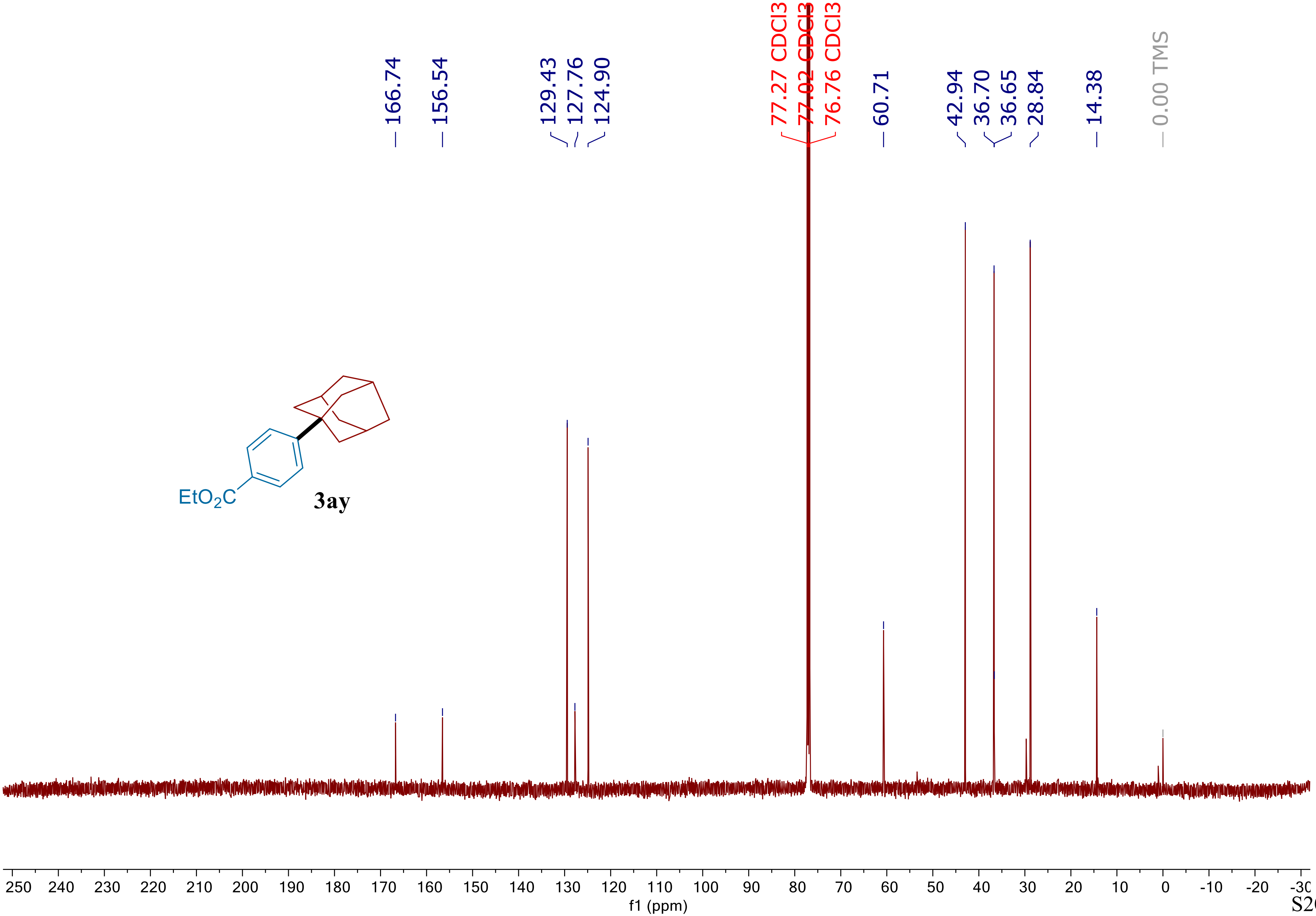

^{1}H NMR (600 MHz) of 3az in DMSO-D$_6$

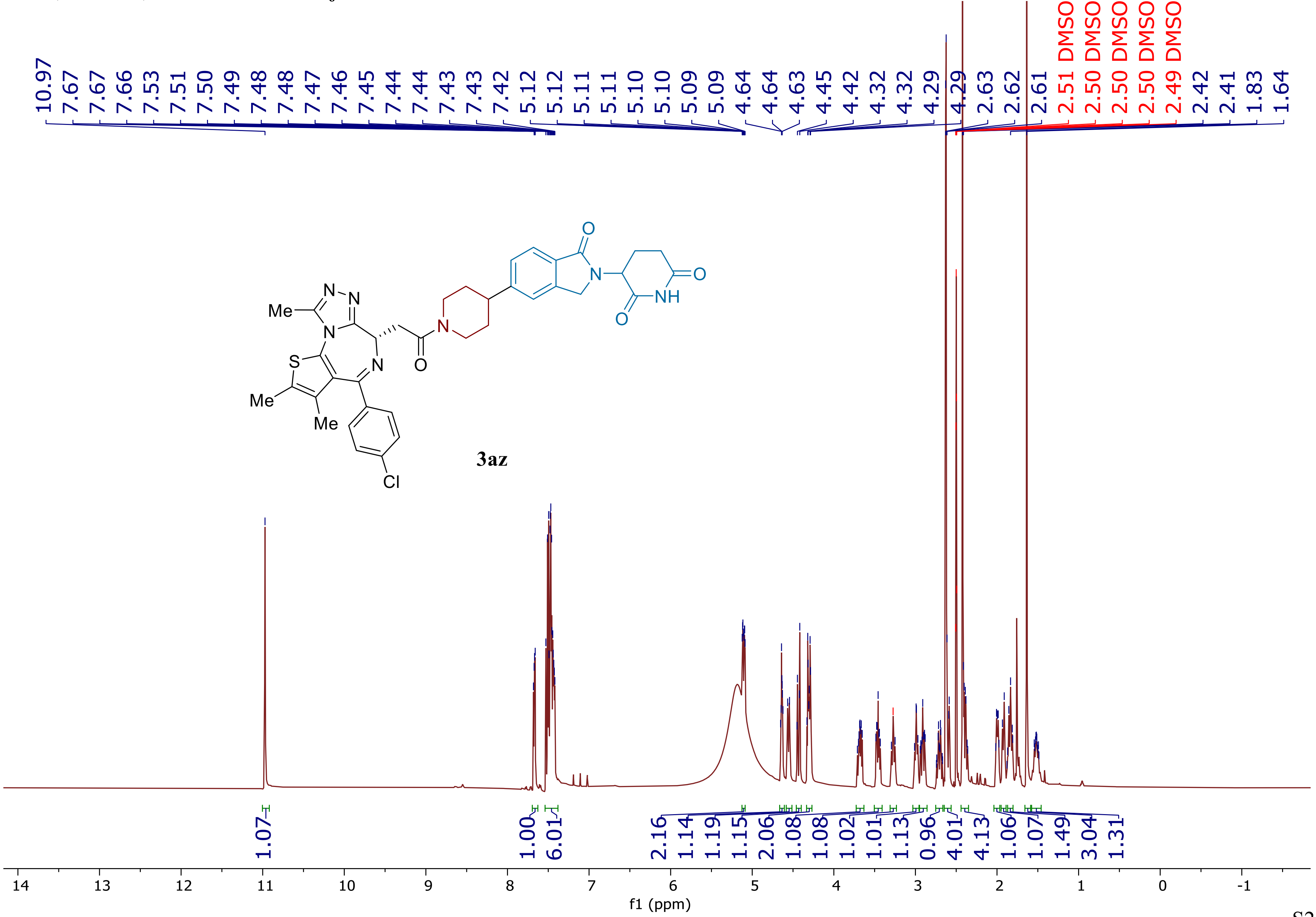

^{13}C-APT NMR (150 MHz) of 3az in DMSO-D$_6$

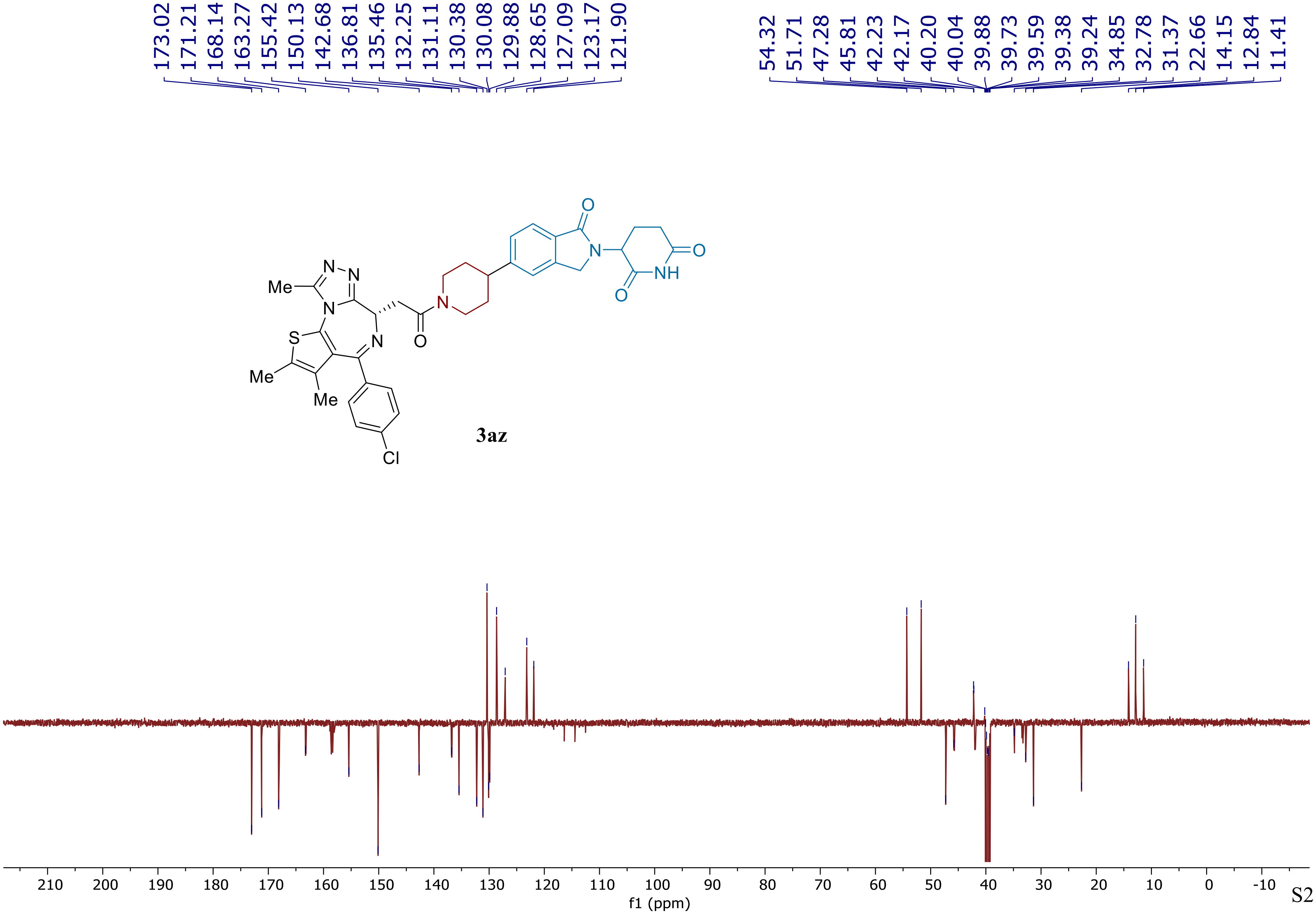

^{1}H NMR (500 MHz) of 4a in $CDCl_3$

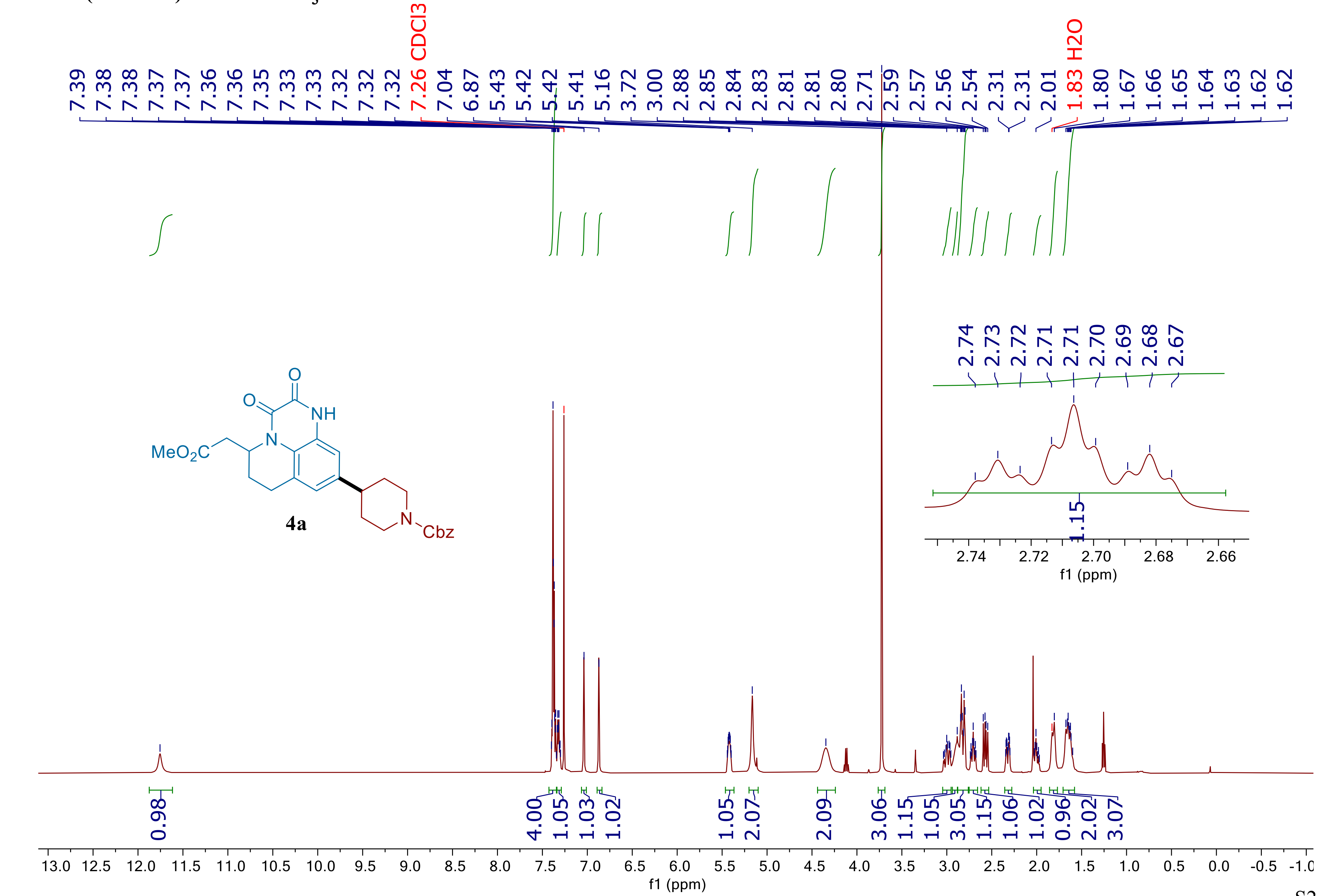

$^{13}C\{^{1}H\}$ NMR (126 MHz) of 4a in $CDCl_3$

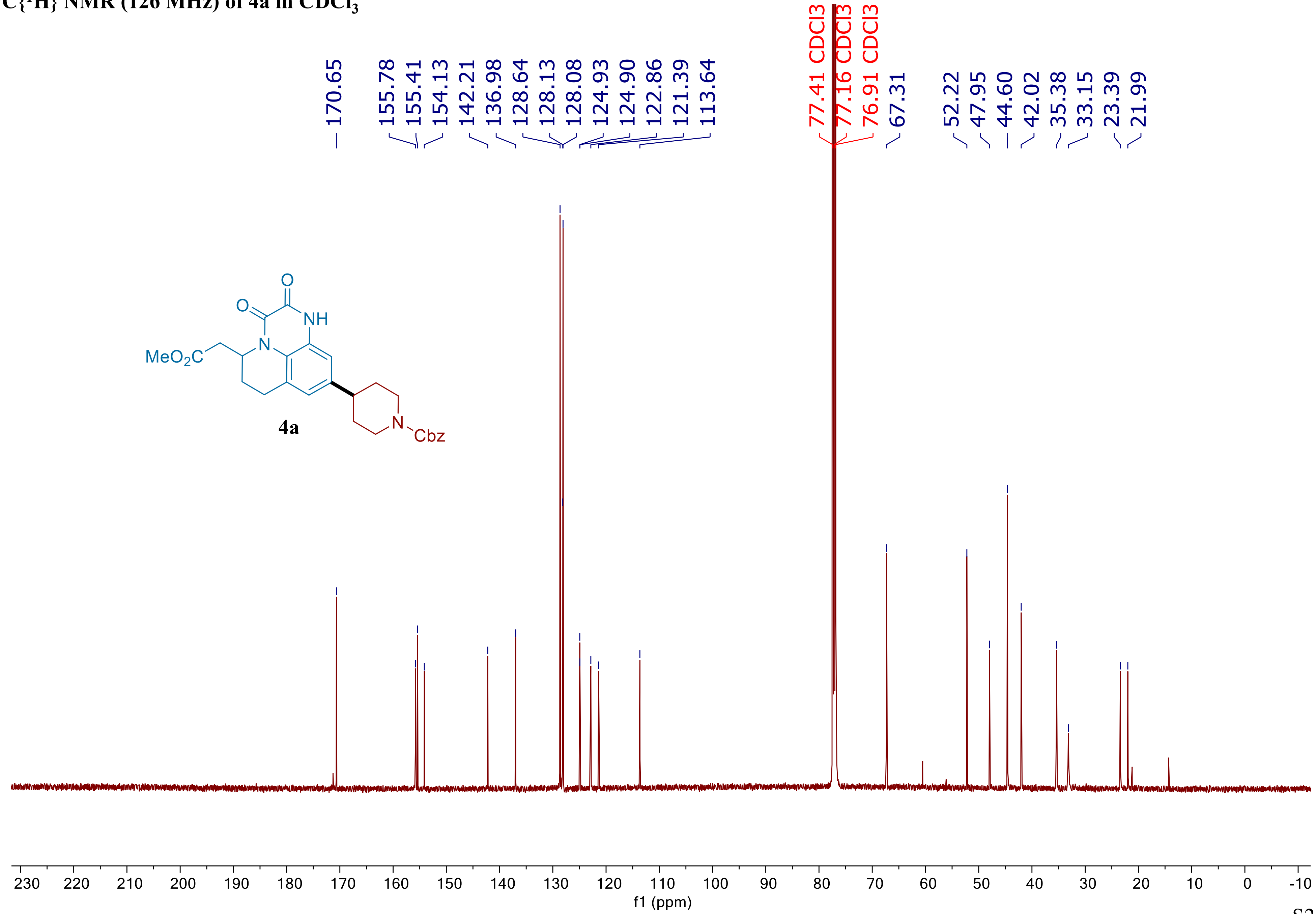

^{1}H NMR (500 MHz) of 4b in $CDCl_3$

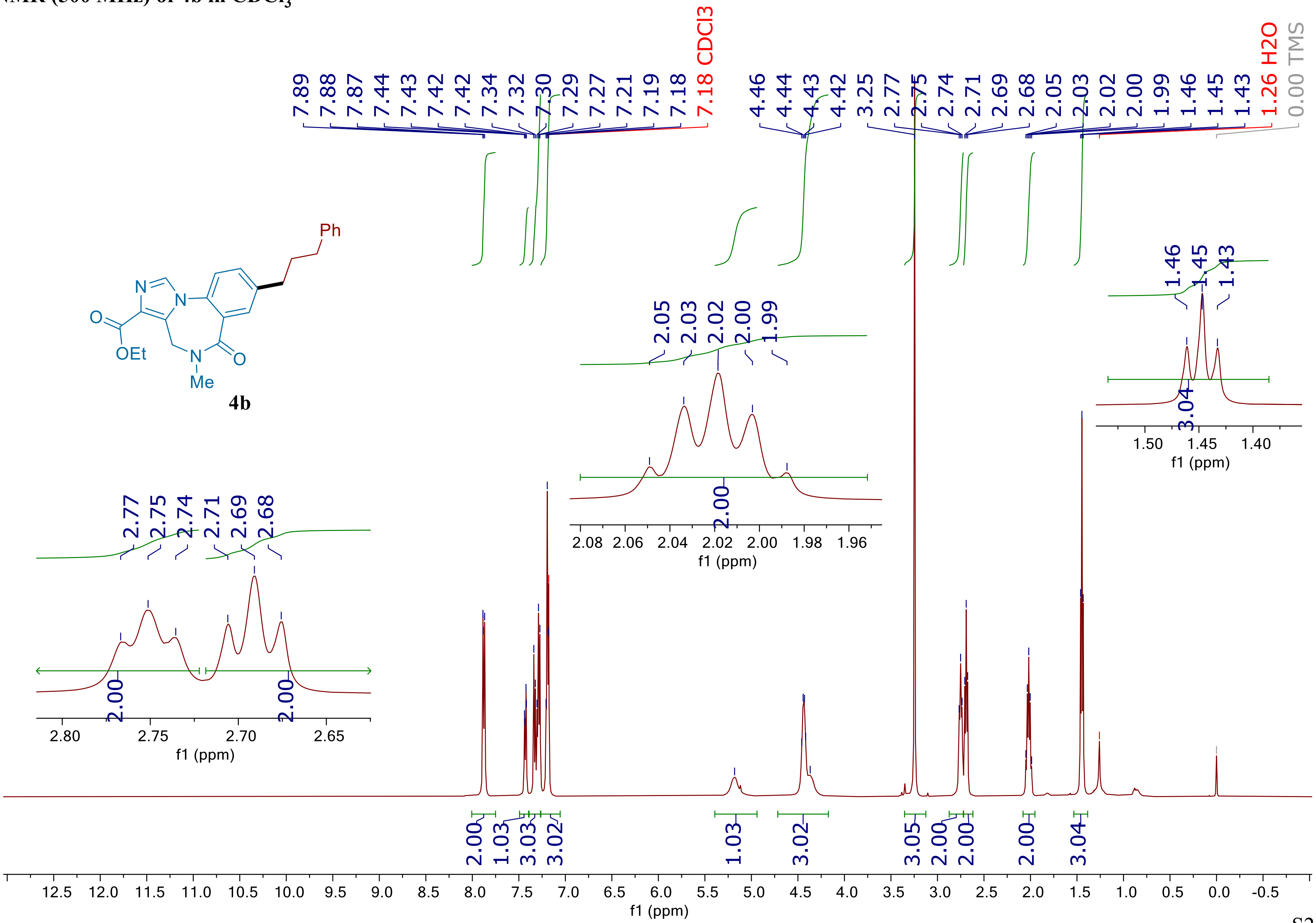

$^{13}C\{^{1}H\}$ NMR (126 MHz) of 4b in $CDCl_3$

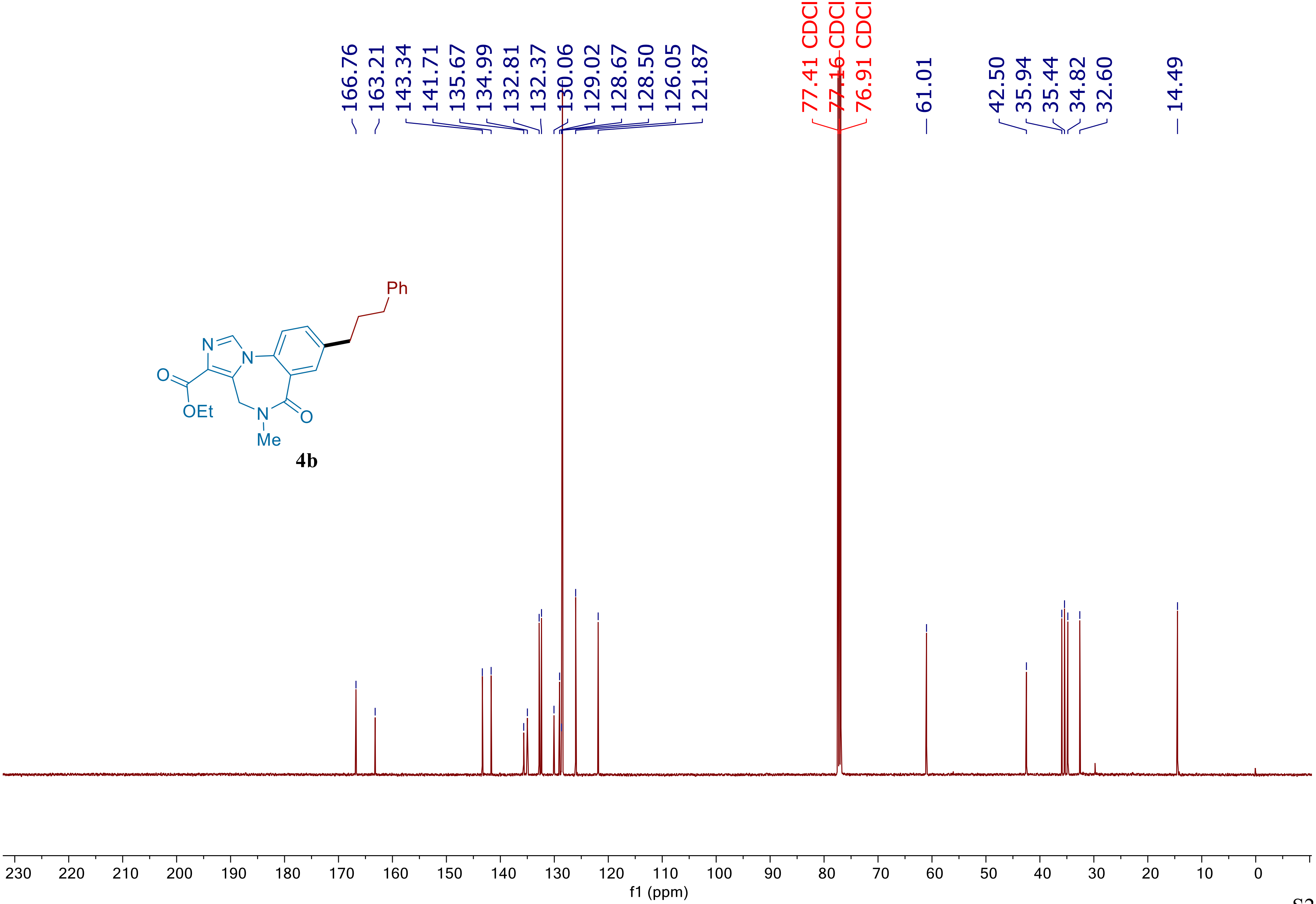

^{1}H NMR (500 MHz) of 4c in CDCl$_3$

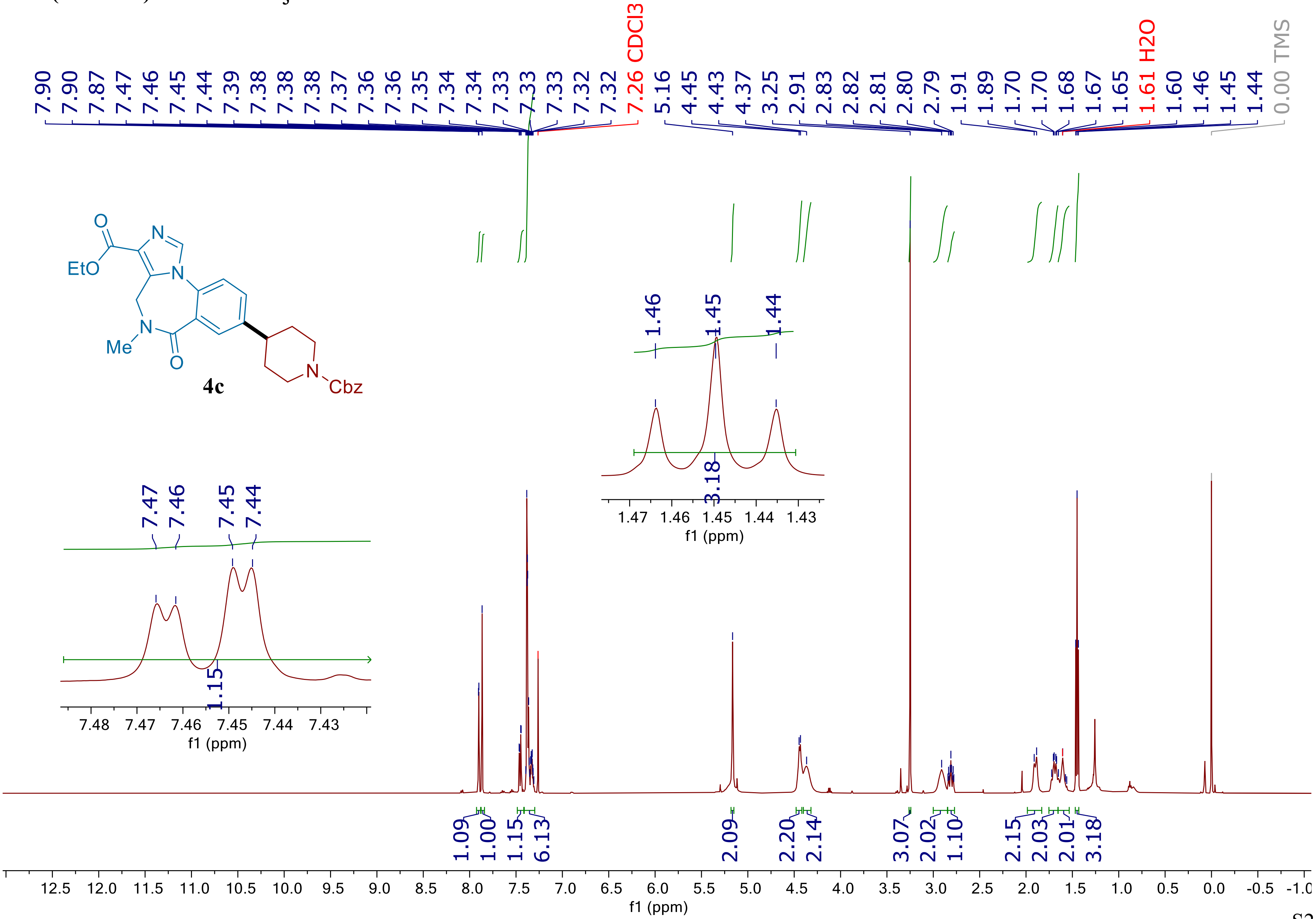

$^{13}C\{^{1}H\}$ NMR (126 MHz) of 4c in $CDCl_3$

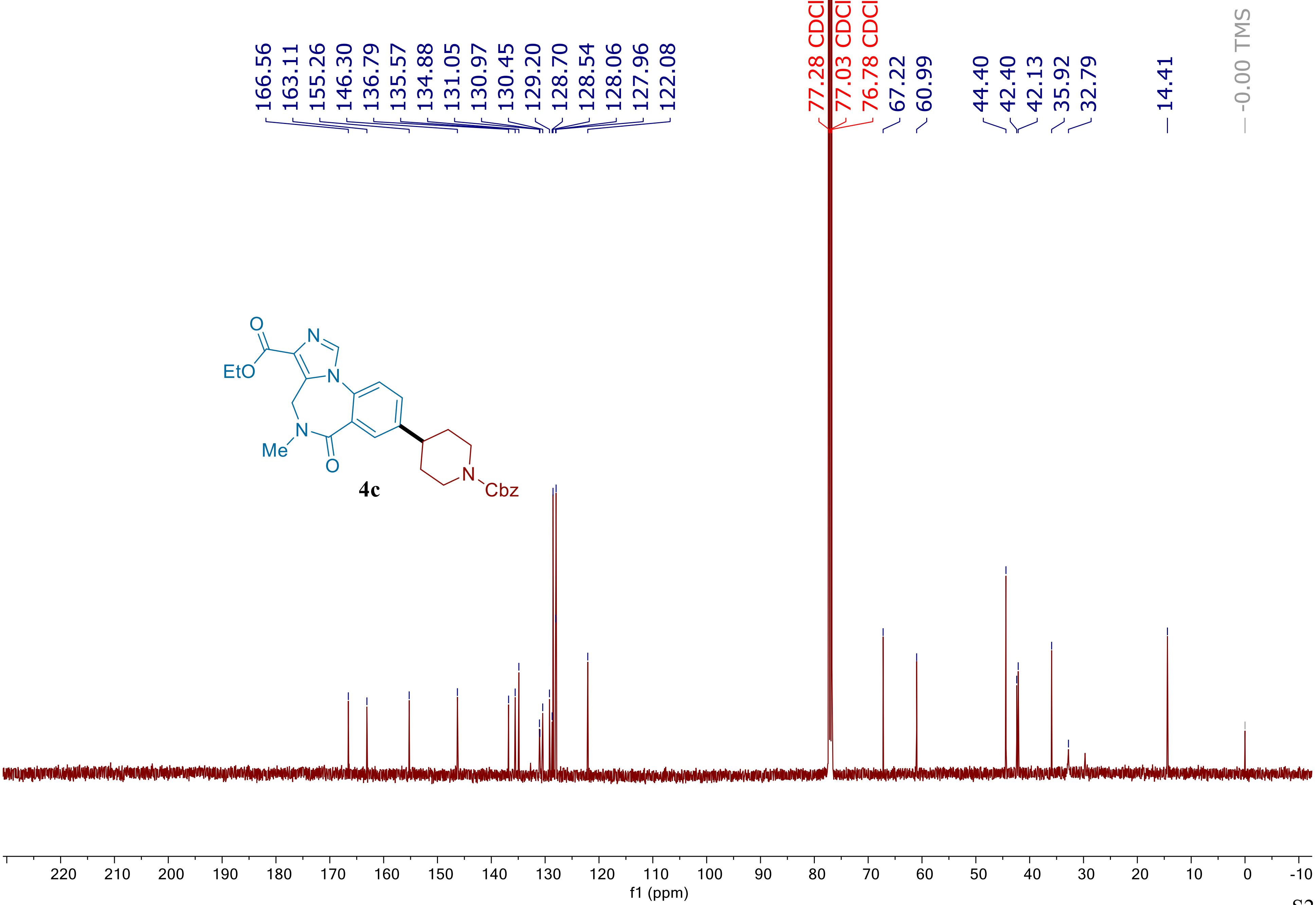

^{1}H NMR (500 MHz) of 4d in $CDCl_3$

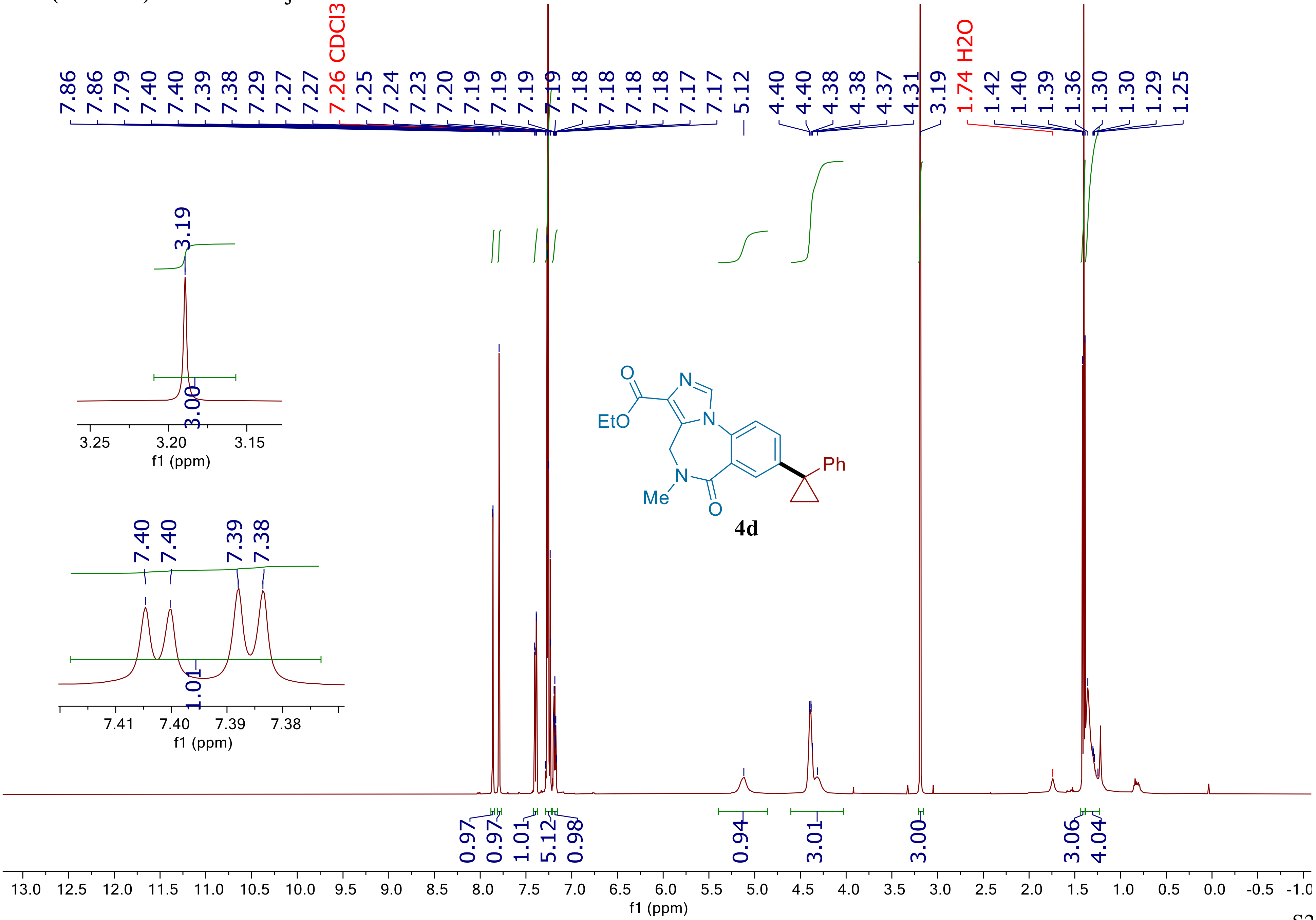

$^{13}C\{^{1}H\}$ NMR (126 MHz) of 4d in $CDCl_3$

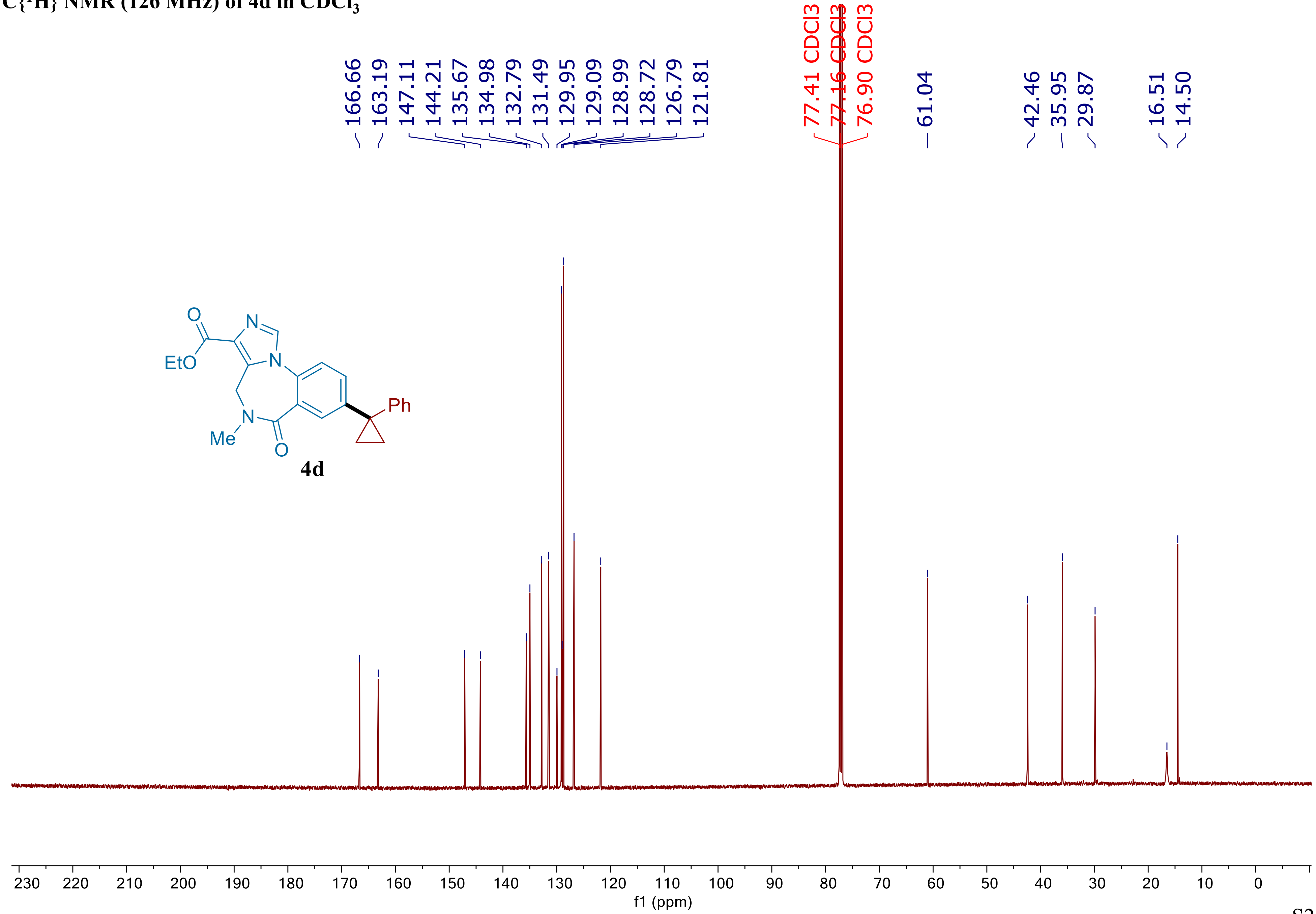

4d

^{1}H NMR (400 MHz) of 4e in $CDCl_3$

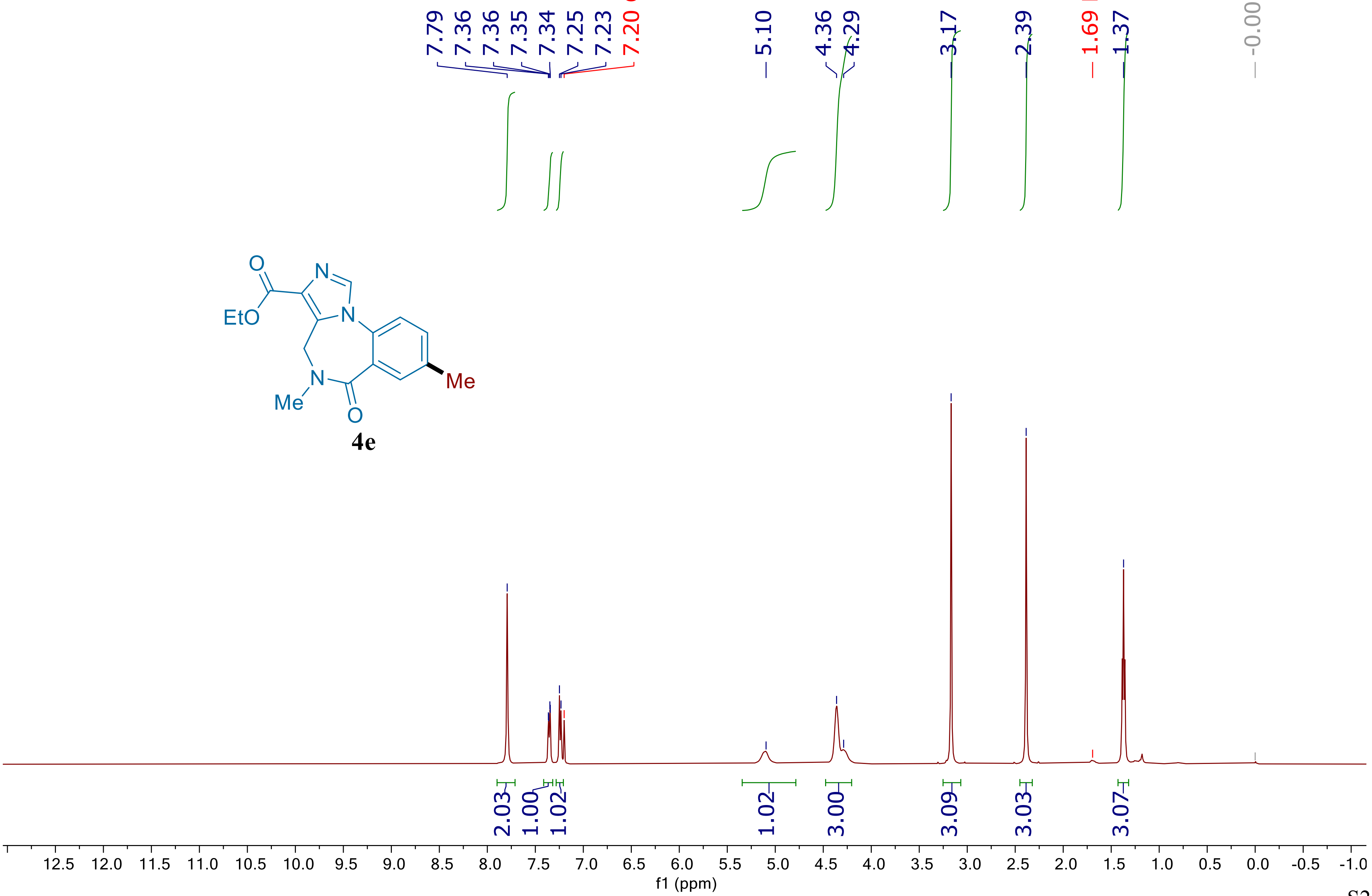

4e

$^{13}C\{^{1}H\}$ NMR (101 MHz) of 4e in $CDCl_3$

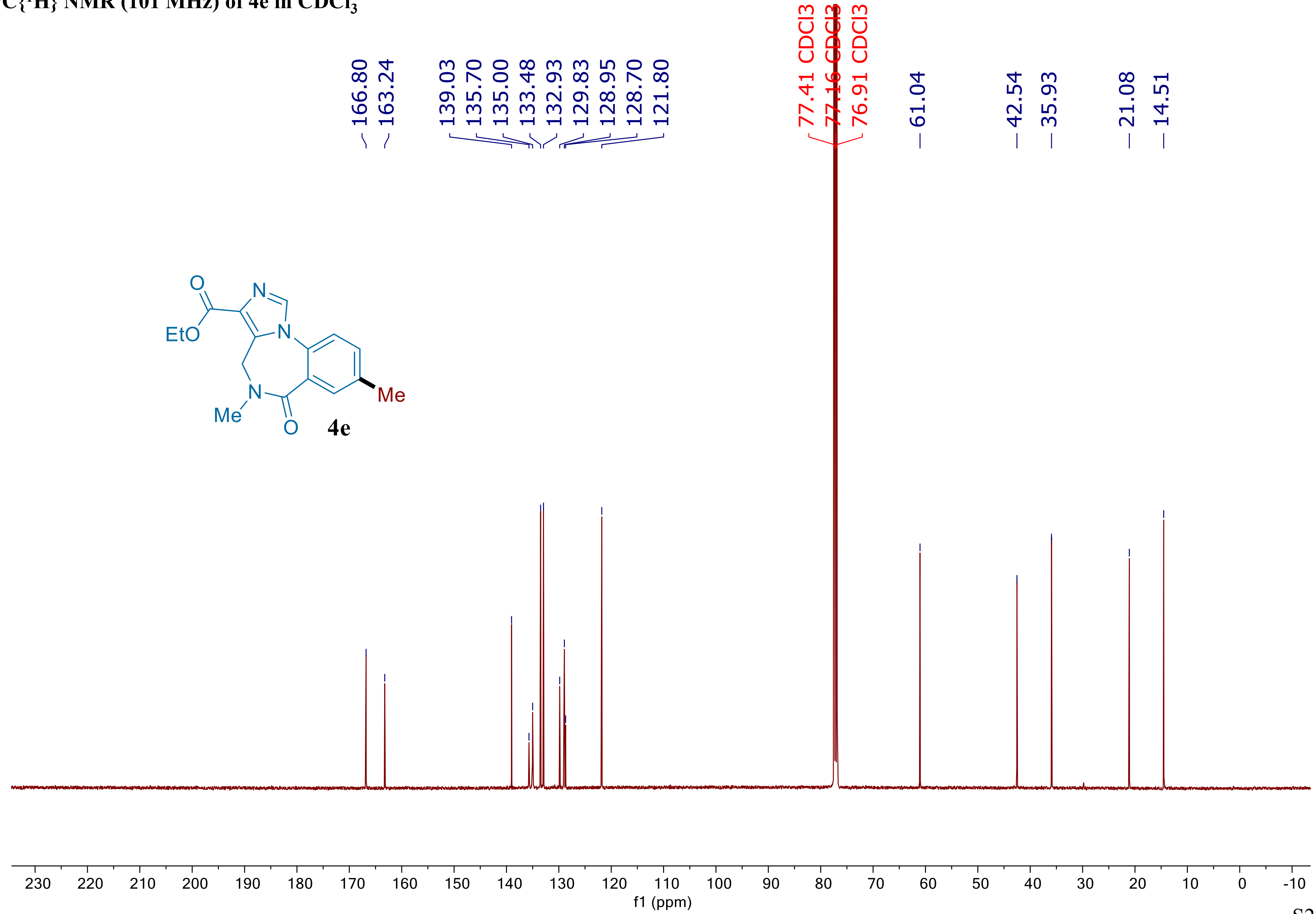

^{1}H NMR (500 MHz) of 4f in $CDCl_3$

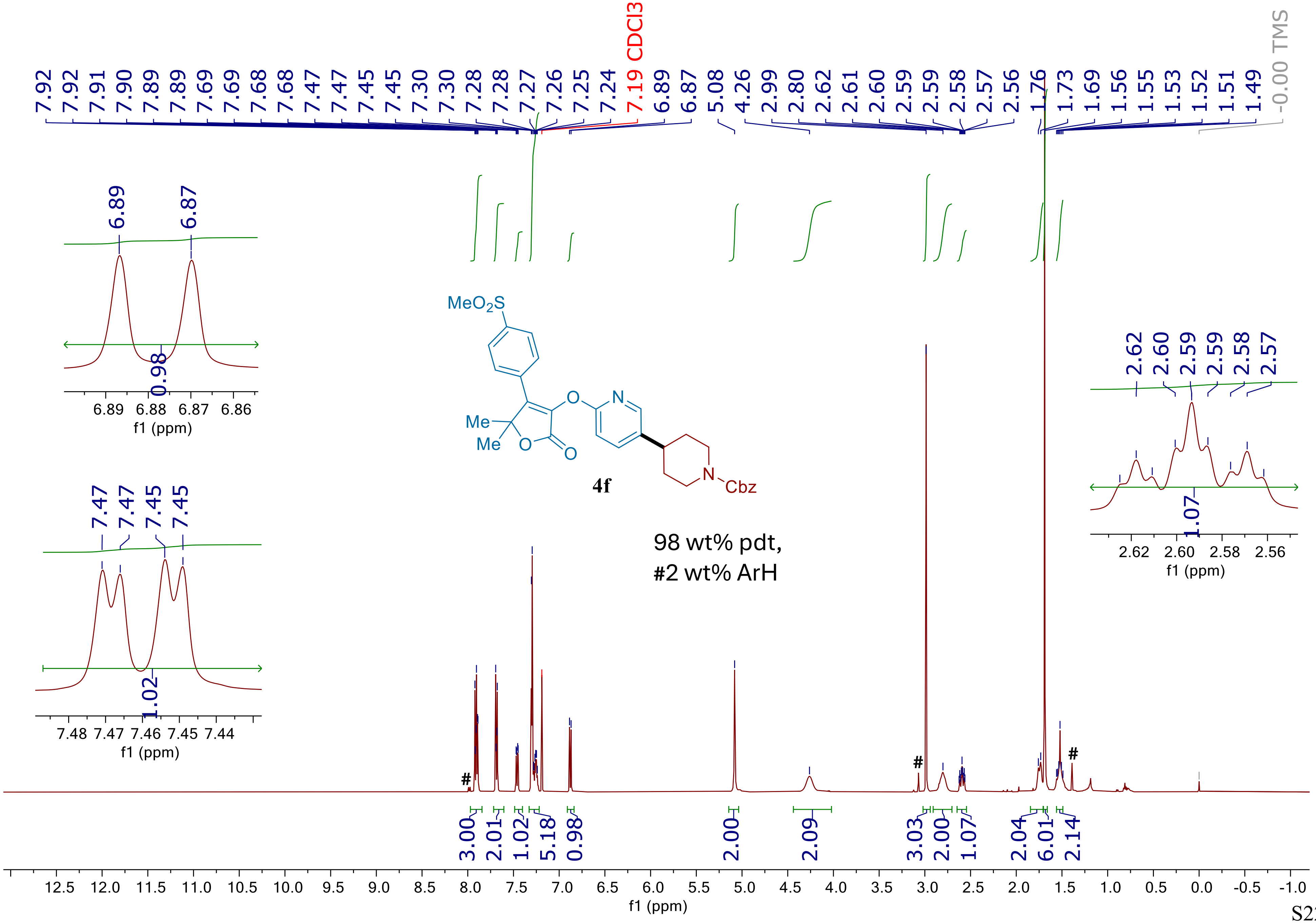

$^{13}C\{^{1}H\}$ NMR (126 MHz) of 4f in $CDCl_3$

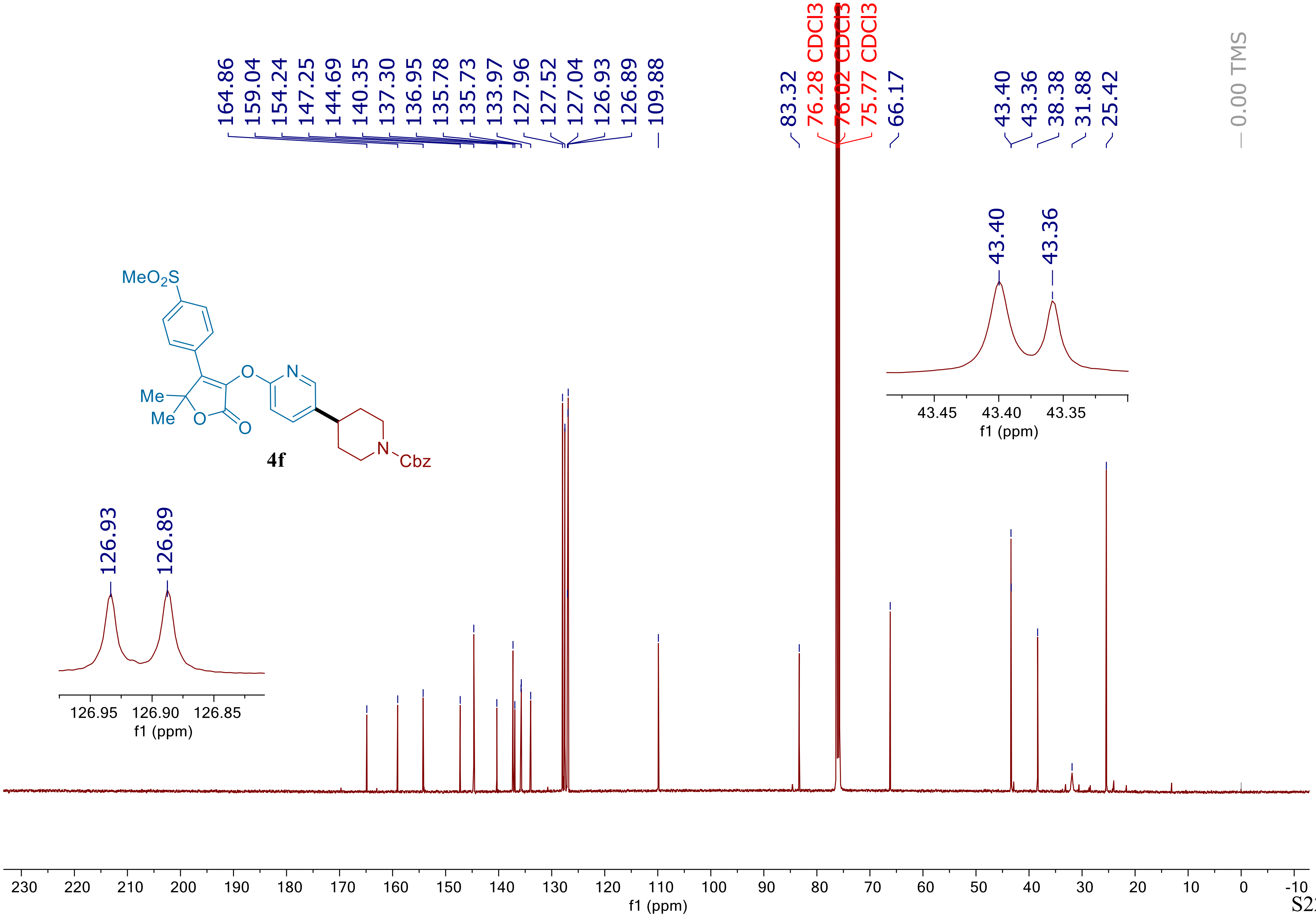

4f

Crude ^{1}H NMR (500 MHz) of 4g (unpurified) in $CDCl_3$

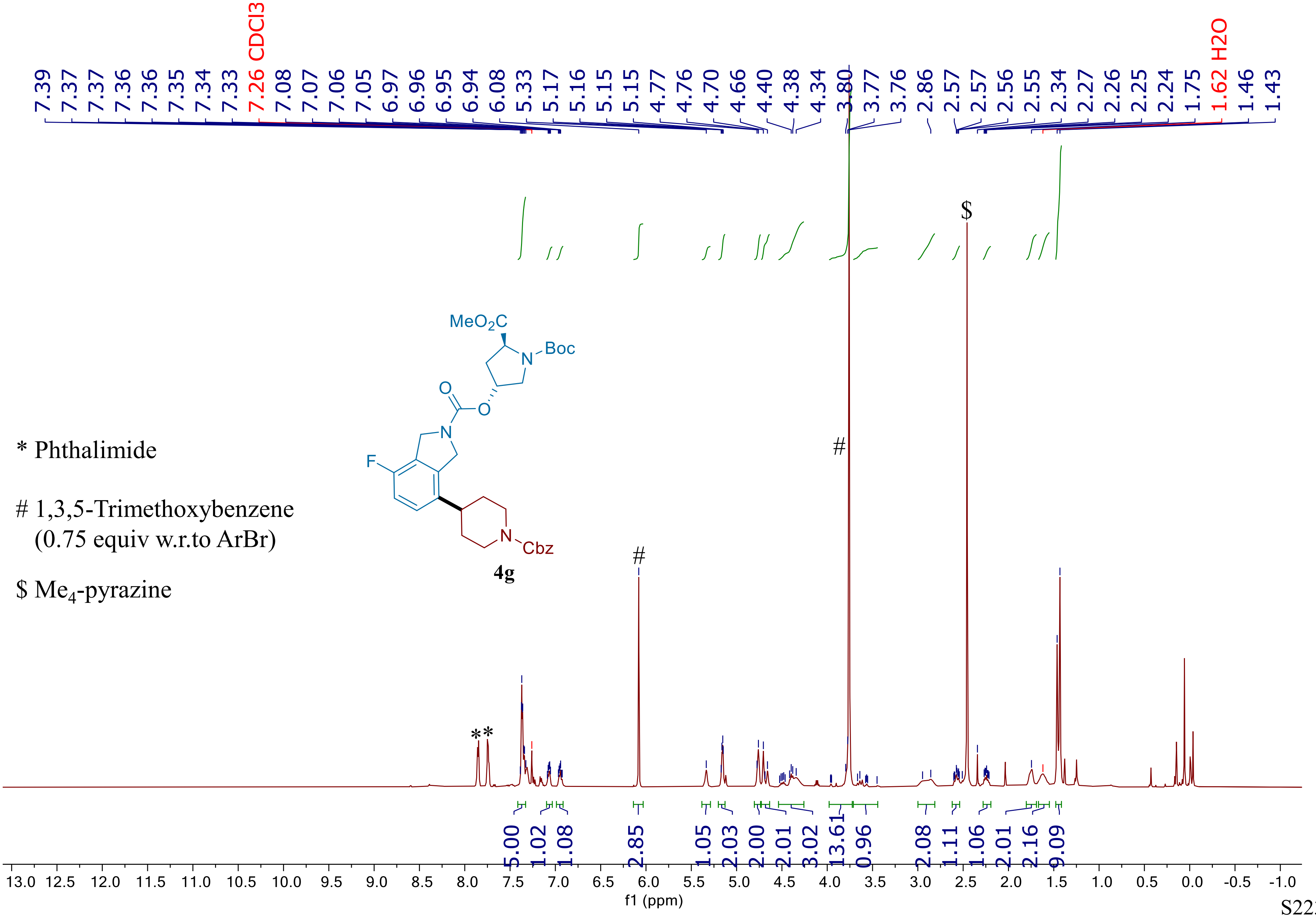

Crude $^{13}C\{^{1}H\}$ NMR (126 MHz) of 4g (unpurified) in $CDCl_3$

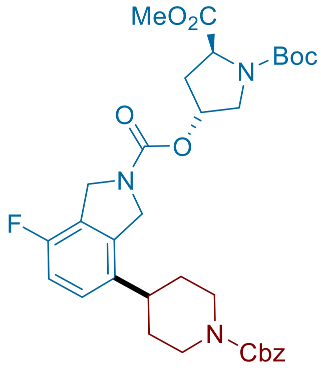

4g

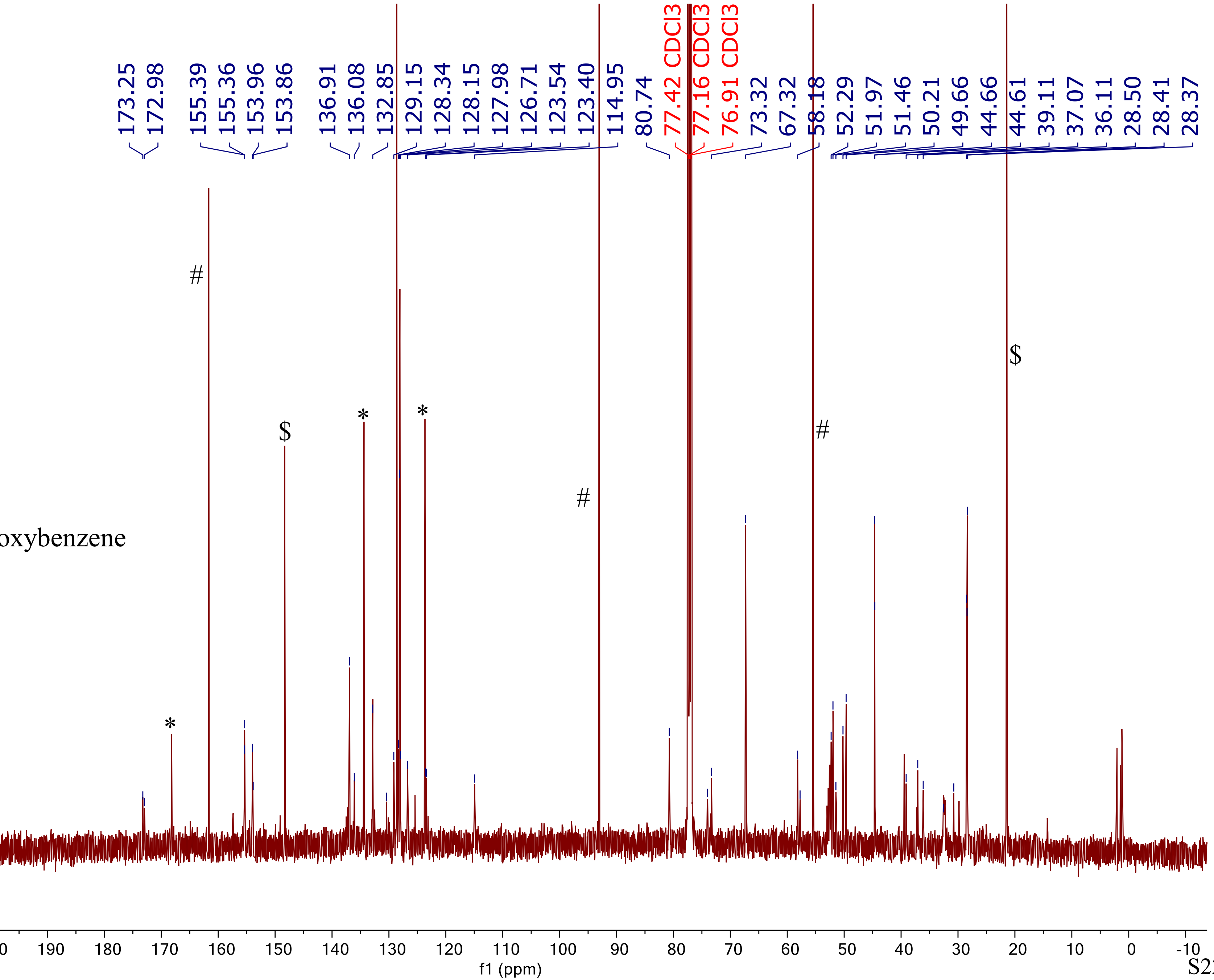

^{1}H NMR (500 MHz) of 4h in $CDCl_3$

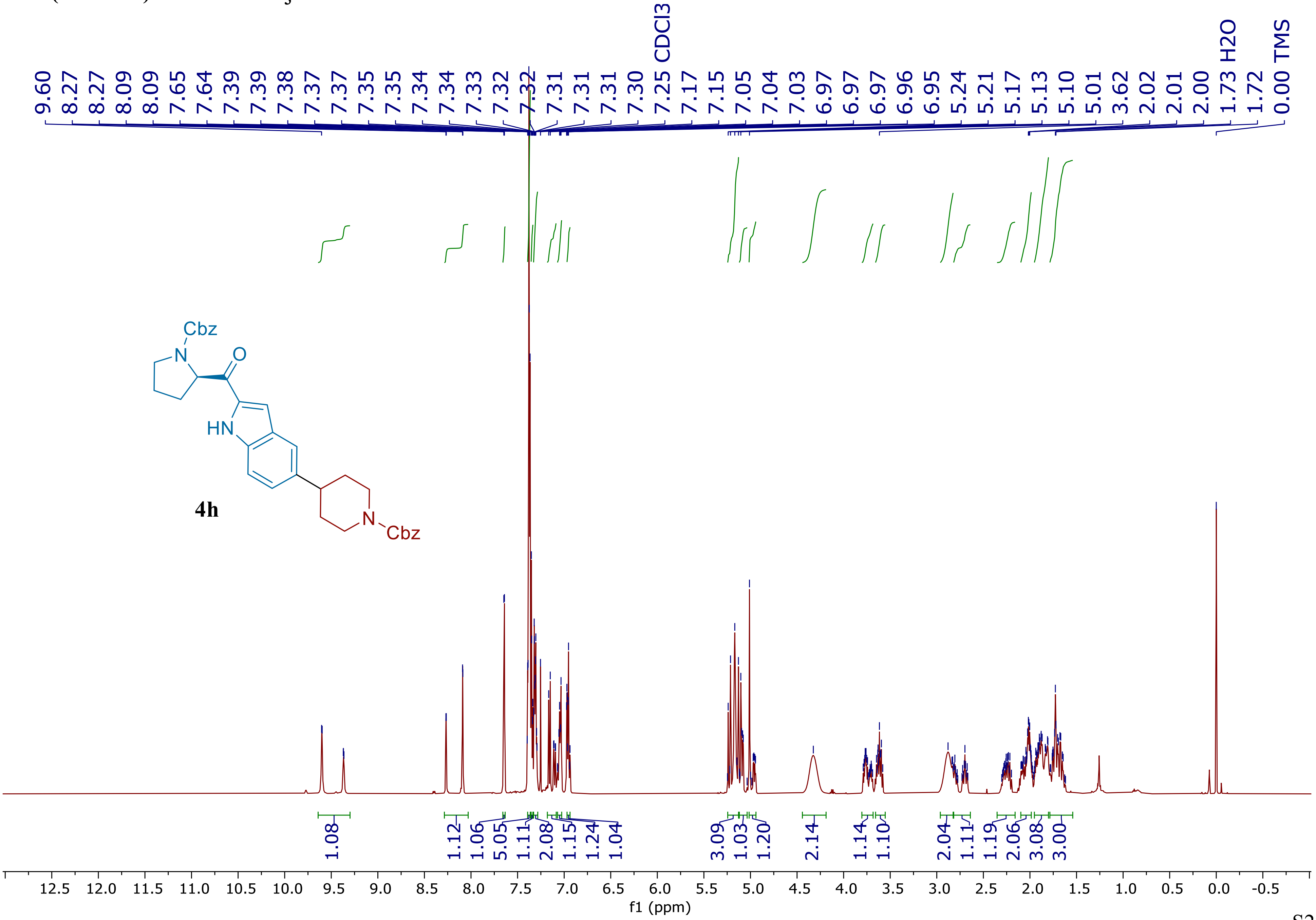

$^{13}C\{^1H\}$ NMR (126 MHz) of 4h in $CDCl_3$

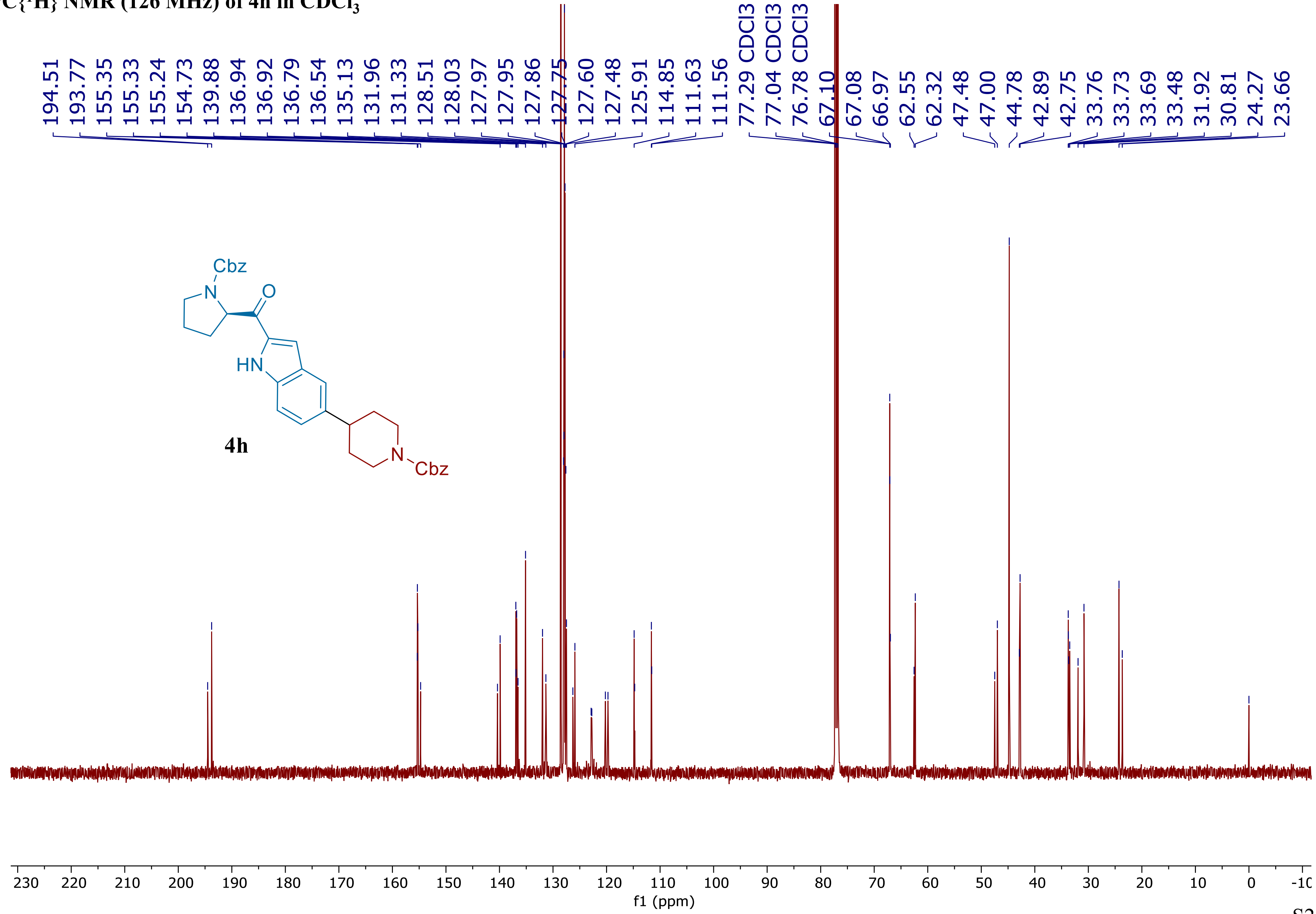

^{1}H NMR (500 MHz) of 4i in $CDCl_3$

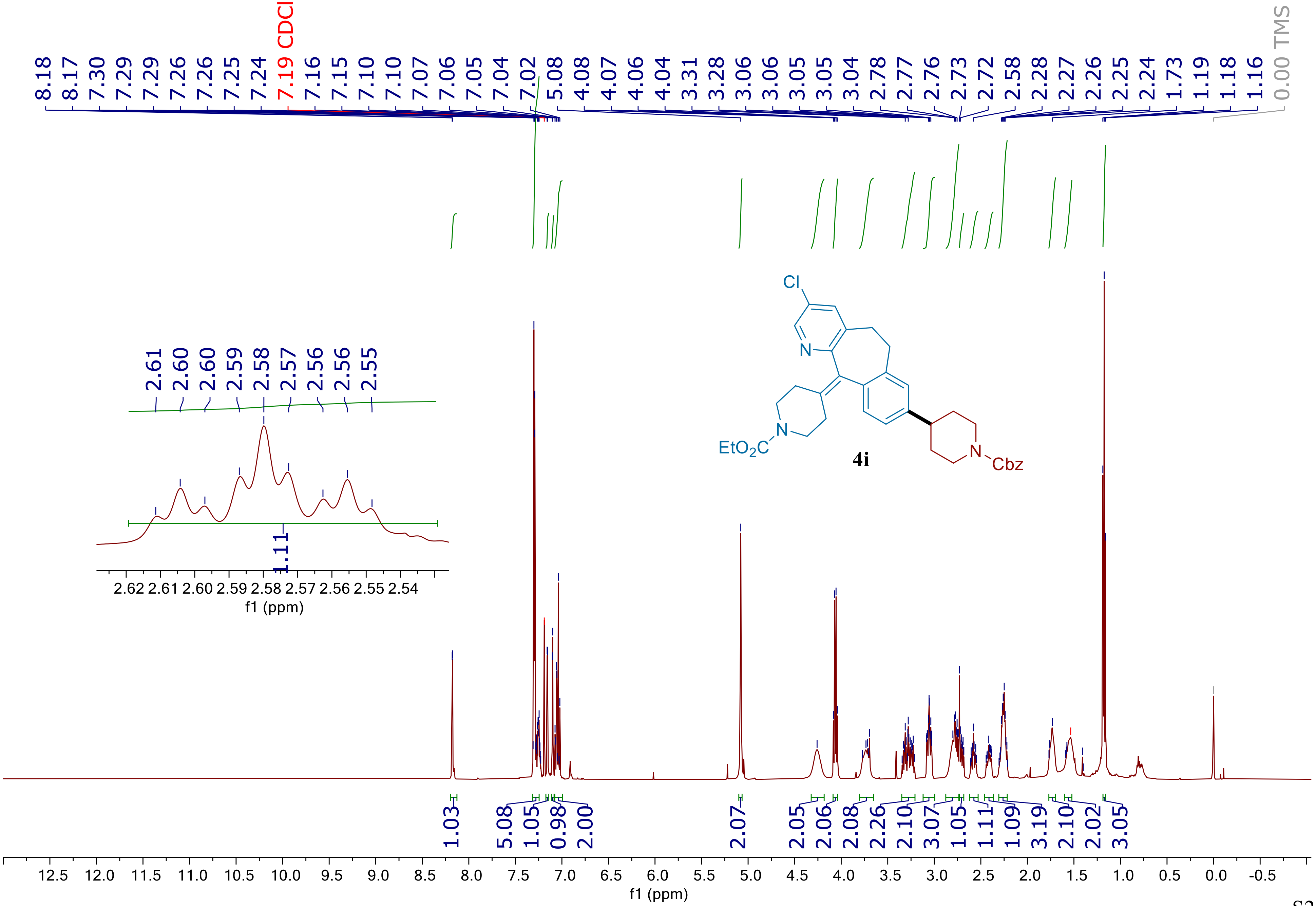

$^{13}C\{^1H\}$ NMR (126 MHz) of 4i in CDCl$_3$

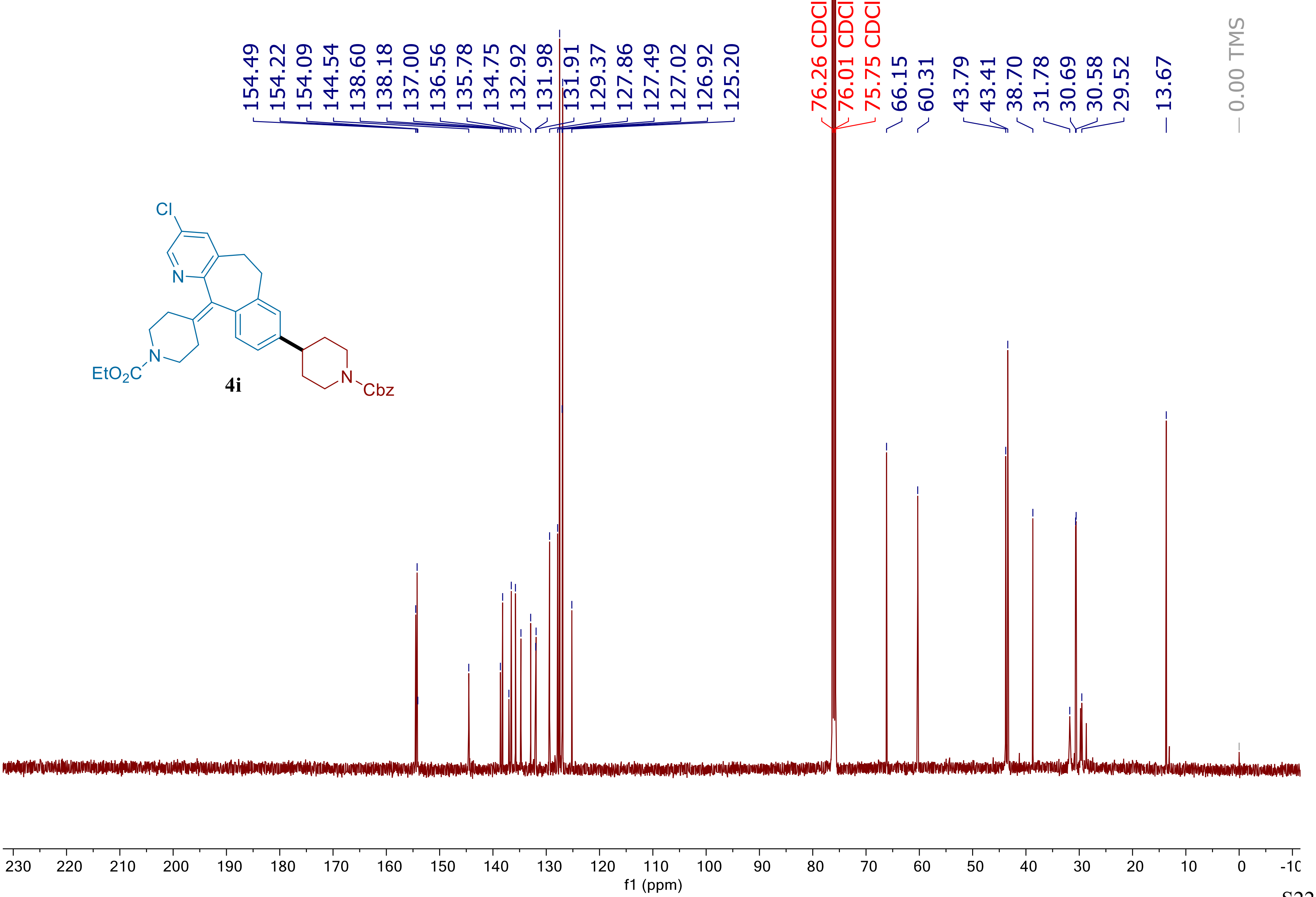

^{1}H NMR (500 MHz) of 4k in $CDCl_3$

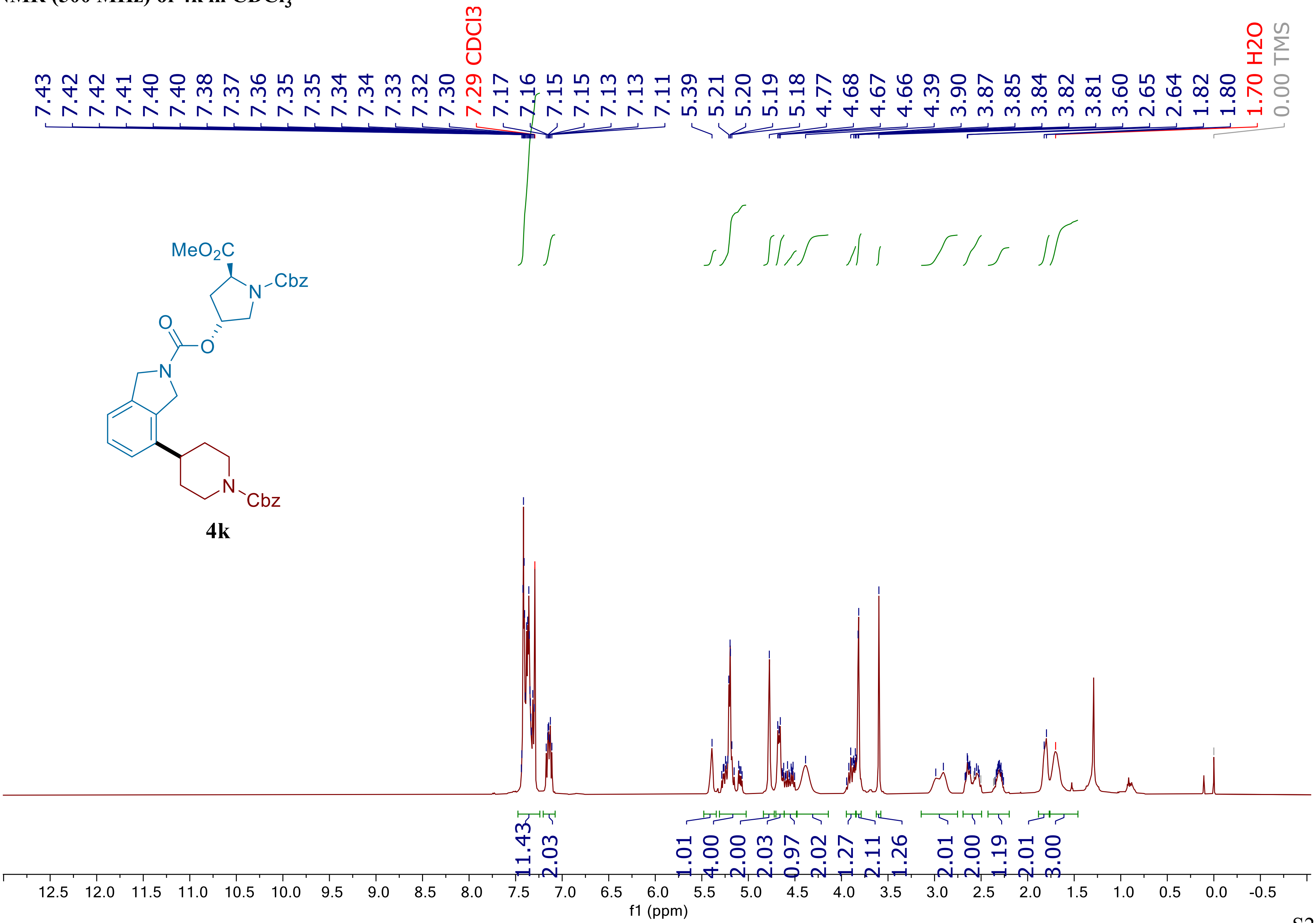

$^{13}C\{^1H\}$ NMR (126 MHz) of 4k in $CDCl_3$

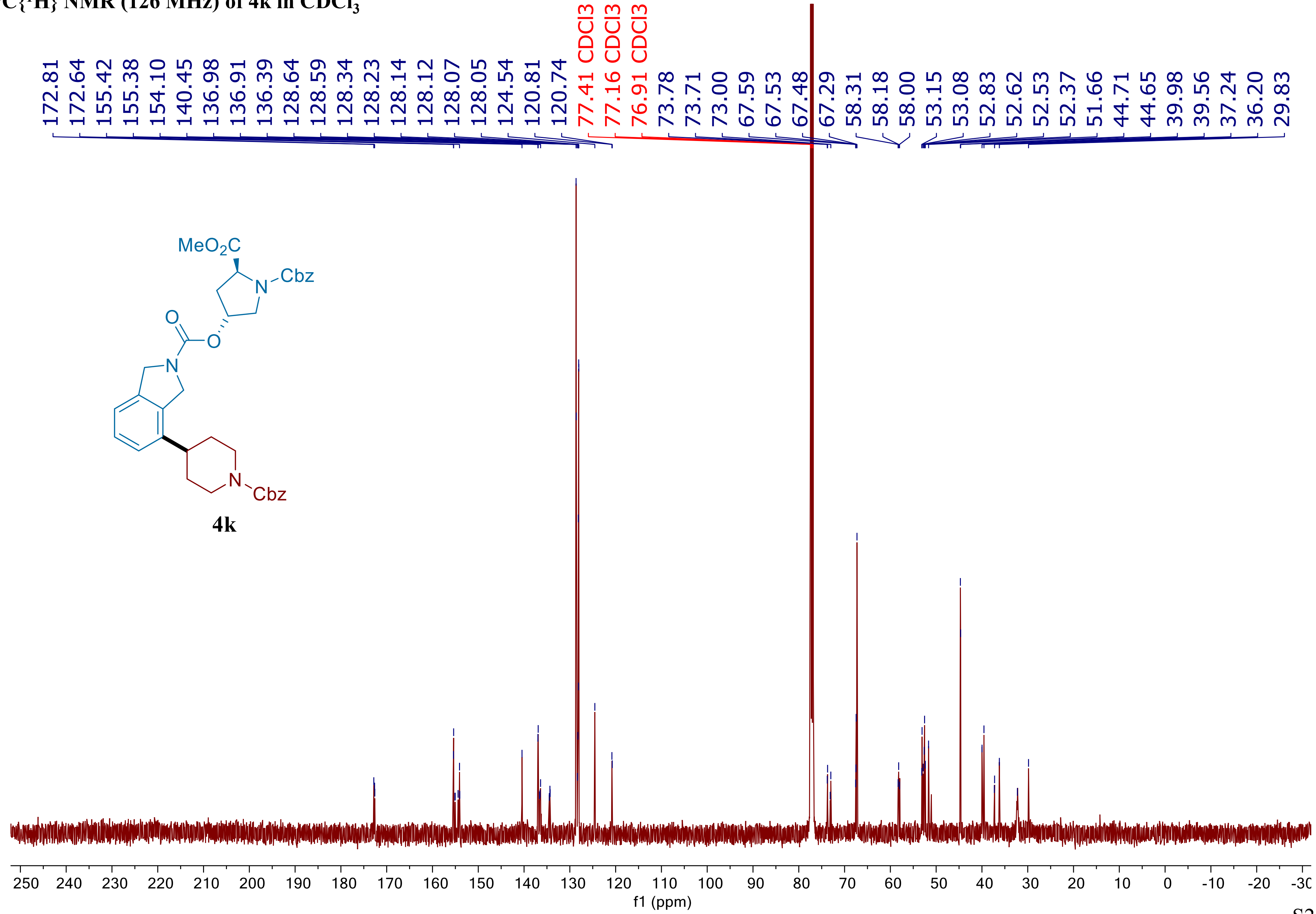

^{1}H NMR (500 MHz) of 4m in $CDCl_3$

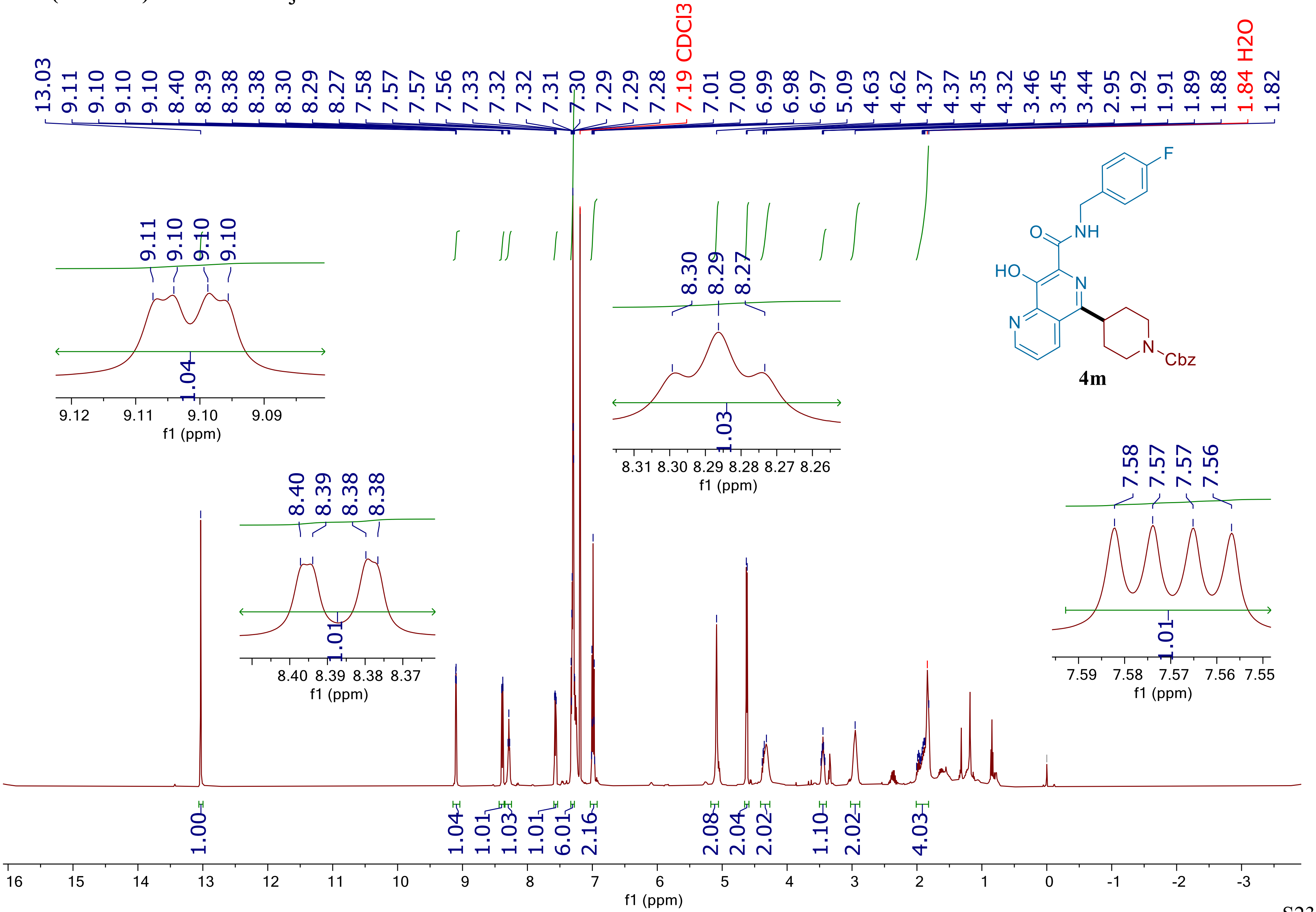

$^{13}C\{^{1}H\}$ NMR (126 MHz) of 4m in $CDCl_3$

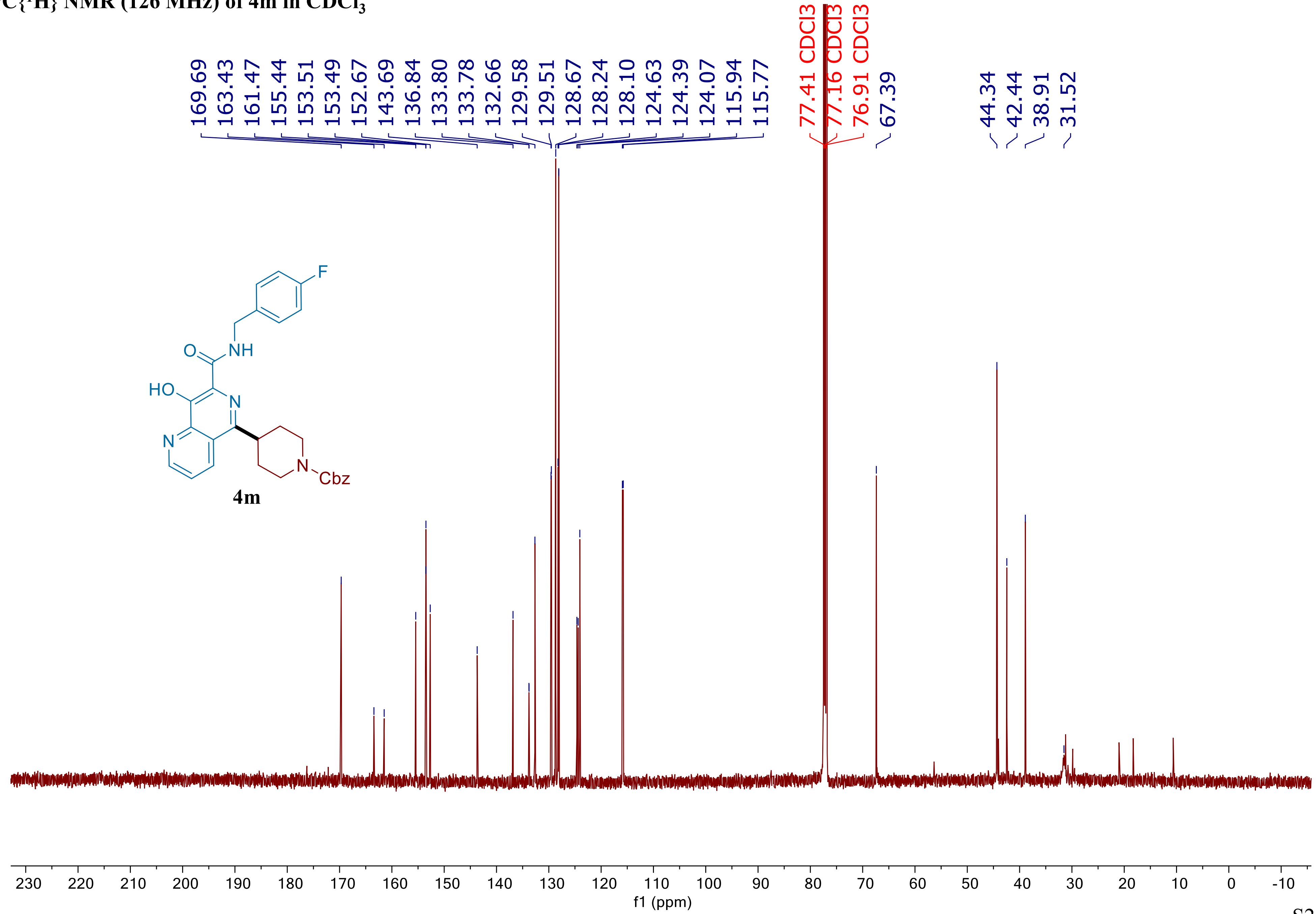

$^{19}F\{^1H\}$ NMR (377 MHz) of **4m** in $CDCl_3$

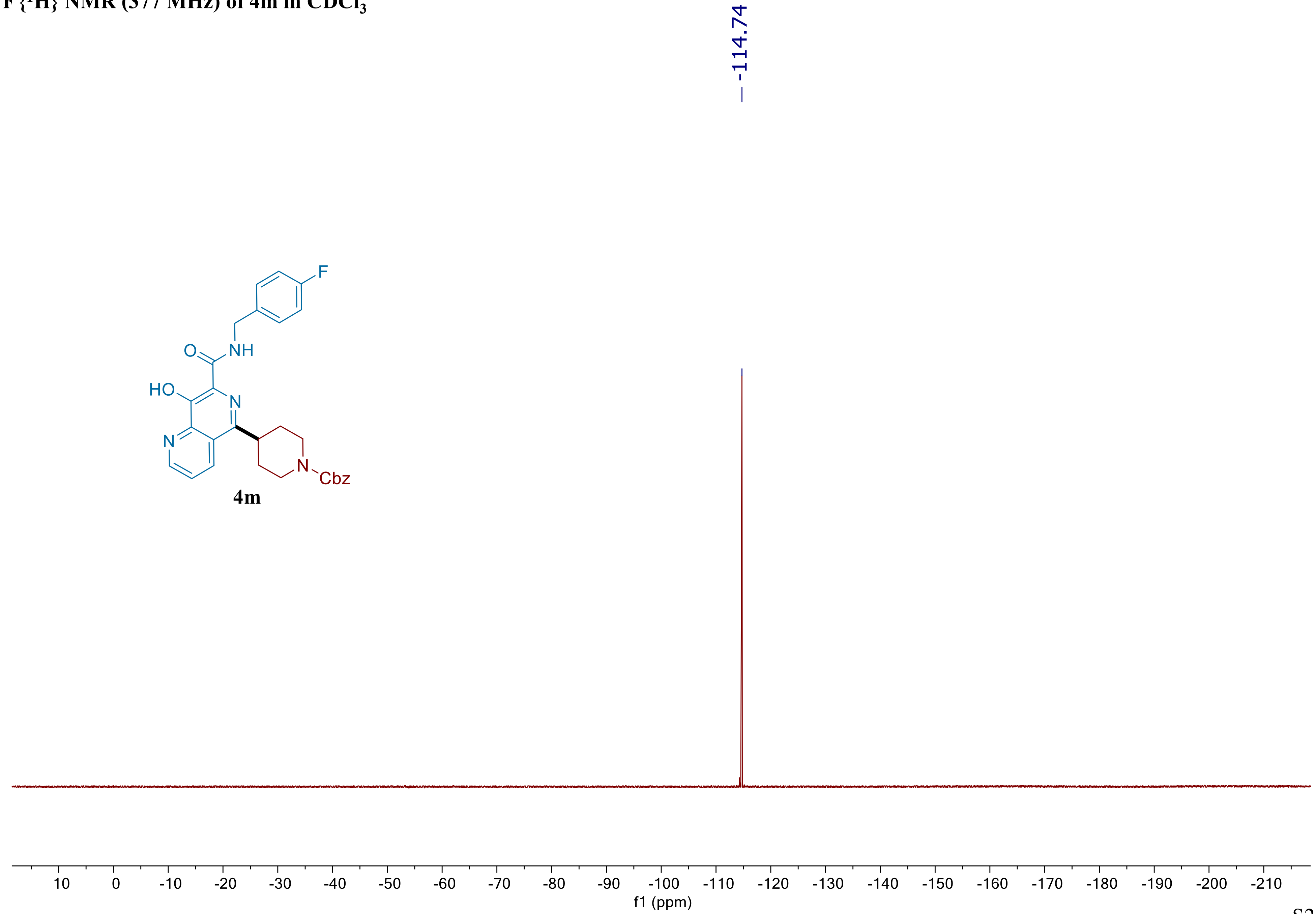

4m

^{1}H NMR (500 MHz) of 4o in $CDCl_3$

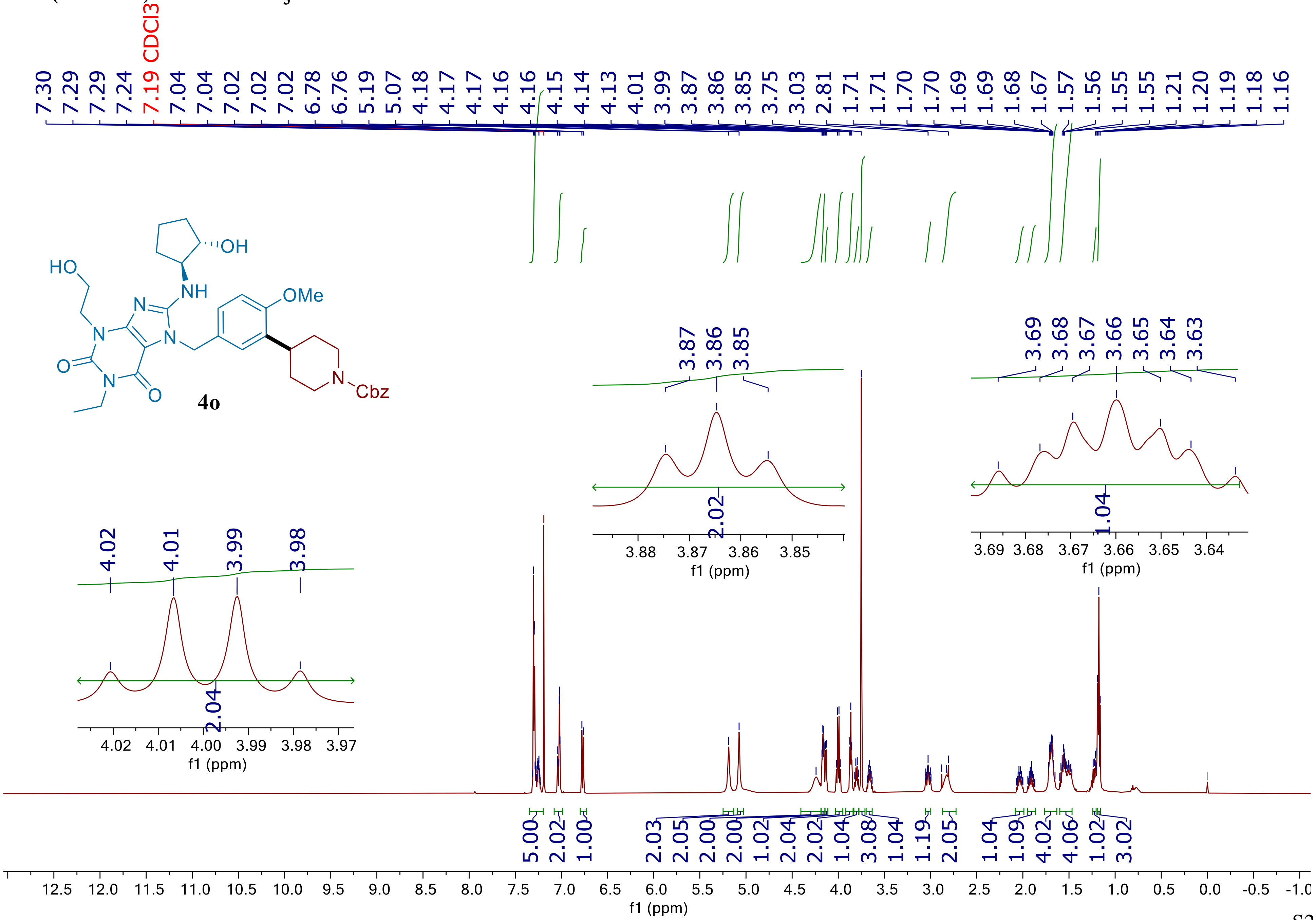

$^{13}C\{^{1}H\}$ NMR (126 MHz) of 4o in $CDCl_3$

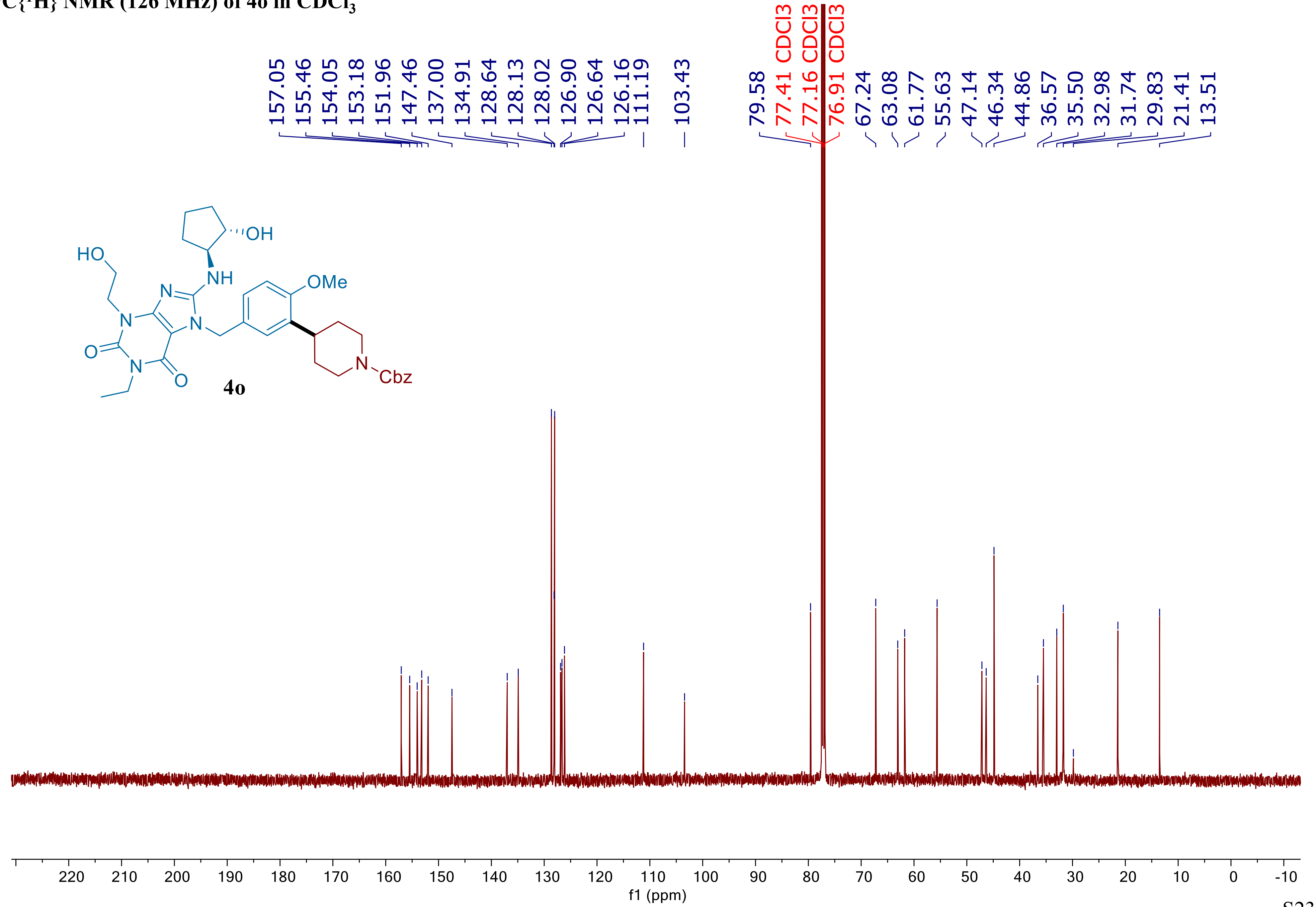

4o

^1H NMR (500 MHz) of 4p in $CDCl_3$

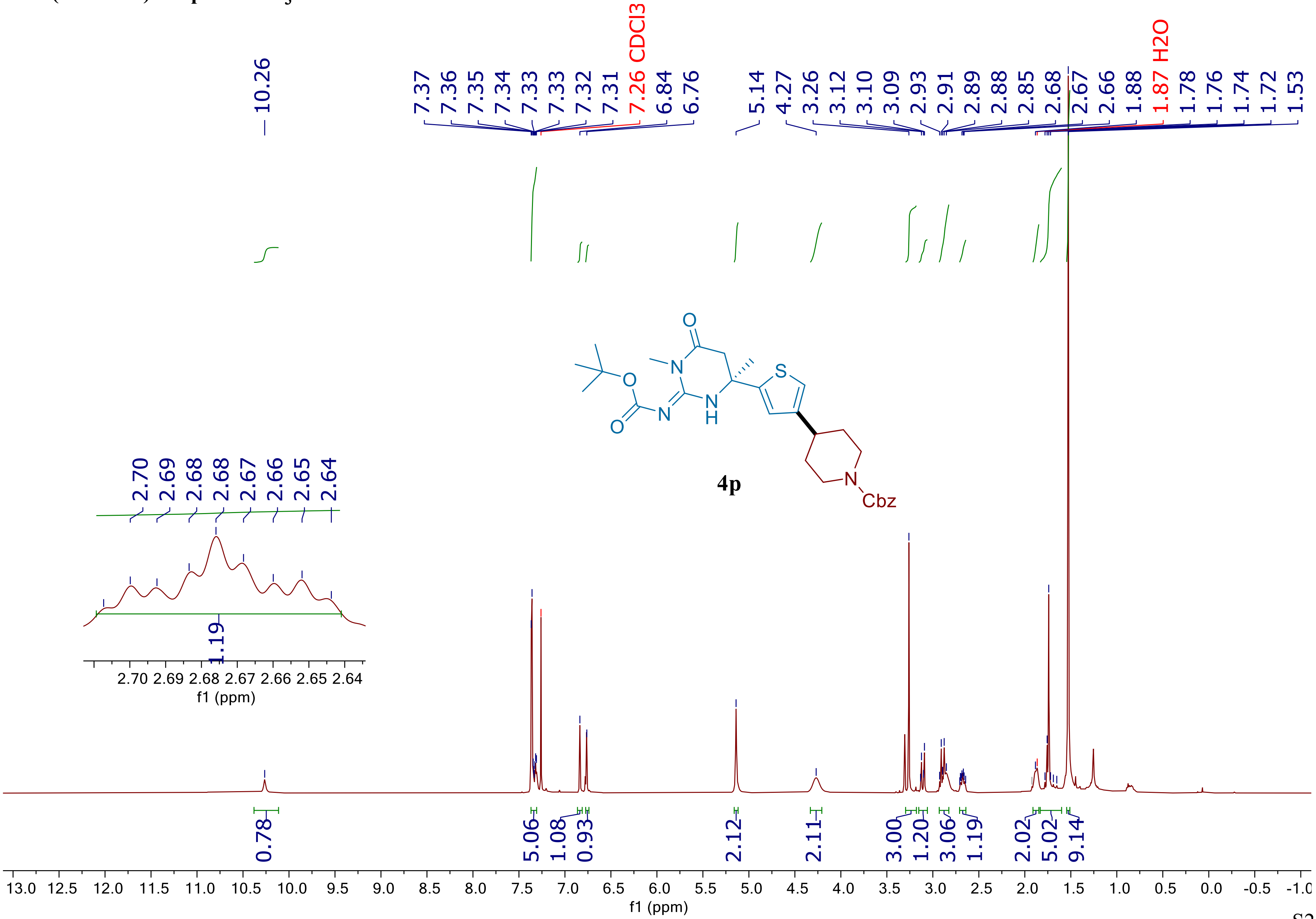

$^{13}C\{^1H\}$ NMR (126 MHz) of 4p in $CDCl_3$

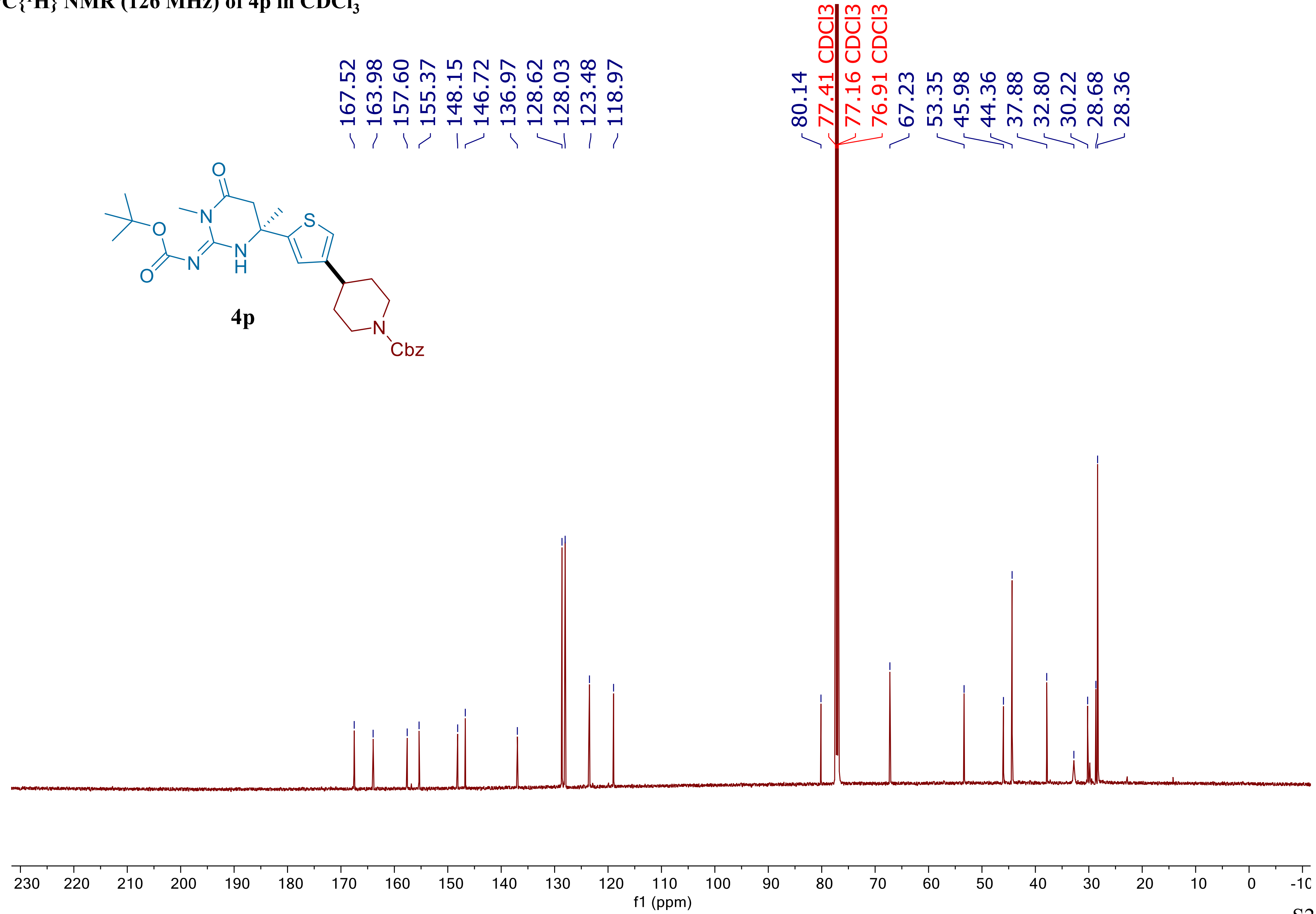

Supplement: Supplementary file 1 [file ja5c18451_si_001.pdf]
